# Antenatal cardiotocography in primary midwife-led care: a budget impact analysis

**DOI:** 10.1136/bmjoq-2023-002578

**Published:** 2024-06-05

**Authors:** Elise Neppelenbroek, Ângela Jornada Ben, Bas S W A Nij Bijvank, Judith E Bosmans, Carola J M Groenen, Ank de Jonge, Corine J M Verhoeven

**Affiliations:** 1 Amsterdam UMC location Vrije Universiteit Amsterdam, Midwifery Science, Amsterdam, Netherlands; 2 Midwifery Academy Amsterdam Groningen, Inholland, Amsterdam, Netherlands; 3 Department of Health Sciences, Faculty of Science, Vrije Universiteit Amsterdam, Amsterdam Public Health Research Institute, Amsterdam, Netherlands; 4 Department of Obstetrics and Gynecology, Isala Women and Children’s Hospital, Zwolle, Netherlands; 5 Amalia Children’s Hospital, Department of Obstetrics and Gynaecology, Radboud University Medical Centre, Nijmegen, Netherlands; 6 Division of Midwifery, School of Health Sciences, University of Nottingham, Nottingham, UK

**Keywords:** Cost-Benefit Analysis, Maternal Health Services, Obstetrics and gynecology, Primary care, Health policy

## Abstract

**Objectives:**

In many countries, the healthcare sector is dealing with important challenges such as increased demand for healthcare services, capacity problems in hospitals and rising healthcare costs. Therefore, one of the aims of the Dutch government is to move care from in-hospital to out-of-hospital care settings. An example of an innovation where care is moved from a more specialised setting to a less specialised setting is the performance of an antenatal cardiotocography (aCTG) in primary midwife-led care. The aim of this study was to assess the budget impact of implementing aCTG for healthy pregnant women in midwife-led care compared with usual obstetrician-led care in the Netherlands.

**Methods:**

A budget impact analysis was conducted to estimate the actual costs and reimbursement of aCTG performed in midwife-led care and obstetrician-led care (ie, base-case analysis) from the Dutch healthcare perspective. Epidemiological and healthcare utilisation data describing both care pathways were obtained from a prospective cohort, survey and national databases. Different implementation rates of aCTG in midwife-led care were explored. A probabilistic sensitivity analysis was conducted to estimate the uncertainty surrounding the budget impact estimates.

**Results:**

Shifting aCTG from obstetrician-led care to midwife-led-care would increase actual costs with €311 763 (97.5% CI €188 574 to €426 072) and €1 247 052 (97.5% CI €754 296 to €1 704 290) for implementation rates of 25% and 100%, respectively, while it would decrease reimbursement with −€7 538 335 (97.5% CI −€10 302 306 to −€4 559 661) and −€30 153 342 (97.5% CI −€41 209 225 to −€18 238 645) for implementation rates of 25% and 100%, respectively. The sensitivity analysis results were consistent with those of the main analysis.

**Conclusions:**

From the Dutch healthcare perspective, we estimated that implementing aCTG in midwife-led care may increase the associated actual costs. At the same time, it might lower the healthcare reimbursement.

**WHAT IS ALREADY KNOWN ON THIS TOPIC**

- E-health facilitates implementing antenatal cardiotocography (aCTGs) in midwife-led care for healthy women with an aCTG indication. A referral to the hospital was indicated after 13.0% aCTGs in midwife-led care, which means that 87% of these women did not need to be referred to the hospital where they normally would have received the aCTG.

**WHAT THIS STUDY ADDS**

- Shifting aCTG to midwife-led care may increase the associated actual costs. At the same time, it might lower the healthcare reimbursement.

**HOW THIS STUDY MIGHT AFFECT RESEARCH, PRACTICE OR POLICY**

- Implementing aCTG in midwife-led care can address a number of important challenges for the healthcare sector, such as the increased demand for healthcare services, capacity problems in hospitals and rising healthcare costs.

## Introduction

 Value-based healthcare has recently emerged as a prominent movement within healthcare. Value-based healthcare aims to improve patient outcomes by coordinating care around individual patients’ values, needs and preferences while efficiently using healthcare resources and eliminating (often harmful) waste.^1^ A basic characteristic is that care is given in the setting with the greatest value: low complex care not in expensive settings (hospitals or specific hospital departments) by expensive care providers (medical specialists and specific nurses) but in settings with lower costs outside the hospital.^2^ In this way, value-based healthcare can be used as a tool to provide the best quality of care for patients and address a number of important challenges for the healthcare sector as well, such as the increased demand for healthcare services and, at the same time, capacity problems in hospitals and rising healthcare costs.^1 3^ Policy of the Dutch government aims at moving care from in-hospital to out-of-hospital care settings, such as the home situation and primary care.^4 5^ An example of a value-based healthcare innovation where care is moved from a more specialised setting to a less specialised setting is the performance of an antenatal cardiotocography (aCTG) in primary midwife-led care (MLC) for specific indications.^6^ Until recently, aCTG was only conducted in obstetrician-led care (OLC) in a hospital setting. Recent technological developments in healthcare facilitated the performance of an aCTG in primary care by a trained midwife using a portable CTG system,^7^ which can be used at the pregnant woman’s home or in the midwifery practice. This is expected to decrease unnecessary referrals and resource use in women with normal aCTG outcomes because only women with a non-reassuring aCTG, who may be at high risk for adverse neonatal outcomes, will be referred to the hospital. Studies showed that referral from primary to secondary care is associated with lower satisfaction among pregnant women due to the lack of continuity of care.^8 9^ It is essential to ensure that the quality of care is not negatively affected when reorganising and shifting tasks and responsibilities. An important aspect of quality of care is safety. When implementing aCTG in primary care, safety and, thus, quality of care may be reduced if primary care midwives are less able to accurately classify aCTGs. However, a previous study showed excellent agreement between primary care midwives, hospital-based midwives, residents and obstetricians in the overall classification of aCTGs in healthy women.^10^ In a prospective study, we evaluated the process maternal and neonatal outcomes of aCTG in MLC (MLC-aCTG). A referral to secondary care was indicated after 13.0% MLC-aCTGs due to non-reassuring aCTGs, ultrasound abnormalities or other reasons, which means that 87% of these women did not need to be referred to the hospital where they normally would have received the aCTG in OLC (OLC-aCTG). The maternal and perinatal outcomes of women who had an MLC-aCTG were in the expected range for a low-risk population in MLC.^11^ Additionally, women were highly satisfied with the care they received.^12^ These outcomes together make MLC-aCTG a promising value-based healthcare innovation. Although we expect that out-of-hospital care reduces healthcare costs, evidence specific to the MLC-aCTG is lacking. Therefore, the aim of this study was to evaluate the budget impact of the implementation of MLC-aCTG for healthy pregnant women compared with OLC-aCTG in the Netherlands at a national level.

## Methods

This study was conducted and reported according to the Dutch guideline for conducting economic evaluations in healthcare^13 14^ and the International Society for Pharmacoeconomics and Outcomes Research (ISPOR) Principles of Good Practice for Budget Impact Analysis.^15^

### Design and target population

A budget impact analysis was conducted to estimate the actual costs and reimbursement of implementing MLC-aCTG (ie, innovation care path) compared with OLC-aCTG (ie, usual care path). The target population included healthy pregnant women receiving antenatal care from a primary care midwife and who were between 28 and 42 weeks gestation with specific aCTG indications, that is, reduced fetal movements, external cephalic version in MLC or postdate pregnancy in the Netherlands.

### Patient and public involvement

Due to the objective of our study, it was not appropriate to involve patients or the public in our research’s design, conduct, reporting or dissemination plans.

### Time horizon and perspective

A 1-year time horizon was used to evaluate the budget impact of implementing MLC-aCTG compared with OLC-aCTG from the Dutch healthcare perspective.

### Care pathways

**Figure 1:**
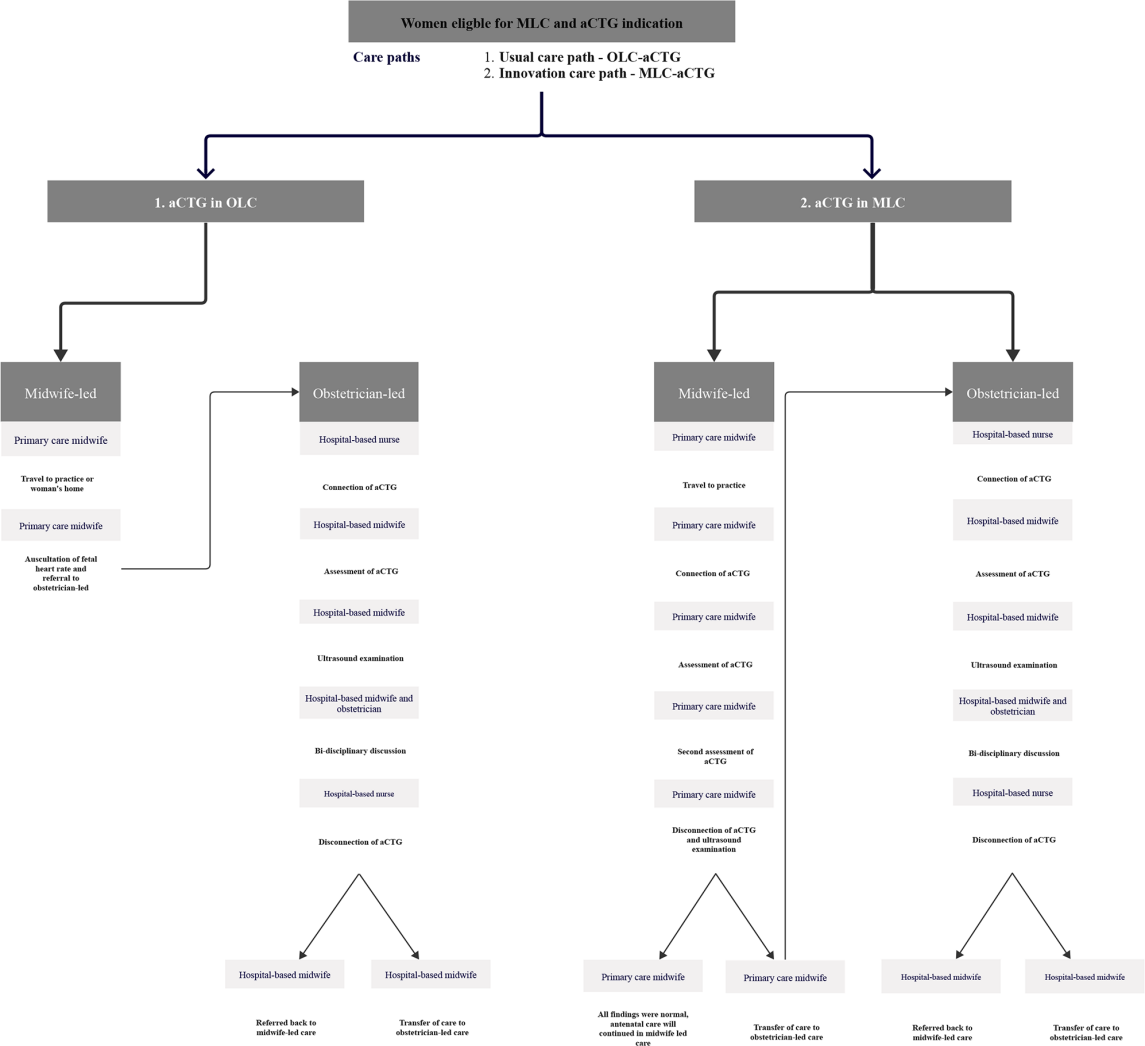
Process mapping care path MLC-aCTG versus OLC-aCTG. MLC-aCTG: midwife-led care antenatal cardiotocograph. OLC-aCTG: obstetrician-led care aCTG.

In the Netherlands, healthy pregnant women receive MLC from primary care midwives. In contrast, those with risk factors (eg, hypertension, diabetes) or complications receive OLC in the hospital from obstetricians, obstetric residents and hospital-based midwives.^16^ These women were outside the scope of this study. Figure 1 shows the process steps of the two care paths compared in this study.

#### Usual care path: OLC-aCTG

In the usual care path, when an aCTG is indicated for a healthy pregnant woman, the primary care midwife refers the woman to OLC for consultation. The woman receives an OLC-aCTG in the hospital. Subsequently, an ultrasound scan is performed to assess fetal growth, amniotic fluid and the presentation of the fetus. Blood pressure is measured as well. When a risk factor or an abnormality arises during these examinations, the care is continued in OLC after transfer of care. If all findings of CTG, ultrasound and blood pressure are normal, the woman is referred back to MLC, where her antenatal care will continue.

#### Innovative care path: MLC-aCTG

In the innovative care path, when an aCTG is indicated for a healthy pregnant woman, the woman is offered an aCTG that is performed and assessed by a primary care midwife either at home, in the midwifery practice, or at a community healthcare centre nearby, using a portable CTG system. If the MLC-aCTG is classified as non-reassuring or of insufficient quality, the woman is immediately referred to OLC for additional evaluation, including a new aCTG. After a reassuring MLC-aCTG, an ultrasound scan is performed in primary care to assess fetal growth, amniotic fluid and presentation of the fetus. Blood pressure is measured as well. If aCTG, ultrasound and blood pressure findings are normal, antenatal care is continued in MLC.

### Data sources

Epidemiological data and healthcare utilisation of MLC-aCTG and OLC-aCTG were based on different data sources.

#### Prospective cohort study

Epidemiological data on the MLC-aCTG care path were obtained from the prospective cohort study investigating the maternal, neonatal and process outcomes.^11^ Data for this study were collected between 1 August 2016 and 31 December 2020, in three regions in the Netherlands (Nijmegen, Zwolle and Amsterdam) during the MLC-aCTG innovation project.

#### Survey

For specific information about the costs in both MLC-aCTG and OLC-aCTG, we developed an online questionnaire in the Castor Electronic Data Capture system.^17^ A link to the questionnaire was sent to healthcare professionals and financial staff working in MLC or OLC in the three regional maternity care networks included in the prospective cohort study between July and October 2020. We asked for details about the healthcare provider’s work setting (eg, midwifery practice, midwifery cooperation, hospital), healthcare capacity (eg, number of aCTGs performed, type of indications of aCTG, workload) and financial information (eg, reimbursed costs, equipment costs and other overhead costs). All data were reported anonymously, and references to the identity of the participants were deleted before analysis.

#### Data-Infrastructure for Parents and Children database

The Data-Infrastructure for Parents and Children (DIAPER) database was used to obtain information about the OLC-aCTG reimbursement. The DIAPER database links data from various sources (Vektis, Perined and Statistics Netherlands) within a secure environment.^18^ DIAPER contains detailed reporting information on reimbursement from health insurers related to pregnancy, childbirth and maternity care (Vektis),^19^ data about the quality of care, health outcomes of pregnant women and newborns (Perined),^20^ and background characteristics of mother and child (Statistics Netherlands).^21^ For the purpose of this study, we only used data from Perined and Vektis for the years 2016–2020 (4 years). All data were anonymised.

### Actual costs

MLC-aCTG actual costs were those incurred by midwifery practices, or at community-based ultrasound centres and were calculated by summing costs related to performing aCTGs and overhead costs (online supplemental material 1, worksheet ‘care pathways costs’). Time costs were calculated by multiplying the prevalence of performing a reassuring and a non-reassuring aCTG by the time spent on an aCTG by the healthcare professionals by their hourly wage (time-driven activity costing). Healthcare professionals’ hourly wages were based on data from the Royal Dutch Organisation of Midwives,^22^ the Dutch Association of Hospitals^23^ and the Dutch guideline for conducting economic evaluations in healthcare.^14^ Hospital admission day costs were based on the Dutch costing manual.^24^ Overhead costs were calculated by summing costs related to equipment, training and quality assessment. Market prices of equipment were first depreciated over 10 years with an interest rate of 4.2% as recommended by the Dutch guideline for conducting economic evaluations in healthcare.^14^ Subsequently, the estimated yearly equipment costs were multiplied by the number of available types of equipment in the prospective cohort and divided by the number of pregnant women in the maternity care network per year. Training costs and quality assessment costs were estimated based on data from the survey and the prospective cohort study (online supplemental material 1, worksheet ‘care pathways costs’). To estimate the actual costs of performing an OLC-aCTG, we used the same approach as for MLC-aCTG. However, to calculate time costs for clinical work, we used hourly wages of healthcare professionals in the hospital (ie, obstetricians, obstetric residents, hospital-based midwives and nurses). Moreover, to account for the temporary admission of the pregnant woman, we estimated admission costs per hour of an admission day (online supplemental material 1, worksheet ‘care pathways costs’). All costs were indexed to 2020.

### Reimbursement

MLC-aCTG reimbursement was defined as the weighted amount reimbursed to health insurance companies according to the Dutch Healthcare Authority when performing an aCTG.^25^ Reimbursement for the MLC-aCTG care path was weighted by the probability of having a reassuring or non-reassuring aCTG (online supplemental material 1, worksheet ‘care pathways costs’).

To estimate the OLC-aCTG reimbursement, we extracted from Perined data of women from 28 weeks of gestation onwards who were referred once to OLC for reduced fetal movements for an aCTG consultation in the hospital and received further care in MLC. We excluded cases if, next to the consultation for reduced fetal movements, there were other consultations with the obstetrician or paediatrician, multiple pregnancies or missing identification numbers for linking Perined data toVektis data. In the Vektis data, reimbursement for a medical specialist was extracted, and cases with missing identification number and parity information were excluded. After applying the inclusion and exclusion criteria, 9822 healthy women remained for the analyses. Subsequently, the Vektis and Perined data files were merged within the secure DIAPER environment by identification number and year of delivery. Descriptive statistics were used to report the OLC-aCTG reimbursement.

### Base-case analysis

The budget impact of implementing MLC-aCTG on a national scale was estimated for three specific indications: reduced fetal movements, external cephalic version in MLC and postdate pregnancy in the Netherlands. The budget impact was estimated using the equation:


$$Budget\;impact=\left(N\times Costs_{MLC-aCTG}\right)-\left(N\times Costs_{OLC-aCTG}\right),$$

Where N represents the estimated number of aCTGs performed per year, and $Costs_{MLC-aCTG\;}$ and $Costs_{OLC-aCTG}$ are the costs per patient per year related to both MLC-aCTG and OLC-aCTG, respectively. N was calculated as the product of the estimated number of healthy pregnant women in the Netherlands in 2020 and the annual prevalence of performing an aCTG for three specific indications. Different MLC-aCTG implementation rates of 25%, 50%, 75% and 100% were explored. For each implementation rate scenario, the expected change in actual costs and reimbursement for the target population in the Netherlands was reported. To estimate the uncertainty surrounding the budget impact estimates, a probabilistic sensitivity analysis was conducted. In the probabilistic sensitivity analysis, a beta distribution was fitted for the prevalence of having an aCTG. Monte Carlo simulations (5000 simulations) were then used to estimate a 95% CI around the budget impact estimate of each implementation rate scenario.^26^

A break-even point analysis was additionally performed to estimate the number of aCTG at which actual costs and reimbursement are equal.^27^ The break-even point was defined as the point where the MLC-aCTG fixed costs (c_fixed: costs of equipment, training, and quality assessment) and the variable MLC-aCTG costs (c_variable: costs of performing reassuring and non-reassuring MLC-aCTG) are equalled by the MLC-aCTG reimbursement (r_MLC-aCTG). The equation represents the Break-even point:


$$Break-even\;point=c\_fixed/\left(r_{MLC-aCTG}-c\_variable\right)$$

Fixed costs for the total estimated number of aCTGs for the Dutch population $\left(N_{aCTG}\right)$ were additionally calculated as represented by the equation below:

$c\_fixed=N_{aCTG}/\left(r_{MLC-aCTG}-c\_variable\right)$

### Sensitivity analyses

We conducted a sensitivity analysis using the data of aCTGs performed for the indication of reduced fetal movements because, in some Dutch regional maternity care networks, aCTG is only performed for this indication. All analyses were conducted in Microsoft Excel (V.2211, 2020).

## Results

### Epidemiological data

In the prospective cohort study, 1795 aCTGs were performed in MLC between 2016 and 2022. Of those aCTGs, 87% and 13% were considered reassuring and non-reassuring, respectively (table 1). In the survey, 33 out of 50 healthcare professionals and 5 out 8 financial employees agreed to participate and gave informed consent. Based on the survey, we estimated the prevalence of performing aCTG for three indications to be 19.8% and for one indication to be 14.8%. We estimated that, on average, a midwife spends 2.1 hours on performing a reassuring aCTG, and 1.8 hours on performing a non-reassuring aCTG. Detailed information on the workload of healthcare professionals to perform an aCTG is presented in table 1.

**Table 1:** Parameters used in the budget impact model

Parameters	Value	Reference
Epidemiological data		
Prevalence of performing a reassuring aCTG	87.0%	^11^
Prevalence of performing a non-reassuring aCTG	13.0%	^11^
Prevalence of performing an aCTG for three indications	19.8%	Online supplemental material1
Prevalence of performing an aCTG for one indication	14.8%	Online supplemental material 1
Number of midwives	138	Online supplemental material 1
Number of aCTG equipment	34	Online supplemental material 1
Number of aCTGs performed per year	1130	Online supplemental material 1
Number of pregnant women in midwife-led care	1816	Online supplemental material 1
Number of healthy pregnant women per year in the Netherlands	144 427	^39^
Resource use		
Total hours spent on performing a reassuring MLC-aCTG by a midwife	2.10	Online supplemental material1
Total hours spent on performing a non-reassuring MLC-aCTG by a midwife	1.80	Online supplemental material 1
Total hours spent on performing an OLC-aCTG by an obstetrician	0.02	Online supplementalmaterial 1
Total hours spent on performing an OLC-aCTG by a medical resident	0.05	Online supplemental material 1
Total hours spent on performing an OLC-aCTG by an obstetrician nurse	0.15	Online supplemental material 1
Total hours spent on preparing an OLC-aCTG by a clinical midwife	0.41	Online supplemental material 1
Average hours using hospital facility when performing an OLC-aCTG	2.00	Online supplementalmaterial 1
Average hours spent on MLC-aCTG quality assessment by a coordinator per year	134	Online supplemental material 1
Average hours spent on MLC-aCTG quality assessment by a midwife per year	1119	Online supplemental material 1
Average hours spent on MLC-aCTG quality assessment by an obstetrician per year	33	Online supplemental material 1
Unit costs		
Hourly wage of a midwife	€53	^22^
Hourly wage of an obstetrician	€125	^14^
Hourly wage of a medical resident	€55	^23^
Hourly wage of an obstetrician nurse	€42	^23^
Hourly wage of a clinical midwife	€53	^22^
Hourly wage of a coordinator	€53	^22^
Admission day—costs per hour	€21	^14^
Training costs per year per midwife	€100	Online supplemental material 1
Equipment costs	€6000	Online supplemental material 1

MLC-aCTG, antenatal cardiotocography in midwife-led care; OLC-aCTG, obstetrician-led care-aCTG.

### Actual and reimbursed costs

Actual costs for healthcare professionals of an MLC-aCTG and an OLC-aCTG summed to €279 and €235, respectively (table 2). According to the Dutch Healthcare Authority, MLC-aCTG reimbursement in 2020 is €280. Due to the percentage of women referred to OLC after a non-reassuring MLC-aCTG, the weighted reimbursement for MLC-aCTG (weighted by the probability of having a reassuring or non-reassuring and subsequently referral) aCTG is €438 (table 2, online supplemental material 1, worksheet ‘care pathways costs’). According to the Vektis data, the mean OLC-aCTG reimbursement was €1492 (SD=€1723), and the median OLC-aCTG reimbursement was €730.

**Table 2:** Actual costs and reimbursement of care pathways

Items	MLC-aCTG	OLC-aCTG
Reassuring aCTG costs	€97	€66
Non-reassuring aCTG costs	€22	€10
Overhead costs: equipment costs	€112	€112
Overhead costs: training costs	€8	€8
Overhead costs: quality assessment costs	€39	€39
Actual costs per aCTG	€279	€235
Weighted reimbursement per aCTG	€438	€1492

Costs are indexed to 2020.

MLC-aCTG, midwife-led care antenatal cardiotocograph; OLC-aCTG, obstetrician-led care aCTG.

### Base-case analysis

The estimated number of MLC-aCTGs performed in the Netherlands in 2020 was 28 597. Shifting aCTG from secondary OLC to primary MLC would lead to an incremental budget impact in terms of actual costs between €311 763 (95% CI €188 574 to €426 072) to 1 247 052 (95% CI €754 296 to €1 704 290) for implementation rates of 25% and 100%, respectively. Reimbursement would decrease by −€7 538 335 (95% CI −€10 302 306 to −€4 559 661) and −€30 153 342 (95% CI −€41 209 225 to −€18 238 645) for implementation rates of 25% and 100%, respectively (table 3, online supplemental material 1, worksheet ‘base-case analysis’). An overview of the costs for 50% and 75% implementation rates is reported in table 3.

**Table 3:** Budget impact results according to different implementation rates of MLC-aCTG

Analysis, n	MLC-aCTG implementation rates	Actual costs	Reimbursed costs	Budget impact: actual costs(95% CI)	Budget impact: reimbursement(95% CI)
Base-case analysis, n=28 597	Reference: 0% (ie, 100% OLC-aCTG)	€6 717 139	€42 666 047		
	25%	€7 028 902	€35 127 711	€311 763(€188 574 to €426 072)	−€7 538 335(−€10 302 306 to −€4559 661)
	50%	€7 340 665	€27 589 376	€623 526(€377 148 to 852 145)	−€15 076 671(−€20 604 613 to −€9 119 322)
	75%	€7 652 428	€20 051 040	€935 289(€565 722 to €1 278 217)	−€22 615 006(−€30 906 919 to −€13 678 984)
	100%	€7 964 191	€12 512 705	€1 247 052(€754 296 to €1 704 290)	−€30 153 342(−€41 209 225 to −€18 238 645)
Sensitivity analysis,n=21 375	Reference: 0% (ie, 100% OLC-aCTG)	€5 020 892	€31 891 792		
	25%	€5 253 927	€26 257 077	€233 035(€149 260 to €372 683)	−€5 634 715(−€9 011 368 to −€3 609 068)
	50%	€5 486 962	€20 622 362	€466 070(€298 520 to €745 366)	−€11 269 431(−€18 022 736 to −€7 218 136)
	75%	€5 719 997	€14 987 646	€699 105(€447 781 to €1 118 049)	−€16 904 146(−€27 034 104 to −€10 827 204)
	100%	€5 953 032	€9 352 931	€932 140(€597 041 to €1 490 733)	−€ 22 538 862(−€36 045 473 to −€14 436 272)

Base-case analysis was based on the prevalence of performing an aCTG with three indications (19.8%).

Sensitivity analysis was based on the prevalence of performing an aCTG with one indication, that is, reduced fetal movements (14.8%).

Costs are indexed to 2020.

MLC-aCTG, midwife-led care antenatal cardiotocograph; OLC-aCTG, obstetrician-led care aCTG.

**Figure 2:**
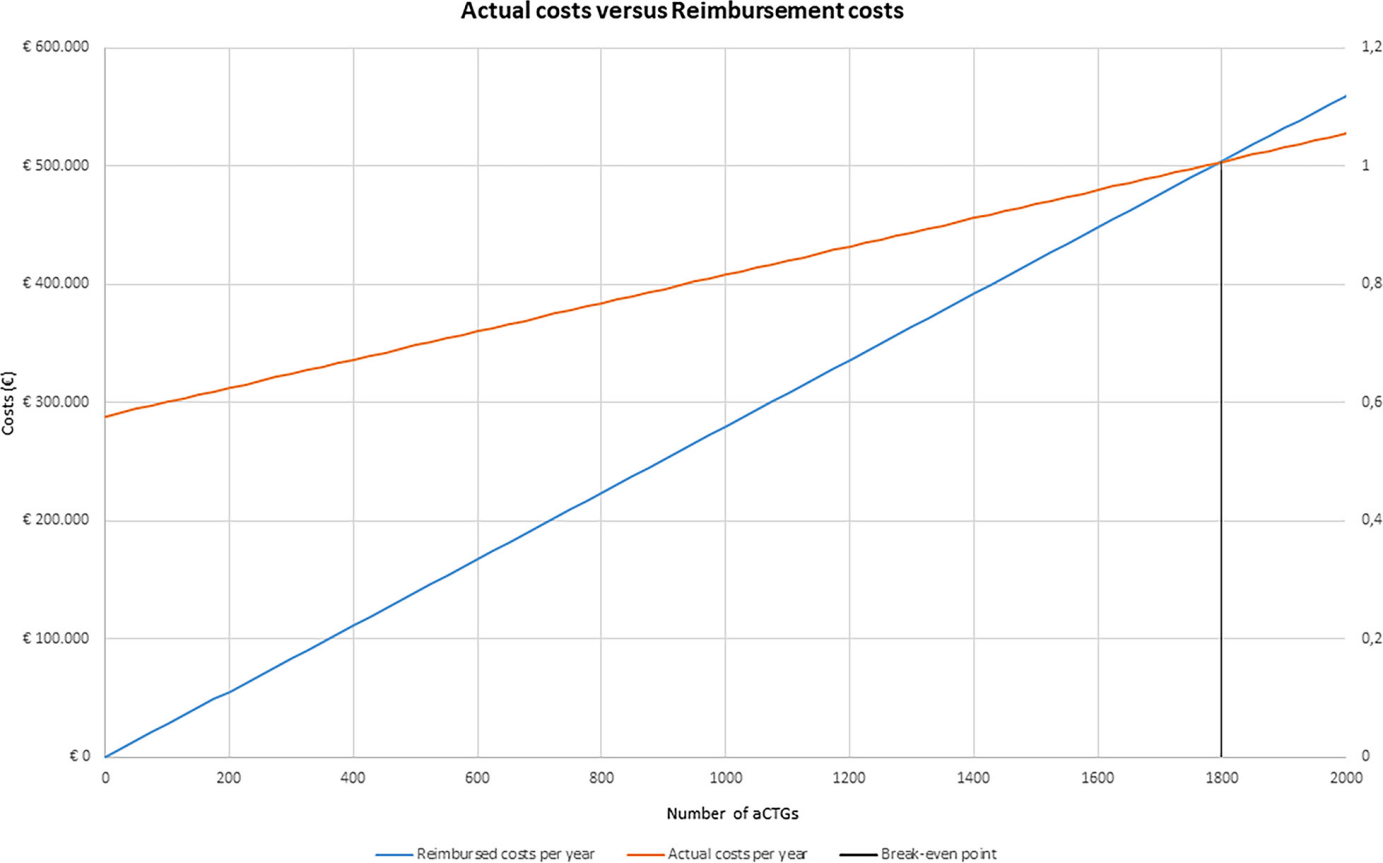
Break-even point actual costs versus reimbursement. The orange line represents the actual costs of MLC-aCTG per year, the blue line represents reimbursed costs of MLC-aCTG per year and black lines represent the number of aCTG at which actual and reimbursed costs are equal. MLC-aCTG, midwife-led care antenatal cardiotocograph; OLC-aCTG, obstetrician-led care aCTG.

The break-even analysis shows that the actual costs of implementing the MLC-aCTG per year in midwifery practices in the three regional maternity care networks together would be €423 741, and the reimbursement would be €316 400. The break-even point in the three regional maternity care networks together at which actual costs and reimbursement are equal was 1799 CTGs, as shown in figure 2 (online supplemental material 1, worksheet ‘costing tool’).

### Sensitivity analysis

The results of the sensitivity analysis were consistent with those obtained from the main analysis, although the budget impact was lower (table 3, online supplemental material 1, worksheet ‘sensitivity analysis).

## Discussion

As far as we know, this is the first study in which the budget impact has been assessed of implementing aCTG in primary care. The results of the base-case analysis suggest that, based on the best information available, implementing MLC-aCTG would result in an increase in actual costs of €311 763 and €1 247 052 for implementation rates of 25% and 100%, respectively, and a decrease in reimbursement of almost €7 538 335 and €30 153 342 million per year, for implementation rates of 25% and 100%, respectively. Our study focused on healthy pregnant women with a specific aCTG indication who received the aCTG in primary care as an alternative to temporary hospital admission during which an aCTG is conducted. Several studies of other innovations in maternity care focussing on out-of-hospital care for high-risk pregnancies had evaluated similar interventions. The trial of Bekker *et al* suggests that home telemonitoring of CTG and blood pressure measurements in pregnancy care is an acceptable alternative to monitoring selected women with complications in hospital, and therefore, has the potential to reduce admissions and costs in obstetric care.^4^ van den Heuvel *et al* reported that using a digital platform for blood pressure and symptom monitoring in antenatal care for high-risk women is associated with lower costs than conventional care while observed maternal and neonatal outcomes are similar.^5^ Our findings showed that the reimbursement of implementing the MLC-aCTG care path might be substantially lower than those of the OLC-aCTG care path and concord with the studies that conclude that out-of-hospital care reduces healthcare costs. However, the cost savings are not reflected in the actual costs incurred by midwifery practices. Compared with obstetrician led, the actual costs of performing an aCTG are higher in MLC. This difference can be explained by the fact that MLC professionals spend more time in attendance during the aCTG, as shown by the time-driven activity-based costing calculation. The break-even analysis shows that income would increase for midwifery practices by performing aCTGs, although at high expenses (eg, in three regional maternity care networks, on average 1799 aCTGs need to be performed together to break even). Hence, midwifery practices must perform sufficient aCTGs to cover costs adequately. The hospital would lose income, but overhead costs like housing costs, personal staff and equipment in obstetrician-led would initially not decrease.^28 29^ The expectation is that hospitals would use the freed-up capacity to provide more care for pregnant women at increased risk. This will further improve the specialised care for those pregnant women who need it most while providing additional revenue for hospitals given the more specialised procedures needed.^28^ However, in the short term, task-shifting aCTG from OLC to MLC would result in a financial burden for hospitals. This means the current reimbursement policy does not support the proposed care path change for maternity care networks.^29^,^33^ As healthcare costs and hospital capacity problems continue to increase worldwide to unsustainable levels, innovations that reduce costs and increase hospital capacity are urgently needed.^3^ Besides, in terms of women’s satisfaction levels, performing aCTGs in primary MLC, thereby improving continuity of care, seems to be a valuable change in the organisation of maternity care in the Netherlands.^12^ However, a fee-for-service payment model is an important barrier to implementing innovations such as MLC-aCTG.^29 32 33^ Within this traditional payment, all maternity care providers (such as gynaecologists and midwives) are paid separately, which hinders collaboration between disciplines. Moreover, fee-for-service models are known to incentivise healthcare professionals to increase the number of medical diagnostic tests and interventions (as long as the price is above marginal costs) and increase overtreatment and low-value care, which is not contributing to the integration of MLC and OLC.^34 35^ Stakeholders in Dutch maternity care agree that a different payment model is needed.^33^ Within the traditional payment model, all maternity care providers (such as obstetricians and midwives) are paid separately, which hinders collaboration between the different disciplines. However, opinions differ about a new payment model’s preferred design and the conditions it should meet.^35^ In terms of further research, it is important to explore the facilitators and barriers for healthcare professionals in MLC and OLC regarding the implementation of MLC-aCTG and explore the possibilities of improving payment models in integrated maternity care. This study has several strengths. First, we analysed the actual costs and reimbursement both at a national level. Second, for the cost calculation, we used time-driven activity-based costing, which helps care providers understand the major cost drivers and find points of action for lowering costs.^3 36^ Third, we performed a probabilistic sensitivity analysis to estimate the uncertainty surrounding our budget impact estimates.^37^ However, some limitations need to be addressed. We used different data sources to estimate the actual costs of MLC-aCTG and OLC-aCTG, including a specifically conducted survey, prospective cohort data and national registry data. Although we tried to be as precise as possible in estimating costs, actual costs in clinical practice may differ from our estimations. Unfortunately, we do not have data for training and CTG equipment costs in OLC, and we assumed in our study equal costs for these components in OLC-aCTG and MLC-aCTG. However, these costs are expected to be lower in OLC, given that the volume of CTGs is higher. This may lead to a lower rate of actual costs of OLC-aCTG. In our analyses, we only focused on the actual costs of performing the aCTG in MLC or OLC without considering a possible impact on the percentage of women that remain in MLC in either of the two care models. Since we expect that more women will stay in MLC when the aCTG is conducted in MLC than in OLC, where the chance of medical interventions is lower,^38^ we expect total childbirth costs to be lower for MLC-aCTG. Future research should address this, for example, in a large prospective cohort study. We chose to focus on the direct material costs, which comprise the majority of expenses incurred in clinical practice. We did not consider the costs of sterilisation, housekeeping, finance, and information and communication technology. Although this might have impacted the costs, we do not expect this would alter our conclusion as this difference will be minimal compared with the costs we have included.

Finally, in this study, we performed a budget impact analysis considering the financial consequences of implementing MLC-aCTG. It was not possible to perform a cost-effectiveness analysis that included outcomes such as safety and women’s satisfaction with the data we had available. However, we have assessed quality of care and women’s satisfaction in separate publications and have shown that there are no differences in the quality of aCTG assessment between primary care midwives, hospital-based midwives, residents and obstetricians.^10^ In addition, our previous work has shown reassuring maternal and perinatal outcomes after MLC-aCTG and high levels of women’s satisfaction with this care.^11 12^ Considering the impact of costs, quality and satisfaction together is crucial when implementing a value-based healthcare innovation such as MLC-aCTG.

### Conclusion

Our findings suggest that shifting aCTG from secondary OLC to primary MLC may increase the associated actual costs for healthcare professionals. At the same time, it might reduce reimbursement. In terms of further research, it is important to explore the facilitators and barriers for healthcare professionals in MLC and OLC regarding the implementation of MLC-aCTG and explore the possibilities of improving payment models in integrated maternity care.

## Supplementary material

10.1136/bmjoq-2023-002578 online supplemental file 1

## Data Availability

Data are available on reasonable request.
