# Supplementary material for: Antenatal cardiotocography in primary midwife-led care: a budget impact analysis
Source: BMJ Open Qual. 2024 Jun 5;13(2):e002578. doi: 10.1136/bmjoq-2023-002578 (PMC11163679; doi:10.1136/bmjoq-2023-002578)

Research question

What is the budget impact of shifting an antenatal cardiotocography (aCTG) from secondary obstetrician-led care to primary midwife-led care?

Traditional care path – OLC-aCTG

Providing an aCTG for healthy pregnant women at secondary obstetrician-led care (OLC-aCTG).

Innovative care path – MLC-aCTG

Providing an aCTG for healthy pregnant women at primary midwife-led care (MLC-aCTG).

Target population

Healthy pregnant women under specific aCTG indications, i.e., reduced fetal movements, external cephalic version, or postdate pregnancy.

Parameters	Value	PSA	Reference
**Epidemiologica data**			
Prevalence of reassuring aCTG	0.87		Prospective cohort study
Prevalence of non-reassuring aCTG	0.13		Prospective cohort study
Prevalence of aCTG under 3 indications	0.20	0.20	Survey
Prevalence of aCTG under 1 indication	0.15	0.15	Survey
Number of midwives	138		Survey
Number of aCTG equipment	34		Survey
Number of aCTGs performed per year	1130		Survey
Number of pregnant women under the midwifery care per year	1816		Survey
Number of healthy pregnant women per year in the Netherlands	144427		Peristat
**Resource use**			
Total hours spent on performing a reassuring MLC-aCTG by a midwife	2.10		Survey
Total hours spent on performing a non-reassuring MLC-aCTG by a midwife	1.80		Survey
Total hours spent on performing an OLC-aCTG by an obstetrician	0.02		Survey
Total hours spent on performing an OLC-aCTG by a medical resident	0.05		Survey
Total hours spent on performing an OLC-aCTG by an O&G nurse	0.15		Survey
Total hours spent on preparing an OLC-aCTG by a clinical midwife	0.41		Survey
Average hours using hospital facility when performing an OLC-aCTG	2.00		Survey
Average hours spent on MLC-aCTG quality assessment by a coordinator per year	134		Survey
Average hours spent on MLC-aCTG quality assessment by a midwife per year	1119		Survey
Average hours spent on MLC-aCTG quality assessment by a obstetrician per year	33		Survey
**Unit costs**			
Hourly wage of a midwife	€ 53		Koninklijke Nederlandse Organisatie van Verloskundigen
Hourly wage of an obstetrician	€ 125		Kostenhandleiding
Hourly wage of a medical resident	€ 55		Nederlandse Vereninging van Ziekenhuizen
Hourly wage of an O&G nurse	€ 42		Nederlandse Vereninging van Ziekenhuizen
Hourly wage of a clinical midwife	€ 53		Koninklijke Nederlandse Organisatie van Verloskundigen
Hourly wage of a coordinator	€ 53		Koninklijke Nederlandse Organisatie van Verloskundigen
Admission day - costs per hour (OLC-aCTG)	€ 21		Kostenhandleiding
Training costs per year per midwife (MLC-aCTG)	€ 100		Survey
Equipment costs	€ 6,000		Market price
Reimbursement MLC-aCTG	€ 280		Nederlandse Zorgautoriteit
Reimbursement OLC-aCTG	€ 1,492		DIAPER database

Costs of care pathways (€, 2020)	MLC-aCTG	OLC-aCTG
Reassuring aCTG costs	€ 97	€ 66
Non-reassuring aCTG costs	€ 22	€ 10
Overhead costs: Equipment costs	€ 112	€ 112
Overhead costs: Training costs	€ 8	€ 8
Overhead costs: Quality assessment costs	€ 39	€ 39
**Actual costs per aCTG**	€ 279	€ 235
**Actual reimbursement per aCTG**	€ 438	€ 1,492

Base-case analysis

Number of aCTGs under three indications in 2020	28597

Implementation rates of MLC-aCTG	Actual costs	Budget impact: actual costs
0% (reference scenario OLC-aCTG)	€ 6,717,139	NA
25%	€ 7,028,902	€ 311,763
50%	€ 7,340,665	€ 623,526
75%	€ 7,652,428	€ 935,289
100%	€ 7,964,191	€ 1,247,052

	Reimbursed costs	Budget impact: reimbursed costs
0% (reference scenario OLC-aCTG)	€ 42,666,047	NA
25%	€ 35,127,711	-€ 7,538,335
50%	€ 27,589,376	-€ 15,076,671
75%	€ 20,051,040	-€ 22,615,006
100%	€ 12,512,705	-€ 30,153,342

PSA	0
Prev	0.20

		Actual costs by implementation rate				Reimbursed costs by implementation rate			
Iterations	Prevalence	25%	50%	75%	100%	25%	50%	75%	100%
1	0.20	€ 311,763	€ 623,526	€ 935,289	##########	-€ 7,538,335	-€ 15,076,671	-€ 22,615,006	-€ 30,153,342
2	0.27	€ 420,420	€ 840,841	##########	##########	-€ 10,165,637	-€ 20,331,275	-€ 30,496,912	-€ 40,662,549
3	0.28	€ 440,789	€ 881,578	##########	##########	-€ 10,658,146	-€ 21,316,292	-€ 31,974,438	-€ 42,632,584
4	0.18	€ 283,387	€ 566,775	€ 850,162	##########	-€ 6,852,222	-€ 13,704,443	-€ 20,556,665	-€ 27,408,886
5	0.21	€ 338,216	€ 676,433	##########	##########	-€ 8,177,973	-€ 16,355,947	-€ 24,533,920	-€ 32,711,893
6	0.15	€ 232,447	€ 464,894	€ 697,342	€ 929,789	-€ 5,620,505	-€ 11,241,010	-€ 16,861,514	-€ 22,482,019
7	0.24	€ 384,521	€ 769,043	##########	##########	-€ 9,297,615	-€ 18,595,230	-€ 27,892,846	-€ 37,190,461
8	0.15	€ 236,021	€ 472,042	€ 708,063	€ 944,084	-€ 5,706,915	-€ 11,413,830	-€ 17,120,745	-€ 22,827,660
9	0.25	€ 387,446	€ 774,891	##########	##########	-€ 9,368,320	-€ 18,736,641	-€ 28,104,961	-€ 37,473,282
10	0.14	€ 214,597	€ 429,195	€ 643,792	€ 858,389	-€ 5,188,899	-€ 10,377,797	-€ 15,566,696	-€ 20,755,594
11	0.15	€ 234,636	€ 469,272	€ 703,908	€ 938,545	-€ 5,673,432	-€ 11,346,864	-€ 17,020,296	-€ 22,693,728
12	0.15	€ 233,646	€ 467,291	€ 700,937	€ 934,583	-€ 5,649,483	-€ 11,298,966	-€ 16,948,450	-€ 22,597,933
13	0.17	€ 272,188	€ 544,377	€ 816,565	##########	-€ 6,581,434	-€ 13,162,868	-€ 19,744,302	-€ 26,325,736
14	0.20	€ 310,267	€ 620,534	€ 930,801	##########	-€ 7,502,163	-€ 15,004,327	-€ 22,506,490	-€ 30,008,654
15	0.24	€ 381,079	€ 762,158	##########	##########	-€ 9,214,375	-€ 18,428,750	-€ 27,643,126	-€ 36,857,501
16	0.15	€ 241,447	€ 482,894	€ 724,340	€ 965,787	-€ 5,838,111	-€ 11,676,222	-€ 17,514,333	-€ 23,352,444
17	0.14	€ 226,994	€ 453,988	€ 680,982	€ 907,975	-€ 5,488,644	-€ 10,977,288	-€ 16,465,933	-€ 21,954,577
18	0.22	€ 346,432	€ 692,864	##########	##########	-€ 8,376,624	-€ 16,753,248	-€ 25,129,872	-€ 33,506,495
19	0.20	€ 315,890	€ 631,780	€ 947,670	##########	-€ 7,638,129	-€ 15,276,258	-€ 22,914,387	-€ 30,552,516
20	0.21	€ 323,668	€ 647,336	€ 971,004	##########	-€ 7,826,194	-€ 15,652,389	-€ 23,478,583	-€ 31,304,778
21	0.16	€ 245,248	€ 490,496	€ 735,743	€ 980,991	-€ 5,930,019	-€ 11,860,037	-€ 17,790,056	-€ 23,720,075
22	0.16	€ 258,855	€ 517,710	€ 776,565	##########	-€ 6,259,039	-€ 12,518,079	-€ 18,777,118	-€ 25,036,157
23	0.14	€ 228,226	€ 456,452	€ 684,678	€ 912,903	-€ 5,518,434	-€ 11,036,867	-€ 16,555,301	-€ 22,073,735
24	0.26	€ 410,156	€ 820,312	##########	##########	-€ 9,917,456	-€ 19,834,911	-€ 29,752,367	-€ 39,669,822
25	0.22	€ 349,320	€ 698,639	##########	##########	-€ 8,446,447	-€ 16,892,893	-€ 25,339,340	-€ 33,785,787
26	0.20	€ 309,573	€ 619,145	€ 928,718	##########	-€ 7,485,372	-€ 14,970,744	-€ 22,456,116	-€ 29,941,488
27	0.24	€ 370,985	€ 741,970	##########	##########	-€ 8,970,307	-€ 17,940,613	-€ 26,910,920	-€ 35,881,227
28	0.21	€ 337,522	€ 675,044	##########	##########	-€ 8,161,176	-€ 16,322,352	-€ 24,483,527	-€ 32,644,703
29	0.16	€ 255,717	€ 511,434	€ 767,150	##########	-€ 6,183,157	-€ 12,366,314	-€ 18,549,471	-€ 24,732,628
30	0.14	€ 227,583	€ 455,167	€ 682,750	€ 910,333	-€ 5,502,897	-€ 11,005,795	-€ 16,508,692	-€ 22,011,590
31	0.27	€ 421,518	€ 843,036	##########	##########	-€ 10,192,175	-€ 20,384,350	-€ 30,576,525	-€ 40,768,700
32	0.15	€ 240,828	€ 481,656	€ 722,484	€ 963,312	-€ 5,823,148	-€ 11,646,296	-€ 17,469,444	-€ 23,292,593
33	0.25	€ 398,686	€ 797,372	##########	##########	-€ 9,640,115	-€ 19,280,229	-€ 28,920,344	-€ 38,560,459
34	0.15	€ 236,461	€ 472,923	€ 709,384	€ 945,846	-€ 5,717,568	-€ 11,435,135	-€ 17,152,703	-€ 22,870,271
35	0.26	€ 409,318	€ 818,635	##########	##########	-€ 9,897,177	-€ 19,794,353	-€ 29,691,530	-€ 39,588,706
36	0.18	€ 281,576	€ 563,152	€ 844,728	##########	-€ 6,808,426	-€ 13,616,853	-€ 20,425,279	-€ 27,233,705
37	0.21	€ 329,951	€ 659,902	€ 989,853	##########	-€ 7,978,118	-€ 15,956,237	-€ 23,934,355	-€ 31,912,473
38	0.22	€ 349,265	€ 698,530	##########	##########	-€ 8,445,121	-€ 16,890,241	-€ 25,335,362	-€ 33,780,483
39	0.17	€ 264,572	€ 529,144	€ 793,716	##########	-€ 6,397,275	-€ 12,794,549	-€ 19,191,824	-€ 25,589,099
40	0.17	€ 274,509	€ 549,018	€ 823,527	##########	-€ 6,637,545	-€ 13,275,090	-€ 19,912,635	-€ 26,550,181
41	0.15	€ 230,510	€ 461,020	€ 691,530	€ 922,040	-€ 5,573,664	-€ 11,147,329	-€ 16,720,993	-€ 22,294,658
42	0.24	€ 372,437	€ 744,873	##########	##########	-€ 9,005,406	-€ 18,010,811	-€ 27,016,217	-€ 36,021,623
43	0.15	€ 240,831	€ 481,663	€ 722,494	€ 963,326	-€ 5,823,233	-€ 11,646,466	-€ 17,469,699	-€ 23,292,932
44	0.15	€ 229,895	€ 459,789	€ 689,684	€ 919,578	-€ 5,558,782	-€ 11,117,565	-€ 16,676,347	-€ 22,235,130
45	0.15	€ 231,627	€ 463,254	€ 694,880	€ 926,507	-€ 5,600,668	-€ 11,201,336	-€ 16,802,004	-€ 22,402,672
46	0.17	€ 265,739	€ 531,478	€ 797,217	##########	-€ 6,425,491	-€ 12,850,983	-€ 19,276,474	-€ 25,701,965
47	0.22	€ 349,394	€ 698,787	##########	##########	-€ 8,448,234	-€ 16,896,467	-€ 25,344,701	-€ 33,792,935
48	0.15	€ 232,426	€ 464,853	€ 697,279	€ 929,706	-€ 5,620,001	-€ 11,240,002	-€ 16,860,003	-€ 22,480,004
49	0.15	€ 239,231	€ 478,462	€ 717,693	€ 956,924	-€ 5,784,537	-€ 11,569,073	-€ 17,353,610	-€ 23,138,146
50	0.21	€ 333,008	€ 666,015	€ 999,023	##########	-€ 8,052,026	-€ 16,104,052	-€ 24,156,079	-€ 32,208,105
51	0.24	€ 382,658	€ 765,316	##########	##########	-€ 9,252,559	-€ 18,505,118	-€ 27,757,677	-€ 37,010,237
52	0.16	€ 258,148	€ 516,296	€ 774,444	##########	-€ 6,241,941	-€ 12,483,883	-€ 18,725,824	-€ 24,967,765
53	0.17	€ 263,747	€ 527,494	€ 791,240	##########	-€ 6,377,319	-€ 12,754,639	-€ 19,131,958	-€ 25,509,277
54	0.24	€ 381,205	€ 762,410	##########	##########	-€ 9,217,422	-€ 18,434,845	-€ 27,652,267	-€ 36,869,690
55	0.20	€ 311,213	€ 622,425	€ 933,638	##########	-€ 7,525,031	-€ 15,050,062	-€ 22,575,093	-€ 30,100,124
56	0.16	€ 252,814	€ 505,627	€ 758,441	##########	-€ 6,112,958	-€ 12,225,916	-€ 18,338,874	-€ 24,451,832
57	0.20	€ 319,772	€ 639,543	€ 959,315	##########	-€ 7,731,980	-€ 15,463,960	-€ 23,195,941	-€ 30,927,921
58	0.15	€ 233,725	€ 467,450	€ 701,175	€ 934,900	-€ 5,651,402	-€ 11,302,805	-€ 16,954,207	-€ 22,605,609
59	0.21	€ 334,182	€ 668,364	##########	##########	-€ 8,080,427	-€ 16,160,855	-€ 24,241,282	-€ 32,321,709
60	0.14	€ 221,859	€ 443,719	€ 665,578	€ 887,438	-€ 5,364,496	-€ 10,728,991	-€ 16,093,487	-€ 21,457,982
61	0.20	€ 308,179	€ 616,357	€ 924,536	##########	-€ 7,451,666	-€ 14,903,332	-€ 22,354,997	-€ 29,806,663
62	0.14	€ 213,566	€ 427,131	€ 640,697	€ 854,262	-€ 5,163,952	-€ 10,327,904	-€ 15,491,856	-€ 20,655,807
63	0.15	€ 243,479	€ 486,958	€ 730,437	€ 973,916	-€ 5,887,251	-€ 11,774,502	-€ 17,661,753	-€ 23,549,004
64	0.20	€ 312,735	€ 625,470	€ 938,204	##########	-€ 7,561,834	-€ 15,123,669	-€ 22,685,503	-€ 30,247,337
65	0.19	€ 304,907	€ 609,813	€ 914,720	##########	-€ 7,372,551	-€ 14,745,103	-€ 22,117,654	-€ 29,490,206
66	0.23	€ 361,726	€ 723,452	##########	##########	-€ 8,746,426	-€ 17,492,853	-€ 26,239,279	-€ 34,985,705
67	0.10	€ 152,201	€ 304,403	€ 456,604	€ 608,806	-€ 3,680,187	-€ 7,360,373	-€ 11,040,560	-€ 14,720,747
68	0.16	€ 250,285	€ 500,570	€ 750,856	##########	-€ 6,051,822	-€ 12,103,644	-€ 18,155,466	-€ 24,207,289
69	0.21	€ 323,865	€ 647,730	€ 971,595	##########	-€ 7,830,959	-€ 15,661,917	-€ 23,492,876	-€ 31,323,835
70	0.19	€ 295,998	€ 591,997	€ 887,995	##########	-€ 7,157,151	-€ 14,314,303	-€ 21,471,454	-€ 28,628,606
71	0.25	€ 386,924	€ 773,849	##########	##########	-€ 9,355,719	-€ 18,711,437	-€ 28,067,156	-€ 37,422,874
72	0.22	€ 342,032	€ 684,064	##########	##########	-€ 8,270,228	-€ 16,540,456	-€ 24,810,684	-€ 33,080,911
73	0.13	€ 212,440	€ 424,879	€ 637,319	€ 849,758	-€ 5,136,725	-€ 10,273,449	-€ 15,410,174	-€ 20,546,899
74	0.20	€ 309,511	€ 619,022	€ 928,533	##########	-€ 7,483,887	-€ 14,967,774	-€ 22,451,662	-€ 29,935,549
75	0.29	€ 453,399	€ 906,798	##########	##########	-€ 10,963,052	-€ 21,926,103	-€ 32,889,155	-€ 43,852,207
76	0.29	€ 463,561	€ 927,121	##########	##########	-€ 11,208,758	-€ 22,417,517	-€ 33,626,275	-€ 44,835,033
77	0.29	€ 459,294	€ 918,588	##########	##########	-€ 11,105,599	-€ 22,211,197	-€ 33,316,796	-€ 44,422,394
78	0.18	€ 289,080	€ 578,161	€ 867,241	##########	-€ 6,989,879	-€ 13,979,757	-€ 20,969,636	-€ 27,959,514
79	0.17	€ 265,492	€ 530,983	€ 796,475	##########	-€ 6,419,508	-€ 12,839,016	-€ 19,258,524	-€ 25,678,031
80	0.17	€ 260,394	€ 520,788	€ 781,182	##########	-€ 6,296,251	-€ 12,592,502	-€ 18,888,753	-€ 25,185,004
81	0.18	€ 281,246	€ 562,493	€ 843,739	##########	-€ 6,800,452	-€ 13,600,904	-€ 20,401,356	-€ 27,201,808
82	0.23	€ 359,400	€ 718,799	##########	##########	-€ 8,690,175	-€ 17,380,350	-€ 26,070,525	-€ 34,760,700
83	0.16	€ 246,096	€ 492,191	€ 738,287	€ 984,383	-€ 5,950,521	-€ 11,901,042	-€ 17,851,564	-€ 23,802,085
84	0.13	€ 204,187	€ 408,374	€ 612,561	€ 816,749	-€ 4,937,185	-€ 9,874,370	-€ 14,811,555	-€ 19,748,740
85	0.21	€ 328,783	€ 657,565	€ 986,348	##########	-€ 7,949,865	-€ 15,899,731	-€ 23,849,596	-€ 31,799,461
86	0.21	€ 331,448	€ 662,895	€ 994,343	##########	-€ 8,014,304	-€ 16,028,609	-€ 24,042,913	-€ 32,057,217
87	0.14	€ 223,022	€ 446,043	€ 669,065	€ 892,087	-€ 5,392,598	-€ 10,785,197	-€ 16,177,795	-€ 21,570,393
88	0.25	€ 400,194	€ 800,388	##########	##########	-€ 9,676,566	-€ 19,353,132	-€ 29,029,698	-€ 38,706,265
89	0.19	€ 295,571	€ 591,142	€ 886,713	##########	-€ 7,146,815	-€ 14,293,630	-€ 21,440,445	-€ 28,587,260
90	0.15	€ 241,321	€ 482,642	€ 723,964	€ 965,285	-€ 5,835,075	-€ 11,670,149	-€ 17,505,224	-€ 23,340,299
91	0.23	€ 364,931	€ 729,863	##########	##########	-€ 8,823,932	-€ 17,647,865	-€ 26,471,797	-€ 35,295,729
92	0.18	€ 281,901	€ 563,802	€ 845,703	##########	-€ 6,816,280	-€ 13,632,561	-€ 20,448,841	-€ 27,265,122
93	0.14	€ 226,925	€ 453,850	€ 680,775	€ 907,701	-€ 5,486,982	-€ 10,973,964	-€ 16,460,947	-€ 21,947,929
94	0.24	€ 382,716	€ 765,432	##########	##########	-€ 9,253,956	-€ 18,507,913	-€ 27,761,869	-€ 37,015,825
95	0.16	€ 248,576	€ 497,152	€ 745,727	€ 994,303	-€ 6,010,488	-€ 12,020,976	-€ 18,031,464	-€ 24,041,952
96	0.21	€ 336,574	€ 673,149	##########	##########	-€ 8,138,269	-€ 16,276,538	-€ 24,414,806	-€ 32,553,075
97	0.20	€ 314,083	€ 628,165	€ 942,248	##########	-€ 7,594,422	-€ 15,188,845	-€ 22,783,267	-€ 30,377,690
98	0.13	€ 209,003	€ 418,007	€ 627,010	€ 836,013	-€ 5,053,639	-€ 10,107,279	-€ 15,160,918	-€ 20,214,557

min_prevalence	0.08
max_prevalence	0.34

25% actual costs	2.5 percentile	€ 188,574
	97.5 percentile	€ 426,072
50% actual costs	2.5 percentile	€ 377,148
	97.5 percentile	€ 852,145
75% actual costs	2.5 percentile	€ 565,722
	97.5 percentile	€ 1,278,217
100% actual costs	2.5 percentile	€ 754,296
	97.5 percentile	€ 1,704,290

25% reimbursed costs	2.5 percentile	-€ 10,302,306
	97.5 percentile	-€ 4,559,661
50% reimbursed costs	2.5 percentile	-€ 20,604,613
	97.5 percentile	-€ 9,119,322
75% reimbursed costs	2.5 percentile	-€ 30,906,919
	97.5 percentile	-€ 13,678,984
100% reimbursed costs	2.5 percentile	-€ 41,209,225
	97.5 percentile	-€ 18,238,645

99	0.22	€ 344,400	€ 688,800	##########	##########	-€ 8,327,493	-€ 16,654,987	-€ 24,982,480	-€ 33,309,973
100	0.18	€ 282,560	€ 565,119	€ 847,679	##########	-€ 6,832,207	-€ 13,664,414	-€ 20,496,622	-€ 27,328,829
101	0.17	€ 274,043	€ 548,086	€ 822,130	##########	-€ 6,626,283	-€ 13,252,566	-€ 19,878,849	-€ 26,505,132
102	0.16	€ 253,952	€ 507,904	€ 761,855	##########	-€ 6,140,478	-€ 12,280,956	-€ 18,421,434	-€ 24,561,912
103	0.21	€ 334,025	€ 668,049	##########	##########	-€ 8,076,614	-€ 16,153,228	-€ 24,229,843	-€ 32,306,457
104	0.23	€ 356,824	€ 713,647	##########	##########	-€ 8,627,891	-€ 17,255,782	-€ 25,883,673	-€ 34,511,564
105	0.17	€ 263,075	€ 526,149	€ 789,224	##########	-€ 6,361,065	-€ 12,722,130	-€ 19,083,195	-€ 25,444,260
106	0.20	€ 320,532	€ 641,064	€ 961,596	##########	-€ 7,750,369	-€ 15,500,739	-€ 23,251,108	-€ 31,001,478
107	0.17	€ 261,898	€ 523,796	€ 785,694	##########	-€ 6,332,617	-€ 12,665,233	-€ 18,997,850	-€ 25,330,466
108	0.24	€ 377,135	€ 754,271	##########	##########	-€ 9,119,020	-€ 18,238,039	-€ 27,357,059	-€ 36,476,079
109	0.18	€ 289,860	€ 579,720	€ 869,580	##########	-€ 7,008,731	-€ 14,017,461	-€ 21,026,192	-€ 28,034,923
110	0.21	€ 333,174	€ 666,347	€ 999,521	##########	-€ 8,056,038	-€ 16,112,076	-€ 24,168,114	-€ 32,224,151
111	0.21	€ 335,484	€ 670,967	##########	##########	-€ 8,111,897	-€ 16,223,794	-€ 24,335,691	-€ 32,447,588
112	0.28	€ 445,133	€ 890,266	##########	##########	-€ 10,763,179	-€ 21,526,358	-€ 32,289,536	-€ 43,052,715
113	0.22	€ 353,031	€ 706,061	##########	##########	-€ 8,536,174	-€ 17,072,348	-€ 25,608,522	-€ 34,144,697
114	0.16	€ 256,293	€ 512,586	€ 768,879	##########	-€ 6,197,089	-€ 12,394,177	-€ 18,591,266	-€ 24,788,354
115	0.14	€ 221,188	€ 442,375	€ 663,563	€ 884,751	-€ 5,348,252	-€ 10,696,504	-€ 16,044,757	-€ 21,393,009
116	0.17	€ 262,527	€ 525,054	€ 787,581	##########	-€ 6,347,826	-€ 12,695,652	-€ 19,043,477	-€ 25,391,303
117	0.16	€ 247,903	€ 495,807	€ 743,710	€ 991,614	-€ 5,994,231	-€ 11,988,463	-€ 17,982,694	-€ 23,976,925
118	0.19	€ 300,565	€ 601,130	€ 901,695	##########	-€ 7,267,576	-€ 14,535,151	-€ 21,802,727	-€ 29,070,302
119	0.18	€ 278,749	€ 557,499	€ 836,248	##########	-€ 6,740,076	-€ 13,480,152	-€ 20,220,229	-€ 26,960,305
120	0.16	€ 250,556	€ 501,113	€ 751,669	##########	-€ 6,058,377	-€ 12,116,754	-€ 18,175,132	-€ 24,233,509
121	0.16	€ 251,968	€ 503,935	€ 755,903	##########	-€ 6,092,503	-€ 12,185,006	-€ 18,277,509	-€ 24,370,012
122	0.18	€ 280,775	€ 561,550	€ 842,325	##########	-€ 6,789,053	-€ 13,578,106	-€ 20,367,158	-€ 27,156,211
123	0.20	€ 320,733	€ 641,466	€ 962,198	##########	-€ 7,755,225	-€ 15,510,449	-€ 23,265,674	-€ 31,020,899
124	0.23	€ 367,236	€ 734,472	##########	##########	-€ 8,879,656	-€ 17,759,312	-€ 26,638,967	-€ 35,518,623
125	0.22	€ 354,185	€ 708,369	##########	##########	-€ 8,564,079	-€ 17,128,157	-€ 25,692,236	-€ 34,256,314
126	0.26	€ 415,345	€ 830,690	##########	##########	-€ 10,042,921	-€ 20,085,843	-€ 30,128,764	-€ 40,171,686
127	0.26	€ 412,281	€ 824,561	##########	##########	-€ 9,968,820	-€ 19,937,641	-€ 29,906,461	-€ 39,875,281
128	0.13	€ 211,362	€ 422,725	€ 634,087	€ 845,449	-€ 5,110,678	-€ 10,221,357	-€ 15,332,035	-€ 20,442,714
129	0.18	€ 286,777	€ 573,554	€ 860,331	##########	-€ 6,934,180	-€ 13,868,360	-€ 20,802,540	-€ 27,736,720
130	0.16	€ 246,265	€ 492,530	€ 738,795	€ 985,060	-€ 5,954,615	-€ 11,909,230	-€ 17,863,844	-€ 23,818,459
131	0.14	€ 213,701	€ 427,401	€ 641,102	€ 854,802	-€ 5,167,216	-€ 10,334,432	-€ 15,501,649	-€ 20,668,865
132	0.19	€ 300,736	€ 601,473	€ 902,209	##########	-€ 7,271,714	-€ 14,543,428	-€ 21,815,141	-€ 29,086,855
133	0.13	€ 199,458	€ 398,916	€ 598,374	€ 797,832	-€ 4,822,837	-€ 9,645,674	-€ 14,468,511	-€ 19,291,348
134	0.28	€ 436,383	€ 872,766	##########	##########	-€ 10,551,612	-€ 21,103,223	-€ 31,654,835	-€ 42,206,446
135	0.23	€ 357,296	€ 714,592	##########	##########	-€ 8,639,317	-€ 17,278,634	-€ 25,917,951	-€ 34,557,267
136	0.20	€ 313,610	€ 627,220	€ 940,830	##########	-€ 7,582,994	-€ 15,165,988	-€ 22,748,982	-€ 30,331,976
137	0.22	€ 347,576	€ 695,153	##########	##########	-€ 8,404,294	-€ 16,808,588	-€ 25,212,883	-€ 33,617,177
138	0.20	€ 315,716	€ 631,432	€ 947,148	##########	-€ 7,633,918	-€ 15,267,835	-€ 22,901,753	-€ 30,535,671
139	0.18	€ 281,402	€ 562,804	€ 844,207	##########	-€ 6,804,222	-€ 13,608,444	-€ 20,412,667	-€ 27,216,889
140	0.18	€ 284,147	€ 568,294	€ 852,441	##########	-€ 6,870,588	-€ 13,741,176	-€ 20,611,765	-€ 27,482,353
141	0.13	€ 206,518	€ 413,037	€ 619,555	€ 826,074	-€ 4,993,553	-€ 9,987,106	-€ 14,980,660	-€ 19,974,213
142	0.19	€ 303,005	€ 606,010	€ 909,015	##########	-€ 7,326,572	-€ 14,653,144	-€ 21,979,716	-€ 29,306,288
143	0.13	€ 206,583	€ 413,165	€ 619,748	€ 826,330	-€ 4,995,106	-€ 9,990,212	-€ 14,985,318	-€ 19,980,424
144	0.20	€ 315,429	€ 630,857	€ 946,286	##########	-€ 7,626,969	-€ 15,253,938	-€ 22,880,907	-€ 30,507,876
145	0.14	€ 225,164	€ 450,328	€ 675,492	€ 900,656	-€ 5,444,401	-€ 10,888,802	-€ 16,333,203	-€ 21,777,604
146	0.34	€ 533,644	########	##########	##########	-€ 12,903,355	-€ 25,806,710	-€ 38,710,065	-€ 51,613,420
147	0.16	€ 249,842	€ 499,684	€ 749,526	€ 999,369	-€ 6,041,109	-€ 12,082,217	-€ 18,123,326	-€ 24,164,434
148	0.25	€ 388,493	€ 776,987	##########	##########	-€ 9,393,652	-€ 18,787,303	-€ 28,180,955	-€ 37,574,606
149	0.19	€ 296,621	€ 593,242	€ 889,862	##########	-€ 7,172,204	-€ 14,344,407	-€ 21,516,611	-€ 28,688,814
150	0.13	€ 201,556	€ 403,113	€ 604,669	€ 806,225	-€ 4,873,573	-€ 9,747,146	-€ 14,620,720	-€ 19,494,293
151	0.18	€ 285,111	€ 570,222	€ 855,333	##########	-€ 6,893,896	-€ 13,787,792	-€ 20,681,689	-€ 27,575,585
152	0.21	€ 327,797	€ 655,594	€ 983,391	##########	-€ 7,926,034	-€ 15,852,067	-€ 23,778,101	-€ 31,704,135
153	0.18	€ 278,902	€ 557,804	€ 836,705	##########	-€ 6,743,762	-€ 13,487,524	-€ 20,231,286	-€ 26,975,048
154	0.20	€ 317,809	€ 635,619	€ 953,428	##########	-€ 7,684,535	-€ 15,369,070	-€ 23,053,605	-€ 30,738,140
155	0.20	€ 312,004	€ 624,009	€ 936,013	##########	-€ 7,544,174	-€ 15,088,348	-€ 22,632,522	-€ 30,176,696
156	0.19	€ 296,919	€ 593,838	€ 890,757	##########	-€ 7,179,409	-€ 14,358,819	-€ 21,538,228	-€ 28,717,638
157	0.23	€ 357,102	€ 714,205	##########	##########	-€ 8,634,630	-€ 17,269,260	-€ 25,903,891	-€ 34,538,521
158	0.20	€ 319,894	€ 639,787	€ 959,681	##########	-€ 7,734,931	-€ 15,469,861	-€ 23,204,792	-€ 30,939,723
159	0.21	€ 324,641	€ 649,283	€ 973,924	##########	-€ 7,849,732	-€ 15,699,465	-€ 23,549,197	-€ 31,398,929
160	0.20	€ 307,311	€ 614,623	€ 921,934	##########	-€ 7,430,699	-€ 14,861,399	-€ 22,292,098	-€ 29,722,797
161	0.20	€ 321,353	€ 642,705	€ 964,058	##########	-€ 7,770,209	-€ 15,540,419	-€ 23,310,628	-€ 31,080,837
162	0.15	€ 241,292	€ 482,583	€ 723,875	€ 965,167	-€ 5,834,361	-€ 11,668,722	-€ 17,503,083	-€ 23,337,444
163	0.25	€ 397,934	€ 795,868	##########	##########	-€ 9,621,923	-€ 19,243,846	-€ 28,865,770	-€ 38,487,693
164	0.18	€ 287,213	€ 574,426	€ 861,638	##########	-€ 6,944,719	-€ 13,889,438	-€ 20,834,158	-€ 27,778,877
165	0.22	€ 345,788	€ 691,575	##########	##########	-€ 8,361,045	-€ 16,722,090	-€ 25,083,135	-€ 33,444,179
166	0.20	€ 310,117	€ 620,235	€ 930,352	##########	-€ 7,498,549	-€ 14,997,097	-€ 22,495,646	-€ 29,994,195
167	0.21	€ 324,262	€ 648,523	€ 972,785	##########	-€ 7,840,550	-€ 15,681,100	-€ 23,521,650	-€ 31,362,200
168	0.19	€ 306,355	€ 612,711	€ 919,066	##########	-€ 7,407,580	-€ 14,815,160	-€ 22,222,740	-€ 29,630,320
169	0.24	€ 375,949	€ 751,898	##########	##########	-€ 9,090,338	-€ 18,180,677	-€ 27,271,015	-€ 36,361,354
170	0.18	€ 285,682	€ 571,363	€ 857,045	##########	-€ 6,907,696	-€ 13,815,393	-€ 20,723,089	-€ 27,630,785
171	0.17	€ 271,781	€ 543,562	€ 815,343	##########	-€ 6,571,581	-€ 13,143,161	-€ 19,714,742	-€ 26,286,323
172	0.24	€ 376,188	€ 752,375	##########	##########	-€ 9,096,104	-€ 18,192,207	-€ 27,288,311	-€ 36,384,415
173	0.29	€ 459,278	€ 918,556	##########	##########	-€ 11,105,211	-€ 22,210,421	-€ 33,315,632	-€ 44,420,843
174	0.12	€ 195,323	€ 390,646	€ 585,969	€ 781,292	-€ 4,722,853	-€ 9,445,705	-€ 14,168,558	-€ 18,891,410
175	0.18	€ 288,208	€ 576,416	€ 864,624	##########	-€ 6,968,780	-€ 13,937,560	-€ 20,906,340	-€ 27,875,120
176	0.15	€ 241,471	€ 482,942	€ 724,413	€ 965,884	-€ 5,838,700	-€ 11,677,399	-€ 17,516,099	-€ 23,354,798
177	0.18	€ 278,434	€ 556,869	€ 835,303	##########	-€ 6,732,459	-€ 13,464,917	-€ 20,197,376	-€ 26,929,835
178	0.24	€ 374,995	€ 749,989	##########	##########	-€ 9,067,257	-€ 18,134,514	-€ 27,201,771	-€ 36,269,029
179	0.15	€ 236,975	€ 473,951	€ 710,926	€ 947,902	-€ 5,729,995	-€ 11,459,990	-€ 17,189,985	-€ 22,919,980
180	0.22	€ 351,215	€ 702,430	##########	##########	-€ 8,492,273	-€ 16,984,545	-€ 25,476,818	-€ 33,969,091
181	0.20	€ 318,674	€ 637,348	€ 956,023	##########	-€ 7,705,448	-€ 15,410,896	-€ 23,116,344	-€ 30,821,792
182	0.17	€ 264,209	€ 528,417	€ 792,626	##########	-€ 6,388,487	-€ 12,776,974	-€ 19,165,461	-€ 25,553,947
183	0.23	€ 364,342	€ 728,684	##########	##########	-€ 8,809,681	-€ 17,619,362	-€ 26,429,044	-€ 35,238,725
184	0.16	€ 248,822	€ 497,644	€ 746,466	€ 995,288	-€ 6,016,441	-€ 12,032,882	-€ 18,049,322	-€ 24,065,763
185	0.22	€ 344,644	€ 689,289	##########	##########	-€ 8,333,399	-€ 16,666,798	-€ 25,000,197	-€ 33,333,595
186	0.21	€ 326,519	€ 653,037	€ 979,556	##########	-€ 7,895,124	-€ 15,790,249	-€ 23,685,373	-€ 31,580,497
187	0.13	€ 204,365	€ 408,730	€ 613,095	€ 817,459	-€ 4,941,482	-€ 9,882,964	-€ 14,824,445	-€ 19,765,927
188	0.22	€ 341,982	€ 683,964	##########	##########	-€ 8,269,018	-€ 16,538,035	-€ 24,807,053	-€ 33,076,070
189	0.22	€ 352,984	€ 705,967	##########	##########	-€ 8,535,042	-€ 17,070,083	-€ 25,605,125	-€ 34,140,166
190	0.21	€ 337,147	€ 674,293	##########	##########	-€ 8,152,103	-€ 16,304,206	-€ 24,456,309	-€ 32,608,411
191	0.24	€ 371,864	€ 743,728	##########	##########	-€ 8,991,557	-€ 17,983,115	-€ 26,974,672	-€ 35,966,230
192	0.15	€ 243,392	€ 486,783	€ 730,175	€ 973,567	-€ 5,885,140	-€ 11,770,279	-€ 17,655,419	-€ 23,540,558
193	0.13	€ 202,977	€ 405,954	€ 608,932	€ 811,909	-€ 4,907,928	-€ 9,815,855	-€ 14,723,783	-€ 19,631,711
194	0.17	€ 272,687	€ 545,375	€ 818,062	##########	-€ 6,593,500	-€ 13,187,000	-€ 19,780,500	-€ 26,374,001
195	0.24	€ 378,940	€ 757,881	##########	##########	-€ 9,162,664	-€ 18,325,329	-€ 27,487,993	-€ 36,650,657
196	0.21	€ 326,814	€ 653,628	€ 980,443	##########	-€ 7,902,270	-€ 15,804,539	-€ 23,706,809	-€ 31,609,078
197	0.22	€ 345,294	€ 690,588	##########	##########	-€ 8,349,105	-€ 16,698,211	-€ 25,047,316	-€ 33,396,422
198	0.18	€ 276,201	€ 552,402	€ 828,603	##########	-€ 6,678,455	-€ 13,356,911	-€ 20,035,366	-€ 26,713,821
199	0.18	€ 276,786	€ 553,573	€ 830,359	##########	-€ 6,692,612	-€ 13,385,225	-€ 20,077,837	-€ 26,770,449
200	0.30	€ 468,247	€ 936,495	##########	##########	-€ 11,322,080	-€ 22,644,160	-€ 33,966,240	-€ 45,288,319

201	0.19	€ 299,090	€ 598,179	€ 897,269	##########	-€ 7,231,899	-€ 14,463,798	-€ 21,695,697	-€ 28,927,596
202	0.14	€ 220,696	€ 441,392	€ 662,088	€ 882,784	-€ 5,336,365	-€ 10,672,730	-€ 16,009,096	-€ 21,345,461
203	0.25	€ 400,341	€ 800,682	##########	##########	-€ 9,680,127	-€ 19,360,255	-€ 29,040,382	-€ 38,720,510
204	0.18	€ 291,264	€ 582,528	€ 873,793	##########	-€ 7,042,682	-€ 14,085,364	-€ 21,128,047	-€ 28,170,729
205	0.17	€ 260,317	€ 520,634	€ 780,950	##########	-€ 6,294,382	-€ 12,588,764	-€ 18,883,146	-€ 25,177,528
206	0.24	€ 370,152	€ 740,305	##########	##########	-€ 8,950,177	-€ 17,900,354	-€ 26,850,531	-€ 35,800,708
207	0.13	€ 205,567	€ 411,135	€ 616,702	€ 822,270	-€ 4,970,560	-€ 9,941,120	-€ 14,911,681	-€ 19,882,241
208	0.16	€ 259,265	€ 518,531	€ 777,796	##########	-€ 6,268,957	-€ 12,537,914	-€ 18,806,870	-€ 25,075,827
209	0.16	€ 257,739	€ 515,478	€ 773,216	##########	-€ 6,232,047	-€ 12,464,094	-€ 18,696,141	-€ 24,928,188
210	0.20	€ 319,606	€ 639,211	€ 958,817	##########	-€ 7,727,969	-€ 15,455,939	-€ 23,183,908	-€ 30,911,877
211	0.19	€ 302,274	€ 604,548	€ 906,822	##########	-€ 7,308,897	-€ 14,617,795	-€ 21,926,692	-€ 29,235,590
212	0.20	€ 322,188	€ 644,376	€ 966,564	##########	-€ 7,790,407	-€ 15,580,815	-€ 23,371,222	-€ 31,161,630
213	0.16	€ 255,868	€ 511,736	€ 767,604	##########	-€ 6,186,810	-€ 12,373,621	-€ 18,560,431	-€ 24,747,241
214	0.20	€ 308,196	€ 616,391	€ 924,587	##########	-€ 7,452,076	-€ 14,904,152	-€ 22,356,229	-€ 29,808,305
215	0.15	€ 237,372	€ 474,744	€ 712,116	€ 949,489	-€ 5,739,588	-€ 11,479,177	-€ 17,218,765	-€ 22,958,354
216	0.18	€ 275,633	€ 551,266	€ 826,900	##########	-€ 6,664,728	-€ 13,329,457	-€ 19,994,185	-€ 26,658,913
217	0.19	€ 293,231	€ 586,462	€ 879,694	##########	-€ 7,090,244	-€ 14,180,488	-€ 21,270,732	-€ 28,360,976
218	0.18	€ 276,294	€ 552,588	€ 828,881	##########	-€ 6,680,702	-€ 13,361,404	-€ 20,042,107	-€ 26,722,809
219	0.18	€ 282,430	€ 564,859	€ 847,289	##########	-€ 6,829,063	-€ 13,658,126	-€ 20,487,188	-€ 27,316,251
220	0.24	€ 371,359	€ 742,718	##########	##########	-€ 8,979,349	-€ 17,958,699	-€ 26,938,048	-€ 35,917,397
221	0.15	€ 236,744	€ 473,489	€ 710,233	€ 946,977	-€ 5,724,406	-€ 11,448,813	-€ 17,173,219	-€ 22,897,625
222	0.13	€ 211,463	€ 422,927	€ 634,390	€ 845,854	-€ 5,113,123	-€ 10,226,246	-€ 15,339,369	-€ 20,452,492
223	0.19	€ 292,527	€ 585,054	€ 877,581	##########	-€ 7,073,217	-€ 14,146,433	-€ 21,219,650	-€ 28,292,866
224	0.19	€ 294,676	€ 589,351	€ 884,027	##########	-€ 7,125,167	-€ 14,250,334	-€ 21,375,501	-€ 28,500,668
225	0.19	€ 292,849	€ 585,698	€ 878,546	##########	-€ 7,080,997	-€ 14,161,993	-€ 21,242,990	-€ 28,323,986
226	0.14	€ 226,578	€ 453,155	€ 679,733	€ 906,311	-€ 5,478,580	-€ 10,957,160	-€ 16,435,740	-€ 21,914,320
227	0.22	€ 344,804	€ 689,608	##########	##########	-€ 8,337,255	-€ 16,674,509	-€ 25,011,764	-€ 33,349,019
228	0.14	€ 215,403	€ 430,807	€ 646,210	€ 861,613	-€ 5,208,389	-€ 10,416,779	-€ 15,625,168	-€ 20,833,557
229	0.17	€ 262,575	€ 525,150	€ 787,726	##########	-€ 6,348,990	-€ 12,697,981	-€ 19,046,971	-€ 25,395,961
230	0.12	€ 187,980	€ 375,960	€ 563,939	€ 751,919	-€ 4,545,295	-€ 9,090,590	-€ 13,635,885	-€ 18,181,180
231	0.17	€ 268,467	€ 536,933	€ 805,400	##########	-€ 6,491,446	-€ 12,982,892	-€ 19,474,338	-€ 25,965,784
232	0.19	€ 299,521	€ 599,041	€ 898,562	##########	-€ 7,242,318	-€ 14,484,637	-€ 21,726,955	-€ 28,969,273
233	0.23	€ 365,050	€ 730,101	##########	##########	-€ 8,826,812	-€ 17,653,623	-€ 26,480,435	-€ 35,307,247
234	0.17	€ 273,431	€ 546,861	€ 820,292	##########	-€ 6,611,473	-€ 13,222,947	-€ 19,834,420	-€ 26,445,893
235	0.19	€ 297,312	€ 594,625	€ 891,937	##########	-€ 7,188,925	-€ 14,377,850	-€ 21,566,774	-€ 28,755,699
236	0.23	€ 356,124	€ 712,249	##########	##########	-€ 8,610,979	-€ 17,221,958	-€ 25,832,937	-€ 34,443,916
237	0.23	€ 354,989	€ 709,977	##########	##########	-€ 8,583,520	-€ 17,167,039	-€ 25,750,559	-€ 34,334,079
238	0.18	€ 283,083	€ 566,166	€ 849,250	##########	-€ 6,844,867	-€ 13,689,735	-€ 20,534,602	-€ 27,379,470
239	0.21	€ 325,273	€ 650,547	€ 975,820	##########	-€ 7,865,012	-€ 15,730,025	-€ 23,595,037	-€ 31,460,049
240	0.27	€ 428,662	€ 857,323	##########	##########	-€ 10,364,910	-€ 20,729,819	-€ 31,094,729	-€ 41,459,638
241	0.16	€ 245,503	€ 491,007	€ 736,510	€ 982,014	-€ 5,936,200	-€ 11,872,400	-€ 17,808,600	-€ 23,744,799
242	0.20	€ 322,248	€ 644,495	€ 966,743	##########	-€ 7,791,854	-€ 15,583,708	-€ 23,375,562	-€ 31,167,416
243	0.18	€ 288,547	€ 577,094	€ 865,640	##########	-€ 6,976,976	-€ 13,953,951	-€ 20,930,927	-€ 27,907,902
244	0.15	€ 237,205	€ 474,410	€ 711,615	€ 948,820	-€ 5,735,549	-€ 11,471,098	-€ 17,206,646	-€ 22,942,195
245	0.19	€ 296,793	€ 593,586	€ 890,379	##########	-€ 7,176,368	-€ 14,352,735	-€ 21,529,103	-€ 28,705,471
246	0.19	€ 295,309	€ 590,618	€ 885,926	##########	-€ 7,140,478	-€ 14,280,956	-€ 21,421,435	-€ 28,561,913
247	0.18	€ 286,712	€ 573,423	€ 860,135	##########	-€ 6,932,599	-€ 13,865,199	-€ 20,797,798	-€ 27,730,397
248	0.15	€ 230,823	€ 461,646	€ 692,470	€ 923,293	-€ 5,581,236	-€ 11,162,472	-€ 16,743,708	-€ 22,324,944
249	0.19	€ 296,519	€ 593,038	€ 889,558	##########	-€ 7,169,747	-€ 14,339,494	-€ 21,509,241	-€ 28,678,988
250	0.19	€ 297,283	€ 594,567	€ 891,850	##########	-€ 7,188,225	-€ 14,376,449	-€ 21,564,674	-€ 28,752,898
251	0.24	€ 383,079	€ 766,158	##########	##########	-€ 9,262,740	-€ 18,525,480	-€ 27,788,219	-€ 37,050,959
252	0.15	€ 241,629	€ 483,258	€ 724,888	€ 966,517	-€ 5,842,523	-€ 11,685,046	-€ 17,527,569	-€ 23,370,091
253	0.20	€ 308,461	€ 616,923	€ 925,384	##########	-€ 7,458,504	-€ 14,917,008	-€ 22,375,512	-€ 29,834,016
254	0.19	€ 295,825	€ 591,650	€ 887,475	##########	-€ 7,152,964	-€ 14,305,928	-€ 21,458,892	-€ 28,611,856
255	0.24	€ 376,814	€ 753,627	##########	##########	-€ 9,111,242	-€ 18,222,484	-€ 27,333,727	-€ 36,444,969
256	0.18	€ 286,106	€ 572,213	€ 858,319	##########	-€ 6,917,965	-€ 13,835,929	-€ 20,753,894	-€ 27,671,858
257	0.21	€ 336,669	€ 673,338	##########	##########	-€ 8,140,553	-€ 16,281,107	-€ 24,421,660	-€ 32,562,214
258	0.23	€ 363,799	€ 727,597	##########	##########	-€ 8,796,543	-€ 17,593,086	-€ 26,389,630	-€ 35,186,173
259	0.18	€ 284,226	€ 568,452	€ 852,678	##########	-€ 6,872,501	-€ 13,745,003	-€ 20,617,504	-€ 27,490,006
260	0.19	€ 297,275	€ 594,551	€ 891,826	##########	-€ 7,188,029	-€ 14,376,059	-€ 21,564,088	-€ 28,752,117
261	0.26	€ 403,061	€ 806,122	##########	##########	-€ 9,745,900	-€ 19,491,799	-€ 29,237,699	-€ 38,983,598
262	0.19	€ 303,810	€ 607,620	€ 911,430	##########	-€ 7,346,035	-€ 14,692,070	-€ 22,038,106	-€ 29,384,141
263	0.15	€ 231,452	€ 462,904	€ 694,356	€ 925,807	-€ 5,596,437	-€ 11,192,874	-€ 16,789,311	-€ 22,385,748
264	0.24	€ 376,143	€ 752,285	##########	##########	-€ 9,095,019	-€ 18,190,038	-€ 27,285,057	-€ 36,380,076
265	0.25	€ 401,491	€ 802,982	##########	##########	-€ 9,707,934	-€ 19,415,868	-€ 29,123,802	-€ 38,831,736
266	0.14	€ 217,706	€ 435,411	€ 653,117	€ 870,822	-€ 5,264,055	-€ 10,528,111	-€ 15,792,166	-€ 21,056,222
267	0.21	€ 328,648	€ 657,296	€ 985,943	##########	-€ 7,946,606	-€ 15,893,211	-€ 23,839,817	-€ 31,786,422
268	0.18	€ 287,685	€ 575,371	€ 863,056	##########	-€ 6,956,145	-€ 13,912,290	-€ 20,868,435	-€ 27,824,580
269	0.16	€ 248,830	€ 497,660	€ 746,490	€ 995,320	-€ 6,016,633	-€ 12,033,267	-€ 18,049,900	-€ 24,066,534
270	0.24	€ 383,220	€ 766,440	##########	##########	-€ 9,266,143	-€ 18,532,286	-€ 27,798,429	-€ 37,064,571
271	0.14	€ 218,178	€ 436,356	€ 654,535	€ 872,713	-€ 5,275,484	-€ 10,550,968	-€ 15,826,452	-€ 21,101,936
272	0.18	€ 282,425	€ 564,851	€ 847,276	##########	-€ 6,828,962	-€ 13,657,925	-€ 20,486,887	-€ 27,315,849
273	0.23	€ 361,827	€ 723,654	##########	##########	-€ 8,748,868	-€ 17,497,736	-€ 26,246,604	-€ 34,995,472
274	0.16	€ 250,082	€ 500,163	€ 750,245	##########	-€ 6,046,901	-€ 12,093,802	-€ 18,140,703	-€ 24,187,603
275	0.21	€ 333,975	€ 667,950	##########	##########	-€ 8,075,420	-€ 16,150,841	-€ 24,226,261	-€ 32,301,681
276	0.19	€ 297,915	€ 595,829	€ 893,744	##########	-€ 7,203,489	-€ 14,406,978	-€ 21,610,467	-€ 28,813,955
277	0.19	€ 292,962	€ 585,925	€ 878,887	##########	-€ 7,083,745	-€ 14,167,490	-€ 21,251,234	-€ 28,334,979
278	0.22	€ 352,684	€ 705,367	##########	##########	-€ 8,527,784	-€ 17,055,568	-€ 25,583,352	-€ 34,111,136
279	0.15	€ 239,350	€ 478,700	€ 718,049	€ 957,399	-€ 5,787,407	-€ 11,574,814	-€ 17,362,220	-€ 23,149,627
280	0.32	€ 502,315	########	##########	##########	-€ 12,145,835	-€ 24,291,670	-€ 36,437,505	-€ 48,583,340
281	0.19	€ 304,969	€ 609,937	€ 914,906	##########	-€ 7,374,053	-€ 14,748,106	-€ 22,122,158	-€ 29,496,211
282	0.14	€ 225,510	€ 451,019	€ 676,529	€ 902,039	-€ 5,452,757	-€ 10,905,514	-€ 16,358,271	-€ 21,811,028
283	0.17	€ 267,933	€ 535,866	€ 803,798	##########	-€ 6,478,536	-€ 12,957,073	-€ 19,435,609	-€ 25,914,145
284	0.13	€ 201,743	€ 403,487	€ 605,230	€ 806,974	-€ 4,878,097	-€ 9,756,195	-€ 14,634,292	-€ 19,512,389
285	0.17	€ 271,025	€ 542,049	€ 813,074	##########	-€ 6,553,296	-€ 13,106,593	-€ 19,659,889	-€ 26,213,185
286	0.25	€ 387,013	€ 774,026	##########	##########	-€ 9,357,862	-€ 18,715,725	-€ 28,073,587	-€ 37,431,450
287	0.18	€ 290,200	€ 580,400	€ 870,600	##########	-€ 7,016,948	-€ 14,033,897	-€ 21,050,845	-€ 28,067,793
288	0.31	€ 495,508	€ 991,016	##########	##########	-€ 11,981,235	-€ 23,962,469	-€ 35,943,704	-€ 47,924,939
289	0.27	€ 429,362	€ 858,724	##########	##########	-€ 10,381,851	-€ 20,763,701	-€ 31,145,552	-€ 41,527,403
290	0.18	€ 291,165	€ 582,331	€ 873,496	##########	-€ 7,040,293	-€ 14,080,585	-€ 21,120,878	-€ 28,161,171
291	0.23	€ 361,880	€ 723,760	##########	##########	-€ 8,750,148	-€ 17,500,297	-€ 26,250,445	-€ 35,000,594
292	0.18	€ 280,204	€ 560,408	€ 840,612	##########	-€ 6,775,252	-€ 13,550,504	-€ 20,325,755	-€ 27,101,007
293	0.22	€ 344,762	€ 689,523	##########	##########	-€ 8,336,234	-€ 16,672,468	-€ 25,008,702	-€ 33,344,936
294	0.18	€ 289,240	€ 578,479	€ 867,719	##########	-€ 6,993,725	-€ 13,987,451	-€ 20,981,176	-€ 27,974,902
295	0.13	€ 202,350	€ 404,700	€ 607,051	€ 809,401	-€ 4,892,769	-€ 9,785,538	-€ 14,678,306	-€ 19,571,075
296	0.22	€ 349,167	€ 698,334	##########	##########	-€ 8,442,760	-€ 16,885,521	-€ 25,328,281	-€ 33,771,042
297	0.21	€ 338,307	€ 676,614	##########	##########	-€ 8,180,168	-€ 16,360,336	-€ 24,540,505	-€ 32,720,673
298	0.24	€ 376,283	€ 752,566	##########	##########	-€ 9,098,406	-€ 18,196,813	-€ 27,295,219	-€ 36,393,626
299	0.17	€ 275,475	€ 550,949	€ 826,424	##########	-€ 6,660,894	-€ 13,321,789	-€ 19,982,683	-€ 26,643,577
300	0.18	€ 284,940	€ 569,880	€ 854,820	##########	-€ 6,889,766	-€ 13,779,533	-€ 20,669,299	-€ 27,559,065
301	0.14	€ 226,085	€ 452,170	€ 678,255	€ 904,340	-€ 5,466,668	-€ 10,933,336	-€ 16,400,003	-€ 21,866,671
302	0.16	€ 252,315	€ 504,629	€ 756,944	##########	-€ 6,100,895	-€ 12,201,790	-€ 18,302,685	-€ 24,403,580

303	0.15	€ 242,292	€ 484,583	€ 726,875	€ 969,166	-€ 5,858,538	-€ 11,717,077	-€ 17,575,615	-€ 23,434,154
304	0.17	€ 267,625	€ 535,249	€ 802,874	##########	-€ 6,471,087	-€ 12,942,174	-€ 19,413,261	-€ 25,884,348
305	0.19	€ 301,087	€ 602,174	€ 903,261	##########	-€ 7,280,194	-€ 14,560,389	-€ 21,840,583	-€ 29,120,777
306	0.21	€ 322,988	€ 645,977	€ 968,965	##########	-€ 7,809,763	-€ 15,619,526	-€ 23,429,289	-€ 31,239,052
307	0.25	€ 386,915	€ 773,830	##########	##########	-€ 9,355,490	-€ 18,710,979	-€ 28,066,469	-€ 37,421,959
308	0.20	€ 319,591	€ 639,182	€ 958,772	##########	-€ 7,727,611	-€ 15,455,223	-€ 23,182,834	-€ 30,910,445
309	0.21	€ 331,486	€ 662,972	€ 994,458	##########	-€ 8,015,237	-€ 16,030,473	-€ 24,045,710	-€ 32,060,947
310	0.19	€ 302,300	€ 604,601	€ 906,901	##########	-€ 7,309,530	-€ 14,619,061	-€ 21,928,591	-€ 29,238,121
311	0.15	€ 234,670	€ 469,340	€ 704,010	€ 938,680	-€ 5,674,249	-€ 11,348,499	-€ 17,022,748	-€ 22,696,998
312	0.16	€ 258,598	€ 517,197	€ 775,795	##########	-€ 6,252,829	-€ 12,505,659	-€ 18,758,488	-€ 25,011,318
313	0.20	€ 314,450	€ 628,900	€ 943,350	##########	-€ 7,603,305	-€ 15,206,609	-€ 22,809,914	-€ 30,413,218
314	0.15	€ 241,416	€ 482,832	€ 724,248	€ 965,664	-€ 5,837,368	-€ 11,674,735	-€ 17,512,103	-€ 23,349,471
315	0.20	€ 309,060	€ 618,121	€ 927,181	##########	-€ 7,472,988	-€ 14,945,975	-€ 22,418,963	-€ 29,891,951
316	0.26	€ 414,519	€ 829,039	##########	##########	-€ 10,022,954	-€ 20,045,908	-€ 30,068,862	-€ 40,091,816
317	0.22	€ 343,535	€ 687,071	##########	##########	-€ 8,306,581	-€ 16,613,163	-€ 24,919,744	-€ 33,226,326
318	0.18	€ 288,977	€ 577,954	€ 866,931	##########	-€ 6,987,378	-€ 13,974,756	-€ 20,962,134	-€ 27,949,512
319	0.24	€ 374,295	€ 748,590	##########	##########	-€ 9,050,342	-€ 18,100,683	-€ 27,151,025	-€ 36,201,367
320	0.17	€ 270,917	€ 541,833	€ 812,750	##########	-€ 6,550,682	-€ 13,101,364	-€ 19,652,046	-€ 26,202,727
321	0.20	€ 320,046	€ 640,092	€ 960,137	##########	-€ 7,738,612	-€ 15,477,224	-€ 23,215,836	-€ 30,954,448
322	0.21	€ 337,084	€ 674,168	##########	##########	-€ 8,150,587	-€ 16,301,174	-€ 24,451,761	-€ 32,602,348
323	0.17	€ 272,292	€ 544,585	€ 816,877	##########	-€ 6,583,950	-€ 13,167,901	-€ 19,751,851	-€ 26,335,801
324	0.25	€ 388,791	€ 777,582	##########	##########	-€ 9,400,850	-€ 18,801,699	-€ 28,202,549	-€ 37,603,399
325	0.17	€ 262,897	€ 525,793	€ 788,690	##########	-€ 6,356,765	-€ 12,713,530	-€ 19,070,295	-€ 25,427,060
326	0.19	€ 298,695	€ 597,389	€ 896,084	##########	-€ 7,222,345	-€ 14,444,690	-€ 21,667,036	-€ 28,889,381
327	0.19	€ 300,338	€ 600,676	€ 901,014	##########	-€ 7,262,086	-€ 14,524,172	-€ 21,786,258	-€ 29,048,344
328	0.19	€ 295,085	€ 590,170	€ 885,255	##########	-€ 7,135,070	-€ 14,270,140	-€ 21,405,209	-€ 28,540,279
329	0.22	€ 346,818	€ 693,637	##########	##########	-€ 8,385,966	-€ 16,771,932	-€ 25,157,899	-€ 33,543,865
330	0.23	€ 368,079	€ 736,158	##########	##########	-€ 8,900,046	-€ 17,800,092	-€ 26,700,138	-€ 35,600,184
331	0.16	€ 249,382	€ 498,764	€ 748,146	€ 997,528	-€ 6,029,985	-€ 12,059,971	-€ 18,089,956	-€ 24,119,941
332	0.22	€ 353,460	€ 706,921	##########	##########	-€ 8,546,566	-€ 17,093,132	-€ 25,639,697	-€ 34,186,263
333	0.15	€ 229,928	€ 459,855	€ 689,783	€ 919,710	-€ 5,559,581	-€ 11,119,161	-€ 16,678,742	-€ 22,238,323
334	0.16	€ 255,885	€ 511,770	€ 767,655	##########	-€ 6,187,227	-€ 12,374,454	-€ 18,561,681	-€ 24,748,908
335	0.30	€ 476,930	€ 953,859	##########	##########	-€ 11,532,018	-€ 23,064,035	-€ 34,596,053	-€ 46,128,071
336	0.23	€ 358,984	€ 717,967	##########	##########	-€ 8,680,116	-€ 17,360,232	-€ 26,040,349	-€ 34,720,465
337	0.18	€ 283,818	€ 567,636	€ 851,454	##########	-€ 6,862,634	-€ 13,725,268	-€ 20,587,902	-€ 27,450,537
338	0.21	€ 337,698	€ 675,396	##########	##########	-€ 8,165,441	-€ 16,330,882	-€ 24,496,322	-€ 32,661,763
339	0.22	€ 347,180	€ 694,361	##########	##########	-€ 8,394,719	-€ 16,789,437	-€ 25,184,156	-€ 33,578,874
340	0.11	€ 171,735	€ 343,470	€ 515,204	€ 686,939	-€ 4,152,496	-€ 8,304,992	-€ 12,457,488	-€ 16,609,984
341	0.17	€ 274,651	€ 549,302	€ 823,953	##########	-€ 6,640,983	-€ 13,281,965	-€ 19,922,948	-€ 26,563,931
342	0.20	€ 316,336	€ 632,673	€ 949,009	##########	-€ 7,648,917	-€ 15,297,833	-€ 22,946,750	-€ 30,595,667
343	0.15	€ 231,220	€ 462,441	€ 693,661	€ 924,882	-€ 5,590,840	-€ 11,181,680	-€ 16,772,520	-€ 22,363,360
344	0.27	€ 418,378	€ 836,756	##########	##########	-€ 10,116,254	-€ 20,232,509	-€ 30,348,763	-€ 40,465,018
345	0.16	€ 249,226	€ 498,453	€ 747,679	€ 996,905	-€ 6,026,218	-€ 12,052,436	-€ 18,078,655	-€ 24,104,873
346	0.17	€ 266,706	€ 533,412	€ 800,119	##########	-€ 6,448,877	-€ 12,897,754	-€ 19,346,631	-€ 25,795,508
347	0.18	€ 290,859	€ 581,718	€ 872,578	##########	-€ 7,032,888	-€ 14,065,777	-€ 21,098,665	-€ 28,131,554
348	0.16	€ 252,586	€ 505,171	€ 757,757	##########	-€ 6,107,447	-€ 12,214,894	-€ 18,322,340	-€ 24,429,787
349	0.19	€ 297,304	€ 594,608	€ 891,913	##########	-€ 7,188,728	-€ 14,377,455	-€ 21,566,183	-€ 28,754,910
350	0.17	€ 267,308	€ 534,616	€ 801,923	##########	-€ 6,463,423	-€ 12,926,845	-€ 19,390,268	-€ 25,853,690
351	0.23	€ 361,082	€ 722,165	##########	##########	-€ 8,730,865	-€ 17,461,729	-€ 26,192,594	-€ 34,923,458
352	0.13	€ 204,786	€ 409,572	€ 614,358	€ 819,144	-€ 4,951,662	-€ 9,903,325	-€ 14,854,987	-€ 19,806,649
353	0.24	€ 374,739	€ 749,478	##########	##########	-€ 9,061,076	-€ 18,122,152	-€ 27,183,229	-€ 36,244,305
354	0.13	€ 211,175	€ 422,349	€ 633,524	€ 844,699	-€ 5,106,142	-€ 10,212,284	-€ 15,318,426	-€ 20,424,568
355	0.21	€ 334,561	€ 669,123	##########	##########	-€ 8,089,593	-€ 16,179,185	-€ 24,268,778	-€ 32,358,370
356	0.23	€ 358,453	€ 716,907	##########	##########	-€ 8,667,298	-€ 17,334,596	-€ 26,001,894	-€ 34,669,192
357	0.23	€ 363,497	€ 726,994	##########	##########	-€ 8,789,255	-€ 17,578,511	-€ 26,367,766	-€ 35,157,022
358	0.15	€ 242,240	€ 484,479	€ 726,719	€ 968,959	-€ 5,857,283	-€ 11,714,565	-€ 17,571,848	-€ 23,429,130
359	0.13	€ 208,665	€ 417,330	€ 625,995	€ 834,660	-€ 5,045,458	-€ 10,090,916	-€ 15,136,375	-€ 20,181,833
360	0.21	€ 335,478	€ 670,955	##########	##########	-€ 8,111,747	-€ 16,223,493	-€ 24,335,240	-€ 32,446,987
361	0.23	€ 367,981	€ 735,963	##########	##########	-€ 8,897,679	-€ 17,795,358	-€ 26,693,038	-€ 35,590,717
362	0.21	€ 337,094	€ 674,187	##########	##########	-€ 8,150,822	-€ 16,301,644	-€ 24,452,467	-€ 32,603,289
363	0.21	€ 336,350	€ 672,700	##########	##########	-€ 8,132,845	-€ 16,265,689	-€ 24,398,534	-€ 32,531,378
364	0.19	€ 303,483	€ 606,966	€ 910,448	##########	-€ 7,338,124	-€ 14,676,248	-€ 22,014,372	-€ 29,352,495
365	0.19	€ 299,683	€ 599,365	€ 899,048	##########	-€ 7,246,240	-€ 14,492,479	-€ 21,738,719	-€ 28,984,959
366	0.19	€ 304,127	€ 608,254	€ 912,381	##########	-€ 7,353,697	-€ 14,707,394	-€ 22,061,091	-€ 29,414,788
367	0.19	€ 296,584	€ 593,167	€ 889,751	##########	-€ 7,171,302	-€ 14,342,604	-€ 21,513,906	-€ 28,685,207
368	0.21	€ 330,794	€ 661,588	€ 992,382	##########	-€ 7,998,500	-€ 15,996,999	-€ 23,995,499	-€ 31,993,998
369	0.12	€ 188,569	€ 377,139	€ 565,708	€ 754,278	-€ 4,559,552	-€ 9,119,105	-€ 13,678,657	-€ 18,238,210
370	0.16	€ 256,444	€ 512,887	€ 769,331	##########	-€ 6,200,731	-€ 12,401,462	-€ 18,602,193	-€ 24,802,924
371	0.12	€ 196,742	€ 393,483	€ 590,225	€ 786,966	-€ 4,757,154	-€ 9,514,308	-€ 14,271,462	-€ 19,028,615
372	0.17	€ 263,079	€ 526,158	€ 789,238	##########	-€ 6,361,178	-€ 12,722,355	-€ 19,083,533	-€ 25,444,710
373	0.13	€ 201,133	€ 402,265	€ 603,398	€ 804,530	-€ 4,863,327	-€ 9,726,654	-€ 14,589,981	-€ 19,453,308
374	0.14	€ 224,216	€ 448,433	€ 672,649	€ 896,866	-€ 5,421,487	-€ 10,842,975	-€ 16,264,462	-€ 21,685,950
375	0.28	€ 447,918	€ 895,835	##########	##########	-€ 10,830,513	-€ 21,661,025	-€ 32,491,538	-€ 43,322,051
376	0.10	€ 162,318	€ 324,636	€ 486,954	€ 649,272	-€ 3,924,799	-€ 7,849,599	-€ 11,774,398	-€ 15,699,198
377	0.20	€ 309,652	€ 619,303	€ 928,955	##########	-€ 7,487,285	-€ 14,974,571	-€ 22,461,856	-€ 29,949,142
378	0.20	€ 314,406	€ 628,812	€ 943,218	##########	-€ 7,602,245	-€ 15,204,490	-€ 22,806,735	-€ 30,408,980
379	0.15	€ 230,882	€ 461,763	€ 692,645	€ 923,526	-€ 5,582,647	-€ 11,165,293	-€ 16,747,940	-€ 22,330,587
380	0.20	€ 314,718	€ 629,436	€ 944,154	##########	-€ 7,609,790	-€ 15,219,580	-€ 22,829,370	-€ 30,439,160
381	0.13	€ 206,149	€ 412,299	€ 618,448	€ 824,598	-€ 4,984,633	-€ 9,969,267	-€ 14,953,900	-€ 19,938,533
382	0.22	€ 341,937	€ 683,873	##########	##########	-€ 8,267,928	-€ 16,535,855	-€ 24,803,783	-€ 33,071,711
383	0.18	€ 291,022	€ 582,044	€ 873,066	##########	-€ 7,036,829	-€ 14,073,658	-€ 21,110,487	-€ 28,147,317
384	0.14	€ 214,404	€ 428,807	€ 643,211	€ 857,614	-€ 5,184,214	-€ 10,368,428	-€ 15,552,641	-€ 20,736,855
385	0.18	€ 277,225	€ 554,451	€ 831,676	##########	-€ 6,703,228	-€ 13,406,456	-€ 20,109,684	-€ 26,812,912
386	0.16	€ 246,791	€ 493,582	€ 740,372	€ 987,163	-€ 5,967,329	-€ 11,934,658	-€ 17,901,987	-€ 23,869,316
387	0.17	€ 274,267	€ 548,534	€ 822,801	##########	-€ 6,631,697	-€ 13,263,394	-€ 19,895,091	-€ 26,526,788
388	0.27	€ 419,860	€ 839,720	##########	##########	-€ 10,152,088	-€ 20,304,176	-€ 30,456,264	-€ 40,608,352
389	0.19	€ 297,134	€ 594,268	€ 891,402	##########	-€ 7,184,611	-€ 14,369,223	-€ 21,553,834	-€ 28,738,446
390	0.14	€ 227,266	€ 454,531	€ 681,797	€ 909,063	-€ 5,495,217	-€ 10,990,435	-€ 16,485,652	-€ 21,980,869
391	0.21	€ 333,740	€ 667,479	##########	##########	-€ 8,069,728	-€ 16,139,455	-€ 24,209,183	-€ 32,278,910
392	0.19	€ 292,046	€ 584,091	€ 876,137	##########	-€ 7,061,577	-€ 14,123,153	-€ 21,184,730	-€ 28,246,307
393	0.17	€ 263,983	€ 527,965	€ 791,948	##########	-€ 6,383,023	-€ 12,766,046	-€ 19,149,070	-€ 25,532,093
394	0.22	€ 346,693	€ 693,387	##########	##########	-€ 8,382,944	-€ 16,765,888	-€ 25,148,831	-€ 33,531,775
395	0.22	€ 343,176	€ 686,353	##########	##########	-€ 8,297,903	-€ 16,595,805	-€ 24,893,708	-€ 33,191,610
396	0.14	€ 217,413	€ 434,826	€ 652,239	€ 869,652	-€ 5,256,979	-€ 10,513,958	-€ 15,770,937	-€ 21,027,916
397	0.24	€ 384,295	€ 768,590	##########	##########	-€ 9,292,135	-€ 18,584,271	-€ 27,876,406	-€ 37,168,541
398	0.19	€ 304,726	€ 609,452	€ 914,177	##########	-€ 7,368,179	-€ 14,736,358	-€ 22,104,538	-€ 29,472,717
399	0.18	€ 286,488	€ 572,975	€ 859,463	##########	-€ 6,927,184	-€ 13,854,369	-€ 20,781,553	-€ 27,708,737
400	0.15	€ 243,147	€ 486,295	€ 729,442	€ 972,589	-€ 5,879,230	-€ 11,758,460	-€ 17,637,690	-€ 23,516,920
401	0.14	€ 226,157	€ 452,315	€ 678,472	€ 904,629	-€ 5,468,416	-€ 10,936,832	-€ 16,405,247	-€ 21,873,663
402	0.22	€ 341,635	€ 683,270	##########	##########	-€ 8,260,638	-€ 16,521,276	-€ 24,781,914	-€ 33,042,552
403	0.26	€ 410,408	€ 820,816	##########	##########	-€ 9,923,541	-€ 19,847,082	-€ 29,770,623	-€ 39,694,164
404	0.14	€ 212,995	€ 425,991	€ 638,986	€ 851,981	-€ 5,150,164	-€ 10,300,327	-€ 15,450,491	-€ 20,600,654

405	0.19	€ 305,737	€ 611,473	€ 917,210	##########	-€ 7,392,618	-€ 14,785,236	-€ 22,177,854	-€ 29,570,472
406	0.19	€ 294,066	€ 588,132	€ 882,198	##########	-€ 7,110,430	-€ 14,220,861	-€ 21,331,291	-€ 28,441,721
407	0.19	€ 301,095	€ 602,190	€ 903,285	##########	-€ 7,280,385	-€ 14,560,770	-€ 21,841,155	-€ 29,121,540
408	0.18	€ 275,832	€ 551,663	€ 827,495	##########	-€ 6,669,529	-€ 13,339,059	-€ 20,008,588	-€ 26,678,118
409	0.15	€ 237,626	€ 475,252	€ 712,878	€ 950,504	-€ 5,745,728	-€ 11,491,456	-€ 17,237,184	-€ 22,982,911
410	0.23	€ 367,646	€ 735,292	##########	##########	-€ 8,889,577	-€ 17,779,154	-€ 26,668,730	-€ 35,558,307
411	0.23	€ 360,565	€ 721,131	##########	##########	-€ 8,718,361	-€ 17,436,722	-€ 26,155,083	-€ 34,873,444
412	0.18	€ 277,443	€ 554,887	€ 832,330	##########	-€ 6,708,501	-€ 13,417,003	-€ 20,125,504	-€ 26,834,005
413	0.16	€ 248,213	€ 496,425	€ 744,638	€ 992,850	-€ 6,001,707	-€ 12,003,414	-€ 18,005,121	-€ 24,006,829
414	0.17	€ 268,386	€ 536,772	€ 805,158	##########	-€ 6,489,495	-€ 12,978,990	-€ 19,468,485	-€ 25,957,980
415	0.23	€ 355,393	€ 710,787	##########	##########	-€ 8,593,306	-€ 17,186,612	-€ 25,779,918	-€ 34,373,224
416	0.22	€ 352,372	€ 704,744	##########	##########	-€ 8,520,250	-€ 17,040,499	-€ 25,560,749	-€ 34,080,999
417	0.22	€ 353,001	€ 706,002	##########	##########	-€ 8,535,465	-€ 17,070,930	-€ 25,606,395	-€ 34,141,860
418	0.17	€ 267,321	€ 534,642	€ 801,963	##########	-€ 6,463,740	-€ 12,927,481	-€ 19,391,221	-€ 25,854,962
419	0.21	€ 329,531	€ 659,062	€ 988,593	##########	-€ 7,967,964	-€ 15,935,928	-€ 23,903,892	-€ 31,871,856
420	0.22	€ 341,939	€ 683,878	##########	##########	-€ 8,267,981	-€ 16,535,962	-€ 24,803,943	-€ 33,071,924
421	0.15	€ 238,211	€ 476,421	€ 714,632	€ 952,843	-€ 5,759,865	-€ 11,519,730	-€ 17,279,595	-€ 23,039,460
422	0.16	€ 258,308	€ 516,617	€ 774,925	##########	-€ 6,245,822	-€ 12,491,645	-€ 18,737,467	-€ 24,983,289
423	0.17	€ 270,079	€ 540,158	€ 810,238	##########	-€ 6,530,435	-€ 13,060,869	-€ 19,591,304	-€ 26,121,738
424	0.22	€ 343,023	€ 686,047	##########	##########	-€ 8,294,206	-€ 16,588,412	-€ 24,882,618	-€ 33,176,824
425	0.19	€ 297,590	€ 595,180	€ 892,770	##########	-€ 7,195,635	-€ 14,391,270	-€ 21,586,905	-€ 28,782,541
426	0.18	€ 279,206	€ 558,413	€ 837,619	##########	-€ 6,751,127	-€ 13,502,254	-€ 20,253,382	-€ 27,004,509
427	0.15	€ 231,520	€ 463,041	€ 694,561	€ 926,081	-€ 5,598,093	-€ 11,196,185	-€ 16,794,278	-€ 22,392,371
428	0.23	€ 364,619	€ 729,237	##########	##########	-€ 8,816,371	-€ 17,632,742	-€ 26,449,112	-€ 35,265,483
429	0.16	€ 259,412	€ 518,823	€ 778,235	##########	-€ 6,272,498	-€ 12,544,997	-€ 18,817,495	-€ 25,089,993
430	0.19	€ 296,056	€ 592,112	€ 888,168	##########	-€ 7,158,545	-€ 14,317,090	-€ 21,475,635	-€ 28,634,179
431	0.18	€ 285,833	€ 571,666	€ 857,500	##########	-€ 6,911,363	-€ 13,822,726	-€ 20,734,088	-€ 27,645,451
432	0.15	€ 230,857	€ 461,715	€ 692,572	€ 923,429	-€ 5,582,061	-€ 11,164,121	-€ 16,746,182	-€ 22,328,243
433	0.18	€ 280,440	€ 560,881	€ 841,321	##########	-€ 6,780,967	-€ 13,561,935	-€ 20,342,902	-€ 27,123,870
434	0.13	€ 211,216	€ 422,431	€ 633,647	€ 844,863	-€ 5,107,134	-€ 10,214,267	-€ 15,321,401	-€ 20,428,534
435	0.17	€ 260,676	€ 521,351	€ 782,027	##########	-€ 6,303,061	-€ 12,606,121	-€ 18,909,182	-€ 25,212,243
436	0.15	€ 228,752	€ 457,504	€ 686,256	€ 915,008	-€ 5,531,156	-€ 11,062,312	-€ 16,593,468	-€ 22,124,625
437	0.20	€ 309,891	€ 619,782	€ 929,673	##########	-€ 7,493,075	-€ 14,986,151	-€ 22,479,226	-€ 29,972,301
438	0.19	€ 294,759	€ 589,518	€ 884,277	##########	-€ 7,127,184	-€ 14,254,368	-€ 21,381,552	-€ 28,508,736
439	0.16	€ 256,031	€ 512,062	€ 768,092	##########	-€ 6,190,748	-€ 12,381,496	-€ 18,572,244	-€ 24,762,993
440	0.14	€ 216,436	€ 432,872	€ 649,307	€ 865,743	-€ 5,233,353	-€ 10,466,707	-€ 15,700,060	-€ 20,933,413
441	0.16	€ 245,625	€ 491,251	€ 736,876	€ 982,501	-€ 5,939,147	-€ 11,878,294	-€ 17,817,440	-€ 23,756,587
442	0.22	€ 347,337	€ 694,674	##########	##########	-€ 8,398,500	-€ 16,797,001	-€ 25,195,501	-€ 33,594,002
443	0.17	€ 268,208	€ 536,415	€ 804,623	##########	-€ 6,485,182	-€ 12,970,365	-€ 19,455,547	-€ 25,940,729
444	0.17	€ 264,233	€ 528,466	€ 792,700	##########	-€ 6,389,081	-€ 12,778,162	-€ 19,167,243	-€ 25,556,324
445	0.20	€ 314,505	€ 629,011	€ 943,516	##########	-€ 7,604,649	-€ 15,209,297	-€ 22,813,946	-€ 30,418,594
446	0.19	€ 294,111	€ 588,222	€ 882,334	##########	-€ 7,111,521	-€ 14,223,042	-€ 21,334,563	-€ 28,446,084
447	0.21	€ 330,107	€ 660,213	€ 990,320	##########	-€ 7,981,878	-€ 15,963,757	-€ 23,945,635	-€ 31,927,514
448	0.18	€ 279,756	€ 559,511	€ 839,267	##########	-€ 6,764,405	-€ 13,528,810	-€ 20,293,215	-€ 27,057,620
449	0.20	€ 310,593	€ 621,185	€ 931,778	##########	-€ 7,510,038	-€ 15,020,076	-€ 22,530,114	-€ 30,040,152
450	0.23	€ 366,292	€ 732,583	##########	##########	-€ 8,856,824	-€ 17,713,649	-€ 26,570,473	-€ 35,427,298
451	0.21	€ 338,043	€ 676,086	##########	##########	-€ 8,173,784	-€ 16,347,569	-€ 24,521,353	-€ 32,695,137
452	0.20	€ 307,200	€ 614,400	€ 921,600	##########	-€ 7,428,001	-€ 14,856,003	-€ 22,284,004	-€ 29,712,006
453	0.14	€ 222,708	€ 445,415	€ 668,123	€ 890,831	-€ 5,385,007	-€ 10,770,013	-€ 16,155,020	-€ 21,540,026
454	0.16	€ 255,631	€ 511,263	€ 766,894	##########	-€ 6,181,093	-€ 12,362,187	-€ 18,543,280	-€ 24,724,373
455	0.16	€ 247,804	€ 495,608	€ 743,412	€ 991,216	-€ 5,991,830	-€ 11,983,659	-€ 17,975,489	-€ 23,967,319
456	0.14	€ 220,260	€ 440,520	€ 660,781	€ 881,041	-€ 5,325,826	-€ 10,651,651	-€ 15,977,477	-€ 21,303,302
457	0.16	€ 244,554	€ 489,108	€ 733,663	€ 978,217	-€ 5,913,248	-€ 11,826,497	-€ 17,739,745	-€ 23,652,994
458	0.21	€ 324,616	€ 649,231	€ 973,847	##########	-€ 7,849,111	-€ 15,698,222	-€ 23,547,333	-€ 31,396,444
459	0.18	€ 289,767	€ 579,533	€ 869,300	##########	-€ 7,006,469	-€ 14,012,938	-€ 21,019,408	-€ 28,025,877
460	0.18	€ 287,761	€ 575,523	€ 863,284	##########	-€ 6,957,985	-€ 13,915,969	-€ 20,873,954	-€ 27,831,939
461	0.15	€ 243,771	€ 487,541	€ 731,312	€ 975,082	-€ 5,894,301	-€ 11,788,602	-€ 17,682,903	-€ 23,577,204
462	0.13	€ 203,074	€ 406,147	€ 609,221	€ 812,294	-€ 4,910,257	-€ 9,820,515	-€ 14,730,772	-€ 19,641,029
463	0.18	€ 287,647	€ 575,295	€ 862,942	##########	-€ 6,955,227	-€ 13,910,454	-€ 20,865,681	-€ 27,820,908
464	0.23	€ 361,941	€ 723,882	##########	##########	-€ 8,751,632	-€ 17,503,264	-€ 26,254,896	-€ 35,006,527
465	0.24	€ 373,156	€ 746,312	##########	##########	-€ 9,022,801	-€ 18,045,602	-€ 27,068,403	-€ 36,091,203
466	0.20	€ 322,747	€ 645,493	€ 968,240	##########	-€ 7,803,916	-€ 15,607,832	-€ 23,411,749	-€ 31,215,665
467	0.21	€ 330,069	€ 660,137	€ 990,206	##########	-€ 7,980,959	-€ 15,961,917	-€ 23,942,876	-€ 31,923,835
468	0.22	€ 349,843	€ 699,686	##########	##########	-€ 8,459,099	-€ 16,918,197	-€ 25,377,296	-€ 33,836,394
469	0.17	€ 267,735	€ 535,470	€ 803,206	##########	-€ 6,473,758	-€ 12,947,515	-€ 19,421,273	-€ 25,895,031
470	0.18	€ 276,003	€ 552,006	€ 828,009	##########	-€ 6,673,674	-€ 13,347,347	-€ 20,021,021	-€ 26,694,694
471	0.22	€ 345,493	€ 690,986	##########	##########	-€ 8,353,923	-€ 16,707,847	-€ 25,061,770	-€ 33,415,694
472	0.17	€ 266,729	€ 533,458	€ 800,187	##########	-€ 6,449,429	-€ 12,898,858	-€ 19,348,287	-€ 25,797,715
473	0.20	€ 308,897	€ 617,793	€ 926,690	##########	-€ 7,469,029	-€ 14,938,058	-€ 22,407,087	-€ 29,876,116
474	0.23	€ 357,650	€ 715,301	##########	##########	-€ 8,647,881	-€ 17,295,762	-€ 25,943,643	-€ 34,591,524
475	0.17	€ 268,722	€ 537,444	€ 806,165	##########	-€ 6,497,613	-€ 12,995,227	-€ 19,492,840	-€ 25,990,454
476	0.15	€ 242,129	€ 484,258	€ 726,388	€ 968,517	-€ 5,854,612	-€ 11,709,224	-€ 17,563,837	-€ 23,418,449
477	0.20	€ 318,838	€ 637,676	€ 956,514	##########	-€ 7,709,411	-€ 15,418,821	-€ 23,128,232	-€ 30,837,643
478	0.23	€ 358,510	€ 717,020	##########	##########	-€ 8,668,670	-€ 17,337,340	-€ 26,006,010	-€ 34,674,680
479	0.21	€ 326,043	€ 652,086	€ 978,129	##########	-€ 7,883,620	-€ 15,767,240	-€ 23,650,860	-€ 31,534,480
480	0.17	€ 269,347	€ 538,694	€ 808,041	##########	-€ 6,512,733	-€ 13,025,466	-€ 19,538,199	-€ 26,050,931
481	0.17	€ 264,251	€ 528,502	€ 792,753	##########	-€ 6,389,508	-€ 12,779,017	-€ 19,168,525	-€ 25,558,034
482	0.18	€ 291,005	€ 582,009	€ 873,014	##########	-€ 7,036,404	-€ 14,072,808	-€ 21,109,211	-€ 28,145,615
483	0.17	€ 264,493	€ 528,986	€ 793,479	##########	-€ 6,395,365	-€ 12,790,731	-€ 19,186,096	-€ 25,581,461
484	0.27	€ 420,531	€ 841,062	##########	##########	-€ 10,168,316	-€ 20,336,632	-€ 30,504,947	-€ 40,673,263
485	0.21	€ 326,907	€ 653,814	€ 980,721	##########	-€ 7,904,516	-€ 15,809,032	-€ 23,713,548	-€ 31,618,064
486	0.15	€ 238,702	€ 477,404	€ 716,106	€ 954,808	-€ 5,771,741	-€ 11,543,482	-€ 17,315,223	-€ 23,086,963
487	0.22	€ 340,173	€ 680,345	##########	##########	-€ 8,225,275	-€ 16,450,550	-€ 24,675,826	-€ 32,901,101
488	0.21	€ 326,948	€ 653,895	€ 980,843	##########	-€ 7,905,498	-€ 15,810,997	-€ 23,716,495	-€ 31,621,994
489	0.14	€ 216,721	€ 433,442	€ 650,163	€ 866,885	-€ 5,240,253	-€ 10,480,507	-€ 15,720,760	-€ 20,961,013
490	0.15	€ 234,045	€ 468,090	€ 702,135	€ 936,180	-€ 5,659,141	-€ 11,318,282	-€ 16,977,423	-€ 22,636,564
491	0.21	€ 326,564	€ 653,127	€ 979,691	##########	-€ 7,896,212	-€ 15,792,424	-€ 23,688,636	-€ 31,584,848
492	0.16	€ 259,557	€ 519,115	€ 778,672	##########	-€ 6,276,021	-€ 12,552,042	-€ 18,828,063	-€ 25,104,084
493	0.20	€ 313,212	€ 626,424	€ 939,636	##########	-€ 7,573,376	-€ 15,146,751	-€ 22,720,127	-€ 30,293,503
494	0.19	€ 305,116	€ 610,232	€ 915,349	##########	-€ 7,377,620	-€ 14,755,241	-€ 22,132,861	-€ 29,510,481
495	0.29	€ 459,367	€ 918,734	##########	##########	-€ 11,107,361	-€ 22,214,722	-€ 33,322,084	-€ 44,429,445
496	0.15	€ 231,564	€ 463,129	€ 694,693	€ 926,257	-€ 5,599,157	-€ 11,198,313	-€ 16,797,470	-€ 22,396,627
497	0.18	€ 290,561	€ 581,121	€ 871,682	##########	-€ 7,025,668	-€ 14,051,337	-€ 21,077,005	-€ 28,102,673
498	0.19	€ 301,773	€ 603,547	€ 905,320	##########	-€ 7,296,791	-€ 14,593,582	-€ 21,890,374	-€ 29,187,165
499	0.15	€ 242,227	€ 484,453	€ 726,680	€ 968,907	-€ 5,856,971	-€ 11,713,941	-€ 17,570,912	-€ 23,427,883
500	0.15	€ 243,094	€ 486,188	€ 729,282	€ 972,376	-€ 5,877,944	-€ 11,755,887	-€ 17,633,831	-€ 23,511,775
501	0.21	€ 334,524	€ 669,048	##########	##########	-€ 8,088,693	-€ 16,177,386	-€ 24,266,079	-€ 32,354,772
502	0.16	€ 246,508	€ 493,016	€ 739,524	€ 986,032	-€ 5,960,488	-€ 11,920,976	-€ 17,881,464	-€ 23,841,952
503	0.22	€ 347,942	€ 695,884	##########	##########	-€ 8,413,135	-€ 16,826,270	-€ 25,239,406	-€ 33,652,541
504	0.10	€ 152,803	€ 305,607	€ 458,410	€ 611,213	-€ 3,694,737	-€ 7,389,475	-€ 11,084,212	-€ 14,778,950
505	0.25	€ 394,428	€ 788,856	##########	##########	-€ 9,537,158	-€ 19,074,315	-€ 28,611,473	-€ 38,148,631
506	0.21	€ 329,146	€ 658,292	€ 987,438	##########	-€ 7,958,656	-€ 15,917,312	-€ 23,875,967	-€ 31,834,623

507	0.20	€ 321,906	€ 643,811	€ 965,717	##########	-€ 7,783,581	-€ 15,567,161	-€ 23,350,742	-€ 31,134,323
508	0.21	€ 330,935	€ 661,871	€ 992,806	##########	-€ 8,001,922	-€ 16,003,844	-€ 24,005,765	-€ 32,007,687
509	0.22	€ 349,185	€ 698,370	##########	##########	-€ 8,443,189	-€ 16,886,379	-€ 25,329,568	-€ 33,772,757
510	0.15	€ 229,683	€ 459,366	€ 689,049	€ 918,733	-€ 5,553,671	-€ 11,107,341	-€ 16,661,012	-€ 22,214,683
511	0.13	€ 211,526	€ 423,051	€ 634,577	€ 846,102	-€ 5,114,625	-€ 10,229,250	-€ 15,343,874	-€ 20,458,499
512	0.18	€ 277,311	€ 554,622	€ 831,933	##########	-€ 6,705,296	-€ 13,410,592	-€ 20,115,888	-€ 26,821,183
513	0.21	€ 324,534	€ 649,069	€ 973,603	##########	-€ 7,847,144	-€ 15,694,288	-€ 23,541,432	-€ 31,388,576
514	0.20	€ 311,823	€ 623,646	€ 935,470	##########	-€ 7,539,794	-€ 15,079,587	-€ 22,619,381	-€ 30,159,174
515	0.17	€ 274,805	€ 549,611	€ 824,416	##########	-€ 6,644,711	-€ 13,289,423	-€ 19,934,134	-€ 26,578,846
516	0.19	€ 296,156	€ 592,312	€ 888,467	##########	-€ 7,160,960	-€ 14,321,920	-€ 21,482,880	-€ 28,643,840
517	0.19	€ 302,529	€ 605,058	€ 907,587	##########	-€ 7,315,065	-€ 14,630,130	-€ 21,945,194	-€ 29,260,259
518	0.09	€ 145,775	€ 291,549	€ 437,324	€ 583,099	-€ 3,524,787	-€ 7,049,574	-€ 10,574,362	-€ 14,099,149
519	0.17	€ 267,861	€ 535,723	€ 803,584	##########	-€ 6,476,809	-€ 12,953,618	-€ 19,430,426	-€ 25,907,235
520	0.16	€ 253,761	€ 507,523	€ 761,284	##########	-€ 6,135,877	-€ 12,271,753	-€ 18,407,630	-€ 24,543,507
521	0.22	€ 348,175	€ 696,349	##########	##########	-€ 8,418,759	-€ 16,837,517	-€ 25,256,276	-€ 33,675,034
522	0.16	€ 256,164	€ 512,329	€ 768,493	##########	-€ 6,193,979	-€ 12,387,959	-€ 18,581,938	-€ 24,775,917
523	0.17	€ 266,426	€ 532,852	€ 799,278	##########	-€ 6,442,099	-€ 12,884,199	-€ 19,326,298	-€ 25,768,398
524	0.14	€ 220,067	€ 440,133	€ 660,200	€ 880,267	-€ 5,321,148	-€ 10,642,296	-€ 15,963,444	-€ 21,284,592
525	0.16	€ 250,412	€ 500,825	€ 751,237	##########	-€ 6,054,897	-€ 12,109,794	-€ 18,164,691	-€ 24,219,588
526	0.25	€ 391,232	€ 782,465	##########	##########	-€ 9,459,881	-€ 18,919,762	-€ 28,379,644	-€ 37,839,525
527	0.22	€ 340,208	€ 680,417	##########	##########	-€ 8,226,135	-€ 16,452,271	-€ 24,678,406	-€ 32,904,541
528	0.23	€ 361,387	€ 722,774	##########	##########	-€ 8,738,228	-€ 17,476,456	-€ 26,214,684	-€ 34,952,912
529	0.25	€ 391,896	€ 783,791	##########	##########	-€ 9,475,920	-€ 18,951,840	-€ 28,427,760	-€ 37,903,681
530	0.20	€ 322,737	€ 645,475	€ 968,212	##########	-€ 7,803,692	-€ 15,607,383	-€ 23,411,075	-€ 31,214,767
531	0.16	€ 250,638	€ 501,276	€ 751,914	##########	-€ 6,060,352	-€ 12,120,703	-€ 18,181,055	-€ 24,241,406
532	0.17	€ 268,287	€ 536,574	€ 804,861	##########	-€ 6,487,102	-€ 12,974,204	-€ 19,461,306	-€ 25,948,408
533	0.14	€ 224,047	€ 448,095	€ 672,142	€ 896,189	-€ 5,417,397	-€ 10,834,795	-€ 16,252,192	-€ 21,669,590
534	0.17	€ 272,356	€ 544,713	€ 817,069	##########	-€ 6,585,494	-€ 13,170,988	-€ 19,756,483	-€ 26,341,977
535	0.16	€ 249,749	€ 499,499	€ 749,248	€ 998,998	-€ 6,038,869	-€ 12,077,737	-€ 18,116,606	-€ 24,155,474
536	0.15	€ 238,842	€ 477,684	€ 716,526	€ 955,368	-€ 5,775,128	-€ 11,550,256	-€ 17,325,384	-€ 23,100,512
537	0.16	€ 247,203	€ 494,406	€ 741,608	€ 988,811	-€ 5,977,290	-€ 11,954,580	-€ 17,931,870	-€ 23,909,160
538	0.15	€ 231,082	€ 462,164	€ 693,246	€ 924,328	-€ 5,587,494	-€ 11,174,988	-€ 16,762,483	-€ 22,349,977
539	0.16	€ 258,985	€ 517,969	€ 776,954	##########	-€ 6,262,172	-€ 12,524,344	-€ 18,786,516	-€ 25,048,688
540	0.21	€ 324,741	€ 649,482	€ 974,223	##########	-€ 7,852,143	-€ 15,704,286	-€ 23,556,429	-€ 31,408,572
541	0.23	€ 363,862	€ 727,723	##########	##########	-€ 8,798,066	-€ 17,596,131	-€ 26,394,197	-€ 35,192,263
542	0.22	€ 346,511	€ 693,022	##########	##########	-€ 8,378,535	-€ 16,757,070	-€ 25,135,606	-€ 33,514,141
543	0.25	€ 392,366	€ 784,732	##########	##########	-€ 9,487,295	-€ 18,974,591	-€ 28,461,886	-€ 37,949,182
544	0.20	€ 309,793	€ 619,587	€ 929,380	##########	-€ 7,490,714	-€ 14,981,429	-€ 22,472,143	-€ 29,962,857
545	0.17	€ 271,679	€ 543,358	€ 815,037	##########	-€ 6,569,116	-€ 13,138,232	-€ 19,707,349	-€ 26,276,465
546	0.19	€ 299,590	€ 599,179	€ 898,769	##########	-€ 7,243,989	-€ 14,487,979	-€ 21,731,968	-€ 28,975,957
547	0.16	€ 257,532	€ 515,063	€ 772,595	##########	-€ 6,227,038	-€ 12,454,075	-€ 18,681,113	-€ 24,908,150
548	0.20	€ 313,132	€ 626,265	€ 939,397	##########	-€ 7,571,447	-€ 15,142,894	-€ 22,714,342	-€ 30,285,789
549	0.21	€ 337,819	€ 675,637	##########	##########	-€ 8,168,353	-€ 16,336,706	-€ 24,505,059	-€ 32,673,412
550	0.20	€ 312,465	€ 624,929	€ 937,394	##########	-€ 7,555,300	-€ 15,110,599	-€ 22,665,899	-€ 30,221,198
551	0.22	€ 354,120	€ 708,239	##########	##########	-€ 8,562,510	-€ 17,125,021	-€ 25,687,531	-€ 34,250,042
552	0.14	€ 215,759	€ 431,518	€ 647,277	€ 863,037	-€ 5,216,992	-€ 10,433,984	-€ 15,650,977	-€ 20,867,969
553	0.20	€ 315,779	€ 631,558	€ 947,337	##########	-€ 7,635,446	-€ 15,270,893	-€ 22,906,339	-€ 30,541,786
554	0.16	€ 253,404	€ 506,808	€ 760,213	##########	-€ 6,127,239	-€ 12,254,478	-€ 18,381,718	-€ 24,508,957
555	0.17	€ 260,059	€ 520,118	€ 780,177	##########	-€ 6,288,152	-€ 12,576,304	-€ 18,864,456	-€ 25,152,609
556	0.16	€ 252,783	€ 505,567	€ 758,350	##########	-€ 6,112,227	-€ 12,224,453	-€ 18,336,680	-€ 24,448,906
557	0.12	€ 185,632	€ 371,265	€ 556,897	€ 742,529	-€ 4,488,533	-€ 8,977,066	-€ 13,465,599	-€ 17,954,133
558	0.28	€ 439,012	€ 878,024	##########	##########	-€ 10,615,175	-€ 21,230,350	-€ 31,845,525	-€ 42,460,699
559	0.18	€ 278,226	€ 556,451	€ 834,677	##########	-€ 6,727,413	-€ 13,454,826	-€ 20,182,239	-€ 26,909,652
560	0.21	€ 330,191	€ 660,382	€ 990,573	##########	-€ 7,983,917	-€ 15,967,833	-€ 23,951,750	-€ 31,935,667
561	0.14	€ 219,521	€ 439,043	€ 658,564	€ 878,085	-€ 5,307,959	-€ 10,615,919	-€ 15,923,878	-€ 21,231,837
562	0.22	€ 342,508	€ 685,017	##########	##########	-€ 8,281,753	-€ 16,563,506	-€ 24,845,258	-€ 33,127,011
563	0.20	€ 316,420	€ 632,840	€ 949,259	##########	-€ 7,650,938	-€ 15,301,875	-€ 22,952,813	-€ 30,603,751
564	0.15	€ 233,360	€ 466,719	€ 700,079	€ 933,439	-€ 5,642,569	-€ 11,285,137	-€ 16,927,706	-€ 22,570,274
565	0.16	€ 259,727	€ 519,453	€ 779,180	##########	-€ 6,280,112	-€ 12,560,224	-€ 18,840,336	-€ 25,120,448
566	0.15	€ 243,163	€ 486,326	€ 729,490	€ 972,653	-€ 5,879,614	-€ 11,759,229	-€ 17,638,843	-€ 23,518,457
567	0.27	€ 417,792	€ 835,583	##########	##########	-€ 10,102,080	-€ 20,204,161	-€ 30,306,241	-€ 40,408,321
568	0.22	€ 339,254	€ 678,507	##########	##########	-€ 8,203,053	-€ 16,406,106	-€ 24,609,159	-€ 32,812,212
569	0.16	€ 250,686	€ 501,372	€ 752,059	##########	-€ 6,061,518	-€ 12,123,035	-€ 18,184,553	-€ 24,246,070
570	0.18	€ 285,242	€ 570,485	€ 855,727	##########	-€ 6,897,077	-€ 13,794,154	-€ 20,691,231	-€ 27,588,308
571	0.18	€ 287,705	€ 575,410	€ 863,115	##########	-€ 6,956,623	-€ 13,913,245	-€ 20,869,868	-€ 27,826,490
572	0.22	€ 341,350	€ 682,700	##########	##########	-€ 8,253,736	-€ 16,507,472	-€ 24,761,208	-€ 33,014,944
573	0.20	€ 308,665	€ 617,330	€ 925,995	##########	-€ 7,463,425	-€ 14,926,850	-€ 22,390,275	-€ 29,853,700
574	0.28	€ 441,086	€ 882,173	##########	##########	-€ 10,665,337	-€ 21,330,675	-€ 31,996,012	-€ 42,661,349
575	0.12	€ 189,223	€ 378,447	€ 567,670	€ 756,894	-€ 4,575,367	-€ 9,150,734	-€ 13,726,101	-€ 18,301,468
576	0.18	€ 290,219	€ 580,438	€ 870,656	##########	-€ 7,017,404	-€ 14,034,808	-€ 21,052,212	-€ 28,069,616
577	0.22	€ 351,309	€ 702,618	##########	##########	-€ 8,494,549	-€ 16,989,098	-€ 25,483,648	-€ 33,978,197
578	0.19	€ 303,149	€ 606,297	€ 909,446	##########	-€ 7,330,046	-€ 14,660,091	-€ 21,990,137	-€ 29,320,182
579	0.19	€ 300,900	€ 601,799	€ 902,699	##########	-€ 7,275,661	-€ 14,551,323	-€ 21,826,984	-€ 29,102,645
580	0.11	€ 165,858	€ 331,716	€ 497,574	€ 663,432	-€ 4,010,398	-€ 8,020,795	-€ 12,031,193	-€ 16,041,590
581	0.28	€ 441,292	€ 882,584	##########	##########	-€ 10,670,308	-€ 21,340,615	-€ 32,010,923	-€ 42,681,231
582	0.17	€ 271,623	€ 543,246	€ 814,869	##########	-€ 6,567,761	-€ 13,135,522	-€ 19,703,283	-€ 26,271,044
583	0.13	€ 201,098	€ 402,195	€ 603,293	€ 804,391	-€ 4,862,481	-€ 9,724,963	-€ 14,587,444	-€ 19,449,925
584	0.21	€ 335,841	€ 671,682	##########	##########	-€ 8,120,541	-€ 16,241,083	-€ 24,361,624	-€ 32,482,166
585	0.26	€ 403,461	€ 806,923	##########	##########	-€ 9,755,577	-€ 19,511,153	-€ 29,266,730	-€ 39,022,307
586	0.22	€ 340,450	€ 680,899	##########	##########	-€ 8,231,971	-€ 16,463,942	-€ 24,695,913	-€ 32,927,884
587	0.13	€ 210,761	€ 421,521	€ 632,282	€ 843,042	-€ 5,096,127	-€ 10,192,255	-€ 15,288,382	-€ 20,384,510
588	0.24	€ 375,542	€ 751,085	##########	##########	-€ 9,080,502	-€ 18,161,004	-€ 27,241,506	-€ 36,322,008
589	0.20	€ 308,275	€ 616,551	€ 924,826	##########	-€ 7,454,007	-€ 14,908,014	-€ 22,362,022	-€ 29,816,029
590	0.20	€ 314,869	€ 629,739	€ 944,608	##########	-€ 7,613,450	-€ 15,226,900	-€ 22,840,350	-€ 30,453,799
591	0.15	€ 236,720	€ 473,441	€ 710,161	€ 946,881	-€ 5,723,827	-€ 11,447,654	-€ 17,171,480	-€ 22,895,307
592	0.20	€ 308,484	€ 616,967	€ 925,451	##########	-€ 7,459,043	-€ 14,918,086	-€ 22,377,129	-€ 29,836,172
593	0.19	€ 301,275	€ 602,549	€ 903,824	##########	-€ 7,284,729	-€ 14,569,458	-€ 21,854,187	-€ 29,138,917
594	0.24	€ 375,047	€ 750,095	##########	##########	-€ 9,068,533	-€ 18,137,066	-€ 27,205,599	-€ 36,274,132
595	0.12	€ 184,279	€ 368,557	€ 552,836	€ 737,115	-€ 4,455,803	-€ 8,911,606	-€ 13,367,409	-€ 17,823,213
596	0.15	€ 231,821	€ 463,643	€ 695,464	€ 927,286	-€ 5,605,373	-€ 11,210,746	-€ 16,816,119	-€ 22,421,493
597	0.18	€ 286,704	€ 573,408	€ 860,112	##########	-€ 6,932,419	-€ 13,864,837	-€ 20,797,256	-€ 27,729,675
598	0.15	€ 230,745	€ 461,490	€ 692,235	€ 922,981	-€ 5,579,349	-€ 11,158,698	-€ 16,738,046	-€ 22,317,395
599	0.15	€ 228,695	€ 457,390	€ 686,085	€ 914,780	-€ 5,529,780	-€ 11,059,559	-€ 16,589,339	-€ 22,119,118
600	0.19	€ 295,252	€ 590,503	€ 885,755	##########	-€ 7,139,096	-€ 14,278,193	-€ 21,417,289	-€ 28,556,386
601	0.14	€ 215,548	€ 431,097	€ 646,645	€ 862,193	-€ 5,211,893	-€ 10,423,786	-€ 15,635,679	-€ 20,847,572
602	0.12	€ 184,802	€ 369,604	€ 554,406	€ 739,207	-€ 4,468,454	-€ 8,936,908	-€ 13,405,362	-€ 17,873,816
603	0.23	€ 361,090	€ 722,179	##########	##########	-€ 8,731,037	-€ 17,462,074	-€ 26,193,112	-€ 34,924,149
604	0.16	€ 255,916	€ 511,833	€ 767,749	##########	-€ 6,187,984	-€ 12,375,969	-€ 18,563,953	-€ 24,751,938
605	0.23	€ 365,499	€ 730,998	##########	##########	-€ 8,837,656	-€ 17,675,313	-€ 26,512,969	-€ 35,350,625
606	0.21	€ 336,059	€ 672,117	##########	##########	-€ 8,125,795	-€ 16,251,590	-€ 24,377,385	-€ 32,503,179
607	0.15	€ 241,091	€ 482,181	€ 723,272	€ 964,362	-€ 5,829,500	-€ 11,658,999	-€ 17,488,499	-€ 23,317,999
608	0.24	€ 382,188	€ 764,375	##########	##########	-€ 9,241,182	-€ 18,482,364	-€ 27,723,546	-€ 36,964,728

609	0.15	€ 243,841	€ 487,682	€ 731,523	€ 975,365	-€ 5,896,007	-€ 11,792,014	-€ 17,688,021	-€ 23,584,028
610	0.14	€ 222,890	€ 445,779	€ 668,669	€ 891,559	-€ 5,389,407	-€ 10,778,814	-€ 16,168,220	-€ 21,557,627
611	0.21	€ 329,032	€ 658,065	€ 987,097	##########	-€ 7,955,905	-€ 15,911,810	-€ 23,867,715	-€ 31,823,620
612	0.18	€ 280,015	€ 560,030	€ 840,045	##########	-€ 6,770,679	-€ 13,541,358	-€ 20,312,037	-€ 27,082,717
613	0.28	€ 442,223	€ 884,445	##########	##########	-€ 10,692,812	-€ 21,385,624	-€ 32,078,436	-€ 42,771,249
614	0.20	€ 308,748	€ 617,497	€ 926,245	##########	-€ 7,465,446	-€ 14,930,893	-€ 22,396,339	-€ 29,861,785
615	0.27	€ 431,126	€ 862,252	##########	##########	-€ 10,424,499	-€ 20,848,999	-€ 31,273,498	-€ 41,697,998
616	0.21	€ 335,516	€ 671,032	##########	##########	-€ 8,112,675	-€ 16,225,349	-€ 24,338,024	-€ 32,450,698
617	0.13	€ 211,823	€ 423,645	€ 635,468	€ 847,291	-€ 5,121,809	-€ 10,243,619	-€ 15,365,428	-€ 20,487,237
618	0.10	€ 158,087	€ 316,175	€ 474,262	€ 632,349	-€ 3,822,504	-€ 7,645,009	-€ 11,467,513	-€ 15,290,017
619	0.14	€ 213,449	€ 426,897	€ 640,346	€ 853,795	-€ 5,161,126	-€ 10,322,252	-€ 15,483,378	-€ 20,644,504
620	0.12	€ 185,593	€ 371,186	€ 556,779	€ 742,372	-€ 4,487,586	-€ 8,975,172	-€ 13,462,758	-€ 17,950,344
621	0.11	€ 180,578	€ 361,157	€ 541,735	€ 722,314	-€ 4,366,332	-€ 8,732,663	-€ 13,098,995	-€ 17,465,327
622	0.24	€ 384,449	€ 768,897	##########	##########	-€ 9,295,855	-€ 18,591,710	-€ 27,887,564	-€ 37,183,419
623	0.24	€ 370,898	€ 741,797	##########	##########	-€ 8,968,212	-€ 17,936,425	-€ 26,904,637	-€ 35,872,850
624	0.22	€ 352,681	€ 705,362	##########	##########	-€ 8,527,723	-€ 17,055,445	-€ 25,583,168	-€ 34,110,890
625	0.25	€ 388,646	€ 777,291	##########	##########	-€ 9,397,337	-€ 18,794,675	-€ 28,192,012	-€ 37,589,349
626	0.25	€ 394,843	€ 789,685	##########	##########	-€ 9,547,180	-€ 19,094,360	-€ 28,641,539	-€ 38,188,719
627	0.20	€ 320,743	€ 641,486	€ 962,229	##########	-€ 7,755,469	-€ 15,510,939	-€ 23,266,408	-€ 31,021,877
628	0.16	€ 253,740	€ 507,480	€ 761,220	##########	-€ 6,135,361	-€ 12,270,723	-€ 18,406,084	-€ 24,541,446
629	0.21	€ 328,781	€ 657,563	€ 986,344	##########	-€ 7,949,835	-€ 15,899,671	-€ 23,849,506	-€ 31,799,342
630	0.13	€ 202,003	€ 404,005	€ 606,008	€ 808,011	-€ 4,884,365	-€ 9,768,731	-€ 14,653,096	-€ 19,537,462
631	0.22	€ 344,719	€ 689,438	##########	##########	-€ 8,335,209	-€ 16,670,417	-€ 25,005,626	-€ 33,340,834
632	0.13	€ 197,837	€ 395,673	€ 593,510	€ 791,347	-€ 4,783,633	-€ 9,567,266	-€ 14,350,899	-€ 19,134,532
633	0.25	€ 386,462	€ 772,923	##########	##########	-€ 9,344,527	-€ 18,689,053	-€ 28,033,580	-€ 37,378,106
634	0.26	€ 408,194	€ 816,388	##########	##########	-€ 9,870,013	-€ 19,740,026	-€ 29,610,039	-€ 39,480,052
635	0.29	€ 454,573	€ 909,145	##########	##########	-€ 10,991,434	-€ 21,982,867	-€ 32,974,301	-€ 43,965,734
636	0.21	€ 323,069	€ 646,139	€ 969,208	##########	-€ 7,811,723	-€ 15,623,446	-€ 23,435,169	-€ 31,246,892
637	0.17	€ 272,675	€ 545,349	€ 818,024	##########	-€ 6,593,189	-€ 13,186,379	-€ 19,779,568	-€ 26,372,758
638	0.19	€ 299,503	€ 599,005	€ 898,508	##########	-€ 7,241,882	-€ 14,483,763	-€ 21,725,645	-€ 28,967,527
639	0.20	€ 311,839	€ 623,678	€ 935,517	##########	-€ 7,540,173	-€ 15,080,346	-€ 22,620,518	-€ 30,160,691
640	0.14	€ 220,061	€ 440,122	€ 660,183	€ 880,244	-€ 5,321,011	-€ 10,642,022	-€ 15,963,033	-€ 21,284,044
641	0.17	€ 268,486	€ 536,973	€ 805,459	##########	-€ 6,491,922	-€ 12,983,843	-€ 19,475,765	-€ 25,967,686
642	0.17	€ 268,075	€ 536,150	€ 804,225	##########	-€ 6,481,977	-€ 12,963,954	-€ 19,445,931	-€ 25,927,908
643	0.26	€ 415,415	€ 830,830	##########	##########	-€ 10,044,616	-€ 20,089,233	-€ 30,133,849	-€ 40,178,466
644	0.21	€ 326,913	€ 653,826	€ 980,739	##########	-€ 7,904,660	-€ 15,809,320	-€ 23,713,980	-€ 31,618,641
645	0.13	€ 199,454	€ 398,907	€ 598,361	€ 797,815	-€ 4,822,731	-€ 9,645,462	-€ 14,468,193	-€ 19,290,924
646	0.28	€ 440,892	€ 881,783	##########	##########	-€ 10,660,631	-€ 21,321,263	-€ 31,981,894	-€ 42,642,526
647	0.19	€ 291,529	€ 583,057	€ 874,586	##########	-€ 7,049,077	-€ 14,098,154	-€ 21,147,231	-€ 28,196,308
648	0.23	€ 367,394	€ 734,789	##########	##########	-€ 8,883,485	-€ 17,766,970	-€ 26,650,455	-€ 35,533,940
649	0.24	€ 379,995	€ 759,989	##########	##########	-€ 9,188,157	-€ 18,376,314	-€ 27,564,471	-€ 36,752,628
650	0.21	€ 325,907	€ 651,815	€ 977,722	##########	-€ 7,880,344	-€ 15,760,687	-€ 23,641,031	-€ 31,521,374
651	0.21	€ 327,868	€ 655,736	€ 983,605	##########	-€ 7,927,755	-€ 15,855,510	-€ 23,783,266	-€ 31,711,021
652	0.21	€ 329,338	€ 658,676	€ 988,014	##########	-€ 7,963,291	-€ 15,926,582	-€ 23,889,874	-€ 31,853,165
653	0.20	€ 308,307	€ 616,615	€ 924,922	##########	-€ 7,454,784	-€ 14,909,568	-€ 22,364,351	-€ 29,819,135
654	0.22	€ 343,498	€ 686,995	##########	##########	-€ 8,305,671	-€ 16,611,342	-€ 24,917,013	-€ 33,222,684
655	0.22	€ 338,619	€ 677,239	##########	##########	-€ 8,187,716	-€ 16,375,431	-€ 24,563,147	-€ 32,750,863
656	0.17	€ 269,879	€ 539,758	€ 809,638	##########	-€ 6,525,600	-€ 13,051,200	-€ 19,576,799	-€ 26,102,399
657	0.23	€ 354,448	€ 708,897	##########	##########	-€ 8,570,457	-€ 17,140,915	-€ 25,711,372	-€ 34,281,830
658	0.14	€ 227,701	€ 455,403	€ 683,104	€ 910,805	-€ 5,505,751	-€ 11,011,502	-€ 16,517,252	-€ 22,023,003
659	0.17	€ 275,221	€ 550,442	€ 825,663	##########	-€ 6,654,766	-€ 13,309,532	-€ 19,964,298	-€ 26,619,064
660	0.18	€ 277,101	€ 554,202	€ 831,302	##########	-€ 6,700,215	-€ 13,400,431	-€ 20,100,646	-€ 26,800,861
661	0.21	€ 335,634	€ 671,267	##########	##########	-€ 8,115,521	-€ 16,231,042	-€ 24,346,563	-€ 32,462,084
662	0.22	€ 345,127	€ 690,253	##########	##########	-€ 8,345,062	-€ 16,690,123	-€ 25,035,185	-€ 33,380,246
663	0.22	€ 343,419	€ 686,837	##########	##########	-€ 8,303,761	-€ 16,607,522	-€ 24,911,282	-€ 33,215,043
664	0.24	€ 376,182	€ 752,365	##########	##########	-€ 9,095,978	-€ 18,191,957	-€ 27,287,935	-€ 36,383,913
665	0.20	€ 312,949	€ 625,899	€ 938,848	##########	-€ 7,567,021	-€ 15,134,043	-€ 22,701,064	-€ 30,268,085
666	0.25	€ 392,474	€ 784,948	##########	##########	-€ 9,489,905	-€ 18,979,811	-€ 28,469,716	-€ 37,959,621
667	0.19	€ 291,407	€ 582,814	€ 874,220	##########	-€ 7,046,130	-€ 14,092,261	-€ 21,138,391	-€ 28,184,521
668	0.16	€ 244,866	€ 489,731	€ 734,597	€ 979,463	-€ 5,920,779	-€ 11,841,559	-€ 17,762,338	-€ 23,683,118
669	0.16	€ 251,295	€ 502,590	€ 753,885	##########	-€ 6,076,236	-€ 12,152,473	-€ 18,228,709	-€ 24,304,945
670	0.16	€ 257,275	€ 514,549	€ 771,824	##########	-€ 6,220,825	-€ 12,441,651	-€ 18,662,476	-€ 24,883,302
671	0.18	€ 281,914	€ 563,828	€ 845,742	##########	-€ 6,816,594	-€ 13,633,188	-€ 20,449,781	-€ 27,266,375
672	0.18	€ 285,168	€ 570,336	€ 855,503	##########	-€ 6,895,273	-€ 13,790,547	-€ 20,685,820	-€ 27,581,094
673	0.14	€ 215,035	€ 430,070	€ 645,104	€ 860,139	-€ 5,199,478	-€ 10,398,956	-€ 15,598,434	-€ 20,797,912
674	0.17	€ 273,293	€ 546,586	€ 819,878	##########	-€ 6,608,138	-€ 13,216,275	-€ 19,824,413	-€ 26,432,551
675	0.20	€ 322,086	€ 644,171	€ 966,257	##########	-€ 7,787,933	-€ 15,575,866	-€ 23,363,799	-€ 31,151,732
676	0.24	€ 381,023	€ 762,045	##########	##########	-€ 9,213,012	-€ 18,426,024	-€ 27,639,036	-€ 36,852,048
677	0.27	€ 431,622	€ 863,243	##########	##########	-€ 10,436,485	-€ 20,872,970	-€ 31,309,455	-€ 41,745,940
678	0.25	€ 393,448	€ 786,897	##########	##########	-€ 9,513,467	-€ 19,026,934	-€ 28,540,401	-€ 38,053,868
679	0.26	€ 404,268	€ 808,536	##########	##########	-€ 9,775,083	-€ 19,550,166	-€ 29,325,248	-€ 39,100,331
680	0.17	€ 270,207	€ 540,414	€ 810,621	##########	-€ 6,533,524	-€ 13,067,048	-€ 19,600,572	-€ 26,134,095
681	0.22	€ 353,298	€ 706,596	##########	##########	-€ 8,542,640	-€ 17,085,280	-€ 25,627,920	-€ 34,170,561
682	0.19	€ 297,468	€ 594,937	€ 892,405	##########	-€ 7,192,698	-€ 14,385,396	-€ 21,578,093	-€ 28,770,791
683	0.18	€ 277,399	€ 554,799	€ 832,198	##########	-€ 6,707,433	-€ 13,414,866	-€ 20,122,299	-€ 26,829,732
684	0.19	€ 305,234	€ 610,469	€ 915,703	##########	-€ 7,380,478	-€ 14,760,955	-€ 22,141,433	-€ 29,521,911
685	0.21	€ 328,286	€ 656,571	€ 984,857	##########	-€ 7,937,852	-€ 15,875,704	-€ 23,813,556	-€ 31,751,407
686	0.20	€ 314,298	€ 628,596	€ 942,894	##########	-€ 7,599,632	-€ 15,199,264	-€ 22,798,896	-€ 30,398,529
687	0.18	€ 287,120	€ 574,240	€ 861,360	##########	-€ 6,942,480	-€ 13,884,961	-€ 20,827,441	-€ 27,769,921
688	0.24	€ 371,756	€ 743,511	##########	##########	-€ 8,988,943	-€ 17,977,885	-€ 26,966,828	-€ 35,955,771
689	0.17	€ 268,499	€ 536,999	€ 805,498	##########	-€ 6,492,235	-€ 12,984,470	-€ 19,476,705	-€ 25,968,940
690	0.13	€ 198,085	€ 396,170	€ 594,255	€ 792,340	-€ 4,789,634	-€ 9,579,268	-€ 14,368,902	-€ 19,158,536
691	0.24	€ 385,558	€ 771,117	##########	##########	-€ 9,322,690	-€ 18,645,379	-€ 27,968,069	-€ 37,290,759
692	0.24	€ 376,463	€ 752,927	##########	##########	-€ 9,102,772	-€ 18,205,544	-€ 27,308,317	-€ 36,411,089
693	0.20	€ 315,408	€ 630,816	€ 946,224	##########	-€ 7,626,473	-€ 15,252,946	-€ 22,879,420	-€ 30,505,893
694	0.16	€ 247,075	€ 494,151	€ 741,226	€ 988,302	-€ 5,974,211	-€ 11,948,422	-€ 17,922,633	-€ 23,896,844
695	0.13	€ 203,991	€ 407,982	€ 611,973	€ 815,964	-€ 4,932,442	-€ 9,864,885	-€ 14,797,327	-€ 19,729,770
696	0.26	€ 407,413	€ 814,826	##########	##########	-€ 9,851,128	-€ 19,702,255	-€ 29,553,383	-€ 39,404,510
697	0.15	€ 234,219	€ 468,437	€ 702,656	€ 936,875	-€ 5,663,337	-€ 11,326,675	-€ 16,990,012	-€ 22,653,350
698	0.18	€ 275,741	€ 551,483	€ 827,224	##########	-€ 6,667,346	-€ 13,334,692	-€ 20,002,037	-€ 26,669,383
699	0.19	€ 294,256	€ 588,511	€ 882,767	##########	-€ 7,115,016	-€ 14,230,032	-€ 21,345,048	-€ 28,460,064
700	0.15	€ 241,469	€ 482,938	€ 724,407	€ 965,876	-€ 5,838,650	-€ 11,677,299	-€ 17,515,949	-€ 23,354,599
701	0.29	€ 461,457	€ 922,913	##########	##########	-€ 11,157,883	-€ 22,315,766	-€ 33,473,649	-€ 44,631,531
702	0.19	€ 303,687	€ 607,373	€ 911,060	##########	-€ 7,343,054	-€ 14,686,109	-€ 22,029,163	-€ 29,372,218
703	0.16	€ 248,154	€ 496,308	€ 744,463	€ 992,617	-€ 6,000,295	-€ 12,000,590	-€ 18,000,885	-€ 24,001,180
704	0.12	€ 182,183	€ 364,365	€ 546,548	€ 728,730	-€ 4,405,119	-€ 8,810,239	-€ 13,215,358	-€ 17,620,478
705	0.22	€ 344,238	€ 688,475	##########	##########	-€ 8,323,566	-€ 16,647,133	-€ 24,970,699	-€ 33,294,265
706	0.29	€ 451,574	€ 903,149	##########	##########	-€ 10,918,934	-€ 21,837,869	-€ 32,756,803	-€ 43,675,737
707	0.26	€ 402,930	€ 805,859	##########	##########	-€ 9,742,721	-€ 19,485,442	-€ 29,228,164	-€ 38,970,885
708	0.16	€ 248,908	€ 497,815	€ 746,723	€ 995,631	-€ 6,018,513	-€ 12,037,026	-€ 18,055,539	-€ 24,074,051
709	0.18	€ 288,346	€ 576,691	€ 865,037	##########	-€ 6,972,111	-€ 13,944,221	-€ 20,916,332	-€ 27,888,443
710	0.24	€ 373,417	€ 746,835	##########	##########	-€ 9,029,124	-€ 18,058,247	-€ 27,087,371	-€ 36,116,495

711	0.18	€ 281,100	€ 562,201	€ 843,301	##########	-€ 6,796,921	-€ 13,593,842	-€ 20,390,763	-€ 27,187,684
712	0.18	€ 285,749	€ 571,498	€ 857,247	##########	-€ 6,909,327	-€ 13,818,653	-€ 20,727,980	-€ 27,637,307
713	0.21	€ 334,108	€ 668,216	##########	##########	-€ 8,078,635	-€ 16,157,269	-€ 24,235,904	-€ 32,314,539
714	0.19	€ 295,536	€ 591,072	€ 886,608	##########	-€ 7,145,971	-€ 14,291,942	-€ 21,437,913	-€ 28,583,885
715	0.22	€ 340,251	€ 680,502	##########	##########	-€ 8,227,163	-€ 16,454,326	-€ 24,681,489	-€ 32,908,652
716	0.18	€ 290,816	€ 581,632	€ 872,448	##########	-€ 7,031,841	-€ 14,063,683	-€ 21,095,524	-€ 28,127,366
717	0.19	€ 300,777	€ 601,555	€ 902,332	##########	-€ 7,272,707	-€ 14,545,414	-€ 21,818,120	-€ 29,090,827
718	0.21	€ 326,405	€ 652,811	€ 979,216	##########	-€ 7,892,385	-€ 15,784,771	-€ 23,677,156	-€ 31,569,542
719	0.24	€ 371,322	€ 742,644	##########	##########	-€ 8,978,458	-€ 17,956,917	-€ 26,935,375	-€ 35,913,834
720	0.26	€ 417,050	€ 834,099	##########	##########	-€ 10,084,139	-€ 20,168,278	-€ 30,252,417	-€ 40,336,556
721	0.20	€ 316,871	€ 633,742	€ 950,613	##########	-€ 7,661,849	-€ 15,323,698	-€ 22,985,547	-€ 30,647,397
722	0.23	€ 367,762	€ 735,525	##########	##########	-€ 8,892,385	-€ 17,784,771	-€ 26,677,156	-€ 35,569,541
723	0.17	€ 271,490	€ 542,980	€ 814,469	##########	-€ 6,564,542	-€ 13,129,085	-€ 19,693,627	-€ 26,258,169
724	0.19	€ 303,649	€ 607,297	€ 910,946	##########	-€ 7,342,135	-€ 14,684,271	-€ 22,026,406	-€ 29,368,542
725	0.19	€ 300,459	€ 600,918	€ 901,377	##########	-€ 7,265,013	-€ 14,530,025	-€ 21,795,038	-€ 29,060,050
726	0.20	€ 322,585	€ 645,169	€ 967,754	##########	-€ 7,800,002	-€ 15,600,004	-€ 23,400,006	-€ 31,200,007
727	0.21	€ 329,097	€ 658,195	€ 987,292	##########	-€ 7,957,479	-€ 15,914,958	-€ 23,872,437	-€ 31,829,916
728	0.19	€ 300,792	€ 601,584	€ 902,377	##########	-€ 7,273,066	-€ 14,546,132	-€ 21,819,197	-€ 29,092,263
729	0.16	€ 259,686	€ 519,373	€ 779,059	##########	-€ 6,279,141	-€ 12,558,281	-€ 18,837,422	-€ 25,116,562
730	0.15	€ 231,390	€ 462,780	€ 694,170	€ 925,560	-€ 5,594,942	-€ 11,189,883	-€ 16,784,825	-€ 22,379,766
731	0.19	€ 296,894	€ 593,788	€ 890,683	##########	-€ 7,178,813	-€ 14,357,626	-€ 21,536,439	-€ 28,715,252
732	0.21	€ 322,847	€ 645,693	€ 968,540	##########	-€ 7,806,335	-€ 15,612,670	-€ 23,419,006	-€ 31,225,341
733	0.22	€ 342,357	€ 684,714	##########	##########	-€ 8,278,096	-€ 16,556,192	-€ 24,834,288	-€ 33,112,383
734	0.18	€ 280,913	€ 561,825	€ 842,738	##########	-€ 6,792,381	-€ 13,584,763	-€ 20,377,144	-€ 27,169,525
735	0.19	€ 292,664	€ 585,328	€ 877,992	##########	-€ 7,076,530	-€ 14,153,060	-€ 21,229,590	-€ 28,306,121
736	0.22	€ 352,960	€ 705,921	##########	##########	-€ 8,534,475	-€ 17,068,950	-€ 25,603,424	-€ 34,137,899
737	0.18	€ 289,943	€ 579,885	€ 869,828	##########	-€ 7,010,725	-€ 14,021,449	-€ 21,032,174	-€ 28,042,899
738	0.13	€ 199,469	€ 398,938	€ 598,407	€ 797,876	-€ 4,823,099	-€ 9,646,197	-€ 14,469,296	-€ 19,292,394
739	0.21	€ 332,145	€ 664,291	€ 996,436	##########	-€ 8,031,178	-€ 16,062,356	-€ 24,093,534	-€ 32,124,711
740	0.30	€ 476,655	€ 953,311	##########	##########	-€ 11,525,383	-€ 23,050,766	-€ 34,576,149	-€ 46,101,532
741	0.25	€ 400,336	€ 800,673	##########	##########	-€ 9,680,017	-€ 19,360,033	-€ 29,040,050	-€ 38,720,066
742	0.22	€ 339,032	€ 678,063	##########	##########	-€ 8,197,686	-€ 16,395,372	-€ 24,593,058	-€ 32,790,744
743	0.22	€ 346,636	€ 693,272	##########	##########	-€ 8,381,559	-€ 16,763,118	-€ 25,144,677	-€ 33,526,237
744	0.29	€ 452,193	€ 904,385	##########	##########	-€ 10,933,881	-€ 21,867,762	-€ 32,801,644	-€ 43,735,525
745	0.22	€ 343,271	€ 686,542	##########	##########	-€ 8,300,195	-€ 16,600,390	-€ 24,900,584	-€ 33,200,779
746	0.17	€ 261,134	€ 522,268	€ 783,402	##########	-€ 6,314,140	-€ 12,628,279	-€ 18,942,419	-€ 25,256,558
747	0.21	€ 338,489	€ 676,977	##########	##########	-€ 8,184,553	-€ 16,369,106	-€ 24,553,660	-€ 32,738,213
748	0.15	€ 243,899	€ 487,798	€ 731,697	€ 975,596	-€ 5,897,406	-€ 11,794,812	-€ 17,692,218	-€ 23,589,625
749	0.19	€ 298,619	€ 597,239	€ 895,858	##########	-€ 7,220,531	-€ 14,441,061	-€ 21,661,592	-€ 28,882,122
750	0.20	€ 320,471	€ 640,942	€ 961,413	##########	-€ 7,748,898	-€ 15,497,795	-€ 23,246,693	-€ 30,995,590
751	0.16	€ 246,102	€ 492,204	€ 738,306	€ 984,408	-€ 5,950,672	-€ 11,901,344	-€ 17,852,016	-€ 23,802,688
752	0.16	€ 257,311	€ 514,623	€ 771,934	##########	-€ 6,221,711	-€ 12,443,423	-€ 18,665,134	-€ 24,886,846
753	0.17	€ 272,358	€ 544,716	€ 817,073	##########	-€ 6,585,531	-€ 13,171,061	-€ 19,756,592	-€ 26,342,123
754	0.17	€ 264,355	€ 528,710	€ 793,065	##########	-€ 6,392,026	-€ 12,784,053	-€ 19,176,079	-€ 25,568,106
755	0.23	€ 358,052	€ 716,104	##########	##########	-€ 8,657,593	-€ 17,315,185	-€ 25,972,778	-€ 34,630,371
756	0.17	€ 269,598	€ 539,196	€ 808,793	##########	-€ 6,518,794	-€ 13,037,589	-€ 19,556,383	-€ 26,075,177
757	0.24	€ 371,950	€ 743,899	##########	##########	-€ 8,993,630	-€ 17,987,261	-€ 26,980,891	-€ 35,974,521
758	0.17	€ 264,247	€ 528,494	€ 792,742	##########	-€ 6,389,419	-€ 12,778,838	-€ 19,168,257	-€ 25,557,676
759	0.15	€ 242,650	€ 485,300	€ 727,949	€ 970,599	-€ 5,867,199	-€ 11,734,398	-€ 17,601,597	-€ 23,468,796
760	0.21	€ 333,024	€ 666,048	€ 999,072	##########	-€ 8,052,418	-€ 16,104,835	-€ 24,157,253	-€ 32,209,670
761	0.14	€ 218,802	€ 437,605	€ 656,407	€ 875,209	-€ 5,290,574	-€ 10,581,148	-€ 15,871,722	-€ 21,162,297
762	0.20	€ 316,026	€ 632,052	€ 948,078	##########	-€ 7,641,411	-€ 15,282,823	-€ 22,924,234	-€ 30,565,646
763	0.24	€ 372,070	€ 744,140	##########	##########	-€ 8,996,545	-€ 17,993,090	-€ 26,989,635	-€ 35,986,180
764	0.12	€ 195,245	€ 390,490	€ 585,735	€ 780,980	-€ 4,720,969	-€ 9,441,938	-€ 14,162,906	-€ 18,883,875
765	0.12	€ 186,703	€ 373,406	€ 560,110	€ 746,813	-€ 4,514,428	-€ 9,028,857	-€ 13,543,285	-€ 18,057,714
766	0.20	€ 314,233	€ 628,467	€ 942,700	##########	-€ 7,598,067	-€ 15,196,134	-€ 22,794,200	-€ 30,392,267
767	0.17	€ 259,820	€ 519,641	€ 779,461	##########	-€ 6,282,381	-€ 12,564,762	-€ 18,847,143	-€ 25,129,524
768	0.25	€ 390,271	€ 780,543	##########	##########	-€ 9,436,645	-€ 18,873,291	-€ 28,309,936	-€ 37,746,582
769	0.18	€ 277,300	€ 554,600	€ 831,900	##########	-€ 6,705,036	-€ 13,410,071	-€ 20,115,107	-€ 26,820,142
770	0.16	€ 254,632	€ 509,263	€ 763,895	##########	-€ 6,156,919	-€ 12,313,838	-€ 18,470,757	-€ 24,627,676
771	0.27	€ 431,031	€ 862,063	##########	##########	-€ 10,422,210	-€ 20,844,420	-€ 31,266,630	-€ 41,688,840
772	0.19	€ 306,154	€ 612,308	€ 918,461	##########	-€ 7,402,709	-€ 14,805,417	-€ 22,208,126	-€ 29,610,835
773	0.19	€ 292,258	€ 584,517	€ 876,775	##########	-€ 7,066,723	-€ 14,133,446	-€ 21,200,169	-€ 28,266,892
774	0.15	€ 236,533	€ 473,066	€ 709,598	€ 946,131	-€ 5,719,293	-€ 11,438,586	-€ 17,157,879	-€ 22,877,173
775	0.15	€ 240,394	€ 480,789	€ 721,183	€ 961,578	-€ 5,812,668	-€ 11,625,335	-€ 17,438,003	-€ 23,250,671
776	0.17	€ 268,701	€ 537,402	€ 806,102	##########	-€ 6,497,105	-€ 12,994,211	-€ 19,491,316	-€ 25,988,422
777	0.22	€ 347,161	€ 694,323	##########	##########	-€ 8,394,261	-€ 16,788,522	-€ 25,182,783	-€ 33,577,044
778	0.20	€ 319,752	€ 639,505	€ 959,257	##########	-€ 7,731,517	-€ 15,463,034	-€ 23,194,551	-€ 30,926,068
779	0.21	€ 334,519	€ 669,037	##########	##########	-€ 8,088,563	-€ 16,177,125	-€ 24,265,688	-€ 32,354,250
780	0.19	€ 297,998	€ 595,996	€ 893,995	##########	-€ 7,205,509	-€ 14,411,018	-€ 21,616,527	-€ 28,822,036
781	0.22	€ 340,857	€ 681,715	##########	##########	-€ 8,241,827	-€ 16,483,655	-€ 24,725,482	-€ 32,967,309
782	0.20	€ 312,636	€ 625,272	€ 937,907	##########	-€ 7,559,441	-€ 15,118,883	-€ 22,678,324	-€ 30,237,766
783	0.18	€ 284,295	€ 568,590	€ 852,886	##########	-€ 6,874,173	-€ 13,748,346	-€ 20,622,519	-€ 27,496,693
784	0.13	€ 198,956	€ 397,912	€ 596,869	€ 795,825	-€ 4,810,703	-€ 9,621,405	-€ 14,432,108	-€ 19,242,811
785	0.16	€ 245,866	€ 491,732	€ 737,598	€ 983,463	-€ 5,944,964	-€ 11,889,927	-€ 17,834,891	-€ 23,779,855
786	0.23	€ 369,286	€ 738,573	##########	##########	-€ 8,929,232	-€ 17,858,465	-€ 26,787,697	-€ 35,716,929
787	0.18	€ 284,955	€ 569,910	€ 854,865	##########	-€ 6,890,127	-€ 13,780,254	-€ 20,670,381	-€ 27,560,508
788	0.16	€ 255,437	€ 510,873	€ 766,310	##########	-€ 6,176,384	-€ 12,352,767	-€ 18,529,151	-€ 24,705,534
789	0.24	€ 371,054	€ 742,107	##########	##########	-€ 8,971,969	-€ 17,943,937	-€ 26,915,906	-€ 35,887,875
790	0.21	€ 336,213	€ 672,425	##########	##########	-€ 8,129,520	-€ 16,259,041	-€ 24,388,561	-€ 32,518,082
791	0.22	€ 346,867	€ 693,734	##########	##########	-€ 8,387,137	-€ 16,774,274	-€ 25,161,410	-€ 33,548,547
792	0.21	€ 329,890	€ 659,780	€ 989,670	##########	-€ 7,976,638	-€ 15,953,276	-€ 23,929,915	-€ 31,906,553
793	0.11	€ 169,303	€ 338,607	€ 507,910	€ 677,214	-€ 4,093,706	-€ 8,187,412	-€ 12,281,118	-€ 16,374,825
794	0.27	€ 421,869	€ 843,739	##########	##########	-€ 10,200,678	-€ 20,401,355	-€ 30,602,033	-€ 40,802,711
795	0.21	€ 328,984	€ 657,968	€ 986,952	##########	-€ 7,954,736	-€ 15,909,471	-€ 23,864,207	-€ 31,818,942
796	0.25	€ 386,951	€ 773,903	##########	##########	-€ 9,356,370	-€ 18,712,739	-€ 28,069,109	-€ 37,425,478
797	0.28	€ 443,933	€ 887,865	##########	##########	-€ 10,734,159	-€ 21,468,318	-€ 32,202,477	-€ 42,936,637
798	0.23	€ 366,541	€ 733,081	##########	##########	-€ 8,862,843	-€ 17,725,686	-€ 26,588,529	-€ 35,451,372
799	0.14	€ 213,758	€ 427,515	€ 641,273	€ 855,030	-€ 5,168,595	-€ 10,337,190	-€ 15,505,784	-€ 20,674,379
800	0.14	€ 222,810	€ 445,620	€ 668,430	€ 891,240	-€ 5,387,479	-€ 10,774,958	-€ 16,162,437	-€ 21,549,916
801	0.24	€ 378,517	€ 757,035	##########	##########	-€ 9,152,438	-€ 18,304,876	-€ 27,457,314	-€ 36,609,753
802	0.21	€ 336,127	€ 672,255	##########	##########	-€ 8,127,459	-€ 16,254,918	-€ 24,382,376	-€ 32,509,835
803	0.24	€ 371,800	€ 743,600	##########	##########	-€ 8,990,019	-€ 17,980,037	-€ 26,970,056	-€ 35,960,075
804	0.16	€ 252,614	€ 505,228	€ 757,842	##########	-€ 6,108,135	-€ 12,216,269	-€ 18,324,404	-€ 24,432,538
805	0.18	€ 283,271	€ 566,543	€ 849,814	##########	-€ 6,849,417	-€ 13,698,834	-€ 20,548,250	-€ 27,397,667
806	0.20	€ 314,730	€ 629,460	€ 944,190	##########	-€ 7,610,078	-€ 15,220,156	-€ 22,830,233	-€ 30,440,311
807	0.18	€ 280,903	€ 561,805	€ 842,708	##########	-€ 6,792,144	-€ 13,584,287	-€ 20,376,431	-€ 27,168,575
808	0.17	€ 269,958	€ 539,916	€ 809,875	##########	-€ 6,527,510	-€ 13,055,021	-€ 19,582,531	-€ 26,110,041
809	0.20	€ 316,143	€ 632,286	€ 948,429	##########	-€ 7,644,245	-€ 15,288,490	-€ 22,932,735	-€ 30,576,980
810	0.14	€ 228,290	€ 456,581	€ 684,871	€ 913,161	-€ 5,519,991	-€ 11,039,982	-€ 16,559,973	-€ 22,079,964
811	0.18	€ 289,596	€ 579,191	€ 868,787	##########	-€ 7,002,337	-€ 14,004,674	-€ 21,007,011	-€ 28,009,348
812	0.14	€ 218,781	€ 437,562	€ 656,342	€ 875,123	-€ 5,290,054	-€ 10,580,108	-€ 15,870,161	-€ 21,160,215

813	0.21	€ 323,679	€ 647,359	€ 971,038	##########	-€ 7,826,474	-€ 15,652,947	-€ 23,479,421	-€ 31,305,894
814	0.17	€ 261,522	€ 523,045	€ 784,567	##########	-€ 6,323,533	-€ 12,647,065	-€ 18,970,598	-€ 25,294,131
815	0.22	€ 354,172	€ 708,345	##########	##########	-€ 8,563,781	-€ 17,127,562	-€ 25,691,342	-€ 34,255,123
816	0.18	€ 276,734	€ 553,468	€ 830,201	##########	-€ 6,691,342	-€ 13,382,683	-€ 20,074,025	-€ 26,765,366
817	0.16	€ 245,730	€ 491,461	€ 737,191	€ 982,922	-€ 5,941,690	-€ 11,883,379	-€ 17,825,069	-€ 23,766,758
818	0.20	€ 317,987	€ 635,974	€ 953,960	##########	-€ 7,688,826	-€ 15,377,652	-€ 23,066,477	-€ 30,755,303
819	0.21	€ 336,567	€ 673,135	##########	##########	-€ 8,138,101	-€ 16,276,201	-€ 24,414,302	-€ 32,552,403
820	0.18	€ 290,324	€ 580,649	€ 870,973	##########	-€ 7,019,955	-€ 14,039,910	-€ 21,059,864	-€ 28,079,819
821	0.16	€ 254,142	€ 508,284	€ 762,426	##########	-€ 6,145,079	-€ 12,290,159	-€ 18,435,238	-€ 24,580,317
822	0.12	€ 191,704	€ 383,408	€ 575,111	€ 766,815	-€ 4,635,341	-€ 9,270,682	-€ 13,906,023	-€ 18,541,364
823	0.17	€ 271,844	€ 543,689	€ 815,533	##########	-€ 6,573,114	-€ 13,146,228	-€ 19,719,341	-€ 26,292,455
824	0.22	€ 351,726	€ 703,453	##########	##########	-€ 8,504,637	-€ 17,009,274	-€ 25,513,911	-€ 34,018,548
825	0.15	€ 242,944	€ 485,887	€ 728,831	€ 971,775	-€ 5,874,306	-€ 11,748,612	-€ 17,622,919	-€ 23,497,225
826	0.20	€ 321,660	€ 643,320	€ 964,979	##########	-€ 7,777,638	-€ 15,555,276	-€ 23,332,914	-€ 31,110,552
827	0.20	€ 318,632	€ 637,265	€ 955,897	##########	-€ 7,704,435	-€ 15,408,870	-€ 23,113,305	-€ 30,817,741
828	0.22	€ 348,556	€ 697,111	##########	##########	-€ 8,427,973	-€ 16,855,946	-€ 25,283,919	-€ 33,711,892
829	0.14	€ 216,013	€ 432,025	€ 648,038	€ 864,051	-€ 5,223,123	-€ 10,446,246	-€ 15,669,369	-€ 20,892,492
830	0.12	€ 189,800	€ 379,600	€ 569,400	€ 759,200	-€ 4,589,308	-€ 9,178,615	-€ 13,767,923	-€ 18,357,230
831	0.16	€ 253,184	€ 506,368	€ 759,552	##########	-€ 6,121,918	-€ 12,243,836	-€ 18,365,753	-€ 24,487,671
832	0.18	€ 280,206	€ 560,412	€ 840,618	##########	-€ 6,775,301	-€ 13,550,602	-€ 20,325,904	-€ 27,101,205
833	0.27	€ 432,439	€ 864,878	##########	##########	-€ 10,456,241	-€ 20,912,482	-€ 31,368,723	-€ 41,824,964
834	0.28	€ 438,314	€ 876,628	##########	##########	-€ 10,598,302	-€ 21,196,603	-€ 31,794,905	-€ 42,393,207
835	0.18	€ 278,495	€ 556,990	€ 835,484	##########	-€ 6,733,921	-€ 13,467,842	-€ 20,201,762	-€ 26,935,683
836	0.13	€ 209,893	€ 419,785	€ 629,678	€ 839,570	-€ 5,075,141	-€ 10,150,283	-€ 15,225,424	-€ 20,300,565
837	0.13	€ 204,768	€ 409,536	€ 614,304	€ 819,072	-€ 4,951,232	-€ 9,902,463	-€ 14,853,695	-€ 19,804,926
838	0.22	€ 348,046	€ 696,092	##########	##########	-€ 8,415,651	-€ 16,831,303	-€ 25,246,954	-€ 33,662,606
839	0.21	€ 327,270	€ 654,540	€ 981,810	##########	-€ 7,913,290	-€ 15,826,580	-€ 23,739,871	-€ 31,653,161
840	0.22	€ 342,936	€ 685,872	##########	##########	-€ 8,292,088	-€ 16,584,175	-€ 24,876,263	-€ 33,168,351
841	0.23	€ 356,742	€ 713,484	##########	##########	-€ 8,625,911	-€ 17,251,822	-€ 25,877,734	-€ 34,503,645
842	0.17	€ 272,957	€ 545,913	€ 818,870	##########	-€ 6,600,010	-€ 13,200,020	-€ 19,800,030	-€ 26,400,040
843	0.12	€ 182,915	€ 365,830	€ 548,745	€ 731,660	-€ 4,422,832	-€ 8,845,664	-€ 13,268,496	-€ 17,691,328
844	0.24	€ 378,946	€ 757,893	##########	##########	-€ 9,162,809	-€ 18,325,619	-€ 27,488,428	-€ 36,651,238
845	0.18	€ 290,687	€ 581,374	€ 872,060	##########	-€ 7,028,721	-€ 14,057,442	-€ 21,086,162	-€ 28,114,883
846	0.19	€ 297,765	€ 595,530	€ 893,295	##########	-€ 7,199,869	-€ 14,399,738	-€ 21,599,607	-€ 28,799,476
847	0.15	€ 241,001	€ 482,002	€ 723,003	€ 964,004	-€ 5,827,332	-€ 11,654,663	-€ 17,481,995	-€ 23,309,327
848	0.20	€ 307,347	€ 614,694	€ 922,041	##########	-€ 7,431,556	-€ 14,863,112	-€ 22,294,668	-€ 29,726,224
849	0.10	€ 162,241	€ 324,481	€ 486,722	€ 648,963	-€ 3,922,932	-€ 7,845,863	-€ 11,768,795	-€ 15,691,726
850	0.12	€ 184,558	€ 369,116	€ 553,674	€ 738,232	-€ 4,462,556	-€ 8,925,113	-€ 13,387,669	-€ 17,850,225
851	0.21	€ 330,458	€ 660,916	€ 991,373	##########	-€ 7,990,371	-€ 15,980,743	-€ 23,971,114	-€ 31,961,485
852	0.18	€ 290,502	€ 581,004	€ 871,506	##########	-€ 7,024,253	-€ 14,048,507	-€ 21,072,760	-€ 28,097,013
853	0.17	€ 271,340	€ 542,680	€ 814,019	##########	-€ 6,560,916	-€ 13,121,832	-€ 19,682,748	-€ 26,243,665
854	0.25	€ 390,745	€ 781,490	##########	##########	-€ 9,448,098	-€ 18,896,195	-€ 28,344,293	-€ 37,792,390
855	0.18	€ 282,852	€ 565,705	€ 848,557	##########	-€ 6,839,284	-€ 13,678,568	-€ 20,517,851	-€ 27,357,135
856	0.21	€ 327,145	€ 654,291	€ 981,436	##########	-€ 7,910,281	-€ 15,820,562	-€ 23,730,842	-€ 31,641,123
857	0.12	€ 182,793	€ 365,587	€ 548,380	€ 731,173	-€ 4,419,887	-€ 8,839,774	-€ 13,259,661	-€ 17,679,548
858	0.27	€ 431,851	€ 863,701	##########	##########	-€ 10,442,020	-€ 20,884,041	-€ 31,326,061	-€ 41,768,081
859	0.16	€ 259,377	€ 518,755	€ 778,132	##########	-€ 6,271,666	-€ 12,543,332	-€ 18,814,998	-€ 25,086,664
860	0.23	€ 361,779	€ 723,557	##########	##########	-€ 8,747,699	-€ 17,495,398	-€ 26,243,097	-€ 34,990,797
861	0.23	€ 361,553	€ 723,106	##########	##########	-€ 8,742,250	-€ 17,484,499	-€ 26,226,749	-€ 34,968,998
862	0.20	€ 320,114	€ 640,227	€ 960,341	##########	-€ 7,740,255	-€ 15,480,511	-€ 23,220,766	-€ 30,961,021
863	0.27	€ 419,214	€ 838,427	##########	##########	-€ 10,136,463	-€ 20,272,927	-€ 30,409,390	-€ 40,545,853
864	0.15	€ 236,457	€ 472,914	€ 709,372	€ 945,829	-€ 5,717,465	-€ 11,434,930	-€ 17,152,394	-€ 22,869,859
865	0.17	€ 261,564	€ 523,127	€ 784,691	##########	-€ 6,324,528	-€ 12,649,056	-€ 18,973,584	-€ 25,298,112
866	0.16	€ 257,474	€ 514,948	€ 772,423	##########	-€ 6,225,650	-€ 12,451,300	-€ 18,676,950	-€ 24,902,600
867	0.19	€ 298,627	€ 597,254	€ 895,881	##########	-€ 7,220,711	-€ 14,441,421	-€ 21,662,132	-€ 28,882,843
868	0.19	€ 296,609	€ 593,217	€ 889,826	##########	-€ 7,171,911	-€ 14,343,822	-€ 21,515,733	-€ 28,687,644
869	0.13	€ 208,100	€ 416,201	€ 624,301	€ 832,401	-€ 5,031,805	-€ 10,063,610	-€ 15,095,415	-€ 20,127,220
870	0.25	€ 391,113	€ 782,225	##########	##########	-€ 9,456,986	-€ 18,913,971	-€ 28,370,957	-€ 37,827,942
871	0.22	€ 348,806	€ 697,612	##########	##########	-€ 8,434,031	-€ 16,868,061	-€ 25,302,092	-€ 33,736,122
872	0.17	€ 262,012	€ 524,024	€ 786,037	##########	-€ 6,335,378	-€ 12,670,756	-€ 19,006,134	-€ 25,341,511
873	0.13	€ 206,804	€ 413,607	€ 620,411	€ 827,214	-€ 5,000,449	-€ 10,000,899	-€ 15,001,348	-€ 20,001,798
874	0.23	€ 364,800	€ 729,599	##########	##########	-€ 8,820,745	-€ 17,641,490	-€ 26,462,234	-€ 35,282,979
875	0.17	€ 265,956	€ 531,913	€ 797,869	##########	-€ 6,430,743	-€ 12,861,486	-€ 19,292,230	-€ 25,722,973
876	0.20	€ 317,696	€ 635,392	€ 953,089	##########	-€ 7,681,801	-€ 15,363,602	-€ 23,045,403	-€ 30,727,204
877	0.21	€ 326,186	€ 652,372	€ 978,558	##########	-€ 7,887,085	-€ 15,774,169	-€ 23,661,254	-€ 31,548,338
878	0.21	€ 337,634	€ 675,267	##########	##########	-€ 8,163,883	-€ 16,327,765	-€ 24,491,648	-€ 32,655,531
879	0.25	€ 393,628	€ 787,257	##########	##########	-€ 9,517,817	-€ 19,035,634	-€ 28,553,451	-€ 38,071,268
880	0.19	€ 293,420	€ 586,839	€ 880,259	##########	-€ 7,094,801	-€ 14,189,603	-€ 21,284,404	-€ 28,379,206
881	0.20	€ 312,849	€ 625,699	€ 938,548	##########	-€ 7,564,603	-€ 15,129,206	-€ 22,693,810	-€ 30,258,413
882	0.12	€ 183,095	€ 366,190	€ 549,285	€ 732,380	-€ 4,427,181	-€ 8,854,362	-€ 13,281,543	-€ 17,708,724
883	0.22	€ 343,335	€ 686,669	##########	##########	-€ 8,301,732	-€ 16,603,465	-€ 24,905,197	-€ 33,206,929
884	0.21	€ 336,140	€ 672,281	##########	##########	-€ 8,127,774	-€ 16,255,548	-€ 24,383,322	-€ 32,511,097
885	0.16	€ 254,473	€ 508,946	€ 763,419	##########	-€ 6,153,086	-€ 12,306,171	-€ 18,459,257	-€ 24,612,342
886	0.17	€ 261,968	€ 523,936	€ 785,904	##########	-€ 6,334,311	-€ 12,668,622	-€ 19,002,933	-€ 25,337,244
887	0.15	€ 243,738	€ 487,476	€ 731,214	€ 974,952	-€ 5,893,514	-€ 11,787,027	-€ 17,680,541	-€ 23,574,055
888	0.20	€ 320,249	€ 640,498	€ 960,746	##########	-€ 7,743,520	-€ 15,487,040	-€ 23,230,560	-€ 30,974,080
889	0.24	€ 377,869	€ 755,738	##########	##########	-€ 9,136,766	-€ 18,273,531	-€ 27,410,297	-€ 36,547,063
890	0.21	€ 325,426	€ 650,853	€ 976,279	##########	-€ 7,868,711	-€ 15,737,423	-€ 23,606,134	-€ 31,474,846
891	0.11	€ 173,654	€ 347,308	€ 520,963	€ 694,617	-€ 4,198,907	-€ 8,397,814	-€ 12,596,721	-€ 16,795,628
892	0.13	€ 211,541	€ 423,083	€ 634,624	€ 846,165	-€ 5,115,007	-€ 10,230,014	-€ 15,345,021	-€ 20,460,028
893	0.25	€ 386,464	€ 772,928	##########	##########	-€ 9,344,582	-€ 18,689,164	-€ 28,033,746	-€ 37,378,328
894	0.15	€ 231,541	€ 463,082	€ 694,624	€ 926,165	-€ 5,598,598	-€ 11,197,197	-€ 16,795,795	-€ 22,394,393
895	0.18	€ 282,333	€ 564,667	€ 847,000	##########	-€ 6,826,734	-€ 13,653,468	-€ 20,480,203	-€ 27,306,937
896	0.21	€ 336,848	€ 673,696	##########	##########	-€ 8,144,879	-€ 16,289,757	-€ 24,434,636	-€ 32,579,514
897	0.13	€ 197,403	€ 394,806	€ 592,208	€ 789,611	-€ 4,773,140	-€ 9,546,281	-€ 14,319,421	-€ 19,092,562
898	0.17	€ 265,004	€ 530,008	€ 795,012	##########	-€ 6,407,718	-€ 12,815,436	-€ 19,223,154	-€ 25,630,872
899	0.15	€ 241,135	€ 482,271	€ 723,406	€ 964,542	-€ 5,830,583	-€ 11,661,166	-€ 17,491,748	-€ 23,322,331
900	0.20	€ 315,034	€ 630,067	€ 945,101	##########	-€ 7,617,421	-€ 15,234,843	-€ 22,852,264	-€ 30,469,685
901	0.16	€ 244,526	€ 489,052	€ 733,578	€ 978,104	-€ 5,912,566	-€ 11,825,132	-€ 17,737,697	-€ 23,650,263
902	0.24	€ 374,111	€ 748,221	##########	##########	-€ 9,045,885	-€ 18,091,771	-€ 27,137,656	-€ 36,183,541
903	0.21	€ 326,421	€ 652,842	€ 979,263	##########	-€ 7,892,766	-€ 15,785,533	-€ 23,678,299	-€ 31,571,065
904	0.24	€ 371,545	€ 743,090	##########	##########	-€ 8,983,851	-€ 17,967,701	-€ 26,951,552	-€ 35,935,402
905	0.19	€ 292,192	€ 584,384	€ 876,576	##########	-€ 7,065,116	-€ 14,130,233	-€ 21,195,349	-€ 28,260,466
906	0.19	€ 299,734	€ 599,468	€ 899,201	##########	-€ 7,247,474	-€ 14,494,949	-€ 21,742,423	-€ 28,989,897
907	0.19	€ 302,375	€ 604,750	€ 907,125	##########	-€ 7,311,335	-€ 14,622,669	-€ 21,934,004	-€ 29,245,338
908	0.21	€ 324,348	€ 648,695	€ 973,043	##########	-€ 7,842,631	-€ 15,685,262	-€ 23,527,893	-€ 31,370,524
909	0.23	€ 361,968	€ 723,936	##########	##########	-€ 8,752,284	-€ 17,504,568	-€ 26,256,851	-€ 35,009,135
910	0.20	€ 322,079	€ 644,159	€ 966,238	##########	-€ 7,787,785	-€ 15,575,570	-€ 23,363,355	-€ 31,151,140
911	0.15	€ 241,365	€ 482,729	€ 724,094	€ 965,458	-€ 5,836,125	-€ 11,672,250	-€ 17,508,374	-€ 23,344,499
912	0.13	€ 205,352	€ 410,703	€ 616,055	€ 821,406	-€ 4,965,341	-€ 9,930,682	-€ 14,896,022	-€ 19,861,363
913	0.28	€ 434,331	€ 868,662	##########	##########	-€ 10,501,994	-€ 21,003,987	-€ 31,505,981	-€ 42,007,974
914	0.20	€ 312,374	€ 624,747	€ 937,121	##########	-€ 7,553,099	-€ 15,106,197	-€ 22,659,296	-€ 30,212,394

915	0.22	€ 345,188	€ 690,375	##########	##########	-€ 8,346,536	-€ 16,693,073	-€ 25,039,609	-€ 33,386,145
916	0.23	€ 367,083	€ 734,165	##########	##########	-€ 8,875,948	-€ 17,751,896	-€ 26,627,845	-€ 35,503,793
917	0.13	€ 199,454	€ 398,908	€ 598,362	€ 797,815	-€ 4,822,735	-€ 9,645,470	-€ 14,468,205	-€ 19,290,939
918	0.17	€ 264,786	€ 529,573	€ 794,359	##########	-€ 6,402,454	-€ 12,804,909	-€ 19,207,363	-€ 25,609,817
919	0.24	€ 374,171	€ 748,343	##########	##########	-€ 9,047,351	-€ 18,094,703	-€ 27,142,054	-€ 36,189,406
920	0.19	€ 302,579	€ 605,158	€ 907,738	##########	-€ 7,316,275	-€ 14,632,551	-€ 21,948,826	-€ 29,265,101
921	0.15	€ 243,365	€ 486,729	€ 730,094	€ 973,458	-€ 5,884,484	-€ 11,768,968	-€ 17,653,453	-€ 23,537,937
922	0.24	€ 379,786	€ 759,573	##########	##########	-€ 9,183,120	-€ 18,366,239	-€ 27,549,359	-€ 36,732,479
923	0.20	€ 320,525	€ 641,050	€ 961,575	##########	-€ 7,750,199	-€ 15,500,397	-€ 23,250,596	-€ 31,000,794
924	0.19	€ 298,963	€ 597,926	€ 896,890	##########	-€ 7,228,841	-€ 14,457,683	-€ 21,686,524	-€ 28,915,365
925	0.23	€ 354,617	€ 709,233	##########	##########	-€ 8,574,526	-€ 17,149,052	-€ 25,723,579	-€ 34,298,105
926	0.27	€ 424,566	€ 849,132	##########	##########	-€ 10,265,882	-€ 20,531,765	-€ 30,797,647	-€ 41,063,529
927	0.20	€ 317,549	€ 635,097	€ 952,646	##########	-€ 7,678,234	-€ 15,356,468	-€ 23,034,703	-€ 30,712,937
928	0.20	€ 319,712	€ 639,425	€ 959,137	##########	-€ 7,730,552	-€ 15,461,104	-€ 23,191,657	-€ 30,922,209
929	0.19	€ 303,787	€ 607,574	€ 911,362	##########	-€ 7,345,485	-€ 14,690,970	-€ 22,036,456	-€ 29,381,941
930	0.18	€ 276,953	€ 553,907	€ 830,860	##########	-€ 6,696,652	-€ 13,393,305	-€ 20,089,957	-€ 26,786,609
931	0.16	€ 258,997	€ 517,993	€ 776,990	##########	-€ 6,262,462	-€ 12,524,924	-€ 18,787,386	-€ 25,049,848
932	0.26	€ 403,158	€ 806,317	##########	##########	-€ 9,748,251	-€ 19,496,503	-€ 29,244,754	-€ 38,993,006
933	0.20	€ 307,194	€ 614,388	€ 921,583	##########	-€ 7,427,865	-€ 14,855,729	-€ 22,283,594	-€ 29,711,458
934	0.24	€ 374,907	€ 749,814	##########	##########	-€ 9,065,135	-€ 18,130,271	-€ 27,195,406	-€ 36,260,542
935	0.09	€ 134,788	€ 269,576	€ 404,365	€ 539,153	-€ 3,259,139	-€ 6,518,278	-€ 9,777,416	-€ 13,036,555
936	0.24	€ 371,899	€ 743,798	##########	##########	-€ 8,992,403	-€ 17,984,807	-€ 26,977,210	-€ 35,969,613
937	0.19	€ 295,699	€ 591,399	€ 887,098	##########	-€ 7,149,924	-€ 14,299,848	-€ 21,449,773	-€ 28,599,697
938	0.18	€ 284,741	€ 569,481	€ 854,222	##########	-€ 6,884,944	-€ 13,769,889	-€ 20,654,833	-€ 27,539,778
939	0.16	€ 257,524	€ 515,047	€ 772,571	##########	-€ 6,226,846	-€ 12,453,693	-€ 18,680,539	-€ 24,907,386
940	0.17	€ 268,351	€ 536,701	€ 805,052	##########	-€ 6,488,636	-€ 12,977,271	-€ 19,465,907	-€ 25,954,542
941	0.24	€ 379,912	€ 759,824	##########	##########	-€ 9,186,161	-€ 18,372,323	-€ 27,558,484	-€ 36,744,645
942	0.21	€ 330,421	€ 660,843	€ 991,264	##########	-€ 7,989,488	-€ 15,978,976	-€ 23,968,465	-€ 31,957,953
943	0.16	€ 251,652	€ 503,303	€ 754,955	##########	-€ 6,084,860	-€ 12,169,721	-€ 18,254,581	-€ 24,339,442
944	0.15	€ 239,273	€ 478,546	€ 717,818	€ 957,091	-€ 5,785,545	-€ 11,571,089	-€ 17,356,634	-€ 23,142,178
945	0.19	€ 296,259	€ 592,519	€ 888,778	##########	-€ 7,163,462	-€ 14,326,924	-€ 21,490,386	-€ 28,653,848
946	0.21	€ 327,512	€ 655,023	€ 982,535	##########	-€ 7,919,133	-€ 15,838,267	-€ 23,757,400	-€ 31,676,534
947	0.16	€ 254,804	€ 509,608	€ 764,412	##########	-€ 6,161,086	-€ 12,322,172	-€ 18,483,258	-€ 24,644,344
948	0.23	€ 358,374	€ 716,749	##########	##########	-€ 8,665,383	-€ 17,330,767	-€ 25,996,150	-€ 34,661,533
949	0.21	€ 324,697	€ 649,394	€ 974,092	##########	-€ 7,851,081	-€ 15,702,163	-€ 23,553,244	-€ 31,404,325
950	0.18	€ 275,779	€ 551,558	€ 827,337	##########	-€ 6,668,257	-€ 13,336,514	-€ 20,004,770	-€ 26,673,027
951	0.10	€ 159,474	€ 318,948	€ 478,422	€ 637,896	-€ 3,856,034	-€ 7,712,069	-€ 11,568,103	-€ 15,424,137
952	0.19	€ 306,442	€ 612,885	€ 919,327	##########	-€ 7,409,686	-€ 14,819,373	-€ 22,229,059	-€ 29,638,745
953	0.22	€ 341,579	€ 683,158	##########	##########	-€ 8,259,276	-€ 16,518,552	-€ 24,777,828	-€ 33,037,104
954	0.22	€ 350,404	€ 700,809	##########	##########	-€ 8,472,673	-€ 16,945,346	-€ 25,418,019	-€ 33,890,692
955	0.14	€ 214,706	€ 429,411	€ 644,117	€ 858,823	-€ 5,191,521	-€ 10,383,042	-€ 15,574,563	-€ 20,766,084
956	0.18	€ 287,208	€ 574,416	€ 861,623	##########	-€ 6,944,599	-€ 13,889,198	-€ 20,833,798	-€ 27,778,397
957	0.16	€ 245,002	€ 490,003	€ 735,005	€ 980,006	-€ 5,924,065	-€ 11,848,129	-€ 17,772,194	-€ 23,696,258
958	0.29	€ 450,559	€ 901,118	##########	##########	-€ 10,894,380	-€ 21,788,760	-€ 32,683,140	-€ 43,577,520
959	0.22	€ 350,656	€ 701,312	##########	##########	-€ 8,478,754	-€ 16,957,508	-€ 25,436,263	-€ 33,915,017
960	0.27	€ 425,582	€ 851,164	##########	##########	-€ 10,290,451	-€ 20,580,902	-€ 30,871,354	-€ 41,161,805
961	0.23	€ 366,118	€ 732,235	##########	##########	-€ 8,852,618	-€ 17,705,237	-€ 26,557,855	-€ 35,410,473
962	0.17	€ 266,529	€ 533,057	€ 799,586	##########	-€ 6,444,582	-€ 12,889,164	-€ 19,333,746	-€ 25,778,328
963	0.21	€ 337,130	€ 674,260	##########	##########	-€ 8,151,705	-€ 16,303,409	-€ 24,455,114	-€ 32,606,818
964	0.22	€ 341,376	€ 682,752	##########	##########	-€ 8,254,370	-€ 16,508,741	-€ 24,763,111	-€ 33,017,482
965	0.18	€ 284,763	€ 569,527	€ 854,290	##########	-€ 6,885,497	-€ 13,770,993	-€ 20,656,490	-€ 27,541,986
966	0.18	€ 291,239	€ 582,478	€ 873,716	##########	-€ 7,042,068	-€ 14,084,137	-€ 21,126,205	-€ 28,168,274
967	0.24	€ 381,879	€ 763,757	##########	##########	-€ 9,233,714	-€ 18,467,427	-€ 27,701,141	-€ 36,934,854
968	0.17	€ 275,094	€ 550,188	€ 825,281	##########	-€ 6,651,686	-€ 13,303,372	-€ 19,955,057	-€ 26,606,743
969	0.21	€ 330,753	€ 661,505	€ 992,258	##########	-€ 7,997,499	-€ 15,994,998	-€ 23,992,496	-€ 31,989,995
970	0.17	€ 272,001	€ 544,002	€ 816,003	##########	-€ 6,576,903	-€ 13,153,807	-€ 19,730,710	-€ 26,307,613
971	0.18	€ 279,683	€ 559,366	€ 839,049	##########	-€ 6,762,656	-€ 13,525,311	-€ 20,287,967	-€ 27,050,622
972	0.20	€ 307,458	€ 614,916	€ 922,375	##########	-€ 7,434,247	-€ 14,868,495	-€ 22,302,742	-€ 29,736,989
973	0.18	€ 289,238	€ 578,476	€ 867,714	##########	-€ 6,993,689	-€ 13,987,379	-€ 20,981,068	-€ 27,974,757
974	0.18	€ 278,001	€ 556,002	€ 834,003	##########	-€ 6,721,979	-€ 13,443,958	-€ 20,165,937	-€ 26,887,916
975	0.29	€ 454,441	€ 908,882	##########	##########	-€ 10,988,254	-€ 21,976,507	-€ 32,964,761	-€ 43,953,014
976	0.21	€ 326,389	€ 652,778	€ 979,167	##########	-€ 7,891,993	-€ 15,783,985	-€ 23,675,978	-€ 31,567,970
977	0.16	€ 245,769	€ 491,539	€ 737,308	€ 983,078	-€ 5,942,632	-€ 11,885,265	-€ 17,827,897	-€ 23,770,529
978	0.13	€ 210,149	€ 420,298	€ 630,447	€ 840,596	-€ 5,081,343	-€ 10,162,686	-€ 15,244,029	-€ 20,325,372
979	0.20	€ 310,648	€ 621,296	€ 931,944	##########	-€ 7,511,379	-€ 15,022,757	-€ 22,534,136	-€ 30,045,514
980	0.15	€ 239,495	€ 478,990	€ 718,486	€ 957,981	-€ 5,790,922	-€ 11,581,844	-€ 17,372,766	-€ 23,163,688
981	0.23	€ 367,738	€ 735,476	##########	##########	-€ 8,891,800	-€ 17,783,600	-€ 26,675,400	-€ 35,567,199
982	0.18	€ 288,640	€ 577,281	€ 865,921	##########	-€ 6,979,240	-€ 13,958,480	-€ 20,937,720	-€ 27,916,961
983	0.19	€ 304,196	€ 608,393	€ 912,589	##########	-€ 7,355,377	-€ 14,710,755	-€ 22,066,132	-€ 29,421,509
984	0.23	€ 362,568	€ 725,136	##########	##########	-€ 8,766,790	-€ 17,533,581	-€ 26,300,371	-€ 35,067,161
985	0.19	€ 299,852	€ 599,703	€ 899,555	##########	-€ 7,250,321	-€ 14,500,642	-€ 21,750,964	-€ 29,001,285
986	0.24	€ 385,579	€ 771,157	##########	##########	-€ 9,323,178	-€ 18,646,357	-€ 27,969,535	-€ 37,292,714
987	0.22	€ 353,055	€ 706,111	##########	##########	-€ 8,536,773	-€ 17,073,546	-€ 25,610,318	-€ 34,147,091
988	0.14	€ 218,470	€ 436,941	€ 655,411	€ 873,881	-€ 5,282,548	-€ 10,565,096	-€ 15,847,644	-€ 21,130,192
989	0.19	€ 298,741	€ 597,483	€ 896,224	##########	-€ 7,223,479	-€ 14,446,958	-€ 21,670,437	-€ 28,893,916
990	0.15	€ 238,199	€ 476,398	€ 714,597	€ 952,796	-€ 5,759,579	-€ 11,519,158	-€ 17,278,738	-€ 23,038,317
991	0.20	€ 311,510	€ 623,020	€ 934,530	##########	-€ 7,532,217	-€ 15,064,435	-€ 22,596,652	-€ 30,128,869
992	0.18	€ 282,602	€ 565,204	€ 847,806	##########	-€ 6,833,229	-€ 13,666,459	-€ 20,499,688	-€ 27,332,917
993	0.18	€ 286,328	€ 572,656	€ 858,984	##########	-€ 6,923,327	-€ 13,846,655	-€ 20,769,982	-€ 27,693,310
994	0.13	€ 209,592	€ 419,185	€ 628,777	€ 838,370	-€ 5,067,884	-€ 10,135,768	-€ 15,203,652	-€ 20,271,535
995	0.18	€ 289,942	€ 579,884	€ 869,826	##########	-€ 7,010,711	-€ 14,021,423	-€ 21,032,134	-€ 28,042,845
996	0.21	€ 336,557	€ 673,115	##########	##########	-€ 8,137,859	-€ 16,275,717	-€ 24,413,576	-€ 32,551,434
997	0.14	€ 225,969	€ 451,938	€ 677,907	€ 903,876	-€ 5,463,866	-€ 10,927,732	-€ 16,391,598	-€ 21,855,464
998	0.19	€ 305,732	€ 611,464	€ 917,196	##########	-€ 7,392,510	-€ 14,785,019	-€ 22,177,529	-€ 29,570,039
999	0.15	€ 237,711	€ 475,421	€ 713,132	€ 950,842	-€ 5,747,771	-€ 11,495,543	-€ 17,243,314	-€ 22,991,086
1000	0.19	€ 303,442	€ 606,884	€ 910,326	##########	-€ 7,337,134	-€ 14,674,269	-€ 22,011,403	-€ 29,348,537
1001	0.15	€ 235,611	€ 471,222	€ 706,834	€ 942,445	-€ 5,697,009	-€ 11,394,018	-€ 17,091,027	-€ 22,788,036
1002	0.16	€ 257,430	€ 514,860	€ 772,289	##########	-€ 6,224,576	-€ 12,449,152	-€ 18,673,728	-€ 24,898,304
1003	0.23	€ 355,934	€ 711,868	##########	##########	-€ 8,606,375	-€ 17,212,749	-€ 25,819,124	-€ 34,425,498
1004	0.21	€ 326,827	€ 653,654	€ 980,481	##########	-€ 7,902,582	-€ 15,805,164	-€ 23,707,745	-€ 31,610,327
1005	0.20	€ 318,827	€ 637,654	€ 956,482	##########	-€ 7,709,147	-€ 15,418,295	-€ 23,127,442	-€ 30,836,589
1006	0.13	€ 196,894	€ 393,789	€ 590,683	€ 787,578	-€ 4,760,849	-€ 9,521,698	-€ 14,282,547	-€ 19,043,395
1007	0.24	€ 371,518	€ 743,035	##########	##########	-€ 8,983,187	-€ 17,966,373	-€ 26,949,560	-€ 35,932,746
1008	0.22	€ 352,399	€ 704,799	##########	##########	-€ 8,520,914	-€ 17,041,829	-€ 25,562,743	-€ 34,083,657
1009	0.24	€ 374,657	€ 749,314	##########	##########	-€ 9,059,089	-€ 18,118,179	-€ 27,177,268	-€ 36,236,357
1010	0.17	€ 265,963	€ 531,926	€ 797,888	##########	-€ 6,430,901	-€ 12,861,802	-€ 19,292,703	-€ 25,723,604
1011	0.20	€ 320,846	€ 641,691	€ 962,537	##########	-€ 7,757,953	-€ 15,515,907	-€ 23,273,860	-€ 31,031,814
1012	0.17	€ 263,881	€ 527,763	€ 791,644	##########	-€ 6,380,574	-€ 12,761,147	-€ 19,141,721	-€ 25,522,295
1013	0.12	€ 195,220	€ 390,440	€ 585,660	€ 780,880	-€ 4,720,363	-€ 9,440,726	-€ 14,161,089	-€ 18,881,453
1014	0.23	€ 366,201	€ 732,403	##########	##########	-€ 8,854,642	-€ 17,709,283	-€ 26,563,925	-€ 35,418,566
1015	0.14	€ 217,821	€ 435,643	€ 653,464	€ 871,286	-€ 5,266,857	-€ 10,533,713	-€ 15,800,570	-€ 21,067,426
1016	0.18	€ 284,850	€ 569,700	€ 854,550	##########	-€ 6,887,585	-€ 13,775,171	-€ 20,662,756	-€ 27,550,342

1017	0.24	€ 384,689	€ 769,379	##########	##########	-€ 9,301,676	-€ 18,603,351	-€ 27,905,027	-€ 37,206,702
1018	0.22	€ 345,423	€ 690,847	##########	##########	-€ 8,352,233	-€ 16,704,466	-€ 25,056,699	-€ 33,408,932
1019	0.16	€ 254,859	€ 509,717	€ 764,576	##########	-€ 6,162,403	-€ 12,324,807	-€ 18,487,210	-€ 24,649,613
1020	0.21	€ 332,889	€ 665,779	€ 998,668	##########	-€ 8,049,168	-€ 16,098,335	-€ 24,147,503	-€ 32,196,670
1021	0.23	€ 364,343	€ 728,686	##########	##########	-€ 8,809,704	-€ 17,619,408	-€ 26,429,112	-€ 35,238,816
1022	0.17	€ 262,226	€ 524,452	€ 786,678	##########	-€ 6,340,548	-€ 12,681,095	-€ 19,021,643	-€ 25,362,191
1023	0.18	€ 286,294	€ 572,588	€ 858,881	##########	-€ 6,922,499	-€ 13,844,999	-€ 20,767,498	-€ 27,689,997
1024	0.18	€ 280,426	€ 560,852	€ 841,278	##########	-€ 6,780,621	-€ 13,561,242	-€ 20,341,863	-€ 27,122,483
1025	0.18	€ 285,062	€ 570,124	€ 855,186	##########	-€ 6,892,711	-€ 13,785,421	-€ 20,678,132	-€ 27,570,842
1026	0.16	€ 249,502	€ 499,005	€ 748,507	€ 998,009	-€ 6,032,893	-€ 12,065,785	-€ 18,098,678	-€ 24,131,571
1027	0.17	€ 274,153	€ 548,306	€ 822,459	##########	-€ 6,628,939	-€ 13,257,879	-€ 19,886,818	-€ 26,515,758
1028	0.22	€ 343,240	€ 686,480	##########	##########	-€ 8,299,441	-€ 16,598,881	-€ 24,898,322	-€ 33,197,762
1029	0.21	€ 338,177	€ 676,355	##########	##########	-€ 8,177,026	-€ 16,354,053	-€ 24,531,079	-€ 32,708,106
1030	0.17	€ 265,235	€ 530,470	€ 795,705	##########	-€ 6,413,303	-€ 12,826,606	-€ 19,239,910	-€ 25,653,213
1031	0.17	€ 272,651	€ 545,302	€ 817,953	##########	-€ 6,592,620	-€ 13,185,240	-€ 19,777,860	-€ 26,370,480
1032	0.16	€ 247,000	€ 493,999	€ 740,999	€ 987,999	-€ 5,972,380	-€ 11,944,760	-€ 17,917,140	-€ 23,889,520
1033	0.17	€ 262,656	€ 525,312	€ 787,968	##########	-€ 6,350,944	-€ 12,701,888	-€ 19,052,832	-€ 25,403,776
1034	0.19	€ 305,364	€ 610,729	€ 916,093	##########	-€ 7,383,617	-€ 14,767,234	-€ 22,150,851	-€ 29,534,468
1035	0.22	€ 348,778	€ 697,556	##########	##########	-€ 8,433,354	-€ 16,866,708	-€ 25,300,061	-€ 33,733,415
1036	0.11	€ 172,637	€ 345,275	€ 517,912	€ 690,550	-€ 4,174,322	-€ 8,348,644	-€ 12,522,966	-€ 16,697,288
1037	0.15	€ 237,700	€ 475,399	€ 713,099	€ 950,799	-€ 5,747,509	-€ 11,495,018	-€ 17,242,527	-€ 22,990,035
1038	0.22	€ 350,983	€ 701,967	##########	##########	-€ 8,486,674	-€ 16,973,347	-€ 25,460,021	-€ 33,946,694
1039	0.13	€ 208,309	€ 416,619	€ 624,928	€ 833,237	-€ 5,036,857	-€ 10,073,713	-€ 15,110,570	-€ 20,147,426
1040	0.17	€ 273,311	€ 546,622	€ 819,934	##########	-€ 6,608,583	-€ 13,217,167	-€ 19,825,750	-€ 26,434,334
1041	0.20	€ 319,844	€ 639,689	€ 959,533	##########	-€ 7,733,740	-€ 15,467,480	-€ 23,201,220	-€ 30,934,960
1042	0.19	€ 303,282	€ 606,565	€ 909,847	##########	-€ 7,333,278	-€ 14,666,556	-€ 21,999,834	-€ 29,333,112
1043	0.18	€ 290,131	€ 580,261	€ 870,392	##########	-€ 7,015,273	-€ 14,030,545	-€ 21,045,818	-€ 28,061,091
1044	0.23	€ 366,075	€ 732,151	##########	##########	-€ 8,851,596	-€ 17,703,192	-€ 26,554,788	-€ 35,406,384
1045	0.22	€ 347,869	€ 695,738	##########	##########	-€ 8,411,373	-€ 16,822,746	-€ 25,234,119	-€ 33,645,492
1046	0.21	€ 329,492	€ 658,985	€ 988,477	##########	-€ 7,967,027	-€ 15,934,054	-€ 23,901,081	-€ 31,868,107
1047	0.19	€ 293,696	€ 587,393	€ 881,089	##########	-€ 7,101,494	-€ 14,202,987	-€ 21,304,481	-€ 28,405,975
1048	0.14	€ 213,131	€ 426,263	€ 639,394	€ 852,525	-€ 5,153,453	-€ 10,306,905	-€ 15,460,358	-€ 20,613,810
1049	0.25	€ 401,439	€ 802,878	##########	##########	-€ 9,706,671	-€ 19,413,342	-€ 29,120,012	-€ 38,826,683
1050	0.22	€ 341,167	€ 682,334	##########	##########	-€ 8,249,321	-€ 16,498,641	-€ 24,747,962	-€ 32,997,283
1051	0.20	€ 311,486	€ 622,972	€ 934,458	##########	-€ 7,531,642	-€ 15,063,283	-€ 22,594,925	-€ 30,126,567
1052	0.18	€ 280,998	€ 561,996	€ 842,994	##########	-€ 6,794,449	-€ 13,588,897	-€ 20,383,346	-€ 27,177,794
1053	0.23	€ 359,264	€ 718,528	##########	##########	-€ 8,686,896	-€ 17,373,792	-€ 26,060,687	-€ 34,747,583
1054	0.18	€ 282,816	€ 565,633	€ 848,449	##########	-€ 6,838,417	-€ 13,676,834	-€ 20,515,251	-€ 27,353,668
1055	0.19	€ 293,533	€ 587,067	€ 880,600	##########	-€ 7,097,551	-€ 14,195,101	-€ 21,292,652	-€ 28,390,203
1056	0.17	€ 275,013	€ 550,027	€ 825,040	##########	-€ 6,649,740	-€ 13,299,479	-€ 19,949,219	-€ 26,598,959
1057	0.19	€ 303,281	€ 606,562	€ 909,843	##########	-€ 7,333,243	-€ 14,666,486	-€ 21,999,729	-€ 29,332,972
1058	0.16	€ 247,201	€ 494,401	€ 741,602	€ 988,802	-€ 5,977,235	-€ 11,954,470	-€ 17,931,705	-€ 23,908,940
1059	0.23	€ 361,363	€ 722,726	##########	##########	-€ 8,737,647	-€ 17,475,295	-€ 26,212,942	-€ 34,950,589
1060	0.19	€ 299,994	€ 599,988	€ 899,982	##########	-€ 7,253,764	-€ 14,507,528	-€ 21,761,292	-€ 29,015,056
1061	0.20	€ 318,139	€ 636,279	€ 954,418	##########	-€ 7,692,517	-€ 15,385,035	-€ 23,077,552	-€ 30,770,069
1062	0.14	€ 225,831	€ 451,662	€ 677,494	€ 903,325	-€ 5,460,531	-€ 10,921,062	-€ 16,381,593	-€ 21,842,124
1063	0.15	€ 231,770	€ 463,541	€ 695,311	€ 927,081	-€ 5,604,137	-€ 11,208,274	-€ 16,812,411	-€ 22,416,549
1064	0.15	€ 242,479	€ 484,959	€ 727,438	€ 969,918	-€ 5,863,081	-€ 11,726,163	-€ 17,589,244	-€ 23,452,326
1065	0.27	€ 425,314	€ 850,628	##########	##########	-€ 10,283,965	-€ 20,567,929	-€ 30,851,894	-€ 41,135,859
1066	0.21	€ 329,929	€ 659,857	€ 989,786	##########	-€ 7,977,574	-€ 15,955,147	-€ 23,932,721	-€ 31,910,295
1067	0.18	€ 282,572	€ 565,143	€ 847,715	##########	-€ 6,832,497	-€ 13,664,994	-€ 20,497,491	-€ 27,329,988
1068	0.22	€ 349,367	€ 698,733	##########	##########	-€ 8,447,583	-€ 16,895,166	-€ 25,342,749	-€ 33,790,332
1069	0.20	€ 309,176	€ 618,352	€ 927,529	##########	-€ 7,475,788	-€ 14,951,576	-€ 22,427,365	-€ 29,903,153
1070	0.24	€ 373,062	€ 746,124	##########	##########	-€ 9,020,532	-€ 18,041,063	-€ 27,061,595	-€ 36,082,127
1071	0.20	€ 321,724	€ 643,447	€ 965,171	##########	-€ 7,779,183	-€ 15,558,366	-€ 23,337,549	-€ 31,116,732
1072	0.21	€ 333,722	€ 667,443	##########	##########	-€ 8,069,288	-€ 16,138,576	-€ 24,207,864	-€ 32,277,152
1073	0.21	€ 333,031	€ 666,062	€ 999,093	##########	-€ 8,052,592	-€ 16,105,184	-€ 24,157,776	-€ 32,210,368
1074	0.17	€ 262,298	€ 524,597	€ 786,895	##########	-€ 6,342,295	-€ 12,684,590	-€ 19,026,884	-€ 25,369,179
1075	0.22	€ 346,716	€ 693,433	##########	##########	-€ 8,383,498	-€ 16,766,996	-€ 25,150,494	-€ 33,533,991
1076	0.12	€ 195,933	€ 391,865	€ 587,798	€ 783,731	-€ 4,737,594	-€ 9,475,188	-€ 14,212,782	-€ 18,950,376
1077	0.11	€ 179,605	€ 359,210	€ 538,815	€ 718,420	-€ 4,342,797	-€ 8,685,594	-€ 13,028,391	-€ 17,371,188
1078	0.15	€ 238,658	€ 477,315	€ 715,973	€ 954,631	-€ 5,770,672	-€ 11,541,343	-€ 17,312,015	-€ 23,082,686
1079	0.17	€ 262,191	€ 524,382	€ 786,573	##########	-€ 6,339,702	-€ 12,679,403	-€ 19,019,105	-€ 25,358,807
1080	0.26	€ 404,511	€ 809,021	##########	##########	-€ 9,780,945	-€ 19,561,890	-€ 29,342,835	-€ 39,123,779
1081	0.19	€ 300,225	€ 600,450	€ 900,674	##########	-€ 7,259,346	-€ 14,518,692	-€ 21,778,038	-€ 29,037,384
1082	0.18	€ 280,648	€ 561,296	€ 841,943	##########	-€ 6,785,981	-€ 13,571,962	-€ 20,357,943	-€ 27,143,924
1083	0.20	€ 308,128	€ 616,256	€ 924,385	##########	-€ 7,450,448	-€ 14,900,895	-€ 22,351,343	-€ 29,801,791
1084	0.13	€ 203,280	€ 406,561	€ 609,841	€ 813,121	-€ 4,915,258	-€ 9,830,516	-€ 14,745,774	-€ 19,661,032
1085	0.19	€ 297,769	€ 595,538	€ 893,307	##########	-€ 7,199,964	-€ 14,399,928	-€ 21,599,891	-€ 28,799,855
1086	0.20	€ 317,475	€ 634,951	€ 952,426	##########	-€ 7,676,461	-€ 15,352,923	-€ 23,029,384	-€ 30,705,846
1087	0.15	€ 238,783	€ 477,566	€ 716,349	€ 955,132	-€ 5,773,705	-€ 11,547,410	-€ 17,321,114	-€ 23,094,819
1088	0.11	€ 180,845	€ 361,691	€ 542,536	€ 723,382	-€ 4,372,788	-€ 8,745,575	-€ 13,118,363	-€ 17,491,150
1089	0.24	€ 371,579	€ 743,159	##########	##########	-€ 8,984,678	-€ 17,969,355	-€ 26,954,033	-€ 35,938,710
1090	0.18	€ 285,995	€ 571,990	€ 857,985	##########	-€ 6,915,275	-€ 13,830,551	-€ 20,745,826	-€ 27,661,102
1091	0.18	€ 288,836	€ 577,673	€ 866,509	##########	-€ 6,983,979	-€ 13,967,958	-€ 20,951,936	-€ 27,935,915
1092	0.17	€ 266,699	€ 533,397	€ 800,096	##########	-€ 6,448,693	-€ 12,897,386	-€ 19,346,078	-€ 25,794,771
1093	0.17	€ 269,401	€ 538,802	€ 808,203	##########	-€ 6,514,039	-€ 13,028,077	-€ 19,542,116	-€ 26,056,154
1094	0.22	€ 349,484	€ 698,969	##########	##########	-€ 8,450,427	-€ 16,900,854	-€ 25,351,281	-€ 33,801,708
1095	0.18	€ 275,943	€ 551,885	€ 827,828	##########	-€ 6,672,213	-€ 13,344,425	-€ 20,016,638	-€ 26,688,850
1096	0.28	€ 445,137	€ 890,275	##########	##########	-€ 10,763,291	-€ 21,526,583	-€ 32,289,874	-€ 43,053,166
1097	0.32	€ 496,778	€ 993,556	##########	##########	-€ 12,011,940	-€ 24,023,880	-€ 36,035,820	-€ 48,047,760
1098	0.14	€ 221,336	€ 442,671	€ 664,007	€ 885,342	-€ 5,351,830	-€ 10,703,659	-€ 16,055,489	-€ 21,407,318
1099	0.15	€ 243,606	€ 487,211	€ 730,817	€ 974,423	-€ 5,890,312	-€ 11,780,624	-€ 17,670,937	-€ 23,561,249
1100	0.21	€ 334,927	€ 669,854	##########	##########	-€ 8,098,438	-€ 16,196,876	-€ 24,295,313	-€ 32,393,751
1101	0.17	€ 275,501	€ 551,002	€ 826,502	##########	-€ 6,661,528	-€ 13,323,055	-€ 19,984,583	-€ 26,646,111
1102	0.20	€ 316,244	€ 632,489	€ 948,733	##########	-€ 7,646,693	-€ 15,293,386	-€ 22,940,079	-€ 30,586,773
1103	0.17	€ 263,962	€ 527,925	€ 791,887	##########	-€ 6,382,532	-€ 12,765,065	-€ 19,147,597	-€ 25,530,130
1104	0.20	€ 307,866	€ 615,733	€ 923,599	##########	-€ 7,444,118	-€ 14,888,236	-€ 22,332,355	-€ 29,776,473
1105	0.16	€ 251,430	€ 502,859	€ 754,289	##########	-€ 6,079,492	-€ 12,158,985	-€ 18,238,477	-€ 24,317,969
1106	0.20	€ 317,800	€ 635,599	€ 953,399	##########	-€ 7,684,303	-€ 15,368,607	-€ 23,052,910	-€ 30,737,214
1107	0.20	€ 318,152	€ 636,303	€ 954,455	##########	-€ 7,692,810	-€ 15,385,621	-€ 23,078,431	-€ 30,771,241
1108	0.18	€ 283,126	€ 566,252	€ 849,378	##########	-€ 6,845,900	-€ 13,691,800	-€ 20,537,699	-€ 27,383,599
1109	0.14	€ 227,761	€ 455,522	€ 683,284	€ 911,045	-€ 5,507,199	-€ 11,014,398	-€ 16,521,597	-€ 22,028,796
1110	0.23	€ 367,550	€ 735,100	##########	##########	-€ 8,887,244	-€ 17,774,488	-€ 26,661,732	-€ 35,548,976
1111	0.24	€ 377,252	€ 754,504	##########	##########	-€ 9,121,844	-€ 18,243,689	-€ 27,365,533	-€ 36,487,378
1112	0.18	€ 275,682	€ 551,364	€ 827,046	##########	-€ 6,665,905	-€ 13,331,810	-€ 19,997,715	-€ 26,663,620
1113	0.17	€ 274,565	€ 549,130	€ 823,695	##########	-€ 6,638,896	-€ 13,277,793	-€ 19,916,689	-€ 26,555,585
1114	0.17	€ 270,748	€ 541,495	€ 812,243	##########	-€ 6,546,600	-€ 13,093,200	-€ 19,639,800	-€ 26,186,401
1115	0.19	€ 293,569	€ 587,138	€ 880,707	##########	-€ 7,098,414	-€ 14,196,827	-€ 21,295,241	-€ 28,393,655
1116	0.21	€ 323,144	€ 646,288	€ 969,432	##########	-€ 7,813,526	-€ 15,627,051	-€ 23,440,577	-€ 31,254,102
1117	0.16	€ 251,119	€ 502,239	€ 753,358	##########	-€ 6,071,995	-€ 12,143,989	-€ 18,215,984	-€ 24,287,978
1118	0.23	€ 366,807	€ 733,614	##########	##########	-€ 8,869,280	-€ 17,738,560	-€ 26,607,840	-€ 35,477,120

1119	0.19	€ 304,020	€ 608,041	€ 912,061	##########	-€ 7,351,120	-€ 14,702,240	-€ 22,053,360	-€ 29,404,479
1120	0.13	€ 210,118	€ 420,236	€ 630,354	€ 840,472	-€ 5,080,590	-€ 10,161,181	-€ 15,241,771	-€ 20,322,362
1121	0.18	€ 288,436	€ 576,871	€ 865,307	##########	-€ 6,974,290	-€ 13,948,581	-€ 20,922,871	-€ 27,897,161
1122	0.22	€ 343,333	€ 686,666	##########	##########	-€ 8,301,688	-€ 16,603,377	-€ 24,905,065	-€ 33,206,753
1123	0.16	€ 250,487	€ 500,973	€ 751,460	##########	-€ 6,056,692	-€ 12,113,384	-€ 18,170,075	-€ 24,226,767
1124	0.16	€ 258,429	€ 516,859	€ 775,288	##########	-€ 6,248,744	-€ 12,497,488	-€ 18,746,232	-€ 24,994,976
1125	0.15	€ 243,109	€ 486,219	€ 729,328	€ 972,438	-€ 5,878,314	-€ 11,756,628	-€ 17,634,942	-€ 23,513,256
1126	0.15	€ 230,275	€ 460,550	€ 690,825	€ 921,100	-€ 5,567,981	-€ 11,135,963	-€ 16,703,944	-€ 22,271,925
1127	0.17	€ 274,987	€ 549,973	€ 824,960	##########	-€ 6,649,094	-€ 13,298,189	-€ 19,947,283	-€ 26,596,378
1128	0.25	€ 393,461	€ 786,922	##########	##########	-€ 9,513,772	-€ 19,027,544	-€ 28,541,316	-€ 38,055,087
1129	0.16	€ 244,238	€ 488,475	€ 732,713	€ 976,950	-€ 5,905,592	-€ 11,811,183	-€ 17,716,775	-€ 23,622,367
1130	0.21	€ 329,524	€ 659,048	€ 988,572	##########	-€ 7,967,793	-€ 15,935,587	-€ 23,903,380	-€ 31,871,173
1131	0.16	€ 257,343	€ 514,687	€ 772,030	##########	-€ 6,222,489	-€ 12,444,978	-€ 18,667,467	-€ 24,889,956
1132	0.11	€ 176,068	€ 352,136	€ 528,205	€ 704,273	-€ 4,257,277	-€ 8,514,554	-€ 12,771,831	-€ 17,029,108
1133	0.18	€ 282,482	€ 564,963	€ 847,445	##########	-€ 6,830,322	-€ 13,660,644	-€ 20,490,966	-€ 27,321,288
1134	0.22	€ 343,127	€ 686,254	##########	##########	-€ 8,296,712	-€ 16,593,424	-€ 24,890,136	-€ 33,186,848
1135	0.25	€ 391,152	€ 782,304	##########	##########	-€ 9,457,935	-€ 18,915,870	-€ 28,373,806	-€ 37,831,741
1136	0.22	€ 347,023	€ 694,045	##########	##########	-€ 8,390,904	-€ 16,781,808	-€ 25,172,711	-€ 33,563,615
1137	0.14	€ 227,301	€ 454,603	€ 681,904	€ 909,205	-€ 5,496,077	-€ 10,992,154	-€ 16,488,232	-€ 21,984,309
1138	0.17	€ 267,016	€ 534,032	€ 801,047	##########	-€ 6,456,362	-€ 12,912,725	-€ 19,369,087	-€ 25,825,450
1139	0.23	€ 354,714	€ 709,428	##########	##########	-€ 8,576,874	-€ 17,153,749	-€ 25,730,623	-€ 34,307,498
1140	0.21	€ 328,545	€ 657,090	€ 985,635	##########	-€ 7,944,118	-€ 15,888,236	-€ 23,832,354	-€ 31,776,472
1141	0.22	€ 346,484	€ 692,968	##########	##########	-€ 8,377,877	-€ 16,755,754	-€ 25,133,631	-€ 33,511,508
1142	0.12	€ 190,342	€ 380,684	€ 571,026	€ 761,368	-€ 4,602,415	-€ 9,204,829	-€ 13,807,244	-€ 18,409,659
1143	0.20	€ 322,346	€ 644,692	€ 967,039	##########	-€ 7,794,236	-€ 15,588,471	-€ 23,382,707	-€ 31,176,943
1144	0.20	€ 309,677	€ 619,353	€ 929,030	##########	-€ 7,487,890	-€ 14,975,779	-€ 22,463,669	-€ 29,951,559
1145	0.15	€ 240,085	€ 480,170	€ 720,255	€ 960,339	-€ 5,805,181	-€ 11,610,362	-€ 17,415,543	-€ 23,220,724
1146	0.23	€ 369,554	€ 739,108	##########	##########	-€ 8,935,706	-€ 17,871,411	-€ 26,807,117	-€ 35,742,823
1147	0.12	€ 196,227	€ 392,454	€ 588,681	€ 784,908	-€ 4,744,709	-€ 9,489,418	-€ 14,234,127	-€ 18,978,836
1148	0.25	€ 387,098	€ 774,196	##########	##########	-€ 9,359,920	-€ 18,719,840	-€ 28,079,760	-€ 37,439,680
1149	0.16	€ 254,660	€ 509,320	€ 763,980	##########	-€ 6,157,607	-€ 12,315,214	-€ 18,472,820	-€ 24,630,427
1150	0.20	€ 316,106	€ 632,213	€ 948,319	##########	-€ 7,643,357	-€ 15,286,714	-€ 22,930,071	-€ 30,573,428
1151	0.15	€ 235,810	€ 471,620	€ 707,429	€ 943,239	-€ 5,701,811	-€ 11,403,623	-€ 17,105,434	-€ 22,807,246
1152	0.21	€ 337,026	€ 674,053	##########	##########	-€ 8,149,198	-€ 16,298,396	-€ 24,447,593	-€ 32,596,791
1153	0.15	€ 230,869	€ 461,737	€ 692,606	€ 923,475	-€ 5,582,336	-€ 11,164,671	-€ 16,747,007	-€ 22,329,343
1154	0.19	€ 298,226	€ 596,451	€ 894,677	##########	-€ 7,211,010	-€ 14,422,019	-€ 21,633,029	-€ 28,844,038
1155	0.19	€ 296,349	€ 592,698	€ 889,047	##########	-€ 7,165,630	-€ 14,331,259	-€ 21,496,889	-€ 28,662,518
1156	0.21	€ 330,103	€ 660,207	€ 990,310	##########	-€ 7,981,803	-€ 15,963,607	-€ 23,945,410	-€ 31,927,214
1157	0.18	€ 283,448	€ 566,897	€ 850,345	##########	-€ 6,853,697	-€ 13,707,394	-€ 20,561,090	-€ 27,414,787
1158	0.23	€ 363,405	€ 726,810	##########	##########	-€ 8,787,022	-€ 17,574,044	-€ 26,361,066	-€ 35,148,088
1159	0.23	€ 363,139	€ 726,277	##########	##########	-€ 8,780,585	-€ 17,561,170	-€ 26,341,755	-€ 35,122,340
1160	0.17	€ 262,986	€ 525,971	€ 788,957	##########	-€ 6,358,916	-€ 12,717,832	-€ 19,076,747	-€ 25,435,663
1161	0.19	€ 292,283	€ 584,565	€ 876,848	##########	-€ 7,067,308	-€ 14,134,616	-€ 21,201,924	-€ 28,269,231
1162	0.17	€ 263,273	€ 526,546	€ 789,818	##########	-€ 6,365,858	-€ 12,731,716	-€ 19,097,573	-€ 25,463,431
1163	0.25	€ 396,337	€ 792,674	##########	##########	-€ 9,583,314	-€ 19,166,629	-€ 28,749,943	-€ 38,333,258
1164	0.20	€ 317,900	€ 635,800	€ 953,701	##########	-€ 7,686,734	-€ 15,373,467	-€ 23,060,201	-€ 30,746,934
1165	0.20	€ 322,529	€ 645,059	€ 967,588	##########	-€ 7,798,665	-€ 15,597,329	-€ 23,395,994	-€ 31,194,658
1166	0.17	€ 265,552	€ 531,104	€ 796,657	##########	-€ 6,420,973	-€ 12,841,946	-€ 19,262,919	-€ 25,683,892
1167	0.15	€ 230,230	€ 460,460	€ 690,690	€ 920,920	-€ 5,566,891	-€ 11,133,781	-€ 16,700,672	-€ 22,267,562
1168	0.18	€ 277,480	€ 554,960	€ 832,440	##########	-€ 6,709,387	-€ 13,418,775	-€ 20,128,162	-€ 26,837,549
1169	0.23	€ 358,275	€ 716,550	##########	##########	-€ 8,662,983	-€ 17,325,965	-€ 25,988,948	-€ 34,651,930
1170	0.17	€ 274,495	€ 548,989	€ 823,484	##########	-€ 6,637,199	-€ 13,274,397	-€ 19,911,596	-€ 26,548,794
1171	0.18	€ 289,569	€ 579,137	€ 868,706	##########	-€ 7,001,685	-€ 14,003,370	-€ 21,005,056	-€ 28,006,741
1172	0.17	€ 264,814	€ 529,628	€ 794,442	##########	-€ 6,403,127	-€ 12,806,254	-€ 19,209,381	-€ 25,612,509
1173	0.19	€ 305,648	€ 611,296	€ 916,944	##########	-€ 7,390,482	-€ 14,780,964	-€ 22,171,446	-€ 29,561,927
1174	0.20	€ 318,329	€ 636,657	€ 954,986	##########	-€ 7,697,090	-€ 15,394,180	-€ 23,091,269	-€ 30,788,359
1175	0.18	€ 277,718	€ 555,436	€ 833,154	##########	-€ 6,715,141	-€ 13,430,282	-€ 20,145,423	-€ 26,860,564
1176	0.22	€ 346,425	€ 692,849	##########	##########	-€ 8,376,443	-€ 16,752,886	-€ 25,129,329	-€ 33,505,771
1177	0.21	€ 336,780	€ 673,561	##########	##########	-€ 8,143,252	-€ 16,286,504	-€ 24,429,756	-€ 32,573,008
1178	0.13	€ 202,520	€ 405,040	€ 607,560	€ 810,079	-€ 4,896,870	-€ 9,793,740	-€ 14,690,610	-€ 19,587,480
1179	0.20	€ 320,043	€ 640,085	€ 960,128	##########	-€ 7,738,534	-€ 15,477,067	-€ 23,215,601	-€ 30,954,134
1180	0.19	€ 296,659	€ 593,318	€ 889,977	##########	-€ 7,173,125	-€ 14,346,251	-€ 21,519,376	-€ 28,692,502
1181	0.17	€ 271,970	€ 543,940	€ 815,910	##########	-€ 6,576,150	-€ 13,152,301	-€ 19,728,451	-€ 26,304,602
1182	0.15	€ 236,934	€ 473,868	€ 710,802	€ 947,736	-€ 5,728,994	-€ 11,457,988	-€ 17,186,982	-€ 22,915,976
1183	0.22	€ 341,401	€ 682,802	##########	##########	-€ 8,254,970	-€ 16,509,941	-€ 24,764,911	-€ 33,019,881
1184	0.20	€ 320,465	€ 640,929	€ 961,394	##########	-€ 7,748,739	-€ 15,497,477	-€ 23,246,216	-€ 30,994,954
1185	0.17	€ 261,986	€ 523,972	€ 785,959	##########	-€ 6,334,750	-€ 12,669,499	-€ 19,004,249	-€ 25,338,998
1186	0.15	€ 243,444	€ 486,888	€ 730,331	€ 973,775	-€ 5,886,399	-€ 11,772,799	-€ 17,659,198	-€ 23,545,597
1187	0.14	€ 218,198	€ 436,396	€ 654,594	€ 872,792	-€ 5,275,961	-€ 10,551,922	-€ 15,827,883	-€ 21,103,843
1188	0.19	€ 295,575	€ 591,150	€ 886,725	##########	-€ 7,146,916	-€ 14,293,832	-€ 21,440,747	-€ 28,587,663
1189	0.12	€ 182,300	€ 364,599	€ 546,899	€ 729,199	-€ 4,407,954	-€ 8,815,907	-€ 13,223,861	-€ 17,631,814
1190	0.27	€ 418,496	€ 836,993	##########	##########	-€ 10,119,120	-€ 20,238,240	-€ 30,357,360	-€ 40,476,481
1191	0.18	€ 277,638	€ 555,276	€ 832,915	##########	-€ 6,713,209	-€ 13,426,417	-€ 20,139,626	-€ 26,852,834
1192	0.20	€ 308,053	€ 616,105	€ 924,158	##########	-€ 7,448,618	-€ 14,897,237	-€ 22,345,855	-€ 29,794,473
1193	0.16	€ 249,012	€ 498,024	€ 747,036	€ 996,047	-€ 6,021,033	-€ 12,042,065	-€ 18,063,098	-€ 24,084,130
1194	0.21	€ 334,348	€ 668,696	##########	##########	-€ 8,084,434	-€ 16,168,869	-€ 24,253,303	-€ 32,337,737
1195	0.23	€ 355,265	€ 710,531	##########	##########	-€ 8,590,211	-€ 17,180,422	-€ 25,770,633	-€ 34,360,844
1196	0.18	€ 289,677	€ 579,355	€ 869,032	##########	-€ 7,004,315	-€ 14,008,630	-€ 21,012,944	-€ 28,017,259
1197	0.27	€ 420,095	€ 840,191	##########	##########	-€ 10,157,783	-€ 20,315,565	-€ 30,473,348	-€ 40,631,130
1198	0.18	€ 276,789	€ 553,578	€ 830,367	##########	-€ 6,692,678	-€ 13,385,355	-€ 20,078,033	-€ 26,770,710
1199	0.20	€ 307,977	€ 615,954	€ 923,931	##########	-€ 7,446,795	-€ 14,893,590	-€ 22,340,385	-€ 29,787,180
1200	0.16	€ 248,145	€ 496,291	€ 744,436	€ 992,582	-€ 6,000,083	-€ 12,000,166	-€ 18,000,249	-€ 24,000,332
1201	0.14	€ 223,187	€ 446,374	€ 669,560	€ 892,747	-€ 5,396,589	-€ 10,793,179	-€ 16,189,768	-€ 21,586,358
1202	0.15	€ 241,192	€ 482,384	€ 723,576	€ 964,768	-€ 5,831,950	-€ 11,663,901	-€ 17,495,851	-€ 23,327,802
1203	0.16	€ 258,914	€ 517,827	€ 776,741	##########	-€ 6,260,453	-€ 12,520,905	-€ 18,781,358	-€ 25,041,810
1204	0.17	€ 273,239	€ 546,478	€ 819,717	##########	-€ 6,606,839	-€ 13,213,679	-€ 19,820,518	-€ 26,427,357
1205	0.21	€ 323,850	€ 647,700	€ 971,550	##########	-€ 7,830,594	-€ 15,661,187	-€ 23,491,781	-€ 31,322,374
1206	0.17	€ 274,205	€ 548,410	€ 822,614	##########	-€ 6,630,191	-€ 13,260,382	-€ 19,890,573	-€ 26,520,764
1207	0.14	€ 224,325	€ 448,651	€ 672,976	€ 897,301	-€ 5,424,120	-€ 10,848,241	-€ 16,272,361	-€ 21,696,481
1208	0.22	€ 341,100	€ 682,200	##########	##########	-€ 8,247,702	-€ 16,495,405	-€ 24,743,107	-€ 32,990,809
1209	0.20	€ 316,208	€ 632,417	€ 948,625	##########	-€ 7,645,826	-€ 15,291,652	-€ 22,937,478	-€ 30,583,303
1210	0.21	€ 322,893	€ 645,786	€ 968,678	##########	-€ 7,807,451	-€ 15,614,903	-€ 23,422,354	-€ 31,229,806
1211	0.21	€ 333,852	€ 667,705	##########	##########	-€ 8,072,451	-€ 16,144,902	-€ 24,217,353	-€ 32,289,804
1212	0.23	€ 365,516	€ 731,031	##########	##########	-€ 8,838,060	-€ 17,676,120	-€ 26,514,181	-€ 35,352,241
1213	0.26	€ 404,858	€ 809,717	##########	##########	-€ 9,789,355	-€ 19,578,709	-€ 29,368,064	-€ 39,157,419
1214	0.15	€ 229,578	€ 459,156	€ 688,733	€ 918,311	-€ 5,551,123	-€ 11,102,246	-€ 16,653,370	-€ 22,204,493
1215	0.18	€ 286,553	€ 573,107	€ 859,660	##########	-€ 6,928,777	-€ 13,857,553	-€ 20,786,330	-€ 27,715,106
1216	0.25	€ 391,616	€ 783,231	##########	##########	-€ 9,469,149	-€ 18,938,298	-€ 28,407,448	-€ 37,876,597
1217	0.23	€ 369,920	€ 739,840	##########	##########	-€ 8,944,550	-€ 17,889,100	-€ 26,833,650	-€ 35,778,200
1218	0.17	€ 264,727	€ 529,454	€ 794,181	##########	-€ 6,401,022	-€ 12,802,044	-€ 19,203,065	-€ 25,604,087
1219	0.17	€ 269,253	€ 538,506	€ 807,760	##########	-€ 6,510,463	-€ 13,020,926	-€ 19,531,389	-€ 26,041,852
1220	0.22	€ 347,710	€ 695,420	##########	##########	-€ 8,407,526	-€ 16,815,053	-€ 25,222,579	-€ 33,630,105

1221	0.25	€ 387,070	€ 774,141	##########	##########	-€ 9,359,248	-€ 18,718,496	-€ 28,077,744	-€ 37,436,993
1222	0.18	€ 279,274	€ 558,548	€ 837,822	##########	-€ 6,752,760	-€ 13,505,519	-€ 20,258,279	-€ 27,011,038
1223	0.23	€ 358,966	€ 717,932	##########	##########	-€ 8,679,689	-€ 17,359,379	-€ 26,039,068	-€ 34,718,757
1224	0.19	€ 298,286	€ 596,572	€ 894,858	##########	-€ 7,212,470	-€ 14,424,939	-€ 21,637,409	-€ 28,849,879
1225	0.22	€ 344,151	€ 688,302	##########	##########	-€ 8,321,469	-€ 16,642,938	-€ 24,964,407	-€ 33,285,876
1226	0.17	€ 263,924	€ 527,848	€ 791,771	##########	-€ 6,381,599	-€ 12,763,197	-€ 19,144,796	-€ 25,526,395
1227	0.32	€ 504,603	########	##########	##########	-€ 12,201,149	-€ 24,402,298	-€ 36,603,447	-€ 48,804,596
1228	0.25	€ 386,521	€ 773,042	##########	##########	-€ 9,345,964	-€ 18,691,927	-€ 28,037,891	-€ 37,383,854
1229	0.13	€ 206,516	€ 413,031	€ 619,547	€ 826,063	-€ 4,993,488	-€ 9,986,976	-€ 14,980,463	-€ 19,973,951
1230	0.22	€ 339,118	€ 678,235	##########	##########	-€ 8,199,761	-€ 16,399,523	-€ 24,599,284	-€ 32,799,046
1231	0.15	€ 229,246	€ 458,492	€ 687,737	€ 916,983	-€ 5,543,095	-€ 11,086,190	-€ 16,629,284	-€ 22,172,379
1232	0.19	€ 306,390	€ 612,779	€ 919,169	##########	-€ 7,408,408	-€ 14,816,816	-€ 22,225,223	-€ 29,633,631
1233	0.23	€ 355,608	€ 711,215	##########	##########	-€ 8,598,488	-€ 17,196,976	-€ 25,795,464	-€ 34,393,953
1234	0.20	€ 310,529	€ 621,059	€ 931,588	##########	-€ 7,508,509	-€ 15,017,018	-€ 22,525,527	-€ 30,034,035
1235	0.20	€ 310,843	€ 621,686	€ 932,529	##########	-€ 7,516,092	-€ 15,032,184	-€ 22,548,275	-€ 30,064,367
1236	0.17	€ 267,065	€ 534,129	€ 801,194	##########	-€ 6,457,546	-€ 12,915,091	-€ 19,372,637	-€ 25,830,182
1237	0.15	€ 232,000	€ 464,001	€ 696,001	€ 928,001	-€ 5,609,699	-€ 11,219,398	-€ 16,829,097	-€ 22,438,796
1238	0.20	€ 318,814	€ 637,628	€ 956,441	##########	-€ 7,708,822	-€ 15,417,644	-€ 23,126,466	-€ 30,835,288
1239	0.17	€ 264,683	€ 529,366	€ 794,048	##########	-€ 6,399,951	-€ 12,799,902	-€ 19,199,852	-€ 25,599,803
1240	0.13	€ 209,521	€ 419,041	€ 628,562	€ 838,082	-€ 5,066,146	-€ 10,132,293	-€ 15,198,439	-€ 20,264,586
1241	0.17	€ 267,579	€ 535,158	€ 802,737	##########	-€ 6,469,978	-€ 12,939,957	-€ 19,409,935	-€ 25,879,913
1242	0.17	€ 261,106	€ 522,212	€ 783,318	##########	-€ 6,313,466	-€ 12,626,931	-€ 18,940,397	-€ 25,253,862
1243	0.14	€ 218,871	€ 437,742	€ 656,613	€ 875,483	-€ 5,292,232	-€ 10,584,465	-€ 15,876,697	-€ 21,168,929
1244	0.20	€ 311,010	€ 622,020	€ 933,030	##########	-€ 7,520,132	-€ 15,040,264	-€ 22,560,397	-€ 30,080,529
1245	0.10	€ 158,134	€ 316,269	€ 474,403	€ 632,537	-€ 3,823,640	-€ 7,647,280	-€ 11,470,920	-€ 15,294,560
1246	0.18	€ 278,053	€ 556,105	€ 834,158	##########	-€ 6,723,228	-€ 13,446,455	-€ 20,169,683	-€ 26,892,911
1247	0.17	€ 273,812	€ 547,624	€ 821,436	##########	-€ 6,620,694	-€ 13,241,388	-€ 19,862,082	-€ 26,482,776
1248	0.30	€ 467,459	€ 934,919	##########	##########	-€ 11,303,028	-€ 22,606,055	-€ 33,909,083	-€ 45,212,110
1249	0.24	€ 385,282	€ 770,564	##########	##########	-€ 9,316,010	-€ 18,632,020	-€ 27,948,030	-€ 37,264,039
1250	0.18	€ 289,084	€ 578,168	€ 867,252	##########	-€ 6,989,965	-€ 13,979,929	-€ 20,969,894	-€ 27,959,858
1251	0.12	€ 190,464	€ 380,929	€ 571,393	€ 761,857	-€ 4,605,369	-€ 9,210,739	-€ 13,816,108	-€ 18,421,477
1252	0.21	€ 338,279	€ 676,559	##########	##########	-€ 8,179,495	-€ 16,358,990	-€ 24,538,485	-€ 32,717,980
1253	0.20	€ 317,373	€ 634,746	€ 952,118	##########	-€ 7,673,979	-€ 15,347,959	-€ 23,021,938	-€ 30,695,918
1254	0.19	€ 301,505	€ 603,011	€ 904,516	##########	-€ 7,290,311	-€ 14,580,621	-€ 21,870,932	-€ 29,161,242
1255	0.20	€ 314,200	€ 628,399	€ 942,599	##########	-€ 7,597,253	-€ 15,194,506	-€ 22,791,760	-€ 30,389,013
1256	0.23	€ 363,131	€ 726,261	##########	##########	-€ 8,780,389	-€ 17,560,778	-€ 26,341,167	-€ 35,121,557
1257	0.24	€ 379,214	€ 758,428	##########	##########	-€ 9,169,281	-€ 18,338,562	-€ 27,507,842	-€ 36,677,123
1258	0.15	€ 235,541	€ 471,082	€ 706,624	€ 942,165	-€ 5,695,316	-€ 11,390,631	-€ 17,085,947	-€ 22,781,262
1259	0.15	€ 229,379	€ 458,758	€ 688,137	€ 917,516	-€ 5,546,316	-€ 11,092,633	-€ 16,638,949	-€ 22,185,265
1260	0.20	€ 319,113	€ 638,225	€ 957,338	##########	-€ 7,716,048	-€ 15,432,096	-€ 23,148,145	-€ 30,864,193
1261	0.20	€ 321,579	€ 643,159	€ 964,738	##########	-€ 7,775,696	-€ 15,551,392	-€ 23,327,088	-€ 31,102,784
1262	0.15	€ 237,748	€ 475,497	€ 713,245	€ 950,994	-€ 5,748,686	-€ 11,497,372	-€ 17,246,058	-€ 22,994,744
1263	0.24	€ 382,606	€ 765,212	##########	##########	-€ 9,251,298	-€ 18,502,596	-€ 27,753,894	-€ 37,005,192
1264	0.14	€ 227,088	€ 454,176	€ 681,265	€ 908,353	-€ 5,490,924	-€ 10,981,849	-€ 16,472,773	-€ 21,963,698
1265	0.15	€ 229,044	€ 458,089	€ 687,133	€ 916,178	-€ 5,538,227	-€ 11,076,453	-€ 16,614,680	-€ 22,152,906
1266	0.24	€ 371,182	€ 742,363	##########	##########	-€ 8,975,059	-€ 17,950,118	-€ 26,925,178	-€ 35,900,237
1267	0.21	€ 332,096	€ 664,192	€ 996,288	##########	-€ 8,029,980	-€ 16,059,959	-€ 24,089,939	-€ 32,119,918
1268	0.20	€ 318,005	€ 636,009	€ 954,014	##########	-€ 7,689,256	-€ 15,378,512	-€ 23,067,768	-€ 30,757,023
1269	0.27	€ 425,512	€ 851,025	##########	##########	-€ 10,288,766	-€ 20,577,531	-€ 30,866,297	-€ 41,155,062
1270	0.26	€ 403,960	€ 807,919	##########	##########	-€ 9,767,626	-€ 19,535,252	-€ 29,302,877	-€ 39,070,503
1271	0.23	€ 354,764	€ 709,528	##########	##########	-€ 8,578,089	-€ 17,156,178	-€ 25,734,267	-€ 34,312,356
1272	0.26	€ 403,593	€ 807,186	##########	##########	-€ 9,758,758	-€ 19,517,516	-€ 29,276,275	-€ 39,035,033
1273	0.24	€ 382,729	€ 765,459	##########	##########	-€ 9,254,280	-€ 18,508,561	-€ 27,762,841	-€ 37,017,121
1274	0.20	€ 318,026	€ 636,052	€ 954,078	##########	-€ 7,689,772	-€ 15,379,544	-€ 23,069,315	-€ 30,759,087
1275	0.17	€ 266,839	€ 533,678	€ 800,517	##########	-€ 6,452,086	-€ 12,904,172	-€ 19,356,258	-€ 25,808,345
1276	0.14	€ 221,672	€ 443,345	€ 665,017	€ 886,689	-€ 5,359,971	-€ 10,719,943	-€ 16,079,914	-€ 21,439,886
1277	0.14	€ 216,278	€ 432,556	€ 648,834	€ 865,112	-€ 5,229,537	-€ 10,459,073	-€ 15,688,610	-€ 20,918,147
1278	0.21	€ 327,330	€ 654,660	€ 981,990	##########	-€ 7,914,745	-€ 15,829,490	-€ 23,744,235	-€ 31,658,980
1279	0.15	€ 242,262	€ 484,525	€ 726,787	€ 969,050	-€ 5,857,834	-€ 11,715,667	-€ 17,573,501	-€ 23,431,334
1280	0.17	€ 263,110	€ 526,221	€ 789,331	##########	-€ 6,361,929	-€ 12,723,858	-€ 19,085,788	-€ 25,447,717
1281	0.20	€ 307,083	€ 614,166	€ 921,249	##########	-€ 7,425,178	-€ 14,850,356	-€ 22,275,533	-€ 29,700,711
1282	0.22	€ 341,073	€ 682,146	##########	##########	-€ 8,247,038	-€ 16,494,077	-€ 24,741,115	-€ 32,988,154
1283	0.13	€ 209,233	€ 418,466	€ 627,699	€ 836,933	-€ 5,059,196	-€ 10,118,391	-€ 15,177,587	-€ 20,236,783
1284	0.23	€ 355,432	€ 710,864	##########	##########	-€ 8,594,241	-€ 17,188,483	-€ 25,782,724	-€ 34,376,966
1285	0.16	€ 247,617	€ 495,234	€ 742,851	€ 990,468	-€ 5,987,304	-€ 11,974,609	-€ 17,961,913	-€ 23,949,218
1286	0.13	€ 208,452	€ 416,905	€ 625,357	€ 833,810	-€ 5,040,319	-€ 10,080,638	-€ 15,120,957	-€ 20,161,276
1287	0.26	€ 402,384	€ 804,767	##########	##########	-€ 9,729,519	-€ 19,459,039	-€ 29,188,558	-€ 38,918,077
1288	0.16	€ 250,904	€ 501,808	€ 752,712	##########	-€ 6,066,788	-€ 12,133,575	-€ 18,200,363	-€ 24,267,150
1289	0.11	€ 166,080	€ 332,160	€ 498,241	€ 664,321	-€ 4,015,770	-€ 8,031,541	-€ 12,047,311	-€ 16,063,082
1290	0.15	€ 231,367	€ 462,734	€ 694,101	€ 925,468	-€ 5,594,385	-€ 11,188,770	-€ 16,783,155	-€ 22,377,540
1291	0.24	€ 370,376	€ 740,753	##########	##########	-€ 8,955,588	-€ 17,911,177	-€ 26,866,765	-€ 35,822,353
1292	0.15	€ 236,787	€ 473,574	€ 710,361	€ 947,147	-€ 5,725,436	-€ 11,450,872	-€ 17,176,308	-€ 22,901,744
1293	0.18	€ 284,191	€ 568,382	€ 852,573	##########	-€ 6,871,652	-€ 13,743,303	-€ 20,614,955	-€ 27,486,607
1294	0.19	€ 304,294	€ 608,588	€ 912,883	##########	-€ 7,357,744	-€ 14,715,489	-€ 22,073,233	-€ 29,430,978
1295	0.13	€ 209,355	€ 418,710	€ 628,065	€ 837,420	-€ 5,062,141	-€ 10,124,282	-€ 15,186,423	-€ 20,248,564
1296	0.22	€ 348,950	€ 697,899	##########	##########	-€ 8,437,500	-€ 16,874,999	-€ 25,312,499	-€ 33,749,998
1297	0.15	€ 239,250	€ 478,500	€ 717,749	€ 956,999	-€ 5,784,988	-€ 11,569,976	-€ 17,354,965	-€ 23,139,953
1298	0.18	€ 280,708	€ 561,416	€ 842,124	##########	-€ 6,787,434	-€ 13,574,869	-€ 20,362,303	-€ 27,149,737
1299	0.14	€ 227,041	€ 454,081	€ 681,122	€ 908,162	-€ 5,489,772	-€ 10,979,545	-€ 16,469,317	-€ 21,959,089
1300	0.16	€ 245,768	€ 491,536	€ 737,304	€ 983,072	-€ 5,942,600	-€ 11,885,199	-€ 17,827,799	-€ 23,770,398
1301	0.22	€ 342,237	€ 684,473	##########	##########	-€ 8,275,179	-€ 16,550,357	-€ 24,825,536	-€ 33,100,714
1302	0.17	€ 260,524	€ 521,047	€ 781,571	##########	-€ 6,299,386	-€ 12,598,772	-€ 18,898,158	-€ 25,197,545
1303	0.10	€ 164,778	€ 329,555	€ 494,333	€ 659,110	-€ 3,984,271	-€ 7,968,542	-€ 11,952,813	-€ 15,937,084
1304	0.18	€ 288,033	€ 576,066	€ 864,098	##########	-€ 6,964,548	-€ 13,929,097	-€ 20,893,645	-€ 27,858,194
1305	0.21	€ 328,396	€ 656,791	€ 985,187	##########	-€ 7,940,510	-€ 15,881,020	-€ 23,821,530	-€ 31,762,040
1306	0.19	€ 291,933	€ 583,867	€ 875,800	##########	-€ 7,058,861	-€ 14,117,721	-€ 21,176,582	-€ 28,235,443
1307	0.17	€ 259,823	€ 519,647	€ 779,470	##########	-€ 6,282,454	-€ 12,564,907	-€ 18,847,361	-€ 25,129,815
1308	0.19	€ 292,512	€ 585,025	€ 877,537	##########	-€ 7,072,861	-€ 14,145,721	-€ 21,218,582	-€ 28,291,443
1309	0.19	€ 296,753	€ 593,506	€ 890,259	##########	-€ 7,175,402	-€ 14,350,803	-€ 21,526,205	-€ 28,701,607
1310	0.20	€ 321,740	€ 643,479	€ 965,219	##########	-€ 7,779,569	-€ 15,559,139	-€ 23,338,708	-€ 31,118,277
1311	0.18	€ 291,037	€ 582,074	€ 873,111	##########	-€ 7,037,189	-€ 14,074,378	-€ 21,111,568	-€ 28,148,757
1312	0.20	€ 313,627	€ 627,254	€ 940,881	##########	-€ 7,583,410	-€ 15,166,821	-€ 22,750,231	-€ 30,333,642
1313	0.19	€ 306,598	€ 613,196	€ 919,794	##########	-€ 7,413,450	-€ 14,826,900	-€ 22,240,350	-€ 29,653,801
1314	0.12	€ 185,364	€ 370,729	€ 556,093	€ 741,457	-€ 4,482,054	-€ 8,964,108	-€ 13,446,162	-€ 17,928,216
1315	0.15	€ 235,454	€ 470,907	€ 706,361	€ 941,815	-€ 5,693,199	-€ 11,386,399	-€ 17,079,598	-€ 22,772,798
1316	0.17	€ 267,928	€ 535,856	€ 803,784	##########	-€ 6,478,417	-€ 12,956,835	-€ 19,435,252	-€ 25,913,670
1317	0.19	€ 293,925	€ 587,850	€ 881,774	##########	-€ 7,107,015	-€ 14,214,030	-€ 21,321,045	-€ 28,428,060
1318	0.18	€ 289,856	€ 579,712	€ 869,568	##########	-€ 7,008,634	-€ 14,017,269	-€ 21,025,903	-€ 28,034,538
1319	0.16	€ 251,392	€ 502,784	€ 754,177	##########	-€ 6,078,589	-€ 12,157,178	-€ 18,235,767	-€ 24,314,356
1320	0.19	€ 294,891	€ 589,782	€ 884,674	##########	-€ 7,130,382	-€ 14,260,763	-€ 21,391,145	-€ 28,521,526
1321	0.20	€ 308,157	€ 616,314	€ 924,471	##########	-€ 7,451,142	-€ 14,902,284	-€ 22,353,425	-€ 29,804,567
1322	0.17	€ 268,206	€ 536,411	€ 804,617	##########	-€ 6,485,130	-€ 12,970,261	-€ 19,455,391	-€ 25,940,522

1323	0.21	€ 328,105	€ 656,210	€ 984,316	##########	-€ 7,933,486	-€ 15,866,972	-€ 23,800,459	-€ 31,733,945
1324	0.17	€ 267,322	€ 534,643	€ 801,965	##########	-€ 6,463,756	-€ 12,927,512	-€ 19,391,268	-€ 25,855,024
1325	0.19	€ 304,593	€ 609,187	€ 913,780	##########	-€ 7,364,978	-€ 14,729,955	-€ 22,094,933	-€ 29,459,911
1326	0.19	€ 294,079	€ 588,157	€ 882,236	##########	-€ 7,110,732	-€ 14,221,464	-€ 21,332,196	-€ 28,442,928
1327	0.23	€ 363,616	€ 727,231	##########	##########	-€ 8,792,118	-€ 17,584,235	-€ 26,376,353	-€ 35,168,471
1328	0.16	€ 246,784	€ 493,569	€ 740,353	€ 987,137	-€ 5,967,170	-€ 11,934,341	-€ 17,901,511	-€ 23,868,682
1329	0.23	€ 359,918	€ 719,836	##########	##########	-€ 8,702,706	-€ 17,405,412	-€ 26,108,119	-€ 34,810,825
1330	0.18	€ 277,524	€ 555,049	€ 832,573	##########	-€ 6,710,458	-€ 13,420,917	-€ 20,131,375	-€ 26,841,833
1331	0.15	€ 235,987	€ 471,975	€ 707,962	€ 943,950	-€ 5,706,105	-€ 11,412,211	-€ 17,118,316	-€ 22,824,421
1332	0.27	€ 421,415	€ 842,831	##########	##########	-€ 10,189,701	-€ 20,379,402	-€ 30,569,103	-€ 40,758,805
1333	0.12	€ 196,006	€ 392,012	€ 588,018	€ 784,024	-€ 4,739,365	-€ 9,478,729	-€ 14,218,094	-€ 18,957,458
1334	0.14	€ 215,068	€ 430,135	€ 645,203	€ 860,270	-€ 5,200,271	-€ 10,400,541	-€ 15,600,812	-€ 20,801,082
1335	0.15	€ 243,363	€ 486,727	€ 730,090	€ 973,454	-€ 5,884,454	-€ 11,768,909	-€ 17,653,363	-€ 23,537,817
1336	0.18	€ 288,216	€ 576,433	€ 864,649	##########	-€ 6,968,985	-€ 13,937,970	-€ 20,906,954	-€ 27,875,939
1337	0.15	€ 240,534	€ 481,068	€ 721,602	€ 962,135	-€ 5,816,037	-€ 11,632,075	-€ 17,448,112	-€ 23,264,149
1338	0.18	€ 285,810	€ 571,619	€ 857,429	##########	-€ 6,910,789	-€ 13,821,578	-€ 20,732,367	-€ 27,643,156
1339	0.16	€ 253,245	€ 506,489	€ 759,734	##########	-€ 6,123,378	-€ 12,246,756	-€ 18,370,134	-€ 24,493,512
1340	0.21	€ 327,884	€ 655,768	€ 983,652	##########	-€ 7,928,134	-€ 15,856,269	-€ 23,784,403	-€ 31,712,537
1341	0.18	€ 282,722	€ 565,445	€ 848,167	##########	-€ 6,836,146	-€ 13,672,292	-€ 20,508,438	-€ 27,344,584
1342	0.23	€ 357,362	€ 714,724	##########	##########	-€ 8,640,905	-€ 17,281,810	-€ 25,922,714	-€ 34,563,619
1343	0.16	€ 256,110	€ 512,221	€ 768,331	##########	-€ 6,192,673	-€ 12,385,347	-€ 18,578,020	-€ 24,770,694
1344	0.18	€ 286,592	€ 573,184	€ 859,776	##########	-€ 6,929,710	-€ 13,859,420	-€ 20,789,131	-€ 27,718,841
1345	0.21	€ 323,245	€ 646,490	€ 969,735	##########	-€ 7,815,965	-€ 15,631,929	-€ 23,447,894	-€ 31,263,858
1346	0.24	€ 370,793	€ 741,585	##########	##########	-€ 8,965,654	-€ 17,931,308	-€ 26,896,962	-€ 35,862,617
1347	0.24	€ 384,758	€ 769,515	##########	##########	-€ 9,303,324	-€ 18,606,647	-€ 27,909,971	-€ 37,213,295
1348	0.27	€ 421,967	€ 843,934	##########	##########	-€ 10,203,042	-€ 20,406,084	-€ 30,609,126	-€ 40,812,168
1349	0.15	€ 236,713	€ 473,426	€ 710,138	€ 946,851	-€ 5,723,646	-€ 11,447,291	-€ 17,170,937	-€ 22,894,583
1350	0.20	€ 314,580	€ 629,161	€ 943,741	##########	-€ 7,606,461	-€ 15,212,922	-€ 22,819,383	-€ 30,425,845
1351	0.19	€ 302,618	€ 605,236	€ 907,854	##########	-€ 7,317,214	-€ 14,634,428	-€ 21,951,642	-€ 29,268,856
1352	0.24	€ 375,050	€ 750,100	##########	##########	-€ 9,068,602	-€ 18,137,205	-€ 27,205,807	-€ 36,274,410
1353	0.17	€ 261,618	€ 523,236	€ 784,854	##########	-€ 6,325,846	-€ 12,651,693	-€ 18,977,539	-€ 25,303,386
1354	0.16	€ 258,413	€ 516,826	€ 775,238	##########	-€ 6,248,345	-€ 12,496,689	-€ 18,745,034	-€ 24,993,379
1355	0.14	€ 220,931	€ 441,861	€ 662,792	€ 883,723	-€ 5,342,040	-€ 10,684,079	-€ 16,026,119	-€ 21,368,158
1356	0.24	€ 381,079	€ 762,158	##########	##########	-€ 9,214,380	-€ 18,428,761	-€ 27,643,141	-€ 36,857,522
1357	0.11	€ 172,412	€ 344,825	€ 517,237	€ 689,649	-€ 4,168,879	-€ 8,337,759	-€ 12,506,638	-€ 16,675,517
1358	0.13	€ 198,840	€ 397,680	€ 596,520	€ 795,361	-€ 4,807,896	-€ 9,615,792	-€ 14,423,688	-€ 19,231,584
1359	0.22	€ 344,482	€ 688,964	##########	##########	-€ 8,329,474	-€ 16,658,948	-€ 24,988,422	-€ 33,317,896
1360	0.19	€ 305,022	€ 610,043	€ 915,065	##########	-€ 7,375,335	-€ 14,750,670	-€ 22,126,005	-€ 29,501,340
1361	0.24	€ 378,159	€ 756,317	##########	##########	-€ 9,143,765	-€ 18,287,531	-€ 27,431,296	-€ 36,575,062
1362	0.12	€ 191,605	€ 383,210	€ 574,816	€ 766,421	-€ 4,632,956	-€ 9,265,913	-€ 13,898,869	-€ 18,531,826
1363	0.17	€ 265,996	€ 531,992	€ 797,988	##########	-€ 6,431,706	-€ 12,863,411	-€ 19,295,117	-€ 25,726,823
1364	0.23	€ 359,995	€ 719,991	##########	##########	-€ 8,704,581	-€ 17,409,162	-€ 26,113,743	-€ 34,818,324
1365	0.10	€ 165,059	€ 330,118	€ 495,176	€ 660,235	-€ 3,991,072	-€ 7,982,144	-€ 11,973,216	-€ 15,964,288
1366	0.23	€ 355,256	€ 710,513	##########	##########	-€ 8,589,992	-€ 17,179,983	-€ 25,769,975	-€ 34,359,967
1367	0.25	€ 393,797	€ 787,594	##########	##########	-€ 9,521,898	-€ 19,043,797	-€ 28,565,695	-€ 38,087,593
1368	0.14	€ 221,435	€ 442,869	€ 664,304	€ 885,739	-€ 5,354,224	-€ 10,708,448	-€ 16,062,672	-€ 21,416,895
1369	0.22	€ 351,694	€ 703,388	##########	##########	-€ 8,503,861	-€ 17,007,723	-€ 25,511,584	-€ 34,015,446
1370	0.17	€ 265,929	€ 531,859	€ 797,788	##########	-€ 6,430,094	-€ 12,860,187	-€ 19,290,281	-€ 25,720,374
1371	0.19	€ 300,575	€ 601,149	€ 901,724	##########	-€ 7,267,805	-€ 14,535,611	-€ 21,803,416	-€ 29,071,221
1372	0.16	€ 246,610	€ 493,220	€ 739,830	€ 986,440	-€ 5,962,957	-€ 11,925,915	-€ 17,888,872	-€ 23,851,830
1373	0.18	€ 290,504	€ 581,008	€ 871,513	##########	-€ 7,024,306	-€ 14,048,611	-€ 21,072,917	-€ 28,097,222
1374	0.26	€ 403,734	€ 807,468	##########	##########	-€ 9,762,165	-€ 19,524,330	-€ 29,286,494	-€ 39,048,659
1375	0.29	€ 455,089	€ 910,177	##########	##########	-€ 11,003,906	-€ 22,007,812	-€ 33,011,719	-€ 44,015,625
1376	0.20	€ 309,442	€ 618,885	€ 928,327	##########	-€ 7,482,228	-€ 14,964,455	-€ 22,446,683	-€ 29,928,910
1377	0.17	€ 270,306	€ 540,612	€ 810,918	##########	-€ 6,535,920	-€ 13,071,839	-€ 19,607,759	-€ 26,143,678
1378	0.15	€ 236,925	€ 473,851	€ 710,776	€ 947,702	-€ 5,728,787	-€ 11,457,574	-€ 17,186,361	-€ 22,915,149
1379	0.20	€ 311,686	€ 623,373	€ 935,059	##########	-€ 7,536,487	-€ 15,072,973	-€ 22,609,460	-€ 30,145,946
1380	0.19	€ 292,824	€ 585,649	€ 878,473	##########	-€ 7,080,404	-€ 14,160,808	-€ 21,241,212	-€ 28,321,616
1381	0.12	€ 181,734	€ 363,469	€ 545,203	€ 726,937	-€ 4,394,281	-€ 8,788,563	-€ 13,182,844	-€ 17,577,126
1382	0.21	€ 333,318	€ 666,635	€ 999,953	##########	-€ 8,059,523	-€ 16,119,046	-€ 24,178,569	-€ 32,238,091
1383	0.26	€ 406,852	€ 813,705	##########	##########	-€ 9,837,568	-€ 19,675,137	-€ 29,512,705	-€ 39,350,273
1384	0.18	€ 277,646	€ 555,293	€ 832,939	##########	-€ 6,713,409	-€ 13,426,818	-€ 20,140,227	-€ 26,853,636
1385	0.19	€ 294,709	€ 589,417	€ 884,126	##########	-€ 7,125,969	-€ 14,251,938	-€ 21,377,907	-€ 28,503,876
1386	0.15	€ 236,302	€ 472,603	€ 708,905	€ 945,206	-€ 5,713,701	-€ 11,427,403	-€ 17,141,104	-€ 22,854,805
1387	0.20	€ 315,380	€ 630,759	€ 946,139	##########	-€ 7,625,783	-€ 15,251,566	-€ 22,877,350	-€ 30,503,133
1388	0.14	€ 219,336	€ 438,673	€ 658,009	€ 877,345	-€ 5,303,487	-€ 10,606,974	-€ 15,910,461	-€ 21,213,949
1389	0.20	€ 310,901	€ 621,802	€ 932,703	##########	-€ 7,517,496	-€ 15,034,991	-€ 22,552,487	-€ 30,069,983
1390	0.16	€ 252,870	€ 505,739	€ 758,609	##########	-€ 6,114,315	-€ 12,228,629	-€ 18,342,944	-€ 24,457,258
1391	0.15	€ 240,490	€ 480,980	€ 721,471	€ 961,961	-€ 5,814,981	-€ 11,629,963	-€ 17,444,944	-€ 23,259,925
1392	0.18	€ 290,141	€ 580,283	€ 870,424	##########	-€ 7,015,532	-€ 14,031,064	-€ 21,046,596	-€ 28,062,128
1393	0.17	€ 261,911	€ 523,822	€ 785,734	##########	-€ 6,332,936	-€ 12,665,872	-€ 18,998,808	-€ 25,331,744
1394	0.26	€ 404,023	€ 808,046	##########	##########	-€ 9,769,161	-€ 19,538,323	-€ 29,307,484	-€ 39,076,646
1395	0.23	€ 359,663	€ 719,327	##########	##########	-€ 8,696,553	-€ 17,393,105	-€ 26,089,658	-€ 34,786,210
1396	0.16	€ 252,631	€ 505,261	€ 757,892	##########	-€ 6,108,533	-€ 12,217,067	-€ 18,325,600	-€ 24,434,134
1397	0.19	€ 300,147	€ 600,294	€ 900,441	##########	-€ 7,257,465	-€ 14,514,929	-€ 21,772,394	-€ 29,029,858
1398	0.14	€ 222,698	€ 445,396	€ 668,093	€ 890,791	-€ 5,384,767	-€ 10,769,533	-€ 16,154,300	-€ 21,539,067
1399	0.16	€ 245,944	€ 491,888	€ 737,831	€ 983,775	-€ 5,946,848	-€ 11,893,696	-€ 17,840,544	-€ 23,787,391
1400	0.21	€ 329,150	€ 658,301	€ 987,451	##########	-€ 7,958,755	-€ 15,917,511	-€ 23,876,266	-€ 31,835,022
1401	0.18	€ 277,270	€ 554,539	€ 831,809	##########	-€ 6,704,297	-€ 13,408,594	-€ 20,112,890	-€ 26,817,187
1402	0.29	€ 462,400	€ 924,801	##########	##########	-€ 11,180,704	-€ 22,361,409	-€ 33,542,113	-€ 44,722,818
1403	0.16	€ 254,846	€ 509,691	€ 764,537	##########	-€ 6,162,090	-€ 12,324,180	-€ 18,486,270	-€ 24,648,360
1404	0.18	€ 283,301	€ 566,602	€ 849,904	##########	-€ 6,850,139	-€ 13,700,277	-€ 20,550,416	-€ 27,400,554
1405	0.14	€ 217,789	€ 435,578	€ 653,367	€ 871,157	-€ 5,266,077	-€ 10,532,154	-€ 15,798,231	-€ 21,064,308
1406	0.23	€ 362,178	€ 724,356	##########	##########	-€ 8,757,352	-€ 17,514,704	-€ 26,272,056	-€ 35,029,408
1407	0.21	€ 337,251	€ 674,501	##########	##########	-€ 8,154,621	-€ 16,309,241	-€ 24,463,862	-€ 32,618,483
1408	0.25	€ 388,926	€ 777,851	##########	##########	-€ 9,404,109	-€ 18,808,218	-€ 28,212,327	-€ 37,616,436
1409	0.18	€ 276,252	€ 552,505	€ 828,757	##########	-€ 6,679,703	-€ 13,359,406	-€ 20,039,109	-€ 26,718,812
1410	0.15	€ 230,350	€ 460,699	€ 691,049	€ 921,398	-€ 5,569,785	-€ 11,139,571	-€ 16,709,356	-€ 22,279,142
1411	0.21	€ 324,252	€ 648,503	€ 972,755	##########	-€ 7,840,311	-€ 15,680,622	-€ 23,520,933	-€ 31,361,244
1412	0.14	€ 219,432	€ 438,864	€ 658,295	€ 877,727	-€ 5,305,797	-€ 10,611,593	-€ 15,917,390	-€ 21,223,186
1413	0.30	€ 479,003	€ 958,006	##########	##########	-€ 11,582,152	-€ 23,164,303	-€ 34,746,455	-€ 46,328,607
1414	0.19	€ 303,203	€ 606,406	€ 909,609	##########	-€ 7,331,355	-€ 14,662,711	-€ 21,994,066	-€ 29,325,422
1415	0.22	€ 346,048	€ 692,096	##########	##########	-€ 8,367,342	-€ 16,734,684	-€ 25,102,026	-€ 33,469,369
1416	0.20	€ 309,590	€ 619,180	€ 928,770	##########	-€ 7,485,794	-€ 14,971,589	-€ 22,457,383	-€ 29,943,178
1417	0.19	€ 293,268	€ 586,535	€ 879,803	##########	-€ 7,091,121	-€ 14,182,242	-€ 21,273,363	-€ 28,364,485
1418	0.19	€ 300,101	€ 600,203	€ 900,304	##########	-€ 7,256,363	-€ 14,512,726	-€ 21,769,090	-€ 29,025,453
1419	0.16	€ 249,321	€ 498,642	€ 747,963	€ 997,284	-€ 6,028,506	-€ 12,057,013	-€ 18,085,519	-€ 24,114,025
1420	0.20	€ 316,963	€ 633,927	€ 950,890	##########	-€ 7,664,082	-€ 15,328,165	-€ 22,992,247	-€ 30,656,330
1421	0.18	€ 278,185	€ 556,369	€ 834,554	##########	-€ 6,726,421	-€ 13,452,842	-€ 20,179,264	-€ 26,905,685
1422	0.21	€ 330,834	€ 661,668	€ 992,502	##########	-€ 7,999,467	-€ 15,998,935	-€ 23,998,402	-€ 31,997,869
1423	0.16	€ 246,770	€ 493,540	€ 740,310	€ 987,080	-€ 5,966,827	-€ 11,933,653	-€ 17,900,480	-€ 23,867,306
1424	0.14	€ 223,510	€ 447,020	€ 670,530	€ 894,040	-€ 5,404,403	-€ 10,808,806	-€ 16,213,210	-€ 21,617,613

1425	0.17	€ 268,139	€ 536,278	€ 804,417	##########	-€ 6,483,522	-€ 12,967,044	-€ 19,450,566	-€ 25,934,088
1426	0.16	€ 256,635	€ 513,270	€ 769,905	##########	-€ 6,205,359	-€ 12,410,719	-€ 18,616,078	-€ 24,821,438
1427	0.17	€ 261,660	€ 523,321	€ 784,981	##########	-€ 6,326,873	-€ 12,653,746	-€ 18,980,619	-€ 25,307,492
1428	0.24	€ 382,746	€ 765,491	##########	##########	-€ 9,254,674	-€ 18,509,349	-€ 27,764,023	-€ 37,018,698
1429	0.17	€ 268,381	€ 536,762	€ 805,143	##########	-€ 6,489,374	-€ 12,978,748	-€ 19,468,121	-€ 25,957,495
1430	0.18	€ 285,985	€ 571,970	€ 857,956	##########	-€ 6,915,038	-€ 13,830,076	-€ 20,745,114	-€ 27,660,151
1431	0.24	€ 370,206	€ 740,413	##########	##########	-€ 8,951,479	-€ 17,902,959	-€ 26,854,438	-€ 35,805,918
1432	0.25	€ 394,683	€ 789,366	##########	##########	-€ 9,543,318	-€ 19,086,636	-€ 28,629,954	-€ 38,173,272
1433	0.20	€ 313,850	€ 627,700	€ 941,550	##########	-€ 7,588,797	-€ 15,177,594	-€ 22,766,391	-€ 30,355,189
1434	0.18	€ 290,853	€ 581,707	€ 872,560	##########	-€ 7,032,745	-€ 14,065,491	-€ 21,098,236	-€ 28,130,981
1435	0.18	€ 280,340	€ 560,679	€ 841,019	##########	-€ 6,778,532	-€ 13,557,063	-€ 20,335,595	-€ 27,114,127
1436	0.15	€ 232,647	€ 465,295	€ 697,942	€ 930,589	-€ 5,625,343	-€ 11,250,686	-€ 16,876,030	-€ 22,501,373
1437	0.13	€ 211,778	€ 423,556	€ 635,334	€ 847,111	-€ 5,120,726	-€ 10,241,452	-€ 15,362,178	-€ 20,482,904
1438	0.16	€ 254,857	€ 509,714	€ 764,571	##########	-€ 6,162,367	-€ 12,324,735	-€ 18,487,102	-€ 24,649,470
1439	0.15	€ 238,600	€ 477,201	€ 715,801	€ 954,401	-€ 5,769,286	-€ 11,538,572	-€ 17,307,858	-€ 23,077,144
1440	0.18	€ 280,186	€ 560,373	€ 840,559	##########	-€ 6,774,820	-€ 13,549,641	-€ 20,324,461	-€ 27,099,282
1441	0.15	€ 242,019	€ 484,037	€ 726,056	€ 968,075	-€ 5,851,941	-€ 11,703,882	-€ 17,555,824	-€ 23,407,765
1442	0.20	€ 308,461	€ 616,922	€ 925,383	##########	-€ 7,458,493	-€ 14,916,986	-€ 22,375,478	-€ 29,833,971
1443	0.18	€ 287,093	€ 574,186	€ 861,279	##########	-€ 6,941,823	-€ 13,883,647	-€ 20,825,470	-€ 27,767,294
1444	0.25	€ 399,815	€ 799,630	##########	##########	-€ 9,667,404	-€ 19,334,808	-€ 29,002,212	-€ 38,669,616
1445	0.12	€ 193,052	€ 386,104	€ 579,156	€ 772,208	-€ 4,667,940	-€ 9,335,879	-€ 14,003,819	-€ 18,671,758
1446	0.16	€ 246,494	€ 492,987	€ 739,481	€ 985,975	-€ 5,960,143	-€ 11,920,287	-€ 17,880,430	-€ 23,840,574
1447	0.16	€ 247,993	€ 495,985	€ 743,978	€ 991,970	-€ 5,996,387	-€ 11,992,774	-€ 17,989,160	-€ 23,985,547
1448	0.26	€ 403,866	€ 807,731	##########	##########	-€ 9,765,349	-€ 19,530,699	-€ 29,296,048	-€ 39,061,397
1449	0.22	€ 342,657	€ 685,313	##########	##########	-€ 8,285,336	-€ 16,570,672	-€ 24,856,007	-€ 33,141,343
1450	0.19	€ 304,213	€ 608,427	€ 912,640	##########	-€ 7,355,787	-€ 14,711,573	-€ 22,067,360	-€ 29,423,147
1451	0.17	€ 271,453	€ 542,906	€ 814,359	##########	-€ 6,563,652	-€ 13,127,304	-€ 19,690,956	-€ 26,254,609
1452	0.24	€ 370,078	€ 740,155	##########	##########	-€ 8,948,368	-€ 17,896,736	-€ 26,845,104	-€ 35,793,471
1453	0.27	€ 426,072	€ 852,145	##########	##########	-€ 10,302,306	-€ 20,604,613	-€ 30,906,919	-€ 41,209,225
1454	0.20	€ 309,806	€ 619,613	€ 929,419	##########	-€ 7,491,024	-€ 14,982,048	-€ 22,473,072	-€ 29,964,095
1455	0.21	€ 332,898	€ 665,796	€ 998,694	##########	-€ 8,049,374	-€ 16,098,748	-€ 24,148,122	-€ 32,197,496
1456	0.16	€ 245,138	€ 490,275	€ 735,413	€ 980,551	-€ 5,927,356	-€ 11,854,713	-€ 17,782,069	-€ 23,709,425
1457	0.20	€ 322,009	€ 644,019	€ 966,028	##########	-€ 7,786,092	-€ 15,572,184	-€ 23,358,276	-€ 31,144,369
1458	0.26	€ 416,218	€ 832,437	##########	##########	-€ 10,064,036	-€ 20,128,073	-€ 30,192,109	-€ 40,256,146
1459	0.17	€ 264,851	€ 529,702	€ 794,553	##########	-€ 6,404,019	-€ 12,808,038	-€ 19,212,057	-€ 25,616,076
1460	0.18	€ 285,931	€ 571,862	€ 857,794	##########	-€ 6,913,732	-€ 13,827,464	-€ 20,741,196	-€ 27,654,928
1461	0.20	€ 318,838	€ 637,676	€ 956,514	##########	-€ 7,709,406	-€ 15,418,813	-€ 23,128,219	-€ 30,837,625
1462	0.19	€ 301,801	€ 603,602	€ 905,403	##########	-€ 7,297,460	-€ 14,594,919	-€ 21,892,379	-€ 29,189,838
1463	0.21	€ 332,015	€ 664,030	€ 996,045	##########	-€ 8,028,020	-€ 16,056,041	-€ 24,084,061	-€ 32,112,082
1464	0.16	€ 247,252	€ 494,504	€ 741,756	€ 989,007	-€ 5,978,477	-€ 11,956,953	-€ 17,935,430	-€ 23,913,906
1465	0.17	€ 260,021	€ 520,042	€ 780,063	##########	-€ 6,287,233	-€ 12,574,466	-€ 18,861,700	-€ 25,148,933
1466	0.21	€ 329,061	€ 658,123	€ 987,184	##########	-€ 7,956,608	-€ 15,913,217	-€ 23,869,825	-€ 31,826,434
1467	0.22	€ 340,364	€ 680,729	##########	##########	-€ 8,229,910	-€ 16,459,820	-€ 24,689,730	-€ 32,919,639
1468	0.18	€ 287,199	€ 574,398	€ 861,598	##########	-€ 6,944,392	-€ 13,888,785	-€ 20,833,177	-€ 27,777,570
1469	0.25	€ 401,398	€ 802,796	##########	##########	-€ 9,705,688	-€ 19,411,376	-€ 29,117,064	-€ 38,822,752
1470	0.22	€ 347,031	€ 694,062	##########	##########	-€ 8,391,110	-€ 16,782,221	-€ 25,173,331	-€ 33,564,441
1471	0.26	€ 407,220	€ 814,440	##########	##########	-€ 9,846,459	-€ 19,692,917	-€ 29,539,376	-€ 39,385,835
1472	0.25	€ 391,058	€ 782,116	##########	##########	-€ 9,455,662	-€ 18,911,325	-€ 28,366,987	-€ 37,822,650
1473	0.17	€ 266,880	€ 533,760	€ 800,640	##########	-€ 6,453,075	-€ 12,906,151	-€ 19,359,226	-€ 25,812,302
1474	0.19	€ 299,722	€ 599,445	€ 899,167	##########	-€ 7,247,199	-€ 14,494,398	-€ 21,741,597	-€ 28,988,797
1475	0.19	€ 295,139	€ 590,279	€ 885,418	##########	-€ 7,136,382	-€ 14,272,764	-€ 21,409,145	-€ 28,545,527
1476	0.22	€ 344,787	€ 689,574	##########	##########	-€ 8,336,844	-€ 16,673,689	-€ 25,010,533	-€ 33,347,377
1477	0.24	€ 374,111	€ 748,222	##########	##########	-€ 9,045,890	-€ 18,091,779	-€ 27,137,669	-€ 36,183,558
1478	0.25	€ 393,888	€ 787,776	##########	##########	-€ 9,524,092	-€ 19,048,183	-€ 28,572,275	-€ 38,096,367
1479	0.21	€ 330,913	€ 661,826	€ 992,739	##########	-€ 8,001,380	-€ 16,002,761	-€ 24,004,141	-€ 32,005,522
1480	0.22	€ 340,577	€ 681,154	##########	##########	-€ 8,235,050	-€ 16,470,100	-€ 24,705,150	-€ 32,940,200
1481	0.21	€ 338,008	€ 676,017	##########	##########	-€ 8,172,944	-€ 16,345,888	-€ 24,518,832	-€ 32,691,776
1482	0.16	€ 244,849	€ 489,698	€ 734,547	€ 979,396	-€ 5,920,377	-€ 11,840,753	-€ 17,761,130	-€ 23,681,507
1483	0.15	€ 233,226	€ 466,451	€ 699,677	€ 932,902	-€ 5,639,324	-€ 11,278,649	-€ 16,917,973	-€ 22,557,298
1484	0.23	€ 365,306	€ 730,611	##########	##########	-€ 8,832,984	-€ 17,665,967	-€ 26,498,951	-€ 35,331,935
1485	0.23	€ 366,835	€ 733,670	##########	##########	-€ 8,869,965	-€ 17,739,931	-€ 26,609,896	-€ 35,479,861
1486	0.15	€ 235,431	€ 470,863	€ 706,294	€ 941,726	-€ 5,692,663	-€ 11,385,326	-€ 17,077,989	-€ 22,770,652
1487	0.21	€ 327,414	€ 654,828	€ 982,243	##########	-€ 7,916,779	-€ 15,833,557	-€ 23,750,336	-€ 31,667,114
1488	0.23	€ 366,831	€ 733,662	##########	##########	-€ 8,869,870	-€ 17,739,740	-€ 26,609,610	-€ 35,479,480
1489	0.22	€ 351,407	€ 702,814	##########	##########	-€ 8,496,922	-€ 16,993,844	-€ 25,490,765	-€ 33,987,687
1490	0.17	€ 266,406	€ 532,812	€ 799,218	##########	-€ 6,441,618	-€ 12,883,237	-€ 19,324,855	-€ 25,766,473
1491	0.17	€ 265,043	€ 530,086	€ 795,129	##########	-€ 6,408,659	-€ 12,817,318	-€ 19,225,978	-€ 25,634,637
1492	0.21	€ 336,359	€ 672,718	##########	##########	-€ 8,133,060	-€ 16,266,120	-€ 24,399,179	-€ 32,532,239
1493	0.22	€ 344,572	€ 689,143	##########	##########	-€ 8,331,640	-€ 16,663,281	-€ 24,994,921	-€ 33,326,562
1494	0.24	€ 381,862	€ 763,725	##########	##########	-€ 9,233,320	-€ 18,466,641	-€ 27,699,961	-€ 36,933,281
1495	0.16	€ 254,876	€ 509,753	€ 764,629	##########	-€ 6,162,838	-€ 12,325,675	-€ 18,488,513	-€ 24,651,351
1496	0.22	€ 350,570	€ 701,140	##########	##########	-€ 8,476,675	-€ 16,953,349	-€ 25,430,024	-€ 33,906,698
1497	0.24	€ 372,517	€ 745,035	##########	##########	-€ 9,007,361	-€ 18,014,722	-€ 27,022,084	-€ 36,029,445
1498	0.21	€ 325,304	€ 650,609	€ 975,913	##########	-€ 7,865,762	-€ 15,731,524	-€ 23,597,286	-€ 31,463,048
1499	0.17	€ 269,478	€ 538,957	€ 808,435	##########	-€ 6,515,905	-€ 13,031,809	-€ 19,547,714	-€ 26,063,619
1500	0.17	€ 266,274	€ 532,548	€ 798,822	##########	-€ 6,438,429	-€ 12,876,857	-€ 19,315,286	-€ 25,753,714
1501	0.21	€ 325,362	€ 650,725	€ 976,087	##########	-€ 7,867,165	-€ 15,734,330	-€ 23,601,495	-€ 31,468,660
1502	0.21	€ 337,321	€ 674,641	##########	##########	-€ 8,156,312	-€ 16,312,623	-€ 24,468,935	-€ 32,625,247
1503	0.20	€ 316,320	€ 632,640	€ 948,961	##########	-€ 7,648,528	-€ 15,297,056	-€ 22,945,584	-€ 30,594,112
1504	0.20	€ 308,121	€ 616,242	€ 924,363	##########	-€ 7,450,276	-€ 14,900,551	-€ 22,350,827	-€ 29,801,103
1505	0.20	€ 310,971	€ 621,943	€ 932,914	##########	-€ 7,519,197	-€ 15,038,393	-€ 22,557,590	-€ 30,076,786
1506	0.20	€ 311,116	€ 622,232	€ 933,349	##########	-€ 7,522,698	-€ 15,045,395	-€ 22,568,093	-€ 30,090,790
1507	0.18	€ 284,346	€ 568,692	€ 853,038	##########	-€ 6,875,401	-€ 13,750,801	-€ 20,626,202	-€ 27,501,603
1508	0.17	€ 269,271	€ 538,541	€ 807,812	##########	-€ 6,510,882	-€ 13,021,763	-€ 19,532,645	-€ 26,043,527
1509	0.21	€ 326,074	€ 652,148	€ 978,222	##########	-€ 7,884,373	-€ 15,768,746	-€ 23,653,119	-€ 31,537,492
1510	0.18	€ 284,428	€ 568,856	€ 853,283	##########	-€ 6,877,379	-€ 13,754,759	-€ 20,632,138	-€ 27,509,518
1511	0.21	€ 333,947	€ 667,894	##########	##########	-€ 8,074,735	-€ 16,149,469	-€ 24,224,204	-€ 32,298,939
1512	0.12	€ 196,482	€ 392,963	€ 589,445	€ 785,926	-€ 4,750,865	-€ 9,501,730	-€ 14,252,595	-€ 19,003,460
1513	0.20	€ 317,274	€ 634,548	€ 951,821	##########	-€ 7,671,586	-€ 15,343,173	-€ 23,014,759	-€ 30,686,345
1514	0.13	€ 207,094	€ 414,187	€ 621,281	€ 828,375	-€ 5,007,463	-€ 10,014,925	-€ 15,022,388	-€ 20,029,851
1515	0.19	€ 300,470	€ 600,940	€ 901,409	##########	-€ 7,265,270	-€ 14,530,541	-€ 21,795,811	-€ 29,061,081
1516	0.19	€ 294,964	€ 589,927	€ 884,891	##########	-€ 7,132,133	-€ 14,264,266	-€ 21,396,398	-€ 28,528,531
1517	0.15	€ 229,818	€ 459,636	€ 689,453	€ 919,271	-€ 5,556,926	-€ 11,113,853	-€ 16,670,779	-€ 22,227,705
1518	0.20	€ 317,637	€ 635,275	€ 952,912	##########	-€ 7,680,375	-€ 15,360,750	-€ 23,041,125	-€ 30,721,499
1519	0.16	€ 259,755	€ 519,510	€ 779,265	##########	-€ 6,280,801	-€ 12,561,602	-€ 18,842,403	-€ 25,123,204
1520	0.17	€ 274,185	€ 548,370	€ 822,555	##########	-€ 6,629,711	-€ 13,259,422	-€ 19,889,133	-€ 26,518,845
1521	0.19	€ 305,700	€ 611,399	€ 917,099	##########	-€ 7,391,726	-€ 14,783,451	-€ 22,175,177	-€ 29,566,903
1522	0.21	€ 333,074	€ 666,148	€ 999,222	##########	-€ 8,053,629	-€ 16,107,259	-€ 24,160,888	-€ 32,214,517
1523	0.20	€ 321,498	€ 642,996	€ 964,493	##########	-€ 7,773,721	-€ 15,547,442	-€ 23,321,163	-€ 31,094,884
1524	0.20	€ 315,887	€ 631,774	€ 947,660	##########	-€ 7,638,049	-€ 15,276,097	-€ 22,914,146	-€ 30,552,195
1525	0.14	€ 223,673	€ 447,345	€ 671,018	€ 894,690	-€ 5,408,337	-€ 10,816,674	-€ 16,225,010	-€ 21,633,347
1526	0.24	€ 371,063	€ 742,126	##########	##########	-€ 8,972,189	-€ 17,944,378	-€ 26,916,566	-€ 35,888,755

1527	0.18	€ 281,925	€ 563,850	€ 845,774	##########	-€ 6,816,859	-€ 13,633,718	-€ 20,450,576	-€ 27,267,435
1528	0.19	€ 295,502	€ 591,004	€ 886,507	##########	-€ 7,145,156	-€ 14,290,312	-€ 21,435,467	-€ 28,580,623
1529	0.14	€ 214,425	€ 428,850	€ 643,275	€ 857,700	-€ 5,184,734	-€ 10,369,467	-€ 15,554,201	-€ 20,738,935
1530	0.21	€ 336,831	€ 673,661	##########	##########	-€ 8,144,468	-€ 16,288,935	-€ 24,433,403	-€ 32,577,870
1531	0.17	€ 263,991	€ 527,981	€ 791,972	##########	-€ 6,383,217	-€ 12,766,434	-€ 19,149,651	-€ 25,532,867
1532	0.17	€ 261,995	€ 523,989	€ 785,984	##########	-€ 6,334,952	-€ 12,669,905	-€ 19,004,857	-€ 25,339,810
1533	0.15	€ 239,274	€ 478,548	€ 717,823	€ 957,097	-€ 5,785,579	-€ 11,571,158	-€ 17,356,737	-€ 23,142,316
1534	0.13	€ 210,153	€ 420,306	€ 630,459	€ 840,612	-€ 5,081,439	-€ 10,162,878	-€ 15,244,317	-€ 20,325,756
1535	0.22	€ 349,805	€ 699,610	##########	##########	-€ 8,458,179	-€ 16,916,358	-€ 25,374,537	-€ 33,832,716
1536	0.15	€ 236,090	€ 472,179	€ 708,269	€ 944,358	-€ 5,708,575	-€ 11,417,151	-€ 17,125,726	-€ 22,834,302
1537	0.23	€ 363,093	€ 726,186	##########	##########	-€ 8,779,479	-€ 17,558,959	-€ 26,338,438	-€ 35,117,917
1538	0.13	€ 211,778	€ 423,556	€ 635,335	€ 847,113	-€ 5,120,734	-€ 10,241,468	-€ 15,362,202	-€ 20,482,936
1539	0.16	€ 250,125	€ 500,250	€ 750,375	##########	-€ 6,047,951	-€ 12,095,902	-€ 18,143,852	-€ 24,191,803
1540	0.19	€ 294,513	€ 589,026	€ 883,539	##########	-€ 7,121,235	-€ 14,242,471	-€ 21,363,706	-€ 28,484,942
1541	0.23	€ 356,108	€ 712,215	##########	##########	-€ 8,610,576	-€ 17,221,152	-€ 25,831,728	-€ 34,442,303
1542	0.17	€ 270,227	€ 540,454	€ 810,681	##########	-€ 6,534,008	-€ 13,068,016	-€ 19,602,024	-€ 26,136,032
1543	0.17	€ 260,279	€ 520,558	€ 780,837	##########	-€ 6,293,466	-€ 12,586,933	-€ 18,880,399	-€ 25,173,866
1544	0.22	€ 340,265	€ 680,529	##########	##########	-€ 8,227,496	-€ 16,454,993	-€ 24,682,489	-€ 32,909,986
1545	0.20	€ 308,993	€ 617,985	€ 926,978	##########	-€ 7,471,352	-€ 14,942,704	-€ 22,414,056	-€ 29,885,408
1546	0.19	€ 300,396	€ 600,793	€ 901,189	##########	-€ 7,263,494	-€ 14,526,987	-€ 21,790,481	-€ 29,053,975
1547	0.18	€ 287,434	€ 574,867	€ 862,301	##########	-€ 6,950,058	-€ 13,900,115	-€ 20,850,173	-€ 27,800,231
1548	0.21	€ 327,355	€ 654,709	€ 982,064	##########	-€ 7,915,339	-€ 15,830,678	-€ 23,746,017	-€ 31,661,356
1549	0.16	€ 254,483	€ 508,966	€ 763,449	##########	-€ 6,153,320	-€ 12,306,640	-€ 18,459,961	-€ 24,613,281
1550	0.26	€ 411,314	€ 822,628	##########	##########	-€ 9,945,454	-€ 19,890,908	-€ 29,836,362	-€ 39,781,816
1551	0.23	€ 366,528	€ 733,056	##########	##########	-€ 8,862,541	-€ 17,725,081	-€ 26,587,622	-€ 35,450,162
1552	0.24	€ 374,317	€ 748,634	##########	##########	-€ 9,050,877	-€ 18,101,754	-€ 27,152,631	-€ 36,203,507
1553	0.16	€ 253,059	€ 506,118	€ 759,177	##########	-€ 6,118,893	-€ 12,237,785	-€ 18,356,678	-€ 24,475,570
1554	0.21	€ 331,674	€ 663,349	€ 995,023	##########	-€ 8,019,790	-€ 16,039,580	-€ 24,059,370	-€ 32,079,160
1555	0.11	€ 175,380	€ 350,760	€ 526,140	€ 701,520	-€ 4,240,636	-€ 8,481,271	-€ 12,721,907	-€ 16,962,543
1556	0.17	€ 264,881	€ 529,763	€ 794,644	##########	-€ 6,404,751	-€ 12,809,502	-€ 19,214,253	-€ 25,619,004
1557	0.21	€ 327,203	€ 654,406	€ 981,608	##########	-€ 7,911,667	-€ 15,823,335	-€ 23,735,002	-€ 31,646,669
1558	0.23	€ 357,807	€ 715,615	##########	##########	-€ 8,651,675	-€ 17,303,350	-€ 25,955,025	-€ 34,606,700
1559	0.15	€ 238,323	€ 476,646	€ 714,969	€ 953,293	-€ 5,762,582	-€ 11,525,165	-€ 17,287,747	-€ 23,050,330
1560	0.19	€ 299,852	€ 599,705	€ 899,557	##########	-€ 7,250,339	-€ 14,500,678	-€ 21,751,017	-€ 29,001,356
1561	0.24	€ 374,820	€ 749,640	##########	##########	-€ 9,063,031	-€ 18,126,062	-€ 27,189,094	-€ 36,252,125
1562	0.21	€ 337,792	€ 675,585	##########	##########	-€ 8,167,720	-€ 16,335,441	-€ 24,503,161	-€ 32,670,882
1563	0.15	€ 231,547	€ 463,094	€ 694,641	€ 926,187	-€ 5,598,734	-€ 11,197,468	-€ 16,796,203	-€ 22,394,937
1564	0.25	€ 397,075	€ 794,150	##########	##########	-€ 9,601,160	-€ 19,202,321	-€ 28,803,481	-€ 38,404,641
1565	0.15	€ 233,813	€ 467,627	€ 701,440	€ 935,253	-€ 5,653,537	-€ 11,307,073	-€ 16,960,610	-€ 22,614,146
1566	0.21	€ 323,165	€ 646,330	€ 969,495	##########	-€ 7,814,034	-€ 15,628,069	-€ 23,442,103	-€ 31,256,137
1567	0.18	€ 286,849	€ 573,697	€ 860,546	##########	-€ 6,935,914	-€ 13,871,827	-€ 20,807,741	-€ 27,743,655
1568	0.15	€ 230,212	€ 460,424	€ 690,636	€ 920,847	-€ 5,566,454	-€ 11,132,909	-€ 16,699,363	-€ 22,265,817
1569	0.12	€ 181,559	€ 363,118	€ 544,677	€ 726,236	-€ 4,390,044	-€ 8,780,088	-€ 13,170,132	-€ 17,560,176
1570	0.25	€ 399,854	€ 799,708	##########	##########	-€ 9,668,350	-€ 19,336,700	-€ 29,005,050	-€ 38,673,401
1571	0.18	€ 282,924	€ 565,848	€ 848,772	##########	-€ 6,841,016	-€ 13,682,032	-€ 20,523,049	-€ 27,364,065
1572	0.13	€ 197,096	€ 394,193	€ 591,289	€ 788,386	-€ 4,765,734	-€ 9,531,469	-€ 14,297,203	-€ 19,062,938
1573	0.22	€ 340,150	€ 680,299	##########	##########	-€ 8,224,717	-€ 16,449,434	-€ 24,674,150	-€ 32,898,867
1574	0.16	€ 255,878	€ 511,756	€ 767,634	##########	-€ 6,187,056	-€ 12,374,111	-€ 18,561,167	-€ 24,748,222
1575	0.30	€ 466,571	€ 933,143	##########	##########	-€ 11,281,558	-€ 22,563,117	-€ 33,844,675	-€ 45,126,233
1576	0.14	€ 213,217	€ 426,433	€ 639,650	€ 852,867	-€ 5,155,516	-€ 10,311,031	-€ 15,466,547	-€ 20,622,062
1577	0.17	€ 274,179	€ 548,358	€ 822,536	##########	-€ 6,629,561	-€ 13,259,122	-€ 19,888,684	-€ 26,518,245
1578	0.21	€ 329,843	€ 659,686	€ 989,529	##########	-€ 7,975,505	-€ 15,951,010	-€ 23,926,515	-€ 31,902,020
1579	0.18	€ 285,528	€ 571,057	€ 856,585	##########	-€ 6,903,990	-€ 13,807,980	-€ 20,711,970	-€ 27,615,960
1580	0.12	€ 190,383	€ 380,765	€ 571,148	€ 761,530	-€ 4,603,394	-€ 9,206,787	-€ 13,810,181	-€ 18,413,575
1581	0.15	€ 236,826	€ 473,652	€ 710,478	€ 947,305	-€ 5,726,386	-€ 11,452,772	-€ 17,179,159	-€ 22,905,545
1582	0.17	€ 272,292	€ 544,583	€ 816,875	##########	-€ 6,583,928	-€ 13,167,856	-€ 19,751,783	-€ 26,335,711
1583	0.23	€ 354,666	€ 709,332	##########	##########	-€ 8,575,719	-€ 17,151,439	-€ 25,727,158	-€ 34,302,877
1584	0.24	€ 377,774	€ 755,548	##########	##########	-€ 9,134,457	-€ 18,268,915	-€ 27,403,372	-€ 36,537,830
1585	0.16	€ 253,887	€ 507,773	€ 761,660	##########	-€ 6,138,902	-€ 12,277,804	-€ 18,416,706	-€ 24,555,608
1586	0.19	€ 298,830	€ 597,660	€ 896,490	##########	-€ 7,225,621	-€ 14,451,242	-€ 21,676,863	-€ 28,902,484
1587	0.21	€ 337,462	€ 674,923	##########	##########	-€ 8,159,720	-€ 16,319,441	-€ 24,479,161	-€ 32,638,882
1588	0.17	€ 272,802	€ 545,603	€ 818,405	##########	-€ 6,596,262	-€ 13,192,524	-€ 19,788,786	-€ 26,385,049
1589	0.12	€ 188,040	€ 376,080	€ 564,119	€ 752,159	-€ 4,546,746	-€ 9,093,492	-€ 13,640,238	-€ 18,186,984
1590	0.12	€ 195,940	€ 391,880	€ 587,820	€ 783,761	-€ 4,737,775	-€ 9,475,550	-€ 14,213,325	-€ 18,951,100
1591	0.24	€ 380,872	€ 761,745	##########	##########	-€ 9,209,380	-€ 18,418,761	-€ 27,628,141	-€ 36,837,522
1592	0.18	€ 279,172	€ 558,344	€ 837,516	##########	-€ 6,750,293	-€ 13,500,586	-€ 20,250,878	-€ 27,001,171
1593	0.24	€ 377,962	€ 755,923	##########	##########	-€ 9,139,001	-€ 18,278,003	-€ 27,417,004	-€ 36,556,005
1594	0.16	€ 248,919	€ 497,839	€ 746,758	€ 995,677	-€ 6,018,796	-€ 12,037,592	-€ 18,056,388	-€ 24,075,184
1595	0.22	€ 342,103	€ 684,206	##########	##########	-€ 8,271,952	-€ 16,543,904	-€ 24,815,857	-€ 33,087,809
1596	0.23	€ 358,913	€ 717,825	##########	##########	-€ 8,678,398	-€ 17,356,796	-€ 26,035,194	-€ 34,713,592
1597	0.15	€ 240,195	€ 480,389	€ 720,584	€ 960,778	-€ 5,807,835	-€ 11,615,669	-€ 17,423,504	-€ 23,231,338
1598	0.17	€ 261,167	€ 522,334	€ 783,501	##########	-€ 6,314,937	-€ 12,629,873	-€ 18,944,810	-€ 25,259,746
1599	0.25	€ 386,553	€ 773,107	##########	##########	-€ 9,346,748	-€ 18,693,496	-€ 28,040,244	-€ 37,386,993
1600	0.19	€ 300,927	€ 601,854	€ 902,782	##########	-€ 7,276,330	-€ 14,552,660	-€ 21,828,990	-€ 29,105,320
1601	0.24	€ 376,377	€ 752,753	##########	##########	-€ 9,100,676	-€ 18,201,351	-€ 27,302,027	-€ 36,402,703
1602	0.25	€ 388,216	€ 776,432	##########	##########	-€ 9,386,949	-€ 18,773,898	-€ 28,160,848	-€ 37,547,797
1603	0.20	€ 312,975	€ 625,950	€ 938,926	##########	-€ 7,567,647	-€ 15,135,295	-€ 22,702,942	-€ 30,270,590
1604	0.16	€ 245,392	€ 490,785	€ 736,177	€ 981,570	-€ 5,933,517	-€ 11,867,034	-€ 17,800,552	-€ 23,734,069
1605	0.17	€ 266,713	€ 533,425	€ 800,138	##########	-€ 6,449,035	-€ 12,898,070	-€ 19,347,104	-€ 25,796,139
1606	0.16	€ 248,281	€ 496,562	€ 744,844	€ 993,125	-€ 6,003,365	-€ 12,006,730	-€ 18,010,095	-€ 24,013,460
1607	0.23	€ 366,470	€ 732,940	##########	##########	-€ 8,861,141	-€ 17,722,282	-€ 26,583,423	-€ 35,444,564
1608	0.25	€ 398,269	€ 796,538	##########	##########	-€ 9,630,022	-€ 19,260,044	-€ 28,890,066	-€ 38,520,089
1609	0.20	€ 307,818	€ 615,636	€ 923,454	##########	-€ 7,442,948	-€ 14,885,897	-€ 22,328,845	-€ 29,771,794
1610	0.16	€ 244,280	€ 488,560	€ 732,840	€ 977,120	-€ 5,906,620	-€ 11,813,241	-€ 17,719,861	-€ 23,626,481
1611	0.20	€ 315,566	€ 631,131	€ 946,697	##########	-€ 7,630,281	-€ 15,260,561	-€ 22,890,842	-€ 30,521,122
1612	0.20	€ 312,883	€ 625,766	€ 938,649	##########	-€ 7,565,420	-€ 15,130,840	-€ 22,696,260	-€ 30,261,679
1613	0.18	€ 291,099	€ 582,198	€ 873,298	##########	-€ 7,038,693	-€ 14,077,385	-€ 21,116,078	-€ 28,154,771
1614	0.16	€ 256,009	€ 512,018	€ 768,027	##########	-€ 6,190,226	-€ 12,380,451	-€ 18,570,677	-€ 24,760,902
1615	0.20	€ 307,261	€ 614,522	€ 921,783	##########	-€ 7,429,482	-€ 14,858,963	-€ 22,288,445	-€ 29,717,927
1616	0.26	€ 415,689	€ 831,377	##########	##########	-€ 10,051,227	-€ 20,102,454	-€ 30,153,680	-€ 40,204,907
1617	0.12	€ 185,129	€ 370,258	€ 555,387	€ 740,517	-€ 4,476,368	-€ 8,952,736	-€ 13,429,105	-€ 17,905,473
1618	0.16	€ 250,570	€ 501,140	€ 751,711	##########	-€ 6,058,714	-€ 12,117,428	-€ 18,176,142	-€ 24,234,856
1619	0.17	€ 269,918	€ 539,836	€ 809,754	##########	-€ 6,526,534	-€ 13,053,067	-€ 19,579,601	-€ 26,106,135
1620	0.17	€ 267,688	€ 535,377	€ 803,065	##########	-€ 6,472,622	-€ 12,945,245	-€ 19,417,867	-€ 25,890,490
1621	0.20	€ 316,326	€ 632,652	€ 948,979	##########	-€ 7,648,674	-€ 15,297,347	-€ 22,946,021	-€ 30,594,694
1622	0.11	€ 178,912	€ 357,824	€ 536,736	€ 715,647	-€ 4,326,035	-€ 8,652,071	-€ 12,978,106	-€ 17,304,142
1623	0.20	€ 307,500	€ 615,000	€ 922,500	##########	-€ 7,435,261	-€ 14,870,523	-€ 22,305,784	-€ 29,741,045
1624	0.12	€ 193,825	€ 387,650	€ 581,475	€ 775,300	-€ 4,686,628	-€ 9,373,257	-€ 14,059,885	-€ 18,746,514
1625	0.22	€ 344,028	€ 688,057	##########	##########	-€ 8,318,505	-€ 16,637,010	-€ 24,955,515	-€ 33,274,020
1626	0.22	€ 341,915	€ 683,830	##########	##########	-€ 8,267,406	-€ 16,534,812	-€ 24,802,217	-€ 33,069,623
1627	0.17	€ 262,306	€ 524,612	€ 786,918	##########	-€ 6,342,484	-€ 12,684,967	-€ 19,027,451	-€ 25,369,935
1628	0.22	€ 338,990	€ 677,979	##########	##########	-€ 8,196,670	-€ 16,393,340	-€ 24,590,010	-€ 32,786,680

1629	0.21	€ 331,530	€ 663,060	€ 994,590	##########	-€ 8,016,297	-€ 16,032,594	-€ 24,048,891	-€ 32,065,188
1630	0.26	€ 404,537	€ 809,074	##########	##########	-€ 9,781,580	-€ 19,563,160	-€ 29,344,740	-€ 39,126,320
1631	0.13	€ 212,309	€ 424,618	€ 636,927	€ 849,236	-€ 5,133,570	-€ 10,267,139	-€ 15,400,709	-€ 20,534,278
1632	0.18	€ 278,204	€ 556,409	€ 834,613	##########	-€ 6,726,896	-€ 13,453,793	-€ 20,180,689	-€ 26,907,585
1633	0.26	€ 407,442	€ 814,884	##########	##########	-€ 9,851,823	-€ 19,703,645	-€ 29,555,468	-€ 39,407,291
1634	0.15	€ 231,119	€ 462,238	€ 693,356	€ 924,475	-€ 5,588,383	-€ 11,176,766	-€ 16,765,150	-€ 22,353,533
1635	0.19	€ 299,958	€ 599,916	€ 899,874	##########	-€ 7,252,894	-€ 14,505,787	-€ 21,758,681	-€ 29,011,574
1636	0.16	€ 244,288	€ 488,575	€ 732,863	€ 977,150	-€ 5,906,801	-€ 11,813,602	-€ 17,720,403	-€ 23,627,205
1637	0.22	€ 344,363	€ 688,727	##########	##########	-€ 8,326,604	-€ 16,653,208	-€ 24,979,812	-€ 33,306,417
1638	0.11	€ 170,209	€ 340,419	€ 510,628	€ 680,838	-€ 4,115,615	-€ 8,231,230	-€ 12,346,844	-€ 16,462,459
1639	0.17	€ 271,804	€ 543,608	€ 815,412	##########	-€ 6,572,138	-€ 13,144,275	-€ 19,716,413	-€ 26,288,550
1640	0.24	€ 384,385	€ 768,770	##########	##########	-€ 9,294,310	-€ 18,588,620	-€ 27,882,930	-€ 37,177,240
1641	0.18	€ 276,052	€ 552,104	€ 828,156	##########	-€ 6,674,859	-€ 13,349,718	-€ 20,024,577	-€ 26,699,436
1642	0.21	€ 330,401	€ 660,802	€ 991,203	##########	-€ 7,988,997	-€ 15,977,994	-€ 23,966,991	-€ 31,955,988
1643	0.16	€ 253,346	€ 506,692	€ 760,038	##########	-€ 6,125,830	-€ 12,251,661	-€ 18,377,491	-€ 24,503,321
1644	0.29	€ 449,247	€ 898,493	##########	##########	-€ 10,862,649	-€ 21,725,297	-€ 32,587,946	-€ 43,450,595
1645	0.22	€ 340,554	€ 681,107	##########	##########	-€ 8,234,485	-€ 16,468,970	-€ 24,703,455	-€ 32,937,940
1646	0.24	€ 384,741	€ 769,483	##########	##########	-€ 9,302,931	-€ 18,605,862	-€ 27,908,793	-€ 37,211,724
1647	0.18	€ 286,507	€ 573,015	€ 859,522	##########	-€ 6,927,663	-€ 13,855,325	-€ 20,782,988	-€ 27,710,650
1648	0.20	€ 314,183	€ 628,366	€ 942,549	##########	-€ 7,596,853	-€ 15,193,706	-€ 22,790,559	-€ 30,387,412
1649	0.23	€ 369,638	€ 739,275	##########	##########	-€ 8,937,729	-€ 17,875,457	-€ 26,813,186	-€ 35,750,915
1650	0.15	€ 235,757	€ 471,513	€ 707,270	€ 943,027	-€ 5,700,528	-€ 11,401,056	-€ 17,101,585	-€ 22,802,113
1651	0.30	€ 466,281	€ 932,561	##########	##########	-€ 11,274,529	-€ 22,549,058	-€ 33,823,587	-€ 45,098,116
1652	0.18	€ 281,683	€ 563,366	€ 845,049	##########	-€ 6,811,012	-€ 13,622,024	-€ 20,433,035	-€ 27,244,047
1653	0.15	€ 239,382	€ 478,763	€ 718,145	€ 957,527	-€ 5,788,177	-€ 11,576,354	-€ 17,364,531	-€ 23,152,708
1654	0.16	€ 255,399	€ 510,798	€ 766,197	##########	-€ 6,175,475	-€ 12,350,949	-€ 18,526,424	-€ 24,701,899
1655	0.18	€ 289,990	€ 579,979	€ 869,969	##########	-€ 7,011,861	-€ 14,023,721	-€ 21,035,582	-€ 28,047,442
1656	0.18	€ 275,851	€ 551,702	€ 827,553	##########	-€ 6,669,996	-€ 13,339,992	-€ 20,009,989	-€ 26,679,985
1657	0.25	€ 397,600	€ 795,200	##########	##########	-€ 9,613,856	-€ 19,227,712	-€ 28,841,567	-€ 38,455,423
1658	0.14	€ 228,212	€ 456,424	€ 684,637	€ 912,849	-€ 5,518,104	-€ 11,036,209	-€ 16,554,313	-€ 22,072,417
1659	0.20	€ 317,199	€ 634,397	€ 951,596	##########	-€ 7,669,766	-€ 15,339,531	-€ 23,009,297	-€ 30,679,063
1660	0.20	€ 320,307	€ 640,614	€ 960,920	##########	-€ 7,744,922	-€ 15,489,844	-€ 23,234,767	-€ 30,979,689
1661	0.23	€ 356,036	€ 712,073	##########	##########	-€ 8,608,854	-€ 17,217,708	-€ 25,826,562	-€ 34,435,416
1662	0.14	€ 221,384	€ 442,769	€ 664,153	€ 885,537	-€ 5,353,006	-€ 10,706,013	-€ 16,059,019	-€ 21,412,025
1663	0.17	€ 260,604	€ 521,208	€ 781,812	##########	-€ 6,301,326	-€ 12,602,652	-€ 18,903,979	-€ 25,205,305
1664	0.21	€ 338,139	€ 676,277	##########	##########	-€ 8,176,093	-€ 16,352,186	-€ 24,528,278	-€ 32,704,371
1665	0.12	€ 189,976	€ 379,952	€ 569,927	€ 759,903	-€ 4,593,559	-€ 9,187,117	-€ 13,780,676	-€ 18,374,234
1666	0.16	€ 256,073	€ 512,147	€ 768,220	##########	-€ 6,191,781	-€ 12,383,562	-€ 18,575,343	-€ 24,767,124
1667	0.18	€ 281,978	€ 563,956	€ 845,934	##########	-€ 6,818,145	-€ 13,636,290	-€ 20,454,436	-€ 27,272,581
1668	0.18	€ 284,188	€ 568,375	€ 852,563	##########	-€ 6,871,571	-€ 13,743,141	-€ 20,614,712	-€ 27,486,282
1669	0.15	€ 243,601	€ 487,203	€ 730,804	€ 974,406	-€ 5,890,210	-€ 11,780,419	-€ 17,670,629	-€ 23,560,839
1670	0.19	€ 298,963	€ 597,925	€ 896,888	##########	-€ 7,228,826	-€ 14,457,651	-€ 21,686,477	-€ 28,915,302
1671	0.18	€ 282,922	€ 565,844	€ 848,765	##########	-€ 6,840,965	-€ 13,681,930	-€ 20,522,896	-€ 27,363,861
1672	0.21	€ 337,897	€ 675,794	##########	##########	-€ 8,170,245	-€ 16,340,489	-€ 24,510,734	-€ 32,680,979
1673	0.21	€ 337,169	€ 674,339	##########	##########	-€ 8,152,657	-€ 16,305,314	-€ 24,457,971	-€ 32,610,628
1674	0.13	€ 210,009	€ 420,017	€ 630,026	€ 840,035	-€ 5,077,949	-€ 10,155,897	-€ 15,233,846	-€ 20,311,794
1675	0.17	€ 268,123	€ 536,245	€ 804,368	##########	-€ 6,483,126	-€ 12,966,251	-€ 19,449,377	-€ 25,932,503
1676	0.17	€ 265,139	€ 530,278	€ 795,417	##########	-€ 6,410,982	-€ 12,821,964	-€ 19,232,946	-€ 25,643,927
1677	0.17	€ 267,818	€ 535,636	€ 803,455	##########	-€ 6,475,764	-€ 12,951,528	-€ 19,427,292	-€ 25,903,056
1678	0.21	€ 326,704	€ 653,408	€ 980,112	##########	-€ 7,899,603	-€ 15,799,206	-€ 23,698,809	-€ 31,598,411
1679	0.26	€ 408,911	€ 817,822	##########	##########	-€ 9,887,344	-€ 19,774,687	-€ 29,662,031	-€ 39,549,374
1680	0.15	€ 239,894	€ 479,788	€ 719,683	€ 959,577	-€ 5,800,571	-€ 11,601,141	-€ 17,401,712	-€ 23,202,282
1681	0.16	€ 248,246	€ 496,493	€ 744,739	€ 992,985	-€ 6,002,521	-€ 12,005,043	-€ 18,007,564	-€ 24,010,085
1682	0.16	€ 254,505	€ 509,011	€ 763,516	##########	-€ 6,153,867	-€ 12,307,734	-€ 18,461,601	-€ 24,615,468
1683	0.21	€ 323,946	€ 647,893	€ 971,839	##########	-€ 7,832,929	-€ 15,665,858	-€ 23,498,788	-€ 31,331,717
1684	0.23	€ 358,067	€ 716,134	##########	##########	-€ 8,657,957	-€ 17,315,913	-€ 25,973,870	-€ 34,631,826
1685	0.27	€ 427,088	€ 854,176	##########	##########	-€ 10,326,863	-€ 20,653,725	-€ 30,980,588	-€ 41,307,451
1686	0.25	€ 394,614	€ 789,228	##########	##########	-€ 9,541,649	-€ 19,083,298	-€ 28,624,947	-€ 38,166,596
1687	0.12	€ 196,545	€ 393,091	€ 589,636	€ 786,182	-€ 4,752,411	-€ 9,504,822	-€ 14,257,233	-€ 19,009,644
1688	0.16	€ 245,279	€ 490,558	€ 735,838	€ 981,117	-€ 5,930,778	-€ 11,861,557	-€ 17,792,335	-€ 23,723,113
1689	0.17	€ 275,297	€ 550,594	€ 825,890	##########	-€ 6,656,595	-€ 13,313,190	-€ 19,969,785	-€ 26,626,379
1690	0.24	€ 372,869	€ 745,738	##########	##########	-€ 9,015,863	-€ 18,031,725	-€ 27,047,588	-€ 36,063,451
1691	0.13	€ 196,962	€ 393,924	€ 590,885	€ 787,847	-€ 4,762,478	-€ 9,524,956	-€ 14,287,434	-€ 19,049,912
1692	0.26	€ 410,772	€ 821,545	##########	##########	-€ 9,932,353	-€ 19,864,707	-€ 29,797,060	-€ 39,729,413
1693	0.22	€ 351,933	€ 703,865	##########	##########	-€ 8,509,626	-€ 17,019,251	-€ 25,528,877	-€ 34,038,502
1694	0.16	€ 247,006	€ 494,011	€ 741,017	€ 988,022	-€ 5,972,521	-€ 11,945,042	-€ 17,917,562	-€ 23,890,083
1695	0.16	€ 248,877	€ 497,754	€ 746,631	€ 995,509	-€ 6,017,776	-€ 12,035,552	-€ 18,053,327	-€ 24,071,103
1696	0.21	€ 327,565	€ 655,129	€ 982,694	##########	-€ 7,920,416	-€ 15,840,833	-€ 23,761,249	-€ 31,681,665
1697	0.18	€ 281,687	€ 563,375	€ 845,062	##########	-€ 6,811,117	-€ 13,622,235	-€ 20,433,352	-€ 27,244,469
1698	0.16	€ 247,543	€ 495,085	€ 742,628	€ 990,171	-€ 5,985,509	-€ 11,971,017	-€ 17,956,526	-€ 23,942,034
1699	0.20	€ 320,375	€ 640,749	€ 961,124	##########	-€ 7,746,564	-€ 15,493,127	-€ 23,239,691	-€ 30,986,254
1700	0.19	€ 295,979	€ 591,958	€ 887,938	##########	-€ 7,156,689	-€ 14,313,378	-€ 21,470,068	-€ 28,626,757
1701	0.22	€ 341,205	€ 682,411	##########	##########	-€ 8,250,246	-€ 16,500,493	-€ 24,750,739	-€ 33,000,986
1702	0.27	€ 430,863	€ 861,726	##########	##########	-€ 10,418,137	-€ 20,836,275	-€ 31,254,412	-€ 41,672,549
1703	0.16	€ 251,704	€ 503,407	€ 755,111	##########	-€ 6,086,121	-€ 12,172,242	-€ 18,258,362	-€ 24,344,483
1704	0.15	€ 234,990	€ 469,979	€ 704,969	€ 939,958	-€ 5,681,978	-€ 11,363,955	-€ 17,045,933	-€ 22,727,910
1705	0.15	€ 235,751	€ 471,501	€ 707,252	€ 943,002	-€ 5,700,380	-€ 11,400,760	-€ 17,101,140	-€ 22,801,520
1706	0.19	€ 299,922	€ 599,845	€ 899,767	##########	-€ 7,252,036	-€ 14,504,072	-€ 21,756,108	-€ 29,008,144
1707	0.20	€ 320,604	€ 641,208	€ 961,813	##########	-€ 7,752,115	-€ 15,504,230	-€ 23,256,345	-€ 31,008,460
1708	0.22	€ 348,920	€ 697,839	##########	##########	-€ 8,436,772	-€ 16,873,545	-€ 25,310,317	-€ 33,747,089
1709	0.17	€ 265,374	€ 530,748	€ 796,123	##########	-€ 6,416,670	-€ 12,833,340	-€ 19,250,011	-€ 25,666,681
1710	0.23	€ 363,685	€ 727,371	##########	##########	-€ 8,793,803	-€ 17,587,606	-€ 26,381,410	-€ 35,175,213
1711	0.19	€ 305,597	€ 611,193	€ 916,790	##########	-€ 7,389,234	-€ 14,778,467	-€ 22,167,701	-€ 29,556,935
1712	0.19	€ 305,791	€ 611,582	€ 917,374	##########	-€ 7,393,941	-€ 14,787,882	-€ 22,181,823	-€ 29,575,764
1713	0.17	€ 263,326	€ 526,651	€ 789,977	##########	-€ 6,367,137	-€ 12,734,273	-€ 19,101,410	-€ 25,468,546
1714	0.19	€ 298,453	€ 596,905	€ 895,358	##########	-€ 7,216,498	-€ 14,432,996	-€ 21,649,494	-€ 28,865,993
1715	0.24	€ 372,102	€ 744,205	##########	##########	-€ 8,997,326	-€ 17,994,652	-€ 26,991,978	-€ 35,989,304
1716	0.19	€ 298,014	€ 596,027	€ 894,041	##########	-€ 7,205,880	-€ 14,411,760	-€ 21,617,640	-€ 28,823,520
1717	0.19	€ 300,259	€ 600,518	€ 900,776	##########	-€ 7,260,169	-€ 14,520,337	-€ 21,780,506	-€ 29,040,675
1718	0.25	€ 388,688	€ 777,377	##########	##########	-€ 9,398,369	-€ 18,796,737	-€ 28,195,106	-€ 37,593,474
1719	0.15	€ 238,191	€ 476,382	€ 714,574	€ 952,765	-€ 5,759,393	-€ 11,518,787	-€ 17,278,180	-€ 23,037,574
1720	0.14	€ 215,697	€ 431,394	€ 647,092	€ 862,789	-€ 5,215,495	-€ 10,430,990	-€ 15,646,484	-€ 20,861,979
1721	0.13	€ 204,823	€ 409,646	€ 614,470	€ 819,293	-€ 4,952,565	-€ 9,905,130	-€ 14,857,695	-€ 19,810,260
1722	0.24	€ 375,129	€ 750,258	##########	##########	-€ 9,070,507	-€ 18,141,014	-€ 27,211,521	-€ 36,282,029
1723	0.15	€ 231,994	€ 463,989	€ 695,983	€ 927,977	-€ 5,609,554	-€ 11,219,107	-€ 16,828,661	-€ 22,438,215
1724	0.19	€ 299,460	€ 598,919	€ 898,379	##########	-€ 7,240,846	-€ 14,481,692	-€ 21,722,538	-€ 28,963,384
1725	0.19	€ 303,999	€ 607,998	€ 911,997	##########	-€ 7,350,605	-€ 14,701,210	-€ 22,051,814	-€ 29,402,419
1726	0.18	€ 287,453	€ 574,906	€ 862,360	##########	-€ 6,950,532	-€ 13,901,065	-€ 20,851,597	-€ 27,802,129
1727	0.15	€ 237,035	€ 474,070	€ 711,106	€ 948,141	-€ 5,731,441	-€ 11,462,882	-€ 17,194,323	-€ 22,925,764
1728	0.19	€ 306,977	€ 613,954	€ 920,931	##########	-€ 7,422,613	-€ 14,845,225	-€ 22,267,838	-€ 29,690,450
1729	0.20	€ 310,885	€ 621,769	€ 932,654	##########	-€ 7,517,100	-€ 15,034,200	-€ 22,551,300	-€ 30,068,400
1730	0.17	€ 261,236	€ 522,471	€ 783,707	##########	-€ 6,316,601	-€ 12,633,201	-€ 18,949,802	-€ 25,266,403

1731	0.28	€ 440,917	€ 881,834	##########	##########	-€ 10,661,240	-€ 21,322,481	-€ 31,983,721	-€ 42,644,962
1732	0.20	€ 314,728	€ 629,455	€ 944,183	##########	-€ 7,610,021	-€ 15,220,042	-€ 22,830,063	-€ 30,440,084
1733	0.25	€ 393,810	€ 787,621	##########	##########	-€ 9,522,215	-€ 19,044,430	-€ 28,566,645	-€ 38,088,860
1734	0.16	€ 245,634	€ 491,267	€ 736,901	€ 982,535	-€ 5,939,349	-€ 11,878,697	-€ 17,818,046	-€ 23,757,394
1735	0.15	€ 242,482	€ 484,964	€ 727,445	€ 969,927	-€ 5,863,137	-€ 11,726,274	-€ 17,589,411	-€ 23,452,548
1736	0.26	€ 415,570	€ 831,141	##########	##########	-€ 10,048,366	-€ 20,096,732	-€ 30,145,098	-€ 40,193,464
1737	0.19	€ 295,383	€ 590,765	€ 886,148	##########	-€ 7,142,263	-€ 14,284,525	-€ 21,426,788	-€ 28,569,050
1738	0.19	€ 302,423	€ 604,846	€ 907,270	##########	-€ 7,312,503	-€ 14,625,006	-€ 21,937,508	-€ 29,250,011
1739	0.16	€ 248,511	€ 497,021	€ 745,532	€ 994,043	-€ 6,008,916	-€ 12,017,831	-€ 18,026,747	-€ 24,035,663
1740	0.20	€ 307,351	€ 614,703	€ 922,054	##########	-€ 7,431,667	-€ 14,863,334	-€ 22,295,001	-€ 29,726,669
1741	0.23	€ 356,827	€ 713,655	##########	##########	-€ 8,627,977	-€ 17,255,954	-€ 25,883,932	-€ 34,511,909
1742	0.20	€ 317,564	€ 635,128	€ 952,692	##########	-€ 7,678,606	-€ 15,357,212	-€ 23,035,818	-€ 30,714,424
1743	0.23	€ 356,417	€ 712,835	##########	##########	-€ 8,618,065	-€ 17,236,130	-€ 25,854,195	-€ 34,472,260
1744	0.13	€ 210,832	€ 421,663	€ 632,495	€ 843,326	-€ 5,097,845	-€ 10,195,689	-€ 15,293,534	-€ 20,391,379
1745	0.21	€ 328,936	€ 657,871	€ 986,807	##########	-€ 7,953,564	-€ 15,907,129	-€ 23,860,693	-€ 31,814,257
1746	0.15	€ 239,912	€ 479,825	€ 719,737	€ 959,650	-€ 5,801,012	-€ 11,602,025	-€ 17,403,037	-€ 23,204,049
1747	0.17	€ 263,977	€ 527,953	€ 791,930	##########	-€ 6,382,875	-€ 12,765,750	-€ 19,148,625	-€ 25,531,500
1748	0.15	€ 228,351	€ 456,702	€ 685,054	€ 913,405	-€ 5,521,464	-€ 11,042,928	-€ 16,564,391	-€ 22,085,855
1749	0.20	€ 311,107	€ 622,214	€ 933,321	##########	-€ 7,522,476	-€ 15,044,951	-€ 22,567,427	-€ 30,089,902
1750	0.17	€ 263,374	€ 526,748	€ 790,122	##########	-€ 6,368,302	-€ 12,736,604	-€ 19,104,906	-€ 25,473,207
1751	0.19	€ 299,177	€ 598,354	€ 897,531	##########	-€ 7,234,011	-€ 14,468,022	-€ 21,702,033	-€ 28,936,044
1752	0.16	€ 254,786	€ 509,571	€ 764,357	##########	-€ 6,160,643	-€ 12,321,286	-€ 18,481,929	-€ 24,642,572
1753	0.10	€ 160,815	€ 321,630	€ 482,445	€ 643,260	-€ 3,888,459	-€ 7,776,918	-€ 11,665,377	-€ 15,553,836
1754	0.23	€ 356,509	€ 713,018	##########	##########	-€ 8,620,282	-€ 17,240,565	-€ 25,860,847	-€ 34,481,130
1755	0.18	€ 279,077	€ 558,155	€ 837,232	##########	-€ 6,748,006	-€ 13,496,013	-€ 20,244,019	-€ 26,992,025
1756	0.22	€ 350,043	€ 700,087	##########	##########	-€ 8,463,942	-€ 16,927,884	-€ 25,391,827	-€ 33,855,769
1757	0.18	€ 283,666	€ 567,332	€ 850,997	##########	-€ 6,858,954	-€ 13,717,907	-€ 20,576,861	-€ 27,435,815
1758	0.22	€ 342,430	€ 684,860	##########	##########	-€ 8,279,850	-€ 16,559,700	-€ 24,839,550	-€ 33,119,401
1759	0.26	€ 408,684	€ 817,367	##########	##########	-€ 9,881,847	-€ 19,763,695	-€ 29,645,542	-€ 39,527,389
1760	0.18	€ 279,756	€ 559,511	€ 839,267	##########	-€ 6,764,406	-€ 13,528,812	-€ 20,293,218	-€ 27,057,624
1761	0.17	€ 263,927	€ 527,853	€ 791,780	##########	-€ 6,381,666	-€ 12,763,332	-€ 19,144,998	-€ 25,526,665
1762	0.21	€ 324,957	€ 649,914	€ 974,871	##########	-€ 7,857,361	-€ 15,714,722	-€ 23,572,084	-€ 31,429,445
1763	0.18	€ 287,650	€ 575,301	€ 862,951	##########	-€ 6,955,302	-€ 13,910,603	-€ 20,865,905	-€ 27,821,206
1764	0.17	€ 263,085	€ 526,170	€ 789,255	##########	-€ 6,361,317	-€ 12,722,633	-€ 19,083,950	-€ 25,445,267
1765	0.19	€ 297,119	€ 594,239	€ 891,358	##########	-€ 7,184,256	-€ 14,368,513	-€ 21,552,769	-€ 28,737,025
1766	0.18	€ 280,080	€ 560,160	€ 840,240	##########	-€ 6,772,247	-€ 13,544,495	-€ 20,316,742	-€ 27,088,989
1767	0.14	€ 216,999	€ 433,999	€ 650,998	€ 867,998	-€ 5,246,983	-€ 10,493,965	-€ 15,740,948	-€ 20,987,931
1768	0.21	€ 336,184	€ 672,368	##########	##########	-€ 8,128,826	-€ 16,257,652	-€ 24,386,478	-€ 32,515,304
1769	0.16	€ 244,352	€ 488,703	€ 733,055	€ 977,407	-€ 5,908,351	-€ 11,816,701	-€ 17,725,052	-€ 23,633,402
1770	0.17	€ 264,415	€ 528,831	€ 793,246	##########	-€ 6,393,487	-€ 12,786,974	-€ 19,180,461	-€ 25,573,949
1771	0.22	€ 347,661	€ 695,322	##########	##########	-€ 8,406,344	-€ 16,812,688	-€ 25,219,031	-€ 33,625,375
1772	0.16	€ 255,233	€ 510,465	€ 765,698	##########	-€ 6,171,449	-€ 12,342,899	-€ 18,514,348	-€ 24,685,798
1773	0.18	€ 287,774	€ 575,548	€ 863,322	##########	-€ 6,958,286	-€ 13,916,573	-€ 20,874,859	-€ 27,833,145
1774	0.18	€ 282,464	€ 564,927	€ 847,391	##########	-€ 6,829,889	-€ 13,659,779	-€ 20,489,668	-€ 27,319,558
1775	0.24	€ 381,269	€ 762,538	##########	##########	-€ 9,218,976	-€ 18,437,953	-€ 27,656,929	-€ 36,875,905
1776	0.20	€ 319,960	€ 639,920	€ 959,881	##########	-€ 7,736,544	-€ 15,473,088	-€ 23,209,631	-€ 30,946,175
1777	0.16	€ 253,544	€ 507,089	€ 760,633	##########	-€ 6,130,628	-€ 12,261,256	-€ 18,391,884	-€ 24,522,512
1778	0.16	€ 256,748	€ 513,497	€ 770,245	##########	-€ 6,208,098	-€ 12,416,196	-€ 18,624,294	-€ 24,832,392
1779	0.31	€ 480,788	€ 961,577	##########	##########	-€ 11,625,319	-€ 23,250,638	-€ 34,875,957	-€ 46,501,275
1780	0.18	€ 289,593	€ 579,185	€ 868,778	##########	-€ 7,002,261	-€ 14,004,522	-€ 21,006,784	-€ 28,009,045
1781	0.25	€ 399,717	€ 799,435	##########	##########	-€ 9,665,048	-€ 19,330,096	-€ 28,995,144	-€ 38,660,193
1782	0.24	€ 375,341	€ 750,681	##########	##########	-€ 9,075,626	-€ 18,151,252	-€ 27,226,878	-€ 36,302,503
1783	0.15	€ 242,292	€ 484,584	€ 726,877	€ 969,169	-€ 5,858,555	-€ 11,717,109	-€ 17,575,664	-€ 23,434,219
1784	0.18	€ 283,856	€ 567,712	€ 851,568	##########	-€ 6,863,552	-€ 13,727,104	-€ 20,590,657	-€ 27,454,209
1785	0.19	€ 306,416	€ 612,832	€ 919,248	##########	-€ 7,409,045	-€ 14,818,091	-€ 22,227,136	-€ 29,636,181
1786	0.18	€ 283,061	€ 566,122	€ 849,184	##########	-€ 6,844,335	-€ 13,688,671	-€ 20,533,006	-€ 27,377,341
1787	0.17	€ 275,276	€ 550,552	€ 825,828	##########	-€ 6,656,094	-€ 13,312,187	-€ 19,968,281	-€ 26,624,375
1788	0.17	€ 272,185	€ 544,371	€ 816,556	##########	-€ 6,581,359	-€ 13,162,718	-€ 19,744,077	-€ 26,325,435
1789	0.11	€ 176,019	€ 352,038	€ 528,058	€ 704,077	-€ 4,256,092	-€ 8,512,184	-€ 12,768,276	-€ 17,024,368
1790	0.17	€ 268,029	€ 536,058	€ 804,087	##########	-€ 6,480,859	-€ 12,961,719	-€ 19,442,578	-€ 25,923,438
1791	0.20	€ 311,751	€ 623,502	€ 935,253	##########	-€ 7,538,045	-€ 15,076,090	-€ 22,614,135	-€ 30,152,180
1792	0.13	€ 201,866	€ 403,732	€ 605,599	€ 807,465	-€ 4,881,064	-€ 9,762,128	-€ 14,643,192	-€ 19,524,256
1793	0.19	€ 303,965	€ 607,930	€ 911,895	##########	-€ 7,349,786	-€ 14,699,572	-€ 22,049,357	-€ 29,399,143
1794	0.17	€ 268,268	€ 536,535	€ 804,803	##########	-€ 6,486,631	-€ 12,973,261	-€ 19,459,892	-€ 25,946,522
1795	0.16	€ 248,738	€ 497,475	€ 746,213	€ 994,951	-€ 6,014,403	-€ 12,028,807	-€ 18,043,210	-€ 24,057,614
1796	0.18	€ 286,429	€ 572,858	€ 859,286	##########	-€ 6,925,763	-€ 13,851,526	-€ 20,777,288	-€ 27,703,051
1797	0.13	€ 211,615	€ 423,230	€ 634,845	€ 846,461	-€ 5,116,791	-€ 10,233,583	-€ 15,350,374	-€ 20,467,165
1798	0.16	€ 249,994	€ 499,989	€ 749,983	€ 999,977	-€ 6,044,788	-€ 12,089,576	-€ 18,134,364	-€ 24,179,153
1799	0.18	€ 285,323	€ 570,647	€ 855,970	##########	-€ 6,899,032	-€ 13,798,064	-€ 20,697,097	-€ 27,596,129
1800	0.16	€ 251,103	€ 502,205	€ 753,308	##########	-€ 6,071,589	-€ 12,143,178	-€ 18,214,768	-€ 24,286,357
1801	0.22	€ 352,629	€ 705,257	##########	##########	-€ 8,526,457	-€ 17,052,915	-€ 25,579,372	-€ 34,105,829
1802	0.17	€ 260,228	€ 520,456	€ 780,683	##########	-€ 6,292,231	-€ 12,584,463	-€ 18,876,694	-€ 25,168,926
1803	0.16	€ 256,292	€ 512,584	€ 768,876	##########	-€ 6,197,061	-€ 12,394,121	-€ 18,591,182	-€ 24,788,242
1804	0.27	€ 421,219	€ 842,439	##########	##########	-€ 10,184,961	-€ 20,369,923	-€ 30,554,884	-€ 40,739,846
1805	0.18	€ 290,628	€ 581,257	€ 871,885	##########	-€ 7,027,309	-€ 14,054,618	-€ 21,081,927	-€ 28,109,236
1806	0.18	€ 288,704	€ 577,408	€ 866,112	##########	-€ 6,980,778	-€ 13,961,556	-€ 20,942,333	-€ 27,923,111
1807	0.32	€ 497,805	€ 995,610	##########	##########	-€ 12,036,782	-€ 24,073,563	-€ 36,110,345	-€ 48,147,127
1808	0.24	€ 375,617	€ 751,234	##########	##########	-€ 9,082,306	-€ 18,164,611	-€ 27,246,917	-€ 36,329,222
1809	0.14	€ 226,743	€ 453,485	€ 680,228	€ 906,970	-€ 5,482,567	-€ 10,965,134	-€ 16,447,701	-€ 21,930,267
1810	0.22	€ 345,148	€ 690,296	##########	##########	-€ 8,345,573	-€ 16,691,146	-€ 25,036,718	-€ 33,382,291
1811	0.18	€ 284,190	€ 568,380	€ 852,570	##########	-€ 6,871,627	-€ 13,743,254	-€ 20,614,881	-€ 27,486,508
1812	0.19	€ 297,136	€ 594,272	€ 891,408	##########	-€ 7,184,658	-€ 14,369,315	-€ 21,553,973	-€ 28,738,630
1813	0.18	€ 290,673	€ 581,346	€ 872,019	##########	-€ 7,028,386	-€ 14,056,771	-€ 21,085,157	-€ 28,113,543
1814	0.23	€ 357,355	€ 714,710	##########	##########	-€ 8,640,744	-€ 17,281,488	-€ 25,922,231	-€ 34,562,975
1815	0.16	€ 253,397	€ 506,794	€ 760,191	##########	-€ 6,127,065	-€ 12,254,129	-€ 18,381,194	-€ 24,508,259
1816	0.22	€ 341,339	€ 682,678	##########	##########	-€ 8,253,473	-€ 16,506,945	-€ 24,760,418	-€ 33,013,891
1817	0.22	€ 346,725	€ 693,449	##########	##########	-€ 8,383,702	-€ 16,767,403	-€ 25,151,105	-€ 33,534,806
1818	0.20	€ 316,168	€ 632,336	€ 948,504	##########	-€ 7,644,849	-€ 15,289,699	-€ 22,934,548	-€ 30,579,397
1819	0.17	€ 273,468	€ 546,936	€ 820,404	##########	-€ 6,612,373	-€ 13,224,747	-€ 19,837,120	-€ 26,449,494
1820	0.14	€ 222,582	€ 445,165	€ 667,747	€ 890,330	-€ 5,381,977	-€ 10,763,953	-€ 16,145,930	-€ 21,527,906
1821	0.26	€ 414,533	€ 829,066	##########	##########	-€ 10,023,287	-€ 20,046,574	-€ 30,069,861	-€ 40,093,148
1822	0.15	€ 229,505	€ 459,009	€ 688,514	€ 918,018	-€ 5,549,351	-€ 11,098,703	-€ 16,648,054	-€ 22,197,405
1823	0.17	€ 263,176	€ 526,351	€ 789,527	##########	-€ 6,363,511	-€ 12,727,022	-€ 19,090,533	-€ 25,454,044
1824	0.26	€ 412,539	€ 825,078	##########	##########	-€ 9,975,069	-€ 19,950,139	-€ 29,925,208	-€ 39,900,278
1825	0.14	€ 222,237	€ 444,473	€ 666,710	€ 888,946	-€ 5,373,614	-€ 10,747,228	-€ 16,120,843	-€ 21,494,457
1826	0.14	€ 226,000	€ 452,000	€ 678,000	€ 904,000	-€ 5,464,611	-€ 10,929,221	-€ 16,393,832	-€ 21,858,442
1827	0.24	€ 378,891	€ 757,783	##########	##########	-€ 9,161,483	-€ 18,322,965	-€ 27,484,448	-€ 36,645,931
1828	0.18	€ 287,015	€ 574,030	€ 861,044	##########	-€ 6,939,933	-€ 13,879,867	-€ 20,819,800	-€ 27,759,733
1829	0.24	€ 381,129	€ 762,258	##########	##########	-€ 9,215,590	-€ 18,431,180	-€ 27,646,770	-€ 36,862,359
1830	0.24	€ 380,642	€ 761,285	##########	##########	-€ 9,203,819	-€ 18,407,637	-€ 27,611,456	-€ 36,815,274
1831	0.22	€ 352,225	€ 704,451	##########	##########	-€ 8,516,706	-€ 17,033,412	-€ 25,550,117	-€ 34,066,823
1832	0.18	€ 290,461	€ 580,922	€ 871,383	##########	-€ 7,023,263	-€ 14,046,526	-€ 21,069,789	-€ 28,093,051

1833	0.25	€ 396,220	€ 792,440	##########	##########	-€ 9,580,482	-€ 19,160,964	-€ 28,741,445	-€ 38,321,927
1834	0.14	€ 222,539	€ 445,078	€ 667,617	€ 890,156	-€ 5,380,924	-€ 10,761,848	-€ 16,142,772	-€ 21,523,696
1835	0.20	€ 314,496	€ 628,991	€ 943,487	##########	-€ 7,604,414	-€ 15,208,827	-€ 22,813,241	-€ 30,417,654
1836	0.14	€ 222,053	€ 444,105	€ 666,158	€ 888,211	-€ 5,369,168	-€ 10,738,335	-€ 16,107,503	-€ 21,476,670
1837	0.18	€ 283,022	€ 566,044	€ 849,066	##########	-€ 6,843,386	-€ 13,686,772	-€ 20,530,158	-€ 27,373,545
1838	0.22	€ 352,487	€ 704,973	##########	##########	-€ 8,523,023	-€ 17,046,046	-€ 25,569,069	-€ 34,092,092
1839	0.15	€ 231,481	€ 462,962	€ 694,443	€ 925,924	-€ 5,597,139	-€ 11,194,279	-€ 16,791,418	-€ 22,388,557
1840	0.20	€ 308,652	€ 617,303	€ 925,955	##########	-€ 7,463,102	-€ 14,926,204	-€ 22,389,305	-€ 29,852,407
1841	0.20	€ 319,220	€ 638,441	€ 957,661	##########	-€ 7,718,652	-€ 15,437,303	-€ 23,155,955	-€ 30,874,606
1842	0.18	€ 283,174	€ 566,347	€ 849,521	##########	-€ 6,847,055	-€ 13,694,110	-€ 20,541,164	-€ 27,388,219
1843	0.26	€ 409,143	€ 818,287	##########	##########	-€ 9,892,965	-€ 19,785,929	-€ 29,678,894	-€ 39,571,858
1844	0.12	€ 187,362	€ 374,723	€ 562,085	€ 749,446	-€ 4,530,346	-€ 9,060,692	-€ 13,591,038	-€ 18,121,385
1845	0.19	€ 292,808	€ 585,616	€ 878,425	##########	-€ 7,080,016	-€ 14,160,033	-€ 21,240,049	-€ 28,320,066
1846	0.18	€ 277,373	€ 554,746	€ 832,120	##########	-€ 6,706,802	-€ 13,413,605	-€ 20,120,407	-€ 26,827,209
1847	0.18	€ 279,861	€ 559,723	€ 839,584	##########	-€ 6,766,967	-€ 13,533,934	-€ 20,300,901	-€ 27,067,868
1848	0.17	€ 270,928	€ 541,855	€ 812,783	##########	-€ 6,550,950	-€ 13,101,901	-€ 19,652,851	-€ 26,203,802
1849	0.19	€ 299,915	€ 599,830	€ 899,745	##########	-€ 7,251,855	-€ 14,503,710	-€ 21,755,565	-€ 29,007,419
1850	0.17	€ 268,896	€ 537,792	€ 806,688	##########	-€ 6,501,823	-€ 13,003,645	-€ 19,505,468	-€ 26,007,291
1851	0.19	€ 293,396	€ 586,791	€ 880,187	##########	-€ 7,094,222	-€ 14,188,443	-€ 21,282,665	-€ 28,376,887
1852	0.24	€ 376,351	€ 752,702	##########	##########	-€ 9,100,060	-€ 18,200,120	-€ 27,300,181	-€ 36,400,241
1853	0.15	€ 241,331	€ 482,661	€ 723,992	€ 965,323	-€ 5,835,305	-€ 11,670,610	-€ 17,505,915	-€ 23,341,220
1854	0.20	€ 314,333	€ 628,665	€ 942,998	##########	-€ 7,600,471	-€ 15,200,942	-€ 22,801,414	-€ 30,401,885
1855	0.24	€ 373,388	€ 746,776	##########	##########	-€ 9,028,407	-€ 18,056,814	-€ 27,085,221	-€ 36,113,628
1856	0.24	€ 374,612	€ 749,225	##########	##########	-€ 9,058,016	-€ 18,116,033	-€ 27,174,049	-€ 36,232,066
1857	0.25	€ 393,225	€ 786,451	##########	##########	-€ 9,508,075	-€ 19,016,151	-€ 28,524,226	-€ 38,032,301
1858	0.19	€ 305,981	€ 611,961	€ 917,942	##########	-€ 7,398,520	-€ 14,797,041	-€ 22,195,561	-€ 29,594,082
1859	0.21	€ 326,114	€ 652,228	€ 978,342	##########	-€ 7,885,343	-€ 15,770,686	-€ 23,656,028	-€ 31,541,371
1860	0.17	€ 267,577	€ 535,153	€ 802,730	##########	-€ 6,469,922	-€ 12,939,844	-€ 19,409,767	-€ 25,879,689
1861	0.23	€ 355,189	€ 710,378	##########	##########	-€ 8,588,370	-€ 17,176,739	-€ 25,765,109	-€ 34,353,479
1862	0.14	€ 228,131	€ 456,262	€ 684,392	€ 912,523	-€ 5,516,135	-€ 11,032,270	-€ 16,548,404	-€ 22,064,539
1863	0.24	€ 380,771	€ 761,541	##########	##########	-€ 9,206,919	-€ 18,413,838	-€ 27,620,758	-€ 36,827,677
1864	0.18	€ 289,883	€ 579,765	€ 869,648	##########	-€ 7,009,278	-€ 14,018,555	-€ 21,027,833	-€ 28,037,110
1865	0.18	€ 286,479	€ 572,958	€ 859,436	##########	-€ 6,926,972	-€ 13,853,943	-€ 20,780,915	-€ 27,707,886
1866	0.19	€ 304,058	€ 608,117	€ 912,175	##########	-€ 7,352,041	-€ 14,704,082	-€ 22,056,123	-€ 29,408,164
1867	0.19	€ 296,150	€ 592,300	€ 888,449	##########	-€ 7,160,813	-€ 14,321,627	-€ 21,482,440	-€ 28,643,254
1868	0.24	€ 379,240	€ 758,480	##########	##########	-€ 9,169,905	-€ 18,339,810	-€ 27,509,715	-€ 36,679,619
1869	0.25	€ 401,059	€ 802,117	##########	##########	-€ 9,697,476	-€ 19,394,951	-€ 29,092,427	-€ 38,789,903
1870	0.19	€ 296,150	€ 592,301	€ 888,451	##########	-€ 7,160,825	-€ 14,321,651	-€ 21,482,476	-€ 28,643,301
1871	0.22	€ 352,638	€ 705,275	##########	##########	-€ 8,526,672	-€ 17,053,345	-€ 25,580,017	-€ 34,106,690
1872	0.21	€ 325,530	€ 651,060	€ 976,589	##########	-€ 7,871,213	-€ 15,742,427	-€ 23,613,640	-€ 31,484,854
1873	0.18	€ 285,349	€ 570,697	€ 856,046	##########	-€ 6,899,647	-€ 13,799,293	-€ 20,698,940	-€ 27,598,586
1874	0.16	€ 253,034	€ 506,067	€ 759,101	##########	-€ 6,118,277	-€ 12,236,555	-€ 18,354,832	-€ 24,473,109
1875	0.25	€ 385,789	€ 771,579	##########	##########	-€ 9,328,274	-€ 18,656,548	-€ 27,984,822	-€ 37,313,096
1876	0.19	€ 299,444	€ 598,888	€ 898,331	##########	-€ 7,240,462	-€ 14,480,924	-€ 21,721,386	-€ 28,961,848
1877	0.22	€ 344,249	€ 688,498	##########	##########	-€ 8,323,839	-€ 16,647,677	-€ 24,971,516	-€ 33,295,355
1878	0.17	€ 261,577	€ 523,154	€ 784,731	##########	-€ 6,324,851	-€ 12,649,703	-€ 18,974,554	-€ 25,299,406
1879	0.16	€ 250,697	€ 501,393	€ 752,090	##########	-€ 6,061,769	-€ 12,123,537	-€ 18,185,306	-€ 24,247,074
1880	0.16	€ 252,975	€ 505,950	€ 758,924	##########	-€ 6,116,856	-€ 12,233,712	-€ 18,350,568	-€ 24,467,424
1881	0.20	€ 310,971	€ 621,942	€ 932,913	##########	-€ 7,519,183	-€ 15,038,366	-€ 22,557,549	-€ 30,076,731
1882	0.24	€ 377,692	€ 755,384	##########	##########	-€ 9,132,476	-€ 18,264,951	-€ 27,397,427	-€ 36,529,902
1883	0.14	€ 228,026	€ 456,052	€ 684,078	€ 912,104	-€ 5,513,600	-€ 11,027,201	-€ 16,540,801	-€ 22,054,401
1884	0.15	€ 241,115	€ 482,230	€ 723,345	€ 964,459	-€ 5,830,086	-€ 11,660,172	-€ 17,490,258	-€ 23,320,344
1885	0.14	€ 219,722	€ 439,443	€ 659,165	€ 878,886	-€ 5,312,802	-€ 10,625,605	-€ 15,938,407	-€ 21,251,210
1886	0.15	€ 234,581	€ 469,163	€ 703,744	€ 938,325	-€ 5,672,107	-€ 11,344,214	-€ 17,016,321	-€ 22,688,428
1887	0.20	€ 315,066	€ 630,132	€ 945,199	##########	-€ 7,618,208	-€ 15,236,416	-€ 22,854,624	-€ 30,472,831
1888	0.23	€ 368,928	€ 737,857	##########	##########	-€ 8,920,580	-€ 17,841,159	-€ 26,761,739	-€ 35,682,318
1889	0.16	€ 252,262	€ 504,523	€ 756,785	##########	-€ 6,099,610	-€ 12,199,220	-€ 18,298,830	-€ 24,398,440
1890	0.13	€ 209,757	€ 419,515	€ 629,272	€ 839,029	-€ 5,071,869	-€ 10,143,739	-€ 15,215,608	-€ 20,287,477
1891	0.16	€ 245,301	€ 490,602	€ 735,903	€ 981,204	-€ 5,931,307	-€ 11,862,614	-€ 17,793,922	-€ 23,725,229
1892	0.14	€ 221,858	€ 443,717	€ 665,575	€ 887,433	-€ 5,364,468	-€ 10,728,936	-€ 16,093,403	-€ 21,457,871
1893	0.25	€ 391,688	€ 783,376	##########	##########	-€ 9,470,894	-€ 18,941,787	-€ 28,412,681	-€ 37,883,575
1894	0.24	€ 372,613	€ 745,226	##########	##########	-€ 9,009,666	-€ 18,019,332	-€ 27,028,998	-€ 36,038,665
1895	0.23	€ 356,088	€ 712,175	##########	##########	-€ 8,610,095	-€ 17,220,189	-€ 25,830,284	-€ 34,440,379
1896	0.26	€ 415,323	€ 830,646	##########	##########	-€ 10,042,391	-€ 20,084,782	-€ 30,127,173	-€ 40,169,564
1897	0.22	€ 342,966	€ 685,932	##########	##########	-€ 8,292,811	-€ 16,585,622	-€ 24,878,434	-€ 33,171,245
1898	0.18	€ 286,403	€ 572,805	€ 859,208	##########	-€ 6,925,133	-€ 13,850,267	-€ 20,775,400	-€ 27,700,534
1899	0.15	€ 235,646	€ 471,291	€ 706,937	€ 942,582	-€ 5,697,841	-€ 11,395,681	-€ 17,093,522	-€ 22,791,363
1900	0.21	€ 327,633	€ 655,266	€ 982,899	##########	-€ 7,922,071	-€ 15,844,142	-€ 23,766,213	-€ 31,688,284
1901	0.15	€ 239,310	€ 478,621	€ 717,931	€ 957,241	-€ 5,786,453	-€ 11,572,907	-€ 17,359,360	-€ 23,145,813
1902	0.18	€ 278,339	€ 556,678	€ 835,018	##########	-€ 6,730,160	-€ 13,460,320	-€ 20,190,481	-€ 26,920,641
1903	0.13	€ 212,138	€ 424,276	€ 636,414	€ 848,552	-€ 5,129,433	-€ 10,258,866	-€ 15,388,299	-€ 20,517,732
1904	0.21	€ 334,892	€ 669,783	##########	##########	-€ 8,097,582	-€ 16,195,163	-€ 24,292,745	-€ 32,390,326
1905	0.19	€ 292,183	€ 584,366	€ 876,549	##########	-€ 7,064,899	-€ 14,129,797	-€ 21,194,696	-€ 28,259,595
1906	0.18	€ 279,022	€ 558,044	€ 837,065	##########	-€ 6,746,664	-€ 13,493,327	-€ 20,239,991	-€ 26,986,654
1907	0.18	€ 284,748	€ 569,496	€ 854,245	##########	-€ 6,885,128	-€ 13,770,256	-€ 20,655,384	-€ 27,540,512
1908	0.17	€ 263,319	€ 526,639	€ 789,958	##########	-€ 6,366,984	-€ 12,733,967	-€ 19,100,951	-€ 25,467,934
1909	0.13	€ 204,016	€ 408,032	€ 612,048	€ 816,065	-€ 4,933,050	-€ 9,866,101	-€ 14,799,151	-€ 19,732,201
1910	0.22	€ 350,064	€ 700,127	##########	##########	-€ 8,464,434	-€ 16,928,868	-€ 25,393,303	-€ 33,857,737
1911	0.19	€ 298,874	€ 597,748	€ 896,622	##########	-€ 7,226,688	-€ 14,453,375	-€ 21,680,063	-€ 28,906,750
1912	0.24	€ 376,255	€ 752,509	##########	##########	-€ 9,097,725	-€ 18,195,449	-€ 27,293,174	-€ 36,390,898
1913	0.22	€ 338,763	€ 677,526	##########	##########	-€ 8,191,193	-€ 16,382,387	-€ 24,573,580	-€ 32,764,774
1914	0.18	€ 290,622	€ 581,245	€ 871,867	##########	-€ 7,027,162	-€ 14,054,323	-€ 21,081,485	-€ 28,108,646
1915	0.30	€ 465,191	€ 930,383	##########	##########	-€ 11,248,188	-€ 22,496,377	-€ 33,744,565	-€ 44,992,753
1916	0.18	€ 278,426	€ 556,851	€ 835,277	##########	-€ 6,732,247	-€ 13,464,494	-€ 20,196,741	-€ 26,928,988
1917	0.16	€ 252,813	€ 505,626	€ 758,439	##########	-€ 6,112,947	-€ 12,225,893	-€ 18,338,840	-€ 24,451,786
1918	0.26	€ 409,866	€ 819,732	##########	##########	-€ 9,910,434	-€ 19,820,868	-€ 29,731,303	-€ 39,641,737
1919	0.20	€ 307,669	€ 615,339	€ 923,008	##########	-€ 7,439,353	-€ 14,878,706	-€ 22,318,059	-€ 29,757,413
1920	0.19	€ 293,045	€ 586,090	€ 879,135	##########	-€ 7,085,743	-€ 14,171,486	-€ 21,257,229	-€ 28,342,972
1921	0.20	€ 321,630	€ 643,259	€ 964,889	##########	-€ 7,776,911	-€ 15,553,823	-€ 23,330,734	-€ 31,107,646
1922	0.17	€ 272,248	€ 544,496	€ 816,744	##########	-€ 6,582,879	-€ 13,165,759	-€ 19,748,638	-€ 26,331,518
1923	0.16	€ 257,375	€ 514,750	€ 772,125	##########	-€ 6,223,252	-€ 12,446,505	-€ 18,669,757	-€ 24,893,010
1924	0.16	€ 255,755	€ 511,510	€ 767,265	##########	-€ 6,184,078	-€ 12,368,156	-€ 18,552,234	-€ 24,736,312
1925	0.12	€ 196,037	€ 392,074	€ 588,110	€ 784,147	-€ 4,740,111	-€ 9,480,223	-€ 14,220,334	-€ 18,960,445
1926	0.23	€ 357,784	€ 715,568	##########	##########	-€ 8,651,110	-€ 17,302,220	-€ 25,953,330	-€ 34,604,440
1927	0.15	€ 229,347	€ 458,695	€ 688,042	€ 917,390	-€ 5,545,552	-€ 11,091,103	-€ 16,636,655	-€ 22,182,207
1928	0.25	€ 395,492	€ 790,984	##########	##########	-€ 9,562,880	-€ 19,125,761	-€ 28,688,641	-€ 38,251,521
1929	0.16	€ 257,766	€ 515,531	€ 773,297	##########	-€ 6,232,694	-€ 12,465,388	-€ 18,698,082	-€ 24,930,776
1930	0.18	€ 279,789	€ 559,578	€ 839,367	##########	-€ 6,765,218	-€ 13,530,436	-€ 20,295,654	-€ 27,060,871
1931	0.18	€ 283,494	€ 566,988	€ 850,483	##########	-€ 6,854,806	-€ 13,709,612	-€ 20,564,418	-€ 27,419,224
1932	0.14	€ 216,473	€ 432,946	€ 649,419	€ 865,892	-€ 5,234,253	-€ 10,468,507	-€ 15,702,760	-€ 20,937,014
1933	0.21	€ 336,448	€ 672,896	##########	##########	-€ 8,135,207	-€ 16,270,414	-€ 24,405,621	-€ 32,540,828
1934	0.15	€ 243,778	€ 487,555	€ 731,333	€ 975,110	-€ 5,894,469	-€ 11,788,937	-€ 17,683,406	-€ 23,577,875

1935	0.21	€ 331,858	€ 663,717	€ 995,575	##########	-€ 8,024,237	-€ 16,048,474	-€ 24,072,711	-€ 32,096,948
1936	0.16	€ 257,953	€ 515,905	€ 773,858	##########	-€ 6,237,216	-€ 12,474,432	-€ 18,711,647	-€ 24,948,863
1937	0.19	€ 300,484	€ 600,968	€ 901,451	##########	-€ 7,265,609	-€ 14,531,218	-€ 21,796,828	-€ 29,062,437
1938	0.23	€ 356,227	€ 712,454	##########	##########	-€ 8,613,469	-€ 17,226,938	-€ 25,840,407	-€ 34,453,876
1939	0.24	€ 371,399	€ 742,798	##########	##########	-€ 8,980,314	-€ 17,960,629	-€ 26,940,943	-€ 35,921,257
1940	0.15	€ 232,259	€ 464,519	€ 696,778	€ 929,037	-€ 5,615,962	-€ 11,231,923	-€ 16,847,885	-€ 22,463,847
1941	0.10	€ 156,684	€ 313,368	€ 470,052	€ 626,736	-€ 3,788,571	-€ 7,577,142	-€ 11,365,712	-€ 15,154,283
1942	0.16	€ 250,037	€ 500,074	€ 750,111	##########	-€ 6,045,820	-€ 12,091,641	-€ 18,137,461	-€ 24,183,281
1943	0.22	€ 343,936	€ 687,872	##########	##########	-€ 8,316,271	-€ 16,632,543	-€ 24,948,814	-€ 33,265,086
1944	0.24	€ 379,891	€ 759,781	##########	##########	-€ 9,185,640	-€ 18,371,281	-€ 27,556,921	-€ 36,742,561
1945	0.18	€ 278,689	€ 557,378	€ 836,068	##########	-€ 6,738,622	-€ 13,477,244	-€ 20,215,866	-€ 26,954,487
1946	0.12	€ 191,396	€ 382,792	€ 574,188	€ 765,584	-€ 4,627,896	-€ 9,255,792	-€ 13,883,688	-€ 18,511,584
1947	0.19	€ 298,213	€ 596,427	€ 894,640	##########	-€ 7,210,713	-€ 14,421,426	-€ 21,632,139	-€ 28,842,852
1948	0.16	€ 254,940	€ 509,880	€ 764,820	##########	-€ 6,164,375	-€ 12,328,750	-€ 18,493,125	-€ 24,657,500
1949	0.24	€ 383,226	€ 766,451	##########	##########	-€ 9,266,284	-€ 18,532,568	-€ 27,798,853	-€ 37,065,137
1950	0.21	€ 335,364	€ 670,729	##########	##########	-€ 8,109,011	-€ 16,218,022	-€ 24,327,033	-€ 32,436,044
1951	0.17	€ 269,884	€ 539,768	€ 809,651	##########	-€ 6,525,709	-€ 13,051,419	-€ 19,577,128	-€ 26,102,837
1952	0.16	€ 252,371	€ 504,742	€ 757,113	##########	-€ 6,102,254	-€ 12,204,509	-€ 18,306,763	-€ 24,409,017
1953	0.19	€ 306,553	€ 613,106	€ 919,659	##########	-€ 7,412,361	-€ 14,824,722	-€ 22,237,083	-€ 29,649,444
1954	0.22	€ 344,204	€ 688,408	##########	##########	-€ 8,322,751	-€ 16,645,503	-€ 24,968,254	-€ 33,291,005
1955	0.20	€ 309,420	€ 618,840	€ 928,260	##########	-€ 7,481,683	-€ 14,963,365	-€ 22,445,048	-€ 29,926,731
1956	0.22	€ 345,912	€ 691,824	##########	##########	-€ 8,364,055	-€ 16,728,111	-€ 25,092,166	-€ 33,456,221
1957	0.14	€ 220,604	€ 441,208	€ 661,812	€ 882,416	-€ 5,334,141	-€ 10,668,282	-€ 16,002,423	-€ 21,336,564
1958	0.25	€ 394,057	€ 788,115	##########	##########	-€ 9,528,192	-€ 19,056,385	-€ 28,584,577	-€ 38,112,770
1959	0.13	€ 206,510	€ 413,019	€ 619,529	€ 826,039	-€ 4,993,342	-€ 9,986,684	-€ 14,980,025	-€ 19,973,367
1960	0.27	€ 420,707	€ 841,414	##########	##########	-€ 10,172,575	-€ 20,345,149	-€ 30,517,724	-€ 40,690,298
1961	0.13	€ 200,109	€ 400,219	€ 600,328	€ 800,438	-€ 4,838,587	-€ 9,677,175	-€ 14,515,762	-€ 19,354,349
1962	0.22	€ 339,242	€ 678,483	##########	##########	-€ 8,202,762	-€ 16,405,524	-€ 24,608,286	-€ 32,811,048
1963	0.14	€ 217,362	€ 434,724	€ 652,086	€ 869,448	-€ 5,255,749	-€ 10,511,498	-€ 15,767,247	-€ 21,022,996
1964	0.21	€ 335,524	€ 671,047	##########	##########	-€ 8,112,861	-€ 16,225,721	-€ 24,338,582	-€ 32,451,443
1965	0.24	€ 372,161	€ 744,322	##########	##########	-€ 8,998,743	-€ 17,997,486	-€ 26,996,229	-€ 35,994,972
1966	0.19	€ 299,535	€ 599,069	€ 898,604	##########	-€ 7,242,658	-€ 14,485,316	-€ 21,727,974	-€ 28,970,632
1967	0.21	€ 336,976	€ 673,951	##########	##########	-€ 8,147,970	-€ 16,295,940	-€ 24,443,910	-€ 32,591,880
1968	0.22	€ 346,762	€ 693,524	##########	##########	-€ 8,384,606	-€ 16,769,212	-€ 25,153,818	-€ 33,538,424
1969	0.19	€ 304,398	€ 608,797	€ 913,195	##########	-€ 7,360,264	-€ 14,720,527	-€ 22,080,791	-€ 29,441,055
1970	0.20	€ 320,810	€ 641,621	€ 962,431	##########	-€ 7,757,100	-€ 15,514,200	-€ 23,271,299	-€ 31,028,399
1971	0.15	€ 239,749	€ 479,498	€ 719,247	€ 958,996	-€ 5,797,059	-€ 11,594,119	-€ 17,391,178	-€ 23,188,237
1972	0.22	€ 353,507	€ 707,013	##########	##########	-€ 8,547,688	-€ 17,095,376	-€ 25,643,064	-€ 34,190,752
1973	0.20	€ 315,665	€ 631,330	€ 946,994	##########	-€ 7,632,681	-€ 15,265,362	-€ 22,898,043	-€ 30,530,725
1974	0.21	€ 327,293	€ 654,586	€ 981,880	##########	-€ 7,913,852	-€ 15,827,705	-€ 23,741,557	-€ 31,655,410
1975	0.31	€ 491,626	€ 983,251	##########	##########	-€ 11,887,359	-€ 23,774,718	-€ 35,662,077	-€ 47,549,436
1976	0.25	€ 393,658	€ 787,317	##########	##########	-€ 9,518,544	-€ 19,037,088	-€ 28,555,632	-€ 38,074,176
1977	0.22	€ 345,687	€ 691,375	##########	##########	-€ 8,358,620	-€ 16,717,240	-€ 25,075,861	-€ 33,434,481
1978	0.16	€ 247,971	€ 495,943	€ 743,914	€ 991,885	-€ 5,995,872	-€ 11,991,743	-€ 17,987,615	-€ 23,983,486
1979	0.14	€ 218,002	€ 436,005	€ 654,007	€ 872,010	-€ 5,271,234	-€ 10,542,468	-€ 15,813,702	-€ 21,084,936
1980	0.16	€ 249,297	€ 498,595	€ 747,892	€ 997,189	-€ 6,027,935	-€ 12,055,870	-€ 18,083,804	-€ 24,111,739
1981	0.20	€ 307,043	€ 614,085	€ 921,128	##########	-€ 7,424,198	-€ 14,848,397	-€ 22,272,595	-€ 29,696,793
1982	0.16	€ 244,141	€ 488,283	€ 732,424	€ 976,566	-€ 5,903,267	-€ 11,806,534	-€ 17,709,801	-€ 23,613,067
1983	0.22	€ 349,806	€ 699,611	##########	##########	-€ 8,458,195	-€ 16,916,390	-€ 25,374,586	-€ 33,832,781
1984	0.18	€ 281,145	€ 562,291	€ 843,436	##########	-€ 6,798,010	-€ 13,596,021	-€ 20,394,031	-€ 27,192,042
1985	0.17	€ 263,180	€ 526,360	€ 789,540	##########	-€ 6,363,615	-€ 12,727,231	-€ 19,090,846	-€ 25,454,461
1986	0.28	€ 439,707	€ 879,415	##########	##########	-€ 10,631,996	-€ 21,263,991	-€ 31,895,987	-€ 42,527,983
1987	0.20	€ 313,403	€ 626,805	€ 940,208	##########	-€ 7,577,984	-€ 15,155,967	-€ 22,733,951	-€ 30,311,935
1988	0.13	€ 198,003	€ 396,006	€ 594,009	€ 792,013	-€ 4,787,657	-€ 9,575,314	-€ 14,362,971	-€ 19,150,628
1989	0.16	€ 250,691	€ 501,382	€ 752,073	##########	-€ 6,061,633	-€ 12,123,266	-€ 18,184,899	-€ 24,246,532
1990	0.24	€ 372,057	€ 744,113	##########	##########	-€ 8,996,220	-€ 17,992,440	-€ 26,988,660	-€ 35,984,880
1991	0.20	€ 319,100	€ 638,200	€ 957,300	##########	-€ 7,715,743	-€ 15,431,486	-€ 23,147,230	-€ 30,862,973
1992	0.17	€ 262,487	€ 524,975	€ 787,462	##########	-€ 6,346,867	-€ 12,693,733	-€ 19,040,600	-€ 25,387,467
1993	0.14	€ 218,197	€ 436,394	€ 654,591	€ 872,788	-€ 5,275,938	-€ 10,551,876	-€ 15,827,815	-€ 21,103,753
1994	0.15	€ 243,107	€ 486,214	€ 729,321	€ 972,429	-€ 5,878,259	-€ 11,756,518	-€ 17,634,777	-€ 23,513,036
1995	0.26	€ 416,506	€ 833,012	##########	##########	-€ 10,070,995	-€ 20,141,991	-€ 30,212,986	-€ 40,283,982
1996	0.16	€ 256,891	€ 513,782	€ 770,674	##########	-€ 6,211,554	-€ 12,423,108	-€ 18,634,662	-€ 24,846,216
1997	0.11	€ 180,502	€ 361,004	€ 541,506	€ 722,007	-€ 4,364,481	-€ 8,728,962	-€ 13,093,444	-€ 17,457,925
1998	0.23	€ 369,594	€ 739,187	##########	##########	-€ 8,936,662	-€ 17,873,324	-€ 26,809,986	-€ 35,746,648
1999	0.13	€ 200,042	€ 400,084	€ 600,126	€ 800,169	-€ 4,836,960	-€ 9,673,920	-€ 14,510,880	-€ 19,347,840
2000	0.15	€ 238,075	€ 476,149	€ 714,224	€ 952,298	-€ 5,756,573	-€ 11,513,146	-€ 17,269,719	-€ 23,026,292
2001	0.23	€ 359,542	€ 719,085	##########	##########	-€ 8,693,625	-€ 17,387,250	-€ 26,080,875	-€ 34,774,501
2002	0.15	€ 228,758	€ 457,516	€ 686,273	€ 915,031	-€ 5,531,296	-€ 11,062,593	-€ 16,593,889	-€ 22,125,185
2003	0.18	€ 281,324	€ 562,649	€ 843,973	##########	-€ 6,802,343	-€ 13,604,685	-€ 20,407,028	-€ 27,209,371
2004	0.16	€ 248,342	€ 496,684	€ 745,025	€ 993,367	-€ 6,004,830	-€ 12,009,660	-€ 18,014,490	-€ 24,019,320
2005	0.23	€ 362,223	€ 724,447	##########	##########	-€ 8,758,451	-€ 17,516,903	-€ 26,275,354	-€ 35,033,806
2006	0.26	€ 414,677	€ 829,355	##########	##########	-€ 10,026,774	-€ 20,053,548	-€ 30,080,322	-€ 40,107,097
2007	0.20	€ 321,406	€ 642,813	€ 964,219	##########	-€ 7,771,512	-€ 15,543,024	-€ 23,314,535	-€ 31,086,047
2008	0.15	€ 241,740	€ 483,480	€ 725,220	€ 966,960	-€ 5,845,199	-€ 11,690,397	-€ 17,535,596	-€ 23,380,794
2009	0.20	€ 312,581	€ 625,162	€ 937,743	##########	-€ 7,558,115	-€ 15,116,229	-€ 22,674,344	-€ 30,232,459
2010	0.16	€ 255,491	€ 510,983	€ 766,474	##########	-€ 6,177,707	-€ 12,355,414	-€ 18,533,121	-€ 24,710,828
2011	0.18	€ 286,311	€ 572,621	€ 858,932	##########	-€ 6,922,907	-€ 13,845,814	-€ 20,768,721	-€ 27,691,628
2012	0.20	€ 311,025	€ 622,050	€ 933,076	##########	-€ 7,520,497	-€ 15,040,994	-€ 22,561,490	-€ 30,081,987
2013	0.10	€ 163,456	€ 326,913	€ 490,369	€ 653,825	-€ 3,952,326	-€ 7,904,651	-€ 11,856,977	-€ 15,809,303
2014	0.25	€ 395,085	€ 790,169	##########	##########	-€ 9,553,028	-€ 19,106,056	-€ 28,659,084	-€ 38,212,112
2015	0.18	€ 285,885	€ 571,770	€ 857,655	##########	-€ 6,912,618	-€ 13,825,235	-€ 20,737,853	-€ 27,650,471
2016	0.20	€ 312,964	€ 625,929	€ 938,893	##########	-€ 7,567,387	-€ 15,134,773	-€ 22,702,160	-€ 30,269,546
2017	0.14	€ 221,466	€ 442,933	€ 664,399	€ 885,865	-€ 5,354,991	-€ 10,709,981	-€ 16,064,972	-€ 21,419,963
2018	0.19	€ 299,950	€ 599,900	€ 899,850	##########	-€ 7,252,703	-€ 14,505,405	-€ 21,758,108	-€ 29,010,810
2019	0.24	€ 384,611	€ 769,221	##########	##########	-€ 9,299,769	-€ 18,599,539	-€ 27,899,308	-€ 37,199,077
2020	0.23	€ 355,279	€ 710,559	##########	##########	-€ 8,590,552	-€ 17,181,105	-€ 25,771,657	-€ 34,362,210
2021	0.13	€ 211,974	€ 423,948	€ 635,922	€ 847,895	-€ 5,125,465	-€ 10,250,930	-€ 15,376,395	-€ 20,501,860
2022	0.14	€ 224,555	€ 449,110	€ 673,665	€ 898,221	-€ 5,429,677	-€ 10,859,354	-€ 16,289,031	-€ 21,718,709
2023	0.27	€ 429,068	€ 858,136	##########	##########	-€ 10,374,740	-€ 20,749,479	-€ 31,124,219	-€ 41,498,958
2024	0.22	€ 352,943	€ 705,886	##########	##########	-€ 8,534,052	-€ 17,068,104	-€ 25,602,156	-€ 34,136,207
2025	0.22	€ 345,610	€ 691,220	##########	##########	-€ 8,356,745	-€ 16,713,491	-€ 25,070,236	-€ 33,426,981
2026	0.22	€ 339,921	€ 679,843	##########	##########	-€ 8,219,195	-€ 16,438,390	-€ 24,657,585	-€ 32,876,781
2027	0.18	€ 282,687	€ 565,374	€ 848,060	##########	-€ 6,835,283	-€ 13,670,566	-€ 20,505,850	-€ 27,341,133
2028	0.17	€ 267,580	€ 535,159	€ 802,739	##########	-€ 6,469,996	-€ 12,939,992	-€ 19,409,988	-€ 25,879,983
2029	0.16	€ 248,968	€ 497,935	€ 746,903	€ 995,871	-€ 6,019,965	-€ 12,039,929	-€ 18,059,894	-€ 24,079,859
2030	0.20	€ 308,926	€ 617,851	€ 926,777	##########	-€ 7,469,733	-€ 14,939,465	-€ 22,409,198	-€ 29,878,931
2031	0.21	€ 324,845	€ 649,689	€ 974,534	##########	-€ 7,854,646	-€ 15,709,292	-€ 23,563,937	-€ 31,418,583
2032	0.23	€ 358,466	€ 716,931	##########	##########	-€ 8,667,593	-€ 17,335,186	-€ 26,002,779	-€ 34,670,371
2033	0.30	€ 472,006	€ 944,012	##########	##########	-€ 11,412,962	-€ 22,825,925	-€ 34,238,887	-€ 45,651,850
2034	0.17	€ 269,619	€ 539,238	€ 808,858	##########	-€ 6,519,313	-€ 13,038,626	-€ 19,557,939	-€ 26,077,252
2035	0.20	€ 313,909	€ 627,819	€ 941,728	##########	-€ 7,590,238	-€ 15,180,477	-€ 22,770,715	-€ 30,360,954
2036	0.17	€ 267,582	€ 535,164	€ 802,746	##########	-€ 6,470,052	-€ 12,940,103	-€ 19,410,155	-€ 25,880,207

2037	0.20	€ 312,688	€ 625,377	€ 938,065	##########	-€ 7,560,710	-€ 15,121,420	-€ 22,682,130	-€ 30,242,840
2038	0.21	€ 329,941	€ 659,882	€ 989,822	##########	-€ 7,977,870	-€ 15,955,740	-€ 23,933,609	-€ 31,911,479
2039	0.20	€ 314,247	€ 628,493	€ 942,740	##########	-€ 7,598,390	-€ 15,196,781	-€ 22,795,171	-€ 30,393,562
2040	0.19	€ 296,532	€ 593,064	€ 889,596	##########	-€ 7,170,054	-€ 14,340,109	-€ 21,510,163	-€ 28,680,218
2041	0.22	€ 348,094	€ 696,188	##########	##########	-€ 8,416,808	-€ 16,833,617	-€ 25,250,425	-€ 33,667,233
2042	0.16	€ 252,017	€ 504,033	€ 756,050	##########	-€ 6,093,690	-€ 12,187,380	-€ 18,281,071	-€ 24,374,761
2043	0.13	€ 206,006	€ 412,012	€ 618,017	€ 824,023	-€ 4,981,160	-€ 9,962,319	-€ 14,943,479	-€ 19,924,638
2044	0.24	€ 382,491	€ 764,983	##########	##########	-€ 9,248,531	-€ 18,497,061	-€ 27,745,592	-€ 36,994,122
2045	0.20	€ 313,528	€ 627,056	€ 940,584	##########	-€ 7,581,013	-€ 15,162,027	-€ 22,743,040	-€ 30,324,053
2046	0.15	€ 238,951	€ 477,901	€ 716,852	€ 955,802	-€ 5,777,753	-€ 11,555,506	-€ 17,333,259	-€ 23,111,012
2047	0.14	€ 222,901	€ 445,802	€ 668,704	€ 891,605	-€ 5,389,684	-€ 10,779,368	-€ 16,169,052	-€ 21,558,736
2048	0.20	€ 310,662	€ 621,324	€ 931,986	##########	-€ 7,511,711	-€ 15,023,422	-€ 22,535,133	-€ 30,046,844
2049	0.25	€ 397,124	€ 794,248	##########	##########	-€ 9,602,342	-€ 19,204,683	-€ 28,807,025	-€ 38,409,367
2050	0.16	€ 247,732	€ 495,464	€ 743,196	€ 990,928	-€ 5,990,087	-€ 11,980,175	-€ 17,970,262	-€ 23,960,350
2051	0.20	€ 321,421	€ 642,841	€ 964,262	##########	-€ 7,771,854	-€ 15,543,708	-€ 23,315,563	-€ 31,087,417
2052	0.25	€ 385,928	€ 771,855	##########	##########	-€ 9,331,614	-€ 18,663,229	-€ 27,994,843	-€ 37,326,457
2053	0.21	€ 327,117	€ 654,234	€ 981,351	##########	-€ 7,909,595	-€ 15,819,190	-€ 23,728,785	-€ 31,638,380
2054	0.22	€ 347,087	€ 694,174	##########	##########	-€ 8,392,464	-€ 16,784,928	-€ 25,177,392	-€ 33,569,856
2055	0.18	€ 286,698	€ 573,397	€ 860,095	##########	-€ 6,932,279	-€ 13,864,557	-€ 20,796,836	-€ 27,729,114
2056	0.17	€ 267,777	€ 535,553	€ 803,330	##########	-€ 6,474,757	-€ 12,949,513	-€ 19,424,270	-€ 25,899,026
2057	0.17	€ 263,037	€ 526,073	€ 789,110	##########	-€ 6,360,145	-€ 12,720,289	-€ 19,080,434	-€ 25,440,579
2058	0.26	€ 406,478	€ 812,956	##########	##########	-€ 9,828,518	-€ 19,657,035	-€ 29,485,553	-€ 39,314,071
2059	0.20	€ 315,297	€ 630,594	€ 945,891	##########	-€ 7,623,785	-€ 15,247,571	-€ 22,871,356	-€ 30,495,142
2060	0.16	€ 257,663	€ 515,326	€ 772,989	##########	-€ 6,230,218	-€ 12,460,436	-€ 18,690,654	-€ 24,920,872
2061	0.19	€ 298,596	€ 597,192	€ 895,789	##########	-€ 7,219,968	-€ 14,439,936	-€ 21,659,904	-€ 28,879,873
2062	0.22	€ 343,602	€ 687,204	##########	##########	-€ 8,308,189	-€ 16,616,377	-€ 24,924,566	-€ 33,232,754
2063	0.17	€ 268,755	€ 537,510	€ 806,264	##########	-€ 6,498,410	-€ 12,996,821	-€ 19,495,231	-€ 25,993,641
2064	0.12	€ 191,259	€ 382,517	€ 573,776	€ 765,034	-€ 4,624,575	-€ 9,249,149	-€ 13,873,724	-€ 18,498,298
2065	0.15	€ 235,785	€ 471,571	€ 707,356	€ 943,141	-€ 5,701,219	-€ 11,402,438	-€ 17,103,657	-€ 22,804,877
2066	0.21	€ 332,698	€ 665,396	€ 998,094	##########	-€ 8,044,537	-€ 16,089,075	-€ 24,133,612	-€ 32,178,150
2067	0.17	€ 274,462	€ 548,924	€ 823,385	##########	-€ 6,636,404	-€ 13,272,809	-€ 19,909,213	-€ 26,545,617
2068	0.27	€ 421,516	€ 843,033	##########	##########	-€ 10,192,143	-€ 20,384,286	-€ 30,576,428	-€ 40,768,571
2069	0.26	€ 402,292	€ 804,583	##########	##########	-€ 9,727,294	-€ 19,454,588	-€ 29,181,883	-€ 38,909,177
2070	0.21	€ 327,366	€ 654,733	€ 982,099	##########	-€ 7,915,625	-€ 15,831,249	-€ 23,746,874	-€ 31,662,498
2071	0.25	€ 398,928	€ 797,856	##########	##########	-€ 9,645,956	-€ 19,291,912	-€ 28,937,868	-€ 38,583,824
2072	0.16	€ 256,932	€ 513,864	€ 770,796	##########	-€ 6,212,539	-€ 12,425,077	-€ 18,637,616	-€ 24,850,154
2073	0.17	€ 263,506	€ 527,011	€ 790,517	##########	-€ 6,371,487	-€ 12,742,973	-€ 19,114,460	-€ 25,485,947
2074	0.24	€ 383,925	€ 767,851	##########	##########	-€ 9,283,202	-€ 18,566,404	-€ 27,849,606	-€ 37,132,809
2075	0.20	€ 320,819	€ 641,638	€ 962,457	##########	-€ 7,757,304	-€ 15,514,609	-€ 23,271,913	-€ 31,029,218
2076	0.19	€ 294,137	€ 588,273	€ 882,410	##########	-€ 7,112,138	-€ 14,224,277	-€ 21,336,415	-€ 28,448,554
2077	0.25	€ 387,562	€ 775,125	##########	##########	-€ 9,371,142	-€ 18,742,283	-€ 28,113,425	-€ 37,484,566
2078	0.19	€ 302,040	€ 604,080	€ 906,120	##########	-€ 7,303,235	-€ 14,606,470	-€ 21,909,704	-€ 29,212,939
2079	0.18	€ 280,008	€ 560,016	€ 840,024	##########	-€ 6,770,510	-€ 13,541,020	-€ 20,311,529	-€ 27,082,039
2080	0.14	€ 219,094	€ 438,188	€ 657,282	€ 876,376	-€ 5,297,629	-€ 10,595,258	-€ 15,892,887	-€ 21,190,516
2081	0.22	€ 346,302	€ 692,605	##########	##########	-€ 8,373,487	-€ 16,746,975	-€ 25,120,462	-€ 33,493,949
2082	0.23	€ 365,459	€ 730,919	##########	##########	-€ 8,836,698	-€ 17,673,396	-€ 26,510,094	-€ 35,346,793
2083	0.21	€ 337,029	€ 674,057	##########	##########	-€ 8,149,252	-€ 16,298,504	-€ 24,447,755	-€ 32,597,007
2084	0.14	€ 223,994	€ 447,988	€ 671,982	€ 895,977	-€ 5,416,112	-€ 10,832,224	-€ 16,248,336	-€ 21,664,447
2085	0.16	€ 259,387	€ 518,775	€ 778,162	##########	-€ 6,271,911	-€ 12,543,822	-€ 18,815,733	-€ 25,087,645
2086	0.18	€ 285,602	€ 571,205	€ 856,807	##########	-€ 6,905,778	-€ 13,811,556	-€ 20,717,334	-€ 27,623,112
2087	0.16	€ 252,814	€ 505,628	€ 758,442	##########	-€ 6,112,969	-€ 12,225,938	-€ 18,338,907	-€ 24,451,875
2088	0.16	€ 249,781	€ 499,563	€ 749,344	€ 999,125	-€ 6,039,639	-€ 12,079,277	-€ 18,118,916	-€ 24,158,555
2089	0.19	€ 304,091	€ 608,182	€ 912,273	##########	-€ 7,352,827	-€ 14,705,654	-€ 22,058,481	-€ 29,411,307
2090	0.17	€ 262,305	€ 524,611	€ 786,916	##########	-€ 6,342,467	-€ 12,684,933	-€ 19,027,400	-€ 25,369,867
2091	0.21	€ 329,655	€ 659,309	€ 988,964	##########	-€ 7,970,950	-€ 15,941,900	-€ 23,912,850	-€ 31,883,800
2092	0.22	€ 352,460	€ 704,921	##########	##########	-€ 8,522,385	-€ 17,044,769	-€ 25,567,154	-€ 34,089,538
2093	0.22	€ 350,756	€ 701,511	##########	##########	-€ 8,481,168	-€ 16,962,336	-€ 25,443,504	-€ 33,924,672
2094	0.24	€ 373,286	€ 746,572	##########	##########	-€ 9,025,942	-€ 18,051,885	-€ 27,077,827	-€ 36,103,769
2095	0.25	€ 397,959	€ 795,917	##########	##########	-€ 9,622,519	-€ 19,245,038	-€ 28,867,557	-€ 38,490,076
2096	0.22	€ 345,564	€ 691,129	##########	##########	-€ 8,355,643	-€ 16,711,287	-€ 25,066,930	-€ 33,422,574
2097	0.18	€ 277,140	€ 554,280	€ 831,420	##########	-€ 6,701,166	-€ 13,402,332	-€ 20,103,498	-€ 26,804,664
2098	0.27	€ 420,264	€ 840,529	##########	##########	-€ 10,161,868	-€ 20,323,735	-€ 30,485,603	-€ 40,647,470
2099	0.19	€ 302,965	€ 605,930	€ 908,895	##########	-€ 7,325,605	-€ 14,651,209	-€ 21,976,814	-€ 29,302,418
2100	0.17	€ 261,287	€ 522,574	€ 783,861	##########	-€ 6,317,843	-€ 12,635,687	-€ 18,953,530	-€ 25,271,373
2101	0.20	€ 313,454	€ 626,908	€ 940,363	##########	-€ 7,579,230	-€ 15,158,460	-€ 22,737,689	-€ 30,316,919
2102	0.18	€ 279,566	€ 559,132	€ 838,698	##########	-€ 6,759,827	-€ 13,519,653	-€ 20,279,480	-€ 27,039,306
2103	0.23	€ 361,483	€ 722,966	##########	##########	-€ 8,740,556	-€ 17,481,113	-€ 26,221,669	-€ 34,962,225
2104	0.21	€ 327,114	€ 654,227	€ 981,341	##########	-€ 7,909,511	-€ 15,819,022	-€ 23,728,533	-€ 31,638,045
2105	0.23	€ 359,603	€ 719,207	##########	##########	-€ 8,695,102	-€ 17,390,204	-€ 26,085,306	-€ 34,780,408
2106	0.17	€ 265,664	€ 531,328	€ 796,992	##########	-€ 6,423,674	-€ 12,847,348	-€ 19,271,022	-€ 25,694,696
2107	0.21	€ 324,187	€ 648,374	€ 972,561	##########	-€ 7,838,746	-€ 15,677,491	-€ 23,516,237	-€ 31,354,982
2108	0.17	€ 272,489	€ 544,977	€ 817,466	##########	-€ 6,588,694	-€ 13,177,389	-€ 19,766,083	-€ 26,354,778
2109	0.24	€ 380,773	€ 761,547	##########	##########	-€ 9,206,989	-€ 18,413,979	-€ 27,620,968	-€ 36,827,957
2110	0.17	€ 267,175	€ 534,349	€ 801,524	##########	-€ 6,460,204	-€ 12,920,407	-€ 19,380,611	-€ 25,840,814
2111	0.20	€ 307,748	€ 615,495	€ 923,243	##########	-€ 7,441,244	-€ 14,882,489	-€ 22,323,733	-€ 29,764,978
2112	0.16	€ 256,166	€ 512,333	€ 768,499	##########	-€ 6,194,026	-€ 12,388,052	-€ 18,582,078	-€ 24,776,104
2113	0.18	€ 285,906	€ 571,812	€ 857,718	##########	-€ 6,913,124	-€ 13,826,248	-€ 20,739,372	-€ 27,652,497
2114	0.19	€ 306,239	€ 612,477	€ 918,716	##########	-€ 7,404,758	-€ 14,809,515	-€ 22,214,273	-€ 29,619,031
2115	0.20	€ 310,936	€ 621,871	€ 932,807	##########	-€ 7,518,334	-€ 15,036,668	-€ 22,555,002	-€ 30,073,337
2116	0.20	€ 315,031	€ 630,061	€ 945,092	##########	-€ 7,617,348	-€ 15,234,696	-€ 22,852,043	-€ 30,469,391
2117	0.16	€ 244,839	€ 489,678	€ 734,516	€ 979,355	-€ 5,920,130	-€ 11,840,260	-€ 17,760,391	-€ 23,680,521
2118	0.23	€ 366,302	€ 732,605	##########	##########	-€ 8,857,083	-€ 17,714,165	-€ 26,571,248	-€ 35,428,330
2119	0.18	€ 284,380	€ 568,760	€ 853,141	##########	-€ 6,876,229	-€ 13,752,458	-€ 20,628,686	-€ 27,504,915
2120	0.16	€ 251,506	€ 503,013	€ 754,519	##########	-€ 6,081,347	-€ 12,162,694	-€ 18,244,041	-€ 24,325,388
2121	0.15	€ 240,541	€ 481,082	€ 721,624	€ 962,165	-€ 5,816,214	-€ 11,632,428	-€ 17,448,642	-€ 23,264,857
2122	0.17	€ 264,470	€ 528,940	€ 793,410	##########	-€ 6,394,805	-€ 12,789,610	-€ 19,184,415	-€ 25,579,220
2123	0.24	€ 376,906	€ 753,811	##########	##########	-€ 9,113,466	-€ 18,226,933	-€ 27,340,399	-€ 36,453,866
2124	0.18	€ 275,657	€ 551,314	€ 826,971	##########	-€ 6,665,302	-€ 13,330,604	-€ 19,995,906	-€ 26,661,207
2125	0.19	€ 300,333	€ 600,665	€ 900,998	##########	-€ 7,261,954	-€ 14,523,909	-€ 21,785,863	-€ 29,047,818
2126	0.16	€ 258,587	€ 517,173	€ 775,760	##########	-€ 6,252,545	-€ 12,505,091	-€ 18,757,636	-€ 25,010,181
2127	0.18	€ 279,465	€ 558,930	€ 838,395	##########	-€ 6,757,384	-€ 13,514,768	-€ 20,272,152	-€ 27,029,536
2128	0.21	€ 331,227	€ 662,453	€ 993,680	##########	-€ 8,008,959	-€ 16,017,917	-€ 24,026,876	-€ 32,035,834
2129	0.18	€ 290,457	€ 580,915	€ 871,372	##########	-€ 7,023,176	-€ 14,046,351	-€ 21,069,527	-€ 28,092,702
2130	0.17	€ 263,041	€ 526,081	€ 789,122	##########	-€ 6,360,245	-€ 12,720,490	-€ 19,080,734	-€ 25,440,979
2131	0.22	€ 344,465	€ 688,929	##########	##########	-€ 8,329,054	-€ 16,658,109	-€ 24,987,163	-€ 33,316,218
2132	0.19	€ 301,309	€ 602,617	€ 903,926	##########	-€ 7,285,554	-€ 14,571,109	-€ 21,856,663	-€ 29,142,218
2133	0.22	€ 341,904	€ 683,808	##########	##########	-€ 8,267,138	-€ 16,534,275	-€ 24,801,413	-€ 33,068,551
2134	0.16	€ 256,538	€ 513,076	€ 769,614	##########	-€ 6,203,017	-€ 12,406,033	-€ 18,609,050	-€ 24,812,067
2135	0.16	€ 251,093	€ 502,187	€ 753,280	##########	-€ 6,071,362	-€ 12,142,725	-€ 18,214,087	-€ 24,285,450
2136	0.24	€ 381,877	€ 763,754	##########	##########	-€ 9,233,668	-€ 18,467,335	-€ 27,701,003	-€ 36,934,670
2137	0.23	€ 357,034	€ 714,068	##########	##########	-€ 8,632,982	-€ 17,265,963	-€ 25,898,945	-€ 34,531,927
2138	0.13	€ 196,899	€ 393,798	€ 590,697	€ 787,596	-€ 4,760,961	-€ 9,521,922	-€ 14,282,882	-€ 19,043,843

2139	0.15	€ 236,404	€ 472,807	€ 709,211	€ 945,614	-€ 5,716,167	-€ 11,432,334	-€ 17,148,501	-€ 22,864,668
2140	0.19	€ 305,731	€ 611,461	€ 917,192	##########	-€ 7,392,478	-€ 14,784,957	-€ 22,177,435	-€ 29,569,913
2141	0.30	€ 464,808	€ 929,615	##########	##########	-€ 11,238,910	-€ 22,477,819	-€ 33,716,729	-€ 44,955,638
2142	0.27	€ 419,355	€ 838,709	##########	##########	-€ 10,139,871	-€ 20,279,741	-€ 30,419,612	-€ 40,559,483
2143	0.17	€ 274,383	€ 548,766	€ 823,148	##########	-€ 6,634,494	-€ 13,268,989	-€ 19,903,483	-€ 26,537,978
2144	0.14	€ 214,646	€ 429,291	€ 643,937	€ 858,583	-€ 5,190,069	-€ 10,380,138	-€ 15,570,207	-€ 20,760,275
2145	0.14	€ 218,511	€ 437,022	€ 655,534	€ 874,045	-€ 5,283,536	-€ 10,567,072	-€ 15,850,608	-€ 21,134,143
2146	0.10	€ 160,682	€ 321,364	€ 482,046	€ 642,728	-€ 3,885,244	-€ 7,770,489	-€ 11,655,733	-€ 15,540,978
2147	0.17	€ 267,801	€ 535,603	€ 803,404	##########	-€ 6,475,356	-€ 12,950,712	-€ 19,426,068	-€ 25,901,424
2148	0.11	€ 181,064	€ 362,128	€ 543,192	€ 724,257	-€ 4,378,078	-€ 8,756,156	-€ 13,134,233	-€ 17,512,311
2149	0.12	€ 187,474	€ 374,948	€ 562,422	€ 749,897	-€ 4,533,070	-€ 9,066,139	-€ 13,599,209	-€ 18,132,278
2150	0.20	€ 322,639	€ 645,279	€ 967,918	##########	-€ 7,801,325	-€ 15,602,651	-€ 23,403,976	-€ 31,205,302
2151	0.18	€ 278,665	€ 557,330	€ 835,996	##########	-€ 6,738,041	-€ 13,476,082	-€ 20,214,124	-€ 26,952,165
2152	0.28	€ 444,525	€ 889,049	##########	##########	-€ 10,748,472	-€ 21,496,943	-€ 32,245,415	-€ 42,993,886
2153	0.21	€ 323,060	€ 646,120	€ 969,180	##########	-€ 7,811,499	-€ 15,622,998	-€ 23,434,497	-€ 31,245,996
2154	0.19	€ 300,992	€ 601,984	€ 902,976	##########	-€ 7,277,896	-€ 14,555,792	-€ 21,833,688	-€ 29,111,584
2155	0.15	€ 243,942	€ 487,884	€ 731,827	€ 975,769	-€ 5,898,450	-€ 11,796,899	-€ 17,695,349	-€ 23,593,799
2156	0.14	€ 225,888	€ 451,775	€ 677,663	€ 903,551	-€ 5,461,896	-€ 10,923,792	-€ 16,385,689	-€ 21,847,585
2157	0.17	€ 269,442	€ 538,885	€ 808,327	##########	-€ 6,515,035	-€ 13,030,070	-€ 19,545,105	-€ 26,060,140
2158	0.16	€ 255,362	€ 510,724	€ 766,086	##########	-€ 6,174,578	-€ 12,349,155	-€ 18,523,733	-€ 24,698,310
2159	0.26	€ 405,795	€ 811,589	##########	##########	-€ 9,811,996	-€ 19,623,992	-€ 29,435,989	-€ 39,247,985
2160	0.20	€ 314,869	€ 629,737	€ 944,606	##########	-€ 7,613,430	-€ 15,226,860	-€ 22,840,290	-€ 30,453,720
2161	0.22	€ 342,375	€ 684,750	##########	##########	-€ 8,278,529	-€ 16,557,058	-€ 24,835,587	-€ 33,114,116
2162	0.28	€ 434,813	€ 869,626	##########	##########	-€ 10,513,648	-€ 21,027,296	-€ 31,540,944	-€ 42,054,591
2163	0.20	€ 319,665	€ 639,329	€ 958,994	##########	-€ 7,729,395	-€ 15,458,790	-€ 23,188,185	-€ 30,917,580
2164	0.27	€ 418,569	€ 837,139	##########	##########	-€ 10,120,886	-€ 20,241,772	-€ 30,362,658	-€ 40,483,543
2165	0.16	€ 252,336	€ 504,672	€ 757,008	##########	-€ 6,101,413	-€ 12,202,826	-€ 18,304,239	-€ 24,405,652
2166	0.20	€ 307,679	€ 615,358	€ 923,038	##########	-€ 7,439,591	-€ 14,879,183	-€ 22,318,774	-€ 29,758,366
2167	0.13	€ 204,037	€ 408,073	€ 612,110	€ 816,147	-€ 4,933,547	-€ 9,867,093	-€ 14,800,640	-€ 19,734,186
2168	0.31	€ 487,449	€ 974,898	##########	##########	-€ 11,786,371	-€ 23,572,741	-€ 35,359,112	-€ 47,145,483
2169	0.14	€ 219,901	€ 439,802	€ 659,702	€ 879,603	-€ 5,317,136	-€ 10,634,273	-€ 15,951,409	-€ 21,268,546
2170	0.13	€ 199,385	€ 398,769	€ 598,154	€ 797,538	-€ 4,821,059	-€ 9,642,118	-€ 14,463,177	-€ 19,284,236
2171	0.18	€ 285,584	€ 571,167	€ 856,751	##########	-€ 6,905,325	-€ 13,810,651	-€ 20,715,976	-€ 27,621,301
2172	0.15	€ 232,910	€ 465,820	€ 698,730	€ 931,640	-€ 5,631,695	-€ 11,263,389	-€ 16,895,084	-€ 22,526,778
2173	0.21	€ 333,684	€ 667,369	##########	##########	-€ 8,068,389	-€ 16,136,778	-€ 24,205,168	-€ 32,273,557
2174	0.29	€ 457,905	€ 915,811	##########	##########	-€ 11,072,019	-€ 22,144,038	-€ 33,216,057	-€ 44,288,076
2175	0.19	€ 298,374	€ 596,748	€ 895,121	##########	-€ 7,214,590	-€ 14,429,180	-€ 21,643,770	-€ 28,858,360
2176	0.13	€ 200,798	€ 401,595	€ 602,393	€ 803,190	-€ 4,855,225	-€ 9,710,450	-€ 14,565,675	-€ 19,420,900
2177	0.27	€ 425,161	€ 850,321	##########	##########	-€ 10,280,260	-€ 20,560,520	-€ 30,840,781	-€ 41,121,041
2178	0.21	€ 337,093	€ 674,186	##########	##########	-€ 8,150,807	-€ 16,301,614	-€ 24,452,420	-€ 32,603,227
2179	0.21	€ 328,390	€ 656,779	€ 985,169	##########	-€ 7,940,366	-€ 15,880,731	-€ 23,821,097	-€ 31,761,462
2180	0.19	€ 300,656	€ 601,312	€ 901,968	##########	-€ 7,269,775	-€ 14,539,550	-€ 21,809,325	-€ 29,079,100
2181	0.22	€ 352,855	€ 705,710	##########	##########	-€ 8,531,932	-€ 17,063,863	-€ 25,595,795	-€ 34,127,726
2182	0.26	€ 409,489	€ 818,978	##########	##########	-€ 9,901,317	-€ 19,802,634	-€ 29,703,951	-€ 39,605,268
2183	0.16	€ 258,166	€ 516,333	€ 774,499	##########	-€ 6,242,389	-€ 12,484,778	-€ 18,727,167	-€ 24,969,556
2184	0.23	€ 363,179	€ 726,359	##########	##########	-€ 8,781,571	-€ 17,563,141	-€ 26,344,712	-€ 35,126,282
2185	0.21	€ 327,219	€ 654,437	€ 981,656	##########	-€ 7,912,051	-€ 15,824,103	-€ 23,736,154	-€ 31,648,205
2186	0.19	€ 298,238	€ 596,475	€ 894,713	##########	-€ 7,211,297	-€ 14,422,594	-€ 21,633,890	-€ 28,845,187
2187	0.18	€ 288,488	€ 576,976	€ 865,463	##########	-€ 6,975,549	-€ 13,951,097	-€ 20,926,646	-€ 27,902,195
2188	0.15	€ 237,550	€ 475,099	€ 712,649	€ 950,199	-€ 5,743,882	-€ 11,487,765	-€ 17,231,647	-€ 22,975,529
2189	0.16	€ 258,789	€ 517,579	€ 776,368	##########	-€ 6,257,449	-€ 12,514,897	-€ 18,772,346	-€ 25,029,795
2190	0.21	€ 328,190	€ 656,380	€ 984,571	##########	-€ 7,935,542	-€ 15,871,084	-€ 23,806,626	-€ 31,742,168
2191	0.25	€ 391,678	€ 783,355	##########	##########	-€ 9,470,649	-€ 18,941,298	-€ 28,411,947	-€ 37,882,596
2192	0.14	€ 228,243	€ 456,485	€ 684,728	€ 912,971	-€ 5,518,841	-€ 11,037,682	-€ 16,556,524	-€ 22,075,365
2193	0.19	€ 303,421	€ 606,841	€ 910,262	##########	-€ 7,336,620	-€ 14,673,240	-€ 22,009,860	-€ 29,346,480
2194	0.28	€ 434,277	€ 868,554	##########	##########	-€ 10,500,692	-€ 21,001,383	-€ 31,502,075	-€ 42,002,767
2195	0.19	€ 292,992	€ 585,983	€ 878,975	##########	-€ 7,084,449	-€ 14,168,897	-€ 21,253,346	-€ 28,337,795
2196	0.17	€ 275,086	€ 550,173	€ 825,259	##########	-€ 6,651,509	-€ 13,303,017	-€ 19,954,526	-€ 26,606,035
2197	0.24	€ 370,075	€ 740,151	##########	##########	-€ 8,948,311	-€ 17,896,621	-€ 26,844,932	-€ 35,793,243
2198	0.15	€ 235,097	€ 470,194	€ 705,290	€ 940,387	-€ 5,684,570	-€ 11,369,140	-€ 17,053,710	-€ 22,738,280
2199	0.12	€ 191,328	€ 382,657	€ 573,985	€ 765,313	-€ 4,626,261	-€ 9,252,523	-€ 13,878,784	-€ 18,505,046
2200	0.18	€ 281,556	€ 563,112	€ 844,669	##########	-€ 6,807,945	-€ 13,615,891	-€ 20,423,836	-€ 27,231,781
2201	0.22	€ 349,299	€ 698,598	##########	##########	-€ 8,445,950	-€ 16,891,901	-€ 25,337,851	-€ 33,783,802
2202	0.17	€ 262,804	€ 525,609	€ 788,413	##########	-€ 6,354,529	-€ 12,709,058	-€ 19,063,587	-€ 25,418,116
2203	0.21	€ 329,998	€ 659,996	€ 989,993	##########	-€ 7,979,248	-€ 15,958,495	-€ 23,937,743	-€ 31,916,991
2204	0.16	€ 257,097	€ 514,195	€ 771,292	##########	-€ 6,216,539	-€ 12,433,077	-€ 18,649,616	-€ 24,866,155
2205	0.16	€ 256,741	€ 513,482	€ 770,224	##########	-€ 6,207,926	-€ 12,415,852	-€ 18,623,777	-€ 24,831,703
2206	0.23	€ 359,350	€ 718,701	##########	##########	-€ 8,688,983	-€ 17,377,965	-€ 26,066,948	-€ 34,755,930
2207	0.13	€ 205,656	€ 411,311	€ 616,967	€ 822,623	-€ 4,972,692	-€ 9,945,384	-€ 14,918,076	-€ 19,890,768
2208	0.16	€ 259,615	€ 519,229	€ 778,844	##########	-€ 6,277,402	-€ 12,554,804	-€ 18,832,207	-€ 25,109,609
2209	0.18	€ 278,497	€ 556,995	€ 835,492	##########	-€ 6,733,984	-€ 13,467,968	-€ 20,201,952	-€ 26,935,936
2210	0.22	€ 351,389	€ 702,778	##########	##########	-€ 8,496,484	-€ 16,992,967	-€ 25,489,451	-€ 33,985,934
2211	0.15	€ 231,927	€ 463,854	€ 695,781	€ 927,709	-€ 5,607,930	-€ 11,215,859	-€ 16,823,789	-€ 22,431,718
2212	0.16	€ 247,739	€ 495,477	€ 743,216	€ 990,955	-€ 5,990,248	-€ 11,980,497	-€ 17,970,745	-€ 23,960,993
2213	0.20	€ 309,901	€ 619,803	€ 929,704	##########	-€ 7,493,321	-€ 14,986,641	-€ 22,479,962	-€ 29,973,282
2214	0.17	€ 274,083	€ 548,167	€ 822,250	##########	-€ 6,627,257	-€ 13,254,513	-€ 19,881,770	-€ 26,509,027
2215	0.23	€ 360,870	€ 721,740	##########	##########	-€ 8,725,732	-€ 17,451,464	-€ 26,177,196	-€ 34,902,928
2216	0.19	€ 294,569	€ 589,138	€ 883,707	##########	-€ 7,122,594	-€ 14,245,187	-€ 21,367,781	-€ 28,490,375
2217	0.19	€ 298,961	€ 597,921	€ 896,882	##########	-€ 7,228,779	-€ 14,457,559	-€ 21,686,338	-€ 28,915,117
2218	0.12	€ 186,823	€ 373,647	€ 560,470	€ 747,294	-€ 4,517,335	-€ 9,034,670	-€ 13,552,004	-€ 18,069,339
2219	0.19	€ 293,560	€ 587,120	€ 880,680	##########	-€ 7,098,196	-€ 14,196,392	-€ 21,294,588	-€ 28,392,784
2220	0.20	€ 315,715	€ 631,430	€ 947,145	##########	-€ 7,633,896	-€ 15,267,793	-€ 22,901,689	-€ 30,535,586
2221	0.12	€ 188,574	€ 377,148	€ 565,722	€ 754,296	-€ 4,559,661	-€ 9,119,322	-€ 13,678,984	-€ 18,238,645
2222	0.22	€ 349,506	€ 699,011	##########	##########	-€ 8,450,941	-€ 16,901,881	-€ 25,352,822	-€ 33,803,762
2223	0.21	€ 328,386	€ 656,772	€ 985,158	##########	-€ 7,940,274	-€ 15,880,547	-€ 23,820,821	-€ 31,761,095
2224	0.23	€ 357,962	€ 715,924	##########	##########	-€ 8,655,416	-€ 17,310,832	-€ 25,966,247	-€ 34,621,663
2225	0.20	€ 320,853	€ 641,705	€ 962,558	##########	-€ 7,758,124	-€ 15,516,248	-€ 23,274,372	-€ 31,032,496
2226	0.14	€ 212,897	€ 425,793	€ 638,690	€ 851,587	-€ 5,147,780	-€ 10,295,560	-€ 15,443,341	-€ 20,591,121
2227	0.27	€ 426,950	€ 853,899	##########	##########	-€ 10,323,517	-€ 20,647,033	-€ 30,970,550	-€ 41,294,066
2228	0.18	€ 289,760	€ 579,521	€ 869,281	##########	-€ 7,006,320	-€ 14,012,640	-€ 21,018,960	-€ 28,025,279
2229	0.10	€ 163,848	€ 327,696	€ 491,545	€ 655,393	-€ 3,961,802	-€ 7,923,603	-€ 11,885,405	-€ 15,847,207
2230	0.29	€ 454,826	€ 909,652	##########	##########	-€ 10,997,556	-€ 21,995,111	-€ 32,992,667	-€ 43,990,222
2231	0.16	€ 249,535	€ 499,070	€ 748,606	€ 998,141	-€ 6,033,686	-€ 12,067,373	-€ 18,101,059	-€ 24,134,746
2232	0.19	€ 296,681	€ 593,362	€ 890,042	##########	-€ 7,173,653	-€ 14,347,307	-€ 21,520,960	-€ 28,694,613
2233	0.14	€ 225,648	€ 451,296	€ 676,944	€ 902,591	-€ 5,456,099	-€ 10,912,197	-€ 16,368,296	-€ 21,824,394
2234	0.10	€ 161,842	€ 323,685	€ 485,527	€ 647,370	-€ 3,913,303	-€ 7,826,606	-€ 11,739,909	-€ 15,653,213
2235	0.16	€ 246,849	€ 493,699	€ 740,548	€ 987,398	-€ 5,968,745	-€ 11,937,491	-€ 17,906,236	-€ 23,874,981
2236	0.18	€ 288,659	€ 577,318	€ 865,977	##########	-€ 6,979,687	-€ 13,959,373	-€ 20,939,060	-€ 27,918,747
2237	0.23	€ 369,108	€ 738,216	##########	##########	-€ 8,924,919	-€ 17,849,838	-€ 26,774,756	-€ 35,699,675
2238	0.24	€ 384,837	€ 769,674	##########	##########	-€ 9,305,246	-€ 18,610,492	-€ 27,915,738	-€ 37,220,984
2239	0.18	€ 287,632	€ 575,263	€ 862,895	##########	-€ 6,954,846	-€ 13,909,693	-€ 20,864,539	-€ 27,819,385
2240	0.22	€ 342,376	€ 684,752	##########	##########	-€ 8,278,554	-€ 16,557,108	-€ 24,835,662	-€ 33,114,216

2241	0.20	€ 313,095	€ 626,191	€ 939,286	##########	-€ 7,570,551	-€ 15,141,102	-€ 22,711,654	-€ 30,282,205
2242	0.15	€ 242,789	€ 485,577	€ 728,366	€ 971,155	-€ 5,870,558	-€ 11,741,116	-€ 17,611,675	-€ 23,482,233
2243	0.20	€ 313,300	€ 626,600	€ 939,900	##########	-€ 7,575,503	-€ 15,151,006	-€ 22,726,510	-€ 30,302,013
2244	0.15	€ 237,779	€ 475,558	€ 713,337	€ 951,115	-€ 5,749,422	-€ 11,498,845	-€ 17,248,267	-€ 22,997,689
2245	0.12	€ 195,862	€ 391,725	€ 587,587	€ 783,449	-€ 4,735,892	-€ 9,471,784	-€ 14,207,676	-€ 18,943,568
2246	0.22	€ 341,725	€ 683,450	##########	##########	-€ 8,262,812	-€ 16,525,625	-€ 24,788,437	-€ 33,051,249
2247	0.17	€ 260,780	€ 521,560	€ 782,340	##########	-€ 6,305,586	-€ 12,611,172	-€ 18,916,758	-€ 25,222,344
2248	0.14	€ 224,422	€ 448,843	€ 673,265	€ 897,687	-€ 5,426,451	-€ 10,852,902	-€ 16,279,353	-€ 21,705,804
2249	0.26	€ 407,200	€ 814,401	##########	##########	-€ 9,845,986	-€ 19,691,972	-€ 29,537,958	-€ 39,383,944
2250	0.17	€ 270,827	€ 541,654	€ 812,482	##########	-€ 6,548,521	-€ 13,097,042	-€ 19,645,563	-€ 26,194,083
2251	0.21	€ 334,526	€ 669,053	##########	##########	-€ 8,088,748	-€ 16,177,496	-€ 24,266,244	-€ 32,354,992
2252	0.15	€ 230,246	€ 460,491	€ 690,737	€ 920,983	-€ 5,567,272	-€ 11,134,544	-€ 16,701,815	-€ 22,269,087
2253	0.15	€ 243,494	€ 486,987	€ 730,481	€ 973,974	-€ 5,887,602	-€ 11,775,203	-€ 17,662,805	-€ 23,550,407
2254	0.21	€ 323,503	€ 647,006	€ 970,509	##########	-€ 7,822,206	-€ 15,644,411	-€ 23,466,617	-€ 31,288,823
2255	0.18	€ 280,239	€ 560,478	€ 840,717	##########	-€ 6,776,094	-€ 13,552,188	-€ 20,328,281	-€ 27,104,375
2256	0.17	€ 265,248	€ 530,496	€ 795,745	##########	-€ 6,413,623	-€ 12,827,247	-€ 19,240,870	-€ 25,654,493
2257	0.13	€ 203,506	€ 407,012	€ 610,517	€ 814,023	-€ 4,920,709	-€ 9,841,417	-€ 14,762,126	-€ 19,682,835
2258	0.12	€ 189,812	€ 379,625	€ 569,437	€ 759,249	-€ 4,589,607	-€ 9,179,213	-€ 13,768,820	-€ 18,358,427
2259	0.13	€ 203,831	€ 407,662	€ 611,493	€ 815,323	-€ 4,928,570	-€ 9,857,139	-€ 14,785,709	-€ 19,714,279
2260	0.20	€ 314,611	€ 629,223	€ 943,834	##########	-€ 7,607,212	-€ 15,214,425	-€ 22,821,637	-€ 30,428,850
2261	0.13	€ 201,185	€ 402,371	€ 603,556	€ 804,742	-€ 4,864,605	-€ 9,729,210	-€ 14,593,815	-€ 19,458,420
2262	0.17	€ 270,978	€ 541,957	€ 812,935	##########	-€ 6,552,175	-€ 13,104,351	-€ 19,656,526	-€ 26,208,702
2263	0.16	€ 246,854	€ 493,707	€ 740,561	€ 987,415	-€ 5,968,850	-€ 11,937,700	-€ 17,906,549	-€ 23,875,399
2264	0.22	€ 339,021	€ 678,041	##########	##########	-€ 8,197,420	-€ 16,394,840	-€ 24,592,261	-€ 32,789,681
2265	0.18	€ 283,513	€ 567,026	€ 850,540	##########	-€ 6,855,265	-€ 13,710,530	-€ 20,565,795	-€ 27,421,061
2266	0.16	€ 247,233	€ 494,467	€ 741,700	€ 988,934	-€ 5,978,031	-€ 11,956,063	-€ 17,934,094	-€ 23,912,126
2267	0.19	€ 305,737	€ 611,473	€ 917,210	##########	-€ 7,392,620	-€ 14,785,239	-€ 22,177,859	-€ 29,570,479
2268	0.22	€ 346,464	€ 692,928	##########	##########	-€ 8,377,399	-€ 16,754,799	-€ 25,132,198	-€ 33,509,598
2269	0.23	€ 361,453	€ 722,905	##########	##########	-€ 8,739,816	-€ 17,479,632	-€ 26,219,449	-€ 34,959,265
2270	0.18	€ 279,842	€ 559,685	€ 839,527	##########	-€ 6,766,505	-€ 13,533,009	-€ 20,299,514	-€ 27,066,019
2271	0.19	€ 298,396	€ 596,791	€ 895,187	##########	-€ 7,215,116	-€ 14,430,233	-€ 21,645,349	-€ 28,860,466
2272	0.17	€ 264,414	€ 528,827	€ 793,241	##########	-€ 6,393,445	-€ 12,786,889	-€ 19,180,334	-€ 25,573,778
2273	0.21	€ 335,858	€ 671,716	##########	##########	-€ 8,120,943	-€ 16,241,886	-€ 24,362,830	-€ 32,483,773
2274	0.12	€ 183,859	€ 367,719	€ 551,578	€ 735,438	-€ 4,445,667	-€ 8,891,334	-€ 13,337,000	-€ 17,782,667
2275	0.15	€ 240,146	€ 480,292	€ 720,438	€ 960,584	-€ 5,806,662	-€ 11,613,323	-€ 17,419,985	-€ 23,226,647
2276	0.18	€ 278,846	€ 557,691	€ 836,537	##########	-€ 6,742,407	-€ 13,484,814	-€ 20,227,221	-€ 26,969,628
2277	0.21	€ 335,360	€ 670,720	##########	##########	-€ 8,108,911	-€ 16,217,822	-€ 24,326,734	-€ 32,435,645
2278	0.27	€ 421,259	€ 842,518	##########	##########	-€ 10,185,913	-€ 20,371,825	-€ 30,557,738	-€ 40,743,650
2279	0.17	€ 270,316	€ 540,632	€ 810,948	##########	-€ 6,536,158	-€ 13,072,316	-€ 19,608,474	-€ 26,144,632
2280	0.16	€ 256,172	€ 512,344	€ 768,516	##########	-€ 6,194,166	-€ 12,388,332	-€ 18,582,499	-€ 24,776,665
2281	0.14	€ 218,209	€ 436,418	€ 654,627	€ 872,837	-€ 5,276,232	-€ 10,552,464	-€ 15,828,697	-€ 21,104,929
2282	0.17	€ 274,964	€ 549,929	€ 824,893	##########	-€ 6,648,558	-€ 13,297,116	-€ 19,945,675	-€ 26,594,233
2283	0.26	€ 415,645	€ 831,291	##########	##########	-€ 10,050,179	-€ 20,100,359	-€ 30,150,538	-€ 40,200,718
2284	0.24	€ 378,655	€ 757,310	##########	##########	-€ 9,155,763	-€ 18,311,525	-€ 27,467,288	-€ 36,623,050
2285	0.26	€ 412,151	€ 824,301	##########	##########	-€ 9,965,680	-€ 19,931,359	-€ 29,897,039	-€ 39,862,718
2286	0.12	€ 186,240	€ 372,480	€ 558,721	€ 744,961	-€ 4,503,233	-€ 9,006,466	-€ 13,509,699	-€ 18,012,932
2287	0.26	€ 403,725	€ 807,450	##########	##########	-€ 9,761,951	-€ 19,523,902	-€ 29,285,852	-€ 39,047,803
2288	0.20	€ 319,250	€ 638,501	€ 957,751	##########	-€ 7,719,382	-€ 15,438,764	-€ 23,158,146	-€ 30,877,528
2289	0.14	€ 214,334	€ 428,668	€ 643,003	€ 857,337	-€ 5,182,538	-€ 10,365,077	-€ 15,547,615	-€ 20,730,154
2290	0.15	€ 231,150	€ 462,300	€ 693,450	€ 924,600	-€ 5,589,136	-€ 11,178,272	-€ 16,767,408	-€ 22,356,545
2291	0.22	€ 341,241	€ 682,483	##########	##########	-€ 8,251,117	-€ 16,502,234	-€ 24,753,351	-€ 33,004,468
2292	0.21	€ 338,221	€ 676,443	##########	##########	-€ 8,178,090	-€ 16,356,181	-€ 24,534,271	-€ 32,712,362
2293	0.18	€ 285,704	€ 571,408	€ 857,112	##########	-€ 6,908,240	-€ 13,816,480	-€ 20,724,719	-€ 27,632,959
2294	0.16	€ 249,746	€ 499,492	€ 749,238	€ 998,984	-€ 6,038,784	-€ 12,077,567	-€ 18,116,351	-€ 24,155,135
2295	0.24	€ 370,812	€ 741,625	##########	##########	-€ 8,966,131	-€ 17,932,262	-€ 26,898,393	-€ 35,864,524
2296	0.17	€ 266,973	€ 533,945	€ 800,918	##########	-€ 6,455,318	-€ 12,910,636	-€ 19,365,954	-€ 25,821,272
2297	0.28	€ 435,475	€ 870,950	##########	##########	-€ 10,529,653	-€ 21,059,307	-€ 31,588,960	-€ 42,118,614
2298	0.21	€ 329,695	€ 659,390	€ 989,085	##########	-€ 7,971,930	-€ 15,943,861	-€ 23,915,791	-€ 31,887,722
2299	0.24	€ 380,049	€ 760,097	##########	##########	-€ 9,189,465	-€ 18,378,929	-€ 27,568,394	-€ 36,757,859
2300	0.20	€ 319,577	€ 639,153	€ 958,730	##########	-€ 7,727,270	-€ 15,454,539	-€ 23,181,809	-€ 30,909,078
2301	0.16	€ 259,228	€ 518,456	€ 777,683	##########	-€ 6,268,050	-€ 12,536,100	-€ 18,804,150	-€ 25,072,200
2302	0.20	€ 317,716	€ 635,432	€ 953,148	##########	-€ 7,682,278	-€ 15,364,556	-€ 23,046,834	-€ 30,729,112
2303	0.12	€ 195,105	€ 390,210	€ 585,315	€ 780,420	-€ 4,717,581	-€ 9,435,161	-€ 14,152,742	-€ 18,870,322
2304	0.13	€ 210,354	€ 420,707	€ 631,061	€ 841,415	-€ 5,086,289	-€ 10,172,579	-€ 15,258,868	-€ 20,345,158
2305	0.15	€ 234,658	€ 469,316	€ 703,974	€ 938,632	-€ 5,673,958	-€ 11,347,916	-€ 17,021,875	-€ 22,695,833
2306	0.15	€ 228,609	€ 457,218	€ 685,826	€ 914,435	-€ 5,527,693	-€ 11,055,387	-€ 16,583,080	-€ 22,110,774
2307	0.15	€ 231,515	€ 463,031	€ 694,546	€ 926,062	-€ 5,597,974	-€ 11,195,948	-€ 16,793,922	-€ 22,391,896
2308	0.14	€ 223,977	€ 447,954	€ 671,931	€ 895,908	-€ 5,415,697	-€ 10,831,395	-€ 16,247,092	-€ 21,662,790
2309	0.19	€ 301,811	€ 603,621	€ 905,432	##########	-€ 7,297,691	-€ 14,595,382	-€ 21,893,073	-€ 29,190,764
2310	0.12	€ 190,694	€ 381,388	€ 572,082	€ 762,775	-€ 4,610,921	-€ 9,221,842	-€ 13,832,763	-€ 18,443,684
2311	0.18	€ 279,834	€ 559,668	€ 839,502	##########	-€ 6,766,302	-€ 13,532,603	-€ 20,298,905	-€ 27,065,207
2312	0.14	€ 216,828	€ 433,657	€ 650,485	€ 867,313	-€ 5,242,845	-€ 10,485,690	-€ 15,728,535	-€ 20,971,380
2313	0.24	€ 373,883	€ 747,766	##########	##########	-€ 9,040,385	-€ 18,080,769	-€ 27,121,154	-€ 36,161,539
2314	0.18	€ 277,326	€ 554,652	€ 831,977	##########	-€ 6,705,655	-€ 13,411,311	-€ 20,116,966	-€ 26,822,621
2315	0.17	€ 262,420	€ 524,839	€ 787,259	##########	-€ 6,345,230	-€ 12,690,459	-€ 19,035,689	-€ 25,380,918
2316	0.18	€ 283,030	€ 566,061	€ 849,091	##########	-€ 6,843,590	-€ 13,687,181	-€ 20,530,771	-€ 27,374,362
2317	0.23	€ 355,784	€ 711,568	##########	##########	-€ 8,602,750	-€ 17,205,501	-€ 25,808,251	-€ 34,411,001
2318	0.17	€ 271,942	€ 543,885	€ 815,827	##########	-€ 6,575,484	-€ 13,150,967	-€ 19,726,451	-€ 26,301,935
2319	0.13	€ 197,150	€ 394,301	€ 591,451	€ 788,601	-€ 4,767,036	-€ 9,534,072	-€ 14,301,108	-€ 19,068,144
2320	0.21	€ 329,965	€ 659,929	€ 989,894	##########	-€ 7,978,449	-€ 15,956,898	-€ 23,935,347	-€ 31,913,796
2321	0.15	€ 242,037	€ 484,074	€ 726,112	€ 968,149	-€ 5,852,387	-€ 11,704,774	-€ 17,557,162	-€ 23,409,549
2322	0.24	€ 382,978	€ 765,957	##########	##########	-€ 9,260,301	-€ 18,520,601	-€ 27,780,902	-€ 37,041,203
2323	0.17	€ 263,845	€ 527,690	€ 791,536	##########	-€ 6,379,699	-€ 12,759,397	-€ 19,139,096	-€ 25,518,795
2324	0.13	€ 206,315	€ 412,631	€ 618,946	€ 825,262	-€ 4,988,648	-€ 9,977,295	-€ 14,965,943	-€ 19,954,590
2325	0.18	€ 278,931	€ 557,862	€ 836,793	##########	-€ 6,744,472	-€ 13,488,945	-€ 20,233,417	-€ 26,977,890
2326	0.15	€ 239,911	€ 479,823	€ 719,734	€ 959,646	-€ 5,800,986	-€ 11,601,972	-€ 17,402,958	-€ 23,203,944
2327	0.19	€ 301,044	€ 602,088	€ 903,132	##########	-€ 7,279,154	-€ 14,558,308	-€ 21,837,463	-€ 29,116,617
2328	0.17	€ 268,461	€ 536,923	€ 805,384	##########	-€ 6,491,316	-€ 12,982,633	-€ 19,473,949	-€ 25,965,266
2329	0.15	€ 231,262	€ 462,525	€ 693,787	€ 925,050	-€ 5,591,857	-€ 11,183,713	-€ 16,775,570	-€ 22,367,426
2330	0.21	€ 326,661	€ 653,323	€ 979,984	##########	-€ 7,898,577	-€ 15,797,154	-€ 23,695,731	-€ 31,594,308
2331	0.16	€ 257,416	€ 514,832	€ 772,248	##########	-€ 6,224,241	-€ 12,448,483	-€ 18,672,724	-€ 24,896,965
2332	0.20	€ 319,207	€ 638,414	€ 957,620	##########	-€ 7,718,325	-€ 15,436,651	-€ 23,154,976	-€ 30,873,302
2333	0.23	€ 361,190	€ 722,379	##########	##########	-€ 8,733,459	-€ 17,466,918	-€ 26,200,377	-€ 34,933,836
2334	0.17	€ 274,598	€ 549,196	€ 823,794	##########	-€ 6,639,699	-€ 13,279,398	-€ 19,919,098	-€ 26,558,797
2335	0.18	€ 290,537	€ 581,075	€ 871,612	##########	-€ 7,025,107	-€ 14,050,214	-€ 21,075,321	-€ 28,100,428
2336	0.12	€ 184,129	€ 368,258	€ 552,386	€ 736,515	-€ 4,452,179	-€ 8,904,358	-€ 13,356,537	-€ 17,808,715
2337	0.25	€ 391,974	€ 783,949	##########	##########	-€ 9,477,825	-€ 18,955,651	-€ 28,433,476	-€ 37,911,302
2338	0.20	€ 308,621	€ 617,242	€ 925,864	##########	-€ 7,462,369	-€ 14,924,737	-€ 22,387,106	-€ 29,849,475
2339	0.20	€ 320,996	€ 641,993	€ 962,989	##########	-€ 7,761,598	-€ 15,523,196	-€ 23,284,795	-€ 31,046,393
2340	0.24	€ 382,408	€ 764,816	##########	##########	-€ 9,246,509	-€ 18,493,018	-€ 27,739,527	-€ 36,986,036
2341	0.14	€ 218,732	€ 437,464	€ 656,196	€ 874,929	-€ 5,288,878	-€ 10,577,757	-€ 15,866,635	-€ 21,155,514
2342	0.18	€ 279,265	€ 558,530	€ 837,795	##########	-€ 6,752,547	-€ 13,505,093	-€ 20,257,640	-€ 27,010,186

2343	0.18	€ 285,289	€ 570,579	€ 855,868	##########	-€ 6,898,214	-€ 13,796,428	-€ 20,694,642	-€ 27,592,856
2344	0.16	€ 257,202	€ 514,403	€ 771,605	##########	-€ 6,219,056	-€ 12,438,112	-€ 18,657,168	-€ 24,876,224
2345	0.18	€ 289,403	€ 578,806	€ 868,209	##########	-€ 6,997,679	-€ 13,995,358	-€ 20,993,037	-€ 27,990,717
2346	0.16	€ 249,639	€ 499,278	€ 748,917	€ 998,556	-€ 6,036,195	-€ 12,072,390	-€ 18,108,585	-€ 24,144,780
2347	0.12	€ 191,093	€ 382,186	€ 573,279	€ 764,372	-€ 4,620,571	-€ 9,241,142	-€ 13,861,713	-€ 18,482,284
2348	0.23	€ 358,406	€ 716,812	##########	##########	-€ 8,666,154	-€ 17,332,308	-€ 25,998,462	-€ 34,664,616
2349	0.21	€ 325,355	€ 650,710	€ 976,065	##########	-€ 7,866,987	-€ 15,733,974	-€ 23,600,960	-€ 31,467,947
2350	0.21	€ 324,329	€ 648,657	€ 972,986	##########	-€ 7,842,169	-€ 15,684,337	-€ 23,526,506	-€ 31,368,674
2351	0.20	€ 314,248	€ 628,495	€ 942,743	##########	-€ 7,598,415	-€ 15,196,829	-€ 22,795,244	-€ 30,393,659
2352	0.16	€ 245,247	€ 490,495	€ 735,742	€ 980,989	-€ 5,930,008	-€ 11,860,015	-€ 17,790,023	-€ 23,720,030
2353	0.26	€ 416,728	€ 833,456	##########	##########	-€ 10,076,362	-€ 20,152,724	-€ 30,229,086	-€ 40,305,447
2354	0.17	€ 273,126	€ 546,252	€ 819,377	##########	-€ 6,604,100	-€ 13,208,199	-€ 19,812,299	-€ 26,416,398
2355	0.14	€ 213,580	€ 427,160	€ 640,740	€ 854,320	-€ 5,164,298	-€ 10,328,597	-€ 15,492,895	-€ 20,657,193
2356	0.21	€ 325,697	€ 651,393	€ 977,090	##########	-€ 7,875,248	-€ 15,750,495	-€ 23,625,743	-€ 31,500,991
2357	0.14	€ 226,551	€ 453,102	€ 679,653	€ 906,204	-€ 5,477,935	-€ 10,955,870	-€ 16,433,806	-€ 21,911,741
2358	0.17	€ 272,709	€ 545,417	€ 818,126	##########	-€ 6,594,013	-€ 13,188,027	-€ 19,782,040	-€ 26,376,053
2359	0.22	€ 348,261	€ 696,522	##########	##########	-€ 8,420,853	-€ 16,841,707	-€ 25,262,560	-€ 33,683,413
2360	0.16	€ 247,969	€ 495,938	€ 743,908	€ 991,877	-€ 5,995,823	-€ 11,991,646	-€ 17,987,468	-€ 23,983,291
2361	0.19	€ 302,381	€ 604,762	€ 907,143	##########	-€ 7,311,479	-€ 14,622,958	-€ 21,934,437	-€ 29,245,916
2362	0.21	€ 325,407	€ 650,813	€ 976,220	##########	-€ 7,868,236	-€ 15,736,472	-€ 23,604,708	-€ 31,472,944
2363	0.26	€ 402,332	€ 804,663	##########	##########	-€ 9,728,262	-€ 19,456,524	-€ 29,184,786	-€ 38,913,048
2364	0.25	€ 398,982	€ 797,964	##########	##########	-€ 9,647,262	-€ 19,294,524	-€ 28,941,786	-€ 38,589,049
2365	0.15	€ 235,129	€ 470,258	€ 705,387	€ 940,516	-€ 5,685,350	-€ 11,370,699	-€ 17,056,049	-€ 22,741,398
2366	0.25	€ 390,197	€ 780,393	##########	##########	-€ 9,434,841	-€ 18,869,683	-€ 28,304,524	-€ 37,739,366
2367	0.22	€ 353,858	€ 707,716	##########	##########	-€ 8,556,185	-€ 17,112,371	-€ 25,668,556	-€ 34,224,741
2368	0.25	€ 390,505	€ 781,009	##########	##########	-€ 9,442,284	-€ 18,884,568	-€ 28,326,853	-€ 37,769,137
2369	0.14	€ 225,332	€ 450,665	€ 675,997	€ 901,329	-€ 5,448,468	-€ 10,896,935	-€ 16,345,403	-€ 21,793,870
2370	0.17	€ 261,568	€ 523,136	€ 784,704	##########	-€ 6,324,635	-€ 12,649,270	-€ 18,973,905	-€ 25,298,540
2371	0.20	€ 311,609	€ 623,219	€ 934,828	##########	-€ 7,534,625	-€ 15,069,249	-€ 22,603,874	-€ 30,138,499
2372	0.13	€ 208,645	€ 417,289	€ 625,934	€ 834,578	-€ 5,044,964	-€ 10,089,929	-€ 15,134,893	-€ 20,179,858
2373	0.20	€ 315,617	€ 631,234	€ 946,851	##########	-€ 7,631,527	-€ 15,263,055	-€ 22,894,582	-€ 30,526,109
2374	0.24	€ 385,708	€ 771,415	##########	##########	-€ 9,326,296	-€ 18,652,591	-€ 27,978,887	-€ 37,305,183
2375	0.19	€ 303,498	€ 606,995	€ 910,493	##########	-€ 7,338,482	-€ 14,676,964	-€ 22,015,447	-€ 29,353,929
2376	0.18	€ 291,021	€ 582,041	€ 873,062	##########	-€ 7,036,793	-€ 14,073,585	-€ 21,110,378	-€ 28,147,170
2377	0.16	€ 249,759	€ 499,518	€ 749,278	€ 999,037	-€ 6,039,104	-€ 12,078,207	-€ 18,117,311	-€ 24,156,415
2378	0.20	€ 314,268	€ 628,537	€ 942,805	##########	-€ 7,598,914	-€ 15,197,828	-€ 22,796,742	-€ 30,395,656
2379	0.15	€ 232,887	€ 465,774	€ 698,660	€ 931,547	-€ 5,631,133	-€ 11,262,266	-€ 16,893,399	-€ 22,524,532
2380	0.18	€ 286,481	€ 572,961	€ 859,442	##########	-€ 6,927,014	-€ 13,854,028	-€ 20,781,042	-€ 27,708,056
2381	0.25	€ 393,835	€ 787,670	##########	##########	-€ 9,522,818	-€ 19,045,636	-€ 28,568,454	-€ 38,091,272
2382	0.15	€ 239,597	€ 479,195	€ 718,792	€ 958,389	-€ 5,793,391	-€ 11,586,781	-€ 17,380,172	-€ 23,173,562
2383	0.18	€ 284,027	€ 568,054	€ 852,082	##########	-€ 6,867,695	-€ 13,735,389	-€ 20,603,084	-€ 27,470,778
2384	0.17	€ 263,902	€ 527,804	€ 791,706	##########	-€ 6,381,075	-€ 12,762,151	-€ 19,143,226	-€ 25,524,301
2385	0.19	€ 293,720	€ 587,439	€ 881,159	##########	-€ 7,102,054	-€ 14,204,107	-€ 21,306,161	-€ 28,408,214
2386	0.15	€ 231,939	€ 463,879	€ 695,818	€ 927,757	-€ 5,608,224	-€ 11,216,447	-€ 16,824,671	-€ 22,432,894
2387	0.18	€ 284,600	€ 569,200	€ 853,801	##########	-€ 6,881,548	-€ 13,763,097	-€ 20,644,645	-€ 27,526,194
2388	0.17	€ 262,859	€ 525,718	€ 788,576	##########	-€ 6,355,848	-€ 12,711,696	-€ 19,067,544	-€ 25,423,392
2389	0.16	€ 248,578	€ 497,156	€ 745,734	€ 994,311	-€ 6,010,539	-€ 12,021,078	-€ 18,031,617	-€ 24,042,156
2390	0.16	€ 244,840	€ 489,680	€ 734,519	€ 979,359	-€ 5,920,153	-€ 11,840,306	-€ 17,760,459	-€ 23,680,611
2391	0.15	€ 235,815	€ 471,631	€ 707,446	€ 943,261	-€ 5,701,946	-€ 11,403,891	-€ 17,105,837	-€ 22,807,782
2392	0.18	€ 286,632	€ 573,264	€ 859,897	##########	-€ 6,930,682	-€ 13,861,364	-€ 20,792,046	-€ 27,722,727
2393	0.25	€ 389,669	€ 779,337	##########	##########	-€ 9,422,074	-€ 18,844,148	-€ 28,266,222	-€ 37,688,295
2394	0.14	€ 219,611	€ 439,221	€ 658,832	€ 878,442	-€ 5,310,117	-€ 10,620,234	-€ 15,930,351	-€ 21,240,468
2395	0.20	€ 308,803	€ 617,605	€ 926,408	##########	-€ 7,466,756	-€ 14,933,511	-€ 22,400,267	-€ 29,867,022
2396	0.21	€ 327,094	€ 654,189	€ 981,283	##########	-€ 7,909,043	-€ 15,818,085	-€ 23,727,128	-€ 31,636,171
2397	0.12	€ 183,950	€ 367,900	€ 551,850	€ 735,800	-€ 4,447,858	-€ 8,895,716	-€ 13,343,575	-€ 17,791,433
2398	0.24	€ 375,349	€ 750,699	##########	##########	-€ 9,075,836	-€ 18,151,672	-€ 27,227,509	-€ 36,303,345
2399	0.16	€ 248,677	€ 497,354	€ 746,030	€ 994,707	-€ 6,012,932	-€ 12,025,864	-€ 18,038,796	-€ 24,051,727
2400	0.22	€ 344,869	€ 689,738	##########	##########	-€ 8,338,833	-€ 16,677,665	-€ 25,016,498	-€ 33,355,330
2401	0.14	€ 228,297	€ 456,595	€ 684,892	€ 913,189	-€ 5,520,161	-€ 11,040,323	-€ 16,560,484	-€ 22,080,645
2402	0.20	€ 320,977	€ 641,955	€ 962,932	##########	-€ 7,761,139	-€ 15,522,278	-€ 23,283,417	-€ 31,044,556
2403	0.17	€ 266,702	€ 533,403	€ 800,105	##########	-€ 6,448,764	-€ 12,897,528	-€ 19,346,292	-€ 25,795,056
2404	0.17	€ 266,280	€ 532,560	€ 798,840	##########	-€ 6,438,568	-€ 12,877,137	-€ 19,315,705	-€ 25,754,273
2405	0.20	€ 321,627	€ 643,254	€ 964,881	##########	-€ 7,776,848	-€ 15,553,696	-€ 23,330,543	-€ 31,107,391
2406	0.15	€ 231,689	€ 463,379	€ 695,068	€ 926,757	-€ 5,602,179	-€ 11,204,359	-€ 16,806,538	-€ 22,408,717
2407	0.19	€ 301,285	€ 602,571	€ 903,856	##########	-€ 7,284,990	-€ 14,569,980	-€ 21,854,970	-€ 29,139,959
2408	0.14	€ 219,599	€ 439,198	€ 658,797	€ 878,396	-€ 5,309,841	-€ 10,619,682	-€ 15,929,524	-€ 21,239,365
2409	0.23	€ 354,806	€ 709,612	##########	##########	-€ 8,579,110	-€ 17,158,219	-€ 25,737,329	-€ 34,316,438
2410	0.31	€ 490,266	€ 980,532	##########	##########	-€ 11,854,490	-€ 23,708,980	-€ 35,563,469	-€ 47,417,959
2411	0.16	€ 251,294	€ 502,589	€ 753,883	##########	-€ 6,076,223	-€ 12,152,446	-€ 18,228,669	-€ 24,304,892
2412	0.14	€ 220,186	€ 440,371	€ 660,557	€ 880,743	-€ 5,324,025	-€ 10,648,050	-€ 15,972,075	-€ 21,296,100
2413	0.20	€ 316,012	€ 632,023	€ 948,035	##########	-€ 7,641,069	-€ 15,282,139	-€ 22,923,208	-€ 30,564,278
2414	0.18	€ 287,146	€ 574,293	€ 861,439	##########	-€ 6,943,113	-€ 13,886,226	-€ 20,829,339	-€ 27,772,452
2415	0.25	€ 386,342	€ 772,685	##########	##########	-€ 9,341,646	-€ 18,683,292	-€ 28,024,938	-€ 37,366,584
2416	0.20	€ 308,820	€ 617,641	€ 926,461	##########	-€ 7,467,186	-€ 14,934,373	-€ 22,401,559	-€ 29,868,746
2417	0.18	€ 285,299	€ 570,597	€ 855,896	##########	-€ 6,898,437	-€ 13,796,873	-€ 20,695,310	-€ 27,593,747
2418	0.18	€ 279,818	€ 559,636	€ 839,454	##########	-€ 6,765,917	-€ 13,531,834	-€ 20,297,752	-€ 27,063,669
2419	0.17	€ 262,802	€ 525,605	€ 788,407	##########	-€ 6,354,481	-€ 12,708,962	-€ 19,063,443	-€ 25,417,924
2420	0.19	€ 298,465	€ 596,931	€ 895,396	##########	-€ 7,216,805	-€ 14,433,610	-€ 21,650,415	-€ 28,867,220
2421	0.18	€ 290,698	€ 581,395	€ 872,093	##########	-€ 7,028,980	-€ 14,057,960	-€ 21,086,941	-€ 28,115,921
2422	0.17	€ 263,189	€ 526,379	€ 789,568	##########	-€ 6,363,843	-€ 12,727,685	-€ 19,091,528	-€ 25,455,371
2423	0.19	€ 298,244	€ 596,488	€ 894,732	##########	-€ 7,211,451	-€ 14,422,903	-€ 21,634,354	-€ 28,845,805
2424	0.19	€ 306,631	€ 613,262	€ 919,893	##########	-€ 7,414,249	-€ 14,828,499	-€ 22,242,748	-€ 29,656,998
2425	0.25	€ 390,694	€ 781,389	##########	##########	-€ 9,446,873	-€ 18,893,747	-€ 28,340,620	-€ 37,787,494
2426	0.13	€ 200,286	€ 400,573	€ 600,859	€ 801,145	-€ 4,842,863	-€ 9,685,726	-€ 14,528,589	-€ 19,371,452
2427	0.19	€ 291,800	€ 583,599	€ 875,399	##########	-€ 7,055,631	-€ 14,111,262	-€ 21,166,893	-€ 28,222,524
2428	0.17	€ 262,162	€ 524,324	€ 786,487	##########	-€ 6,339,004	-€ 12,678,007	-€ 19,017,011	-€ 25,356,015
2429	0.14	€ 225,655	€ 451,310	€ 676,965	€ 902,620	-€ 5,456,274	-€ 10,912,548	-€ 16,368,821	-€ 21,825,095
2430	0.20	€ 321,822	€ 643,644	€ 965,465	##########	-€ 7,781,556	-€ 15,563,112	-€ 23,344,669	-€ 31,126,225
2431	0.15	€ 234,681	€ 469,361	€ 704,042	€ 938,722	-€ 5,674,507	-€ 11,349,014	-€ 17,023,522	-€ 22,698,029
2432	0.16	€ 258,156	€ 516,311	€ 774,467	##########	-€ 6,242,124	-€ 12,484,247	-€ 18,726,371	-€ 24,968,494
2433	0.14	€ 224,227	€ 448,455	€ 672,682	€ 896,909	-€ 5,421,750	-€ 10,843,500	-€ 16,265,250	-€ 21,687,000
2434	0.23	€ 369,738	€ 739,475	##########	##########	-€ 8,940,145	-€ 17,880,290	-€ 26,820,435	-€ 35,760,581
2435	0.24	€ 382,661	€ 765,323	##########	##########	-€ 9,252,638	-€ 18,505,276	-€ 27,757,914	-€ 37,010,551
2436	0.21	€ 338,393	€ 676,786	##########	##########	-€ 8,182,247	-€ 16,364,493	-€ 24,546,740	-€ 32,728,986
2437	0.14	€ 220,336	€ 440,672	€ 661,007	€ 881,343	-€ 5,327,655	-€ 10,655,310	-€ 15,982,965	-€ 21,310,620
2438	0.20	€ 318,105	€ 636,211	€ 954,316	##########	-€ 7,691,694	-€ 15,383,389	-€ 23,075,083	-€ 30,766,777
2439	0.15	€ 236,634	€ 473,269	€ 709,903	€ 946,537	-€ 5,721,747	-€ 11,443,494	-€ 17,165,241	-€ 22,886,988
2440	0.21	€ 337,051	€ 674,101	##########	##########	-€ 8,149,781	-€ 16,299,562	-€ 24,449,343	-€ 32,599,124
2441	0.14	€ 216,182	€ 432,363	€ 648,545	€ 864,726	-€ 5,227,206	-€ 10,454,412	-€ 15,681,617	-€ 20,908,823
2442	0.16	€ 258,757	€ 517,515	€ 776,272	##########	-€ 6,256,677	-€ 12,513,354	-€ 18,770,031	-€ 25,026,708
2443	0.18	€ 281,354	€ 562,708	€ 844,062	##########	-€ 6,803,056	-€ 13,606,111	-€ 20,409,167	-€ 27,212,222
2444	0.18	€ 283,969	€ 567,938	€ 851,907	##########	-€ 6,866,284	-€ 13,732,568	-€ 20,598,853	-€ 27,465,137

2445	0.18	€ 284,844	€ 569,687	€ 854,531	##########	-€ 6,887,433	-€ 13,774,866	-€ 20,662,299	-€ 27,549,732
2446	0.13	€ 205,480	€ 410,960	€ 616,440	€ 821,921	-€ 4,968,449	-€ 9,936,899	-€ 14,905,348	-€ 19,873,797
2447	0.19	€ 301,006	€ 602,013	€ 903,019	##########	-€ 7,278,245	-€ 14,556,490	-€ 21,834,734	-€ 29,112,979
2448	0.14	€ 217,576	€ 435,151	€ 652,727	€ 870,303	-€ 5,260,917	-€ 10,521,834	-€ 15,782,751	-€ 21,043,668
2449	0.15	€ 242,228	€ 484,456	€ 726,683	€ 968,911	-€ 5,856,997	-€ 11,713,994	-€ 17,570,991	-€ 23,427,988
2450	0.13	€ 206,828	€ 413,655	€ 620,483	€ 827,310	-€ 5,001,030	-€ 10,002,061	-€ 15,003,091	-€ 20,004,122
2451	0.20	€ 314,301	€ 628,603	€ 942,904	##########	-€ 7,599,717	-€ 15,199,434	-€ 22,799,151	-€ 30,398,868
2452	0.29	€ 463,384	€ 926,768	##########	##########	-€ 11,204,490	-€ 22,408,980	-€ 33,613,471	-€ 44,817,961
2453	0.26	€ 412,067	€ 824,134	##########	##########	-€ 9,963,657	-€ 19,927,314	-€ 29,890,971	-€ 39,854,628
2454	0.25	€ 389,207	€ 778,414	##########	##########	-€ 9,410,904	-€ 18,821,808	-€ 28,232,712	-€ 37,643,616
2455	0.18	€ 279,682	€ 559,365	€ 839,047	##########	-€ 6,762,635	-€ 13,525,270	-€ 20,287,906	-€ 27,050,541
2456	0.26	€ 416,568	€ 833,135	##########	##########	-€ 10,072,484	-€ 20,144,968	-€ 30,217,452	-€ 40,289,935
2457	0.15	€ 230,124	€ 460,248	€ 690,372	€ 920,496	-€ 5,564,329	-€ 11,128,658	-€ 16,692,986	-€ 22,257,315
2458	0.19	€ 299,574	€ 599,148	€ 898,722	##########	-€ 7,243,611	-€ 14,487,222	-€ 21,730,833	-€ 28,974,444
2459	0.19	€ 306,847	€ 613,693	€ 920,540	##########	-€ 7,419,461	-€ 14,838,921	-€ 22,258,382	-€ 29,677,842
2460	0.19	€ 296,228	€ 592,456	€ 888,684	##########	-€ 7,162,703	-€ 14,325,406	-€ 21,488,109	-€ 28,650,812
2461	0.13	€ 212,307	€ 424,614	€ 636,921	€ 849,228	-€ 5,133,519	-€ 10,267,039	-€ 15,400,558	-€ 20,534,077
2462	0.20	€ 320,323	€ 640,647	€ 960,970	##########	-€ 7,745,325	-€ 15,490,649	-€ 23,235,974	-€ 30,981,298
2463	0.25	€ 393,159	€ 786,317	##########	##########	-€ 9,506,460	-€ 19,012,921	-€ 28,519,381	-€ 38,025,842
2464	0.18	€ 278,492	€ 556,985	€ 835,477	##########	-€ 6,733,861	-€ 13,467,722	-€ 20,201,583	-€ 26,935,444
2465	0.13	€ 208,369	€ 416,738	€ 625,107	€ 833,476	-€ 5,038,301	-€ 10,076,602	-€ 15,114,903	-€ 20,153,204
2466	0.25	€ 398,625	€ 797,249	##########	##########	-€ 9,638,625	-€ 19,277,249	-€ 28,915,874	-€ 38,554,499
2467	0.16	€ 250,299	€ 500,598	€ 750,896	##########	-€ 6,052,150	-€ 12,104,299	-€ 18,156,449	-€ 24,208,598
2468	0.14	€ 214,879	€ 429,757	€ 644,636	€ 859,514	-€ 5,195,701	-€ 10,391,402	-€ 15,587,104	-€ 20,782,805
2469	0.15	€ 240,347	€ 480,694	€ 721,041	€ 961,388	-€ 5,811,521	-€ 11,623,043	-€ 17,434,564	-€ 23,246,085
2470	0.19	€ 302,695	€ 605,390	€ 908,085	##########	-€ 7,319,079	-€ 14,638,158	-€ 21,957,237	-€ 29,276,315
2471	0.18	€ 285,072	€ 570,143	€ 855,215	##########	-€ 6,892,945	-€ 13,785,889	-€ 20,678,834	-€ 27,571,779
2472	0.21	€ 329,837	€ 659,674	€ 989,511	##########	-€ 7,975,359	-€ 15,950,718	-€ 23,926,076	-€ 31,901,435
2473	0.14	€ 221,490	€ 442,980	€ 664,470	€ 885,960	-€ 5,355,563	-€ 10,711,126	-€ 16,066,689	-€ 21,422,252
2474	0.12	€ 186,231	€ 372,461	€ 558,692	€ 744,922	-€ 4,503,000	-€ 9,006,000	-€ 13,509,000	-€ 18,012,000
2475	0.27	€ 422,984	€ 845,968	##########	##########	-€ 10,227,633	-€ 20,455,266	-€ 30,682,899	-€ 40,910,531
2476	0.18	€ 285,660	€ 571,320	€ 856,980	##########	-€ 6,907,178	-€ 13,814,356	-€ 20,721,534	-€ 27,628,711
2477	0.19	€ 291,380	€ 582,760	€ 874,140	##########	-€ 7,045,480	-€ 14,090,961	-€ 21,136,441	-€ 28,181,921
2478	0.23	€ 358,406	€ 716,811	##########	##########	-€ 8,666,139	-€ 17,332,278	-€ 25,998,417	-€ 34,664,556
2479	0.18	€ 282,362	€ 564,724	€ 847,087	##########	-€ 6,827,434	-€ 13,654,869	-€ 20,482,303	-€ 27,309,738
2480	0.24	€ 373,197	€ 746,395	##########	##########	-€ 9,023,803	-€ 18,047,606	-€ 27,071,410	-€ 36,095,213
2481	0.21	€ 337,377	€ 674,753	##########	##########	-€ 8,157,664	-€ 16,315,328	-€ 24,472,991	-€ 32,630,655
2482	0.18	€ 284,835	€ 569,670	€ 854,506	##########	-€ 6,887,232	-€ 13,774,463	-€ 20,661,695	-€ 27,548,927
2483	0.20	€ 309,487	€ 618,974	€ 928,461	##########	-€ 7,483,307	-€ 14,966,614	-€ 22,449,921	-€ 29,933,227
2484	0.26	€ 403,347	€ 806,695	##########	##########	-€ 9,752,819	-€ 19,505,639	-€ 29,258,458	-€ 39,011,277
2485	0.16	€ 245,628	€ 491,256	€ 736,884	€ 982,512	-€ 5,939,211	-€ 11,878,423	-€ 17,817,634	-€ 23,756,846
2486	0.20	€ 309,653	€ 619,306	€ 928,959	##########	-€ 7,487,320	-€ 14,974,639	-€ 22,461,959	-€ 29,949,278
2487	0.16	€ 259,534	€ 519,068	€ 778,602	##########	-€ 6,275,454	-€ 12,550,909	-€ 18,826,363	-€ 25,101,817
2488	0.23	€ 361,825	€ 723,649	##########	##########	-€ 8,748,814	-€ 17,497,628	-€ 26,246,441	-€ 34,995,255
2489	0.17	€ 266,857	€ 533,714	€ 800,572	##########	-€ 6,452,529	-€ 12,905,058	-€ 19,357,587	-€ 25,810,116
2490	0.19	€ 305,755	€ 611,510	€ 917,264	##########	-€ 7,393,059	-€ 14,786,119	-€ 22,179,178	-€ 29,572,238
2491	0.18	€ 283,415	€ 566,831	€ 850,246	##########	-€ 6,852,899	-€ 13,705,798	-€ 20,558,696	-€ 27,411,595
2492	0.17	€ 267,027	€ 534,053	€ 801,080	##########	-€ 6,456,624	-€ 12,913,248	-€ 19,369,872	-€ 25,826,496
2493	0.22	€ 344,151	€ 688,302	##########	##########	-€ 8,321,471	-€ 16,642,942	-€ 24,964,413	-€ 33,285,883
2494	0.10	€ 156,084	€ 312,167	€ 468,251	€ 624,334	-€ 3,774,054	-€ 7,548,109	-€ 11,322,163	-€ 15,096,217
2495	0.15	€ 241,983	€ 483,967	€ 725,950	€ 967,934	-€ 5,851,088	-€ 11,702,176	-€ 17,553,264	-€ 23,404,352
2496	0.24	€ 382,930	€ 765,859	##########	##########	-€ 9,259,124	-€ 18,518,248	-€ 27,777,371	-€ 37,036,495
2497	0.26	€ 407,365	€ 814,729	##########	##########	-€ 9,849,953	-€ 19,699,907	-€ 29,549,860	-€ 39,399,814
2498	0.23	€ 361,423	€ 722,845	##########	##########	-€ 8,739,090	-€ 17,478,181	-€ 26,217,271	-€ 34,956,361
2499	0.14	€ 227,611	€ 455,222	€ 682,833	€ 910,444	-€ 5,503,568	-€ 11,007,136	-€ 16,510,704	-€ 22,014,272
2500	0.17	€ 263,391	€ 526,781	€ 790,172	##########	-€ 6,368,708	-€ 12,737,416	-€ 19,106,124	-€ 25,474,831
2501	0.20	€ 319,322	€ 638,644	€ 957,965	##########	-€ 7,721,106	-€ 15,442,212	-€ 23,163,318	-€ 30,884,424
2502	0.16	€ 256,887	€ 513,773	€ 770,660	##########	-€ 6,211,442	-€ 12,422,883	-€ 18,634,325	-€ 24,845,766
2503	0.23	€ 354,508	€ 709,015	##########	##########	-€ 8,571,886	-€ 17,143,773	-€ 25,715,659	-€ 34,287,546
2504	0.17	€ 272,462	€ 544,925	€ 817,387	##########	-€ 6,588,062	-€ 13,176,123	-€ 19,764,185	-€ 26,352,247
2505	0.20	€ 308,222	€ 616,443	€ 924,665	##########	-€ 7,452,710	-€ 14,905,419	-€ 22,358,129	-€ 29,810,839
2506	0.17	€ 270,809	€ 541,617	€ 812,426	##########	-€ 6,548,075	-€ 13,096,150	-€ 19,644,225	-€ 26,192,300
2507	0.23	€ 355,574	€ 711,147	##########	##########	-€ 8,597,663	-€ 17,195,327	-€ 25,792,990	-€ 34,390,654
2508	0.19	€ 303,245	€ 606,490	€ 909,734	##########	-€ 7,332,370	-€ 14,664,740	-€ 21,997,110	-€ 29,329,480
2509	0.18	€ 286,730	€ 573,460	€ 860,190	##########	-€ 6,933,046	-€ 13,866,092	-€ 20,799,138	-€ 27,732,184
2510	0.26	€ 411,838	€ 823,677	##########	##########	-€ 9,958,132	-€ 19,916,264	-€ 29,874,396	-€ 39,832,528
2511	0.21	€ 330,732	€ 661,465	€ 992,197	##########	-€ 7,997,011	-€ 15,994,021	-€ 23,991,032	-€ 31,988,043
2512	0.19	€ 299,263	€ 598,526	€ 897,790	##########	-€ 7,236,095	-€ 14,472,190	-€ 21,708,285	-€ 28,944,381
2513	0.21	€ 329,524	€ 659,049	€ 988,573	##########	-€ 7,967,803	-€ 15,935,605	-€ 23,903,408	-€ 31,871,211
2514	0.18	€ 281,976	€ 563,952	€ 845,929	##########	-€ 6,818,102	-€ 13,636,204	-€ 20,454,306	-€ 27,272,408
2515	0.20	€ 319,647	€ 639,294	€ 958,941	##########	-€ 7,728,971	-€ 15,457,942	-€ 23,186,913	-€ 30,915,884
2516	0.17	€ 266,962	€ 533,925	€ 800,887	##########	-€ 6,455,071	-€ 12,910,143	-€ 19,365,214	-€ 25,820,285
2517	0.20	€ 309,921	€ 619,842	€ 929,763	##########	-€ 7,493,799	-€ 14,987,598	-€ 22,481,398	-€ 29,975,197
2518	0.16	€ 246,419	€ 492,837	€ 739,256	€ 985,674	-€ 5,958,328	-€ 11,916,656	-€ 17,874,984	-€ 23,833,313
2519	0.17	€ 266,519	€ 533,037	€ 799,556	##########	-€ 6,444,341	-€ 12,888,683	-€ 19,333,024	-€ 25,777,366
2520	0.17	€ 269,051	€ 538,103	€ 807,154	##########	-€ 6,505,585	-€ 13,011,169	-€ 19,516,754	-€ 26,022,338
2521	0.24	€ 375,145	€ 750,289	##########	##########	-€ 9,070,886	-€ 18,141,772	-€ 27,212,658	-€ 36,283,545
2522	0.16	€ 248,808	€ 497,616	€ 746,425	€ 995,233	-€ 6,016,108	-€ 12,032,216	-€ 18,048,324	-€ 24,064,431
2523	0.23	€ 355,165	€ 710,330	##########	##########	-€ 8,587,788	-€ 17,175,575	-€ 25,763,363	-€ 34,351,151
2524	0.20	€ 310,842	€ 621,685	€ 932,527	##########	-€ 7,516,074	-€ 15,032,147	-€ 22,548,221	-€ 30,064,294
2525	0.19	€ 298,778	€ 597,557	€ 896,335	##########	-€ 7,224,374	-€ 14,448,747	-€ 21,673,121	-€ 28,897,495
2526	0.18	€ 282,091	€ 564,182	€ 846,273	##########	-€ 6,820,875	-€ 13,641,750	-€ 20,462,625	-€ 27,283,500
2527	0.13	€ 202,229	€ 404,457	€ 606,686	€ 808,914	-€ 4,889,827	-€ 9,779,654	-€ 14,669,481	-€ 19,559,308
2528	0.17	€ 265,698	€ 531,395	€ 797,093	##########	-€ 6,424,489	-€ 12,848,978	-€ 19,273,467	-€ 25,697,956
2529	0.22	€ 340,263	€ 680,526	##########	##########	-€ 8,227,452	-€ 16,454,905	-€ 24,682,357	-€ 32,909,810
2530	0.16	€ 251,407	€ 502,815	€ 754,222	##########	-€ 6,078,958	-€ 12,157,916	-€ 18,236,874	-€ 24,315,832
2531	0.21	€ 327,216	€ 654,433	€ 981,649	##########	-€ 7,911,998	-€ 15,823,996	-€ 23,735,993	-€ 31,647,991
2532	0.19	€ 296,850	€ 593,700	€ 890,550	##########	-€ 7,177,741	-€ 14,355,483	-€ 21,533,224	-€ 28,710,965
2533	0.15	€ 228,696	€ 457,393	€ 686,089	€ 914,786	-€ 5,529,813	-€ 11,059,625	-€ 16,589,438	-€ 22,119,251
2534	0.18	€ 280,545	€ 561,090	€ 841,635	##########	-€ 6,783,495	-€ 13,566,990	-€ 20,350,485	-€ 27,133,979
2535	0.11	€ 169,756	€ 339,512	€ 509,268	€ 679,024	-€ 4,104,647	-€ 8,209,294	-€ 12,313,942	-€ 16,418,589
2536	0.23	€ 360,331	€ 720,663	##########	##########	-€ 8,712,705	-€ 17,425,410	-€ 26,138,115	-€ 34,850,820
2537	0.20	€ 308,008	€ 616,017	€ 924,025	##########	-€ 7,447,553	-€ 14,895,105	-€ 22,342,658	-€ 29,790,210
2538	0.18	€ 291,018	€ 582,035	€ 873,053	##########	-€ 7,036,720	-€ 14,073,439	-€ 21,110,159	-€ 28,146,879
2539	0.17	€ 265,232	€ 530,463	€ 795,695	##########	-€ 6,413,222	-€ 12,826,443	-€ 19,239,665	-€ 25,652,886
2540	0.15	€ 229,106	€ 458,213	€ 687,319	€ 916,426	-€ 5,539,726	-€ 11,079,453	-€ 16,619,179	-€ 22,158,906
2541	0.24	€ 374,258	€ 748,515	##########	##########	-€ 9,049,437	-€ 18,098,874	-€ 27,148,310	-€ 36,197,747
2542	0.20	€ 319,289	€ 638,579	€ 957,868	##########	-€ 7,720,323	-€ 15,440,645	-€ 23,160,968	-€ 30,881,291
2543	0.21	€ 337,800	€ 675,601	##########	##########	-€ 8,167,912	-€ 16,335,823	-€ 24,503,735	-€ 32,671,646
2544	0.19	€ 304,217	€ 608,435	€ 912,652	##########	-€ 7,355,884	-€ 14,711,769	-€ 22,067,653	-€ 29,423,537
2545	0.22	€ 346,797	€ 693,595	##########	##########	-€ 8,385,459	-€ 16,770,918	-€ 25,156,378	-€ 33,541,837
2546	0.16	€ 254,306	€ 508,612	€ 762,919	##########	-€ 6,149,049	-€ 12,298,098	-€ 18,447,147	-€ 24,596,196

2547	0.20	€ 307,241	€ 614,482	€ 921,723	##########	-€ 7,428,998	-€ 14,857,996	-€ 22,286,995	-€ 29,715,993
2548	0.18	€ 286,607	€ 573,214	€ 859,821	##########	-€ 6,930,073	-€ 13,860,145	-€ 20,790,218	-€ 27,720,291
2549	0.22	€ 343,443	€ 686,885	##########	##########	-€ 8,304,343	-€ 16,608,686	-€ 24,913,029	-€ 33,217,371
2550	0.18	€ 276,590	€ 553,180	€ 829,771	##########	-€ 6,687,870	-€ 13,375,740	-€ 20,063,610	-€ 26,751,480
2551	0.19	€ 300,546	€ 601,091	€ 901,637	##########	-€ 7,267,105	-€ 14,534,209	-€ 21,801,314	-€ 29,068,419
2552	0.25	€ 388,274	€ 776,547	##########	##########	-€ 9,388,343	-€ 18,776,686	-€ 28,165,029	-€ 37,553,373
2553	0.15	€ 241,978	€ 483,956	€ 725,934	€ 967,913	-€ 5,850,960	-€ 11,701,919	-€ 17,552,879	-€ 23,403,839
2554	0.14	€ 221,189	€ 442,378	€ 663,567	€ 884,756	-€ 5,348,282	-€ 10,696,564	-€ 16,044,846	-€ 21,393,127
2555	0.23	€ 369,817	€ 739,634	##########	##########	-€ 8,942,067	-€ 17,884,135	-€ 26,826,202	-€ 35,768,269
2556	0.22	€ 348,358	€ 696,717	##########	##########	-€ 8,423,205	-€ 16,846,410	-€ 25,269,615	-€ 33,692,820
2557	0.23	€ 357,964	€ 715,928	##########	##########	-€ 8,655,459	-€ 17,310,917	-€ 25,966,376	-€ 34,621,835
2558	0.28	€ 434,461	€ 868,921	##########	##########	-€ 10,505,132	-€ 21,010,264	-€ 31,515,396	-€ 42,020,527
2559	0.27	€ 423,666	€ 847,333	##########	##########	-€ 10,244,127	-€ 20,488,253	-€ 30,732,380	-€ 40,976,507
2560	0.10	€ 151,215	€ 302,431	€ 453,646	€ 604,861	-€ 3,656,342	-€ 7,312,685	-€ 10,969,027	-€ 14,625,369
2561	0.20	€ 319,253	€ 638,505	€ 957,758	##########	-€ 7,719,432	-€ 15,438,864	-€ 23,158,296	-€ 30,877,728
2562	0.22	€ 340,680	€ 681,360	##########	##########	-€ 8,237,545	-€ 16,475,089	-€ 24,712,634	-€ 32,950,179
2563	0.16	€ 253,087	€ 506,175	€ 759,262	##########	-€ 6,119,575	-€ 12,239,151	-€ 18,358,726	-€ 24,478,302
2564	0.19	€ 296,326	€ 592,653	€ 888,979	##########	-€ 7,165,081	-€ 14,330,162	-€ 21,495,242	-€ 28,660,323
2565	0.18	€ 285,541	€ 571,082	€ 856,622	##########	-€ 6,904,292	-€ 13,808,584	-€ 20,712,876	-€ 27,617,168
2566	0.22	€ 348,405	€ 696,810	##########	##########	-€ 8,424,326	-€ 16,848,653	-€ 25,272,979	-€ 33,697,305
2567	0.20	€ 313,677	€ 627,354	€ 941,030	##########	-€ 7,584,612	-€ 15,169,224	-€ 22,753,835	-€ 30,338,447
2568	0.24	€ 385,316	€ 770,632	##########	##########	-€ 9,316,832	-€ 18,633,664	-€ 27,950,496	-€ 37,267,328
2569	0.19	€ 300,233	€ 600,466	€ 900,698	##########	-€ 7,259,539	-€ 14,519,078	-€ 21,778,617	-€ 29,038,156
2570	0.15	€ 240,007	€ 480,015	€ 720,022	€ 960,029	-€ 5,803,304	-€ 11,606,609	-€ 17,409,913	-€ 23,213,218
2571	0.14	€ 222,278	€ 444,556	€ 666,833	€ 889,111	-€ 5,374,611	-€ 10,749,222	-€ 16,123,834	-€ 21,498,445
2572	0.19	€ 305,697	€ 611,395	€ 917,092	##########	-€ 7,391,671	-€ 14,783,342	-€ 22,175,013	-€ 29,566,684
2573	0.21	€ 323,414	€ 646,828	€ 970,242	##########	-€ 7,820,058	-€ 15,640,115	-€ 23,460,173	-€ 31,280,230
2574	0.21	€ 332,835	€ 665,671	€ 998,506	##########	-€ 8,047,863	-€ 16,095,725	-€ 24,143,588	-€ 32,191,450
2575	0.17	€ 262,425	€ 524,849	€ 787,274	##########	-€ 6,345,352	-€ 12,690,703	-€ 19,036,055	-€ 25,381,407
2576	0.17	€ 273,214	€ 546,428	€ 819,641	##########	-€ 6,606,228	-€ 13,212,457	-€ 19,818,685	-€ 26,424,913
2577	0.14	€ 222,764	€ 445,529	€ 668,293	€ 891,057	-€ 5,386,374	-€ 10,772,748	-€ 16,159,122	-€ 21,545,496
2578	0.14	€ 212,580	€ 425,160	€ 637,740	€ 850,319	-€ 5,140,118	-€ 10,280,236	-€ 15,420,354	-€ 20,560,472
2579	0.20	€ 308,265	€ 616,530	€ 924,795	##########	-€ 7,453,759	-€ 14,907,518	-€ 22,361,277	-€ 29,815,036
2580	0.19	€ 294,569	€ 589,139	€ 883,708	##########	-€ 7,122,600	-€ 14,245,201	-€ 21,367,801	-€ 28,490,402
2581	0.21	€ 337,306	€ 674,612	##########	##########	-€ 8,155,956	-€ 16,311,912	-€ 24,467,868	-€ 32,623,824
2582	0.19	€ 299,644	€ 599,288	€ 898,933	##########	-€ 7,245,309	-€ 14,490,617	-€ 21,735,926	-€ 28,981,235
2583	0.11	€ 174,409	€ 348,817	€ 523,226	€ 697,634	-€ 4,217,147	-€ 8,434,294	-€ 12,651,441	-€ 16,868,587
2584	0.15	€ 231,912	€ 463,823	€ 695,735	€ 927,647	-€ 5,607,556	-€ 11,215,111	-€ 16,822,667	-€ 22,430,223
2585	0.18	€ 287,991	€ 575,982	€ 863,973	##########	-€ 6,963,539	-€ 13,927,078	-€ 20,890,617	-€ 27,854,155
2586	0.19	€ 294,669	€ 589,338	€ 884,007	##########	-€ 7,125,010	-€ 14,250,020	-€ 21,375,031	-€ 28,500,041
2587	0.17	€ 272,204	€ 544,408	€ 816,612	##########	-€ 6,581,813	-€ 13,163,626	-€ 19,745,438	-€ 26,327,251
2588	0.19	€ 293,379	€ 586,757	€ 880,136	##########	-€ 7,093,809	-€ 14,187,619	-€ 21,281,428	-€ 28,375,237
2589	0.13	€ 202,192	€ 404,385	€ 606,577	€ 808,770	-€ 4,888,953	-€ 9,777,906	-€ 14,666,858	-€ 19,555,811
2590	0.19	€ 294,243	€ 588,486	€ 882,728	##########	-€ 7,114,704	-€ 14,229,407	-€ 21,344,111	-€ 28,458,814
2591	0.16	€ 246,555	€ 493,110	€ 739,665	€ 986,220	-€ 5,961,625	-€ 11,923,250	-€ 17,884,875	-€ 23,846,501
2592	0.22	€ 339,338	€ 678,676	##########	##########	-€ 8,205,090	-€ 16,410,180	-€ 24,615,270	-€ 32,820,360
2593	0.17	€ 272,407	€ 544,813	€ 817,220	##########	-€ 6,586,710	-€ 13,173,421	-€ 19,760,131	-€ 26,346,841
2594	0.17	€ 268,266	€ 536,532	€ 804,799	##########	-€ 6,486,597	-€ 12,973,194	-€ 19,459,790	-€ 25,946,387
2595	0.17	€ 268,015	€ 536,030	€ 804,046	##########	-€ 6,480,528	-€ 12,961,056	-€ 19,441,584	-€ 25,922,111
2596	0.22	€ 346,574	€ 693,147	##########	##########	-€ 8,380,045	-€ 16,760,090	-€ 25,140,136	-€ 33,520,181
2597	0.19	€ 304,461	€ 608,922	€ 913,384	##########	-€ 7,361,781	-€ 14,723,563	-€ 22,085,344	-€ 29,447,125
2598	0.23	€ 360,088	€ 720,177	##########	##########	-€ 8,706,829	-€ 17,413,659	-€ 26,120,488	-€ 34,827,318
2599	0.13	€ 206,916	€ 413,831	€ 620,747	€ 827,662	-€ 5,003,158	-€ 10,006,316	-€ 15,009,473	-€ 20,012,631
2600	0.17	€ 269,769	€ 539,539	€ 809,308	##########	-€ 6,522,946	-€ 13,045,892	-€ 19,568,838	-€ 26,091,784
2601	0.19	€ 306,253	€ 612,507	€ 918,760	##########	-€ 7,405,116	-€ 14,810,231	-€ 22,215,347	-€ 29,620,463
2602	0.20	€ 321,327	€ 642,654	€ 963,981	##########	-€ 7,769,595	-€ 15,539,189	-€ 23,308,784	-€ 31,078,378
2603	0.14	€ 219,645	€ 439,290	€ 658,934	€ 878,579	-€ 5,310,946	-€ 10,621,891	-€ 15,932,837	-€ 21,243,783
2604	0.16	€ 255,112	€ 510,224	€ 765,336	##########	-€ 6,168,534	-€ 12,337,069	-€ 18,505,603	-€ 24,674,138
2605	0.19	€ 296,285	€ 592,569	€ 888,854	##########	-€ 7,164,076	-€ 14,328,152	-€ 21,492,228	-€ 28,656,304
2606	0.15	€ 243,423	€ 486,846	€ 730,269	€ 973,692	-€ 5,885,897	-€ 11,771,794	-€ 17,657,691	-€ 23,543,588
2607	0.21	€ 335,105	€ 670,211	##########	##########	-€ 8,102,746	-€ 16,205,491	-€ 24,308,237	-€ 32,410,983
2608	0.25	€ 392,122	€ 784,244	##########	##########	-€ 9,481,394	-€ 18,962,789	-€ 28,444,183	-€ 37,925,577
2609	0.17	€ 264,882	€ 529,764	€ 794,645	##########	-€ 6,404,763	-€ 12,809,526	-€ 19,214,288	-€ 25,619,051
2610	0.21	€ 335,729	€ 671,458	##########	##########	-€ 8,117,830	-€ 16,235,659	-€ 24,353,489	-€ 32,471,319
2611	0.22	€ 342,526	€ 685,052	##########	##########	-€ 8,282,179	-€ 16,564,357	-€ 24,846,536	-€ 33,128,714
2612	0.21	€ 325,277	€ 650,553	€ 975,830	##########	-€ 7,865,093	-€ 15,730,185	-€ 23,595,278	-€ 31,460,371
2613	0.23	€ 359,427	€ 718,854	##########	##########	-€ 8,690,833	-€ 17,381,665	-€ 26,072,498	-€ 34,763,331
2614	0.17	€ 272,959	€ 545,918	€ 818,877	##########	-€ 6,600,066	-€ 13,200,131	-€ 19,800,197	-€ 26,400,262
2615	0.23	€ 368,103	€ 736,206	##########	##########	-€ 8,900,622	-€ 17,801,244	-€ 26,701,866	-€ 35,602,488
2616	0.21	€ 325,142	€ 650,284	€ 975,427	##########	-€ 7,861,843	-€ 15,723,686	-€ 23,585,529	-€ 31,447,372
2617	0.16	€ 247,550	€ 495,099	€ 742,649	€ 990,198	-€ 5,985,676	-€ 11,971,352	-€ 17,957,028	-€ 23,942,704
2618	0.18	€ 286,735	€ 573,471	€ 860,206	##########	-€ 6,933,178	-€ 13,866,356	-€ 20,799,534	-€ 27,732,712
2619	0.20	€ 321,289	€ 642,577	€ 963,866	##########	-€ 7,768,661	-€ 15,537,322	-€ 23,305,983	-€ 31,074,644
2620	0.12	€ 187,973	€ 375,946	€ 563,920	€ 751,893	-€ 4,545,136	-€ 9,090,271	-€ 13,635,407	-€ 18,180,543
2621	0.19	€ 293,688	€ 587,377	€ 881,065	##########	-€ 7,101,297	-€ 14,202,595	-€ 21,303,892	-€ 28,405,189
2622	0.24	€ 378,389	€ 756,778	##########	##########	-€ 9,149,335	-€ 18,298,669	-€ 27,448,004	-€ 36,597,338
2623	0.23	€ 368,272	€ 736,543	##########	##########	-€ 8,904,701	-€ 17,809,402	-€ 26,714,103	-€ 35,618,804
2624	0.17	€ 261,527	€ 523,054	€ 784,580	##########	-€ 6,323,640	-€ 12,647,280	-€ 18,970,920	-€ 25,294,560
2625	0.13	€ 206,116	€ 412,231	€ 618,347	€ 824,463	-€ 4,983,817	-€ 9,967,634	-€ 14,951,451	-€ 19,935,268
2626	0.24	€ 370,868	€ 741,735	##########	##########	-€ 8,967,468	-€ 17,934,935	-€ 26,902,403	-€ 35,869,870
2627	0.17	€ 267,388	€ 534,776	€ 802,164	##########	-€ 6,465,359	-€ 12,930,717	-€ 19,396,076	-€ 25,861,435
2628	0.16	€ 249,158	€ 498,315	€ 747,473	€ 996,630	-€ 6,024,555	-€ 12,049,109	-€ 18,073,664	-€ 24,098,218
2629	0.17	€ 263,913	€ 527,826	€ 791,739	##########	-€ 6,381,338	-€ 12,762,676	-€ 19,144,014	-€ 25,525,352
2630	0.15	€ 230,303	€ 460,606	€ 690,908	€ 921,211	-€ 5,568,653	-€ 11,137,305	-€ 16,705,958	-€ 22,274,611
2631	0.17	€ 265,132	€ 530,263	€ 795,395	##########	-€ 6,410,803	-€ 12,821,606	-€ 19,232,409	-€ 25,643,212
2632	0.21	€ 325,936	€ 651,872	€ 977,809	##########	-€ 7,881,041	-€ 15,762,081	-€ 23,643,122	-€ 31,524,162
2633	0.21	€ 328,779	€ 657,557	€ 986,336	##########	-€ 7,949,770	-€ 15,899,539	-€ 23,849,309	-€ 31,799,078
2634	0.16	€ 254,046	€ 508,091	€ 762,137	##########	-€ 6,142,745	-€ 12,285,490	-€ 18,428,235	-€ 24,570,981
2635	0.18	€ 280,232	€ 560,463	€ 840,695	##########	-€ 6,775,918	-€ 13,551,836	-€ 20,327,754	-€ 27,103,672
2636	0.18	€ 284,354	€ 568,709	€ 853,063	##########	-€ 6,875,602	-€ 13,751,204	-€ 20,626,805	-€ 27,502,407
2637	0.20	€ 317,586	€ 635,173	€ 952,759	##########	-€ 7,679,146	-€ 15,358,291	-€ 23,037,437	-€ 30,716,582
2638	0.20	€ 317,663	€ 635,326	€ 952,990	##########	-€ 7,681,003	-€ 15,362,005	-€ 23,043,008	-€ 30,724,010
2639	0.14	€ 218,282	€ 436,564	€ 654,846	€ 873,128	-€ 5,277,995	-€ 10,555,990	-€ 15,833,985	-€ 21,111,979
2640	0.17	€ 265,152	€ 530,304	€ 795,456	##########	-€ 6,411,297	-€ 12,822,595	-€ 19,233,892	-€ 25,645,190
2641	0.25	€ 395,942	€ 791,885	##########	##########	-€ 9,573,770	-€ 19,147,541	-€ 28,721,311	-€ 38,295,081
2642	0.20	€ 309,302	€ 618,604	€ 927,906	##########	-€ 7,478,831	-€ 14,957,662	-€ 22,436,493	-€ 29,915,324
2643	0.24	€ 375,847	€ 751,695	##########	##########	-€ 9,087,877	-€ 18,175,755	-€ 27,263,632	-€ 36,351,510
2644	0.15	€ 240,948	€ 481,897	€ 722,845	€ 963,794	-€ 5,826,063	-€ 11,652,126	-€ 17,478,189	-€ 23,304,252
2645	0.27	€ 421,142	€ 842,284	##########	##########	-€ 10,183,084	-€ 20,366,168	-€ 30,549,252	-€ 40,732,337
2646	0.20	€ 313,037	€ 626,075	€ 939,112	##########	-€ 7,569,149	-€ 15,138,297	-€ 22,707,446	-€ 30,276,594
2647	0.17	€ 273,432	€ 546,864	€ 820,296	##########	-€ 6,611,505	-€ 13,223,010	-€ 19,834,515	-€ 26,446,019
2648	0.21	€ 328,596	€ 657,192	€ 985,787	##########	-€ 7,945,348	-€ 15,890,696	-€ 23,836,045	-€ 31,781,393

2649	0.22	€ 342,888	€ 685,777	##########	##########	-€ 8,290,937	-€ 16,581,873	-€ 24,872,810	-€ 33,163,747
2650	0.16	€ 244,254	€ 488,508	€ 732,761	€ 977,015	-€ 5,905,984	-€ 11,811,967	-€ 17,717,951	-€ 23,623,935
2651	0.25	€ 388,316	€ 776,632	##########	##########	-€ 9,389,361	-€ 18,778,723	-€ 28,168,084	-€ 37,557,445
2652	0.19	€ 297,018	€ 594,035	€ 891,053	##########	-€ 7,181,796	-€ 14,363,592	-€ 21,545,388	-€ 28,727,184
2653	0.17	€ 266,113	€ 532,225	€ 798,338	##########	-€ 6,434,527	-€ 12,869,053	-€ 19,303,580	-€ 25,738,107
2654	0.26	€ 401,810	€ 803,621	##########	##########	-€ 9,715,657	-€ 19,431,314	-€ 29,146,970	-€ 38,862,627
2655	0.14	€ 222,828	€ 445,656	€ 668,484	€ 891,312	-€ 5,387,916	-€ 10,775,832	-€ 16,163,747	-€ 21,551,663
2656	0.17	€ 268,469	€ 536,939	€ 805,408	##########	-€ 6,491,508	-€ 12,983,016	-€ 19,474,524	-€ 25,966,033
2657	0.16	€ 253,474	€ 506,948	€ 760,422	##########	-€ 6,128,930	-€ 12,257,859	-€ 18,386,789	-€ 24,515,719
2658	0.21	€ 333,864	€ 667,728	##########	##########	-€ 8,072,733	-€ 16,145,466	-€ 24,218,199	-€ 32,290,932
2659	0.20	€ 315,467	€ 630,934	€ 946,401	##########	-€ 7,627,900	-€ 15,255,801	-€ 22,883,701	-€ 30,511,601
2660	0.16	€ 253,672	€ 507,344	€ 761,016	##########	-€ 6,133,714	-€ 12,267,429	-€ 18,401,143	-€ 24,534,858
2661	0.14	€ 218,363	€ 436,725	€ 655,088	€ 873,450	-€ 5,279,943	-€ 10,559,886	-€ 15,839,829	-€ 21,119,771
2662	0.25	€ 395,291	€ 790,581	##########	##########	-€ 9,558,010	-€ 19,116,021	-€ 28,674,031	-€ 38,232,041
2663	0.16	€ 254,248	€ 508,497	€ 762,745	##########	-€ 6,147,650	-€ 12,295,300	-€ 18,442,949	-€ 24,590,599
2664	0.16	€ 246,978	€ 493,957	€ 740,935	€ 987,914	-€ 5,971,866	-€ 11,943,732	-€ 17,915,597	-€ 23,887,463
2665	0.15	€ 236,586	€ 473,172	€ 709,758	€ 946,344	-€ 5,720,579	-€ 11,441,159	-€ 17,161,738	-€ 22,882,318
2666	0.23	€ 366,434	€ 732,868	##########	##########	-€ 8,860,261	-€ 17,720,521	-€ 26,580,782	-€ 35,441,043
2667	0.21	€ 336,310	€ 672,620	##########	##########	-€ 8,131,877	-€ 16,263,755	-€ 24,395,632	-€ 32,527,510
2668	0.22	€ 348,103	€ 696,207	##########	##########	-€ 8,417,039	-€ 16,834,077	-€ 25,251,116	-€ 33,668,155
2669	0.15	€ 242,610	€ 485,220	€ 727,830	€ 970,440	-€ 5,866,236	-€ 11,732,471	-€ 17,598,707	-€ 23,464,943
2670	0.24	€ 370,356	€ 740,713	##########	##########	-€ 8,955,109	-€ 17,910,218	-€ 26,865,327	-€ 35,820,436
2671	0.18	€ 285,679	€ 571,359	€ 857,038	##########	-€ 6,907,642	-€ 13,815,284	-€ 20,722,925	-€ 27,630,567
2672	0.16	€ 251,638	€ 503,275	€ 754,913	##########	-€ 6,084,523	-€ 12,169,046	-€ 18,253,570	-€ 24,338,093
2673	0.17	€ 262,162	€ 524,324	€ 786,486	##########	-€ 6,339,002	-€ 12,678,005	-€ 19,017,007	-€ 25,356,010
2674	0.20	€ 313,795	€ 627,589	€ 941,384	##########	-€ 7,587,461	-€ 15,174,922	-€ 22,762,383	-€ 30,349,844
2675	0.26	€ 403,609	€ 807,217	##########	##########	-€ 9,759,137	-€ 19,518,273	-€ 29,277,410	-€ 39,036,547
2676	0.16	€ 257,712	€ 515,425	€ 773,137	##########	-€ 6,231,407	-€ 12,462,815	-€ 18,694,222	-€ 24,925,629
2677	0.23	€ 362,881	€ 725,761	##########	##########	-€ 8,774,347	-€ 17,548,695	-€ 26,323,042	-€ 35,097,390
2678	0.24	€ 382,696	€ 765,392	##########	##########	-€ 9,253,479	-€ 18,506,957	-€ 27,760,436	-€ 37,013,914
2679	0.17	€ 261,421	€ 522,841	€ 784,262	##########	-€ 6,321,071	-€ 12,642,142	-€ 18,963,213	-€ 25,284,283
2680	0.17	€ 267,481	€ 534,961	€ 802,442	##########	-€ 6,467,601	-€ 12,935,203	-€ 19,402,804	-€ 25,870,405
2681	0.17	€ 264,973	€ 529,946	€ 794,920	##########	-€ 6,406,974	-€ 12,813,948	-€ 19,220,923	-€ 25,627,897
2682	0.26	€ 401,770	€ 803,540	##########	##########	-€ 9,714,684	-€ 19,429,368	-€ 29,144,052	-€ 38,858,736
2683	0.21	€ 334,019	€ 668,038	##########	##########	-€ 8,076,485	-€ 16,152,970	-€ 24,229,455	-€ 32,305,940
2684	0.12	€ 186,679	€ 373,358	€ 560,038	€ 746,717	-€ 4,513,849	-€ 9,027,697	-€ 13,541,546	-€ 18,055,395
2685	0.13	€ 200,896	€ 401,793	€ 602,689	€ 803,585	-€ 4,857,615	-€ 9,715,229	-€ 14,572,844	-€ 19,430,458
2686	0.19	€ 300,010	€ 600,020	€ 900,030	##########	-€ 7,254,157	-€ 14,508,313	-€ 21,762,470	-€ 29,016,627
2687	0.21	€ 337,917	€ 675,835	##########	##########	-€ 8,170,744	-€ 16,341,488	-€ 24,512,232	-€ 32,682,976
2688	0.16	€ 251,597	€ 503,194	€ 754,791	##########	-€ 6,083,543	-€ 12,167,086	-€ 18,250,629	-€ 24,334,172
2689	0.22	€ 343,853	€ 687,707	##########	##########	-€ 8,314,275	-€ 16,628,549	-€ 24,942,824	-€ 33,257,098
2690	0.19	€ 300,905	€ 601,811	€ 902,716	##########	-€ 7,275,804	-€ 14,551,608	-€ 21,827,413	-€ 29,103,217
2691	0.17	€ 268,377	€ 536,754	€ 805,131	##########	-€ 6,489,276	-€ 12,978,552	-€ 19,467,828	-€ 25,957,104
2692	0.13	€ 200,423	€ 400,847	€ 601,270	€ 801,694	-€ 4,846,180	-€ 9,692,361	-€ 14,538,541	-€ 19,384,721
2693	0.11	€ 171,359	€ 342,717	€ 514,076	€ 685,434	-€ 4,143,398	-€ 8,286,797	-€ 12,430,195	-€ 16,573,593
2694	0.16	€ 248,446	€ 496,892	€ 745,338	€ 993,783	-€ 6,007,347	-€ 12,014,694	-€ 18,022,041	-€ 24,029,388
2695	0.31	€ 481,738	€ 963,476	##########	##########	-€ 11,648,287	-€ 23,296,573	-€ 34,944,860	-€ 46,593,147
2696	0.19	€ 296,170	€ 592,340	€ 888,510	##########	-€ 7,161,299	-€ 14,322,599	-€ 21,483,898	-€ 28,645,198
2697	0.14	€ 223,412	€ 446,825	€ 670,237	€ 893,650	-€ 5,402,046	-€ 10,804,093	-€ 16,206,139	-€ 21,608,185
2698	0.17	€ 275,090	€ 550,181	€ 825,271	##########	-€ 6,651,604	-€ 13,303,207	-€ 19,954,811	-€ 26,606,414
2699	0.17	€ 272,725	€ 545,451	€ 818,176	##########	-€ 6,594,419	-€ 13,188,839	-€ 19,783,258	-€ 26,377,677
2700	0.19	€ 291,732	€ 583,463	€ 875,195	##########	-€ 7,053,982	-€ 14,107,965	-€ 21,161,947	-€ 28,215,929
2701	0.26	€ 404,499	€ 808,997	##########	##########	-€ 9,780,657	-€ 19,561,313	-€ 29,341,970	-€ 39,122,627
2702	0.24	€ 370,540	€ 741,079	##########	##########	-€ 8,959,536	-€ 17,919,072	-€ 26,878,609	-€ 35,838,145
2703	0.17	€ 263,395	€ 526,791	€ 790,186	##########	-€ 6,368,819	-€ 12,737,639	-€ 19,106,458	-€ 25,475,278
2704	0.21	€ 331,077	€ 662,155	€ 993,232	##########	-€ 8,005,353	-€ 16,010,707	-€ 24,016,060	-€ 32,021,413
2705	0.24	€ 370,336	€ 740,671	##########	##########	-€ 8,954,608	-€ 17,909,217	-€ 26,863,825	-€ 35,818,434
2706	0.20	€ 307,117	€ 614,235	€ 921,352	##########	-€ 7,426,007	-€ 14,852,015	-€ 22,278,022	-€ 29,704,030
2707	0.17	€ 275,017	€ 550,035	€ 825,052	##########	-€ 6,649,840	-€ 13,299,679	-€ 19,949,519	-€ 26,599,358
2708	0.13	€ 207,474	€ 414,947	€ 622,421	€ 829,894	-€ 5,016,649	-€ 10,033,298	-€ 15,049,947	-€ 20,066,595
2709	0.18	€ 281,752	€ 563,504	€ 845,256	##########	-€ 6,812,679	-€ 13,625,358	-€ 20,438,038	-€ 27,250,717
2710	0.26	€ 409,058	€ 818,117	##########	##########	-€ 9,890,911	-€ 19,781,822	-€ 29,672,733	-€ 39,563,644
2711	0.19	€ 297,701	€ 595,402	€ 893,104	##########	-€ 7,198,326	-€ 14,396,653	-€ 21,594,979	-€ 28,793,306
2712	0.17	€ 263,041	€ 526,082	€ 789,123	##########	-€ 6,360,251	-€ 12,720,502	-€ 19,080,753	-€ 25,441,004
2713	0.26	€ 416,890	€ 833,779	##########	##########	-€ 10,080,267	-€ 20,160,535	-€ 30,240,802	-€ 40,321,070
2714	0.20	€ 309,803	€ 619,606	€ 929,409	##########	-€ 7,490,946	-€ 14,981,891	-€ 22,472,837	-€ 29,963,783
2715	0.12	€ 182,953	€ 365,907	€ 548,860	€ 731,813	-€ 4,423,756	-€ 8,847,512	-€ 13,271,268	-€ 17,695,024
2716	0.19	€ 296,781	€ 593,562	€ 890,344	##########	-€ 7,176,082	-€ 14,352,164	-€ 21,528,247	-€ 28,704,329
2717	0.19	€ 306,216	€ 612,431	€ 918,647	##########	-€ 7,404,203	-€ 14,808,406	-€ 22,212,609	-€ 29,616,812
2718	0.24	€ 371,952	€ 743,904	##########	##########	-€ 8,993,693	-€ 17,987,386	-€ 26,981,079	-€ 35,974,771
2719	0.18	€ 280,161	€ 560,322	€ 840,482	##########	-€ 6,774,204	-€ 13,548,408	-€ 20,322,612	-€ 27,096,816
2720	0.11	€ 178,851	€ 357,701	€ 536,552	€ 715,403	-€ 4,324,556	-€ 8,649,111	-€ 12,973,667	-€ 17,298,223
2721	0.20	€ 321,800	€ 643,600	€ 965,400	##########	-€ 7,781,028	-€ 15,562,056	-€ 23,343,085	-€ 31,124,113
2722	0.16	€ 259,272	€ 518,544	€ 777,816	##########	-€ 6,269,119	-€ 12,538,239	-€ 18,807,358	-€ 25,076,477
2723	0.25	€ 400,005	€ 800,010	##########	##########	-€ 9,672,007	-€ 19,344,015	-€ 29,016,022	-€ 38,688,029
2724	0.21	€ 324,709	€ 649,418	€ 974,127	##########	-€ 7,851,366	-€ 15,702,731	-€ 23,554,097	-€ 31,405,462
2725	0.21	€ 335,845	€ 671,690	##########	##########	-€ 8,120,633	-€ 16,241,266	-€ 24,361,899	-€ 32,482,532
2726	0.18	€ 281,122	€ 562,245	€ 843,367	##########	-€ 6,797,454	-€ 13,594,907	-€ 20,392,361	-€ 27,189,814
2727	0.22	€ 339,090	€ 678,180	##########	##########	-€ 8,199,100	-€ 16,398,200	-€ 24,597,300	-€ 32,796,400
2728	0.20	€ 312,691	€ 625,382	€ 938,074	##########	-€ 7,560,781	-€ 15,121,563	-€ 22,682,344	-€ 30,243,125
2729	0.17	€ 269,128	€ 538,255	€ 807,383	##########	-€ 6,507,424	-€ 13,014,847	-€ 19,522,271	-€ 26,029,695
2730	0.24	€ 376,082	€ 752,163	##########	##########	-€ 9,093,539	-€ 18,187,078	-€ 27,280,617	-€ 36,374,157
2731	0.18	€ 291,245	€ 582,490	€ 873,736	##########	-€ 7,042,222	-€ 14,084,445	-€ 21,126,667	-€ 28,168,890
2732	0.22	€ 344,286	€ 688,572	##########	##########	-€ 8,324,736	-€ 16,649,473	-€ 24,974,209	-€ 33,298,946
2733	0.21	€ 337,559	€ 675,118	##########	##########	-€ 8,162,074	-€ 16,324,148	-€ 24,486,222	-€ 32,648,296
2734	0.17	€ 260,640	€ 521,281	€ 781,921	##########	-€ 6,302,205	-€ 12,604,410	-€ 18,906,615	-€ 25,208,820
2735	0.17	€ 270,565	€ 541,130	€ 811,695	##########	-€ 6,542,182	-€ 13,084,364	-€ 19,626,545	-€ 26,168,727
2736	0.10	€ 151,531	€ 303,062	€ 454,593	€ 606,124	-€ 3,663,972	-€ 7,327,945	-€ 10,991,917	-€ 14,655,889
2737	0.14	€ 224,685	€ 449,371	€ 674,056	€ 898,741	-€ 5,432,824	-€ 10,865,648	-€ 16,298,472	-€ 21,731,296
2738	0.21	€ 333,595	€ 667,189	##########	##########	-€ 8,066,218	-€ 16,132,436	-€ 24,198,653	-€ 32,264,871
2739	0.32	€ 496,289	€ 992,578	##########	##########	-€ 12,000,125	-€ 24,000,251	-€ 36,000,376	-€ 48,000,502
2740	0.15	€ 230,479	€ 460,957	€ 691,436	€ 921,914	-€ 5,572,902	-€ 11,145,805	-€ 16,718,707	-€ 22,291,610
2741	0.19	€ 299,113	€ 598,227	€ 897,340	##########	-€ 7,232,475	-€ 14,464,950	-€ 21,697,425	-€ 28,929,900
2742	0.26	€ 410,440	€ 820,879	##########	##########	-€ 9,924,308	-€ 19,848,616	-€ 29,772,924	-€ 39,697,232
2743	0.20	€ 310,657	€ 621,314	€ 931,971	##########	-€ 7,511,591	-€ 15,023,182	-€ 22,534,773	-€ 30,046,364
2744	0.19	€ 303,571	€ 607,141	€ 910,712	##########	-€ 7,340,247	-€ 14,680,494	-€ 22,020,741	-€ 29,360,987
2745	0.17	€ 263,507	€ 527,014	€ 790,521	##########	-€ 6,371,523	-€ 12,743,047	-€ 19,114,570	-€ 25,486,093
2746	0.15	€ 241,428	€ 482,857	€ 724,285	€ 965,714	-€ 5,837,667	-€ 11,675,333	-€ 17,513,000	-€ 23,350,666
2747	0.16	€ 253,457	€ 506,914	€ 760,371	##########	-€ 6,128,519	-€ 12,257,038	-€ 18,385,557	-€ 24,514,076
2748	0.17	€ 259,995	€ 519,990	€ 779,985	##########	-€ 6,286,602	-€ 12,573,204	-€ 18,859,806	-€ 25,146,407
2749	0.22	€ 343,825	€ 687,651	##########	##########	-€ 8,313,597	-€ 16,627,194	-€ 24,940,792	-€ 33,254,389
2750	0.19	€ 303,394	€ 606,788	€ 910,182	##########	-€ 7,335,978	-€ 14,671,956	-€ 22,007,934	-€ 29,343,912

2751	0.16	€ 258,868	€ 517,735	€ 776,603	##########	-€ 6,259,341	-€ 12,518,682	-€ 18,778,022	-€ 25,037,363
2752	0.25	€ 385,922	€ 771,844	##########	##########	-€ 9,331,484	-€ 18,662,967	-€ 27,994,451	-€ 37,325,935
2753	0.16	€ 257,551	€ 515,103	€ 772,654	##########	-€ 6,227,516	-€ 12,455,032	-€ 18,682,548	-€ 24,910,064
2754	0.15	€ 236,987	€ 473,975	€ 710,962	€ 947,949	-€ 5,730,284	-€ 11,460,567	-€ 17,190,851	-€ 22,921,134
2755	0.17	€ 261,310	€ 522,619	€ 783,929	##########	-€ 6,318,391	-€ 12,636,781	-€ 18,955,172	-€ 25,273,562
2756	0.12	€ 191,113	€ 382,227	€ 573,340	€ 764,453	-€ 4,621,063	-€ 9,242,125	-€ 13,863,188	-€ 18,484,251
2757	0.20	€ 316,765	€ 633,530	€ 950,294	##########	-€ 7,659,280	-€ 15,318,559	-€ 22,977,839	-€ 30,637,118
2758	0.18	€ 281,344	€ 562,687	€ 844,031	##########	-€ 6,802,807	-€ 13,605,614	-€ 20,408,422	-€ 27,211,229
2759	0.25	€ 393,588	€ 787,176	##########	##########	-€ 9,516,836	-€ 19,033,671	-€ 28,550,507	-€ 38,067,343
2760	0.25	€ 393,473	€ 786,945	##########	##########	-€ 9,514,051	-€ 19,028,102	-€ 28,542,154	-€ 38,056,205
2761	0.25	€ 386,018	€ 772,036	##########	##########	-€ 9,333,797	-€ 18,667,595	-€ 28,001,392	-€ 37,335,190
2762	0.16	€ 254,059	€ 508,117	€ 762,176	##########	-€ 6,143,060	-€ 12,286,120	-€ 18,429,181	-€ 24,572,241
2763	0.18	€ 289,261	€ 578,522	€ 867,784	##########	-€ 6,994,250	-€ 13,988,499	-€ 20,982,749	-€ 27,976,999
2764	0.17	€ 261,815	€ 523,630	€ 785,446	##########	-€ 6,330,613	-€ 12,661,226	-€ 18,991,840	-€ 25,322,453
2765	0.19	€ 306,193	€ 612,385	€ 918,578	##########	-€ 7,403,645	-€ 14,807,289	-€ 22,210,934	-€ 29,614,578
2766	0.20	€ 317,780	€ 635,560	€ 953,341	##########	-€ 7,683,830	-€ 15,367,661	-€ 23,051,491	-€ 30,735,322
2767	0.21	€ 329,067	€ 658,134	€ 987,202	##########	-€ 7,956,747	-€ 15,913,494	-€ 23,870,241	-€ 31,826,988
2768	0.20	€ 309,404	€ 618,809	€ 928,213	##########	-€ 7,481,308	-€ 14,962,615	-€ 22,443,923	-€ 29,925,230
2769	0.19	€ 298,705	€ 597,409	€ 896,114	##########	-€ 7,222,588	-€ 14,445,176	-€ 21,667,763	-€ 28,890,351
2770	0.18	€ 276,729	€ 553,458	€ 830,188	##########	-€ 6,691,231	-€ 13,382,461	-€ 20,073,692	-€ 26,764,922
2771	0.21	€ 336,180	€ 672,360	##########	##########	-€ 8,128,732	-€ 16,257,463	-€ 24,386,195	-€ 32,514,926
2772	0.18	€ 282,801	€ 565,603	€ 848,404	##########	-€ 6,838,052	-€ 13,676,105	-€ 20,514,157	-€ 27,352,209
2773	0.24	€ 372,020	€ 744,040	##########	##########	-€ 8,995,330	-€ 17,990,661	-€ 26,985,991	-€ 35,981,322
2774	0.11	€ 166,924	€ 333,848	€ 500,772	€ 667,696	-€ 4,036,172	-€ 8,072,344	-€ 12,108,516	-€ 16,144,688
2775	0.19	€ 297,731	€ 595,462	€ 893,193	##########	-€ 7,199,046	-€ 14,398,092	-€ 21,597,138	-€ 28,796,184
2776	0.15	€ 228,684	€ 457,367	€ 686,051	€ 914,735	-€ 5,529,504	-€ 11,059,009	-€ 16,588,513	-€ 22,118,018
2777	0.13	€ 212,375	€ 424,749	€ 637,124	€ 849,498	-€ 5,135,154	-€ 10,270,307	-€ 15,405,461	-€ 20,540,614
2778	0.19	€ 301,209	€ 602,419	€ 903,628	##########	-€ 7,283,155	-€ 14,566,309	-€ 21,849,464	-€ 29,132,619
2779	0.20	€ 313,707	€ 627,415	€ 941,122	##########	-€ 7,585,353	-€ 15,170,706	-€ 22,756,060	-€ 30,341,413
2780	0.20	€ 308,468	€ 616,937	€ 925,405	##########	-€ 7,458,671	-€ 14,917,342	-€ 22,376,014	-€ 29,834,685
2781	0.19	€ 298,885	€ 597,770	€ 896,655	##########	-€ 7,226,953	-€ 14,453,905	-€ 21,680,858	-€ 28,907,810
2782	0.22	€ 342,212	€ 684,425	##########	##########	-€ 8,274,593	-€ 16,549,187	-€ 24,823,780	-€ 33,098,374
2783	0.13	€ 199,456	€ 398,912	€ 598,368	€ 797,824	-€ 4,822,788	-€ 9,645,577	-€ 14,468,365	-€ 19,291,154
2784	0.26	€ 401,811	€ 803,621	##########	##########	-€ 9,715,660	-€ 19,431,319	-€ 29,146,979	-€ 38,862,638
2785	0.22	€ 349,641	€ 699,283	##########	##########	-€ 8,454,222	-€ 16,908,444	-€ 25,362,666	-€ 33,816,887
2786	0.16	€ 259,270	€ 518,539	€ 777,809	##########	-€ 6,269,061	-€ 12,538,121	-€ 18,807,182	-€ 25,076,242
2787	0.16	€ 252,396	€ 504,793	€ 757,189	##########	-€ 6,102,871	-€ 12,205,741	-€ 18,308,612	-€ 24,411,482
2788	0.22	€ 347,944	€ 695,888	##########	##########	-€ 8,413,186	-€ 16,826,371	-€ 25,239,557	-€ 33,652,742
2789	0.23	€ 366,249	€ 732,498	##########	##########	-€ 8,855,794	-€ 17,711,587	-€ 26,567,381	-€ 35,423,175
2790	0.20	€ 317,250	€ 634,500	€ 951,750	##########	-€ 7,671,009	-€ 15,342,019	-€ 23,013,028	-€ 30,684,038
2791	0.21	€ 338,066	€ 676,131	##########	##########	-€ 8,174,329	-€ 16,348,659	-€ 24,522,988	-€ 32,697,318
2792	0.20	€ 312,554	€ 625,109	€ 937,663	##########	-€ 7,557,474	-€ 15,114,947	-€ 22,672,421	-€ 30,229,895
2793	0.22	€ 342,799	€ 685,597	##########	##########	-€ 8,288,768	-€ 16,577,535	-€ 24,866,303	-€ 33,155,071
2794	0.25	€ 396,359	€ 792,719	##########	##########	-€ 9,583,852	-€ 19,167,705	-€ 28,751,557	-€ 38,335,410
2795	0.24	€ 382,597	€ 765,195	##########	##########	-€ 9,251,088	-€ 18,502,177	-€ 27,753,265	-€ 37,004,353
2796	0.14	€ 220,494	€ 440,989	€ 661,483	€ 881,977	-€ 5,331,486	-€ 10,662,973	-€ 15,994,459	-€ 21,325,945
2797	0.24	€ 376,354	€ 752,708	##########	##########	-€ 9,100,126	-€ 18,200,253	-€ 27,300,379	-€ 36,400,506
2798	0.21	€ 336,764	€ 673,529	##########	##########	-€ 8,142,863	-€ 16,285,726	-€ 24,428,590	-€ 32,571,453
2799	0.26	€ 416,663	€ 833,326	##########	##########	-€ 10,074,793	-€ 20,149,585	-€ 30,224,378	-€ 40,299,171
2800	0.17	€ 272,134	€ 544,267	€ 816,401	##########	-€ 6,580,110	-€ 13,160,219	-€ 19,740,329	-€ 26,320,439
2801	0.13	€ 208,916	€ 417,833	€ 626,749	€ 835,665	-€ 5,051,535	-€ 10,103,070	-€ 15,154,605	-€ 20,206,140
2802	0.17	€ 267,515	€ 535,031	€ 802,546	##########	-€ 6,468,442	-€ 12,936,883	-€ 19,405,325	-€ 25,873,766
2803	0.23	€ 366,650	€ 733,299	##########	##########	-€ 8,865,479	-€ 17,730,958	-€ 26,596,437	-€ 35,461,916
2804	0.25	€ 389,169	€ 778,338	##########	##########	-€ 9,409,994	-€ 18,819,989	-€ 28,229,983	-€ 37,639,977
2805	0.26	€ 409,761	€ 819,523	##########	##########	-€ 9,907,908	-€ 19,815,817	-€ 29,723,725	-€ 39,631,633
2806	0.17	€ 269,671	€ 539,342	€ 809,013	##########	-€ 6,520,564	-€ 13,041,128	-€ 19,561,693	-€ 26,082,257
2807	0.22	€ 350,378	€ 700,756	##########	##########	-€ 8,472,036	-€ 16,944,073	-€ 25,416,109	-€ 33,888,145
2808	0.21	€ 335,785	€ 671,570	##########	##########	-€ 8,119,177	-€ 16,238,354	-€ 24,357,531	-€ 32,476,708
2809	0.20	€ 309,017	€ 618,033	€ 927,050	##########	-€ 7,471,933	-€ 14,943,866	-€ 22,415,799	-€ 29,887,732
2810	0.20	€ 311,896	€ 623,793	€ 935,689	##########	-€ 7,541,559	-€ 15,083,119	-€ 22,624,678	-€ 30,166,238
2811	0.11	€ 175,486	€ 350,973	€ 526,459	€ 701,946	-€ 4,243,211	-€ 8,486,423	-€ 12,729,634	-€ 16,972,845
2812	0.13	€ 204,136	€ 408,272	€ 612,408	€ 816,544	-€ 4,935,947	-€ 9,871,894	-€ 14,807,841	-€ 19,743,788
2813	0.17	€ 263,879	€ 527,758	€ 791,637	##########	-€ 6,380,519	-€ 12,761,038	-€ 19,141,557	-€ 25,522,076
2814	0.22	€ 339,870	€ 679,740	##########	##########	-€ 8,217,957	-€ 16,435,914	-€ 24,653,871	-€ 32,871,828
2815	0.18	€ 288,422	€ 576,844	€ 865,266	##########	-€ 6,973,959	-€ 13,947,917	-€ 20,921,876	-€ 27,895,834
2816	0.14	€ 225,237	€ 450,473	€ 675,710	€ 900,946	-€ 5,446,153	-€ 10,892,306	-€ 16,338,460	-€ 21,784,613
2817	0.20	€ 320,118	€ 640,237	€ 960,355	##########	-€ 7,740,370	-€ 15,480,740	-€ 23,221,111	-€ 30,961,481
2818	0.20	€ 307,375	€ 614,750	€ 922,125	##########	-€ 7,432,240	-€ 14,864,480	-€ 22,296,720	-€ 29,728,961
2819	0.25	€ 394,815	€ 789,629	##########	##########	-€ 9,546,499	-€ 19,092,997	-€ 28,639,496	-€ 38,185,995
2820	0.27	€ 422,586	€ 845,173	##########	##########	-€ 10,218,013	-€ 20,436,026	-€ 30,654,040	-€ 40,872,053
2821	0.18	€ 278,897	€ 557,794	€ 836,690	##########	-€ 6,743,641	-€ 13,487,282	-€ 20,230,923	-€ 26,974,563
2822	0.24	€ 385,745	€ 771,490	##########	##########	-€ 9,327,196	-€ 18,654,391	-€ 27,981,587	-€ 37,308,783
2823	0.21	€ 330,430	€ 660,861	€ 991,291	##########	-€ 7,989,707	-€ 15,979,414	-€ 23,969,122	-€ 31,958,829
2824	0.22	€ 340,436	€ 680,873	##########	##########	-€ 8,231,649	-€ 16,463,297	-€ 24,694,946	-€ 32,926,595
2825	0.23	€ 362,255	€ 724,509	##########	##########	-€ 8,759,206	-€ 17,518,413	-€ 26,277,619	-€ 35,036,825
2826	0.19	€ 296,358	€ 592,715	€ 889,073	##########	-€ 7,165,839	-€ 14,331,678	-€ 21,497,517	-€ 28,663,357
2827	0.14	€ 223,904	€ 447,808	€ 671,713	€ 895,617	-€ 5,413,938	-€ 10,827,875	-€ 16,241,813	-€ 21,655,751
2828	0.19	€ 294,280	€ 588,560	€ 882,841	##########	-€ 7,115,609	-€ 14,231,218	-€ 21,346,826	-€ 28,462,435
2829	0.23	€ 363,117	€ 726,235	##########	##########	-€ 8,780,071	-€ 17,560,143	-€ 26,340,214	-€ 35,120,286
2830	0.19	€ 294,511	€ 589,022	€ 883,534	##########	-€ 7,121,193	-€ 14,242,386	-€ 21,363,579	-€ 28,484,771
2831	0.31	€ 485,618	€ 971,235	##########	##########	-€ 11,742,091	-€ 23,484,181	-€ 35,226,272	-€ 46,968,363
2832	0.20	€ 311,540	€ 623,081	€ 934,621	##########	-€ 7,532,954	-€ 15,065,908	-€ 22,598,862	-€ 30,131,816
2833	0.15	€ 230,858	€ 461,715	€ 692,573	€ 923,430	-€ 5,582,066	-€ 11,164,133	-€ 16,746,199	-€ 22,328,265
2834	0.17	€ 272,819	€ 545,638	€ 818,457	##########	-€ 6,596,686	-€ 13,193,371	-€ 19,790,057	-€ 26,386,743
2835	0.16	€ 254,131	€ 508,262	€ 762,394	##########	-€ 6,144,818	-€ 12,289,636	-€ 18,434,454	-€ 24,579,271
2836	0.24	€ 378,897	€ 757,794	##########	##########	-€ 9,161,615	-€ 18,323,229	-€ 27,484,844	-€ 36,646,459
2837	0.18	€ 287,188	€ 574,377	€ 861,565	##########	-€ 6,944,129	-€ 13,888,257	-€ 20,832,386	-€ 27,776,515
2838	0.18	€ 285,793	€ 571,587	€ 857,380	##########	-€ 6,910,400	-€ 13,820,799	-€ 20,731,199	-€ 27,641,599
2839	0.17	€ 261,802	€ 523,604	€ 785,407	##########	-€ 6,330,300	-€ 12,660,601	-€ 18,990,901	-€ 25,321,201
2840	0.26	€ 402,729	€ 805,457	##########	##########	-€ 9,737,857	-€ 19,475,713	-€ 29,213,570	-€ 38,951,427
2841	0.27	€ 425,965	€ 851,930	##########	##########	-€ 10,299,702	-€ 20,599,403	-€ 30,899,105	-€ 41,198,806
2842	0.20	€ 314,875	€ 629,749	€ 944,624	##########	-€ 7,613,574	-€ 15,227,148	-€ 22,840,722	-€ 30,454,296
2843	0.24	€ 378,347	€ 756,695	##########	##########	-€ 9,148,324	-€ 18,296,649	-€ 27,444,973	-€ 36,593,297
2844	0.24	€ 384,936	€ 769,871	##########	##########	-€ 9,307,631	-€ 18,615,262	-€ 27,922,892	-€ 37,230,523
2845	0.18	€ 278,442	€ 556,884	€ 835,326	##########	-€ 6,732,648	-€ 13,465,296	-€ 20,197,944	-€ 26,930,591
2846	0.19	€ 306,522	€ 613,045	€ 919,567	##########	-€ 7,411,623	-€ 14,823,245	-€ 22,234,868	-€ 29,646,490
2847	0.23	€ 364,499	€ 728,997	##########	##########	-€ 8,813,470	-€ 17,626,939	-€ 26,440,409	-€ 35,253,879
2848	0.16	€ 259,519	€ 519,038	€ 778,557	##########	-€ 6,275,094	-€ 12,550,189	-€ 18,825,283	-€ 25,100,377
2849	0.21	€ 334,188	€ 668,375	##########	##########	-€ 8,080,559	-€ 16,161,117	-€ 24,241,676	-€ 32,322,234
2850	0.18	€ 284,258	€ 568,516	€ 852,774	##########	-€ 6,873,276	-€ 13,746,552	-€ 20,619,828	-€ 27,493,104
2851	0.20	€ 307,541	€ 615,082	€ 922,624	##########	-€ 7,436,255	-€ 14,872,511	-€ 22,308,766	-€ 29,745,022
2852	0.15	€ 229,058	€ 458,116	€ 687,173	€ 916,231	-€ 5,538,550	-€ 11,077,100	-€ 16,615,650	-€ 22,154,200

2853	0.14	€ 215,190	€ 430,380	€ 645,570	€ 860,760	-€ 5,203,232	-€ 10,406,464	-€ 15,609,696	-€ 20,812,928
2854	0.17	€ 269,554	€ 539,108	€ 808,661	##########	-€ 6,517,730	-€ 13,035,460	-€ 19,553,190	-€ 26,070,920
2855	0.26	€ 406,221	€ 812,443	##########	##########	-€ 9,822,309	-€ 19,644,619	-€ 29,466,928	-€ 39,289,237
2856	0.14	€ 226,846	€ 453,693	€ 680,539	€ 907,385	-€ 5,485,075	-€ 10,970,151	-€ 16,455,226	-€ 21,940,302
2857	0.20	€ 319,750	€ 639,501	€ 959,251	##########	-€ 7,731,468	-€ 15,462,936	-€ 23,194,403	-€ 30,925,871
2858	0.22	€ 343,072	€ 686,144	##########	##########	-€ 8,295,380	-€ 16,590,761	-€ 24,886,141	-€ 33,181,521
2859	0.23	€ 359,759	€ 719,519	##########	##########	-€ 8,698,875	-€ 17,397,750	-€ 26,096,626	-€ 34,795,501
2860	0.16	€ 249,049	€ 498,098	€ 747,148	€ 996,197	-€ 6,021,935	-€ 12,043,870	-€ 18,065,805	-€ 24,087,740
2861	0.24	€ 381,176	€ 762,351	##########	##########	-€ 9,216,714	-€ 18,433,428	-€ 27,650,143	-€ 36,866,857
2862	0.17	€ 271,634	€ 543,268	€ 814,902	##########	-€ 6,568,027	-€ 13,136,054	-€ 19,704,081	-€ 26,272,108
2863	0.15	€ 230,304	€ 460,608	€ 690,913	€ 921,217	-€ 5,568,687	-€ 11,137,374	-€ 16,706,061	-€ 22,274,748
2864	0.20	€ 310,581	€ 621,161	€ 931,742	##########	-€ 7,509,749	-€ 15,019,498	-€ 22,529,246	-€ 30,038,995
2865	0.20	€ 318,312	€ 636,624	€ 954,935	##########	-€ 7,696,685	-€ 15,393,370	-€ 23,090,056	-€ 30,786,741
2866	0.20	€ 322,354	€ 644,707	€ 967,061	##########	-€ 7,794,416	-€ 15,588,833	-€ 23,383,249	-€ 31,177,666
2867	0.13	€ 210,034	€ 420,068	€ 630,103	€ 840,137	-€ 5,078,565	-€ 10,157,130	-€ 15,235,694	-€ 20,314,259
2868	0.20	€ 322,779	€ 645,558	€ 968,338	##########	-€ 7,804,705	-€ 15,609,411	-€ 23,414,116	-€ 31,218,821
2869	0.23	€ 361,285	€ 722,569	##########	##########	-€ 8,735,755	-€ 17,471,510	-€ 26,207,265	-€ 34,943,020
2870	0.17	€ 269,895	€ 539,791	€ 809,686	##########	-€ 6,525,989	-€ 13,051,978	-€ 19,577,967	-€ 26,103,956
2871	0.14	€ 220,873	€ 441,746	€ 662,619	€ 883,493	-€ 5,340,647	-€ 10,681,293	-€ 16,021,940	-€ 21,362,586
2872	0.14	€ 226,818	€ 453,636	€ 680,454	€ 907,272	-€ 5,484,393	-€ 10,968,786	-€ 16,453,179	-€ 21,937,572
2873	0.18	€ 281,489	€ 562,977	€ 844,466	##########	-€ 6,806,310	-€ 13,612,620	-€ 20,418,931	-€ 27,225,241
2874	0.20	€ 321,660	€ 643,320	€ 964,981	##########	-€ 7,777,648	-€ 15,555,296	-€ 23,332,944	-€ 31,110,592
2875	0.30	€ 468,025	€ 936,051	##########	##########	-€ 11,316,714	-€ 22,633,427	-€ 33,950,141	-€ 45,266,854
2876	0.18	€ 275,634	€ 551,269	€ 826,903	##########	-€ 6,664,759	-€ 13,329,519	-€ 19,994,278	-€ 26,659,037
2877	0.16	€ 256,068	€ 512,137	€ 768,205	##########	-€ 6,191,658	-€ 12,383,316	-€ 18,574,974	-€ 24,766,632
2878	0.20	€ 317,586	€ 635,173	€ 952,759	##########	-€ 7,679,144	-€ 15,358,288	-€ 23,037,432	-€ 30,716,576
2879	0.13	€ 203,876	€ 407,752	€ 611,628	€ 815,504	-€ 4,929,660	-€ 9,859,320	-€ 14,788,981	-€ 19,718,641
2880	0.21	€ 329,586	€ 659,173	€ 988,759	##########	-€ 7,969,298	-€ 15,938,595	-€ 23,907,893	-€ 31,877,191
2881	0.17	€ 263,342	€ 526,684	€ 790,026	##########	-€ 6,367,529	-€ 12,735,058	-€ 19,102,586	-€ 25,470,115
2882	0.19	€ 306,053	€ 612,106	€ 918,160	##########	-€ 7,400,275	-€ 14,800,550	-€ 22,200,825	-€ 29,601,099
2883	0.17	€ 270,024	€ 540,049	€ 810,073	##########	-€ 6,529,111	-€ 13,058,222	-€ 19,587,333	-€ 26,116,444
2884	0.17	€ 267,393	€ 534,786	€ 802,178	##########	-€ 6,465,479	-€ 12,930,957	-€ 19,396,436	-€ 25,861,914
2885	0.19	€ 299,265	€ 598,530	€ 897,795	##########	-€ 7,236,139	-€ 14,472,279	-€ 21,708,418	-€ 28,944,558
2886	0.14	€ 219,500	€ 439,001	€ 658,501	€ 878,001	-€ 5,307,452	-€ 10,614,904	-€ 15,922,356	-€ 21,229,808
2887	0.20	€ 321,034	€ 642,068	€ 963,102	##########	-€ 7,762,503	-€ 15,525,006	-€ 23,287,508	-€ 31,050,011
2888	0.24	€ 375,987	€ 751,973	##########	##########	-€ 9,091,245	-€ 18,182,491	-€ 27,273,736	-€ 36,364,982
2889	0.21	€ 323,834	€ 647,669	€ 971,503	##########	-€ 7,830,218	-€ 15,660,436	-€ 23,490,653	-€ 31,320,871
2890	0.20	€ 320,314	€ 640,628	€ 960,942	##########	-€ 7,745,101	-€ 15,490,202	-€ 23,235,303	-€ 30,980,404
2891	0.16	€ 250,264	€ 500,527	€ 750,791	##########	-€ 6,051,300	-€ 12,102,599	-€ 18,153,899	-€ 24,205,198
2892	0.17	€ 270,198	€ 540,396	€ 810,594	##########	-€ 6,533,307	-€ 13,066,614	-€ 19,599,922	-€ 26,133,229
2893	0.20	€ 313,261	€ 626,522	€ 939,783	##########	-€ 7,574,557	-€ 15,149,113	-€ 22,723,670	-€ 30,298,226
2894	0.20	€ 319,797	€ 639,594	€ 959,391	##########	-€ 7,732,601	-€ 15,465,201	-€ 23,197,802	-€ 30,930,402
2895	0.18	€ 279,446	€ 558,893	€ 838,339	##########	-€ 6,756,933	-€ 13,513,865	-€ 20,270,798	-€ 27,027,730
2896	0.29	€ 453,854	€ 907,707	##########	##########	-€ 10,974,047	-€ 21,948,094	-€ 32,922,141	-€ 43,896,188
2897	0.22	€ 343,572	€ 687,145	##########	##########	-€ 8,307,478	-€ 16,614,957	-€ 24,922,435	-€ 33,229,913
2898	0.21	€ 338,292	€ 676,584	##########	##########	-€ 8,179,799	-€ 16,359,599	-€ 24,539,398	-€ 32,719,198
2899	0.13	€ 198,724	€ 397,449	€ 596,173	€ 794,898	-€ 4,805,097	-€ 9,610,195	-€ 14,415,292	-€ 19,220,389
2900	0.22	€ 350,508	€ 701,016	##########	##########	-€ 8,475,179	-€ 16,950,358	-€ 25,425,537	-€ 33,900,716
2901	0.20	€ 320,354	€ 640,708	€ 961,062	##########	-€ 7,746,061	-€ 15,492,122	-€ 23,238,183	-€ 30,984,245
2902	0.12	€ 189,195	€ 378,390	€ 567,585	€ 756,780	-€ 4,574,679	-€ 9,149,358	-€ 13,724,037	-€ 18,298,716
2903	0.20	€ 316,290	€ 632,579	€ 948,869	##########	-€ 7,647,787	-€ 15,295,575	-€ 22,943,362	-€ 30,591,149
2904	0.16	€ 248,280	€ 496,559	€ 744,839	€ 993,119	-€ 6,003,330	-€ 12,006,659	-€ 18,009,989	-€ 24,013,319
2905	0.19	€ 300,865	€ 601,731	€ 902,596	##########	-€ 7,274,836	-€ 14,549,671	-€ 21,824,507	-€ 29,099,343
2906	0.17	€ 269,680	€ 539,360	€ 809,040	##########	-€ 6,520,785	-€ 13,041,569	-€ 19,562,354	-€ 26,083,139
2907	0.10	€ 154,816	€ 309,632	€ 464,448	€ 619,264	-€ 3,743,404	-€ 7,486,807	-€ 11,230,211	-€ 14,973,614
2908	0.25	€ 389,376	€ 778,752	##########	##########	-€ 9,414,991	-€ 18,829,982	-€ 28,244,972	-€ 37,659,963
2909	0.26	€ 416,469	€ 832,938	##########	##########	-€ 10,070,093	-€ 20,140,185	-€ 30,210,278	-€ 40,280,371
2910	0.11	€ 179,918	€ 359,835	€ 539,753	€ 719,670	-€ 4,350,354	-€ 8,700,708	-€ 13,051,061	-€ 17,401,415
2911	0.20	€ 322,547	€ 645,094	€ 967,641	##########	-€ 7,799,089	-€ 15,598,177	-€ 23,397,266	-€ 31,196,354
2912	0.15	€ 241,290	€ 482,580	€ 723,870	€ 965,160	-€ 5,834,323	-€ 11,668,645	-€ 17,502,968	-€ 23,337,291
2913	0.21	€ 324,967	€ 649,933	€ 974,900	##########	-€ 7,857,598	-€ 15,715,197	-€ 23,572,795	-€ 31,430,394
2914	0.18	€ 281,248	€ 562,496	€ 843,743	##########	-€ 6,800,489	-€ 13,600,978	-€ 20,401,467	-€ 27,201,956
2915	0.16	€ 245,348	€ 490,695	€ 736,043	€ 981,391	-€ 5,932,434	-€ 11,864,869	-€ 17,797,303	-€ 23,729,737
2916	0.15	€ 238,546	€ 477,091	€ 715,637	€ 954,183	-€ 5,767,963	-€ 11,535,926	-€ 17,303,889	-€ 23,071,852
2917	0.19	€ 299,946	€ 599,891	€ 899,837	##########	-€ 7,252,597	-€ 14,505,194	-€ 21,757,791	-€ 29,010,388
2918	0.19	€ 294,629	€ 589,257	€ 883,886	##########	-€ 7,124,031	-€ 14,248,062	-€ 21,372,093	-€ 28,496,124
2919	0.16	€ 252,504	€ 505,009	€ 757,513	##########	-€ 6,105,482	-€ 12,210,963	-€ 18,316,445	-€ 24,421,926
2920	0.19	€ 293,277	€ 586,554	€ 879,831	##########	-€ 7,091,351	-€ 14,182,701	-€ 21,274,052	-€ 28,365,403
2921	0.20	€ 311,695	€ 623,390	€ 935,085	##########	-€ 7,536,696	-€ 15,073,391	-€ 22,610,087	-€ 30,146,783
2922	0.25	€ 395,441	€ 790,882	##########	##########	-€ 9,561,651	-€ 19,123,303	-€ 28,684,954	-€ 38,246,605
2923	0.16	€ 247,377	€ 494,755	€ 742,132	€ 989,509	-€ 5,981,510	-€ 11,963,021	-€ 17,944,531	-€ 23,926,041
2924	0.22	€ 351,490	€ 702,980	##########	##########	-€ 8,498,920	-€ 16,997,840	-€ 25,496,761	-€ 33,995,681
2925	0.19	€ 301,132	€ 602,265	€ 903,397	##########	-€ 7,281,290	-€ 14,562,580	-€ 21,843,870	-€ 29,125,160
2926	0.23	€ 359,937	€ 719,874	##########	##########	-€ 8,703,172	-€ 17,406,343	-€ 26,109,515	-€ 34,812,686
2927	0.17	€ 262,174	€ 524,347	€ 786,521	##########	-€ 6,339,280	-€ 12,678,561	-€ 19,017,841	-€ 25,357,121
2928	0.19	€ 300,361	€ 600,721	€ 901,082	##########	-€ 7,262,628	-€ 14,525,257	-€ 21,787,885	-€ 29,050,514
2929	0.23	€ 364,841	€ 729,682	##########	##########	-€ 8,821,746	-€ 17,643,493	-€ 26,465,239	-€ 35,286,986
2930	0.19	€ 295,017	€ 590,035	€ 885,052	##########	-€ 7,133,432	-€ 14,266,865	-€ 21,400,297	-€ 28,533,730
2931	0.20	€ 310,117	€ 620,234	€ 930,351	##########	-€ 7,498,540	-€ 14,997,079	-€ 22,495,619	-€ 29,994,158
2932	0.24	€ 378,979	€ 757,959	##########	##########	-€ 9,163,607	-€ 18,327,213	-€ 27,490,820	-€ 36,654,426
2933	0.20	€ 319,892	€ 639,784	€ 959,677	##########	-€ 7,734,899	-€ 15,469,799	-€ 23,204,698	-€ 30,939,597
2934	0.21	€ 329,817	€ 659,634	€ 989,450	##########	-€ 7,974,873	-€ 15,949,745	-€ 23,924,618	-€ 31,899,490
2935	0.13	€ 201,589	€ 403,177	€ 604,766	€ 806,355	-€ 4,874,355	-€ 9,748,710	-€ 14,623,066	-€ 19,497,421
2936	0.20	€ 316,636	€ 633,271	€ 949,907	##########	-€ 7,656,158	-€ 15,312,315	-€ 22,968,473	-€ 30,624,631
2937	0.22	€ 353,869	€ 707,737	##########	##########	-€ 8,556,440	-€ 17,112,880	-€ 25,669,320	-€ 34,225,760
2938	0.16	€ 253,581	€ 507,163	€ 760,744	##########	-€ 6,131,524	-€ 12,263,047	-€ 18,394,571	-€ 24,526,094
2939	0.26	€ 405,342	€ 810,684	##########	##########	-€ 9,801,047	-€ 19,602,094	-€ 29,403,141	-€ 39,204,189
2940	0.17	€ 274,493	€ 548,986	€ 823,480	##########	-€ 6,637,165	-€ 13,274,330	-€ 19,911,496	-€ 26,548,661
2941	0.21	€ 330,515	€ 661,030	€ 991,545	##########	-€ 7,991,758	-€ 15,983,516	-€ 23,975,273	-€ 31,967,031
2942	0.19	€ 302,060	€ 604,120	€ 906,179	##########	-€ 7,303,716	-€ 14,607,433	-€ 21,911,149	-€ 29,214,866
2943	0.17	€ 274,172	€ 548,344	€ 822,516	##########	-€ 6,629,394	-€ 13,258,789	-€ 19,888,183	-€ 26,517,577
2944	0.16	€ 254,214	€ 508,429	€ 762,643	##########	-€ 6,146,826	-€ 12,293,653	-€ 18,440,479	-€ 24,587,306
2945	0.21	€ 335,697	€ 671,394	##########	##########	-€ 8,117,049	-€ 16,234,098	-€ 24,351,147	-€ 32,468,196
2946	0.18	€ 284,961	€ 569,923	€ 854,884	##########	-€ 6,890,280	-€ 13,780,560	-€ 20,670,840	-€ 27,561,121
2947	0.21	€ 334,600	€ 669,200	##########	##########	-€ 8,090,523	-€ 16,181,046	-€ 24,271,568	-€ 32,362,091
2948	0.21	€ 332,193	€ 664,385	€ 996,578	##########	-€ 8,032,317	-€ 16,064,635	-€ 24,096,952	-€ 32,129,269
2949	0.18	€ 280,344	€ 560,688	€ 841,032	##########	-€ 6,778,631	-€ 13,557,261	-€ 20,335,892	-€ 27,114,523
2950	0.24	€ 371,472	€ 742,944	##########	##########	-€ 8,982,078	-€ 17,964,156	-€ 26,946,234	-€ 35,928,312
2951	0.20	€ 317,956	€ 635,912	€ 953,868	##########	-€ 7,688,081	-€ 15,376,162	-€ 23,064,243	-€ 30,752,324
2952	0.23	€ 366,564	€ 733,128	##########	##########	-€ 8,863,403	-€ 17,726,806	-€ 26,590,209	-€ 35,453,613
2953	0.16	€ 259,007	€ 518,014	€ 777,021	##########	-€ 6,262,709	-€ 12,525,418	-€ 18,788,128	-€ 25,050,837
2954	0.13	€ 210,240	€ 420,481	€ 630,721	€ 840,961	-€ 5,083,548	-€ 10,167,095	-€ 15,250,643	-€ 20,334,190

2955	0.15	€ 235,811	€ 471,622	€ 707,433	€ 943,244	-€ 5,701,840	-€ 11,403,680	-€ 17,105,519	-€ 22,807,359
2956	0.22	€ 339,158	€ 678,315	##########	##########	-€ 8,200,731	-€ 16,401,462	-€ 24,602,192	-€ 32,802,923
2957	0.15	€ 231,636	€ 463,273	€ 694,909	€ 926,545	-€ 5,600,899	-€ 11,201,797	-€ 16,802,696	-€ 22,403,595
2958	0.22	€ 339,379	€ 678,757	##########	##########	-€ 8,206,072	-€ 16,412,144	-€ 24,618,215	-€ 32,824,287
2959	0.19	€ 303,754	€ 607,507	€ 911,261	##########	-€ 7,344,675	-€ 14,689,349	-€ 22,034,024	-€ 29,378,698
2960	0.18	€ 289,431	€ 578,861	€ 868,292	##########	-€ 6,998,349	-€ 13,996,698	-€ 20,995,047	-€ 27,993,397
2961	0.19	€ 301,497	€ 602,993	€ 904,490	##########	-€ 7,290,100	-€ 14,580,200	-€ 21,870,300	-€ 29,160,399
2962	0.17	€ 273,097	€ 546,194	€ 819,291	##########	-€ 6,603,406	-€ 13,206,811	-€ 19,810,217	-€ 26,413,623
2963	0.17	€ 266,003	€ 532,007	€ 798,010	##########	-€ 6,431,880	-€ 12,863,760	-€ 19,295,639	-€ 25,727,519
2964	0.13	€ 210,742	€ 421,484	€ 632,226	€ 842,968	-€ 5,095,678	-€ 10,191,355	-€ 15,287,033	-€ 20,382,711
2965	0.16	€ 254,898	€ 509,796	€ 764,695	##########	-€ 6,163,364	-€ 12,326,727	-€ 18,490,091	-€ 24,653,454
2966	0.16	€ 249,977	€ 499,954	€ 749,931	€ 999,908	-€ 6,044,370	-€ 12,088,740	-€ 18,133,109	-€ 24,177,479
2967	0.17	€ 266,365	€ 532,730	€ 799,095	##########	-€ 6,440,630	-€ 12,881,260	-€ 19,321,890	-€ 25,762,520
2968	0.24	€ 376,979	€ 753,958	##########	##########	-€ 9,115,243	-€ 18,230,486	-€ 27,345,729	-€ 36,460,973
2969	0.20	€ 315,694	€ 631,388	€ 947,083	##########	-€ 7,633,392	-€ 15,266,784	-€ 22,900,176	-€ 30,533,567
2970	0.28	€ 438,895	€ 877,790	##########	##########	-€ 10,612,352	-€ 21,224,704	-€ 31,837,056	-€ 42,449,409
2971	0.20	€ 321,722	€ 643,444	€ 965,166	##########	-€ 7,779,143	-€ 15,558,286	-€ 23,337,429	-€ 31,116,572
2972	0.17	€ 272,202	€ 544,404	€ 816,607	##########	-€ 6,581,768	-€ 13,163,536	-€ 19,745,304	-€ 26,327,072
2973	0.16	€ 257,050	€ 514,100	€ 771,150	##########	-€ 6,215,394	-€ 12,430,787	-€ 18,646,181	-€ 24,861,575
2974	0.21	€ 332,164	€ 664,329	€ 996,493	##########	-€ 8,031,637	-€ 16,063,274	-€ 24,094,911	-€ 32,126,548
2975	0.17	€ 267,110	€ 534,221	€ 801,331	##########	-€ 6,458,647	-€ 12,917,294	-€ 19,375,940	-€ 25,834,587
2976	0.16	€ 247,496	€ 494,992	€ 742,488	€ 989,984	-€ 5,984,378	-€ 11,968,756	-€ 17,953,133	-€ 23,937,511
2977	0.21	€ 333,947	€ 667,894	##########	##########	-€ 8,074,738	-€ 16,149,476	-€ 24,224,214	-€ 32,298,952
2978	0.17	€ 263,225	€ 526,451	€ 789,676	##########	-€ 6,364,713	-€ 12,729,426	-€ 19,094,139	-€ 25,458,852
2979	0.18	€ 279,617	€ 559,234	€ 838,852	##########	-€ 6,761,062	-€ 13,522,123	-€ 20,283,185	-€ 27,044,247
2980	0.21	€ 334,755	€ 669,511	##########	##########	-€ 8,094,283	-€ 16,188,566	-€ 24,282,849	-€ 32,377,132
2981	0.21	€ 335,635	€ 671,270	##########	##########	-€ 8,115,549	-€ 16,231,099	-€ 24,346,648	-€ 32,462,198
2982	0.20	€ 308,071	€ 616,141	€ 924,212	##########	-€ 7,449,058	-€ 14,898,116	-€ 22,347,174	-€ 29,796,232
2983	0.18	€ 282,398	€ 564,796	€ 847,193	##########	-€ 6,828,294	-€ 13,656,589	-€ 20,484,883	-€ 27,313,177
2984	0.14	€ 221,247	€ 442,494	€ 663,741	€ 884,988	-€ 5,349,687	-€ 10,699,373	-€ 16,049,060	-€ 21,398,747
2985	0.21	€ 334,361	€ 668,722	##########	##########	-€ 8,084,745	-€ 16,169,490	-€ 24,254,235	-€ 32,338,980
2986	0.19	€ 306,221	€ 612,443	€ 918,664	##########	-€ 7,404,343	-€ 14,808,687	-€ 22,213,030	-€ 29,617,374
2987	0.19	€ 304,010	€ 608,020	€ 912,029	##########	-€ 7,350,866	-€ 14,701,732	-€ 22,052,598	-€ 29,403,464
2988	0.12	€ 194,229	€ 388,458	€ 582,687	€ 776,917	-€ 4,696,404	-€ 9,392,807	-€ 14,089,211	-€ 18,785,614
2989	0.16	€ 252,702	€ 505,403	€ 758,105	##########	-€ 6,110,249	-€ 12,220,499	-€ 18,330,748	-€ 24,440,997
2990	0.17	€ 269,003	€ 538,006	€ 807,009	##########	-€ 6,504,410	-€ 13,008,820	-€ 19,513,230	-€ 26,017,640
2991	0.20	€ 315,662	€ 631,325	€ 946,987	##########	-€ 7,632,621	-€ 15,265,242	-€ 22,897,863	-€ 30,530,484
2992	0.17	€ 263,401	€ 526,802	€ 790,204	##########	-€ 6,368,964	-€ 12,737,928	-€ 19,106,892	-€ 25,475,856
2993	0.20	€ 321,704	€ 643,409	€ 965,113	##########	-€ 7,778,717	-€ 15,557,434	-€ 23,336,151	-€ 31,114,869
2994	0.26	€ 414,803	€ 829,607	##########	##########	-€ 10,029,821	-€ 20,059,642	-€ 30,089,463	-€ 40,119,283
2995	0.15	€ 241,235	€ 482,471	€ 723,706	€ 964,941	-€ 5,832,998	-€ 11,665,997	-€ 17,498,995	-€ 23,331,993
2996	0.25	€ 392,895	€ 785,790	##########	##########	-€ 9,500,086	-€ 19,000,172	-€ 28,500,258	-€ 38,000,344
2997	0.21	€ 327,433	€ 654,867	€ 982,300	##########	-€ 7,917,241	-€ 15,834,482	-€ 23,751,723	-€ 31,668,964
2998	0.18	€ 276,735	€ 553,470	€ 830,205	##########	-€ 6,691,366	-€ 13,382,733	-€ 20,074,099	-€ 26,765,466
2999	0.14	€ 222,107	€ 444,215	€ 666,322	€ 888,430	-€ 5,370,493	-€ 10,740,987	-€ 16,111,480	-€ 21,481,973
3000	0.13	€ 210,890	€ 421,781	€ 632,671	€ 843,561	-€ 5,099,264	-€ 10,198,528	-€ 15,297,792	-€ 20,397,055
3001	0.13	€ 210,154	€ 420,308	€ 630,462	€ 840,617	-€ 5,081,465	-€ 10,162,931	-€ 15,244,396	-€ 20,325,861
3002	0.20	€ 309,061	€ 618,122	€ 927,183	##########	-€ 7,473,005	-€ 14,946,009	-€ 22,419,014	-€ 29,892,019
3003	0.14	€ 220,084	€ 440,168	€ 660,253	€ 880,337	-€ 5,321,571	-€ 10,643,141	-€ 15,964,712	-€ 21,286,282
3004	0.23	€ 362,454	€ 724,909	##########	##########	-€ 8,764,040	-€ 17,528,081	-€ 26,292,121	-€ 35,056,162
3005	0.19	€ 303,962	€ 607,923	€ 911,885	##########	-€ 7,349,701	-€ 14,699,403	-€ 22,049,104	-€ 29,398,805
3006	0.13	€ 207,433	€ 414,867	€ 622,300	€ 829,733	-€ 5,015,675	-€ 10,031,350	-€ 15,047,025	-€ 20,062,700
3007	0.18	€ 284,415	€ 568,830	€ 853,246	##########	-€ 6,877,075	-€ 13,754,150	-€ 20,631,225	-€ 27,508,300
3008	0.15	€ 241,905	€ 483,811	€ 725,716	€ 967,621	-€ 5,849,198	-€ 11,698,396	-€ 17,547,594	-€ 23,396,792
3009	0.17	€ 263,748	€ 527,497	€ 791,245	##########	-€ 6,377,357	-€ 12,754,714	-€ 19,132,072	-€ 25,509,429
3010	0.17	€ 262,455	€ 524,910	€ 787,365	##########	-€ 6,346,087	-€ 12,692,175	-€ 19,038,262	-€ 25,384,349
3011	0.23	€ 362,710	€ 725,420	##########	##########	-€ 8,770,218	-€ 17,540,435	-€ 26,310,653	-€ 35,080,871
3012	0.13	€ 198,660	€ 397,319	€ 595,979	€ 794,639	-€ 4,803,532	-€ 9,607,065	-€ 14,410,597	-€ 19,214,130
3013	0.18	€ 285,962	€ 571,925	€ 857,887	##########	-€ 6,914,485	-€ 13,828,969	-€ 20,743,454	-€ 27,657,938
3014	0.16	€ 251,589	€ 503,178	€ 754,767	##########	-€ 6,083,347	-€ 12,166,694	-€ 18,250,040	-€ 24,333,387
3015	0.20	€ 313,218	€ 626,435	€ 939,653	##########	-€ 7,573,511	-€ 15,147,021	-€ 22,720,532	-€ 30,294,043
3016	0.24	€ 375,314	€ 750,628	##########	##########	-€ 9,074,983	-€ 18,149,966	-€ 27,224,950	-€ 36,299,933
3017	0.25	€ 400,782	€ 801,563	##########	##########	-€ 9,690,782	-€ 19,381,565	-€ 29,072,347	-€ 38,763,129
3018	0.19	€ 293,571	€ 587,142	€ 880,713	##########	-€ 7,098,460	-€ 14,196,920	-€ 21,295,381	-€ 28,393,841
3019	0.27	€ 426,639	€ 853,279	##########	##########	-€ 10,316,015	-€ 20,632,030	-€ 30,948,045	-€ 41,264,060
3020	0.15	€ 235,980	€ 471,959	€ 707,939	€ 943,918	-€ 5,705,915	-€ 11,411,830	-€ 17,117,746	-€ 22,823,661
3021	0.16	€ 248,759	€ 497,518	€ 746,277	€ 995,036	-€ 6,014,918	-€ 12,029,837	-€ 18,044,755	-€ 24,059,674
3022	0.22	€ 338,559	€ 677,117	##########	##########	-€ 8,186,245	-€ 16,372,490	-€ 24,558,735	-€ 32,744,980
3023	0.22	€ 343,060	€ 686,120	##########	##########	-€ 8,295,094	-€ 16,590,188	-€ 24,885,282	-€ 33,180,376
3024	0.21	€ 323,828	€ 647,656	€ 971,485	##########	-€ 7,830,069	-€ 15,660,138	-€ 23,490,207	-€ 31,320,277
3025	0.19	€ 301,419	€ 602,838	€ 904,257	##########	-€ 7,288,225	-€ 14,576,449	-€ 21,864,674	-€ 29,152,899
3026	0.15	€ 229,731	€ 459,462	€ 689,193	€ 918,923	-€ 5,554,824	-€ 11,109,647	-€ 16,664,471	-€ 22,219,294
3027	0.17	€ 271,315	€ 542,630	€ 813,945	##########	-€ 6,560,319	-€ 13,120,638	-€ 19,680,958	-€ 26,241,277
3028	0.17	€ 273,051	€ 546,102	€ 819,153	##########	-€ 6,602,294	-€ 13,204,588	-€ 19,806,883	-€ 26,409,177
3029	0.21	€ 331,584	€ 663,167	€ 994,751	##########	-€ 8,017,593	-€ 16,035,186	-€ 24,052,778	-€ 32,070,371
3030	0.21	€ 334,238	€ 668,476	##########	##########	-€ 8,081,776	-€ 16,163,552	-€ 24,245,329	-€ 32,327,105
3031	0.18	€ 284,539	€ 569,077	€ 853,616	##########	-€ 6,880,059	-€ 13,760,118	-€ 20,640,177	-€ 27,520,235
3032	0.19	€ 291,529	€ 583,058	€ 874,586	##########	-€ 7,049,080	-€ 14,098,160	-€ 21,147,240	-€ 28,196,320
3033	0.26	€ 414,141	€ 828,282	##########	##########	-€ 10,013,807	-€ 20,027,614	-€ 30,041,421	-€ 40,055,229
3034	0.14	€ 215,978	€ 431,956	€ 647,935	€ 863,913	-€ 5,222,289	-€ 10,444,578	-€ 15,666,867	-€ 20,889,156
3035	0.21	€ 332,174	€ 664,347	€ 996,521	##########	-€ 8,031,862	-€ 16,063,724	-€ 24,095,587	-€ 32,127,449
3036	0.21	€ 337,589	€ 675,178	##########	##########	-€ 8,162,798	-€ 16,325,595	-€ 24,488,393	-€ 32,651,191
3037	0.14	€ 217,784	€ 435,568	€ 653,352	€ 871,136	-€ 5,265,952	-€ 10,531,905	-€ 15,797,857	-€ 21,063,809
3038	0.22	€ 347,166	€ 694,332	##########	##########	-€ 8,394,377	-€ 16,788,753	-€ 25,183,130	-€ 33,577,507
3039	0.26	€ 408,432	€ 816,865	##########	##########	-€ 9,875,775	-€ 19,751,550	-€ 29,627,324	-€ 39,503,099
3040	0.17	€ 265,262	€ 530,524	€ 795,786	##########	-€ 6,413,954	-€ 12,827,908	-€ 19,241,863	-€ 25,655,817
3041	0.15	€ 232,360	€ 464,720	€ 697,081	€ 929,441	-€ 5,618,400	-€ 11,236,800	-€ 16,855,200	-€ 22,473,600
3042	0.18	€ 279,993	€ 559,985	€ 839,978	##########	-€ 6,770,139	-€ 13,540,279	-€ 20,310,418	-€ 27,080,558
3043	0.16	€ 259,594	€ 519,188	€ 778,782	##########	-€ 6,276,909	-€ 12,553,818	-€ 18,830,727	-€ 25,107,636
3044	0.21	€ 322,977	€ 645,953	€ 968,930	##########	-€ 7,809,479	-€ 15,618,958	-€ 23,428,438	-€ 31,237,917
3045	0.20	€ 313,186	€ 626,372	€ 939,557	##########	-€ 7,572,739	-€ 15,145,478	-€ 22,718,217	-€ 30,290,956
3046	0.27	€ 423,054	€ 846,107	##########	##########	-€ 10,229,313	-€ 20,458,625	-€ 30,687,938	-€ 40,917,250
3047	0.17	€ 275,362	€ 550,724	€ 826,086	##########	-€ 6,658,173	-€ 13,316,345	-€ 19,974,518	-€ 26,632,691
3048	0.12	€ 196,610	€ 393,220	€ 589,830	€ 786,440	-€ 4,753,972	-€ 9,507,944	-€ 14,261,916	-€ 19,015,888
3049	0.12	€ 191,310	€ 382,621	€ 573,931	€ 765,241	-€ 4,625,827	-€ 9,251,653	-€ 13,877,480	-€ 18,503,307
3050	0.19	€ 299,870	€ 599,740	€ 899,610	##########	-€ 7,250,765	-€ 14,501,530	-€ 21,752,295	-€ 29,003,060
3051	0.22	€ 345,585	€ 691,169	##########	##########	-€ 8,356,136	-€ 16,712,271	-€ 25,068,407	-€ 33,424,542
3052	0.21	€ 328,397	€ 656,795	€ 985,192	##########	-€ 7,940,551	-€ 15,881,102	-€ 23,821,653	-€ 31,762,204
3053	0.25	€ 395,025	€ 790,050	##########	##########	-€ 9,551,591	-€ 19,103,182	-€ 28,654,773	-€ 38,206,364
3054	0.24	€ 371,418	€ 742,836	##########	##########	-€ 8,980,778	-€ 17,961,557	-€ 26,942,335	-€ 35,923,113
3055	0.20	€ 314,121	€ 628,243	€ 942,364	##########	-€ 7,595,361	-€ 15,190,723	-€ 22,786,084	-€ 30,381,445
3056	0.15	€ 231,050	€ 462,100	€ 693,149	€ 924,199	-€ 5,586,715	-€ 11,173,430	-€ 16,760,145	-€ 22,346,860

3057	0.15	€ 233,001	€ 466,003	€ 699,004	€ 932,005	-€ 5,633,901	-€ 11,267,803	-€ 16,901,704	-€ 22,535,605
3058	0.18	€ 288,236	€ 576,471	€ 864,707	##########	-€ 6,969,453	-€ 13,938,906	-€ 20,908,359	-€ 27,877,811
3059	0.17	€ 267,150	€ 534,300	€ 801,450	##########	-€ 6,459,606	-€ 12,919,211	-€ 19,378,817	-€ 25,838,423
3060	0.20	€ 320,479	€ 640,958	€ 961,437	##########	-€ 7,749,084	-€ 15,498,168	-€ 23,247,252	-€ 30,996,336
3061	0.26	€ 417,078	€ 834,155	##########	##########	-€ 10,084,810	-€ 20,169,621	-€ 30,254,431	-€ 40,339,242
3062	0.12	€ 186,409	€ 372,819	€ 559,228	€ 745,638	-€ 4,507,325	-€ 9,014,650	-€ 13,521,975	-€ 18,029,300
3063	0.18	€ 275,942	€ 551,885	€ 827,827	##########	-€ 6,672,207	-€ 13,344,415	-€ 20,016,622	-€ 26,688,829
3064	0.23	€ 363,060	€ 726,121	##########	##########	-€ 8,778,690	-€ 17,557,380	-€ 26,336,070	-€ 35,114,761
3065	0.16	€ 256,002	€ 512,005	€ 768,007	##########	-€ 6,190,059	-€ 12,380,118	-€ 18,570,177	-€ 24,760,236
3066	0.20	€ 310,248	€ 620,496	€ 930,744	##########	-€ 7,501,707	-€ 15,003,415	-€ 22,505,122	-€ 30,006,830
3067	0.18	€ 278,138	€ 556,277	€ 834,415	##########	-€ 6,725,303	-€ 13,450,606	-€ 20,175,909	-€ 26,901,212
3068	0.14	€ 220,559	€ 441,117	€ 661,676	€ 882,235	-€ 5,333,044	-€ 10,666,088	-€ 15,999,133	-€ 21,332,177
3069	0.23	€ 359,187	€ 718,374	##########	##########	-€ 8,685,040	-€ 17,370,080	-€ 26,055,119	-€ 34,740,159
3070	0.20	€ 312,043	€ 624,085	€ 936,128	##########	-€ 7,545,100	-€ 15,090,200	-€ 22,635,300	-€ 30,180,401
3071	0.18	€ 291,227	€ 582,454	€ 873,681	##########	-€ 7,041,783	-€ 14,083,566	-€ 21,125,349	-€ 28,167,132
3072	0.16	€ 257,999	€ 515,997	€ 773,996	##########	-€ 6,238,330	-€ 12,476,660	-€ 18,714,990	-€ 24,953,320
3073	0.18	€ 277,002	€ 554,004	€ 831,005	##########	-€ 6,697,820	-€ 13,395,640	-€ 20,093,460	-€ 26,791,280
3074	0.26	€ 412,854	€ 825,708	##########	##########	-€ 9,982,691	-€ 19,965,381	-€ 29,948,072	-€ 39,930,763
3075	0.20	€ 322,091	€ 644,182	€ 966,273	##########	-€ 7,788,067	-€ 15,576,134	-€ 23,364,202	-€ 31,152,269
3076	0.14	€ 219,451	€ 438,902	€ 658,353	€ 877,804	-€ 5,306,262	-€ 10,612,523	-€ 15,918,785	-€ 21,225,046
3077	0.18	€ 289,739	€ 579,479	€ 869,218	##########	-€ 7,005,811	-€ 14,011,622	-€ 21,017,433	-€ 28,023,244
3078	0.13	€ 206,572	€ 413,145	€ 619,717	€ 826,289	-€ 4,994,859	-€ 9,989,717	-€ 14,984,576	-€ 19,979,435
3079	0.15	€ 229,276	€ 458,552	€ 687,828	€ 917,104	-€ 5,543,827	-€ 11,087,655	-€ 16,631,482	-€ 22,175,309
3080	0.17	€ 265,445	€ 530,890	€ 796,335	##########	-€ 6,418,379	-€ 12,836,759	-€ 19,255,138	-€ 25,673,518
3081	0.16	€ 249,829	€ 499,658	€ 749,487	€ 999,316	-€ 6,040,789	-€ 12,081,577	-€ 18,122,366	-€ 24,163,155
3082	0.19	€ 303,861	€ 607,723	€ 911,584	##########	-€ 7,347,275	-€ 14,694,551	-€ 22,041,826	-€ 29,389,102
3083	0.17	€ 271,556	€ 543,112	€ 814,669	##########	-€ 6,566,148	-€ 13,132,296	-€ 19,698,444	-€ 26,264,592
3084	0.19	€ 297,741	€ 595,482	€ 893,224	##########	-€ 7,199,293	-€ 14,398,587	-€ 21,597,880	-€ 28,797,173
3085	0.32	€ 496,119	€ 992,238	##########	##########	-€ 11,996,005	-€ 23,992,011	-€ 35,988,016	-€ 47,984,022
3086	0.25	€ 393,825	€ 787,650	##########	##########	-€ 9,522,576	-€ 19,045,153	-€ 28,567,729	-€ 38,090,306
3087	0.23	€ 356,033	€ 712,065	##########	##########	-€ 8,608,763	-€ 17,217,525	-€ 25,826,288	-€ 34,435,050
3088	0.24	€ 370,929	€ 741,859	##########	##########	-€ 8,968,964	-€ 17,937,927	-€ 26,906,891	-€ 35,875,854
3089	0.16	€ 256,158	€ 512,316	€ 768,473	##########	-€ 6,193,819	-€ 12,387,637	-€ 18,581,456	-€ 24,775,274
3090	0.19	€ 295,741	€ 591,482	€ 887,224	##########	-€ 7,150,934	-€ 14,301,868	-€ 21,452,802	-€ 28,603,736
3091	0.16	€ 248,806	€ 497,612	€ 746,417	€ 995,223	-€ 6,016,050	-€ 12,032,100	-€ 18,048,150	-€ 24,064,200
3092	0.24	€ 374,842	€ 749,685	##########	##########	-€ 9,063,580	-€ 18,127,159	-€ 27,190,739	-€ 36,254,319
3093	0.16	€ 246,092	€ 492,184	€ 738,276	€ 984,368	-€ 5,950,430	-€ 11,900,860	-€ 17,851,290	-€ 23,801,719
3094	0.23	€ 360,505	€ 721,009	##########	##########	-€ 8,716,895	-€ 17,433,791	-€ 26,150,686	-€ 34,867,582
3095	0.22	€ 344,858	€ 689,716	##########	##########	-€ 8,338,560	-€ 16,677,119	-€ 25,015,679	-€ 33,354,239
3096	0.16	€ 246,631	€ 493,262	€ 739,894	€ 986,525	-€ 5,963,470	-€ 11,926,940	-€ 17,890,410	-€ 23,853,880
3097	0.19	€ 300,966	€ 601,932	€ 902,898	##########	-€ 7,277,266	-€ 14,554,532	-€ 21,831,798	-€ 29,109,064
3098	0.20	€ 318,541	€ 637,082	€ 955,623	##########	-€ 7,702,229	-€ 15,404,458	-€ 23,106,687	-€ 30,808,917
3099	0.14	€ 225,639	€ 451,278	€ 676,918	€ 902,557	-€ 5,455,890	-€ 10,911,780	-€ 16,367,670	-€ 21,823,559
3100	0.13	€ 198,978	€ 397,956	€ 596,934	€ 795,912	-€ 4,811,226	-€ 9,622,452	-€ 14,433,678	-€ 19,244,904
3101	0.22	€ 353,589	€ 707,178	##########	##########	-€ 8,549,682	-€ 17,099,365	-€ 25,649,047	-€ 34,198,729
3102	0.19	€ 296,284	€ 592,568	€ 888,852	##########	-€ 7,164,060	-€ 14,328,120	-€ 21,492,181	-€ 28,656,241
3103	0.17	€ 270,229	€ 540,457	€ 810,686	##########	-€ 6,534,045	-€ 13,068,090	-€ 19,602,135	-€ 26,136,180
3104	0.20	€ 320,887	€ 641,773	€ 962,660	##########	-€ 7,758,943	-€ 15,517,886	-€ 23,276,829	-€ 31,035,772
3105	0.14	€ 228,229	€ 456,457	€ 684,686	€ 912,914	-€ 5,518,499	-€ 11,036,997	-€ 16,555,496	-€ 22,073,995
3106	0.18	€ 276,403	€ 552,807	€ 829,210	##########	-€ 6,683,350	-€ 13,366,700	-€ 20,050,050	-€ 26,733,400
3107	0.15	€ 238,150	€ 476,300	€ 714,450	€ 952,601	-€ 5,758,400	-€ 11,516,800	-€ 17,275,201	-€ 23,033,601
3108	0.18	€ 280,288	€ 560,577	€ 840,865	##########	-€ 6,777,289	-€ 13,554,579	-€ 20,331,868	-€ 27,109,157
3109	0.20	€ 312,358	€ 624,717	€ 937,075	##########	-€ 7,552,731	-€ 15,105,461	-€ 22,658,192	-€ 30,210,923
3110	0.15	€ 240,103	€ 480,206	€ 720,310	€ 960,413	-€ 5,805,623	-€ 11,611,247	-€ 17,416,870	-€ 23,222,493
3111	0.14	€ 224,606	€ 449,212	€ 673,818	€ 898,424	-€ 5,430,908	-€ 10,861,816	-€ 16,292,723	-€ 21,723,631
3112	0.15	€ 234,441	€ 468,882	€ 703,323	€ 937,765	-€ 5,668,717	-€ 11,337,434	-€ 17,006,151	-€ 22,674,868
3113	0.22	€ 346,275	€ 692,550	##########	##########	-€ 8,372,823	-€ 16,745,646	-€ 25,118,469	-€ 33,491,291
3114	0.16	€ 250,584	€ 501,167	€ 751,751	##########	-€ 6,059,040	-€ 12,118,081	-€ 18,177,121	-€ 24,236,161
3115	0.26	€ 409,046	€ 818,093	##########	##########	-€ 9,890,620	-€ 19,781,241	-€ 29,671,861	-€ 39,562,482
3116	0.20	€ 315,557	€ 631,115	€ 946,672	##########	-€ 7,630,082	-€ 15,260,164	-€ 22,890,247	-€ 30,520,329
3117	0.20	€ 311,573	€ 623,146	€ 934,720	##########	-€ 7,533,748	-€ 15,067,497	-€ 22,601,245	-€ 30,134,994
3118	0.17	€ 260,764	€ 521,527	€ 782,291	##########	-€ 6,305,190	-€ 12,610,380	-€ 18,915,570	-€ 25,220,760
3119	0.23	€ 359,681	€ 719,362	##########	##########	-€ 8,696,976	-€ 17,393,952	-€ 26,090,927	-€ 34,787,903
3120	0.20	€ 312,487	€ 624,974	€ 937,461	##########	-€ 7,555,843	-€ 15,111,685	-€ 22,667,528	-€ 30,223,370
3121	0.16	€ 258,858	€ 517,715	€ 776,573	##########	-€ 6,259,100	-€ 12,518,200	-€ 18,777,300	-€ 25,036,400
3122	0.14	€ 217,908	€ 435,817	€ 653,725	€ 871,633	-€ 5,268,957	-€ 10,537,914	-€ 15,806,871	-€ 21,075,828
3123	0.21	€ 329,818	€ 659,636	€ 989,454	##########	-€ 7,974,901	-€ 15,949,802	-€ 23,924,703	-€ 31,899,604
3124	0.23	€ 358,268	€ 716,537	##########	##########	-€ 8,662,824	-€ 17,325,649	-€ 25,988,473	-€ 34,651,298
3125	0.22	€ 351,985	€ 703,970	##########	##########	-€ 8,510,897	-€ 17,021,794	-€ 25,532,691	-€ 34,043,587
3126	0.14	€ 216,081	€ 432,162	€ 648,244	€ 864,325	-€ 5,224,779	-€ 10,449,559	-€ 15,674,338	-€ 20,899,117
3127	0.14	€ 219,695	€ 439,389	€ 659,084	€ 878,778	-€ 5,312,149	-€ 10,624,299	-€ 15,936,448	-€ 21,248,597
3128	0.13	€ 211,510	€ 423,021	€ 634,531	€ 846,042	-€ 5,114,259	-€ 10,228,517	-€ 15,342,776	-€ 20,457,035
3129	0.18	€ 276,063	€ 552,127	€ 828,190	##########	-€ 6,675,128	-€ 13,350,257	-€ 20,025,385	-€ 26,700,513
3130	0.17	€ 263,709	€ 527,418	€ 791,126	##########	-€ 6,376,400	-€ 12,752,799	-€ 19,129,199	-€ 25,505,599
3131	0.16	€ 245,388	€ 490,777	€ 736,165	€ 981,554	-€ 5,933,420	-€ 11,866,840	-€ 17,800,260	-€ 23,733,680
3132	0.17	€ 262,818	€ 525,636	€ 788,454	##########	-€ 6,354,860	-€ 12,709,720	-€ 19,064,579	-€ 25,419,439
3133	0.21	€ 331,938	€ 663,875	€ 995,813	##########	-€ 8,026,151	-€ 16,052,303	-€ 24,078,454	-€ 32,104,605
3134	0.14	€ 213,773	€ 427,545	€ 641,318	€ 855,090	-€ 5,168,957	-€ 10,337,914	-€ 15,506,870	-€ 20,675,827
3135	0.18	€ 280,574	€ 561,147	€ 841,721	##########	-€ 6,784,185	-€ 13,568,371	-€ 20,352,556	-€ 27,136,741
3136	0.14	€ 219,597	€ 439,193	€ 658,790	€ 878,386	-€ 5,309,778	-€ 10,619,557	-€ 15,929,335	-€ 21,239,113
3137	0.17	€ 271,649	€ 543,298	€ 814,946	##########	-€ 6,568,388	-€ 13,136,776	-€ 19,705,163	-€ 26,273,551
3138	0.23	€ 364,035	€ 728,069	##########	##########	-€ 8,802,247	-€ 17,604,494	-€ 26,406,741	-€ 35,208,988
3139	0.23	€ 368,103	€ 736,206	##########	##########	-€ 8,900,619	-€ 17,801,238	-€ 26,701,857	-€ 35,602,476
3140	0.24	€ 377,107	€ 754,214	##########	##########	-€ 9,118,333	-€ 18,236,666	-€ 27,354,999	-€ 36,473,332
3141	0.15	€ 228,770	€ 457,539	€ 686,309	€ 915,079	-€ 5,531,584	-€ 11,063,168	-€ 16,594,752	-€ 22,126,336
3142	0.22	€ 341,346	€ 682,693	##########	##########	-€ 8,253,656	-€ 16,507,313	-€ 24,760,969	-€ 33,014,626
3143	0.18	€ 287,403	€ 574,806	€ 862,209	##########	-€ 6,949,319	-€ 13,898,638	-€ 20,847,957	-€ 27,797,276
3144	0.21	€ 331,921	€ 663,843	€ 995,764	##########	-€ 8,025,759	-€ 16,051,518	-€ 24,077,277	-€ 32,103,036
3145	0.15	€ 243,711	€ 487,422	€ 731,133	€ 974,844	-€ 5,892,862	-€ 11,785,724	-€ 17,678,586	-€ 23,571,449
3146	0.21	€ 325,493	€ 650,986	€ 976,478	##########	-€ 7,870,319	-€ 15,740,637	-€ 23,610,956	-€ 31,481,275
3147	0.21	€ 336,060	€ 672,120	##########	##########	-€ 8,125,827	-€ 16,251,655	-€ 24,377,482	-€ 32,503,309
3148	0.18	€ 277,336	€ 554,671	€ 832,007	##########	-€ 6,705,892	-€ 13,411,783	-€ 20,117,675	-€ 26,823,566
3149	0.14	€ 222,779	€ 445,558	€ 668,337	€ 891,116	-€ 5,386,733	-€ 10,773,466	-€ 16,160,199	-€ 21,546,932
3150	0.13	€ 197,208	€ 394,416	€ 591,624	€ 788,832	-€ 4,768,428	-€ 9,536,857	-€ 14,305,285	-€ 19,073,713
3151	0.16	€ 248,103	€ 496,205	€ 744,308	€ 992,410	-€ 5,999,047	-€ 11,998,094	-€ 17,997,141	-€ 23,996,188
3152	0.17	€ 265,513	€ 531,025	€ 796,538	##########	-€ 6,420,019	-€ 12,840,038	-€ 19,260,057	-€ 25,680,076
3153	0.17	€ 260,618	€ 521,235	€ 781,853	##########	-€ 6,301,659	-€ 12,603,318	-€ 18,904,977	-€ 25,206,636
3154	0.21	€ 329,523	€ 659,047	€ 988,570	##########	-€ 7,967,775	-€ 15,935,549	-€ 23,903,324	-€ 31,871,098
3155	0.19	€ 297,092	€ 594,185	€ 891,277	##########	-€ 7,183,604	-€ 14,367,208	-€ 21,550,813	-€ 28,734,417
3156	0.17	€ 273,755	€ 547,510	€ 821,264	##########	-€ 6,619,310	-€ 13,238,619	-€ 19,857,929	-€ 26,477,238
3157	0.22	€ 346,011	€ 692,022	##########	##########	-€ 8,366,448	-€ 16,732,897	-€ 25,099,345	-€ 33,465,794
3158	0.17	€ 275,321	€ 550,642	€ 825,963	##########	-€ 6,657,183	-€ 13,314,365	-€ 19,971,548	-€ 26,628,730

3159	0.18	€ 280,375	€ 560,751	€ 841,126	##########	-€ 6,779,393	-€ 13,558,786	-€ 20,338,178	-€ 27,117,571
3160	0.29	€ 458,886	€ 917,771	##########	##########	-€ 11,095,718	-€ 22,191,435	-€ 33,287,153	-€ 44,382,871
3161	0.25	€ 387,014	€ 774,027	##########	##########	-€ 9,357,875	-€ 18,715,750	-€ 28,073,626	-€ 37,431,501
3162	0.18	€ 278,185	€ 556,370	€ 834,556	##########	-€ 6,726,436	-€ 13,452,871	-€ 20,179,307	-€ 26,905,743
3163	0.26	€ 402,906	€ 805,813	##########	##########	-€ 9,742,155	-€ 19,484,309	-€ 29,226,464	-€ 38,968,619
3164	0.24	€ 376,323	€ 752,645	##########	##########	-€ 9,099,372	-€ 18,198,745	-€ 27,298,117	-€ 36,397,489
3165	0.15	€ 237,982	€ 475,963	€ 713,945	€ 951,926	-€ 5,754,324	-€ 11,508,648	-€ 17,262,971	-€ 23,017,295
3166	0.20	€ 311,618	€ 623,237	€ 934,855	##########	-€ 7,534,837	-€ 15,069,674	-€ 22,604,510	-€ 30,139,347
3167	0.21	€ 329,307	€ 658,613	€ 987,920	##########	-€ 7,962,538	-€ 15,925,075	-€ 23,887,613	-€ 31,850,150
3168	0.12	€ 187,708	€ 375,416	€ 563,123	€ 750,831	-€ 4,538,719	-€ 9,077,438	-€ 13,616,158	-€ 18,154,877
3169	0.11	€ 170,413	€ 340,827	€ 511,240	€ 681,653	-€ 4,120,542	-€ 8,241,084	-€ 12,361,626	-€ 16,482,169
3170	0.14	€ 225,566	€ 451,133	€ 676,699	€ 902,266	-€ 5,454,130	-€ 10,908,261	-€ 16,362,391	-€ 21,816,521
3171	0.24	€ 370,779	€ 741,558	##########	##########	-€ 8,965,331	-€ 17,930,662	-€ 26,895,993	-€ 35,861,324
3172	0.18	€ 280,275	€ 560,551	€ 840,826	##########	-€ 6,776,975	-€ 13,553,950	-€ 20,330,925	-€ 27,107,899
3173	0.26	€ 415,219	€ 830,437	##########	##########	-€ 10,039,861	-€ 20,079,722	-€ 30,119,583	-€ 40,159,444
3174	0.22	€ 342,979	€ 685,959	##########	##########	-€ 8,293,141	-€ 16,586,281	-€ 24,879,422	-€ 33,172,563
3175	0.16	€ 247,678	€ 495,355	€ 743,033	€ 990,710	-€ 5,988,768	-€ 11,977,537	-€ 17,966,305	-€ 23,955,074
3176	0.13	€ 206,863	€ 413,727	€ 620,590	€ 827,453	-€ 5,001,895	-€ 10,003,790	-€ 15,005,685	-€ 20,007,579
3177	0.21	€ 327,191	€ 654,383	€ 981,574	##########	-€ 7,911,391	-€ 15,822,783	-€ 23,734,174	-€ 31,645,565
3178	0.18	€ 283,996	€ 567,992	€ 851,989	##########	-€ 6,866,943	-€ 13,733,887	-€ 20,600,830	-€ 27,467,774
3179	0.14	€ 218,213	€ 436,426	€ 654,640	€ 872,853	-€ 5,276,330	-€ 10,552,660	-€ 15,828,990	-€ 21,105,320
3180	0.14	€ 226,144	€ 452,288	€ 678,431	€ 904,575	-€ 5,468,089	-€ 10,936,178	-€ 16,404,268	-€ 21,872,357
3181	0.21	€ 331,071	€ 662,142	€ 993,213	##########	-€ 8,005,198	-€ 16,010,396	-€ 24,015,594	-€ 32,020,792
3182	0.20	€ 309,098	€ 618,196	€ 927,294	##########	-€ 7,473,900	-€ 14,947,800	-€ 22,421,700	-€ 29,895,600
3183	0.20	€ 318,828	€ 637,656	€ 956,484	##########	-€ 7,709,168	-€ 15,418,336	-€ 23,127,504	-€ 30,836,672
3184	0.09	€ 138,949	€ 277,898	€ 416,848	€ 555,797	-€ 3,359,750	-€ 6,719,500	-€ 10,079,250	-€ 13,439,000
3185	0.28	€ 440,580	€ 881,160	##########	##########	-€ 10,653,096	-€ 21,306,192	-€ 31,959,289	-€ 42,612,385
3186	0.29	€ 456,010	€ 912,021	##########	##########	-€ 11,026,193	-€ 22,052,387	-€ 33,078,580	-€ 44,104,773
3187	0.22	€ 351,204	€ 702,407	##########	##########	-€ 8,491,999	-€ 16,983,998	-€ 25,475,997	-€ 33,967,996
3188	0.20	€ 319,982	€ 639,965	€ 959,947	##########	-€ 7,737,077	-€ 15,474,154	-€ 23,211,230	-€ 30,948,307
3189	0.13	€ 201,199	€ 402,397	€ 603,596	€ 804,794	-€ 4,864,922	-€ 9,729,844	-€ 14,594,766	-€ 19,459,688
3190	0.18	€ 290,728	€ 581,456	€ 872,184	##########	-€ 7,029,713	-€ 14,059,427	-€ 21,089,140	-€ 28,118,853
3191	0.10	€ 162,338	€ 324,675	€ 487,013	€ 649,350	-€ 3,925,275	-€ 7,850,551	-€ 11,775,826	-€ 15,701,101
3192	0.20	€ 310,524	€ 621,048	€ 931,572	##########	-€ 7,508,377	-€ 15,016,753	-€ 22,525,130	-€ 30,033,506
3193	0.17	€ 264,028	€ 528,056	€ 792,084	##########	-€ 6,384,118	-€ 12,768,237	-€ 19,152,355	-€ 25,536,473
3194	0.19	€ 292,882	€ 585,764	€ 878,646	##########	-€ 7,081,800	-€ 14,163,601	-€ 21,245,401	-€ 28,327,201
3195	0.24	€ 376,603	€ 753,207	##########	##########	-€ 9,106,157	-€ 18,212,314	-€ 27,318,471	-€ 36,424,627
3196	0.16	€ 258,142	€ 516,285	€ 774,427	##########	-€ 6,241,809	-€ 12,483,618	-€ 18,725,427	-€ 24,967,235
3197	0.14	€ 214,661	€ 429,321	€ 643,982	€ 858,642	-€ 5,190,428	-€ 10,380,855	-€ 15,571,283	-€ 20,761,710
3198	0.20	€ 318,945	€ 637,890	€ 956,835	##########	-€ 7,711,994	-€ 15,423,988	-€ 23,135,982	-€ 30,847,976
3199	0.18	€ 290,874	€ 581,748	€ 872,622	##########	-€ 7,033,245	-€ 14,066,489	-€ 21,099,734	-€ 28,132,978
3200	0.17	€ 268,683	€ 537,365	€ 806,048	##########	-€ 6,496,666	-€ 12,993,332	-€ 19,489,998	-€ 25,986,664
3201	0.18	€ 284,959	€ 569,919	€ 854,878	##########	-€ 6,890,233	-€ 13,780,467	-€ 20,670,700	-€ 27,560,934
3202	0.20	€ 314,542	€ 629,083	€ 943,625	##########	-€ 7,605,525	-€ 15,211,051	-€ 22,816,576	-€ 30,422,101
3203	0.24	€ 371,586	€ 743,171	##########	##########	-€ 8,984,830	-€ 17,969,660	-€ 26,954,490	-€ 35,939,320
3204	0.18	€ 281,701	€ 563,401	€ 845,102	##########	-€ 6,811,437	-€ 13,622,874	-€ 20,434,312	-€ 27,245,749
3205	0.24	€ 374,310	€ 748,619	##########	##########	-€ 9,050,695	-€ 18,101,389	-€ 27,152,084	-€ 36,202,779
3206	0.15	€ 230,131	€ 460,262	€ 690,393	€ 920,524	-€ 5,564,498	-€ 11,128,996	-€ 16,693,494	-€ 22,257,992
3207	0.20	€ 310,868	€ 621,736	€ 932,605	##########	-€ 7,516,701	-€ 15,033,403	-€ 22,550,104	-€ 30,066,805
3208	0.20	€ 315,884	€ 631,769	€ 947,653	##########	-€ 7,637,991	-€ 15,275,981	-€ 22,913,972	-€ 30,551,963
3209	0.21	€ 323,369	€ 646,739	€ 970,108	##########	-€ 7,818,976	-€ 15,637,951	-€ 23,456,927	-€ 31,275,902
3210	0.18	€ 276,422	€ 552,844	€ 829,267	##########	-€ 6,683,806	-€ 13,367,612	-€ 20,051,419	-€ 26,735,225
3211	0.22	€ 353,509	€ 707,019	##########	##########	-€ 8,547,751	-€ 17,095,502	-€ 25,643,254	-€ 34,191,005
3212	0.14	€ 217,871	€ 435,741	€ 653,612	€ 871,482	-€ 5,268,047	-€ 10,536,094	-€ 15,804,141	-€ 21,072,188
3213	0.24	€ 380,527	€ 761,054	##########	##########	-€ 9,201,026	-€ 18,402,052	-€ 27,603,078	-€ 36,804,103
3214	0.19	€ 297,965	€ 595,930	€ 893,896	##########	-€ 7,204,711	-€ 14,409,422	-€ 21,614,133	-€ 28,818,843
3215	0.16	€ 245,064	€ 490,129	€ 735,193	€ 980,257	-€ 5,925,583	-€ 11,851,165	-€ 17,776,748	-€ 23,702,331
3216	0.12	€ 194,644	€ 389,288	€ 583,932	€ 778,576	-€ 4,706,436	-€ 9,412,871	-€ 14,119,307	-€ 18,825,742
3217	0.24	€ 370,738	€ 741,476	##########	##########	-€ 8,964,340	-€ 17,928,679	-€ 26,893,019	-€ 35,857,359
3218	0.20	€ 322,356	€ 644,713	€ 967,069	##########	-€ 7,794,483	-€ 15,588,966	-€ 23,383,448	-€ 31,177,931
3219	0.16	€ 255,435	€ 510,871	€ 766,306	##########	-€ 6,176,352	-€ 12,352,703	-€ 18,529,055	-€ 24,705,406
3220	0.22	€ 339,521	€ 679,042	##########	##########	-€ 8,209,517	-€ 16,419,034	-€ 24,628,551	-€ 32,838,068
3221	0.20	€ 320,526	€ 641,052	€ 961,578	##########	-€ 7,750,224	-€ 15,500,448	-€ 23,250,671	-€ 31,000,895
3222	0.21	€ 328,991	€ 657,981	€ 986,972	##########	-€ 7,954,897	-€ 15,909,794	-€ 23,864,691	-€ 31,819,588
3223	0.21	€ 325,541	€ 651,082	€ 976,623	##########	-€ 7,871,484	-€ 15,742,967	-€ 23,614,451	-€ 31,485,935
3224	0.24	€ 380,005	€ 760,011	##########	##########	-€ 9,188,420	-€ 18,376,840	-€ 27,565,260	-€ 36,753,679
3225	0.18	€ 288,622	€ 577,243	€ 865,865	##########	-€ 6,978,785	-€ 13,957,570	-€ 20,936,355	-€ 27,915,140
3226	0.18	€ 285,650	€ 571,300	€ 856,950	##########	-€ 6,906,929	-€ 13,813,857	-€ 20,720,786	-€ 27,627,714
3227	0.13	€ 208,300	€ 416,600	€ 624,901	€ 833,201	-€ 5,036,637	-€ 10,073,275	-€ 15,109,912	-€ 20,146,549
3228	0.19	€ 298,684	€ 597,368	€ 896,052	##########	-€ 7,222,090	-€ 14,444,181	-€ 21,666,271	-€ 28,888,361
3229	0.17	€ 272,793	€ 545,587	€ 818,380	##########	-€ 6,596,061	-€ 13,192,123	-€ 19,788,184	-€ 26,384,245
3230	0.20	€ 314,345	€ 628,690	€ 943,035	##########	-€ 7,600,765	-€ 15,201,531	-€ 22,802,296	-€ 30,403,062
3231	0.17	€ 271,447	€ 542,895	€ 814,342	##########	-€ 6,563,514	-€ 13,127,029	-€ 19,690,543	-€ 26,254,058
3232	0.21	€ 327,056	€ 654,113	€ 981,169	##########	-€ 7,908,126	-€ 15,816,252	-€ 23,724,377	-€ 31,632,503
3233	0.17	€ 268,006	€ 536,011	€ 804,017	##########	-€ 6,480,299	-€ 12,960,598	-€ 19,440,897	-€ 25,921,196
3234	0.14	€ 228,033	€ 456,065	€ 684,098	€ 912,130	-€ 5,513,760	-€ 11,027,520	-€ 16,541,279	-€ 22,055,039
3235	0.21	€ 324,372	€ 648,744	€ 973,116	##########	-€ 7,843,217	-€ 15,686,434	-€ 23,529,650	-€ 31,372,867
3236	0.16	€ 246,152	€ 492,304	€ 738,457	€ 984,609	-€ 5,951,887	-€ 11,903,774	-€ 17,855,661	-€ 23,807,549
3237	0.12	€ 181,478	€ 362,956	€ 544,434	€ 725,912	-€ 4,388,083	-€ 8,776,166	-€ 13,164,250	-€ 17,552,333
3238	0.17	€ 271,692	€ 543,383	€ 815,075	##########	-€ 6,569,425	-€ 13,138,850	-€ 19,708,275	-€ 26,277,700
3239	0.21	€ 322,882	€ 645,765	€ 968,647	##########	-€ 7,807,201	-€ 15,614,402	-€ 23,421,603	-€ 31,228,804
3240	0.21	€ 337,415	€ 674,829	##########	##########	-€ 8,158,584	-€ 16,317,168	-€ 24,475,753	-€ 32,634,337
3241	0.16	€ 249,827	€ 499,655	€ 749,482	€ 999,310	-€ 6,040,753	-€ 12,081,506	-€ 18,122,259	-€ 24,163,011
3242	0.18	€ 284,277	€ 568,554	€ 852,830	##########	-€ 6,873,728	-€ 13,747,456	-€ 20,621,184	-€ 27,494,912
3243	0.16	€ 244,839	€ 489,678	€ 734,518	€ 979,357	-€ 5,920,140	-€ 11,840,280	-€ 17,760,421	-€ 23,680,561
3244	0.17	€ 261,541	€ 523,083	€ 784,624	##########	-€ 6,323,992	-€ 12,647,984	-€ 18,971,976	-€ 25,295,968
3245	0.19	€ 300,470	€ 600,941	€ 901,411	##########	-€ 7,265,285	-€ 14,530,570	-€ 21,795,856	-€ 29,061,141
3246	0.16	€ 244,143	€ 488,287	€ 732,430	€ 976,573	-€ 5,903,312	-€ 11,806,624	-€ 17,709,937	-€ 23,613,249
3247	0.15	€ 236,022	€ 472,044	€ 708,066	€ 944,088	-€ 5,706,943	-€ 11,413,887	-€ 17,120,830	-€ 22,827,774
3248	0.18	€ 280,468	€ 560,936	€ 841,404	##########	-€ 6,781,632	-€ 13,563,264	-€ 20,344,896	-€ 27,126,528
3249	0.17	€ 269,879	€ 539,758	€ 809,638	##########	-€ 6,525,598	-€ 13,051,196	-€ 19,576,795	-€ 26,102,393
3250	0.16	€ 259,121	€ 518,241	€ 777,362	##########	-€ 6,265,462	-€ 12,530,923	-€ 18,796,385	-€ 25,061,847
3251	0.20	€ 309,063	€ 618,125	€ 927,188	##########	-€ 7,473,043	-€ 14,946,087	-€ 22,419,130	-€ 29,892,173
3252	0.20	€ 309,309	€ 618,619	€ 927,928	##########	-€ 7,479,008	-€ 14,958,016	-€ 22,437,024	-€ 29,916,031
3253	0.21	€ 334,871	€ 669,742	##########	##########	-€ 8,097,077	-€ 16,194,154	-€ 24,291,231	-€ 32,388,308
3254	0.22	€ 346,551	€ 693,102	##########	##########	-€ 8,379,497	-€ 16,758,995	-€ 25,138,492	-€ 33,517,989
3255	0.28	€ 436,833	€ 873,667	##########	##########	-€ 10,562,499	-€ 21,124,999	-€ 31,687,498	-€ 42,249,998
3256	0.13	€ 203,683	€ 407,367	€ 611,050	€ 814,733	-€ 4,925,002	-€ 9,850,003	-€ 14,775,005	-€ 19,700,006
3257	0.15	€ 239,593	€ 479,185	€ 718,778	€ 958,370	-€ 5,793,277	-€ 11,586,554	-€ 17,379,831	-€ 23,173,108
3258	0.10	€ 162,579	€ 325,157	€ 487,736	€ 650,315	-€ 3,931,103	-€ 7,862,206	-€ 11,793,309	-€ 15,724,412
3259	0.22	€ 350,557	€ 701,114	##########	##########	-€ 8,476,363	-€ 16,952,726	-€ 25,429,089	-€ 33,905,453
3260	0.17	€ 271,716	€ 543,432	€ 815,149	##########	-€ 6,570,018	-€ 13,140,035	-€ 19,710,053	-€ 26,280,070

3261	0.19	€ 296,872	€ 593,744	€ 890,615	##########	-€ 7,178,271	-€ 14,356,543	-€ 21,534,814	-€ 28,713,086
3262	0.23	€ 366,221	€ 732,442	##########	##########	-€ 8,855,113	-€ 17,710,227	-€ 26,565,340	-€ 35,420,453
3263	0.27	€ 425,085	€ 850,170	##########	##########	-€ 10,278,425	-€ 20,556,851	-€ 30,835,276	-€ 41,113,702
3264	0.22	€ 348,843	€ 697,686	##########	##########	-€ 8,434,925	-€ 16,869,851	-€ 25,304,776	-€ 33,739,702
3265	0.21	€ 331,784	€ 663,569	€ 995,353	##########	-€ 8,022,450	-€ 16,044,899	-€ 24,067,349	-€ 32,089,799
3266	0.23	€ 362,790	€ 725,580	##########	##########	-€ 8,772,159	-€ 17,544,318	-€ 26,316,478	-€ 35,088,637
3267	0.12	€ 190,191	€ 380,383	€ 570,574	€ 760,766	-€ 4,598,771	-€ 9,197,542	-€ 13,796,313	-€ 18,395,084
3268	0.19	€ 292,574	€ 585,148	€ 877,722	##########	-€ 7,074,355	-€ 14,148,709	-€ 21,223,064	-€ 28,297,419
3269	0.22	€ 348,176	€ 696,353	##########	##########	-€ 8,418,804	-€ 16,837,608	-€ 25,256,413	-€ 33,675,217
3270	0.18	€ 283,476	€ 566,952	€ 850,429	##########	-€ 6,854,371	-€ 13,708,743	-€ 20,563,114	-€ 27,417,486
3271	0.28	€ 433,673	€ 867,347	##########	##########	-€ 10,486,096	-€ 20,972,192	-€ 31,458,288	-€ 41,944,384
3272	0.17	€ 274,454	€ 548,907	€ 823,361	##########	-€ 6,636,210	-€ 13,272,420	-€ 19,908,630	-€ 26,544,840
3273	0.16	€ 255,361	€ 510,722	€ 766,083	##########	-€ 6,174,557	-€ 12,349,114	-€ 18,523,671	-€ 24,698,228
3274	0.21	€ 330,581	€ 661,162	€ 991,743	##########	-€ 7,993,349	-€ 15,986,697	-€ 23,980,046	-€ 31,973,395
3275	0.20	€ 315,771	€ 631,542	€ 947,314	##########	-€ 7,635,253	-€ 15,270,507	-€ 22,905,760	-€ 30,541,014
3276	0.20	€ 317,609	€ 635,217	€ 952,826	##########	-€ 7,679,680	-€ 15,359,360	-€ 23,039,040	-€ 30,718,720
3277	0.25	€ 392,578	€ 785,156	##########	##########	-€ 9,492,416	-€ 18,984,833	-€ 28,477,249	-€ 37,969,665
3278	0.18	€ 285,636	€ 571,271	€ 856,907	##########	-€ 6,906,588	-€ 13,813,175	-€ 20,719,763	-€ 27,626,350
3279	0.14	€ 223,401	€ 446,803	€ 670,204	€ 893,606	-€ 5,401,781	-€ 10,803,563	-€ 16,205,344	-€ 21,607,125
3280	0.19	€ 306,360	€ 612,719	€ 919,079	##########	-€ 7,407,683	-€ 14,815,366	-€ 22,223,049	-€ 29,630,732
3281	0.19	€ 301,930	€ 603,861	€ 905,791	##########	-€ 7,300,589	-€ 14,601,178	-€ 21,901,768	-€ 29,202,357
3282	0.20	€ 315,497	€ 630,993	€ 946,490	##########	-€ 7,628,615	-€ 15,257,229	-€ 22,885,844	-€ 30,514,458
3283	0.21	€ 329,164	€ 658,329	€ 987,493	##########	-€ 7,959,095	-€ 15,918,190	-€ 23,877,286	-€ 31,836,381
3284	0.25	€ 396,969	€ 793,938	##########	##########	-€ 9,598,594	-€ 19,197,187	-€ 28,795,781	-€ 38,394,374
3285	0.26	€ 403,876	€ 807,751	##########	##########	-€ 9,765,595	-€ 19,531,190	-€ 29,296,785	-€ 39,062,380
3286	0.18	€ 281,659	€ 563,318	€ 844,977	##########	-€ 6,810,434	-€ 13,620,867	-€ 20,431,301	-€ 27,241,734
3287	0.20	€ 311,860	€ 623,719	€ 935,579	##########	-€ 7,540,673	-€ 15,081,346	-€ 22,622,019	-€ 30,162,692
3288	0.24	€ 373,521	€ 747,041	##########	##########	-€ 9,031,615	-€ 18,063,230	-€ 27,094,845	-€ 36,126,461
3289	0.19	€ 292,762	€ 585,524	€ 878,286	##########	-€ 7,078,897	-€ 14,157,793	-€ 21,236,690	-€ 28,315,586
3290	0.20	€ 309,247	€ 618,493	€ 927,740	##########	-€ 7,477,493	-€ 14,954,985	-€ 22,432,478	-€ 29,909,970
3291	0.22	€ 343,738	€ 687,476	##########	##########	-€ 8,311,489	-€ 16,622,977	-€ 24,934,466	-€ 33,245,954
3292	0.20	€ 316,517	€ 633,034	€ 949,551	##########	-€ 7,653,290	-€ 15,306,580	-€ 22,959,870	-€ 30,613,160
3293	0.17	€ 266,459	€ 532,918	€ 799,378	##########	-€ 6,442,905	-€ 12,885,811	-€ 19,328,716	-€ 25,771,622
3294	0.16	€ 255,530	€ 511,060	€ 766,591	##########	-€ 6,178,644	-€ 12,357,289	-€ 18,535,933	-€ 24,714,577
3295	0.24	€ 370,283	€ 740,567	##########	##########	-€ 8,953,340	-€ 17,906,679	-€ 26,860,019	-€ 35,813,359
3296	0.25	€ 387,500	€ 774,999	##########	##########	-€ 9,369,625	-€ 18,739,250	-€ 28,108,874	-€ 37,478,499
3297	0.16	€ 258,763	€ 517,527	€ 776,290	##########	-€ 6,256,822	-€ 12,513,644	-€ 18,770,466	-€ 25,027,289
3298	0.12	€ 191,506	€ 383,011	€ 574,517	€ 766,023	-€ 4,630,549	-€ 9,261,098	-€ 13,891,648	-€ 18,522,197
3299	0.16	€ 258,667	€ 517,334	€ 776,001	##########	-€ 6,254,493	-€ 12,508,987	-€ 18,763,480	-€ 25,017,973
3300	0.17	€ 261,290	€ 522,580	€ 783,869	##########	-€ 6,317,909	-€ 12,635,817	-€ 18,953,726	-€ 25,271,635
3301	0.17	€ 265,604	€ 531,208	€ 796,813	##########	-€ 6,422,231	-€ 12,844,462	-€ 19,266,693	-€ 25,688,924
3302	0.22	€ 346,879	€ 693,759	##########	##########	-€ 8,387,438	-€ 16,774,876	-€ 25,162,314	-€ 33,549,753
3303	0.17	€ 269,552	€ 539,103	€ 808,655	##########	-€ 6,517,676	-€ 13,035,352	-€ 19,553,028	-€ 26,070,703
3304	0.24	€ 371,592	€ 743,183	##########	##########	-€ 8,984,972	-€ 17,969,945	-€ 26,954,917	-€ 35,939,889
3305	0.30	€ 464,551	€ 929,102	##########	##########	-€ 11,232,701	-€ 22,465,403	-€ 33,698,104	-€ 44,930,805
3306	0.19	€ 303,438	€ 606,877	€ 910,315	##########	-€ 7,337,050	-€ 14,674,099	-€ 22,011,149	-€ 29,348,198
3307	0.27	€ 429,761	€ 859,522	##########	##########	-€ 10,391,499	-€ 20,782,998	-€ 31,174,496	-€ 41,565,995
3308	0.17	€ 264,954	€ 529,909	€ 794,863	##########	-€ 6,406,519	-€ 12,813,037	-€ 19,219,556	-€ 25,626,074
3309	0.15	€ 234,335	€ 468,670	€ 703,005	€ 937,340	-€ 5,666,153	-€ 11,332,307	-€ 16,998,460	-€ 22,664,613
3310	0.12	€ 194,607	€ 389,214	€ 583,821	€ 778,428	-€ 4,705,537	-€ 9,411,075	-€ 14,116,612	-€ 18,822,150
3311	0.21	€ 324,505	€ 649,010	€ 973,515	##########	-€ 7,846,435	-€ 15,692,871	-€ 23,539,306	-€ 31,385,741
3312	0.19	€ 301,093	€ 602,187	€ 903,280	##########	-€ 7,280,349	-€ 14,560,698	-€ 21,841,048	-€ 29,121,397
3313	0.18	€ 281,676	€ 563,353	€ 845,029	##########	-€ 6,810,853	-€ 13,621,705	-€ 20,432,558	-€ 27,243,411
3314	0.18	€ 288,561	€ 577,121	€ 865,682	##########	-€ 6,977,313	-€ 13,954,625	-€ 20,931,938	-€ 27,909,251
3315	0.16	€ 256,458	€ 512,916	€ 769,374	##########	-€ 6,201,081	-€ 12,402,163	-€ 18,603,244	-€ 24,804,326
3316	0.12	€ 191,981	€ 383,962	€ 575,944	€ 767,925	-€ 4,642,048	-€ 9,284,097	-€ 13,926,145	-€ 18,568,194
3317	0.22	€ 340,113	€ 680,226	##########	##########	-€ 8,223,831	-€ 16,447,662	-€ 24,671,493	-€ 32,895,324
3318	0.25	€ 390,758	€ 781,516	##########	##########	-€ 9,448,410	-€ 18,896,820	-€ 28,345,230	-€ 37,793,640
3319	0.15	€ 231,089	€ 462,178	€ 693,266	€ 924,355	-€ 5,587,659	-€ 11,175,318	-€ 16,762,977	-€ 22,350,636
3320	0.14	€ 212,603	€ 425,205	€ 637,808	€ 850,411	-€ 5,140,671	-€ 10,281,342	-€ 15,422,013	-€ 20,562,683
3321	0.14	€ 224,591	€ 449,183	€ 673,774	€ 898,365	-€ 5,430,551	-€ 10,861,102	-€ 16,291,653	-€ 21,722,204
3322	0.23	€ 365,855	€ 731,710	##########	##########	-€ 8,846,265	-€ 17,692,530	-€ 26,538,795	-€ 35,385,059
3323	0.22	€ 349,820	€ 699,639	##########	##########	-€ 8,458,535	-€ 16,917,070	-€ 25,375,605	-€ 33,834,140
3324	0.16	€ 247,532	€ 495,063	€ 742,595	€ 990,126	-€ 5,985,239	-€ 11,970,478	-€ 17,955,718	-€ 23,940,957
3325	0.16	€ 253,073	€ 506,145	€ 759,218	##########	-€ 6,119,219	-€ 12,238,439	-€ 18,357,658	-€ 24,476,878
3326	0.14	€ 224,576	€ 449,152	€ 673,729	€ 898,305	-€ 5,430,186	-€ 10,860,372	-€ 16,290,559	-€ 21,720,745
3327	0.15	€ 234,003	€ 468,005	€ 702,008	€ 936,011	-€ 5,658,116	-€ 11,316,233	-€ 16,974,349	-€ 22,632,466
3328	0.21	€ 325,806	€ 651,613	€ 977,419	##########	-€ 7,877,904	-€ 15,755,808	-€ 23,633,713	-€ 31,511,617
3329	0.16	€ 252,712	€ 505,424	€ 758,137	##########	-€ 6,110,506	-€ 12,221,012	-€ 18,331,518	-€ 24,442,024
3330	0.19	€ 304,201	€ 608,402	€ 912,602	##########	-€ 7,355,484	-€ 14,710,969	-€ 22,066,453	-€ 29,421,937
3331	0.17	€ 264,208	€ 528,416	€ 792,624	##########	-€ 6,388,470	-€ 12,776,939	-€ 19,165,409	-€ 25,553,879
3332	0.17	€ 273,961	€ 547,922	€ 821,883	##########	-€ 6,624,299	-€ 13,248,598	-€ 19,872,898	-€ 26,497,197
3333	0.14	€ 216,842	€ 433,683	€ 650,525	€ 867,367	-€ 5,243,168	-€ 10,486,336	-€ 15,729,503	-€ 20,972,671
3334	0.16	€ 256,313	€ 512,626	€ 768,939	##########	-€ 6,197,572	-€ 12,395,145	-€ 18,592,717	-€ 24,790,289
3335	0.25	€ 389,762	€ 779,525	##########	##########	-€ 9,424,339	-€ 18,848,677	-€ 28,273,016	-€ 37,697,355
3336	0.20	€ 319,802	€ 639,605	€ 959,407	##########	-€ 7,732,726	-€ 15,465,452	-€ 23,198,178	-€ 30,930,904
3337	0.21	€ 328,757	€ 657,513	€ 986,270	##########	-€ 7,949,240	-€ 15,898,479	-€ 23,847,719	-€ 31,796,958
3338	0.16	€ 244,562	€ 489,123	€ 733,685	€ 978,246	-€ 5,913,427	-€ 11,826,855	-€ 17,740,282	-€ 23,653,710
3339	0.21	€ 334,607	€ 669,215	##########	##########	-€ 8,090,707	-€ 16,181,413	-€ 24,272,120	-€ 32,362,826
3340	0.24	€ 378,478	€ 756,955	##########	##########	-€ 9,151,475	-€ 18,302,949	-€ 27,454,424	-€ 36,605,899
3341	0.18	€ 288,429	€ 576,859	€ 865,288	##########	-€ 6,974,138	-€ 13,948,276	-€ 20,922,414	-€ 27,896,552
3342	0.19	€ 298,785	€ 597,570	€ 896,355	##########	-€ 7,224,532	-€ 14,449,064	-€ 21,673,597	-€ 28,898,129
3343	0.23	€ 365,797	€ 731,595	##########	##########	-€ 8,844,870	-€ 17,689,741	-€ 26,534,611	-€ 35,379,481
3344	0.23	€ 365,520	€ 731,040	##########	##########	-€ 8,838,165	-€ 17,676,331	-€ 26,514,496	-€ 35,352,661
3345	0.21	€ 326,742	€ 653,485	€ 980,227	##########	-€ 7,900,536	-€ 15,801,072	-€ 23,701,609	-€ 31,602,145
3346	0.25	€ 400,127	€ 800,254	##########	##########	-€ 9,674,949	-€ 19,349,897	-€ 29,024,846	-€ 38,699,795
3347	0.15	€ 239,664	€ 479,327	€ 718,991	€ 958,655	-€ 5,794,996	-€ 11,589,991	-€ 17,384,987	-€ 23,179,983
3348	0.19	€ 305,970	€ 611,940	€ 917,911	##########	-€ 7,398,270	-€ 14,796,539	-€ 22,194,809	-€ 29,593,079
3349	0.11	€ 175,603	€ 351,207	€ 526,810	€ 702,414	-€ 4,246,038	-€ 8,492,077	-€ 12,738,115	-€ 16,984,154
3350	0.27	€ 432,251	€ 864,502	##########	##########	-€ 10,451,699	-€ 20,903,398	-€ 31,355,097	-€ 41,806,796
3351	0.22	€ 348,273	€ 696,545	##########	##########	-€ 8,421,130	-€ 16,842,260	-€ 25,263,390	-€ 33,684,520
3352	0.18	€ 278,886	€ 557,773	€ 836,659	##########	-€ 6,743,389	-€ 13,486,778	-€ 20,230,167	-€ 26,973,556
3353	0.15	€ 234,282	€ 468,564	€ 702,846	€ 937,128	-€ 5,664,872	-€ 11,329,743	-€ 16,994,615	-€ 22,659,487
3354	0.17	€ 262,206	€ 524,412	€ 786,618	##########	-€ 6,340,060	-€ 12,680,121	-€ 19,020,181	-€ 25,360,242
3355	0.27	€ 418,664	€ 837,329	##########	##########	-€ 10,123,181	-€ 20,246,361	-€ 30,369,542	-€ 40,492,723
3356	0.20	€ 319,697	€ 639,394	€ 959,091	##########	-€ 7,730,175	-€ 15,460,351	-€ 23,190,526	-€ 30,920,701
3357	0.17	€ 260,346	€ 520,691	€ 781,037	##########	-€ 6,295,078	-€ 12,590,155	-€ 18,885,233	-€ 25,180,310
3358	0.16	€ 255,924	€ 511,847	€ 767,771	##########	-€ 6,188,159	-€ 12,376,317	-€ 18,564,476	-€ 24,752,635
3359	0.17	€ 264,139	€ 528,277	€ 792,416	##########	-€ 6,386,791	-€ 12,773,582	-€ 19,160,373	-€ 25,547,163
3360	0.18	€ 284,428	€ 568,857	€ 853,285	##########	-€ 6,877,392	-€ 13,754,784	-€ 20,632,176	-€ 27,509,568
3361	0.19	€ 294,092	€ 588,184	€ 882,276	##########	-€ 7,111,057	-€ 14,222,115	-€ 21,333,172	-€ 28,444,229
3362	0.18	€ 281,916	€ 563,832	€ 845,748	##########	-€ 6,816,646	-€ 13,633,292	-€ 20,449,938	-€ 27,266,584

3363	0.19	€ 302,076	€ 604,152	€ 906,228	##########	-€ 7,304,109	-€ 14,608,217	-€ 21,912,326	-€ 29,216,435
3364	0.12	€ 195,143	€ 390,286	€ 585,429	€ 780,571	-€ 4,718,496	-€ 9,436,993	-€ 14,155,489	-€ 18,873,985
3365	0.14	€ 214,864	€ 429,728	€ 644,593	€ 859,457	-€ 5,195,352	-€ 10,390,704	-€ 15,586,056	-€ 20,781,408
3366	0.18	€ 279,399	€ 558,799	€ 838,198	##########	-€ 6,755,792	-€ 13,511,583	-€ 20,267,375	-€ 27,023,167
3367	0.19	€ 305,949	€ 611,898	€ 917,847	##########	-€ 7,397,759	-€ 14,795,518	-€ 22,193,277	-€ 29,591,036
3368	0.20	€ 309,694	€ 619,388	€ 929,081	##########	-€ 7,488,303	-€ 14,976,606	-€ 22,464,910	-€ 29,953,213
3369	0.20	€ 322,720	€ 645,440	€ 968,160	##########	-€ 7,803,272	-€ 15,606,545	-€ 23,409,817	-€ 31,213,090
3370	0.24	€ 379,445	€ 758,891	##########	##########	-€ 9,174,874	-€ 18,349,747	-€ 27,524,621	-€ 36,699,494
3371	0.23	€ 354,591	€ 709,183	##########	##########	-€ 8,573,912	-€ 17,147,825	-€ 25,721,737	-€ 34,295,649
3372	0.24	€ 373,274	€ 746,548	##########	##########	-€ 9,025,658	-€ 18,051,317	-€ 27,076,975	-€ 36,102,633
3373	0.19	€ 299,644	€ 599,289	€ 898,933	##########	-€ 7,245,315	-€ 14,490,630	-€ 21,735,944	-€ 28,981,259
3374	0.18	€ 281,858	€ 563,715	€ 845,573	##########	-€ 6,815,233	-€ 13,630,466	-€ 20,445,699	-€ 27,260,932
3375	0.17	€ 273,365	€ 546,730	€ 820,095	##########	-€ 6,609,887	-€ 13,219,774	-€ 19,829,661	-€ 26,439,548
3376	0.20	€ 318,252	€ 636,503	€ 954,755	##########	-€ 7,695,233	-€ 15,390,465	-€ 23,085,698	-€ 30,780,931
3377	0.19	€ 302,636	€ 605,273	€ 907,909	##########	-€ 7,317,655	-€ 14,635,310	-€ 21,952,965	-€ 29,270,620
3378	0.22	€ 348,685	€ 697,370	##########	##########	-€ 8,431,106	-€ 16,862,212	-€ 25,293,317	-€ 33,724,423
3379	0.21	€ 325,968	€ 651,935	€ 977,903	##########	-€ 7,881,799	-€ 15,763,598	-€ 23,645,397	-€ 31,527,196
3380	0.20	€ 313,752	€ 627,503	€ 941,255	##########	-€ 7,586,420	-€ 15,172,840	-€ 22,759,260	-€ 30,345,680
3381	0.12	€ 185,145	€ 370,290	€ 555,435	€ 740,580	-€ 4,476,749	-€ 8,953,498	-€ 13,430,247	-€ 17,906,996
3382	0.20	€ 314,315	€ 628,630	€ 942,945	##########	-€ 7,600,045	-€ 15,200,090	-€ 22,800,135	-€ 30,400,179
3383	0.24	€ 378,365	€ 756,730	##########	##########	-€ 9,148,748	-€ 18,297,496	-€ 27,446,244	-€ 36,594,992
3384	0.28	€ 438,745	€ 877,489	##########	##########	-€ 10,608,716	-€ 21,217,431	-€ 31,826,147	-€ 42,434,863
3385	0.18	€ 284,294	€ 568,587	€ 852,881	##########	-€ 6,874,138	-€ 13,748,277	-€ 20,622,415	-€ 27,496,553
3386	0.17	€ 263,671	€ 527,342	€ 791,013	##########	-€ 6,375,486	-€ 12,750,972	-€ 19,126,458	-€ 25,501,944
3387	0.28	€ 433,665	€ 867,330	##########	##########	-€ 10,485,889	-€ 20,971,778	-€ 31,457,668	-€ 41,943,557
3388	0.23	€ 363,442	€ 726,884	##########	##########	-€ 8,787,921	-€ 17,575,842	-€ 26,363,762	-€ 35,151,683
3389	0.20	€ 315,450	€ 630,899	€ 946,349	##########	-€ 7,627,480	-€ 15,254,960	-€ 22,882,440	-€ 30,509,920
3390	0.17	€ 265,774	€ 531,548	€ 797,322	##########	-€ 6,426,339	-€ 12,852,677	-€ 19,279,016	-€ 25,705,354
3391	0.20	€ 311,051	€ 622,102	€ 933,153	##########	-€ 7,521,124	-€ 15,042,248	-€ 22,563,372	-€ 30,084,496
3392	0.16	€ 244,294	€ 488,589	€ 732,883	€ 977,177	-€ 5,906,965	-€ 11,813,931	-€ 17,720,896	-€ 23,627,861
3393	0.19	€ 298,649	€ 597,297	€ 895,946	##########	-€ 7,221,236	-€ 14,442,471	-€ 21,663,707	-€ 28,884,942
3394	0.21	€ 337,122	€ 674,244	##########	##########	-€ 8,151,505	-€ 16,303,010	-€ 24,454,515	-€ 32,606,020
3395	0.20	€ 307,856	€ 615,712	€ 923,568	##########	-€ 7,443,869	-€ 14,887,738	-€ 22,331,606	-€ 29,775,475
3396	0.17	€ 269,064	€ 538,127	€ 807,191	##########	-€ 6,505,877	-€ 13,011,754	-€ 19,517,631	-€ 26,023,508
3397	0.11	€ 180,747	€ 361,493	€ 542,240	€ 722,987	-€ 4,370,402	-€ 8,740,804	-€ 13,111,206	-€ 17,481,608
3398	0.20	€ 318,843	€ 637,687	€ 956,530	##########	-€ 7,709,537	-€ 15,419,074	-€ 23,128,611	-€ 30,838,148
3399	0.22	€ 343,204	€ 686,409	##########	##########	-€ 8,298,580	-€ 16,597,160	-€ 24,895,740	-€ 33,194,319
3400	0.18	€ 281,350	€ 562,700	€ 844,050	##########	-€ 6,802,961	-€ 13,605,921	-€ 20,408,882	-€ 27,211,842
3401	0.17	€ 268,508	€ 537,016	€ 805,524	##########	-€ 6,492,443	-€ 12,984,885	-€ 19,477,328	-€ 25,969,771
3402	0.26	€ 404,951	€ 809,901	##########	##########	-€ 9,791,587	-€ 19,583,174	-€ 29,374,761	-€ 39,166,347
3403	0.22	€ 345,465	€ 690,931	##########	##########	-€ 8,353,248	-€ 16,706,495	-€ 25,059,743	-€ 33,412,990
3404	0.23	€ 363,901	€ 727,802	##########	##########	-€ 8,799,019	-€ 17,598,039	-€ 26,397,058	-€ 35,196,077
3405	0.14	€ 220,550	€ 441,099	€ 661,649	€ 882,198	-€ 5,332,823	-€ 10,665,645	-€ 15,998,468	-€ 21,331,290
3406	0.21	€ 336,510	€ 673,019	##########	##########	-€ 8,136,706	-€ 16,273,411	-€ 24,410,117	-€ 32,546,822
3407	0.15	€ 232,838	€ 465,675	€ 698,513	€ 931,350	-€ 5,629,945	-€ 11,259,889	-€ 16,889,834	-€ 22,519,778
3408	0.19	€ 293,006	€ 586,012	€ 879,018	##########	-€ 7,084,798	-€ 14,169,596	-€ 21,254,395	-€ 28,339,193
3409	0.19	€ 302,090	€ 604,181	€ 906,271	##########	-€ 7,304,458	-€ 14,608,917	-€ 21,913,375	-€ 29,217,834
3410	0.24	€ 371,911	€ 743,823	##########	##########	-€ 8,992,705	-€ 17,985,410	-€ 26,978,115	-€ 35,970,820
3411	0.23	€ 364,984	€ 729,968	##########	##########	-€ 8,825,210	-€ 17,650,421	-€ 26,475,631	-€ 35,300,841
3412	0.20	€ 321,983	€ 643,966	€ 965,949	##########	-€ 7,785,456	-€ 15,570,912	-€ 23,356,368	-€ 31,141,824
3413	0.25	€ 400,575	€ 801,150	##########	##########	-€ 9,685,786	-€ 19,371,572	-€ 29,057,358	-€ 38,743,143
3414	0.22	€ 340,302	€ 680,604	##########	##########	-€ 8,228,400	-€ 16,456,799	-€ 24,685,199	-€ 32,913,598
3415	0.15	€ 231,821	€ 463,642	€ 695,463	€ 927,285	-€ 5,605,367	-€ 11,210,733	-€ 16,816,100	-€ 22,421,467
3416	0.22	€ 349,066	€ 698,131	##########	##########	-€ 8,440,303	-€ 16,880,607	-€ 25,320,910	-€ 33,761,213
3417	0.13	€ 201,956	€ 403,912	€ 605,869	€ 807,825	-€ 4,883,241	-€ 9,766,483	-€ 14,649,724	-€ 19,532,966
3418	0.19	€ 294,229	€ 588,458	€ 882,687	##########	-€ 7,114,369	-€ 14,228,738	-€ 21,343,107	-€ 28,457,477
3419	0.19	€ 300,093	€ 600,186	€ 900,279	##########	-€ 7,256,161	-€ 14,512,321	-€ 21,768,482	-€ 29,024,643
3420	0.19	€ 298,695	€ 597,390	€ 896,084	##########	-€ 7,222,350	-€ 14,444,701	-€ 21,667,051	-€ 28,889,402
3421	0.14	€ 218,007	€ 436,013	€ 654,020	€ 872,027	-€ 5,271,338	-€ 10,542,676	-€ 15,814,014	-€ 21,085,352
3422	0.19	€ 302,141	€ 604,281	€ 906,422	##########	-€ 7,305,669	-€ 14,611,339	-€ 21,917,008	-€ 29,222,678
3423	0.20	€ 317,344	€ 634,689	€ 952,033	##########	-€ 7,673,294	-€ 15,346,588	-€ 23,019,882	-€ 30,693,176
3424	0.19	€ 293,215	€ 586,431	€ 879,646	##########	-€ 7,089,862	-€ 14,179,725	-€ 21,269,587	-€ 28,359,450
3425	0.17	€ 266,559	€ 533,118	€ 799,678	##########	-€ 6,445,323	-€ 12,890,645	-€ 19,335,968	-€ 25,781,291
3426	0.21	€ 326,160	€ 652,319	€ 978,479	##########	-€ 7,886,445	-€ 15,772,890	-€ 23,659,335	-€ 31,545,779
3427	0.20	€ 309,418	€ 618,836	€ 928,255	##########	-€ 7,481,640	-€ 14,963,279	-€ 22,444,919	-€ 29,926,558
3428	0.15	€ 236,077	€ 472,153	€ 708,230	€ 944,306	-€ 5,708,260	-€ 11,416,519	-€ 17,124,779	-€ 22,833,039
3429	0.15	€ 234,647	€ 469,294	€ 703,941	€ 938,588	-€ 5,673,693	-€ 11,347,386	-€ 17,021,079	-€ 22,694,772
3430	0.16	€ 255,444	€ 510,888	€ 766,332	##########	-€ 6,176,558	-€ 12,353,116	-€ 18,529,674	-€ 24,706,232
3431	0.18	€ 288,319	€ 576,638	€ 864,957	##########	-€ 6,971,464	-€ 13,942,928	-€ 20,914,392	-€ 27,885,856
3432	0.17	€ 269,689	€ 539,379	€ 809,068	##########	-€ 6,521,011	-€ 13,042,023	-€ 19,563,034	-€ 26,084,046
3433	0.17	€ 262,843	€ 525,685	€ 788,528	##########	-€ 6,355,456	-€ 12,710,912	-€ 19,066,368	-€ 25,421,824
3434	0.18	€ 278,768	€ 557,535	€ 836,303	##########	-€ 6,740,516	-€ 13,481,031	-€ 20,221,547	-€ 26,962,063
3435	0.18	€ 286,719	€ 573,439	€ 860,158	##########	-€ 6,932,788	-€ 13,865,575	-€ 20,798,363	-€ 27,731,150
3436	0.22	€ 343,472	€ 686,943	##########	##########	-€ 8,305,044	-€ 16,610,088	-€ 24,915,131	-€ 33,220,175
3437	0.23	€ 365,619	€ 731,237	##########	##########	-€ 8,840,550	-€ 17,681,100	-€ 26,521,650	-€ 35,362,200
3438	0.20	€ 315,624	€ 631,248	€ 946,872	##########	-€ 7,631,696	-€ 15,263,393	-€ 22,895,089	-€ 30,526,786
3439	0.13	€ 206,684	€ 413,369	€ 620,053	€ 826,737	-€ 4,997,567	-€ 9,995,133	-€ 14,992,700	-€ 19,990,267
3440	0.15	€ 232,604	€ 465,208	€ 697,812	€ 930,417	-€ 5,624,299	-€ 11,248,598	-€ 16,872,896	-€ 22,497,195
3441	0.16	€ 250,338	€ 500,676	€ 751,014	##########	-€ 6,053,098	-€ 12,106,196	-€ 18,159,295	-€ 24,212,393
3442	0.20	€ 312,011	€ 624,021	€ 936,032	##########	-€ 7,544,324	-€ 15,088,647	-€ 22,632,971	-€ 30,177,294
3443	0.26	€ 406,268	€ 812,537	##########	##########	-€ 9,823,448	-€ 19,646,895	-€ 29,470,343	-€ 39,293,790
3444	0.19	€ 291,401	€ 582,802	€ 874,204	##########	-€ 7,045,994	-€ 14,091,989	-€ 21,137,983	-€ 28,183,977
3445	0.21	€ 325,672	€ 651,344	€ 977,015	##########	-€ 7,874,648	-€ 15,749,296	-€ 23,623,944	-€ 31,498,592
3446	0.16	€ 253,102	€ 506,205	€ 759,307	##########	-€ 6,119,941	-€ 12,239,883	-€ 18,359,824	-€ 24,479,766
3447	0.22	€ 350,251	€ 700,502	##########	##########	-€ 8,468,969	-€ 16,937,938	-€ 25,406,906	-€ 33,875,875
3448	0.15	€ 239,786	€ 479,571	€ 719,357	€ 959,143	-€ 5,797,948	-€ 11,595,896	-€ 17,393,844	-€ 23,191,791
3449	0.15	€ 237,059	€ 474,118	€ 711,177	€ 948,236	-€ 5,732,019	-€ 11,464,038	-€ 17,196,056	-€ 22,928,075
3450	0.15	€ 243,524	€ 487,048	€ 730,572	€ 974,096	-€ 5,888,339	-€ 11,776,678	-€ 17,665,017	-€ 23,553,355
3451	0.23	€ 369,574	€ 739,147	##########	##########	-€ 8,936,182	-€ 17,872,365	-€ 26,808,547	-€ 35,744,730
3452	0.22	€ 347,798	€ 695,595	##########	##########	-€ 8,409,640	-€ 16,819,281	-€ 25,228,921	-€ 33,638,561
3453	0.12	€ 188,189	€ 376,377	€ 564,566	€ 752,754	-€ 4,550,342	-€ 9,100,685	-€ 13,651,027	-€ 18,201,369
3454	0.28	€ 446,711	€ 893,423	##########	##########	-€ 10,801,350	-€ 21,602,700	-€ 32,404,050	-€ 43,205,400
3455	0.26	€ 417,243	€ 834,485	##########	##########	-€ 10,088,801	-€ 20,177,602	-€ 30,266,403	-€ 40,355,204
3456	0.15	€ 238,845	€ 477,689	€ 716,534	€ 955,378	-€ 5,775,192	-€ 11,550,384	-€ 17,325,575	-€ 23,100,767
3457	0.23	€ 359,009	€ 718,018	##########	##########	-€ 8,680,729	-€ 17,361,457	-€ 26,042,186	-€ 34,722,915
3458	0.15	€ 232,696	€ 465,392	€ 698,087	€ 930,783	-€ 5,626,515	-€ 11,253,030	-€ 16,879,545	-€ 22,506,060
3459	0.23	€ 355,559	€ 711,117	##########	##########	-€ 8,597,305	-€ 17,194,609	-€ 25,791,914	-€ 34,389,219
3460	0.15	€ 235,130	€ 470,259	€ 705,389	€ 940,519	-€ 5,685,367	-€ 11,370,735	-€ 17,056,102	-€ 22,741,469
3461	0.16	€ 251,493	€ 502,985	€ 754,478	##########	-€ 6,081,019	-€ 12,162,037	-€ 18,243,056	-€ 24,324,074
3462	0.13	€ 209,952	€ 419,905	€ 629,857	€ 839,809	-€ 5,076,584	-€ 10,153,168	-€ 15,229,752	-€ 20,306,336
3463	0.23	€ 359,608	€ 719,216	##########	##########	-€ 8,695,209	-€ 17,390,419	-€ 26,085,628	-€ 34,780,838
3464	0.17	€ 268,878	€ 537,757	€ 806,635	##########	-€ 6,501,400	-€ 13,002,800	-€ 19,504,199	-€ 26,005,599

3465	0.17	€ 268,625	€ 537,249	€ 805,874	##########	-€ 6,495,263	-€ 12,990,525	-€ 19,485,788	-€ 25,981,050
3466	0.18	€ 277,044	€ 554,089	€ 831,133	##########	-€ 6,698,852	-€ 13,397,703	-€ 20,096,555	-€ 26,795,406
3467	0.21	€ 337,160	€ 674,320	##########	##########	-€ 8,152,428	-€ 16,304,856	-€ 24,457,284	-€ 32,609,713
3468	0.15	€ 242,813	€ 485,626	€ 728,439	€ 971,252	-€ 5,871,148	-€ 11,742,295	-€ 17,613,443	-€ 23,484,590
3469	0.19	€ 293,985	€ 587,969	€ 881,954	##########	-€ 7,108,464	-€ 14,216,928	-€ 21,325,392	-€ 28,433,856
3470	0.23	€ 364,844	€ 729,688	##########	##########	-€ 8,821,825	-€ 17,643,651	-€ 26,465,476	-€ 35,287,302
3471	0.17	€ 260,176	€ 520,352	€ 780,528	##########	-€ 6,290,980	-€ 12,581,961	-€ 18,872,941	-€ 25,163,922
3472	0.13	€ 204,015	€ 408,029	€ 612,044	€ 816,059	-€ 4,933,016	-€ 9,866,032	-€ 14,799,048	-€ 19,732,064
3473	0.23	€ 367,924	€ 735,848	##########	##########	-€ 8,896,291	-€ 17,792,581	-€ 26,688,872	-€ 35,585,162
3474	0.16	€ 256,585	€ 513,171	€ 769,756	##########	-€ 6,204,155	-€ 12,408,311	-€ 18,612,466	-€ 24,816,621
3475	0.17	€ 262,659	€ 525,318	€ 787,978	##########	-€ 6,351,022	-€ 12,702,045	-€ 19,053,067	-€ 25,404,090
3476	0.15	€ 232,037	€ 464,074	€ 696,112	€ 928,149	-€ 5,610,591	-€ 11,221,183	-€ 16,831,774	-€ 22,442,365
3477	0.14	€ 221,322	€ 442,645	€ 663,967	€ 885,290	-€ 5,351,510	-€ 10,703,020	-€ 16,054,531	-€ 21,406,041
3478	0.20	€ 312,541	€ 625,081	€ 937,622	##########	-€ 7,557,142	-€ 15,114,284	-€ 22,671,426	-€ 30,228,568
3479	0.26	€ 412,365	€ 824,731	##########	##########	-€ 9,970,874	-€ 19,941,748	-€ 29,912,622	-€ 39,883,496
3480	0.15	€ 240,683	€ 481,367	€ 722,050	€ 962,734	-€ 5,819,655	-€ 11,639,311	-€ 17,458,966	-€ 23,278,622
3481	0.14	€ 217,974	€ 435,947	€ 653,921	€ 871,895	-€ 5,270,539	-€ 10,541,078	-€ 15,811,617	-€ 21,082,156
3482	0.19	€ 303,356	€ 606,713	€ 910,069	##########	-€ 7,335,065	-€ 14,670,130	-€ 22,005,195	-€ 29,340,260
3483	0.18	€ 288,621	€ 577,241	€ 865,862	##########	-€ 6,978,760	-€ 13,957,520	-€ 20,936,279	-€ 27,915,039
3484	0.16	€ 249,818	€ 499,636	€ 749,454	€ 999,272	-€ 6,040,523	-€ 12,081,046	-€ 18,121,570	-€ 24,162,093
3485	0.23	€ 354,315	€ 708,630	##########	##########	-€ 8,567,230	-€ 17,134,459	-€ 25,701,689	-€ 34,268,919
3486	0.13	€ 207,233	€ 414,465	€ 621,698	€ 828,931	-€ 5,010,825	-€ 10,021,649	-€ 15,032,474	-€ 20,043,299
3487	0.25	€ 396,614	€ 793,228	##########	##########	-€ 9,590,010	-€ 19,180,020	-€ 28,770,030	-€ 38,360,040
3488	0.16	€ 249,221	€ 498,442	€ 747,664	€ 996,885	-€ 6,026,094	-€ 12,052,189	-€ 18,078,283	-€ 24,104,378
3489	0.14	€ 224,697	€ 449,394	€ 674,092	€ 898,789	-€ 5,433,112	-€ 10,866,224	-€ 16,299,335	-€ 21,732,447
3490	0.22	€ 346,361	€ 692,723	##########	##########	-€ 8,374,914	-€ 16,749,828	-€ 25,124,742	-€ 33,499,656
3491	0.19	€ 291,406	€ 582,812	€ 874,218	##########	-€ 7,046,111	-€ 14,092,222	-€ 21,138,333	-€ 28,184,444
3492	0.22	€ 347,806	€ 695,611	##########	##########	-€ 8,409,839	-€ 16,819,678	-€ 25,229,517	-€ 33,639,355
3493	0.14	€ 227,456	€ 454,912	€ 682,368	€ 909,824	-€ 5,499,820	-€ 10,999,639	-€ 16,499,459	-€ 21,999,278
3494	0.22	€ 347,277	€ 694,554	##########	##########	-€ 8,397,050	-€ 16,794,100	-€ 25,191,150	-€ 33,588,200
3495	0.19	€ 297,540	€ 595,080	€ 892,621	##########	-€ 7,194,434	-€ 14,388,869	-€ 21,583,303	-€ 28,777,737
3496	0.21	€ 326,803	€ 653,606	€ 980,409	##########	-€ 7,902,001	-€ 15,804,002	-€ 23,706,003	-€ 31,608,004
3497	0.23	€ 355,785	€ 711,570	##########	##########	-€ 8,602,771	-€ 17,205,542	-€ 25,808,313	-€ 34,411,085
3498	0.20	€ 321,323	€ 642,647	€ 963,970	##########	-€ 7,769,505	-€ 15,539,010	-€ 23,308,514	-€ 31,078,019
3499	0.15	€ 241,110	€ 482,220	€ 723,329	€ 964,439	-€ 5,829,964	-€ 11,659,928	-€ 17,489,892	-€ 23,319,856
3500	0.12	€ 190,397	€ 380,793	€ 571,190	€ 761,586	-€ 4,603,732	-€ 9,207,463	-€ 13,811,195	-€ 18,414,926
3501	0.15	€ 231,096	€ 462,192	€ 693,288	€ 924,385	-€ 5,587,837	-€ 11,175,673	-€ 16,763,510	-€ 22,351,346
3502	0.19	€ 301,524	€ 603,048	€ 904,572	##########	-€ 7,290,761	-€ 14,581,523	-€ 21,872,284	-€ 29,163,046
3503	0.18	€ 288,851	€ 577,702	€ 866,553	##########	-€ 6,984,333	-€ 13,968,666	-€ 20,952,999	-€ 27,937,332
3504	0.13	€ 204,860	€ 409,720	€ 614,580	€ 819,440	-€ 4,953,452	-€ 9,906,905	-€ 14,860,357	-€ 19,813,810
3505	0.16	€ 249,889	€ 499,778	€ 749,666	€ 999,555	-€ 6,042,236	-€ 12,084,472	-€ 18,126,708	-€ 24,168,944
3506	0.16	€ 255,463	€ 510,926	€ 766,388	##########	-€ 6,177,014	-€ 12,354,028	-€ 18,531,042	-€ 24,708,055
3507	0.20	€ 318,439	€ 636,878	€ 955,317	##########	-€ 7,699,757	-€ 15,399,515	-€ 23,099,272	-€ 30,799,030
3508	0.19	€ 297,173	€ 594,346	€ 891,519	##########	-€ 7,185,551	-€ 14,371,103	-€ 21,556,654	-€ 28,742,205
3509	0.18	€ 281,542	€ 563,083	€ 844,625	##########	-€ 6,807,594	-€ 13,615,188	-€ 20,422,783	-€ 27,230,377
3510	0.18	€ 279,598	€ 559,197	€ 838,795	##########	-€ 6,760,605	-€ 13,521,211	-€ 20,281,816	-€ 27,042,422
3511	0.19	€ 306,855	€ 613,710	€ 920,565	##########	-€ 7,419,662	-€ 14,839,324	-€ 22,258,987	-€ 29,678,649
3512	0.24	€ 380,614	€ 761,228	##########	##########	-€ 9,203,132	-€ 18,406,264	-€ 27,609,396	-€ 36,812,528
3513	0.26	€ 402,490	€ 804,981	##########	##########	-€ 9,732,099	-€ 19,464,197	-€ 29,196,296	-€ 38,928,394
3514	0.21	€ 332,873	€ 665,747	€ 998,620	##########	-€ 8,048,779	-€ 16,097,557	-€ 24,146,336	-€ 32,195,114
3515	0.16	€ 259,168	€ 518,336	€ 777,504	##########	-€ 6,266,609	-€ 12,533,219	-€ 18,799,828	-€ 25,066,437
3516	0.22	€ 347,886	€ 695,773	##########	##########	-€ 8,411,790	-€ 16,823,580	-€ 25,235,369	-€ 33,647,159
3517	0.22	€ 344,453	€ 688,907	##########	##########	-€ 8,328,781	-€ 16,657,562	-€ 24,986,343	-€ 33,315,124
3518	0.25	€ 394,270	€ 788,540	##########	##########	-€ 9,533,329	-€ 19,066,657	-€ 28,599,986	-€ 38,133,314
3519	0.12	€ 188,835	€ 377,671	€ 566,506	€ 755,342	-€ 4,565,986	-€ 9,131,972	-€ 13,697,957	-€ 18,263,943
3520	0.22	€ 340,340	€ 680,680	##########	##########	-€ 8,229,317	-€ 16,458,635	-€ 24,687,952	-€ 32,917,270
3521	0.14	€ 222,894	€ 445,787	€ 668,681	€ 891,575	-€ 5,389,502	-€ 10,779,004	-€ 16,168,506	-€ 21,558,008
3522	0.25	€ 399,875	€ 799,750	##########	##########	-€ 9,668,859	-€ 19,337,718	-€ 29,006,576	-€ 38,675,435
3523	0.28	€ 440,575	€ 881,150	##########	##########	-€ 10,652,978	-€ 21,305,957	-€ 31,958,935	-€ 42,611,913
3524	0.16	€ 244,375	€ 488,750	€ 733,124	€ 977,499	-€ 5,908,909	-€ 11,817,818	-€ 17,726,727	-€ 23,635,636
3525	0.20	€ 309,394	€ 618,787	€ 928,181	##########	-€ 7,481,045	-€ 14,962,091	-€ 22,443,136	-€ 29,924,181
3526	0.21	€ 331,660	€ 663,320	€ 994,980	##########	-€ 8,019,442	-€ 16,038,884	-€ 24,058,327	-€ 32,077,769
3527	0.16	€ 257,265	€ 514,531	€ 771,796	##########	-€ 6,220,599	-€ 12,441,199	-€ 18,661,798	-€ 24,882,397
3528	0.19	€ 292,645	€ 585,289	€ 877,934	##########	-€ 7,076,062	-€ 14,152,123	-€ 21,228,185	-€ 28,304,246
3529	0.17	€ 269,822	€ 539,645	€ 809,467	##########	-€ 6,524,224	-€ 13,048,447	-€ 19,572,671	-€ 26,096,895
3530	0.13	€ 205,418	€ 410,836	€ 616,254	€ 821,672	-€ 4,966,946	-€ 9,933,891	-€ 14,900,837	-€ 19,867,782
3531	0.15	€ 232,208	€ 464,416	€ 696,623	€ 928,831	-€ 5,614,715	-€ 11,229,431	-€ 16,844,146	-€ 22,458,862
3532	0.17	€ 271,036	€ 542,072	€ 813,108	##########	-€ 6,553,573	-€ 13,107,145	-€ 19,660,718	-€ 26,214,290
3533	0.19	€ 299,072	€ 598,145	€ 897,217	##########	-€ 7,231,479	-€ 14,462,958	-€ 21,694,437	-€ 28,925,917
3534	0.16	€ 248,299	€ 496,598	€ 744,897	€ 993,196	-€ 6,003,797	-€ 12,007,594	-€ 18,011,391	-€ 24,015,187
3535	0.20	€ 312,171	€ 624,343	€ 936,514	##########	-€ 7,548,212	-€ 15,096,423	-€ 22,644,635	-€ 30,192,847
3536	0.16	€ 254,303	€ 508,606	€ 762,909	##########	-€ 6,148,969	-€ 12,297,938	-€ 18,446,906	-€ 24,595,875
3537	0.17	€ 264,701	€ 529,401	€ 794,102	##########	-€ 6,400,381	-€ 12,800,762	-€ 19,201,143	-€ 25,601,524
3538	0.21	€ 330,726	€ 661,452	€ 992,177	##########	-€ 7,996,851	-€ 15,993,702	-€ 23,990,553	-€ 31,987,405
3539	0.18	€ 276,791	€ 553,581	€ 830,372	##########	-€ 6,692,715	-€ 13,385,431	-€ 20,078,146	-€ 26,770,861
3540	0.12	€ 192,358	€ 384,716	€ 577,073	€ 769,431	-€ 4,651,155	-€ 9,302,309	-€ 13,953,464	-€ 18,604,619
3541	0.21	€ 334,219	€ 668,439	##########	##########	-€ 8,081,327	-€ 16,162,653	-€ 24,243,980	-€ 32,325,306
3542	0.18	€ 275,808	€ 551,616	€ 827,424	##########	-€ 6,668,954	-€ 13,337,908	-€ 20,006,862	-€ 26,675,816
3543	0.15	€ 230,559	€ 461,118	€ 691,677	€ 922,236	-€ 5,574,850	-€ 11,149,700	-€ 16,724,551	-€ 22,299,401
3544	0.18	€ 290,045	€ 580,090	€ 870,135	##########	-€ 7,013,206	-€ 14,026,412	-€ 21,039,618	-€ 28,052,824
3545	0.20	€ 321,894	€ 643,788	€ 965,682	##########	-€ 7,783,302	-€ 15,566,605	-€ 23,349,907	-€ 31,133,209
3546	0.18	€ 288,021	€ 576,042	€ 864,063	##########	-€ 6,964,262	-€ 13,928,524	-€ 20,892,786	-€ 27,857,047
3547	0.18	€ 280,495	€ 560,989	€ 841,484	##########	-€ 6,782,275	-€ 13,564,550	-€ 20,346,826	-€ 27,129,101
3548	0.18	€ 286,456	€ 572,912	€ 859,368	##########	-€ 6,926,421	-€ 13,852,841	-€ 20,779,262	-€ 27,705,682
3549	0.19	€ 298,551	€ 597,102	€ 895,654	##########	-€ 7,218,880	-€ 14,437,760	-€ 21,656,640	-€ 28,875,520
3550	0.23	€ 369,529	€ 739,058	##########	##########	-€ 8,935,104	-€ 17,870,208	-€ 26,805,312	-€ 35,740,416
3551	0.17	€ 260,014	€ 520,028	€ 780,042	##########	-€ 6,287,064	-€ 12,574,128	-€ 18,861,193	-€ 25,148,257
3552	0.17	€ 262,853	€ 525,706	€ 788,559	##########	-€ 6,355,705	-€ 12,711,409	-€ 19,067,114	-€ 25,422,819
3553	0.17	€ 265,490	€ 530,979	€ 796,469	##########	-€ 6,419,458	-€ 12,838,916	-€ 19,258,374	-€ 25,677,832
3554	0.16	€ 251,881	€ 503,763	€ 755,644	##########	-€ 6,090,417	-€ 12,180,833	-€ 18,271,250	-€ 24,361,666
3555	0.19	€ 302,149	€ 604,299	€ 906,448	##########	-€ 7,305,884	-€ 14,611,767	-€ 21,917,651	-€ 29,223,534
3556	0.24	€ 379,062	€ 758,124	##########	##########	-€ 9,165,606	-€ 18,331,212	-€ 27,496,818	-€ 36,662,423
3557	0.18	€ 281,349	€ 562,698	€ 844,047	##########	-€ 6,802,939	-€ 13,605,878	-€ 20,408,818	-€ 27,211,757
3558	0.18	€ 285,211	€ 570,421	€ 855,632	##########	-€ 6,896,308	-€ 13,792,617	-€ 20,688,925	-€ 27,585,233
3559	0.31	€ 483,838	€ 967,676	##########	##########	-€ 11,699,060	-€ 23,398,120	-€ 35,097,179	-€ 46,796,239
3560	0.21	€ 322,834	€ 645,668	€ 968,503	##########	-€ 7,806,035	-€ 15,612,070	-€ 23,418,105	-€ 31,224,140
3561	0.19	€ 293,820	€ 587,640	€ 881,461	##########	-€ 7,104,485	-€ 14,208,971	-€ 21,313,456	-€ 28,417,941
3562	0.23	€ 364,827	€ 729,654	##########	##########	-€ 8,821,409	-€ 17,642,817	-€ 26,464,226	-€ 35,285,635
3563	0.22	€ 345,540	€ 691,080	##########	##########	-€ 8,355,054	-€ 16,710,107	-€ 25,065,161	-€ 33,420,215
3564	0.19	€ 301,847	€ 603,694	€ 905,541	##########	-€ 7,298,568	-€ 14,597,136	-€ 21,895,704	-€ 29,194,272
3565	0.24	€ 376,817	€ 753,634	##########	##########	-€ 9,111,328	-€ 18,222,656	-€ 27,333,985	-€ 36,445,313
3566	0.12	€ 185,981	€ 371,962	€ 557,943	€ 743,925	-€ 4,496,969	-€ 8,993,937	-€ 13,490,906	-€ 17,987,874

3567	0.21	€ 336,287	€ 672,573	##########	##########	-€ 8,131,308	-€ 16,262,616	-€ 24,393,924	-€ 32,525,232
3568	0.23	€ 356,645	€ 713,291	##########	##########	-€ 8,623,581	-€ 17,247,162	-€ 25,870,743	-€ 34,494,324
3569	0.12	€ 192,134	€ 384,268	€ 576,402	€ 768,536	-€ 4,645,742	-€ 9,291,484	-€ 13,937,225	-€ 18,582,967
3570	0.30	€ 465,836	€ 931,673	##########	##########	-€ 11,263,785	-€ 22,527,570	-€ 33,791,356	-€ 45,055,141
3571	0.16	€ 259,259	€ 518,518	€ 777,777	##########	-€ 6,268,809	-€ 12,537,619	-€ 18,806,428	-€ 25,075,237
3572	0.18	€ 277,678	€ 555,357	€ 833,035	##########	-€ 6,714,180	-€ 13,428,359	-€ 20,142,539	-€ 26,856,718
3573	0.23	€ 361,772	€ 723,544	##########	##########	-€ 8,747,538	-€ 17,495,076	-€ 26,242,614	-€ 34,990,152
3574	0.22	€ 341,435	€ 682,870	##########	##########	-€ 8,255,794	-€ 16,511,588	-€ 24,767,382	-€ 33,023,176
3575	0.22	€ 342,382	€ 684,763	##########	##########	-€ 8,278,687	-€ 16,557,374	-€ 24,836,060	-€ 33,114,747
3576	0.17	€ 265,672	€ 531,344	€ 797,017	##########	-€ 6,423,875	-€ 12,847,751	-€ 19,271,626	-€ 25,695,501
3577	0.17	€ 275,426	€ 550,853	€ 826,279	##########	-€ 6,659,728	-€ 13,319,456	-€ 19,979,183	-€ 26,638,911
3578	0.23	€ 359,565	€ 719,129	##########	##########	-€ 8,694,164	-€ 17,388,328	-€ 26,082,492	-€ 34,776,655
3579	0.20	€ 311,002	€ 622,005	€ 933,007	##########	-€ 7,519,946	-€ 15,039,892	-€ 22,559,838	-€ 30,079,784
3580	0.19	€ 293,644	€ 587,288	€ 880,932	##########	-€ 7,100,227	-€ 14,200,453	-€ 21,300,680	-€ 28,400,906
3581	0.16	€ 248,458	€ 496,916	€ 745,375	€ 993,833	-€ 6,007,645	-€ 12,015,290	-€ 18,022,935	-€ 24,030,579
3582	0.19	€ 297,837	€ 595,675	€ 893,512	##########	-€ 7,201,621	-€ 14,403,242	-€ 21,604,863	-€ 28,806,484
3583	0.20	€ 311,510	€ 623,021	€ 934,531	##########	-€ 7,532,228	-€ 15,064,456	-€ 22,596,684	-€ 30,128,911
3584	0.12	€ 194,241	€ 388,482	€ 582,723	€ 776,964	-€ 4,696,690	-€ 9,393,380	-€ 14,090,070	-€ 18,786,759
3585	0.29	€ 457,793	€ 915,587	##########	##########	-€ 11,069,309	-€ 22,138,618	-€ 33,207,927	-€ 44,277,236
3586	0.27	€ 417,775	€ 835,551	##########	##########	-€ 10,101,684	-€ 20,203,367	-€ 30,305,051	-€ 40,406,734
3587	0.17	€ 260,348	€ 520,696	€ 781,044	##########	-€ 6,295,134	-€ 12,590,268	-€ 18,885,403	-€ 25,180,537
3588	0.19	€ 295,143	€ 590,285	€ 885,428	##########	-€ 7,136,460	-€ 14,272,920	-€ 21,409,381	-€ 28,545,841
3589	0.24	€ 371,787	€ 743,574	##########	##########	-€ 8,989,698	-€ 17,979,396	-€ 26,969,094	-€ 35,958,792
3590	0.21	€ 332,802	€ 665,603	€ 998,405	##########	-€ 8,047,047	-€ 16,094,094	-€ 24,141,141	-€ 32,188,188
3591	0.15	€ 229,099	€ 458,197	€ 687,296	€ 916,394	-€ 5,539,534	-€ 11,079,068	-€ 16,618,602	-€ 22,158,136
3592	0.14	€ 226,140	€ 452,280	€ 678,421	€ 904,561	-€ 5,468,003	-€ 10,936,007	-€ 16,404,010	-€ 21,872,013
3593	0.25	€ 386,663	€ 773,325	##########	##########	-€ 9,349,389	-€ 18,698,779	-€ 28,048,168	-€ 37,397,558
3594	0.18	€ 277,712	€ 555,423	€ 833,135	##########	-€ 6,714,987	-€ 13,429,974	-€ 20,144,961	-€ 26,859,949
3595	0.16	€ 253,126	€ 506,252	€ 759,378	##########	-€ 6,120,513	-€ 12,241,026	-€ 18,361,539	-€ 24,482,052
3596	0.15	€ 236,609	€ 473,218	€ 709,826	€ 946,435	-€ 5,721,129	-€ 11,442,259	-€ 17,163,388	-€ 22,884,518
3597	0.12	€ 191,114	€ 382,229	€ 573,343	€ 764,458	-€ 4,621,090	-€ 9,242,181	-€ 13,863,271	-€ 18,484,361
3598	0.14	€ 213,340	€ 426,680	€ 640,020	€ 853,359	-€ 5,158,495	-€ 10,316,990	-€ 15,475,484	-€ 20,633,979
3599	0.22	€ 352,188	€ 704,375	##########	##########	-€ 8,515,795	-€ 17,031,590	-€ 25,547,385	-€ 34,063,180
3600	0.19	€ 294,698	€ 589,396	€ 884,094	##########	-€ 7,125,713	-€ 14,251,426	-€ 21,377,138	-€ 28,502,851
3601	0.20	€ 320,814	€ 641,627	€ 962,441	##########	-€ 7,757,177	-€ 15,514,355	-€ 23,271,532	-€ 31,028,710
3602	0.18	€ 290,025	€ 580,050	€ 870,076	##########	-€ 7,012,724	-€ 14,025,448	-€ 21,038,172	-€ 28,050,896
3603	0.16	€ 251,246	€ 502,491	€ 753,737	##########	-€ 6,075,045	-€ 12,150,091	-€ 18,225,136	-€ 24,300,182
3604	0.25	€ 393,864	€ 787,729	##########	##########	-€ 9,523,522	-€ 19,047,045	-€ 28,570,567	-€ 38,094,089
3605	0.17	€ 272,184	€ 544,368	€ 816,552	##########	-€ 6,581,330	-€ 13,162,661	-€ 19,743,991	-€ 26,325,321
3606	0.10	€ 163,758	€ 327,516	€ 491,273	€ 655,031	-€ 3,959,614	-€ 7,919,228	-€ 11,878,842	-€ 15,838,457
3607	0.19	€ 300,272	€ 600,544	€ 900,815	##########	-€ 7,260,483	-€ 14,520,966	-€ 21,781,449	-€ 29,041,931
3608	0.18	€ 281,941	€ 563,882	€ 845,823	##########	-€ 6,817,253	-€ 13,634,507	-€ 20,451,760	-€ 27,269,013
3609	0.22	€ 352,434	€ 704,868	##########	##########	-€ 8,521,747	-€ 17,043,494	-€ 25,565,240	-€ 34,086,987
3610	0.19	€ 291,305	€ 582,609	€ 873,914	##########	-€ 7,043,658	-€ 14,087,315	-€ 21,130,973	-€ 28,174,631
3611	0.20	€ 307,750	€ 615,501	€ 923,251	##########	-€ 7,441,315	-€ 14,882,631	-€ 22,323,946	-€ 29,765,261
3612	0.18	€ 285,254	€ 570,508	€ 855,762	##########	-€ 6,897,360	-€ 13,794,720	-€ 20,692,080	-€ 27,589,439
3613	0.14	€ 219,076	€ 438,151	€ 657,227	€ 876,303	-€ 5,297,186	-€ 10,594,372	-€ 15,891,558	-€ 21,188,744
3614	0.23	€ 361,381	€ 722,761	##########	##########	-€ 8,738,074	-€ 17,476,148	-€ 26,214,223	-€ 34,952,297
3615	0.18	€ 284,660	€ 569,319	€ 853,979	##########	-€ 6,882,984	-€ 13,765,967	-€ 20,648,951	-€ 27,531,935
3616	0.14	€ 227,189	€ 454,379	€ 681,568	€ 908,757	-€ 5,493,370	-€ 10,986,741	-€ 16,480,111	-€ 21,973,482
3617	0.14	€ 220,212	€ 440,424	€ 660,636	€ 880,849	-€ 5,324,665	-€ 10,649,329	-€ 15,973,994	-€ 21,298,659
3618	0.19	€ 302,075	€ 604,151	€ 906,226	##########	-€ 7,304,091	-€ 14,608,183	-€ 21,912,274	-€ 29,216,365
3619	0.14	€ 223,898	€ 447,796	€ 671,695	€ 895,593	-€ 5,413,792	-€ 10,827,584	-€ 16,241,377	-€ 21,655,169
3620	0.19	€ 305,946	€ 611,892	€ 917,837	##########	-€ 7,397,679	-€ 14,795,358	-€ 22,193,037	-€ 29,590,716
3621	0.19	€ 299,971	€ 599,943	€ 899,914	##########	-€ 7,253,220	-€ 14,506,439	-€ 21,759,659	-€ 29,012,878
3622	0.14	€ 216,696	€ 433,392	€ 650,089	€ 866,785	-€ 5,239,650	-€ 10,479,299	-€ 15,718,949	-€ 20,958,599
3623	0.13	€ 210,661	€ 421,322	€ 631,982	€ 842,643	-€ 5,093,716	-€ 10,187,432	-€ 15,281,147	-€ 20,374,863
3624	0.19	€ 306,724	€ 613,448	€ 920,173	##########	-€ 7,416,501	-€ 14,833,002	-€ 22,249,503	-€ 29,666,005
3625	0.24	€ 382,398	€ 764,795	##########	##########	-€ 9,246,259	-€ 18,492,518	-€ 27,738,778	-€ 36,985,037
3626	0.18	€ 277,376	€ 554,752	€ 832,128	##########	-€ 6,706,866	-€ 13,413,731	-€ 20,120,597	-€ 26,827,462
3627	0.23	€ 363,441	€ 726,882	##########	##########	-€ 8,787,892	-€ 17,575,783	-€ 26,363,675	-€ 35,151,567
3628	0.20	€ 319,533	€ 639,066	€ 958,599	##########	-€ 7,726,214	-€ 15,452,427	-€ 23,178,641	-€ 30,904,854
3629	0.14	€ 214,590	€ 429,180	€ 643,769	€ 858,359	-€ 5,188,717	-€ 10,377,434	-€ 15,566,151	-€ 20,754,868
3630	0.15	€ 240,833	€ 481,666	€ 722,499	€ 963,332	-€ 5,823,272	-€ 11,646,544	-€ 17,469,817	-€ 23,293,089
3631	0.23	€ 366,520	€ 733,040	##########	##########	-€ 8,862,341	-€ 17,724,683	-€ 26,587,024	-€ 35,449,365
3632	0.22	€ 347,929	€ 695,858	##########	##########	-€ 8,412,820	-€ 16,825,641	-€ 25,238,461	-€ 33,651,282
3633	0.19	€ 292,727	€ 585,454	€ 878,181	##########	-€ 7,078,052	-€ 14,156,104	-€ 21,234,156	-€ 28,312,208
3634	0.18	€ 280,735	€ 561,470	€ 842,205	##########	-€ 6,788,092	-€ 13,576,185	-€ 20,364,277	-€ 27,152,370
3635	0.22	€ 345,763	€ 691,527	##########	##########	-€ 8,360,458	-€ 16,720,916	-€ 25,081,375	-€ 33,441,833
3636	0.19	€ 300,331	€ 600,662	€ 900,993	##########	-€ 7,261,916	-€ 14,523,832	-€ 21,785,748	-€ 29,047,665
3637	0.18	€ 288,855	€ 577,710	€ 866,565	##########	-€ 6,984,425	-€ 13,968,849	-€ 20,953,274	-€ 27,937,698
3638	0.23	€ 368,717	€ 737,433	##########	##########	-€ 8,915,459	-€ 17,830,918	-€ 26,746,377	-€ 35,661,836
3639	0.14	€ 213,572	€ 427,145	€ 640,717	€ 854,289	-€ 5,164,116	-€ 10,328,232	-€ 15,492,348	-€ 20,656,464
3640	0.19	€ 304,582	€ 609,164	€ 913,746	##########	-€ 7,364,705	-€ 14,729,411	-€ 22,094,116	-€ 29,458,822
3641	0.13	€ 209,445	€ 418,889	€ 628,334	€ 837,778	-€ 5,064,307	-€ 10,128,613	-€ 15,192,920	-€ 20,257,227
3642	0.17	€ 273,984	€ 547,968	€ 821,952	##########	-€ 6,624,850	-€ 13,249,701	-€ 19,874,551	-€ 26,499,401
3643	0.21	€ 330,871	€ 661,743	€ 992,614	##########	-€ 8,000,372	-€ 16,000,744	-€ 24,001,115	-€ 32,001,487
3644	0.15	€ 235,837	€ 471,675	€ 707,512	€ 943,350	-€ 5,702,479	-€ 11,404,958	-€ 17,107,437	-€ 22,809,916
3645	0.20	€ 310,683	€ 621,366	€ 932,048	##########	-€ 7,512,218	-€ 15,024,436	-€ 22,536,654	-€ 30,048,871
3646	0.14	€ 220,629	€ 441,257	€ 661,886	€ 882,515	-€ 5,334,737	-€ 10,669,474	-€ 16,004,210	-€ 21,338,947
3647	0.24	€ 375,591	€ 751,182	##########	##########	-€ 9,081,677	-€ 18,163,354	-€ 27,245,032	-€ 36,326,709
3648	0.13	€ 208,279	€ 416,557	€ 624,836	€ 833,114	-€ 5,036,114	-€ 10,072,228	-€ 15,108,342	-€ 20,144,456
3649	0.19	€ 295,147	€ 590,294	€ 885,441	##########	-€ 7,136,564	-€ 14,273,127	-€ 21,409,691	-€ 28,546,255
3650	0.16	€ 248,042	€ 496,084	€ 744,126	€ 992,168	-€ 5,997,584	-€ 11,995,169	-€ 17,992,753	-€ 23,990,337
3651	0.16	€ 252,186	€ 504,372	€ 756,558	##########	-€ 6,097,779	-€ 12,195,558	-€ 18,293,337	-€ 24,391,116
3652	0.20	€ 321,868	€ 643,737	€ 965,605	##########	-€ 7,782,681	-€ 15,565,363	-€ 23,348,044	-€ 31,130,725
3653	0.21	€ 326,434	€ 652,868	€ 979,302	##########	-€ 7,893,077	-€ 15,786,153	-€ 23,679,230	-€ 31,572,306
3654	0.15	€ 238,650	€ 477,299	€ 715,949	€ 954,599	-€ 5,770,480	-€ 11,540,960	-€ 17,311,440	-€ 23,081,920
3655	0.20	€ 319,894	€ 639,788	€ 959,682	##########	-€ 7,734,938	-€ 15,469,877	-€ 23,204,815	-€ 30,939,753
3656	0.15	€ 228,428	€ 456,856	€ 685,284	€ 913,712	-€ 5,523,320	-€ 11,046,641	-€ 16,569,961	-€ 22,093,281
3657	0.18	€ 278,164	€ 556,329	€ 834,493	##########	-€ 6,725,932	-€ 13,451,865	-€ 20,177,797	-€ 26,903,729
3658	0.14	€ 218,829	€ 437,659	€ 656,488	€ 875,318	-€ 5,291,231	-€ 10,582,463	-€ 15,873,694	-€ 21,164,926
3659	0.17	€ 269,254	€ 538,508	€ 807,763	##########	-€ 6,510,487	-€ 13,020,973	-€ 19,531,460	-€ 26,041,947
3660	0.22	€ 345,297	€ 690,594	##########	##########	-€ 8,349,181	-€ 16,698,363	-€ 25,047,544	-€ 33,396,725
3661	0.18	€ 284,875	€ 569,750	€ 854,625	##########	-€ 6,888,192	-€ 13,776,383	-€ 20,664,575	-€ 27,552,767
3662	0.18	€ 290,248	€ 580,497	€ 870,745	##########	-€ 7,018,118	-€ 14,036,237	-€ 21,054,355	-€ 28,072,473
3663	0.18	€ 278,009	€ 556,018	€ 834,027	##########	-€ 6,722,173	-€ 13,444,345	-€ 20,166,518	-€ 26,888,690
3664	0.15	€ 231,663	€ 463,326	€ 694,990	€ 926,653	-€ 5,601,549	-€ 11,203,097	-€ 16,804,646	-€ 22,406,194
3665	0.15	€ 238,555	€ 477,110	€ 715,665	€ 954,220	-€ 5,768,189	-€ 11,536,379	-€ 17,304,568	-€ 23,072,758
3666	0.18	€ 288,063	€ 576,126	€ 864,189	##########	-€ 6,965,282	-€ 13,930,564	-€ 20,895,845	-€ 27,861,127
3667	0.24	€ 381,618	€ 763,236	##########	##########	-€ 9,227,409	-€ 18,454,818	-€ 27,682,227	-€ 36,909,636
3668	0.15	€ 235,321	€ 470,642	€ 705,963	€ 941,284	-€ 5,689,993	-€ 11,379,987	-€ 17,069,980	-€ 22,759,973

3669	0.21	€ 328,081	€ 656,161	€ 984,242	##########	-€ 7,932,895	-€ 15,865,789	-€ 23,798,684	-€ 31,731,578
3670	0.20	€ 317,871	€ 635,741	€ 953,612	##########	-€ 7,686,019	-€ 15,372,039	-€ 23,058,058	-€ 30,744,077
3671	0.17	€ 261,569	€ 523,138	€ 784,707	##########	-€ 6,324,659	-€ 12,649,317	-€ 18,973,976	-€ 25,298,635
3672	0.17	€ 267,269	€ 534,537	€ 801,806	##########	-€ 6,462,477	-€ 12,924,954	-€ 19,387,431	-€ 25,849,908
3673	0.15	€ 230,316	€ 460,633	€ 690,949	€ 921,266	-€ 5,568,983	-€ 11,137,966	-€ 16,706,949	-€ 22,275,932
3674	0.22	€ 349,486	€ 698,973	##########	##########	-€ 8,450,474	-€ 16,900,948	-€ 25,351,422	-€ 33,801,896
3675	0.15	€ 237,512	€ 475,024	€ 712,536	€ 950,048	-€ 5,742,970	-€ 11,485,940	-€ 17,228,910	-€ 22,971,880
3676	0.20	€ 318,667	€ 637,333	€ 956,000	##########	-€ 7,705,262	-€ 15,410,524	-€ 23,115,787	-€ 30,821,049
3677	0.11	€ 177,608	€ 355,217	€ 532,825	€ 710,433	-€ 4,294,516	-€ 8,589,032	-€ 12,883,547	-€ 17,178,063
3678	0.20	€ 307,348	€ 614,696	€ 922,044	##########	-€ 7,431,580	-€ 14,863,161	-€ 22,294,741	-€ 29,726,321
3679	0.12	€ 182,238	€ 364,476	€ 546,714	€ 728,951	-€ 4,406,457	-€ 8,812,914	-€ 13,219,371	-€ 17,625,828
3680	0.15	€ 238,622	€ 477,245	€ 715,867	€ 954,489	-€ 5,769,817	-€ 11,539,634	-€ 17,309,451	-€ 23,079,268
3681	0.18	€ 279,350	€ 558,701	€ 838,051	##########	-€ 6,754,607	-€ 13,509,214	-€ 20,263,821	-€ 27,018,428
3682	0.14	€ 215,852	€ 431,703	€ 647,555	€ 863,406	-€ 5,219,226	-€ 10,438,452	-€ 15,657,678	-€ 20,876,904
3683	0.21	€ 336,371	€ 672,741	##########	##########	-€ 8,133,345	-€ 16,266,689	-€ 24,400,034	-€ 32,533,378
3684	0.20	€ 319,990	€ 639,980	€ 959,969	##########	-€ 7,737,258	-€ 15,474,516	-€ 23,211,775	-€ 30,949,033
3685	0.21	€ 336,511	€ 673,022	##########	##########	-€ 8,136,731	-€ 16,273,463	-€ 24,410,194	-€ 32,546,926
3686	0.16	€ 256,141	€ 512,282	€ 768,423	##########	-€ 6,193,412	-€ 12,386,823	-€ 18,580,235	-€ 24,773,646
3687	0.19	€ 292,382	€ 584,764	€ 877,147	##########	-€ 7,069,715	-€ 14,139,430	-€ 21,209,145	-€ 28,278,861
3688	0.18	€ 285,541	€ 571,082	€ 856,623	##########	-€ 6,904,297	-€ 13,808,594	-€ 20,712,892	-€ 27,617,189
3689	0.21	€ 323,289	€ 646,577	€ 969,866	##########	-€ 7,817,020	-€ 15,634,041	-€ 23,451,061	-€ 31,268,081
3690	0.27	€ 430,309	€ 860,618	##########	##########	-€ 10,404,745	-€ 20,809,490	-€ 31,214,236	-€ 41,618,981
3691	0.19	€ 305,662	€ 611,325	€ 916,987	##########	-€ 7,390,826	-€ 14,781,653	-€ 22,172,479	-€ 29,563,306
3692	0.22	€ 351,287	€ 702,575	##########	##########	-€ 8,494,023	-€ 16,988,046	-€ 25,482,069	-€ 33,976,092
3693	0.16	€ 244,283	€ 488,565	€ 732,848	€ 977,131	-€ 5,906,683	-€ 11,813,367	-€ 17,720,050	-€ 23,626,733
3694	0.24	€ 384,320	€ 768,641	##########	##########	-€ 9,292,753	-€ 18,585,506	-€ 27,878,259	-€ 37,171,012
3695	0.16	€ 248,578	€ 497,157	€ 745,735	€ 994,314	-€ 6,010,554	-€ 12,021,109	-€ 18,031,663	-€ 24,042,217
3696	0.16	€ 253,560	€ 507,121	€ 760,681	##########	-€ 6,131,014	-€ 12,262,028	-€ 18,393,042	-€ 24,524,057
3697	0.21	€ 332,938	€ 665,877	€ 998,815	##########	-€ 8,050,351	-€ 16,100,702	-€ 24,151,053	-€ 32,201,404
3698	0.19	€ 296,152	€ 592,304	€ 888,456	##########	-€ 7,160,865	-€ 14,321,730	-€ 21,482,595	-€ 28,643,460
3699	0.15	€ 238,913	€ 477,826	€ 716,740	€ 955,653	-€ 5,776,851	-€ 11,553,702	-€ 17,330,553	-€ 23,107,404
3700	0.22	€ 352,734	€ 705,467	##########	##########	-€ 8,528,993	-€ 17,057,985	-€ 25,586,978	-€ 34,115,971
3701	0.23	€ 359,729	€ 719,457	##########	##########	-€ 8,698,131	-€ 17,396,261	-€ 26,094,392	-€ 34,792,522
3702	0.24	€ 379,982	€ 759,965	##########	##########	-€ 9,187,860	-€ 18,375,720	-€ 27,563,579	-€ 36,751,439
3703	0.15	€ 239,774	€ 479,548	€ 719,323	€ 959,097	-€ 5,797,670	-€ 11,595,340	-€ 17,393,010	-€ 23,190,680
3704	0.19	€ 299,655	€ 599,310	€ 898,965	##########	-€ 7,245,567	-€ 14,491,135	-€ 21,736,702	-€ 28,982,270
3705	0.17	€ 266,706	€ 533,412	€ 800,118	##########	-€ 6,448,874	-€ 12,897,748	-€ 19,346,622	-€ 25,795,496
3706	0.20	€ 319,526	€ 639,051	€ 958,577	##########	-€ 7,726,034	-€ 15,452,067	-€ 23,178,101	-€ 30,904,135
3707	0.21	€ 332,472	€ 664,944	€ 997,415	##########	-€ 8,039,069	-€ 16,078,137	-€ 24,117,206	-€ 32,156,275
3708	0.17	€ 267,037	€ 534,073	€ 801,110	##########	-€ 6,456,867	-€ 12,913,735	-€ 19,370,602	-€ 25,827,469
3709	0.17	€ 259,999	€ 519,998	€ 779,996	##########	-€ 6,286,693	-€ 12,573,387	-€ 18,860,080	-€ 25,146,773
3710	0.20	€ 322,359	€ 644,719	€ 967,078	##########	-€ 7,794,556	-€ 15,589,112	-€ 23,383,668	-€ 31,178,224
3711	0.16	€ 254,651	€ 509,301	€ 763,952	##########	-€ 6,157,375	-€ 12,314,750	-€ 18,472,125	-€ 24,629,499
3712	0.18	€ 276,093	€ 552,186	€ 828,279	##########	-€ 6,675,849	-€ 13,351,698	-€ 20,027,547	-€ 26,703,396
3713	0.19	€ 294,810	€ 589,620	€ 884,429	##########	-€ 7,128,413	-€ 14,256,826	-€ 21,385,239	-€ 28,513,652
3714	0.23	€ 367,395	€ 734,789	##########	##########	-€ 8,883,494	-€ 17,766,989	-€ 26,650,483	-€ 35,533,978
3715	0.16	€ 252,704	€ 505,408	€ 758,113	##########	-€ 6,110,312	-€ 12,220,625	-€ 18,330,937	-€ 24,441,250
3716	0.16	€ 258,581	€ 517,162	€ 775,743	##########	-€ 6,252,411	-€ 12,504,822	-€ 18,757,232	-€ 25,009,643
3717	0.19	€ 304,221	€ 608,442	€ 912,663	##########	-€ 7,355,970	-€ 14,711,940	-€ 22,067,911	-€ 29,423,881
3718	0.17	€ 265,079	€ 530,157	€ 795,236	##########	-€ 6,409,519	-€ 12,819,039	-€ 19,228,558	-€ 25,638,078
3719	0.21	€ 327,639	€ 655,277	€ 982,916	##########	-€ 7,922,207	-€ 15,844,415	-€ 23,766,622	-€ 31,688,830
3720	0.21	€ 323,107	€ 646,214	€ 969,321	##########	-€ 7,812,635	-€ 15,625,269	-€ 23,437,904	-€ 31,250,538
3721	0.28	€ 437,473	€ 874,947	##########	##########	-€ 10,577,976	-€ 21,155,951	-€ 31,733,927	-€ 42,311,903
3722	0.25	€ 392,693	€ 785,386	##########	##########	-€ 9,495,195	-€ 18,990,389	-€ 28,485,584	-€ 37,980,779
3723	0.18	€ 276,937	€ 553,874	€ 830,810	##########	-€ 6,696,249	-€ 13,392,498	-€ 20,088,748	-€ 26,784,997
3724	0.24	€ 377,957	€ 755,913	##########	##########	-€ 9,138,878	-€ 18,277,757	-€ 27,416,635	-€ 36,555,514
3725	0.23	€ 363,803	€ 727,606	##########	##########	-€ 8,796,652	-€ 17,593,303	-€ 26,389,955	-€ 35,186,606
3726	0.17	€ 274,118	€ 548,236	€ 822,354	##########	-€ 6,628,093	-€ 13,256,186	-€ 19,884,279	-€ 26,512,372
3727	0.18	€ 280,974	€ 561,948	€ 842,923	##########	-€ 6,793,872	-€ 13,587,745	-€ 20,381,617	-€ 27,175,489
3728	0.20	€ 320,131	€ 640,262	€ 960,393	##########	-€ 7,740,673	-€ 15,481,346	-€ 23,222,019	-€ 30,962,692
3729	0.19	€ 292,953	€ 585,907	€ 878,860	##########	-€ 7,083,523	-€ 14,167,047	-€ 21,250,570	-€ 28,334,093
3730	0.19	€ 293,585	€ 587,169	€ 880,754	##########	-€ 7,098,789	-€ 14,197,579	-€ 21,296,368	-€ 28,395,158
3731	0.15	€ 242,377	€ 484,753	€ 727,130	€ 969,506	-€ 5,860,593	-€ 11,721,185	-€ 17,581,778	-€ 23,442,370
3732	0.18	€ 289,354	€ 578,709	€ 868,063	##########	-€ 6,996,506	-€ 13,993,011	-€ 20,989,517	-€ 27,986,023
3733	0.18	€ 288,314	€ 576,628	€ 864,942	##########	-€ 6,971,344	-€ 13,942,688	-€ 20,914,032	-€ 27,885,376
3734	0.15	€ 236,321	€ 472,642	€ 708,964	€ 945,285	-€ 5,714,177	-€ 11,428,354	-€ 17,142,531	-€ 22,856,708
3735	0.19	€ 305,103	€ 610,206	€ 915,308	##########	-€ 7,377,295	-€ 14,754,591	-€ 22,131,886	-€ 29,509,181
3736	0.20	€ 319,693	€ 639,387	€ 959,080	##########	-€ 7,730,092	-€ 15,460,185	-€ 23,190,277	-€ 30,920,369
3737	0.16	€ 250,895	€ 501,791	€ 752,686	##########	-€ 6,066,574	-€ 12,133,148	-€ 18,199,722	-€ 24,266,296
3738	0.17	€ 263,559	€ 527,117	€ 790,676	##########	-€ 6,372,771	-€ 12,745,542	-€ 19,118,313	-€ 25,491,084
3739	0.27	€ 419,997	€ 839,993	##########	##########	-€ 10,155,393	-€ 20,310,787	-€ 30,466,180	-€ 40,621,574
3740	0.19	€ 306,004	€ 612,008	€ 918,012	##########	-€ 7,399,084	-€ 14,798,168	-€ 22,197,252	-€ 29,596,335
3741	0.26	€ 413,672	€ 827,343	##########	##########	-€ 10,002,457	-€ 20,004,913	-€ 30,007,370	-€ 40,009,827
3742	0.19	€ 296,161	€ 592,321	€ 888,482	##########	-€ 7,161,077	-€ 14,322,154	-€ 21,483,231	-€ 28,644,308
3743	0.23	€ 366,830	€ 733,660	##########	##########	-€ 8,869,838	-€ 17,739,677	-€ 26,609,515	-€ 35,479,354
3744	0.21	€ 328,784	€ 657,567	€ 986,351	##########	-€ 7,949,891	-€ 15,899,782	-€ 23,849,673	-€ 31,799,565
3745	0.24	€ 372,217	€ 744,434	##########	##########	-€ 9,000,092	-€ 18,000,184	-€ 27,000,275	-€ 36,000,367
3746	0.20	€ 307,278	€ 614,556	€ 921,834	##########	-€ 7,429,890	-€ 14,859,780	-€ 22,289,670	-€ 29,719,560
3747	0.24	€ 376,347	€ 752,695	##########	##########	-€ 9,099,970	-€ 18,199,939	-€ 27,299,909	-€ 36,399,878
3748	0.14	€ 220,464	€ 440,928	€ 661,392	€ 881,855	-€ 5,330,751	-€ 10,661,502	-€ 15,992,253	-€ 21,323,003
3749	0.19	€ 302,380	€ 604,760	€ 907,140	##########	-€ 7,311,461	-€ 14,622,922	-€ 21,934,382	-€ 29,245,843
3750	0.18	€ 278,966	€ 557,933	€ 836,899	##########	-€ 6,745,326	-€ 13,490,652	-€ 20,235,978	-€ 26,981,304
3751	0.15	€ 230,935	€ 461,870	€ 692,806	€ 923,741	-€ 5,583,945	-€ 11,167,890	-€ 16,751,835	-€ 22,335,781
3752	0.14	€ 217,496	€ 434,993	€ 652,489	€ 869,985	-€ 5,258,996	-€ 10,517,993	-€ 15,776,989	-€ 21,035,985
3753	0.15	€ 230,970	€ 461,941	€ 692,911	€ 923,882	-€ 5,584,796	-€ 11,169,591	-€ 16,754,387	-€ 22,339,183
3754	0.19	€ 306,933	€ 613,866	€ 920,799	##########	-€ 7,421,547	-€ 14,843,095	-€ 22,264,642	-€ 29,686,190
3755	0.16	€ 257,634	€ 515,268	€ 772,902	##########	-€ 6,229,511	-€ 12,459,022	-€ 18,688,533	-€ 24,918,044
3756	0.17	€ 260,086	€ 520,172	€ 780,258	##########	-€ 6,288,803	-€ 12,577,607	-€ 18,866,410	-€ 25,155,214
3757	0.16	€ 255,111	€ 510,222	€ 765,333	##########	-€ 6,168,507	-€ 12,337,013	-€ 18,505,520	-€ 24,674,026
3758	0.15	€ 242,393	€ 484,785	€ 727,178	€ 969,571	-€ 5,860,984	-€ 11,721,967	-€ 17,582,951	-€ 23,443,935
3759	0.23	€ 365,785	€ 731,570	##########	##########	-€ 8,844,573	-€ 17,689,145	-€ 26,533,718	-€ 35,378,291
3760	0.24	€ 380,007	€ 760,015	##########	##########	-€ 9,188,466	-€ 18,376,932	-€ 27,565,399	-€ 36,753,865
3761	0.26	€ 409,125	€ 818,251	##########	##########	-€ 9,892,532	-€ 19,785,065	-€ 29,677,597	-€ 39,570,130
3762	0.15	€ 234,587	€ 469,174	€ 703,762	€ 938,349	-€ 5,672,249	-€ 11,344,498	-€ 17,016,747	-€ 22,688,995
3763	0.16	€ 251,571	€ 503,141	€ 754,712	##########	-€ 6,082,905	-€ 12,165,810	-€ 18,248,714	-€ 24,331,619
3764	0.15	€ 235,790	€ 471,580	€ 707,370	€ 943,160	-€ 5,701,332	-€ 11,402,665	-€ 17,103,997	-€ 22,805,329
3765	0.21	€ 327,374	€ 654,748	€ 982,122	##########	-€ 7,915,808	-€ 15,831,616	-€ 23,747,424	-€ 31,663,232
3766	0.20	€ 317,718	€ 635,436	€ 953,154	##########	-€ 7,682,328	-€ 15,364,656	-€ 23,046,984	-€ 30,729,312
3767	0.21	€ 329,556	€ 659,113	€ 988,669	##########	-€ 7,968,574	-€ 15,937,148	-€ 23,905,722	-€ 31,874,296
3768	0.21	€ 334,253	€ 668,505	##########	##########	-€ 8,082,128	-€ 16,164,257	-€ 24,246,385	-€ 32,328,514
3769	0.16	€ 250,610	€ 501,221	€ 751,831	##########	-€ 6,059,684	-€ 12,119,368	-€ 18,179,051	-€ 24,238,735
3770	0.17	€ 271,748	€ 543,496	€ 815,244	##########	-€ 6,570,787	-€ 13,141,574	-€ 19,712,362	-€ 26,283,149

3771	0.20	€ 318,108	€ 636,217	€ 954,325	##########	-€ 7,691,763	-€ 15,383,526	-€ 23,075,289	-€ 30,767,053
3772	0.17	€ 267,949	€ 535,898	€ 803,847	##########	-€ 6,478,929	-€ 12,957,859	-€ 19,436,788	-€ 25,915,718
3773	0.17	€ 267,525	€ 535,049	€ 802,574	##########	-€ 6,468,667	-€ 12,937,333	-€ 19,406,000	-€ 25,874,666
3774	0.15	€ 237,776	€ 475,553	€ 713,329	€ 951,105	-€ 5,749,361	-€ 11,498,723	-€ 17,248,084	-€ 22,997,445
3775	0.18	€ 284,845	€ 569,691	€ 854,536	##########	-€ 6,887,478	-€ 13,774,955	-€ 20,662,433	-€ 27,549,911
3776	0.21	€ 323,282	€ 646,565	€ 969,847	##########	-€ 7,816,872	-€ 15,633,743	-€ 23,450,615	-€ 31,267,486
3777	0.18	€ 277,683	€ 555,366	€ 833,049	##########	-€ 6,714,289	-€ 13,428,579	-€ 20,142,868	-€ 26,857,158
3778	0.28	€ 434,779	€ 869,558	##########	##########	-€ 10,512,831	-€ 21,025,662	-€ 31,538,494	-€ 42,051,325
3779	0.22	€ 352,978	€ 705,957	##########	##########	-€ 8,534,910	-€ 17,069,819	-€ 25,604,729	-€ 34,139,639
3780	0.26	€ 410,654	€ 821,309	##########	##########	-€ 9,929,498	-€ 19,858,997	-€ 29,788,495	-€ 39,717,994
3781	0.21	€ 326,038	€ 652,076	€ 978,113	##########	-€ 7,883,497	-€ 15,766,993	-€ 23,650,490	-€ 31,533,987
3782	0.15	€ 235,945	€ 471,891	€ 707,836	€ 943,782	-€ 5,705,090	-€ 11,410,180	-€ 17,115,270	-€ 22,820,360
3783	0.19	€ 298,220	€ 596,441	€ 894,661	##########	-€ 7,210,878	-€ 14,421,756	-€ 21,632,634	-€ 28,843,512
3784	0.23	€ 359,999	€ 719,998	##########	##########	-€ 8,704,663	-€ 17,409,326	-€ 26,113,989	-€ 34,818,652
3785	0.24	€ 377,505	€ 755,010	##########	##########	-€ 9,127,955	-€ 18,255,909	-€ 27,383,864	-€ 36,511,818
3786	0.14	€ 220,968	€ 441,937	€ 662,905	€ 883,873	-€ 5,342,948	-€ 10,685,896	-€ 16,028,844	-€ 21,371,792
3787	0.13	€ 211,296	€ 422,593	€ 633,889	€ 845,186	-€ 5,109,085	-€ 10,218,171	-€ 15,327,256	-€ 20,436,342
3788	0.29	€ 459,816	€ 919,632	##########	##########	-€ 11,118,215	-€ 22,236,430	-€ 33,354,645	-€ 44,472,860
3789	0.11	€ 168,590	€ 337,181	€ 505,771	€ 674,362	-€ 4,076,466	-€ 8,152,932	-€ 12,229,398	-€ 16,305,864
3790	0.18	€ 280,439	€ 560,879	€ 841,318	##########	-€ 6,780,940	-€ 13,561,880	-€ 20,342,820	-€ 27,123,760
3791	0.22	€ 345,571	€ 691,142	##########	##########	-€ 8,355,809	-€ 16,711,618	-€ 25,067,427	-€ 33,423,236
3792	0.19	€ 292,748	€ 585,496	€ 878,243	##########	-€ 7,078,555	-€ 14,157,110	-€ 21,235,665	-€ 28,314,221
3793	0.19	€ 292,554	€ 585,107	€ 877,661	##########	-€ 7,073,862	-€ 14,147,724	-€ 21,221,586	-€ 28,295,447
3794	0.17	€ 261,430	€ 522,861	€ 784,291	##########	-€ 6,321,308	-€ 12,642,617	-€ 18,963,925	-€ 25,285,233
3795	0.17	€ 266,247	€ 532,495	€ 798,742	##########	-€ 6,437,782	-€ 12,875,563	-€ 19,313,345	-€ 25,751,127
3796	0.19	€ 296,840	€ 593,680	€ 890,521	##########	-€ 7,177,508	-€ 14,355,016	-€ 21,532,525	-€ 28,710,033
3797	0.25	€ 393,157	€ 786,315	##########	##########	-€ 9,506,428	-€ 19,012,855	-€ 28,519,283	-€ 38,025,711
3798	0.21	€ 324,041	€ 648,082	€ 972,123	##########	-€ 7,835,216	-€ 15,670,433	-€ 23,505,649	-€ 31,340,866
3799	0.14	€ 225,696	€ 451,393	€ 677,089	€ 902,786	-€ 5,457,272	-€ 10,914,544	-€ 16,371,815	-€ 21,829,087
3800	0.19	€ 306,079	€ 612,159	€ 918,238	##########	-€ 7,400,906	-€ 14,801,811	-€ 22,202,717	-€ 29,603,623
3801	0.22	€ 342,099	€ 684,198	##########	##########	-€ 8,271,853	-€ 16,543,706	-€ 24,815,558	-€ 33,087,411
3802	0.22	€ 347,595	€ 695,190	##########	##########	-€ 8,404,746	-€ 16,809,491	-€ 25,214,237	-€ 33,618,982
3803	0.21	€ 327,014	€ 654,028	€ 981,042	##########	-€ 7,907,103	-€ 15,814,207	-€ 23,721,310	-€ 31,628,413
3804	0.26	€ 401,949	€ 803,899	##########	##########	-€ 9,719,019	-€ 19,438,037	-€ 29,157,056	-€ 38,876,075
3805	0.22	€ 339,783	€ 679,566	##########	##########	-€ 8,215,850	-€ 16,431,700	-€ 24,647,550	-€ 32,863,400
3806	0.20	€ 321,163	€ 642,327	€ 963,490	##########	-€ 7,765,633	-€ 15,531,266	-€ 23,296,900	-€ 31,062,533
3807	0.15	€ 232,801	€ 465,601	€ 698,402	€ 931,202	-€ 5,629,049	-€ 11,258,097	-€ 16,887,146	-€ 22,516,195
3808	0.18	€ 278,641	€ 557,281	€ 835,922	##########	-€ 6,737,449	-€ 13,474,898	-€ 20,212,347	-€ 26,949,796
3809	0.17	€ 270,644	€ 541,288	€ 811,932	##########	-€ 6,544,091	-€ 13,088,183	-€ 19,632,274	-€ 26,176,365
3810	0.16	€ 256,844	€ 513,688	€ 770,532	##########	-€ 6,210,409	-€ 12,420,818	-€ 18,631,227	-€ 24,841,636
3811	0.20	€ 317,322	€ 634,644	€ 951,966	##########	-€ 7,672,753	-€ 15,345,507	-€ 23,018,260	-€ 30,691,014
3812	0.18	€ 282,715	€ 565,429	€ 848,144	##########	-€ 6,835,957	-€ 13,671,915	-€ 20,507,872	-€ 27,343,830
3813	0.23	€ 365,405	€ 730,811	##########	##########	-€ 8,835,394	-€ 17,670,788	-€ 26,506,182	-€ 35,341,576
3814	0.15	€ 232,592	€ 465,184	€ 697,777	€ 930,369	-€ 5,624,011	-€ 11,248,022	-€ 16,872,034	-€ 22,496,045
3815	0.20	€ 322,482	€ 644,963	€ 967,445	##########	-€ 7,797,511	-€ 15,595,021	-€ 23,392,532	-€ 31,190,042
3816	0.17	€ 263,333	€ 526,667	€ 790,000	##########	-€ 6,367,321	-€ 12,734,643	-€ 19,101,964	-€ 25,469,286
3817	0.19	€ 297,919	€ 595,838	€ 893,757	##########	-€ 7,203,590	-€ 14,407,179	-€ 21,610,769	-€ 28,814,359
3818	0.23	€ 358,280	€ 716,559	##########	##########	-€ 8,663,093	-€ 17,326,186	-€ 25,989,279	-€ 34,652,372
3819	0.18	€ 288,115	€ 576,230	€ 864,345	##########	-€ 6,966,536	-€ 13,933,072	-€ 20,899,608	-€ 27,866,144
3820	0.27	€ 424,089	€ 848,178	##########	##########	-€ 10,254,352	-€ 20,508,703	-€ 30,763,055	-€ 41,017,407
3821	0.24	€ 378,701	€ 757,403	##########	##########	-€ 9,156,886	-€ 18,313,772	-€ 27,470,659	-€ 36,627,545
3822	0.19	€ 297,715	€ 595,429	€ 893,144	##########	-€ 7,198,653	-€ 14,397,306	-€ 21,595,959	-€ 28,794,612
3823	0.23	€ 361,878	€ 723,757	##########	##########	-€ 8,750,111	-€ 17,500,223	-€ 26,250,334	-€ 35,000,446
3824	0.17	€ 260,124	€ 520,248	€ 780,372	##########	-€ 6,289,722	-€ 12,579,444	-€ 18,869,166	-€ 25,158,887
3825	0.23	€ 362,306	€ 724,612	##########	##########	-€ 8,760,453	-€ 17,520,905	-€ 26,281,358	-€ 35,041,810
3826	0.19	€ 296,279	€ 592,557	€ 888,836	##########	-€ 7,163,930	-€ 14,327,860	-€ 21,491,789	-€ 28,655,719
3827	0.17	€ 263,384	€ 526,769	€ 790,153	##########	-€ 6,368,553	-€ 12,737,106	-€ 19,105,660	-€ 25,474,213
3828	0.19	€ 295,152	€ 590,304	€ 885,455	##########	-€ 7,136,683	-€ 14,273,366	-€ 21,410,049	-€ 28,546,732
3829	0.16	€ 258,841	€ 517,682	€ 776,523	##########	-€ 6,258,701	-€ 12,517,401	-€ 18,776,102	-€ 25,034,802
3830	0.17	€ 261,571	€ 523,141	€ 784,712	##########	-€ 6,324,702	-€ 12,649,403	-€ 18,974,105	-€ 25,298,806
3831	0.18	€ 281,127	€ 562,255	€ 843,382	##########	-€ 6,797,575	-€ 13,595,149	-€ 20,392,724	-€ 27,190,299
3832	0.18	€ 281,416	€ 562,832	€ 844,248	##########	-€ 6,804,553	-€ 13,609,105	-€ 20,413,658	-€ 27,218,211
3833	0.24	€ 384,824	€ 769,648	##########	##########	-€ 9,304,928	-€ 18,609,857	-€ 27,914,785	-€ 37,219,714
3834	0.18	€ 278,122	€ 556,244	€ 834,366	##########	-€ 6,724,905	-€ 13,449,809	-€ 20,174,714	-€ 26,899,618
3835	0.20	€ 319,615	€ 639,230	€ 958,845	##########	-€ 7,728,197	-€ 15,456,394	-€ 23,184,592	-€ 30,912,789
3836	0.20	€ 318,454	€ 636,908	€ 955,362	##########	-€ 7,700,126	-€ 15,400,253	-€ 23,100,379	-€ 30,800,505
3837	0.14	€ 224,291	€ 448,583	€ 672,874	€ 897,166	-€ 5,423,300	-€ 10,846,601	-€ 16,269,901	-€ 21,693,201
3838	0.28	€ 447,434	€ 894,868	##########	##########	-€ 10,818,819	-€ 21,637,638	-€ 32,456,457	-€ 43,275,276
3839	0.18	€ 282,795	€ 565,591	€ 848,386	##########	-€ 6,837,908	-€ 13,675,816	-€ 20,513,724	-€ 27,351,632
3840	0.15	€ 243,152	€ 486,304	€ 729,456	€ 972,608	-€ 5,879,342	-€ 11,758,685	-€ 17,638,027	-€ 23,517,369
3841	0.15	€ 238,479	€ 476,958	€ 715,436	€ 953,915	-€ 5,766,346	-€ 11,532,691	-€ 17,299,037	-€ 23,065,383
3842	0.16	€ 253,857	€ 507,714	€ 761,571	##########	-€ 6,138,189	-€ 12,276,377	-€ 18,414,566	-€ 24,552,755
3843	0.16	€ 247,655	€ 495,309	€ 742,964	€ 990,618	-€ 5,988,213	-€ 11,976,425	-€ 17,964,638	-€ 23,952,851
3844	0.23	€ 360,032	€ 720,064	##########	##########	-€ 8,705,466	-€ 17,410,932	-€ 26,116,398	-€ 34,821,864
3845	0.21	€ 333,691	€ 667,381	##########	##########	-€ 8,068,542	-€ 16,137,083	-€ 24,205,625	-€ 32,274,166
3846	0.21	€ 331,929	€ 663,858	€ 995,786	##########	-€ 8,025,940	-€ 16,051,879	-€ 24,077,819	-€ 32,103,759
3847	0.24	€ 373,093	€ 746,185	##########	##########	-€ 9,021,268	-€ 18,042,537	-€ 27,063,805	-€ 36,085,073
3848	0.16	€ 249,610	€ 499,220	€ 748,830	€ 998,440	-€ 6,035,496	-€ 12,070,992	-€ 18,106,488	-€ 24,141,984
3849	0.21	€ 329,920	€ 659,840	€ 989,760	##########	-€ 7,977,368	-€ 15,954,735	-€ 23,932,103	-€ 31,909,471
3850	0.17	€ 271,929	€ 543,858	€ 815,787	##########	-€ 6,575,160	-€ 13,150,320	-€ 19,725,480	-€ 26,300,639
3851	0.16	€ 247,669	€ 495,339	€ 743,008	€ 990,677	-€ 5,988,571	-€ 11,977,141	-€ 17,965,712	-€ 23,954,283
3852	0.16	€ 247,796	€ 495,593	€ 743,389	€ 991,185	-€ 5,991,642	-€ 11,983,284	-€ 17,974,927	-€ 23,966,569
3853	0.21	€ 330,769	€ 661,538	€ 992,306	##########	-€ 7,997,892	-€ 15,995,783	-€ 23,993,675	-€ 31,991,566
3854	0.19	€ 302,986	€ 605,972	€ 908,958	##########	-€ 7,326,112	-€ 14,652,224	-€ 21,978,336	-€ 29,304,447
3855	0.24	€ 378,261	€ 756,522	##########	##########	-€ 9,146,237	-€ 18,292,473	-€ 27,438,710	-€ 36,584,946
3856	0.22	€ 346,012	€ 692,024	##########	##########	-€ 8,366,464	-€ 16,732,928	-€ 25,099,392	-€ 33,465,855
3857	0.20	€ 309,688	€ 619,375	€ 929,063	##########	-€ 7,488,157	-€ 14,976,314	-€ 22,464,471	-€ 29,952,628
3858	0.21	€ 332,280	€ 664,560	€ 996,840	##########	-€ 8,034,435	-€ 16,068,871	-€ 24,103,306	-€ 32,137,742
3859	0.19	€ 304,185	€ 608,370	€ 912,555	##########	-€ 7,355,102	-€ 14,710,204	-€ 22,065,306	-€ 29,420,408
3860	0.20	€ 310,338	€ 620,677	€ 931,015	##########	-€ 7,503,890	-€ 15,007,780	-€ 22,511,670	-€ 30,015,560
3861	0.16	€ 246,716	€ 493,431	€ 740,147	€ 986,862	-€ 5,965,510	-€ 11,931,021	-€ 17,896,531	-€ 23,862,042
3862	0.24	€ 372,941	€ 745,882	##########	##########	-€ 9,017,606	-€ 18,035,212	-€ 27,052,818	-€ 36,070,424
3863	0.22	€ 345,032	€ 690,063	##########	##########	-€ 8,342,760	-€ 16,685,520	-€ 25,028,279	-€ 33,371,039
3864	0.17	€ 266,992	€ 533,984	€ 800,976	##########	-€ 6,455,791	-€ 12,911,583	-€ 19,367,374	-€ 25,823,165
3865	0.17	€ 261,363	€ 522,726	€ 784,089	##########	-€ 6,319,681	-€ 12,639,362	-€ 18,959,043	-€ 25,278,724
3866	0.20	€ 310,914	€ 621,828	€ 932,742	##########	-€ 7,517,812	-€ 15,035,623	-€ 22,553,435	-€ 30,071,246
3867	0.22	€ 342,987	€ 685,975	##########	##########	-€ 8,293,331	-€ 16,586,663	-€ 24,879,994	-€ 33,173,325
3868	0.20	€ 319,425	€ 638,850	€ 958,275	##########	-€ 7,723,604	-€ 15,447,208	-€ 23,170,812	-€ 30,894,416
3869	0.11	€ 167,791	€ 335,581	€ 503,372	€ 671,163	-€ 4,057,129	-€ 8,114,259	-€ 12,171,388	-€ 16,228,518
3870	0.19	€ 305,694	€ 611,387	€ 917,081	##########	-€ 7,391,583	-€ 14,783,165	-€ 22,174,748	-€ 29,566,330
3871	0.18	€ 283,725	€ 567,451	€ 851,176	##########	-€ 6,860,394	-€ 13,720,788	-€ 20,581,182	-€ 27,441,576
3872	0.16	€ 250,331	€ 500,662	€ 750,993	##########	-€ 6,052,926	-€ 12,105,851	-€ 18,158,777	-€ 24,211,702

3873	0.30	€ 473,993	€ 947,985	##########	##########	-€ 11,461,000	-€ 22,922,000	-€ 34,383,000	-€ 45,844,000
3874	0.20	€ 319,966	€ 639,931	€ 959,897	##########	-€ 7,736,677	-€ 15,473,353	-€ 23,210,030	-€ 30,946,707
3875	0.15	€ 243,385	€ 486,771	€ 730,156	€ 973,542	-€ 5,884,988	-€ 11,769,976	-€ 17,654,963	-€ 23,539,951
3876	0.23	€ 363,334	€ 726,667	##########	##########	-€ 8,785,299	-€ 17,570,599	-€ 26,355,898	-€ 35,141,198
3877	0.22	€ 350,904	€ 701,808	##########	##########	-€ 8,484,756	-€ 16,969,512	-€ 25,454,267	-€ 33,939,023
3878	0.17	€ 260,657	€ 521,313	€ 781,970	##########	-€ 6,302,599	-€ 12,605,197	-€ 18,907,796	-€ 25,210,395
3879	0.18	€ 287,584	€ 575,167	€ 862,751	##########	-€ 6,953,685	-€ 13,907,370	-€ 20,861,055	-€ 27,814,741
3880	0.16	€ 249,472	€ 498,945	€ 748,417	€ 997,889	-€ 6,032,167	-€ 12,064,334	-€ 18,096,500	-€ 24,128,667
3881	0.22	€ 341,666	€ 683,333	##########	##########	-€ 8,261,391	-€ 16,522,782	-€ 24,784,174	-€ 33,045,565
3882	0.22	€ 344,646	€ 689,291	##########	##########	-€ 8,333,427	-€ 16,666,855	-€ 25,000,282	-€ 33,333,710
3883	0.19	€ 293,499	€ 586,999	€ 880,498	##########	-€ 7,096,727	-€ 14,193,454	-€ 21,290,181	-€ 28,386,908
3884	0.23	€ 362,235	€ 724,470	##########	##########	-€ 8,758,739	-€ 17,517,479	-€ 26,276,218	-€ 35,034,958
3885	0.23	€ 369,685	€ 739,370	##########	##########	-€ 8,938,872	-€ 17,877,743	-€ 26,816,615	-€ 35,755,486
3886	0.19	€ 298,508	€ 597,016	€ 895,524	##########	-€ 7,217,835	-€ 14,435,670	-€ 21,653,505	-€ 28,871,339
3887	0.15	€ 244,002	€ 488,004	€ 732,006	€ 976,008	-€ 5,899,895	-€ 11,799,789	-€ 17,699,684	-€ 23,599,579
3888	0.16	€ 247,055	€ 494,109	€ 741,164	€ 988,218	-€ 5,973,707	-€ 11,947,415	-€ 17,921,122	-€ 23,894,829
3889	0.18	€ 288,891	€ 577,783	€ 866,674	##########	-€ 6,985,309	-€ 13,970,618	-€ 20,955,927	-€ 27,941,237
3890	0.27	€ 420,573	€ 841,146	##########	##########	-€ 10,169,327	-€ 20,338,653	-€ 30,507,980	-€ 40,677,307
3891	0.28	€ 435,691	€ 871,381	##########	##########	-€ 10,534,869	-€ 21,069,739	-€ 31,604,608	-€ 42,139,478
3892	0.19	€ 306,025	€ 612,049	€ 918,074	##########	-€ 7,399,582	-€ 14,799,164	-€ 22,198,747	-€ 29,598,329
3893	0.16	€ 250,461	€ 500,922	€ 751,383	##########	-€ 6,056,071	-€ 12,112,141	-€ 18,168,212	-€ 24,224,283
3894	0.22	€ 350,081	€ 700,161	##########	##########	-€ 8,464,849	-€ 16,929,697	-€ 25,394,546	-€ 33,859,394
3895	0.19	€ 299,706	€ 599,411	€ 899,117	##########	-€ 7,246,795	-€ 14,493,591	-€ 21,740,386	-€ 28,987,182
3896	0.19	€ 300,053	€ 600,105	€ 900,158	##########	-€ 7,255,182	-€ 14,510,365	-€ 21,765,547	-€ 29,020,729
3897	0.16	€ 245,390	€ 490,780	€ 736,170	€ 981,560	-€ 5,933,454	-€ 11,866,909	-€ 17,800,363	-€ 23,733,818
3898	0.16	€ 258,588	€ 517,176	€ 775,764	##########	-€ 6,252,584	-€ 12,505,168	-€ 18,757,751	-€ 25,010,335
3899	0.23	€ 357,613	€ 715,226	##########	##########	-€ 8,646,978	-€ 17,293,957	-€ 25,940,935	-€ 34,587,913
3900	0.23	€ 355,930	€ 711,860	##########	##########	-€ 8,606,280	-€ 17,212,559	-€ 25,818,839	-€ 34,425,118
3901	0.15	€ 232,183	€ 464,365	€ 696,548	€ 928,730	-€ 5,614,105	-€ 11,228,210	-€ 16,842,315	-€ 22,456,421
3902	0.19	€ 298,289	€ 596,578	€ 894,867	##########	-€ 7,212,540	-€ 14,425,080	-€ 21,637,620	-€ 28,850,160
3903	0.22	€ 343,340	€ 686,680	##########	##########	-€ 8,301,854	-€ 16,603,709	-€ 24,905,563	-€ 33,207,418
3904	0.17	€ 270,663	€ 541,326	€ 811,989	##########	-€ 6,544,549	-€ 13,089,098	-€ 19,633,647	-€ 26,178,196
3905	0.16	€ 254,833	€ 509,666	€ 764,499	##########	-€ 6,161,788	-€ 12,323,577	-€ 18,485,365	-€ 24,647,153
3906	0.18	€ 287,675	€ 575,351	€ 863,026	##########	-€ 6,955,903	-€ 13,911,805	-€ 20,867,708	-€ 27,823,611
3907	0.13	€ 210,280	€ 420,559	€ 630,839	€ 841,119	-€ 5,084,499	-€ 10,168,998	-€ 15,253,497	-€ 20,337,996
3908	0.25	€ 389,062	€ 778,125	##########	##########	-€ 9,407,415	-€ 18,814,831	-€ 28,222,246	-€ 37,629,661
3909	0.13	€ 210,148	€ 420,297	€ 630,445	€ 840,593	-€ 5,081,323	-€ 10,162,646	-€ 15,243,970	-€ 20,325,293
3910	0.18	€ 284,333	€ 568,667	€ 853,000	##########	-€ 6,875,096	-€ 13,750,192	-€ 20,625,288	-€ 27,500,384
3911	0.16	€ 256,215	€ 512,431	€ 768,646	##########	-€ 6,195,214	-€ 12,390,429	-€ 18,585,643	-€ 24,780,858
3912	0.22	€ 339,831	€ 679,661	##########	##########	-€ 8,217,006	-€ 16,434,012	-€ 24,651,017	-€ 32,868,023
3913	0.17	€ 269,561	€ 539,121	€ 808,682	##########	-€ 6,517,899	-€ 13,035,798	-€ 19,553,697	-€ 26,071,595
3914	0.19	€ 304,822	€ 609,645	€ 914,467	##########	-€ 7,370,512	-€ 14,741,024	-€ 22,111,537	-€ 29,482,049
3915	0.27	€ 430,182	€ 860,365	##########	##########	-€ 10,401,685	-€ 20,803,370	-€ 31,205,055	-€ 41,606,740
3916	0.13	€ 208,151	€ 416,302	€ 624,454	€ 832,605	-€ 5,033,035	-€ 10,066,070	-€ 15,099,105	-€ 20,132,140
3917	0.21	€ 328,258	€ 656,516	€ 984,774	##########	-€ 7,937,182	-€ 15,874,364	-€ 23,811,546	-€ 31,748,728
3918	0.20	€ 315,879	€ 631,758	€ 947,637	##########	-€ 7,637,864	-€ 15,275,729	-€ 22,913,593	-€ 30,551,458
3919	0.20	€ 316,991	€ 633,982	€ 950,972	##########	-€ 7,664,744	-€ 15,329,488	-€ 22,994,232	-€ 30,658,976
3920	0.16	€ 248,459	€ 496,918	€ 745,377	€ 993,835	-€ 6,007,661	-€ 12,015,322	-€ 18,022,983	-€ 24,030,644
3921	0.20	€ 310,927	€ 621,853	€ 932,780	##########	-€ 7,518,111	-€ 15,036,222	-€ 22,554,333	-€ 30,072,445
3922	0.20	€ 315,699	€ 631,399	€ 947,098	##########	-€ 7,633,517	-€ 15,267,035	-€ 22,900,552	-€ 30,534,070
3923	0.17	€ 275,504	€ 551,008	€ 826,511	##########	-€ 6,661,599	-€ 13,323,199	-€ 19,984,798	-€ 26,646,398
3924	0.20	€ 307,465	€ 614,930	€ 922,395	##########	-€ 7,434,410	-€ 14,868,821	-€ 22,303,231	-€ 29,737,641
3925	0.17	€ 271,669	€ 543,338	€ 815,007	##########	-€ 6,568,877	-€ 13,137,754	-€ 19,706,631	-€ 26,275,508
3926	0.21	€ 329,450	€ 658,900	€ 988,349	##########	-€ 7,965,998	-€ 15,931,997	-€ 23,897,995	-€ 31,863,994
3927	0.20	€ 319,155	€ 638,310	€ 957,465	##########	-€ 7,717,076	-€ 15,434,152	-€ 23,151,227	-€ 30,868,303
3928	0.16	€ 255,098	€ 510,196	€ 765,294	##########	-€ 6,168,194	-€ 12,336,387	-€ 18,504,581	-€ 24,672,774
3929	0.15	€ 228,989	€ 457,979	€ 686,968	€ 915,957	-€ 5,536,893	-€ 11,073,787	-€ 16,610,680	-€ 22,147,574
3930	0.18	€ 279,242	€ 558,484	€ 837,726	##########	-€ 6,751,992	-€ 13,503,983	-€ 20,255,975	-€ 27,007,967
3931	0.14	€ 224,994	€ 449,988	€ 674,982	€ 899,976	-€ 5,440,290	-€ 10,880,580	-€ 16,320,870	-€ 21,761,160
3932	0.23	€ 356,473	€ 712,947	##########	##########	-€ 8,619,422	-€ 17,238,845	-€ 25,858,267	-€ 34,477,690
3933	0.19	€ 295,002	€ 590,004	€ 885,006	##########	-€ 7,133,061	-€ 14,266,122	-€ 21,399,184	-€ 28,532,245
3934	0.25	€ 387,740	€ 775,481	##########	##########	-€ 9,375,449	-€ 18,750,898	-€ 28,126,347	-€ 37,501,795
3935	0.21	€ 332,559	€ 665,118	€ 997,677	##########	-€ 8,041,176	-€ 16,082,352	-€ 24,123,528	-€ 32,164,704
3936	0.19	€ 300,476	€ 600,951	€ 901,427	##########	-€ 7,265,411	-€ 14,530,821	-€ 21,796,232	-€ 29,061,642
3937	0.16	€ 255,625	€ 511,249	€ 766,874	##########	-€ 6,180,929	-€ 12,361,859	-€ 18,542,788	-€ 24,723,718
3938	0.12	€ 190,344	€ 380,688	€ 571,031	€ 761,375	-€ 4,602,456	-€ 9,204,911	-€ 13,807,367	-€ 18,409,822
3939	0.11	€ 174,275	€ 348,551	€ 522,826	€ 697,102	-€ 4,213,929	-€ 8,427,859	-€ 12,641,788	-€ 16,855,717
3940	0.20	€ 309,022	€ 618,043	€ 927,065	##########	-€ 7,472,054	-€ 14,944,108	-€ 22,416,162	-€ 29,888,216
3941	0.21	€ 326,432	€ 652,865	€ 979,297	##########	-€ 7,893,041	-€ 15,786,081	-€ 23,679,122	-€ 31,572,163
3942	0.15	€ 242,349	€ 484,698	€ 727,047	€ 969,397	-€ 5,859,930	-€ 11,719,860	-€ 17,579,790	-€ 23,439,720
3943	0.18	€ 291,117	€ 582,234	€ 873,352	##########	-€ 7,039,129	-€ 14,078,257	-€ 21,117,386	-€ 28,156,514
3944	0.16	€ 256,067	€ 512,134	€ 768,202	##########	-€ 6,191,629	-€ 12,383,258	-€ 18,574,887	-€ 24,766,515
3945	0.31	€ 482,853	€ 965,705	##########	##########	-€ 11,675,235	-€ 23,350,470	-€ 35,025,705	-€ 46,700,940
3946	0.21	€ 334,249	€ 668,497	##########	##########	-€ 8,082,034	-€ 16,164,068	-€ 24,246,101	-€ 32,328,135
3947	0.21	€ 334,312	€ 668,623	##########	##########	-€ 8,083,554	-€ 16,167,108	-€ 24,250,661	-€ 32,334,215
3948	0.13	€ 211,893	€ 423,786	€ 635,680	€ 847,573	-€ 5,123,514	-€ 10,247,028	-€ 15,370,542	-€ 20,494,056
3949	0.17	€ 271,530	€ 543,060	€ 814,590	##########	-€ 6,565,519	-€ 13,131,037	-€ 19,696,556	-€ 26,262,075
3950	0.18	€ 291,192	€ 582,383	€ 873,575	##########	-€ 7,040,930	-€ 14,081,860	-€ 21,122,790	-€ 28,163,720
3951	0.16	€ 249,917	€ 499,834	€ 749,751	€ 999,668	-€ 6,042,918	-€ 12,085,836	-€ 18,128,754	-€ 24,171,672
3952	0.16	€ 250,997	€ 501,995	€ 752,992	##########	-€ 6,069,043	-€ 12,138,086	-€ 18,207,129	-€ 24,276,172
3953	0.18	€ 281,398	€ 562,797	€ 844,195	##########	-€ 6,804,130	-€ 13,608,260	-€ 20,412,390	-€ 27,216,520
3954	0.14	€ 225,495	€ 450,990	€ 676,485	€ 901,980	-€ 5,452,404	-€ 10,904,808	-€ 16,357,213	-€ 21,809,617
3955	0.16	€ 258,487	€ 516,975	€ 775,462	##########	-€ 6,250,146	-€ 12,500,291	-€ 18,750,437	-€ 25,000,582
3956	0.15	€ 233,967	€ 467,935	€ 701,902	€ 935,869	-€ 5,657,261	-€ 11,314,523	-€ 16,971,784	-€ 22,629,046
3957	0.19	€ 302,251	€ 604,502	€ 906,753	##########	-€ 7,308,337	-€ 14,616,674	-€ 21,925,011	-€ 29,233,348
3958	0.23	€ 357,605	€ 715,211	##########	##########	-€ 8,646,792	-€ 17,293,584	-€ 25,940,376	-€ 34,587,168
3959	0.21	€ 329,257	€ 658,514	€ 987,771	##########	-€ 7,961,340	-€ 15,922,680	-€ 23,884,020	-€ 31,845,360
3960	0.18	€ 276,960	€ 553,920	€ 830,880	##########	-€ 6,696,809	-€ 13,393,619	-€ 20,090,428	-€ 26,787,237
3961	0.15	€ 230,924	€ 461,848	€ 692,772	€ 923,696	-€ 5,583,672	-€ 11,167,344	-€ 16,751,016	-€ 22,334,688
3962	0.19	€ 298,987	€ 597,974	€ 896,960	##########	-€ 7,229,413	-€ 14,458,825	-€ 21,688,238	-€ 28,917,651
3963	0.17	€ 264,522	€ 529,043	€ 793,565	##########	-€ 6,396,053	-€ 12,792,106	-€ 19,188,159	-€ 25,584,213
3964	0.15	€ 238,874	€ 477,747	€ 716,621	€ 955,495	-€ 5,775,894	-€ 11,551,788	-€ 17,327,683	-€ 23,103,577
3965	0.18	€ 284,156	€ 568,313	€ 852,469	##########	-€ 6,870,816	-€ 13,741,631	-€ 20,612,447	-€ 27,483,263
3966	0.18	€ 286,430	€ 572,861	€ 859,291	##########	-€ 6,925,800	-€ 13,851,601	-€ 20,777,401	-€ 27,703,201
3967	0.17	€ 259,849	€ 519,699	€ 779,548	##########	-€ 6,283,082	-€ 12,566,164	-€ 18,849,246	-€ 25,132,328
3968	0.20	€ 322,533	€ 645,067	€ 967,600	##########	-€ 7,798,762	-€ 15,597,524	-€ 23,396,286	-€ 31,195,048
3969	0.21	€ 329,024	€ 658,047	€ 987,071	##########	-€ 7,955,694	-€ 15,911,388	-€ 23,867,083	-€ 31,822,777
3970	0.21	€ 324,150	€ 648,299	€ 972,449	##########	-€ 7,837,841	-€ 15,675,682	-€ 23,513,523	-€ 31,351,364
3971	0.20	€ 310,754	€ 621,509	€ 932,263	##########	-€ 7,513,947	-€ 15,027,893	-€ 22,541,840	-€ 30,055,786
3972	0.18	€ 287,760	€ 575,521	€ 863,281	##########	-€ 6,957,959	-€ 13,915,919	-€ 20,873,878	-€ 27,831,837
3973	0.19	€ 297,683	€ 595,366	€ 893,050	##########	-€ 7,197,891	-€ 14,395,781	-€ 21,593,672	-€ 28,791,563
3974	0.28	€ 433,468	€ 866,936	##########	##########	-€ 10,481,127	-€ 20,962,255	-€ 31,443,382	-€ 41,924,509

3975	0.17	€ 266,889	€ 533,778	€ 800,667	##########	-€ 6,453,301	-€ 12,906,601	-€ 19,359,902	-€ 25,813,203
3976	0.21	€ 326,335	€ 652,670	€ 979,005	##########	-€ 7,890,681	-€ 15,781,362	-€ 23,672,042	-€ 31,562,723
3977	0.18	€ 275,800	€ 551,600	€ 827,400	##########	-€ 6,668,763	-€ 13,337,525	-€ 20,006,288	-€ 26,675,051
3978	0.23	€ 360,460	€ 720,919	##########	##########	-€ 8,715,806	-€ 17,431,612	-€ 26,147,418	-€ 34,863,224
3979	0.17	€ 274,001	€ 548,002	€ 822,002	##########	-€ 6,625,257	-€ 13,250,514	-€ 19,875,770	-€ 26,501,027
3980	0.22	€ 338,636	€ 677,272	##########	##########	-€ 8,188,122	-€ 16,376,245	-€ 24,564,367	-€ 32,752,490
3981	0.26	€ 415,012	€ 830,023	##########	##########	-€ 10,034,861	-€ 20,069,721	-€ 30,104,582	-€ 40,139,442
3982	0.16	€ 259,278	€ 518,557	€ 777,835	##########	-€ 6,269,275	-€ 12,538,550	-€ 18,807,825	-€ 25,077,101
3983	0.27	€ 431,580	€ 863,160	##########	##########	-€ 10,435,482	-€ 20,870,963	-€ 31,306,445	-€ 41,741,926
3984	0.16	€ 246,313	€ 492,627	€ 738,940	€ 985,253	-€ 5,955,784	-€ 11,911,567	-€ 17,867,351	-€ 23,823,135
3985	0.22	€ 341,363	€ 682,726	##########	##########	-€ 8,254,056	-€ 16,508,113	-€ 24,762,169	-€ 33,016,226
3986	0.15	€ 234,361	€ 468,722	€ 703,083	€ 937,444	-€ 5,666,778	-€ 11,333,556	-€ 17,000,333	-€ 22,667,111
3987	0.19	€ 293,023	€ 586,046	€ 879,069	##########	-€ 7,085,208	-€ 14,170,416	-€ 21,255,624	-€ 28,340,832
3988	0.18	€ 280,891	€ 561,783	€ 842,674	##########	-€ 6,791,873	-€ 13,583,746	-€ 20,375,619	-€ 27,167,492
3989	0.18	€ 281,911	€ 563,821	€ 845,732	##########	-€ 6,816,517	-€ 13,633,034	-€ 20,449,551	-€ 27,266,067
3990	0.20	€ 312,628	€ 625,256	€ 937,884	##########	-€ 7,559,254	-€ 15,118,507	-€ 22,677,761	-€ 30,237,015
3991	0.19	€ 302,395	€ 604,790	€ 907,186	##########	-€ 7,311,826	-€ 14,623,653	-€ 21,935,479	-€ 29,247,306
3992	0.26	€ 417,135	€ 834,269	##########	##########	-€ 10,086,191	-€ 20,172,382	-€ 30,258,573	-€ 40,344,764
3993	0.16	€ 255,609	€ 511,219	€ 766,828	##########	-€ 6,180,561	-€ 12,361,122	-€ 18,541,683	-€ 24,722,244
3994	0.16	€ 255,348	€ 510,696	€ 766,044	##########	-€ 6,174,238	-€ 12,348,476	-€ 18,522,713	-€ 24,696,951
3995	0.20	€ 311,220	€ 622,440	€ 933,660	##########	-€ 7,525,210	-€ 15,050,419	-€ 22,575,629	-€ 30,100,838
3996	0.12	€ 185,550	€ 371,099	€ 556,649	€ 742,198	-€ 4,486,532	-€ 8,973,064	-€ 13,459,597	-€ 17,946,129
3997	0.18	€ 278,428	€ 556,856	€ 835,283	##########	-€ 6,732,300	-€ 13,464,601	-€ 20,196,901	-€ 26,929,202
3998	0.15	€ 242,762	€ 485,523	€ 728,285	€ 971,047	-€ 5,869,907	-€ 11,739,814	-€ 17,609,720	-€ 23,479,627
3999	0.22	€ 344,737	€ 689,474	##########	##########	-€ 8,335,635	-€ 16,671,270	-€ 25,006,905	-€ 33,342,540
4000	0.17	€ 266,079	€ 532,159	€ 798,238	##########	-€ 6,433,718	-€ 12,867,435	-€ 19,301,153	-€ 25,734,871
4001	0.24	€ 380,963	€ 761,926	##########	##########	-€ 9,211,569	-€ 18,423,137	-€ 27,634,706	-€ 36,846,275
4002	0.23	€ 359,934	€ 719,868	##########	##########	-€ 8,703,102	-€ 17,406,205	-€ 26,109,307	-€ 34,812,409
4003	0.19	€ 299,526	€ 599,052	€ 898,578	##########	-€ 7,242,454	-€ 14,484,907	-€ 21,727,361	-€ 28,969,815
4004	0.22	€ 346,950	€ 693,900	##########	##########	-€ 8,389,154	-€ 16,778,308	-€ 25,167,462	-€ 33,556,615
4005	0.18	€ 289,851	€ 579,702	€ 869,554	##########	-€ 7,008,516	-€ 14,017,031	-€ 21,025,547	-€ 28,034,063
4006	0.23	€ 361,137	€ 722,274	##########	##########	-€ 8,732,184	-€ 17,464,368	-€ 26,196,553	-€ 34,928,737
4007	0.23	€ 362,823	€ 725,645	##########	##########	-€ 8,772,943	-€ 17,545,887	-€ 26,318,830	-€ 35,091,773
4008	0.20	€ 317,882	€ 635,764	€ 953,646	##########	-€ 7,686,295	-€ 15,372,589	-€ 23,058,884	-€ 30,745,178
4009	0.28	€ 434,659	€ 869,318	##########	##########	-€ 10,509,924	-€ 21,019,848	-€ 31,529,771	-€ 42,039,695
4010	0.23	€ 360,296	€ 720,593	##########	##########	-€ 8,711,858	-€ 17,423,715	-€ 26,135,573	-€ 34,847,431
4011	0.22	€ 345,762	€ 691,524	##########	##########	-€ 8,360,426	-€ 16,720,852	-€ 25,081,278	-€ 33,441,704
4012	0.21	€ 335,165	€ 670,329	##########	##########	-€ 8,104,178	-€ 16,208,357	-€ 24,312,535	-€ 32,416,714
4013	0.18	€ 287,615	€ 575,229	€ 862,844	##########	-€ 6,954,436	-€ 13,908,873	-€ 20,863,309	-€ 27,817,745
4014	0.24	€ 374,352	€ 748,704	##########	##########	-€ 9,051,717	-€ 18,103,433	-€ 27,155,150	-€ 36,206,866
4015	0.18	€ 278,564	€ 557,127	€ 835,691	##########	-€ 6,735,584	-€ 13,471,168	-€ 20,206,752	-€ 26,942,335
4016	0.12	€ 183,673	€ 367,345	€ 551,018	€ 734,690	-€ 4,441,149	-€ 8,882,297	-€ 13,323,446	-€ 17,764,594
4017	0.23	€ 355,660	€ 711,320	##########	##########	-€ 8,599,751	-€ 17,199,502	-€ 25,799,253	-€ 34,399,004
4018	0.18	€ 281,274	€ 562,548	€ 843,822	##########	-€ 6,801,123	-€ 13,602,246	-€ 20,403,369	-€ 27,204,492
4019	0.25	€ 395,229	€ 790,457	##########	##########	-€ 9,556,509	-€ 19,113,018	-€ 28,669,527	-€ 38,226,036
4020	0.22	€ 349,121	€ 698,241	##########	##########	-€ 8,441,635	-€ 16,883,271	-€ 25,324,906	-€ 33,766,541
4021	0.23	€ 356,941	€ 713,882	##########	##########	-€ 8,630,732	-€ 17,261,464	-€ 25,892,196	-€ 34,522,928
4022	0.25	€ 397,223	€ 794,447	##########	##########	-€ 9,604,743	-€ 19,209,487	-€ 28,814,230	-€ 38,418,974
4023	0.21	€ 329,387	€ 658,775	€ 988,162	##########	-€ 7,964,488	-€ 15,928,976	-€ 23,893,464	-€ 31,857,953
4024	0.18	€ 285,332	€ 570,664	€ 855,997	##########	-€ 6,899,247	-€ 13,798,494	-€ 20,697,741	-€ 27,596,988
4025	0.20	€ 316,079	€ 632,159	€ 948,238	##########	-€ 7,642,707	-€ 15,285,413	-€ 22,928,120	-€ 30,570,827
4026	0.19	€ 300,925	€ 601,849	€ 902,774	##########	-€ 7,276,270	-€ 14,552,539	-€ 21,828,809	-€ 29,105,079
4027	0.17	€ 260,375	€ 520,750	€ 781,125	##########	-€ 6,295,788	-€ 12,591,576	-€ 18,887,365	-€ 25,183,153
4028	0.22	€ 352,651	€ 705,302	##########	##########	-€ 8,526,994	-€ 17,053,988	-€ 25,580,983	-€ 34,107,977
4029	0.16	€ 256,570	€ 513,140	€ 769,710	##########	-€ 6,203,790	-€ 12,407,581	-€ 18,611,371	-€ 24,815,162
4030	0.14	€ 223,854	€ 447,708	€ 671,562	€ 895,416	-€ 5,412,726	-€ 10,825,452	-€ 16,238,177	-€ 21,650,903
4031	0.18	€ 285,898	€ 571,795	€ 857,693	##########	-€ 6,912,921	-€ 13,825,842	-€ 20,738,764	-€ 27,651,685
4032	0.23	€ 366,875	€ 733,751	##########	##########	-€ 8,870,937	-€ 17,741,875	-€ 26,612,812	-€ 35,483,749
4033	0.25	€ 398,105	€ 796,209	##########	##########	-€ 9,626,051	-€ 19,252,102	-€ 28,878,153	-€ 38,504,204
4034	0.18	€ 280,386	€ 560,771	€ 841,157	##########	-€ 6,779,643	-€ 13,559,286	-€ 20,338,928	-€ 27,118,571
4035	0.24	€ 380,946	€ 761,893	##########	##########	-€ 9,211,171	-€ 18,422,342	-€ 27,633,513	-€ 36,844,683
4036	0.14	€ 221,190	€ 442,380	€ 663,569	€ 884,759	-€ 5,348,303	-€ 10,696,606	-€ 16,044,909	-€ 21,393,212
4037	0.19	€ 293,388	€ 586,776	€ 880,164	##########	-€ 7,094,037	-€ 14,188,075	-€ 21,282,112	-€ 28,376,149
4038	0.24	€ 380,250	€ 760,500	##########	##########	-€ 9,194,334	-€ 18,388,669	-€ 27,583,003	-€ 36,777,337
4039	0.20	€ 321,523	€ 643,047	€ 964,570	##########	-€ 7,774,338	-€ 15,548,677	-€ 23,323,015	-€ 31,097,353
4040	0.12	€ 182,885	€ 365,770	€ 548,654	€ 731,539	-€ 4,422,099	-€ 8,844,198	-€ 13,266,297	-€ 17,688,396
4041	0.22	€ 347,476	€ 694,951	##########	##########	-€ 8,401,858	-€ 16,803,715	-€ 25,205,573	-€ 33,607,430
4042	0.23	€ 368,437	€ 736,873	##########	##########	-€ 8,908,686	-€ 17,817,372	-€ 26,726,058	-€ 35,634,743
4043	0.21	€ 328,666	€ 657,332	€ 985,998	##########	-€ 7,947,049	-€ 15,894,098	-€ 23,841,147	-€ 31,788,196
4044	0.17	€ 262,908	€ 525,816	€ 788,724	##########	-€ 6,357,036	-€ 12,714,072	-€ 19,071,107	-€ 25,428,143
4045	0.20	€ 312,357	€ 624,714	€ 937,070	##########	-€ 7,552,695	-€ 15,105,390	-€ 22,658,085	-€ 30,210,780
4046	0.18	€ 281,069	€ 562,138	€ 843,206	##########	-€ 6,796,159	-€ 13,592,318	-€ 20,388,478	-€ 27,184,637
4047	0.16	€ 252,323	€ 504,646	€ 756,969	##########	-€ 6,101,098	-€ 12,202,196	-€ 18,303,294	-€ 24,404,392
4048	0.16	€ 255,483	€ 510,966	€ 766,449	##########	-€ 6,177,505	-€ 12,355,009	-€ 18,532,514	-€ 24,710,018
4049	0.22	€ 345,967	€ 691,934	##########	##########	-€ 8,365,383	-€ 16,730,766	-€ 25,096,149	-€ 33,461,532
4050	0.19	€ 293,024	€ 586,049	€ 879,073	##########	-€ 7,085,242	-€ 14,170,485	-€ 21,255,727	-€ 28,340,970
4051	0.10	€ 152,499	€ 304,999	€ 457,498	€ 609,998	-€ 3,687,392	-€ 7,374,784	-€ 11,062,176	-€ 14,749,568
4052	0.26	€ 413,240	€ 826,479	##########	##########	-€ 9,992,013	-€ 19,984,026	-€ 29,976,038	-€ 39,968,051
4053	0.21	€ 338,224	€ 676,448	##########	##########	-€ 8,178,155	-€ 16,356,311	-€ 24,534,466	-€ 32,712,622
4054	0.13	€ 205,716	€ 411,433	€ 617,149	€ 822,865	-€ 4,974,159	-€ 9,948,318	-€ 14,922,478	-€ 19,896,637
4055	0.17	€ 273,138	€ 546,276	€ 819,414	##########	-€ 6,604,393	-€ 13,208,786	-€ 19,813,179	-€ 26,417,572
4056	0.20	€ 316,910	€ 633,819	€ 950,729	##########	-€ 7,662,780	-€ 15,325,560	-€ 22,988,340	-€ 30,651,119
4057	0.19	€ 306,880	€ 613,760	€ 920,641	##########	-€ 7,420,273	-€ 14,840,546	-€ 22,260,819	-€ 29,681,093
4058	0.22	€ 339,253	€ 678,506	##########	##########	-€ 8,203,035	-€ 16,406,069	-€ 24,609,104	-€ 32,812,139
4059	0.14	€ 224,655	€ 449,309	€ 673,964	€ 898,618	-€ 5,432,080	-€ 10,864,160	-€ 16,296,239	-€ 21,728,319
4060	0.20	€ 313,354	€ 626,708	€ 940,062	##########	-€ 7,576,807	-€ 15,153,613	-€ 22,730,420	-€ 30,307,226
4061	0.22	€ 340,465	€ 680,929	##########	##########	-€ 8,232,334	-€ 16,464,668	-€ 24,697,002	-€ 32,929,336
4062	0.15	€ 237,074	€ 474,148	€ 711,222	€ 948,296	-€ 5,732,377	-€ 11,464,754	-€ 17,197,131	-€ 22,929,508
4063	0.19	€ 299,820	€ 599,640	€ 899,460	##########	-€ 7,249,559	-€ 14,499,118	-€ 21,748,677	-€ 28,998,236
4064	0.22	€ 353,712	€ 707,423	##########	##########	-€ 8,552,643	-€ 17,105,286	-€ 25,657,928	-€ 34,210,571
4065	0.20	€ 307,709	€ 615,418	€ 923,127	##########	-€ 7,440,312	-€ 14,880,624	-€ 22,320,936	-€ 29,761,248
4066	0.25	€ 389,588	€ 779,177	##########	##########	-€ 9,420,133	-€ 18,840,266	-€ 28,260,398	-€ 37,680,531
4067	0.17	€ 273,434	€ 546,867	€ 820,301	##########	-€ 6,611,541	-€ 13,223,082	-€ 19,834,624	-€ 26,446,165
4068	0.23	€ 362,415	€ 724,830	##########	##########	-€ 8,763,091	-€ 17,526,181	-€ 26,289,272	-€ 35,052,363
4069	0.26	€ 407,799	€ 815,599	##########	##########	-€ 9,860,466	-€ 19,720,932	-€ 29,581,398	-€ 39,441,864
4070	0.20	€ 316,649	€ 633,298	€ 949,946	##########	-€ 7,656,474	-€ 15,312,949	-€ 22,969,423	-€ 30,625,898
4071	0.16	€ 249,243	€ 498,487	€ 747,730	€ 996,973	-€ 6,026,628	-€ 12,053,256	-€ 18,079,884	-€ 24,106,512
4072	0.15	€ 232,767	€ 465,534	€ 698,302	€ 931,069	-€ 5,628,242	-€ 11,256,483	-€ 16,884,725	-€ 22,512,967
4073	0.14	€ 219,601	€ 439,201	€ 658,802	€ 878,402	-€ 5,309,876	-€ 10,619,751	-€ 15,929,627	-€ 21,239,502
4074	0.14	€ 220,200	€ 440,400	€ 660,600	€ 880,800	-€ 5,324,373	-€ 10,648,746	-€ 15,973,119	-€ 21,297,493
4075	0.15	€ 229,339	€ 458,679	€ 688,018	€ 917,358	-€ 5,545,358	-€ 11,090,717	-€ 16,636,075	-€ 22,181,434
4076	0.21	€ 326,222	€ 652,443	€ 978,665	##########	-€ 7,887,944	-€ 15,775,889	-€ 23,663,833	-€ 31,551,778

4077	0.20	€ 308,241	€ 616,482	€ 924,724	##########	-€ 7,453,181	-€ 14,906,361	-€ 22,359,542	-€ 29,812,722
4078	0.19	€ 298,694	€ 597,387	€ 896,081	##########	-€ 7,222,322	-€ 14,444,643	-€ 21,666,965	-€ 28,889,286
4079	0.24	€ 378,638	€ 757,276	##########	##########	-€ 9,155,353	-€ 18,310,706	-€ 27,466,059	-€ 36,621,412
4080	0.15	€ 233,256	€ 466,512	€ 699,768	€ 933,024	-€ 5,640,058	-€ 11,280,116	-€ 16,920,174	-€ 22,560,232
4081	0.19	€ 305,055	€ 610,111	€ 915,166	##########	-€ 7,376,148	-€ 14,752,295	-€ 22,128,443	-€ 29,504,590
4082	0.17	€ 268,712	€ 537,425	€ 806,137	##########	-€ 6,497,383	-€ 12,994,766	-€ 19,492,149	-€ 25,989,532
4083	0.15	€ 243,249	€ 486,498	€ 729,748	€ 972,997	-€ 5,881,693	-€ 11,763,387	-€ 17,645,080	-€ 23,526,773
4084	0.16	€ 256,949	€ 513,898	€ 770,847	##########	-€ 6,212,948	-€ 12,425,896	-€ 18,638,845	-€ 24,851,793
4085	0.17	€ 268,397	€ 536,793	€ 805,190	##########	-€ 6,489,748	-€ 12,979,497	-€ 19,469,245	-€ 25,958,994
4086	0.19	€ 304,418	€ 608,836	€ 913,254	##########	-€ 7,360,737	-€ 14,721,473	-€ 22,082,210	-€ 29,442,947
4087	0.19	€ 305,172	€ 610,345	€ 915,517	##########	-€ 7,378,977	-€ 14,757,955	-€ 22,136,932	-€ 29,515,910
4088	0.23	€ 367,873	€ 735,746	##########	##########	-€ 8,895,062	-€ 17,790,124	-€ 26,685,186	-€ 35,580,248
4089	0.14	€ 217,825	€ 435,651	€ 653,476	€ 871,301	-€ 5,266,952	-€ 10,533,903	-€ 15,800,855	-€ 21,067,806
4090	0.26	€ 413,567	€ 827,134	##########	##########	-€ 9,999,930	-€ 19,999,860	-€ 29,999,790	-€ 39,999,720
4091	0.20	€ 318,618	€ 637,236	€ 955,853	##########	-€ 7,704,084	-€ 15,408,167	-€ 23,112,251	-€ 30,816,334
4092	0.16	€ 247,891	€ 495,782	€ 743,673	€ 991,563	-€ 5,993,927	-€ 11,987,854	-€ 17,981,780	-€ 23,975,707
4093	0.18	€ 288,634	€ 577,269	€ 865,903	##########	-€ 6,979,095	-€ 13,958,190	-€ 20,937,285	-€ 27,916,381
4094	0.15	€ 229,723	€ 459,446	€ 689,168	€ 918,891	-€ 5,554,628	-€ 11,109,256	-€ 16,663,884	-€ 22,218,512
4095	0.17	€ 269,169	€ 538,339	€ 807,508	##########	-€ 6,508,436	-€ 13,016,871	-€ 19,525,307	-€ 26,033,743
4096	0.16	€ 246,454	€ 492,908	€ 739,362	€ 985,816	-€ 5,959,186	-€ 11,918,372	-€ 17,877,558	-€ 23,836,744
4097	0.19	€ 301,259	€ 602,517	€ 903,776	##########	-€ 7,284,345	-€ 14,568,690	-€ 21,853,035	-€ 29,137,380
4098	0.26	€ 411,821	€ 823,642	##########	##########	-€ 9,957,704	-€ 19,915,409	-€ 29,873,113	-€ 39,830,818
4099	0.18	€ 286,691	€ 573,382	€ 860,073	##########	-€ 6,932,107	-€ 13,864,215	-€ 20,796,322	-€ 27,728,429
4100	0.17	€ 267,145	€ 534,290	€ 801,436	##########	-€ 6,459,492	-€ 12,918,985	-€ 19,378,477	-€ 25,837,970
4101	0.23	€ 359,145	€ 718,290	##########	##########	-€ 8,684,025	-€ 17,368,049	-€ 26,052,074	-€ 34,736,099
4102	0.16	€ 247,541	€ 495,082	€ 742,622	€ 990,163	-€ 5,985,463	-€ 11,970,927	-€ 17,956,390	-€ 23,941,854
4103	0.24	€ 373,105	€ 746,210	##########	##########	-€ 9,021,568	-€ 18,043,135	-€ 27,064,703	-€ 36,086,271
4104	0.23	€ 368,427	€ 736,854	##########	##########	-€ 8,908,460	-€ 17,816,921	-€ 26,725,381	-€ 35,633,842
4105	0.21	€ 335,752	€ 671,505	##########	##########	-€ 8,118,393	-€ 16,236,787	-€ 24,355,180	-€ 32,473,573
4106	0.23	€ 357,598	€ 715,197	##########	##########	-€ 8,646,621	-€ 17,293,242	-€ 25,939,863	-€ 34,586,484
4107	0.20	€ 307,979	€ 615,958	€ 923,937	##########	-€ 7,446,844	-€ 14,893,688	-€ 22,340,532	-€ 29,787,377
4108	0.24	€ 374,776	€ 749,553	##########	##########	-€ 9,061,982	-€ 18,123,964	-€ 27,185,946	-€ 36,247,928
4109	0.16	€ 248,018	€ 496,037	€ 744,055	€ 992,073	-€ 5,997,011	-€ 11,994,021	-€ 17,991,032	-€ 23,988,043
4110	0.21	€ 330,024	€ 660,048	€ 990,072	##########	-€ 7,979,882	-€ 15,959,763	-€ 23,939,645	-€ 31,919,526
4111	0.21	€ 331,229	€ 662,458	€ 993,687	##########	-€ 8,009,020	-€ 16,018,041	-€ 24,027,061	-€ 32,036,081
4112	0.21	€ 337,309	€ 674,618	##########	##########	-€ 8,156,027	-€ 16,312,053	-€ 24,468,080	-€ 32,624,106
4113	0.18	€ 286,433	€ 572,867	€ 859,300	##########	-€ 6,925,876	-€ 13,851,751	-€ 20,777,627	-€ 27,703,503
4114	0.27	€ 433,002	€ 866,004	##########	##########	-€ 10,469,862	-€ 20,939,725	-€ 31,409,587	-€ 41,879,449
4115	0.17	€ 273,601	€ 547,201	€ 820,802	##########	-€ 6,615,583	-€ 13,231,166	-€ 19,846,749	-€ 26,462,332
4116	0.21	€ 329,673	€ 659,346	€ 989,019	##########	-€ 7,971,393	-€ 15,942,786	-€ 23,914,179	-€ 31,885,573
4117	0.16	€ 253,034	€ 506,069	€ 759,103	##########	-€ 6,118,293	-€ 12,236,586	-€ 18,354,880	-€ 24,473,173
4118	0.27	€ 422,455	€ 844,910	##########	##########	-€ 10,214,836	-€ 20,429,671	-€ 30,644,507	-€ 40,859,342
4119	0.17	€ 261,050	€ 522,101	€ 783,151	##########	-€ 6,312,122	-€ 12,624,244	-€ 18,936,366	-€ 25,248,488
4120	0.19	€ 304,405	€ 608,810	€ 913,214	##########	-€ 7,360,417	-€ 14,720,834	-€ 22,081,251	-€ 29,441,668
4121	0.21	€ 324,224	€ 648,448	€ 972,672	##########	-€ 7,839,641	-€ 15,679,282	-€ 23,518,923	-€ 31,358,564
4122	0.16	€ 259,467	€ 518,934	€ 778,401	##########	-€ 6,273,832	-€ 12,547,664	-€ 18,821,496	-€ 25,095,328
4123	0.25	€ 394,519	€ 789,039	##########	##########	-€ 9,539,360	-€ 19,078,720	-€ 28,618,080	-€ 38,157,440
4124	0.30	€ 477,950	€ 955,899	##########	##########	-€ 11,556,682	-€ 23,113,364	-€ 34,670,046	-€ 46,226,728
4125	0.15	€ 240,899	€ 481,799	€ 722,698	€ 963,597	-€ 5,824,874	-€ 11,649,748	-€ 17,474,621	-€ 23,299,495
4126	0.23	€ 355,471	€ 710,942	##########	##########	-€ 8,595,186	-€ 17,190,372	-€ 25,785,558	-€ 34,380,744
4127	0.20	€ 308,733	€ 617,467	€ 926,200	##########	-€ 7,465,079	-€ 14,930,158	-€ 22,395,237	-€ 29,860,317
4128	0.24	€ 374,769	€ 749,538	##########	##########	-€ 9,061,807	-€ 18,123,614	-€ 27,185,422	-€ 36,247,229
4129	0.20	€ 307,085	€ 614,171	€ 921,256	##########	-€ 7,425,232	-€ 14,850,464	-€ 22,275,696	-€ 29,700,927
4130	0.23	€ 362,925	€ 725,850	##########	##########	-€ 8,775,423	-€ 17,550,847	-€ 26,326,270	-€ 35,101,693
4131	0.18	€ 290,812	€ 581,625	€ 872,437	##########	-€ 7,031,757	-€ 14,063,514	-€ 21,095,271	-€ 28,127,028
4132	0.25	€ 395,708	€ 791,416	##########	##########	-€ 9,568,107	-€ 19,136,214	-€ 28,704,321	-€ 38,272,427
4133	0.16	€ 257,455	€ 514,909	€ 772,364	##########	-€ 6,225,177	-€ 12,450,354	-€ 18,675,530	-€ 24,900,707
4134	0.20	€ 315,243	€ 630,486	€ 945,729	##########	-€ 7,622,483	-€ 15,244,966	-€ 22,867,449	-€ 30,489,932
4135	0.19	€ 291,329	€ 582,658	€ 873,987	##########	-€ 7,044,252	-€ 14,088,503	-€ 21,132,755	-€ 28,177,006
4136	0.21	€ 330,791	€ 661,583	€ 992,374	##########	-€ 7,998,435	-€ 15,996,870	-€ 23,995,304	-€ 31,993,739
4137	0.16	€ 244,091	€ 488,181	€ 732,272	€ 976,362	-€ 5,902,037	-€ 11,804,075	-€ 17,706,112	-€ 23,608,150
4138	0.22	€ 349,011	€ 698,021	##########	##########	-€ 8,438,976	-€ 16,877,951	-€ 25,316,927	-€ 33,755,903
4139	0.09	€ 148,492	€ 296,985	€ 445,477	€ 593,970	-€ 3,590,504	-€ 7,181,009	-€ 10,771,513	-€ 14,362,017
4140	0.20	€ 307,271	€ 614,541	€ 921,812	##########	-€ 7,429,710	-€ 14,859,420	-€ 22,289,130	-€ 29,718,840
4141	0.16	€ 246,935	€ 493,871	€ 740,806	€ 987,742	-€ 5,970,825	-€ 11,941,650	-€ 17,912,476	-€ 23,883,301
4142	0.14	€ 215,854	€ 431,709	€ 647,563	€ 863,417	-€ 5,219,293	-€ 10,438,587	-€ 15,657,880	-€ 20,877,173
4143	0.18	€ 282,970	€ 565,939	€ 848,909	##########	-€ 6,842,123	-€ 13,684,246	-€ 20,526,369	-€ 27,368,492
4144	0.15	€ 239,446	€ 478,892	€ 718,339	€ 957,785	-€ 5,789,739	-€ 11,579,478	-€ 17,369,216	-€ 23,158,955
4145	0.19	€ 291,525	€ 583,051	€ 874,576	##########	-€ 7,049,000	-€ 14,098,000	-€ 21,147,000	-€ 28,196,000
4146	0.23	€ 357,968	€ 715,936	##########	##########	-€ 8,655,558	-€ 17,311,116	-€ 25,966,673	-€ 34,622,231
4147	0.20	€ 314,395	€ 628,790	€ 943,185	##########	-€ 7,601,979	-€ 15,203,958	-€ 22,805,937	-€ 30,407,917
4148	0.24	€ 372,225	€ 744,450	##########	##########	-€ 9,000,286	-€ 18,000,573	-€ 27,000,859	-€ 36,001,145
4149	0.26	€ 412,591	€ 825,181	##########	##########	-€ 9,976,320	-€ 19,952,641	-€ 29,928,961	-€ 39,905,281
4150	0.19	€ 293,320	€ 586,641	€ 879,961	##########	-€ 7,092,400	-€ 14,184,800	-€ 21,277,199	-€ 28,369,599
4151	0.26	€ 408,559	€ 817,118	##########	##########	-€ 9,878,833	-€ 19,757,666	-€ 29,636,499	-€ 39,515,333
4152	0.16	€ 244,598	€ 489,195	€ 733,793	€ 978,391	-€ 5,914,299	-€ 11,828,599	-€ 17,742,898	-€ 23,657,198
4153	0.16	€ 252,966	€ 505,933	€ 758,899	##########	-€ 6,116,652	-€ 12,233,304	-€ 18,349,957	-€ 24,466,609
4154	0.21	€ 335,605	€ 671,210	##########	##########	-€ 8,114,832	-€ 16,229,664	-€ 24,344,496	-€ 32,459,328
4155	0.18	€ 289,986	€ 579,972	€ 869,958	##########	-€ 7,011,773	-€ 14,023,546	-€ 21,035,320	-€ 28,047,093
4156	0.26	€ 413,147	€ 826,295	##########	##########	-€ 9,989,783	-€ 19,979,566	-€ 29,969,350	-€ 39,959,133
4157	0.24	€ 384,527	€ 769,053	##########	##########	-€ 9,297,737	-€ 18,595,474	-€ 27,893,211	-€ 37,190,949
4158	0.21	€ 330,090	€ 660,181	€ 990,271	##########	-€ 7,981,485	-€ 15,962,970	-€ 23,944,454	-€ 31,925,939
4159	0.17	€ 267,617	€ 535,233	€ 802,850	##########	-€ 6,470,893	-€ 12,941,786	-€ 19,412,679	-€ 25,883,572
4160	0.15	€ 235,200	€ 470,400	€ 705,600	€ 940,800	-€ 5,687,064	-€ 11,374,128	-€ 17,061,192	-€ 22,748,256
4161	0.17	€ 262,863	€ 525,726	€ 788,589	##########	-€ 6,355,947	-€ 12,711,895	-€ 19,067,842	-€ 25,423,790
4162	0.14	€ 213,681	€ 427,362	€ 641,042	€ 854,723	-€ 5,166,738	-€ 10,333,476	-€ 15,500,214	-€ 20,666,953
4163	0.16	€ 254,282	€ 508,564	€ 762,847	##########	-€ 6,148,468	-€ 12,296,936	-€ 18,445,404	-€ 24,593,871
4164	0.13	€ 198,340	€ 396,680	€ 595,020	€ 793,360	-€ 4,795,803	-€ 9,591,605	-€ 14,387,408	-€ 19,183,210
4165	0.20	€ 313,978	€ 627,957	€ 941,935	##########	-€ 7,591,907	-€ 15,183,814	-€ 22,775,721	-€ 30,367,627
4166	0.20	€ 312,123	€ 624,245	€ 936,368	##########	-€ 7,547,033	-€ 15,094,066	-€ 22,641,098	-€ 30,188,131
4167	0.22	€ 342,554	€ 685,107	##########	##########	-€ 8,282,842	-€ 16,565,685	-€ 24,848,527	-€ 33,131,369
4168	0.24	€ 370,659	€ 741,318	##########	##########	-€ 8,962,422	-€ 17,924,844	-€ 26,887,267	-€ 35,849,689
4169	0.17	€ 267,122	€ 534,244	€ 801,366	##########	-€ 6,458,928	-€ 12,917,857	-€ 19,376,785	-€ 25,835,714
4170	0.16	€ 250,271	€ 500,541	€ 750,812	##########	-€ 6,051,472	-€ 12,102,944	-€ 18,154,417	-€ 24,205,889
4171	0.20	€ 317,475	€ 634,950	€ 952,425	##########	-€ 7,676,450	-€ 15,352,901	-€ 23,029,351	-€ 30,705,801
4172	0.14	€ 217,660	€ 435,319	€ 652,979	€ 870,639	-€ 5,262,947	-€ 10,525,894	-€ 15,788,841	-€ 21,051,787
4173	0.15	€ 242,680	€ 485,361	€ 728,041	€ 970,722	-€ 5,867,941	-€ 11,735,882	-€ 17,603,823	-€ 23,471,765
4174	0.15	€ 239,622	€ 479,245	€ 718,867	€ 958,489	-€ 5,793,996	-€ 11,587,992	-€ 17,381,987	-€ 23,175,983
4175	0.17	€ 263,601	€ 527,202	€ 790,803	##########	-€ 6,373,797	-€ 12,747,594	-€ 19,121,391	-€ 25,495,188
4176	0.27	€ 422,782	€ 845,563	##########	##########	-€ 10,222,736	-€ 20,445,473	-€ 30,668,209	-€ 40,890,946
4177	0.15	€ 237,312	€ 474,625	€ 711,937	€ 949,249	-€ 5,738,142	-€ 11,476,285	-€ 17,214,427	-€ 22,952,570
4178	0.23	€ 357,478	€ 714,955	##########	##########	-€ 8,643,703	-€ 17,287,406	-€ 25,931,109	-€ 34,574,812

4179	0.16	€ 256,383	€ 512,766	€ 769,150	##########	-€ 6,199,269	-€ 12,398,538	-€ 18,597,807	-€ 24,797,077
4180	0.25	€ 395,643	€ 791,286	##########	##########	-€ 9,566,528	-€ 19,133,055	-€ 28,699,583	-€ 38,266,110
4181	0.17	€ 274,299	€ 548,597	€ 822,896	##########	-€ 6,632,458	-€ 13,264,916	-€ 19,897,374	-€ 26,529,832
4182	0.11	€ 179,991	€ 359,981	€ 539,972	€ 719,962	-€ 4,352,119	-€ 8,704,239	-€ 13,056,358	-€ 17,408,478
4183	0.15	€ 243,440	€ 486,879	€ 730,319	€ 973,759	-€ 5,886,298	-€ 11,772,596	-€ 17,658,895	-€ 23,545,193
4184	0.15	€ 242,693	€ 485,387	€ 728,080	€ 970,773	-€ 5,868,253	-€ 11,736,505	-€ 17,604,758	-€ 23,473,011
4185	0.17	€ 267,230	€ 534,460	€ 801,691	##########	-€ 6,461,547	-€ 12,923,095	-€ 19,384,642	-€ 25,846,189
4186	0.15	€ 232,011	€ 464,023	€ 696,034	€ 928,046	-€ 5,609,969	-€ 11,219,938	-€ 16,829,907	-€ 22,439,876
4187	0.14	€ 219,552	€ 439,104	€ 658,655	€ 878,207	-€ 5,308,698	-€ 10,617,396	-€ 15,926,094	-€ 21,234,792
4188	0.22	€ 349,626	€ 699,251	##########	##########	-€ 8,453,847	-€ 16,907,693	-€ 25,361,540	-€ 33,815,387
4189	0.20	€ 313,262	€ 626,524	€ 939,785	##########	-€ 7,574,578	-€ 15,149,156	-€ 22,723,734	-€ 30,298,312
4190	0.17	€ 270,727	€ 541,454	€ 812,181	##########	-€ 6,546,095	-€ 13,092,190	-€ 19,638,284	-€ 26,184,379
4191	0.14	€ 225,402	€ 450,804	€ 676,206	€ 901,608	-€ 5,450,156	-€ 10,900,311	-€ 16,350,467	-€ 21,800,623
4192	0.21	€ 333,714	€ 667,429	##########	##########	-€ 8,069,116	-€ 16,138,231	-€ 24,207,347	-€ 32,276,463
4193	0.17	€ 262,418	€ 524,835	€ 787,253	##########	-€ 6,345,179	-€ 12,690,358	-€ 19,035,537	-€ 25,380,716
4194	0.17	€ 266,765	€ 533,531	€ 800,296	##########	-€ 6,450,305	-€ 12,900,609	-€ 19,350,914	-€ 25,801,219
4195	0.19	€ 298,186	€ 596,373	€ 894,559	##########	-€ 7,210,060	-€ 14,420,119	-€ 21,630,179	-€ 28,840,239
4196	0.21	€ 330,341	€ 660,682	€ 991,023	##########	-€ 7,987,547	-€ 15,975,093	-€ 23,962,640	-€ 31,950,186
4197	0.23	€ 354,786	€ 709,573	##########	##########	-€ 8,578,632	-€ 17,157,263	-€ 25,735,895	-€ 34,314,526
4198	0.21	€ 324,196	€ 648,392	€ 972,589	##########	-€ 7,838,967	-€ 15,677,934	-€ 23,516,902	-€ 31,355,869
4199	0.18	€ 283,213	€ 566,426	€ 849,639	##########	-€ 6,848,009	-€ 13,696,018	-€ 20,544,026	-€ 27,392,035
4200	0.14	€ 215,554	€ 431,109	€ 646,663	€ 862,217	-€ 5,212,040	-€ 10,424,080	-€ 15,636,120	-€ 20,848,160
4201	0.14	€ 227,128	€ 454,255	€ 681,383	€ 908,510	-€ 5,491,877	-€ 10,983,754	-€ 16,475,631	-€ 21,967,508
4202	0.19	€ 299,932	€ 599,863	€ 899,795	##########	-€ 7,252,259	-€ 14,504,518	-€ 21,756,777	-€ 29,009,036
4203	0.21	€ 323,022	€ 646,045	€ 969,067	##########	-€ 7,810,585	-€ 15,621,170	-€ 23,431,755	-€ 31,242,340
4204	0.13	€ 211,388	€ 422,776	€ 634,164	€ 845,551	-€ 5,111,296	-€ 10,222,592	-€ 15,333,888	-€ 20,445,184
4205	0.17	€ 263,681	€ 527,362	€ 791,044	##########	-€ 6,375,733	-€ 12,751,466	-€ 19,127,199	-€ 25,502,932
4206	0.17	€ 264,470	€ 528,940	€ 793,409	##########	-€ 6,394,802	-€ 12,789,603	-€ 19,184,405	-€ 25,579,207
4207	0.19	€ 292,669	€ 585,339	€ 878,008	##########	-€ 7,076,661	-€ 14,153,322	-€ 21,229,982	-€ 28,306,643
4208	0.22	€ 353,230	€ 706,460	##########	##########	-€ 8,540,991	-€ 17,081,983	-€ 25,622,974	-€ 34,163,965
4209	0.17	€ 272,257	€ 544,514	€ 816,771	##########	-€ 6,583,094	-€ 13,166,188	-€ 19,749,281	-€ 26,332,375
4210	0.19	€ 294,327	€ 588,654	€ 882,981	##########	-€ 7,116,738	-€ 14,233,476	-€ 21,350,214	-€ 28,466,952
4211	0.24	€ 385,066	€ 770,132	##########	##########	-€ 9,310,785	-€ 18,621,571	-€ 27,932,356	-€ 37,243,142
4212	0.13	€ 198,188	€ 396,376	€ 594,564	€ 792,752	-€ 4,792,127	-€ 9,584,253	-€ 14,376,380	-€ 19,168,507
4213	0.21	€ 326,215	€ 652,430	€ 978,645	##########	-€ 7,887,782	-€ 15,775,564	-€ 23,663,346	-€ 31,551,128
4214	0.17	€ 261,209	€ 522,419	€ 783,628	##########	-€ 6,315,963	-€ 12,631,925	-€ 18,947,888	-€ 25,263,850
4215	0.14	€ 217,912	€ 435,824	€ 653,736	€ 871,648	-€ 5,269,048	-€ 10,538,095	-€ 15,807,143	-€ 21,076,191
4216	0.21	€ 336,810	€ 673,620	##########	##########	-€ 8,143,962	-€ 16,287,923	-€ 24,431,885	-€ 32,575,847
4217	0.20	€ 312,503	€ 625,005	€ 937,508	##########	-€ 7,556,219	-€ 15,112,439	-€ 22,668,658	-€ 30,224,878
4218	0.22	€ 350,516	€ 701,032	##########	##########	-€ 8,475,370	-€ 16,950,741	-€ 25,426,111	-€ 33,901,481
4219	0.19	€ 296,703	€ 593,406	€ 890,109	##########	-€ 7,174,194	-€ 14,348,387	-€ 21,522,581	-€ 28,696,775
4220	0.12	€ 187,934	€ 375,868	€ 563,802	€ 751,736	-€ 4,544,191	-€ 9,088,381	-€ 13,632,572	-€ 18,176,763
4221	0.21	€ 326,865	€ 653,729	€ 980,594	##########	-€ 7,903,487	-€ 15,806,974	-€ 23,710,460	-€ 31,613,947
4222	0.20	€ 314,049	€ 628,097	€ 942,146	##########	-€ 7,593,601	-€ 15,187,203	-€ 22,780,804	-€ 30,374,406
4223	0.16	€ 247,204	€ 494,409	€ 741,613	€ 988,818	-€ 5,977,331	-€ 11,954,663	-€ 17,931,994	-€ 23,909,326
4224	0.18	€ 288,278	€ 576,556	€ 864,834	##########	-€ 6,970,478	-€ 13,940,957	-€ 20,911,435	-€ 27,881,914
4225	0.15	€ 235,978	€ 471,957	€ 707,935	€ 943,914	-€ 5,705,889	-€ 11,411,778	-€ 17,117,668	-€ 22,823,557
4226	0.13	€ 202,637	€ 405,273	€ 607,910	€ 810,546	-€ 4,899,693	-€ 9,799,385	-€ 14,699,078	-€ 19,598,770
4227	0.18	€ 290,337	€ 580,673	€ 871,010	##########	-€ 7,020,251	-€ 14,040,502	-€ 21,060,753	-€ 28,081,004
4228	0.21	€ 325,688	€ 651,377	€ 977,065	##########	-€ 7,875,050	-€ 15,750,099	-€ 23,625,149	-€ 31,500,199
4229	0.14	€ 215,440	€ 430,880	€ 646,320	€ 861,760	-€ 5,209,275	-€ 10,418,551	-€ 15,627,826	-€ 20,837,102
4230	0.24	€ 373,740	€ 747,481	##########	##########	-€ 9,036,930	-€ 18,073,861	-€ 27,110,791	-€ 36,147,722
4231	0.23	€ 363,649	€ 727,298	##########	##########	-€ 8,792,922	-€ 17,585,845	-€ 26,378,767	-€ 35,171,690
4232	0.17	€ 273,361	€ 546,722	€ 820,082	##########	-€ 6,609,784	-€ 13,219,567	-€ 19,829,351	-€ 26,439,135
4233	0.14	€ 226,581	€ 453,162	€ 679,743	€ 906,325	-€ 5,478,665	-€ 10,957,331	-€ 16,435,996	-€ 21,914,661
4234	0.13	€ 197,041	€ 394,081	€ 591,122	€ 788,162	-€ 4,764,382	-€ 9,528,763	-€ 14,293,145	-€ 19,057,527
4235	0.11	€ 169,986	€ 339,972	€ 509,959	€ 679,945	-€ 4,110,216	-€ 8,220,433	-€ 12,330,649	-€ 16,440,866
4236	0.17	€ 274,486	€ 548,971	€ 823,457	##########	-€ 6,636,979	-€ 13,273,957	-€ 19,910,936	-€ 26,547,914
4237	0.12	€ 196,389	€ 392,777	€ 589,166	€ 785,554	-€ 4,748,617	-€ 9,497,235	-€ 14,245,852	-€ 18,994,469
4238	0.14	€ 217,805	€ 435,611	€ 653,416	€ 871,221	-€ 5,266,469	-€ 10,532,938	-€ 15,799,407	-€ 21,065,876
4239	0.25	€ 388,805	€ 777,610	##########	##########	-€ 9,401,192	-€ 18,802,383	-€ 28,203,575	-€ 37,604,767
4240	0.19	€ 301,292	€ 602,584	€ 903,875	##########	-€ 7,285,146	-€ 14,570,293	-€ 21,855,439	-€ 29,140,585
4241	0.14	€ 216,715	€ 433,430	€ 650,145	€ 866,860	-€ 5,240,106	-€ 10,480,212	-€ 15,720,319	-€ 20,960,425
4242	0.24	€ 380,869	€ 761,739	##########	##########	-€ 9,209,309	-€ 18,418,617	-€ 27,627,926	-€ 36,837,234
4243	0.24	€ 378,948	€ 757,896	##########	##########	-€ 9,162,853	-€ 18,325,706	-€ 27,488,559	-€ 36,651,413
4244	0.20	€ 308,906	€ 617,811	€ 926,717	##########	-€ 7,469,249	-€ 14,938,498	-€ 22,407,747	-€ 29,876,996
4245	0.25	€ 392,610	€ 785,219	##########	##########	-€ 9,493,186	-€ 18,986,371	-€ 28,479,557	-€ 37,972,742
4246	0.19	€ 295,063	€ 590,127	€ 885,190	##########	-€ 7,134,546	-€ 14,269,093	-€ 21,403,639	-€ 28,538,185
4247	0.19	€ 298,318	€ 596,636	€ 894,954	##########	-€ 7,213,241	-€ 14,426,481	-€ 21,639,722	-€ 28,852,963
4248	0.09	€ 141,623	€ 283,246	€ 424,869	€ 566,492	-€ 3,424,402	-€ 6,848,804	-€ 10,273,206	-€ 13,697,608
4249	0.14	€ 223,769	€ 447,539	€ 671,308	€ 895,078	-€ 5,410,680	-€ 10,821,359	-€ 16,232,039	-€ 21,642,718
4250	0.20	€ 319,942	€ 639,884	€ 959,826	##########	-€ 7,736,106	-€ 15,472,212	-€ 23,208,318	-€ 30,944,424
4251	0.11	€ 175,629	€ 351,257	€ 526,886	€ 702,514	-€ 4,246,646	-€ 8,493,293	-€ 12,739,939	-€ 16,986,586
4252	0.18	€ 281,603	€ 563,205	€ 844,808	##########	-€ 6,809,069	-€ 13,618,138	-€ 20,427,207	-€ 27,236,276
4253	0.23	€ 361,915	€ 723,830	##########	##########	-€ 8,750,993	-€ 17,501,985	-€ 26,252,978	-€ 35,003,971
4254	0.15	€ 234,704	€ 469,408	€ 704,112	€ 938,816	-€ 5,675,074	-€ 11,350,148	-€ 17,025,223	-€ 22,700,297
4255	0.19	€ 298,840	€ 597,679	€ 896,519	##########	-€ 7,225,853	-€ 14,451,706	-€ 21,677,559	-€ 28,903,411
4256	0.19	€ 303,704	€ 607,407	€ 911,111	##########	-€ 7,343,461	-€ 14,686,921	-€ 22,030,382	-€ 29,373,843
4257	0.20	€ 318,554	€ 637,108	€ 955,663	##########	-€ 7,702,546	-€ 15,405,092	-€ 23,107,638	-€ 30,810,184
4258	0.17	€ 268,436	€ 536,872	€ 805,308	##########	-€ 6,490,704	-€ 12,981,407	-€ 19,472,111	-€ 25,962,815
4259	0.27	€ 420,560	€ 841,121	##########	##########	-€ 10,169,024	-€ 20,338,047	-€ 30,507,071	-€ 40,676,095
4260	0.16	€ 244,287	€ 488,573	€ 732,860	€ 977,146	-€ 5,906,776	-€ 11,813,553	-€ 17,720,329	-€ 23,627,106
4261	0.10	€ 156,499	€ 312,999	€ 469,498	€ 625,998	-€ 3,784,110	-€ 7,568,221	-€ 11,352,331	-€ 15,136,441
4262	0.22	€ 353,997	€ 707,994	##########	##########	-€ 8,559,541	-€ 17,119,082	-€ 25,678,623	-€ 34,238,164
4263	0.21	€ 338,198	€ 676,396	##########	##########	-€ 8,177,522	-€ 16,355,045	-€ 24,532,567	-€ 32,710,090
4264	0.15	€ 239,785	€ 479,571	€ 719,356	€ 959,141	-€ 5,797,939	-€ 11,595,877	-€ 17,393,816	-€ 23,191,754
4265	0.12	€ 182,784	€ 365,568	€ 548,352	€ 731,137	-€ 4,419,666	-€ 8,839,332	-€ 13,258,999	-€ 17,678,665
4266	0.20	€ 311,790	€ 623,579	€ 935,369	##########	-€ 7,538,980	-€ 15,077,960	-€ 22,616,940	-€ 30,155,920
4267	0.15	€ 239,594	€ 479,188	€ 718,782	€ 958,377	-€ 5,793,315	-€ 11,586,630	-€ 17,379,945	-€ 23,173,260
4268	0.22	€ 340,623	€ 681,245	##########	##########	-€ 8,236,156	-€ 16,472,312	-€ 24,708,468	-€ 32,944,624
4269	0.14	€ 226,579	€ 453,158	€ 679,737	€ 906,315	-€ 5,478,609	-€ 10,957,219	-€ 16,435,828	-€ 21,914,437
4270	0.19	€ 293,377	€ 586,754	€ 880,131	##########	-€ 7,093,772	-€ 14,187,543	-€ 21,281,315	-€ 28,375,086
4271	0.18	€ 283,786	€ 567,571	€ 851,357	##########	-€ 6,861,851	-€ 13,723,702	-€ 20,585,553	-€ 27,447,405
4272	0.22	€ 344,313	€ 688,625	##########	##########	-€ 8,325,376	-€ 16,650,753	-€ 24,976,129	-€ 33,301,506
4273	0.17	€ 274,171	€ 548,341	€ 822,512	##########	-€ 6,629,366	-€ 13,258,732	-€ 19,888,098	-€ 26,517,465
4274	0.24	€ 373,206	€ 746,412	##########	##########	-€ 9,024,011	-€ 18,048,022	-€ 27,072,033	-€ 36,096,044
4275	0.20	€ 318,335	€ 636,670	€ 955,006	##########	-€ 7,697,250	-€ 15,394,500	-€ 23,091,750	-€ 30,789,000
4276	0.19	€ 297,461	€ 594,922	€ 892,383	##########	-€ 7,192,517	-€ 14,385,034	-€ 21,577,550	-€ 28,770,067
4277	0.19	€ 294,262	€ 588,524	€ 882,786	##########	-€ 7,115,168	-€ 14,230,336	-€ 21,345,503	-€ 28,460,671
4278	0.21	€ 330,188	€ 660,376	€ 990,564	##########	-€ 7,983,846	-€ 15,967,692	-€ 23,951,538	-€ 31,935,384
4279	0.18	€ 275,800	€ 551,600	€ 827,400	##########	-€ 6,668,765	-€ 13,337,531	-€ 20,006,296	-€ 26,675,061
4280	0.22	€ 339,020	€ 678,040	##########	##########	-€ 8,197,401	-€ 16,394,801	-€ 24,592,202	-€ 32,789,602

4281	0.12	€ 192,801	€ 385,602	€ 578,404	€ 771,205	-€ 4,661,876	-€ 9,323,753	-€ 13,985,629	-€ 18,647,505
4282	0.12	€ 194,999	€ 389,999	€ 584,998	€ 779,998	-€ 4,715,028	-€ 9,430,055	-€ 14,145,083	-€ 18,860,111
4283	0.12	€ 189,882	€ 379,763	€ 569,645	€ 759,527	-€ 4,591,283	-€ 9,182,566	-€ 13,773,848	-€ 18,365,131
4284	0.11	€ 170,534	€ 341,067	€ 511,601	€ 682,134	-€ 4,123,451	-€ 8,246,902	-€ 12,370,353	-€ 16,493,804
4285	0.18	€ 289,256	€ 578,513	€ 867,769	##########	-€ 6,994,136	-€ 13,988,272	-€ 20,982,409	-€ 27,976,545
4286	0.19	€ 301,104	€ 602,208	€ 903,312	##########	-€ 7,280,608	-€ 14,561,216	-€ 21,841,824	-€ 29,122,432
4287	0.20	€ 322,148	€ 644,296	€ 966,443	##########	-€ 7,789,437	-€ 15,578,875	-€ 23,368,312	-€ 31,157,749
4288	0.18	€ 278,102	€ 556,205	€ 834,307	##########	-€ 6,724,435	-€ 13,448,870	-€ 20,173,306	-€ 26,897,741
4289	0.15	€ 230,002	€ 460,004	€ 690,007	€ 920,009	-€ 5,561,385	-€ 11,122,770	-€ 16,684,156	-€ 22,245,541
4290	0.27	€ 421,943	€ 843,885	##########	##########	-€ 10,202,450	-€ 20,404,900	-€ 30,607,351	-€ 40,809,801
4291	0.16	€ 246,802	€ 493,604	€ 740,407	€ 987,209	-€ 5,967,605	-€ 11,935,209	-€ 17,902,814	-€ 23,870,419
4292	0.18	€ 277,319	€ 554,639	€ 831,958	##########	-€ 6,705,503	-€ 13,411,005	-€ 20,116,508	-€ 26,822,010
4293	0.15	€ 229,258	€ 458,516	€ 687,775	€ 917,033	-€ 5,543,396	-€ 11,086,792	-€ 16,630,188	-€ 22,173,584
4294	0.22	€ 346,397	€ 692,794	##########	##########	-€ 8,375,777	-€ 16,751,554	-€ 25,127,331	-€ 33,503,109
4295	0.21	€ 330,527	€ 661,054	€ 991,580	##########	-€ 7,992,039	-€ 15,984,078	-€ 23,976,117	-€ 31,968,156
4296	0.22	€ 353,898	€ 707,796	##########	##########	-€ 8,557,145	-€ 17,114,289	-€ 25,671,434	-€ 34,228,578
4297	0.16	€ 253,176	€ 506,351	€ 759,527	##########	-€ 6,121,710	-€ 12,243,421	-€ 18,365,131	-€ 24,486,842
4298	0.22	€ 339,310	€ 678,620	##########	##########	-€ 8,204,420	-€ 16,408,840	-€ 24,613,260	-€ 32,817,680
4299	0.14	€ 222,031	€ 444,061	€ 666,092	€ 888,123	-€ 5,368,637	-€ 10,737,274	-€ 16,105,911	-€ 21,474,548
4300	0.20	€ 310,364	€ 620,729	€ 931,093	##########	-€ 7,504,521	-€ 15,009,041	-€ 22,513,562	-€ 30,018,082
4301	0.23	€ 365,543	€ 731,087	##########	##########	-€ 8,838,730	-€ 17,677,460	-€ 26,516,191	-€ 35,354,921
4302	0.18	€ 286,054	€ 572,109	€ 858,163	##########	-€ 6,916,709	-€ 13,833,418	-€ 20,750,128	-€ 27,666,837
4303	0.19	€ 294,879	€ 589,758	€ 884,637	##########	-€ 7,130,086	-€ 14,260,171	-€ 21,390,257	-€ 28,520,342
4304	0.17	€ 265,123	€ 530,246	€ 795,368	##########	-€ 6,410,590	-€ 12,821,180	-€ 19,231,769	-€ 25,642,359
4305	0.10	€ 158,721	€ 317,442	€ 476,163	€ 634,885	-€ 3,837,830	-€ 7,675,661	-€ 11,513,491	-€ 15,351,321
4306	0.18	€ 280,689	€ 561,377	€ 842,066	##########	-€ 6,786,970	-€ 13,573,939	-€ 20,360,909	-€ 27,147,878
4307	0.24	€ 380,638	€ 761,277	##########	##########	-€ 9,203,724	-€ 18,407,448	-€ 27,611,172	-€ 36,814,896
4308	0.16	€ 245,703	€ 491,406	€ 737,109	€ 982,812	-€ 5,941,028	-€ 11,882,057	-€ 17,823,085	-€ 23,764,114
4309	0.22	€ 354,099	€ 708,199	##########	##########	-€ 8,562,021	-€ 17,124,041	-€ 25,686,062	-€ 34,248,083
4310	0.24	€ 370,754	€ 741,509	##########	##########	-€ 8,964,732	-€ 17,929,464	-€ 26,894,196	-€ 35,858,928
4311	0.19	€ 298,173	€ 596,347	€ 894,520	##########	-€ 7,209,747	-€ 14,419,493	-€ 21,629,240	-€ 28,838,987
4312	0.24	€ 383,687	€ 767,374	##########	##########	-€ 9,277,433	-€ 18,554,867	-€ 27,832,300	-€ 37,109,734
4313	0.28	€ 437,016	€ 874,032	##########	##########	-€ 10,566,919	-€ 21,133,839	-€ 31,700,758	-€ 42,267,678
4314	0.17	€ 271,827	€ 543,653	€ 815,480	##########	-€ 6,572,688	-€ 13,145,376	-€ 19,718,065	-€ 26,290,753
4315	0.21	€ 335,031	€ 670,063	##########	##########	-€ 8,100,959	-€ 16,201,917	-€ 24,302,876	-€ 32,403,835
4316	0.21	€ 328,375	€ 656,751	€ 985,126	##########	-€ 7,940,018	-€ 15,880,036	-€ 23,820,054	-€ 31,760,072
4317	0.17	€ 272,817	€ 545,633	€ 818,450	##########	-€ 6,596,626	-€ 13,193,251	-€ 19,789,877	-€ 26,386,502
4318	0.18	€ 287,157	€ 574,314	€ 861,470	##########	-€ 6,943,366	-€ 13,886,732	-€ 20,830,099	-€ 27,773,465
4319	0.14	€ 220,946	€ 441,892	€ 662,838	€ 883,784	-€ 5,342,411	-€ 10,684,823	-€ 16,027,234	-€ 21,369,645
4320	0.17	€ 272,194	€ 544,389	€ 816,583	##########	-€ 6,581,580	-€ 13,163,160	-€ 19,744,741	-€ 26,326,321
4321	0.18	€ 277,794	€ 555,587	€ 833,381	##########	-€ 6,716,965	-€ 13,433,931	-€ 20,150,896	-€ 26,867,862
4322	0.17	€ 264,316	€ 528,633	€ 792,949	##########	-€ 6,391,094	-€ 12,782,188	-€ 19,173,283	-€ 25,564,377
4323	0.16	€ 246,527	€ 493,053	€ 739,580	€ 986,106	-€ 5,960,938	-€ 11,921,877	-€ 17,882,815	-€ 23,843,754
4324	0.23	€ 361,553	€ 723,106	##########	##########	-€ 8,742,243	-€ 17,484,487	-€ 26,226,730	-€ 34,968,974
4325	0.19	€ 302,577	€ 605,154	€ 907,731	##########	-€ 7,316,221	-€ 14,632,442	-€ 21,948,663	-€ 29,264,884
4326	0.14	€ 214,351	€ 428,702	€ 643,053	€ 857,404	-€ 5,182,946	-€ 10,365,891	-€ 15,548,837	-€ 20,731,782
4327	0.16	€ 248,950	€ 497,900	€ 746,850	€ 995,800	-€ 6,019,539	-€ 12,039,078	-€ 18,058,617	-€ 24,078,156
4328	0.21	€ 326,651	€ 653,303	€ 979,954	##########	-€ 7,898,336	-€ 15,796,671	-€ 23,695,007	-€ 31,593,343
4329	0.14	€ 224,065	€ 448,130	€ 672,194	€ 896,259	-€ 5,417,821	-€ 10,835,642	-€ 16,253,463	-€ 21,671,284
4330	0.16	€ 252,788	€ 505,577	€ 758,365	##########	-€ 6,112,347	-€ 12,224,695	-€ 18,337,042	-€ 24,449,389
4331	0.17	€ 265,407	€ 530,814	€ 796,221	##########	-€ 6,417,462	-€ 12,834,923	-€ 19,252,385	-€ 25,669,846
4332	0.20	€ 308,305	€ 616,610	€ 924,915	##########	-€ 7,454,724	-€ 14,909,448	-€ 22,364,173	-€ 29,818,897
4333	0.18	€ 283,038	€ 566,076	€ 849,113	##########	-€ 6,843,770	-€ 13,687,539	-€ 20,531,309	-€ 27,375,079
4334	0.19	€ 306,810	€ 613,620	€ 920,431	##########	-€ 7,418,580	-€ 14,837,160	-€ 22,255,740	-€ 29,674,320
4335	0.09	€ 145,414	€ 290,829	€ 436,243	€ 581,657	-€ 3,516,075	-€ 7,032,150	-€ 10,548,225	-€ 14,064,301
4336	0.20	€ 307,873	€ 615,747	€ 923,620	##########	-€ 7,444,286	-€ 14,888,571	-€ 22,332,857	-€ 29,777,143
4337	0.20	€ 312,890	€ 625,780	€ 938,670	##########	-€ 7,565,585	-€ 15,131,171	-€ 22,696,756	-€ 30,262,341
4338	0.24	€ 374,736	€ 749,472	##########	##########	-€ 9,061,006	-€ 18,122,011	-€ 27,183,017	-€ 36,244,023
4339	0.15	€ 228,959	€ 457,917	€ 686,876	€ 915,835	-€ 5,536,152	-€ 11,072,304	-€ 16,608,456	-€ 22,144,608
4340	0.15	€ 241,785	€ 483,571	€ 725,356	€ 967,141	-€ 5,846,296	-€ 11,692,593	-€ 17,538,889	-€ 23,385,185
4341	0.16	€ 245,140	€ 490,280	€ 735,419	€ 980,559	-€ 5,927,407	-€ 11,854,813	-€ 17,782,220	-€ 23,709,627
4342	0.19	€ 300,459	€ 600,918	€ 901,377	##########	-€ 7,265,007	-€ 14,530,014	-€ 21,795,020	-€ 29,060,027
4343	0.19	€ 295,966	€ 591,932	€ 887,899	##########	-€ 7,156,375	-€ 14,312,750	-€ 21,469,125	-€ 28,625,500
4344	0.22	€ 348,895	€ 697,791	##########	##########	-€ 8,436,186	-€ 16,872,373	-€ 25,308,559	-€ 33,744,745
4345	0.21	€ 331,895	€ 663,790	€ 995,685	##########	-€ 8,025,126	-€ 16,050,252	-€ 24,075,378	-€ 32,100,505
4346	0.19	€ 292,527	€ 585,053	€ 877,580	##########	-€ 7,073,208	-€ 14,146,415	-€ 21,219,623	-€ 28,292,830
4347	0.17	€ 271,363	€ 542,727	€ 814,090	##########	-€ 6,561,485	-€ 13,122,970	-€ 19,684,454	-€ 26,245,939
4348	0.13	€ 204,813	€ 409,626	€ 614,438	€ 819,251	-€ 4,952,313	-€ 9,904,626	-€ 14,856,939	-€ 19,809,251
4349	0.16	€ 255,020	€ 510,039	€ 765,059	##########	-€ 6,166,300	-€ 12,332,599	-€ 18,498,899	-€ 24,665,199
4350	0.20	€ 315,465	€ 630,930	€ 946,394	##########	-€ 7,627,844	-€ 15,255,689	-€ 22,883,533	-€ 30,511,377
4351	0.19	€ 298,534	€ 597,068	€ 895,602	##########	-€ 7,218,464	-€ 14,436,928	-€ 21,655,393	-€ 28,873,857
4352	0.21	€ 331,742	€ 663,485	€ 995,227	##########	-€ 8,021,434	-€ 16,042,867	-€ 24,064,301	-€ 32,085,734
4353	0.15	€ 243,245	€ 486,489	€ 729,734	€ 972,979	-€ 5,881,585	-€ 11,763,169	-€ 17,644,754	-€ 23,526,339
4354	0.22	€ 341,701	€ 683,403	##########	##########	-€ 8,262,235	-€ 16,524,470	-€ 24,786,705	-€ 33,048,940
4355	0.09	€ 138,198	€ 276,395	€ 414,593	€ 552,791	-€ 3,341,579	-€ 6,683,158	-€ 10,024,738	-€ 13,366,317
4356	0.23	€ 357,623	€ 715,247	##########	##########	-€ 8,647,226	-€ 17,294,451	-€ 25,941,677	-€ 34,588,902
4357	0.14	€ 227,419	€ 454,838	€ 682,256	€ 909,675	-€ 5,498,918	-€ 10,997,837	-€ 16,496,755	-€ 21,995,674
4358	0.15	€ 240,784	€ 481,568	€ 722,352	€ 963,136	-€ 5,822,085	-€ 11,644,170	-€ 17,466,255	-€ 23,288,339
4359	0.23	€ 368,395	€ 736,791	##########	##########	-€ 8,907,690	-€ 17,815,381	-€ 26,723,071	-€ 35,630,761
4360	0.14	€ 220,480	€ 440,960	€ 661,441	€ 881,921	-€ 5,331,146	-€ 10,662,291	-€ 15,993,437	-€ 21,324,582
4361	0.19	€ 300,758	€ 601,516	€ 902,273	##########	-€ 7,272,235	-€ 14,544,470	-€ 21,816,705	-€ 29,088,940
4362	0.15	€ 238,142	€ 476,284	€ 714,426	€ 952,568	-€ 5,758,204	-€ 11,516,408	-€ 17,274,612	-€ 23,032,816
4363	0.16	€ 255,473	€ 510,946	€ 766,419	##########	-€ 6,177,259	-€ 12,354,519	-€ 18,531,778	-€ 24,709,037
4364	0.19	€ 306,749	€ 613,497	€ 920,246	##########	-€ 7,417,092	-€ 14,834,185	-€ 22,251,277	-€ 29,668,369
4365	0.20	€ 321,113	€ 642,227	€ 963,340	##########	-€ 7,764,424	-€ 15,528,848	-€ 23,293,273	-€ 31,057,697
4366	0.16	€ 253,687	€ 507,374	€ 761,061	##########	-€ 6,134,075	-€ 12,268,151	-€ 18,402,226	-€ 24,536,301
4367	0.22	€ 349,828	€ 699,656	##########	##########	-€ 8,458,738	-€ 16,917,476	-€ 25,376,214	-€ 33,834,952
4368	0.18	€ 284,534	€ 569,069	€ 853,603	##########	-€ 6,879,958	-€ 13,759,915	-€ 20,639,873	-€ 27,519,830
4369	0.21	€ 323,245	€ 646,490	€ 969,735	##########	-€ 7,815,965	-€ 15,631,931	-€ 23,447,896	-€ 31,263,862
4370	0.22	€ 353,740	€ 707,479	##########	##########	-€ 8,553,319	-€ 17,106,637	-€ 25,659,956	-€ 34,213,274
4371	0.15	€ 229,092	€ 458,184	€ 687,275	€ 916,367	-€ 5,539,371	-€ 11,078,741	-€ 16,618,112	-€ 22,157,482
4372	0.13	€ 204,077	€ 408,154	€ 612,231	€ 816,308	-€ 4,934,518	-€ 9,869,037	-€ 14,803,555	-€ 19,738,074
4373	0.21	€ 335,287	€ 670,573	##########	##########	-€ 8,107,132	-€ 16,214,264	-€ 24,321,397	-€ 32,428,529
4374	0.19	€ 297,561	€ 595,121	€ 892,682	##########	-€ 7,194,929	-€ 14,389,859	-€ 21,584,788	-€ 28,779,717
4375	0.16	€ 254,342	€ 508,683	€ 763,025	##########	-€ 6,149,908	-€ 12,299,815	-€ 18,449,723	-€ 24,599,631
4376	0.25	€ 387,303	€ 774,606	##########	##########	-€ 9,364,869	-€ 18,729,739	-€ 28,094,608	-€ 37,459,478
4377	0.19	€ 292,540	€ 585,080	€ 877,620	##########	-€ 7,073,529	-€ 14,147,058	-€ 21,220,586	-€ 28,294,115
4378	0.22	€ 353,165	€ 706,330	##########	##########	-€ 8,539,429	-€ 17,078,859	-€ 25,618,288	-€ 34,157,718
4379	0.15	€ 237,681	€ 475,363	€ 713,044	€ 950,726	-€ 5,747,066	-€ 11,494,133	-€ 17,241,199	-€ 22,988,266
4380	0.26	€ 416,725	€ 833,451	##########	##########	-€ 10,076,294	-€ 20,152,588	-€ 30,228,883	-€ 40,305,177
4381	0.22	€ 342,744	€ 685,487	##########	##########	-€ 8,287,436	-€ 16,574,872	-€ 24,862,308	-€ 33,149,744
4382	0.21	€ 333,671	€ 667,342	##########	##########	-€ 8,068,062	-€ 16,136,124	-€ 24,204,186	-€ 32,272,248

4383	0.23	€ 368,284	€ 736,569	##########	##########	-€ 8,905,006	-€ 17,810,011	-€ 26,715,017	-€ 35,620,022
4384	0.21	€ 336,778	€ 673,556	##########	##########	-€ 8,143,187	-€ 16,286,375	-€ 24,429,562	-€ 32,572,750
4385	0.15	€ 232,843	€ 465,686	€ 698,530	€ 931,373	-€ 5,630,080	-€ 11,260,161	-€ 16,890,241	-€ 22,520,322
4386	0.20	€ 316,555	€ 633,109	€ 949,664	##########	-€ 7,654,196	-€ 15,308,391	-€ 22,962,587	-€ 30,616,783
4387	0.13	€ 201,036	€ 402,072	€ 603,108	€ 804,144	-€ 4,860,990	-€ 9,721,981	-€ 14,582,971	-€ 19,443,961
4388	0.19	€ 299,993	€ 599,985	€ 899,978	##########	-€ 7,253,733	-€ 14,507,465	-€ 21,761,198	-€ 29,014,931
4389	0.18	€ 288,040	€ 576,079	€ 864,119	##########	-€ 6,964,711	-€ 13,929,422	-€ 20,894,133	-€ 27,858,844
4390	0.25	€ 392,058	€ 784,117	##########	##########	-€ 9,479,858	-€ 18,959,715	-€ 28,439,573	-€ 37,919,430
4391	0.14	€ 219,506	€ 439,011	€ 658,517	€ 878,023	-€ 5,307,582	-€ 10,615,163	-€ 15,922,745	-€ 21,230,327
4392	0.23	€ 355,373	€ 710,746	##########	##########	-€ 8,592,820	-€ 17,185,639	-€ 25,778,459	-€ 34,371,279
4393	0.20	€ 318,989	€ 637,978	€ 956,967	##########	-€ 7,713,056	-€ 15,426,113	-€ 23,139,169	-€ 30,852,225
4394	0.18	€ 281,152	€ 562,304	€ 843,455	##########	-€ 6,798,167	-€ 13,596,335	-€ 20,394,502	-€ 27,192,670
4395	0.29	€ 461,574	€ 923,148	##########	##########	-€ 11,160,724	-€ 22,321,448	-€ 33,482,173	-€ 44,642,897
4396	0.17	€ 273,947	€ 547,894	€ 821,841	##########	-€ 6,623,956	-€ 13,247,912	-€ 19,871,868	-€ 26,495,824
4397	0.16	€ 249,277	€ 498,553	€ 747,830	€ 997,106	-€ 6,027,433	-€ 12,054,865	-€ 18,082,298	-€ 24,109,731
4398	0.17	€ 274,456	€ 548,912	€ 823,369	##########	-€ 6,636,269	-€ 13,272,538	-€ 19,908,807	-€ 26,545,076
4399	0.17	€ 261,197	€ 522,395	€ 783,592	##########	-€ 6,315,678	-€ 12,631,355	-€ 18,947,033	-€ 25,262,710
4400	0.25	€ 388,507	€ 777,013	##########	##########	-€ 9,393,972	-€ 18,787,944	-€ 28,181,916	-€ 37,575,888
4401	0.18	€ 280,945	€ 561,890	€ 842,835	##########	-€ 6,793,165	-€ 13,586,329	-€ 20,379,494	-€ 27,172,658
4402	0.25	€ 389,634	€ 779,268	##########	##########	-€ 9,421,232	-€ 18,842,465	-€ 28,263,697	-€ 37,684,929
4403	0.17	€ 260,811	€ 521,622	€ 782,434	##########	-€ 6,306,338	-€ 12,612,676	-€ 18,919,013	-€ 25,225,351
4404	0.14	€ 219,327	€ 438,654	€ 657,981	€ 877,308	-€ 5,303,264	-€ 10,606,527	-€ 15,909,791	-€ 21,213,054
4405	0.19	€ 292,988	€ 585,976	€ 878,964	##########	-€ 7,084,360	-€ 14,168,719	-€ 21,253,079	-€ 28,337,438
4406	0.19	€ 304,104	€ 608,209	€ 912,313	##########	-€ 7,353,151	-€ 14,706,302	-€ 22,059,453	-€ 29,412,604
4407	0.13	€ 209,961	€ 419,921	€ 629,882	€ 839,843	-€ 5,076,788	-€ 10,153,577	-€ 15,230,365	-€ 20,307,153
4408	0.18	€ 286,388	€ 572,775	€ 859,163	##########	-€ 6,924,769	-€ 13,849,539	-€ 20,774,308	-€ 27,699,078
4409	0.23	€ 356,400	€ 712,800	##########	##########	-€ 8,617,646	-€ 17,235,291	-€ 25,852,937	-€ 34,470,583
4410	0.13	€ 207,540	€ 415,080	€ 622,621	€ 830,161	-€ 5,018,260	-€ 10,036,520	-€ 15,054,779	-€ 20,073,039
4411	0.21	€ 323,345	€ 646,690	€ 970,034	##########	-€ 7,818,382	-€ 15,636,763	-€ 23,455,145	-€ 31,273,527
4412	0.17	€ 267,966	€ 535,932	€ 803,898	##########	-€ 6,479,337	-€ 12,958,674	-€ 19,438,011	-€ 25,917,348
4413	0.21	€ 333,548	€ 667,097	##########	##########	-€ 8,065,099	-€ 16,130,198	-€ 24,195,298	-€ 32,260,397
4414	0.16	€ 254,412	€ 508,825	€ 763,237	##########	-€ 6,151,616	-€ 12,303,231	-€ 18,454,847	-€ 24,606,462
4415	0.18	€ 288,683	€ 577,366	€ 866,050	##########	-€ 6,980,275	-€ 13,960,550	-€ 20,940,826	-€ 27,921,101
4416	0.15	€ 243,776	€ 487,552	€ 731,329	€ 975,105	-€ 5,894,435	-€ 11,788,871	-€ 17,683,306	-€ 23,577,742
4417	0.17	€ 269,829	€ 539,659	€ 809,488	##########	-€ 6,524,392	-€ 13,048,785	-€ 19,573,177	-€ 26,097,569
4418	0.15	€ 233,687	€ 467,373	€ 701,060	€ 934,747	-€ 5,650,474	-€ 11,300,948	-€ 16,951,422	-€ 22,601,896
4419	0.17	€ 265,938	€ 531,877	€ 797,815	##########	-€ 6,430,311	-€ 12,860,621	-€ 19,290,932	-€ 25,721,242
4420	0.28	€ 437,708	€ 875,416	##########	##########	-€ 10,583,651	-€ 21,167,303	-€ 31,750,954	-€ 42,334,605
4421	0.20	€ 318,215	€ 636,430	€ 954,645	##########	-€ 7,694,344	-€ 15,388,689	-€ 23,083,033	-€ 30,777,377
4422	0.19	€ 305,850	€ 611,701	€ 917,551	##########	-€ 7,395,371	-€ 14,790,742	-€ 22,186,113	-€ 29,581,484
4423	0.26	€ 409,221	€ 818,443	##########	##########	-€ 9,894,852	-€ 19,789,704	-€ 29,684,556	-€ 39,579,409
4424	0.22	€ 344,669	€ 689,338	##########	##########	-€ 8,333,993	-€ 16,667,987	-€ 25,001,980	-€ 33,335,974
4425	0.29	€ 455,272	€ 910,544	##########	##########	-€ 11,008,345	-€ 22,016,691	-€ 33,025,036	-€ 44,033,381
4426	0.18	€ 286,152	€ 572,304	€ 858,456	##########	-€ 6,919,070	-€ 13,838,140	-€ 20,757,210	-€ 27,676,280
4427	0.14	€ 221,401	€ 442,801	€ 664,202	€ 885,603	-€ 5,353,403	-€ 10,706,806	-€ 16,060,209	-€ 21,413,612
4428	0.21	€ 330,728	€ 661,456	€ 992,184	##########	-€ 7,996,905	-€ 15,993,811	-€ 23,990,716	-€ 31,987,622
4429	0.18	€ 285,632	€ 571,264	€ 856,896	##########	-€ 6,906,498	-€ 13,812,996	-€ 20,719,493	-€ 27,625,991
4430	0.22	€ 345,728	€ 691,456	##########	##########	-€ 8,359,599	-€ 16,719,199	-€ 25,078,798	-€ 33,438,397
4431	0.18	€ 286,733	€ 573,466	€ 860,200	##########	-€ 6,933,125	-€ 13,866,249	-€ 20,799,374	-€ 27,732,498
4432	0.21	€ 329,205	€ 658,410	€ 987,616	##########	-€ 7,960,085	-€ 15,920,170	-€ 23,880,255	-€ 31,840,340
4433	0.27	€ 428,155	€ 856,310	##########	##########	-€ 10,352,665	-€ 20,705,330	-€ 31,057,995	-€ 41,410,660
4434	0.15	€ 243,183	€ 486,365	€ 729,548	€ 972,730	-€ 5,880,082	-€ 11,760,165	-€ 17,640,247	-€ 23,520,329
4435	0.25	€ 398,111	€ 796,223	##########	##########	-€ 9,626,216	-€ 19,252,433	-€ 28,878,649	-€ 38,504,865
4436	0.17	€ 263,938	€ 527,876	€ 791,814	##########	-€ 6,381,940	-€ 12,763,880	-€ 19,145,820	-€ 25,527,760
4437	0.19	€ 300,544	€ 601,088	€ 901,632	##########	-€ 7,267,067	-€ 14,534,135	-€ 21,801,202	-€ 29,068,269
4438	0.30	€ 467,911	€ 935,823	##########	##########	-€ 11,313,957	-€ 22,627,915	-€ 33,941,872	-€ 45,255,830
4439	0.15	€ 228,453	€ 456,905	€ 685,358	€ 913,811	-€ 5,523,918	-€ 11,047,835	-€ 16,571,753	-€ 22,095,670
4440	0.20	€ 315,292	€ 630,585	€ 945,877	##########	-€ 7,623,673	-€ 15,247,346	-€ 22,871,019	-€ 30,494,692
4441	0.13	€ 210,584	€ 421,168	€ 631,751	€ 842,335	-€ 5,091,853	-€ 10,183,706	-€ 15,275,558	-€ 20,367,411
4442	0.17	€ 262,198	€ 524,396	€ 786,595	##########	-€ 6,339,875	-€ 12,679,749	-€ 19,019,624	-€ 25,359,499
4443	0.10	€ 160,960	€ 321,920	€ 482,880	€ 643,840	-€ 3,891,964	-€ 7,783,929	-€ 11,675,893	-€ 15,567,857
4444	0.27	€ 423,659	€ 847,318	##########	##########	-€ 10,243,951	-€ 20,487,902	-€ 30,731,853	-€ 40,975,804
4445	0.20	€ 308,300	€ 616,601	€ 924,901	##########	-€ 7,454,613	-€ 14,909,225	-€ 22,363,838	-€ 29,818,451
4446	0.24	€ 376,982	€ 753,964	##########	##########	-€ 9,115,313	-€ 18,230,626	-€ 27,345,939	-€ 36,461,252
4447	0.15	€ 230,776	€ 461,553	€ 692,329	€ 923,106	-€ 5,580,105	-€ 11,160,211	-€ 16,740,316	-€ 22,320,422
4448	0.22	€ 347,247	€ 694,495	##########	##########	-€ 8,396,341	-€ 16,792,682	-€ 25,189,023	-€ 33,585,365
4449	0.14	€ 225,265	€ 450,531	€ 675,796	€ 901,062	-€ 5,446,851	-€ 10,893,702	-€ 16,340,553	-€ 21,787,405
4450	0.19	€ 295,319	€ 590,637	€ 885,956	##########	-€ 7,140,714	-€ 14,281,428	-€ 21,422,142	-€ 28,562,857
4451	0.17	€ 267,281	€ 534,562	€ 801,843	##########	-€ 6,462,772	-€ 12,925,544	-€ 19,388,316	-€ 25,851,088
4452	0.20	€ 308,445	€ 616,889	€ 925,334	##########	-€ 7,458,101	-€ 14,916,202	-€ 22,374,302	-€ 29,832,403
4453	0.22	€ 350,004	€ 700,008	##########	##########	-€ 8,462,989	-€ 16,925,977	-€ 25,388,966	-€ 33,851,955
4454	0.15	€ 229,543	€ 459,087	€ 688,630	€ 918,173	-€ 5,550,289	-€ 11,100,578	-€ 16,650,866	-€ 22,201,155
4455	0.17	€ 266,730	€ 533,460	€ 800,190	##########	-€ 6,449,453	-€ 12,898,906	-€ 19,348,359	-€ 25,797,812
4456	0.14	€ 216,090	€ 432,181	€ 648,271	€ 864,361	-€ 5,225,000	-€ 10,450,001	-€ 15,675,001	-€ 20,900,001
4457	0.22	€ 348,752	€ 697,505	##########	##########	-€ 8,432,731	-€ 16,865,461	-€ 25,298,192	-€ 33,730,922
4458	0.17	€ 262,325	€ 524,651	€ 786,976	##########	-€ 6,342,947	-€ 12,685,894	-€ 19,028,841	-€ 25,371,789
4459	0.26	€ 404,603	€ 809,206	##########	##########	-€ 9,783,180	-€ 19,566,360	-€ 29,349,541	-€ 39,132,721
4460	0.23	€ 361,284	€ 722,567	##########	##########	-€ 8,735,733	-€ 17,471,466	-€ 26,207,199	-€ 34,942,933
4461	0.24	€ 381,353	€ 762,706	##########	##########	-€ 9,221,004	-€ 18,442,009	-€ 27,663,013	-€ 36,884,018
4462	0.15	€ 232,140	€ 464,279	€ 696,419	€ 928,559	-€ 5,613,069	-€ 11,226,139	-€ 16,839,208	-€ 22,452,277
4463	0.15	€ 241,080	€ 482,161	€ 723,241	€ 964,322	-€ 5,829,253	-€ 11,658,506	-€ 17,487,759	-€ 23,317,011
4464	0.25	€ 395,438	€ 790,876	##########	##########	-€ 9,561,569	-€ 19,123,139	-€ 28,684,708	-€ 38,246,278
4465	0.18	€ 285,336	€ 570,672	€ 856,008	##########	-€ 6,899,341	-€ 13,798,682	-€ 20,698,023	-€ 27,597,364
4466	0.15	€ 234,807	€ 469,613	€ 704,420	€ 939,226	-€ 5,677,553	-€ 11,355,106	-€ 17,032,659	-€ 22,710,212
4467	0.16	€ 251,628	€ 503,257	€ 754,885	##########	-€ 6,084,302	-€ 12,168,604	-€ 18,252,906	-€ 24,337,208
4468	0.21	€ 326,708	€ 653,415	€ 980,123	##########	-€ 7,899,695	-€ 15,799,391	-€ 23,699,086	-€ 31,598,781
4469	0.21	€ 327,801	€ 655,601	€ 983,402	##########	-€ 7,926,121	-€ 15,852,243	-€ 23,778,364	-€ 31,704,486
4470	0.18	€ 286,366	€ 572,732	€ 859,098	##########	-€ 6,924,243	-€ 13,848,486	-€ 20,772,728	-€ 27,696,971
4471	0.12	€ 184,342	€ 368,683	€ 553,025	€ 737,367	-€ 4,457,328	-€ 8,914,656	-€ 13,371,984	-€ 17,829,312
4472	0.18	€ 276,694	€ 553,387	€ 830,081	##########	-€ 6,690,373	-€ 13,380,745	-€ 20,071,118	-€ 26,761,490
4473	0.23	€ 365,377	€ 730,753	##########	##########	-€ 8,834,698	-€ 17,669,396	-€ 26,504,095	-€ 35,338,793
4474	0.16	€ 258,062	€ 516,124	€ 774,186	##########	-€ 6,239,859	-€ 12,479,719	-€ 18,719,578	-€ 24,959,438
4475	0.26	€ 410,411	€ 820,823	##########	##########	-€ 9,923,628	-€ 19,847,255	-€ 29,770,883	-€ 39,694,510
4476	0.19	€ 298,615	€ 597,230	€ 895,844	##########	-€ 7,220,416	-€ 14,440,833	-€ 21,661,249	-€ 28,881,666
4477	0.16	€ 259,226	€ 518,453	€ 777,679	##########	-€ 6,268,019	-€ 12,536,037	-€ 18,804,056	-€ 25,072,074
4478	0.14	€ 228,303	€ 456,607	€ 684,910	€ 913,214	-€ 5,520,308	-€ 11,040,616	-€ 16,560,925	-€ 22,081,233
4479	0.15	€ 230,532	€ 461,064	€ 691,596	€ 922,128	-€ 5,574,196	-€ 11,148,392	-€ 16,722,587	-€ 22,296,783
4480	0.20	€ 316,131	€ 632,263	€ 948,394	##########	-€ 7,643,964	-€ 15,287,929	-€ 22,931,893	-€ 30,575,857
4481	0.14	€ 225,585	€ 451,171	€ 676,756	€ 902,342	-€ 5,454,588	-€ 10,909,175	-€ 16,363,763	-€ 21,818,351
4482	0.29	€ 453,031	€ 906,062	##########	##########	-€ 10,954,158	-€ 21,908,316	-€ 32,862,474	-€ 43,816,632
4483	0.18	€ 275,649	€ 551,298	€ 826,947	##########	-€ 6,665,107	-€ 13,330,214	-€ 19,995,321	-€ 26,660,428
4484	0.23	€ 360,281	€ 720,563	##########	##########	-€ 8,711,495	-€ 17,422,991	-€ 26,134,486	-€ 34,845,981

4485	0.20	€ 314,924	€ 629,848	€ 944,772	##########	-€ 7,614,766	-€ 15,229,532	-€ 22,844,297	-€ 30,459,063
4486	0.14	€ 217,875	€ 435,749	€ 653,624	€ 871,498	-€ 5,268,142	-€ 10,536,284	-€ 15,804,426	-€ 21,072,568
4487	0.18	€ 286,411	€ 572,822	€ 859,234	##########	-€ 6,925,338	-€ 13,850,677	-€ 20,776,015	-€ 27,701,354
4488	0.16	€ 246,022	€ 492,045	€ 738,067	€ 984,089	-€ 5,948,746	-€ 11,897,493	-€ 17,846,239	-€ 23,794,986
4489	0.20	€ 310,664	€ 621,329	€ 931,993	##########	-€ 7,511,769	-€ 15,023,539	-€ 22,535,308	-€ 30,047,078
4490	0.18	€ 288,273	€ 576,547	€ 864,820	##########	-€ 6,970,364	-€ 13,940,729	-€ 20,911,093	-€ 27,881,457
4491	0.25	€ 396,605	€ 793,210	##########	##########	-€ 9,589,787	-€ 19,179,573	-€ 28,769,360	-€ 38,359,147
4492	0.18	€ 283,816	€ 567,632	€ 851,448	##########	-€ 6,862,583	-€ 13,725,165	-€ 20,587,748	-€ 27,450,331
4493	0.26	€ 407,909	€ 815,817	##########	##########	-€ 9,863,112	-€ 19,726,224	-€ 29,589,336	-€ 39,452,447
4494	0.20	€ 312,002	€ 624,004	€ 936,006	##########	-€ 7,544,112	-€ 15,088,224	-€ 22,632,336	-€ 30,176,448
4495	0.22	€ 347,680	€ 695,359	##########	##########	-€ 8,406,788	-€ 16,813,576	-€ 25,220,364	-€ 33,627,152
4496	0.20	€ 309,114	€ 618,228	€ 927,343	##########	-€ 7,474,290	-€ 14,948,580	-€ 22,422,870	-€ 29,897,160
4497	0.11	€ 172,960	€ 345,920	€ 518,881	€ 691,841	-€ 4,182,127	-€ 8,364,254	-€ 12,546,381	-€ 16,728,508
4498	0.17	€ 273,321	€ 546,643	€ 819,964	##########	-€ 6,608,832	-€ 13,217,664	-€ 19,826,496	-€ 26,435,328
4499	0.22	€ 339,477	€ 678,954	##########	##########	-€ 8,208,453	-€ 16,416,905	-€ 24,625,358	-€ 32,833,810
4500	0.18	€ 282,062	€ 564,124	€ 846,186	##########	-€ 6,820,178	-€ 13,640,355	-€ 20,460,533	-€ 27,280,711
4501	0.13	€ 204,697	€ 409,395	€ 614,092	€ 818,789	-€ 4,949,520	-€ 9,899,040	-€ 14,848,560	-€ 19,798,080
4502	0.12	€ 193,644	€ 387,289	€ 580,933	€ 774,577	-€ 4,682,262	-€ 9,364,524	-€ 14,046,786	-€ 18,729,048
4503	0.16	€ 253,856	€ 507,712	€ 761,567	##########	-€ 6,138,158	-€ 12,276,316	-€ 18,414,474	-€ 24,552,632
4504	0.13	€ 211,718	€ 423,437	€ 635,155	€ 846,874	-€ 5,119,290	-€ 10,238,580	-€ 15,357,870	-€ 20,477,160
4505	0.17	€ 264,129	€ 528,257	€ 792,386	##########	-€ 6,386,549	-€ 12,773,098	-€ 19,159,647	-€ 25,546,197
4506	0.19	€ 291,770	€ 583,541	€ 875,311	##########	-€ 7,054,919	-€ 14,109,839	-€ 21,164,758	-€ 28,219,677
4507	0.10	€ 163,479	€ 326,958	€ 490,437	€ 653,915	-€ 3,952,870	-€ 7,905,741	-€ 11,858,611	-€ 15,811,481
4508	0.25	€ 401,189	€ 802,379	##########	##########	-€ 9,700,639	-€ 19,401,279	-€ 29,101,918	-€ 38,802,558
4509	0.15	€ 242,216	€ 484,431	€ 726,647	€ 968,863	-€ 5,856,704	-€ 11,713,408	-€ 17,570,112	-€ 23,426,816
4510	0.27	€ 428,421	€ 856,841	##########	##########	-€ 10,359,081	-€ 20,718,163	-€ 31,077,244	-€ 41,436,325
4511	0.16	€ 256,155	€ 512,311	€ 768,466	##########	-€ 6,193,761	-€ 12,387,522	-€ 18,581,283	-€ 24,775,044
4512	0.20	€ 316,747	€ 633,493	€ 950,240	##########	-€ 7,658,840	-€ 15,317,680	-€ 22,976,519	-€ 30,635,359
4513	0.18	€ 290,935	€ 581,869	€ 872,804	##########	-€ 7,034,711	-€ 14,069,423	-€ 21,104,134	-€ 28,138,846
4514	0.16	€ 253,622	€ 507,245	€ 760,867	##########	-€ 6,132,512	-€ 12,265,024	-€ 18,397,535	-€ 24,530,047
4515	0.16	€ 257,518	€ 515,037	€ 772,555	##########	-€ 6,226,717	-€ 12,453,435	-€ 18,680,152	-€ 24,906,870
4516	0.14	€ 220,914	€ 441,828	€ 662,742	€ 883,656	-€ 5,341,636	-€ 10,683,272	-€ 16,024,908	-€ 21,366,544
4517	0.20	€ 311,393	€ 622,785	€ 934,178	##########	-€ 7,529,383	-€ 15,058,765	-€ 22,588,148	-€ 30,117,530
4518	0.21	€ 323,674	€ 647,349	€ 971,023	##########	-€ 7,826,352	-€ 15,652,703	-€ 23,479,055	-€ 31,305,407
4519	0.19	€ 306,587	€ 613,174	€ 919,761	##########	-€ 7,413,180	-€ 14,826,359	-€ 22,239,539	-€ 29,652,719
4520	0.13	€ 198,759	€ 397,519	€ 596,278	€ 795,038	-€ 4,805,945	-€ 9,611,890	-€ 14,417,835	-€ 19,223,781
4521	0.23	€ 358,917	€ 717,835	##########	##########	-€ 8,678,517	-€ 17,357,033	-€ 26,035,550	-€ 34,714,066
4522	0.18	€ 286,908	€ 573,815	€ 860,723	##########	-€ 6,937,339	-€ 13,874,678	-€ 20,812,017	-€ 27,749,355
4523	0.19	€ 294,028	€ 588,056	€ 882,084	##########	-€ 7,109,509	-€ 14,219,019	-€ 21,328,528	-€ 28,438,037
4524	0.19	€ 302,772	€ 605,544	€ 908,316	##########	-€ 7,320,938	-€ 14,641,876	-€ 21,962,815	-€ 29,283,753
4525	0.18	€ 278,689	€ 557,377	€ 836,066	##########	-€ 6,738,608	-€ 13,477,217	-€ 20,215,825	-€ 26,954,433
4526	0.23	€ 364,025	€ 728,049	##########	##########	-€ 8,802,010	-€ 17,604,020	-€ 26,406,030	-€ 35,208,040
4527	0.19	€ 301,150	€ 602,299	€ 903,449	##########	-€ 7,281,710	-€ 14,563,419	-€ 21,845,129	-€ 29,126,838
4528	0.23	€ 355,260	€ 710,520	##########	##########	-€ 8,590,080	-€ 17,180,160	-€ 25,770,240	-€ 34,360,320
4529	0.23	€ 366,075	€ 732,150	##########	##########	-€ 8,851,590	-€ 17,703,180	-€ 26,554,770	-€ 35,406,361
4530	0.16	€ 259,605	€ 519,209	€ 778,814	##########	-€ 6,277,160	-€ 12,554,321	-€ 18,831,481	-€ 25,108,642
4531	0.16	€ 252,378	€ 504,756	€ 757,134	##########	-€ 6,102,424	-€ 12,204,847	-€ 18,307,271	-€ 24,409,695
4532	0.27	€ 419,487	€ 838,975	##########	##########	-€ 10,143,081	-€ 20,286,161	-€ 30,429,242	-€ 40,572,323
4533	0.21	€ 334,055	€ 668,109	##########	##########	-€ 8,077,340	-€ 16,154,680	-€ 24,232,019	-€ 32,309,359
4534	0.15	€ 236,842	€ 473,683	€ 710,525	€ 947,366	-€ 5,726,760	-€ 11,453,520	-€ 17,180,280	-€ 22,907,040
4535	0.21	€ 325,534	€ 651,067	€ 976,601	##########	-€ 7,871,306	-€ 15,742,613	-€ 23,613,919	-€ 31,485,225
4536	0.16	€ 258,247	€ 516,493	€ 774,740	##########	-€ 6,244,327	-€ 12,488,654	-€ 18,732,982	-€ 24,977,309
4537	0.17	€ 267,045	€ 534,090	€ 801,136	##########	-€ 6,457,074	-€ 12,914,147	-€ 19,371,221	-€ 25,828,294
4538	0.12	€ 190,338	€ 380,677	€ 571,015	€ 761,353	-€ 4,602,324	-€ 9,204,648	-€ 13,806,972	-€ 18,409,296
4539	0.18	€ 289,746	€ 579,491	€ 869,237	##########	-€ 7,005,961	-€ 14,011,923	-€ 21,017,884	-€ 28,023,846
4540	0.29	€ 450,713	€ 901,426	##########	##########	-€ 10,898,101	-€ 21,796,203	-€ 32,694,304	-€ 43,592,405
4541	0.18	€ 279,819	€ 559,639	€ 839,458	##########	-€ 6,765,949	-€ 13,531,897	-€ 20,297,846	-€ 27,063,795
4542	0.18	€ 288,203	€ 576,406	€ 864,610	##########	-€ 6,968,668	-€ 13,937,336	-€ 20,906,004	-€ 27,874,673
4543	0.20	€ 312,323	€ 624,645	€ 936,968	##########	-€ 7,551,867	-€ 15,103,734	-€ 22,655,601	-€ 30,207,468
4544	0.23	€ 361,948	€ 723,895	##########	##########	-€ 8,751,788	-€ 17,503,576	-€ 26,255,365	-€ 35,007,153
4545	0.25	€ 391,600	€ 783,200	##########	##########	-€ 9,468,771	-€ 18,937,542	-€ 28,406,313	-€ 37,875,084
4546	0.17	€ 261,656	€ 523,313	€ 784,969	##########	-€ 6,326,772	-€ 12,653,544	-€ 18,980,315	-€ 25,307,087
4547	0.23	€ 360,030	€ 720,061	##########	##########	-€ 8,705,429	-€ 17,410,858	-€ 26,116,286	-€ 34,821,715
4548	0.21	€ 334,949	€ 669,897	##########	##########	-€ 8,098,961	-€ 16,197,921	-€ 24,296,882	-€ 32,395,843
4549	0.20	€ 311,442	€ 622,883	€ 934,325	##########	-€ 7,530,564	-€ 15,061,128	-€ 22,591,693	-€ 30,122,257
4550	0.16	€ 247,696	€ 495,391	€ 743,087	€ 990,782	-€ 5,989,204	-€ 11,978,408	-€ 17,967,612	-€ 23,956,816
4551	0.20	€ 320,541	€ 641,081	€ 961,622	##########	-€ 7,750,575	-€ 15,501,150	-€ 23,251,724	-€ 31,002,299
4552	0.15	€ 231,156	€ 462,312	€ 693,468	€ 924,624	-€ 5,589,285	-€ 11,178,571	-€ 16,767,856	-€ 22,357,142
4553	0.15	€ 240,875	€ 481,750	€ 722,625	€ 963,500	-€ 5,824,285	-€ 11,648,570	-€ 17,472,855	-€ 23,297,139
4554	0.15	€ 236,716	€ 473,433	€ 710,149	€ 946,866	-€ 5,723,734	-€ 11,447,468	-€ 17,171,203	-€ 22,894,937
4555	0.21	€ 337,923	€ 675,846	##########	##########	-€ 8,170,874	-€ 16,341,748	-€ 24,512,622	-€ 32,683,496
4556	0.16	€ 252,533	€ 505,066	€ 757,599	##########	-€ 6,106,172	-€ 12,212,344	-€ 18,318,515	-€ 24,424,687
4557	0.15	€ 232,128	€ 464,256	€ 696,383	€ 928,511	-€ 5,612,781	-€ 11,225,562	-€ 16,838,343	-€ 22,451,124
4558	0.11	€ 175,352	€ 350,704	€ 526,056	€ 701,408	-€ 4,239,960	-€ 8,479,920	-€ 12,719,880	-€ 16,959,840
4559	0.23	€ 364,454	€ 728,907	##########	##########	-€ 8,812,381	-€ 17,624,762	-€ 26,437,143	-€ 35,249,524
4560	0.27	€ 432,250	€ 864,501	##########	##########	-€ 10,451,689	-€ 20,903,377	-€ 31,355,066	-€ 41,806,754
4561	0.17	€ 269,558	€ 539,115	€ 808,673	##########	-€ 6,517,825	-€ 13,035,650	-€ 19,553,475	-€ 26,071,300
4562	0.17	€ 266,459	€ 532,918	€ 799,377	##########	-€ 6,442,898	-€ 12,885,796	-€ 19,328,694	-€ 25,771,591
4563	0.24	€ 378,765	€ 757,530	##########	##########	-€ 9,158,430	-€ 18,316,860	-€ 27,475,290	-€ 36,633,720
4564	0.16	€ 253,948	€ 507,897	€ 761,845	##########	-€ 6,140,398	-€ 12,280,796	-€ 18,421,195	-€ 24,561,593
4565	0.23	€ 357,560	€ 715,120	##########	##########	-€ 8,645,696	-€ 17,291,391	-€ 25,937,087	-€ 34,582,782
4566	0.15	€ 243,504	€ 487,008	€ 730,511	€ 974,015	-€ 5,887,850	-€ 11,775,700	-€ 17,663,550	-€ 23,551,401
4567	0.15	€ 232,863	€ 465,726	€ 698,589	€ 931,452	-€ 5,630,559	-€ 11,261,119	-€ 16,891,678	-€ 22,522,237
4568	0.18	€ 289,467	€ 578,934	€ 868,401	##########	-€ 6,999,224	-€ 13,998,447	-€ 20,997,671	-€ 27,996,894
4569	0.23	€ 356,055	€ 712,109	##########	##########	-€ 8,609,296	-€ 17,218,592	-€ 25,827,889	-€ 34,437,185
4570	0.19	€ 305,387	€ 610,774	€ 916,161	##########	-€ 7,384,170	-€ 14,768,340	-€ 22,152,510	-€ 29,536,680
4571	0.20	€ 310,292	€ 620,584	€ 930,876	##########	-€ 7,502,771	-€ 15,005,542	-€ 22,508,314	-€ 30,011,085
4572	0.27	€ 425,912	€ 851,825	##########	##########	-€ 10,298,434	-€ 20,596,869	-€ 30,895,303	-€ 41,193,737
4573	0.25	€ 390,096	€ 780,193	##########	##########	-€ 9,432,412	-€ 18,864,824	-€ 28,297,236	-€ 37,729,648
4574	0.24	€ 373,492	€ 746,985	##########	##########	-€ 9,030,937	-€ 18,061,874	-€ 27,092,812	-€ 36,123,749
4575	0.20	€ 318,662	€ 637,325	€ 955,987	##########	-€ 7,705,163	-€ 15,410,326	-€ 23,115,489	-€ 30,820,652
4576	0.20	€ 316,767	€ 633,534	€ 950,301	##########	-€ 7,659,334	-€ 15,318,668	-€ 22,978,002	-€ 30,637,336
4577	0.24	€ 371,528	€ 743,056	##########	##########	-€ 8,983,441	-€ 17,966,882	-€ 26,950,324	-€ 35,933,765
4578	0.24	€ 378,177	€ 756,354	##########	##########	-€ 9,144,210	-€ 18,288,420	-€ 27,432,630	-€ 36,576,840
4579	0.21	€ 334,924	€ 669,849	##########	##########	-€ 8,098,374	-€ 16,196,747	-€ 24,295,121	-€ 32,393,494
4580	0.26	€ 402,080	€ 804,160	##########	##########	-€ 9,722,170	-€ 19,444,341	-€ 29,166,511	-€ 38,888,682
4581	0.17	€ 275,073	€ 550,146	€ 825,219	##########	-€ 6,651,183	-€ 13,302,365	-€ 19,953,548	-€ 26,604,730
4582	0.16	€ 251,383	€ 502,766	€ 754,149	##########	-€ 6,078,366	-€ 12,156,731	-€ 18,235,097	-€ 24,313,462
4583	0.21	€ 323,202	€ 646,405	€ 969,607	##########	-€ 7,814,938	-€ 15,629,876	-€ 23,444,815	-€ 31,259,753
4584	0.16	€ 256,113	€ 512,226	€ 768,339	##########	-€ 6,192,735	-€ 12,385,471	-€ 18,578,206	-€ 24,770,942
4585	0.18	€ 289,917	€ 579,834	€ 869,752	##########	-€ 7,010,113	-€ 14,020,225	-€ 21,030,338	-€ 28,040,450
4586	0.14	€ 218,642	€ 437,285	€ 655,927	€ 874,570	-€ 5,286,709	-€ 10,573,419	-€ 15,860,128	-€ 21,146,838

4587	0.19	€ 299,418	€ 598,836	€ 898,254	##########	-€ 7,239,835	-€ 14,479,670	-€ 21,719,505	-€ 28,959,341
4588	0.15	€ 234,382	€ 468,763	€ 703,145	€ 937,527	-€ 5,667,278	-€ 11,334,556	-€ 17,001,835	-€ 22,669,113
4589	0.21	€ 335,565	€ 671,131	##########	##########	-€ 8,113,874	-€ 16,227,748	-€ 24,341,622	-€ 32,455,496
4590	0.22	€ 345,821	€ 691,642	##########	##########	-€ 8,361,855	-€ 16,723,710	-€ 25,085,564	-€ 33,447,419
4591	0.20	€ 315,427	€ 630,853	€ 946,280	##########	-€ 7,626,920	-€ 15,253,840	-€ 22,880,760	-€ 30,507,679
4592	0.22	€ 352,776	€ 705,552	##########	##########	-€ 8,530,015	-€ 17,060,031	-€ 25,590,046	-€ 34,120,061
4593	0.21	€ 334,389	€ 668,778	##########	##########	-€ 8,085,425	-€ 16,170,851	-€ 24,256,276	-€ 32,341,702
4594	0.16	€ 253,286	€ 506,571	€ 759,857	##########	-€ 6,124,373	-€ 12,248,747	-€ 18,373,120	-€ 24,497,494
4595	0.17	€ 264,727	€ 529,455	€ 794,182	##########	-€ 6,401,029	-€ 12,802,057	-€ 19,203,086	-€ 25,604,114
4596	0.23	€ 357,456	€ 714,911	##########	##########	-€ 8,643,169	-€ 17,286,338	-€ 25,929,507	-€ 34,572,676
4597	0.22	€ 343,500	€ 686,999	##########	##########	-€ 8,305,717	-€ 16,611,435	-€ 24,917,152	-€ 33,222,870
4598	0.17	€ 271,119	€ 542,238	€ 813,357	##########	-€ 6,555,575	-€ 13,111,149	-€ 19,666,724	-€ 26,222,298
4599	0.18	€ 287,072	€ 574,143	€ 861,215	##########	-€ 6,941,307	-€ 13,882,614	-€ 20,823,921	-€ 27,765,229
4600	0.18	€ 291,167	€ 582,334	€ 873,500	##########	-€ 7,040,327	-€ 14,080,654	-€ 21,120,982	-€ 28,161,309
4601	0.14	€ 214,364	€ 428,729	€ 643,093	€ 857,457	-€ 5,183,267	-€ 10,366,533	-€ 15,549,800	-€ 20,733,067
4602	0.22	€ 345,305	€ 690,611	##########	##########	-€ 8,349,379	-€ 16,698,757	-€ 25,048,136	-€ 33,397,514
4603	0.20	€ 315,633	€ 631,267	€ 946,900	##########	-€ 7,631,923	-€ 15,263,845	-€ 22,895,768	-€ 30,527,690
4604	0.13	€ 208,622	€ 417,244	€ 625,866	€ 834,488	-€ 5,044,416	-€ 10,088,832	-€ 15,133,248	-€ 20,177,664
4605	0.17	€ 267,464	€ 534,928	€ 802,392	##########	-€ 6,467,201	-€ 12,934,401	-€ 19,401,602	-€ 25,868,802
4606	0.23	€ 368,413	€ 736,826	##########	##########	-€ 8,908,113	-€ 17,816,226	-€ 26,724,340	-€ 35,632,453
4607	0.24	€ 383,301	€ 766,602	##########	##########	-€ 9,268,107	-€ 18,536,214	-€ 27,804,321	-€ 37,072,428
4608	0.19	€ 303,875	€ 607,750	€ 911,625	##########	-€ 7,347,610	-€ 14,695,220	-€ 22,042,831	-€ 29,390,441
4609	0.20	€ 308,633	€ 617,266	€ 925,898	##########	-€ 7,462,650	-€ 14,925,300	-€ 22,387,950	-€ 29,850,600
4610	0.18	€ 288,856	€ 577,711	€ 866,567	##########	-€ 6,984,442	-€ 13,968,883	-€ 20,953,325	-€ 27,937,767
4611	0.20	€ 309,189	€ 618,377	€ 927,566	##########	-€ 7,476,090	-€ 14,952,180	-€ 22,428,270	-€ 29,904,360
4612	0.29	€ 449,832	€ 899,664	##########	##########	-€ 10,876,798	-€ 21,753,597	-€ 32,630,395	-€ 43,507,194
4613	0.10	€ 157,795	€ 315,591	€ 473,386	€ 631,182	-€ 3,815,446	-€ 7,630,892	-€ 11,446,338	-€ 15,261,784
4614	0.25	€ 392,587	€ 785,173	##########	##########	-€ 9,492,628	-€ 18,985,255	-€ 28,477,883	-€ 37,970,510
4615	0.20	€ 317,365	€ 634,731	€ 952,096	##########	-€ 7,673,799	-€ 15,347,597	-€ 23,021,396	-€ 30,695,195
4616	0.18	€ 286,878	€ 573,757	€ 860,635	##########	-€ 6,936,631	-€ 13,873,263	-€ 20,809,894	-€ 27,746,525
4617	0.17	€ 268,202	€ 536,404	€ 804,606	##########	-€ 6,485,042	-€ 12,970,084	-€ 19,455,125	-€ 25,940,167
4618	0.15	€ 233,576	€ 467,152	€ 700,728	€ 934,304	-€ 5,647,799	-€ 11,295,598	-€ 16,943,398	-€ 22,591,197
4619	0.19	€ 304,032	€ 608,063	€ 912,095	##########	-€ 7,351,392	-€ 14,702,784	-€ 22,054,176	-€ 29,405,568
4620	0.19	€ 296,494	€ 592,989	€ 889,483	##########	-€ 7,169,147	-€ 14,338,294	-€ 21,507,441	-€ 28,676,587
4621	0.19	€ 299,412	€ 598,824	€ 898,235	##########	-€ 7,239,689	-€ 14,479,378	-€ 21,719,067	-€ 28,958,756
4622	0.15	€ 240,625	€ 481,250	€ 721,874	€ 962,499	-€ 5,818,236	-€ 11,636,471	-€ 17,454,707	-€ 23,272,942
4623	0.19	€ 292,966	€ 585,932	€ 878,898	##########	-€ 7,083,828	-€ 14,167,655	-€ 21,251,483	-€ 28,335,310
4624	0.18	€ 286,316	€ 572,632	€ 858,948	##########	-€ 6,923,035	-€ 13,846,070	-€ 20,769,105	-€ 27,692,141
4625	0.18	€ 285,305	€ 570,610	€ 855,915	##########	-€ 6,898,588	-€ 13,797,176	-€ 20,695,764	-€ 27,594,352
4626	0.22	€ 347,778	€ 695,555	##########	##########	-€ 8,409,161	-€ 16,818,322	-€ 25,227,482	-€ 33,636,643
4627	0.16	€ 257,117	€ 514,234	€ 771,351	##########	-€ 6,217,014	-€ 12,434,027	-€ 18,651,041	-€ 24,868,055
4628	0.17	€ 274,458	€ 548,915	€ 823,373	##########	-€ 6,636,304	-€ 13,272,609	-€ 19,908,913	-€ 26,545,218
4629	0.17	€ 269,560	€ 539,119	€ 808,679	##########	-€ 6,517,874	-€ 13,035,747	-€ 19,553,621	-€ 26,071,494
4630	0.21	€ 326,968	€ 653,936	€ 980,905	##########	-€ 7,905,993	-€ 15,811,987	-€ 23,717,980	-€ 31,623,974
4631	0.28	€ 436,374	€ 872,747	##########	##########	-€ 10,551,386	-€ 21,102,773	-€ 31,654,159	-€ 42,205,545
4632	0.18	€ 286,461	€ 572,922	€ 859,382	##########	-€ 6,926,536	-€ 13,853,073	-€ 20,779,609	-€ 27,706,146
4633	0.19	€ 294,643	€ 589,286	€ 883,929	##########	-€ 7,124,381	-€ 14,248,762	-€ 21,373,143	-€ 28,497,524
4634	0.23	€ 365,512	€ 731,024	##########	##########	-€ 8,837,967	-€ 17,675,934	-€ 26,513,901	-€ 35,351,868
4635	0.19	€ 296,696	€ 593,393	€ 890,089	##########	-€ 7,174,030	-€ 14,348,060	-€ 21,522,090	-€ 28,696,119
4636	0.16	€ 252,041	€ 504,081	€ 756,122	##########	-€ 6,094,269	-€ 12,188,538	-€ 18,282,807	-€ 24,377,077
4637	0.25	€ 398,593	€ 797,187	##########	##########	-€ 9,637,871	-€ 19,275,742	-€ 28,913,613	-€ 38,551,484
4638	0.19	€ 299,939	€ 599,877	€ 899,816	##########	-€ 7,252,428	-€ 14,504,856	-€ 21,757,283	-€ 29,009,711
4639	0.17	€ 263,539	€ 527,078	€ 790,618	##########	-€ 6,372,301	-€ 12,744,601	-€ 19,116,902	-€ 25,489,203
4640	0.24	€ 370,210	€ 740,419	##########	##########	-€ 8,951,560	-€ 17,903,119	-€ 26,854,679	-€ 35,806,239
4641	0.17	€ 265,245	€ 530,490	€ 795,735	##########	-€ 6,413,544	-€ 12,827,088	-€ 19,240,632	-€ 25,654,176
4642	0.18	€ 288,162	€ 576,323	€ 864,485	##########	-€ 6,967,662	-€ 13,935,323	-€ 20,902,985	-€ 27,870,647
4643	0.21	€ 324,318	€ 648,636	€ 972,954	##########	-€ 7,841,915	-€ 15,683,830	-€ 23,525,745	-€ 31,367,660
4644	0.15	€ 241,055	€ 482,109	€ 723,164	€ 964,218	-€ 5,828,628	-€ 11,657,256	-€ 17,485,884	-€ 23,314,512
4645	0.12	€ 181,904	€ 363,809	€ 545,713	€ 727,618	-€ 4,398,395	-€ 8,796,791	-€ 13,195,186	-€ 17,593,581
4646	0.15	€ 238,663	€ 477,325	€ 715,988	€ 954,651	-€ 5,770,792	-€ 11,541,584	-€ 17,312,377	-€ 23,083,169
4647	0.18	€ 276,599	€ 553,197	€ 829,796	##########	-€ 6,688,074	-€ 13,376,148	-€ 20,064,222	-€ 26,752,295
4648	0.24	€ 382,180	€ 764,359	##########	##########	-€ 9,240,989	-€ 18,481,977	-€ 27,722,966	-€ 36,963,955
4649	0.13	€ 204,222	€ 408,444	€ 612,665	€ 816,887	-€ 4,938,022	-€ 9,876,044	-€ 14,814,066	-€ 19,752,088
4650	0.15	€ 231,203	€ 462,406	€ 693,610	€ 924,813	-€ 5,590,425	-€ 11,180,851	-€ 16,771,276	-€ 22,361,702
4651	0.18	€ 284,880	€ 569,760	€ 854,640	##########	-€ 6,888,317	-€ 13,776,634	-€ 20,664,952	-€ 27,553,269
4652	0.17	€ 273,968	€ 547,935	€ 821,903	##########	-€ 6,624,453	-€ 13,248,906	-€ 19,873,360	-€ 26,497,813
4653	0.24	€ 382,229	€ 764,458	##########	##########	-€ 9,242,185	-€ 18,484,370	-€ 27,726,554	-€ 36,968,739
4654	0.24	€ 373,656	€ 747,312	##########	##########	-€ 9,034,894	-€ 18,069,788	-€ 27,104,682	-€ 36,139,576
4655	0.15	€ 240,609	€ 481,217	€ 721,826	€ 962,435	-€ 5,817,845	-€ 11,635,691	-€ 17,453,536	-€ 23,271,381
4656	0.15	€ 234,185	€ 468,371	€ 702,556	€ 936,741	-€ 5,662,530	-€ 11,325,060	-€ 16,987,590	-€ 22,650,120
4657	0.15	€ 230,945	€ 461,890	€ 692,834	€ 923,779	-€ 5,584,177	-€ 11,168,353	-€ 16,752,530	-€ 22,336,706
4658	0.23	€ 360,936	€ 721,871	##########	##########	-€ 8,727,316	-€ 17,454,631	-€ 26,181,947	-€ 34,909,263
4659	0.19	€ 298,095	€ 596,189	€ 894,284	##########	-€ 7,207,842	-€ 14,415,685	-€ 21,623,527	-€ 28,831,370
4660	0.15	€ 229,826	€ 459,653	€ 689,479	€ 919,305	-€ 5,557,131	-€ 11,114,263	-€ 16,671,394	-€ 22,228,526
4661	0.16	€ 252,904	€ 505,809	€ 758,713	##########	-€ 6,115,151	-€ 12,230,301	-€ 18,345,452	-€ 24,460,603
4662	0.18	€ 288,859	€ 577,719	€ 866,578	##########	-€ 6,984,532	-€ 13,969,064	-€ 20,953,596	-€ 27,938,128
4663	0.13	€ 203,009	€ 406,017	€ 609,026	€ 812,034	-€ 4,908,685	-€ 9,817,371	-€ 14,726,056	-€ 19,634,741
4664	0.21	€ 334,580	€ 669,160	##########	##########	-€ 8,090,050	-€ 16,180,100	-€ 24,270,150	-€ 32,360,200
4665	0.14	€ 220,123	€ 440,246	€ 660,369	€ 880,492	-€ 5,322,510	-€ 10,645,019	-€ 15,967,529	-€ 21,290,038
4666	0.20	€ 312,570	€ 625,140	€ 937,711	##########	-€ 7,557,855	-€ 15,115,709	-€ 22,673,564	-€ 30,231,418
4667	0.17	€ 269,701	€ 539,401	€ 809,102	##########	-€ 6,521,283	-€ 13,042,565	-€ 19,563,848	-€ 26,085,130
4668	0.16	€ 252,991	€ 505,982	€ 758,973	##########	-€ 6,117,244	-€ 12,234,489	-€ 18,351,733	-€ 24,468,977
4669	0.08	€ 124,803	€ 249,605	€ 374,408	€ 499,210	-€ 3,017,689	-€ 6,035,377	-€ 9,053,066	-€ 12,070,755
4670	0.17	€ 263,095	€ 526,189	€ 789,284	##########	-€ 6,361,553	-€ 12,723,105	-€ 19,084,658	-€ 25,446,210
4671	0.15	€ 234,449	€ 468,897	€ 703,346	€ 937,795	-€ 5,668,899	-€ 11,337,798	-€ 17,006,698	-€ 22,675,597
4672	0.20	€ 322,391	€ 644,782	€ 967,173	##########	-€ 7,795,316	-€ 15,590,632	-€ 23,385,949	-€ 31,181,265
4673	0.19	€ 294,713	€ 589,425	€ 884,138	##########	-€ 7,126,061	-€ 14,252,122	-€ 21,378,182	-€ 28,504,243
4674	0.16	€ 249,022	€ 498,044	€ 747,066	€ 996,088	-€ 6,021,281	-€ 12,042,562	-€ 18,063,843	-€ 24,085,124
4675	0.18	€ 277,209	€ 554,417	€ 831,626	##########	-€ 6,702,825	-€ 13,405,650	-€ 20,108,475	-€ 26,811,299
4676	0.21	€ 325,231	€ 650,462	€ 975,693	##########	-€ 7,863,992	-€ 15,727,984	-€ 23,591,976	-€ 31,455,968
4677	0.21	€ 323,411	€ 646,822	€ 970,233	##########	-€ 7,819,983	-€ 15,639,966	-€ 23,459,949	-€ 31,279,932
4678	0.28	€ 447,453	€ 894,907	##########	##########	-€ 10,819,292	-€ 21,638,585	-€ 32,457,877	-€ 43,277,169
4679	0.18	€ 278,031	€ 556,062	€ 834,093	##########	-€ 6,722,709	-€ 13,445,419	-€ 20,168,128	-€ 26,890,838
4680	0.15	€ 229,013	€ 458,026	€ 687,039	€ 916,052	-€ 5,537,465	-€ 11,074,929	-€ 16,612,394	-€ 22,149,859
4681	0.22	€ 352,080	€ 704,160	##########	##########	-€ 8,513,195	-€ 17,026,390	-€ 25,539,585	-€ 34,052,780
4682	0.21	€ 334,615	€ 669,230	##########	##########	-€ 8,090,892	-€ 16,181,784	-€ 24,272,675	-€ 32,363,567
4683	0.15	€ 241,028	€ 482,057	€ 723,085	€ 964,113	-€ 5,827,993	-€ 11,655,985	-€ 17,483,978	-€ 23,311,971
4684	0.21	€ 332,702	€ 665,405	€ 998,107	##########	-€ 8,044,647	-€ 16,089,294	-€ 24,133,940	-€ 32,178,587
4685	0.17	€ 269,424	€ 538,848	€ 808,272	##########	-€ 6,514,594	-€ 13,029,188	-€ 19,543,782	-€ 26,058,376
4686	0.24	€ 371,560	€ 743,120	##########	##########	-€ 8,984,216	-€ 17,968,433	-€ 26,952,649	-€ 35,936,866
4687	0.19	€ 300,644	€ 601,289	€ 901,933	##########	-€ 7,269,490	-€ 14,538,980	-€ 21,808,470	-€ 29,077,960
4688	0.22	€ 344,699	€ 689,398	##########	##########	-€ 8,334,723	-€ 16,669,445	-€ 25,004,168	-€ 33,338,891

4689	0.20	€ 314,613	€ 629,225	€ 943,838	##########	-€ 7,607,240	-€ 15,214,479	-€ 22,821,719	-€ 30,428,959
4690	0.18	€ 286,570	€ 573,140	€ 859,710	##########	-€ 6,929,180	-€ 13,858,361	-€ 20,787,541	-€ 27,716,721
4691	0.25	€ 395,997	€ 791,994	##########	##########	-€ 9,575,084	-€ 19,150,168	-€ 28,725,252	-€ 38,300,336
4692	0.22	€ 349,934	€ 699,868	##########	##########	-€ 8,461,297	-€ 16,922,593	-€ 25,383,890	-€ 33,845,186
4693	0.18	€ 277,561	€ 555,122	€ 832,683	##########	-€ 6,711,344	-€ 13,422,689	-€ 20,134,033	-€ 26,845,378
4694	0.20	€ 308,400	€ 616,800	€ 925,200	##########	-€ 7,457,018	-€ 14,914,037	-€ 22,371,055	-€ 29,828,074
4695	0.22	€ 341,737	€ 683,474	##########	##########	-€ 8,263,097	-€ 16,526,194	-€ 24,789,290	-€ 33,052,387
4696	0.19	€ 305,204	€ 610,409	€ 915,613	##########	-€ 7,379,754	-€ 14,759,507	-€ 22,139,261	-€ 29,519,014
4697	0.16	€ 256,079	€ 512,158	€ 768,236	##########	-€ 6,191,910	-€ 12,383,820	-€ 18,575,730	-€ 24,767,641
4698	0.20	€ 318,626	€ 637,252	€ 955,878	##########	-€ 7,704,282	-€ 15,408,564	-€ 23,112,846	-€ 30,817,128
4699	0.20	€ 321,070	€ 642,141	€ 963,211	##########	-€ 7,763,389	-€ 15,526,778	-€ 23,290,167	-€ 31,053,556
4700	0.18	€ 288,860	€ 577,719	€ 866,579	##########	-€ 6,984,542	-€ 13,969,085	-€ 20,953,627	-€ 27,938,169
4701	0.21	€ 323,722	€ 647,443	€ 971,165	##########	-€ 7,827,494	-€ 15,654,989	-€ 23,482,483	-€ 31,309,977
4702	0.16	€ 253,948	€ 507,895	€ 761,843	##########	-€ 6,140,376	-€ 12,280,751	-€ 18,421,127	-€ 24,561,502
4703	0.17	€ 269,336	€ 538,672	€ 808,008	##########	-€ 6,512,462	-€ 13,024,924	-€ 19,537,386	-€ 26,049,848
4704	0.16	€ 246,148	€ 492,297	€ 738,445	€ 984,593	-€ 5,951,793	-€ 11,903,586	-€ 17,855,379	-€ 23,807,172
4705	0.16	€ 248,758	€ 497,516	€ 746,274	€ 995,032	-€ 6,014,898	-€ 12,029,795	-€ 18,044,693	-€ 24,059,590
4706	0.19	€ 296,897	€ 593,794	€ 890,691	##########	-€ 7,178,880	-€ 14,357,759	-€ 21,536,639	-€ 28,715,518
4707	0.19	€ 294,253	€ 588,507	€ 882,760	##########	-€ 7,114,958	-€ 14,229,916	-€ 21,344,874	-€ 28,459,832
4708	0.21	€ 329,562	€ 659,125	€ 988,687	##########	-€ 7,968,721	-€ 15,937,441	-€ 23,906,162	-€ 31,874,883
4709	0.12	€ 190,770	€ 381,540	€ 572,310	€ 763,080	-€ 4,612,761	-€ 9,225,523	-€ 13,838,284	-€ 18,451,046
4710	0.20	€ 313,454	€ 626,909	€ 940,363	##########	-€ 7,579,234	-€ 15,158,468	-€ 22,737,703	-€ 30,316,937
4711	0.17	€ 261,763	€ 523,525	€ 785,288	##########	-€ 6,329,345	-€ 12,658,690	-€ 18,988,035	-€ 25,317,380
4712	0.23	€ 355,986	€ 711,972	##########	##########	-€ 8,607,638	-€ 17,215,276	-€ 25,822,914	-€ 34,430,552
4713	0.12	€ 195,959	€ 391,919	€ 587,878	€ 783,837	-€ 4,738,237	-€ 9,476,474	-€ 14,214,710	-€ 18,952,947
4714	0.23	€ 360,514	€ 721,028	##########	##########	-€ 8,717,122	-€ 17,434,245	-€ 26,151,367	-€ 34,868,490
4715	0.22	€ 351,160	€ 702,321	##########	##########	-€ 8,490,953	-€ 16,981,907	-€ 25,472,860	-€ 33,963,814
4716	0.13	€ 200,479	€ 400,958	€ 601,437	€ 801,916	-€ 4,847,520	-€ 9,695,040	-€ 14,542,560	-€ 19,390,080
4717	0.14	€ 226,860	€ 453,720	€ 680,580	€ 907,440	-€ 5,485,408	-€ 10,970,815	-€ 16,456,223	-€ 21,941,630
4718	0.18	€ 289,963	€ 579,927	€ 869,890	##########	-€ 7,011,230	-€ 14,022,460	-€ 21,033,690	-€ 28,044,919
4719	0.20	€ 308,654	€ 617,309	€ 925,963	##########	-€ 7,463,171	-€ 14,926,343	-€ 22,389,514	-€ 29,852,685
4720	0.16	€ 245,729	€ 491,458	€ 737,186	€ 982,915	-€ 5,941,650	-€ 11,883,300	-€ 17,824,950	-€ 23,766,599
4721	0.15	€ 235,157	€ 470,314	€ 705,472	€ 940,629	-€ 5,686,031	-€ 11,372,061	-€ 17,058,092	-€ 22,744,122
4722	0.18	€ 283,439	€ 566,877	€ 850,316	##########	-€ 6,853,462	-€ 13,706,923	-€ 20,560,385	-€ 27,413,847
4723	0.16	€ 249,533	€ 499,066	€ 748,599	€ 998,132	-€ 6,033,636	-€ 12,067,272	-€ 18,100,908	-€ 24,134,544
4724	0.17	€ 273,425	€ 546,849	€ 820,274	##########	-€ 6,611,324	-€ 13,222,648	-€ 19,833,972	-€ 26,445,296
4725	0.23	€ 362,981	€ 725,962	##########	##########	-€ 8,776,774	-€ 17,553,548	-€ 26,330,322	-€ 35,107,096
4726	0.16	€ 258,570	€ 517,140	€ 775,710	##########	-€ 6,252,143	-€ 12,504,285	-€ 18,756,428	-€ 25,008,571
4727	0.21	€ 329,843	€ 659,685	€ 989,528	##########	-€ 7,975,497	-€ 15,950,993	-€ 23,926,490	-€ 31,901,987
4728	0.19	€ 302,327	€ 604,654	€ 906,981	##########	-€ 7,310,180	-€ 14,620,361	-€ 21,930,541	-€ 29,240,721
4729	0.21	€ 332,990	€ 665,979	€ 998,969	##########	-€ 8,051,588	-€ 16,103,175	-€ 24,154,763	-€ 32,206,351
4730	0.28	€ 437,085	€ 874,170	##########	##########	-€ 10,568,580	-€ 21,137,161	-€ 31,705,741	-€ 42,274,322
4731	0.24	€ 382,881	€ 765,761	##########	##########	-€ 9,257,939	-€ 18,515,878	-€ 27,773,817	-€ 37,031,756
4732	0.21	€ 328,305	€ 656,610	€ 984,915	##########	-€ 7,938,316	-€ 15,876,632	-€ 23,814,947	-€ 31,753,263
4733	0.23	€ 356,778	€ 713,556	##########	##########	-€ 8,626,792	-€ 17,253,584	-€ 25,880,375	-€ 34,507,167
4734	0.15	€ 240,433	€ 480,866	€ 721,299	€ 961,732	-€ 5,813,598	-€ 11,627,197	-€ 17,440,795	-€ 23,254,394
4735	0.19	€ 295,708	€ 591,415	€ 887,123	##########	-€ 7,150,120	-€ 14,300,240	-€ 21,450,360	-€ 28,600,480
4736	0.15	€ 240,968	€ 481,936	€ 722,904	€ 963,873	-€ 5,826,538	-€ 11,653,076	-€ 17,479,614	-€ 23,306,152
4737	0.24	€ 376,393	€ 752,786	##########	##########	-€ 9,101,073	-€ 18,202,147	-€ 27,303,220	-€ 36,404,293
4738	0.21	€ 325,766	€ 651,531	€ 977,297	##########	-€ 7,876,914	-€ 15,753,828	-€ 23,630,742	-€ 31,507,656
4739	0.10	€ 163,713	€ 327,426	€ 491,139	€ 654,852	-€ 3,958,533	-€ 7,917,067	-€ 11,875,600	-€ 15,834,134
4740	0.24	€ 377,326	€ 754,653	##########	##########	-€ 9,123,640	-€ 18,247,280	-€ 27,370,920	-€ 36,494,560
4741	0.18	€ 290,848	€ 581,696	€ 872,545	##########	-€ 7,032,623	-€ 14,065,246	-€ 21,097,870	-€ 28,130,493
4742	0.21	€ 324,266	€ 648,533	€ 972,799	##########	-€ 7,840,664	-€ 15,681,329	-€ 23,521,993	-€ 31,362,658
4743	0.20	€ 321,958	€ 643,916	€ 965,875	##########	-€ 7,784,853	-€ 15,569,707	-€ 23,354,560	-€ 31,139,414
4744	0.18	€ 288,693	€ 577,385	€ 866,078	##########	-€ 6,980,502	-€ 13,961,005	-€ 20,941,507	-€ 27,922,009
4745	0.24	€ 385,247	€ 770,493	##########	##########	-€ 9,315,150	-€ 18,630,300	-€ 27,945,450	-€ 37,260,600
4746	0.18	€ 284,255	€ 568,511	€ 852,766	##########	-€ 6,873,209	-€ 13,746,417	-€ 20,619,626	-€ 27,492,834
4747	0.20	€ 310,765	€ 621,529	€ 932,294	##########	-€ 7,514,197	-€ 15,028,394	-€ 22,542,592	-€ 30,056,789
4748	0.24	€ 376,975	€ 753,949	##########	##########	-€ 9,115,133	-€ 18,230,266	-€ 27,345,400	-€ 36,460,533
4749	0.20	€ 307,611	€ 615,223	€ 922,834	##########	-€ 7,437,950	-€ 14,875,899	-€ 22,313,849	-€ 29,751,798
4750	0.21	€ 330,648	€ 661,296	€ 991,944	##########	-€ 7,994,969	-€ 15,989,938	-€ 23,984,906	-€ 31,979,875
4751	0.22	€ 349,949	€ 699,897	##########	##########	-€ 8,461,656	-€ 16,923,312	-€ 25,384,968	-€ 33,846,624
4752	0.14	€ 223,955	€ 447,911	€ 671,866	€ 895,821	-€ 5,415,174	-€ 10,830,347	-€ 16,245,521	-€ 21,660,695
4753	0.17	€ 273,916	€ 547,832	€ 821,749	##########	-€ 6,623,212	-€ 13,246,424	-€ 19,869,635	-€ 26,492,847
4754	0.25	€ 388,751	€ 777,503	##########	##########	-€ 9,399,894	-€ 18,799,788	-€ 28,199,682	-€ 37,599,576
4755	0.25	€ 394,804	€ 789,609	##########	##########	-€ 9,546,252	-€ 19,092,504	-€ 28,638,756	-€ 38,185,008
4756	0.24	€ 382,927	€ 765,854	##########	##########	-€ 9,259,064	-€ 18,518,127	-€ 27,777,191	-€ 37,036,254
4757	0.23	€ 359,706	€ 719,412	##########	##########	-€ 8,697,582	-€ 17,395,163	-€ 26,092,745	-€ 34,790,327
4758	0.18	€ 287,264	€ 574,528	€ 861,792	##########	-€ 6,945,956	-€ 13,891,913	-€ 20,837,869	-€ 27,783,825
4759	0.20	€ 313,115	€ 626,230	€ 939,346	##########	-€ 7,571,033	-€ 15,142,066	-€ 22,713,100	-€ 30,284,133
4760	0.22	€ 346,851	€ 693,702	##########	##########	-€ 8,386,753	-€ 16,773,506	-€ 25,160,260	-€ 33,547,013
4761	0.22	€ 346,133	€ 692,265	##########	##########	-€ 8,369,386	-€ 16,738,773	-€ 25,108,159	-€ 33,477,546
4762	0.27	€ 424,042	€ 848,084	##########	##########	-€ 10,253,204	-€ 20,506,408	-€ 30,759,613	-€ 41,012,817
4763	0.18	€ 279,787	€ 559,575	€ 839,362	##########	-€ 6,765,178	-€ 13,530,356	-€ 20,295,534	-€ 27,060,713
4764	0.21	€ 332,612	€ 665,224	€ 997,835	##########	-€ 8,042,455	-€ 16,084,910	-€ 24,127,365	-€ 32,169,820
4765	0.20	€ 316,662	€ 633,324	€ 949,986	##########	-€ 7,656,791	-€ 15,313,581	-€ 22,970,372	-€ 30,627,162
4766	0.15	€ 230,879	€ 461,757	€ 692,636	€ 923,514	-€ 5,582,576	-€ 11,165,152	-€ 16,747,728	-€ 22,330,304
4767	0.21	€ 330,474	€ 660,948	€ 991,422	##########	-€ 7,990,761	-€ 15,981,523	-€ 23,972,284	-€ 31,963,046
4768	0.21	€ 324,477	€ 648,955	€ 973,432	##########	-€ 7,845,768	-€ 15,691,536	-€ 23,537,304	-€ 31,383,073
4769	0.19	€ 301,759	€ 603,518	€ 905,277	##########	-€ 7,296,443	-€ 14,592,886	-€ 21,889,330	-€ 29,185,773
4770	0.15	€ 228,569	€ 457,138	€ 685,707	€ 914,275	-€ 5,526,727	-€ 11,053,454	-€ 16,580,181	-€ 22,106,908
4771	0.21	€ 325,878	€ 651,756	€ 977,634	##########	-€ 7,879,630	-€ 15,759,260	-€ 23,638,890	-€ 31,518,520
4772	0.15	€ 236,534	€ 473,068	€ 709,601	€ 946,135	-€ 5,719,318	-€ 11,438,636	-€ 17,157,954	-€ 22,877,271
4773	0.20	€ 314,985	€ 629,970	€ 944,954	##########	-€ 7,616,238	-€ 15,232,476	-€ 22,848,714	-€ 30,464,952
4774	0.18	€ 275,916	€ 551,831	€ 827,747	##########	-€ 6,671,558	-€ 13,343,116	-€ 20,014,674	-€ 26,686,232
4775	0.13	€ 202,715	€ 405,430	€ 608,146	€ 810,861	-€ 4,901,594	-€ 9,803,188	-€ 14,704,782	-€ 19,606,376
4776	0.15	€ 241,333	€ 482,666	€ 723,999	€ 965,332	-€ 5,835,359	-€ 11,670,719	-€ 17,506,078	-€ 23,341,437
4777	0.19	€ 292,769	€ 585,539	€ 878,308	##########	-€ 7,079,077	-€ 14,158,155	-€ 21,237,232	-€ 28,316,310
4778	0.17	€ 275,468	€ 550,936	€ 826,404	##########	-€ 6,660,737	-€ 13,321,474	-€ 19,982,211	-€ 26,642,948
4779	0.17	€ 266,521	€ 533,041	€ 799,562	##########	-€ 6,444,392	-€ 12,888,784	-€ 19,333,176	-€ 25,777,568
4780	0.26	€ 402,506	€ 805,013	##########	##########	-€ 9,732,486	-€ 19,464,971	-€ 29,197,457	-€ 38,929,943
4781	0.24	€ 370,914	€ 741,827	##########	##########	-€ 8,968,581	-€ 17,937,162	-€ 26,905,744	-€ 35,874,325
4782	0.23	€ 369,642	€ 739,284	##########	##########	-€ 8,937,837	-€ 17,875,675	-€ 26,813,512	-€ 35,751,350
4783	0.21	€ 334,624	€ 669,249	##########	##########	-€ 8,091,116	-€ 16,182,232	-€ 24,273,348	-€ 32,364,465
4784	0.19	€ 304,952	€ 609,904	€ 914,856	##########	-€ 7,373,650	-€ 14,747,300	-€ 22,120,950	-€ 29,494,600
4785	0.17	€ 268,154	€ 536,307	€ 804,461	##########	-€ 6,483,875	-€ 12,967,749	-€ 19,451,624	-€ 25,935,498
4786	0.21	€ 335,258	€ 670,515	##########	##########	-€ 8,106,431	-€ 16,212,862	-€ 24,319,294	-€ 32,425,725
4787	0.20	€ 308,268	€ 616,536	€ 924,805	##########	-€ 7,453,835	-€ 14,907,669	-€ 22,361,504	-€ 29,815,339
4788	0.20	€ 309,920	€ 619,840	€ 929,760	##########	-€ 7,493,776	-€ 14,987,552	-€ 22,481,328	-€ 29,975,104
4789	0.18	€ 285,884	€ 571,767	€ 857,651	##########	-€ 6,912,580	-€ 13,825,160	-€ 20,737,740	-€ 27,650,320
4790	0.19	€ 302,430	€ 604,860	€ 907,289	##########	-€ 7,312,662	-€ 14,625,325	-€ 21,937,987	-€ 29,250,649

4791	0.16	€ 244,651	€ 489,301	€ 733,952	€ 978,603	-€ 5,915,581	-€ 11,831,162	-€ 17,746,743	-€ 23,662,324
4792	0.11	€ 165,959	€ 331,919	€ 497,878	€ 663,838	-€ 4,012,850	-€ 8,025,700	-€ 12,038,550	-€ 16,051,400
4793	0.24	€ 374,054	€ 748,107	##########	##########	-€ 9,044,507	-€ 18,089,014	-€ 27,133,521	-€ 36,178,028
4794	0.15	€ 237,730	€ 475,459	€ 713,189	€ 950,919	-€ 5,748,233	-€ 11,496,467	-€ 17,244,700	-€ 22,992,934
4795	0.16	€ 248,945	€ 497,890	€ 746,834	€ 995,779	-€ 6,019,411	-€ 12,038,822	-€ 18,058,233	-€ 24,077,644
4796	0.26	€ 414,655	€ 829,309	##########	##########	-€ 10,026,226	-€ 20,052,452	-€ 30,078,678	-€ 40,104,904
4797	0.22	€ 341,518	€ 683,036	##########	##########	-€ 8,257,802	-€ 16,515,605	-€ 24,773,407	-€ 33,031,210
4798	0.19	€ 297,280	€ 594,561	€ 891,841	##########	-€ 7,188,149	-€ 14,376,299	-€ 21,564,448	-€ 28,752,597
4799	0.20	€ 314,793	€ 629,586	€ 944,379	##########	-€ 7,611,597	-€ 15,223,195	-€ 22,834,792	-€ 30,446,389
4800	0.14	€ 221,456	€ 442,911	€ 664,367	€ 885,822	-€ 5,354,730	-€ 10,709,461	-€ 16,064,191	-€ 21,418,921
4801	0.18	€ 285,553	€ 571,105	€ 856,658	##########	-€ 6,904,578	-€ 13,809,157	-€ 20,713,735	-€ 27,618,314
4802	0.19	€ 299,534	€ 599,069	€ 898,603	##########	-€ 7,242,649	-€ 14,485,299	-€ 21,727,948	-€ 28,970,597
4803	0.19	€ 296,199	€ 592,397	€ 888,596	##########	-€ 7,161,994	-€ 14,323,988	-€ 21,485,982	-€ 28,647,976
4804	0.22	€ 339,346	€ 678,693	##########	##########	-€ 8,205,295	-€ 16,410,589	-€ 24,615,884	-€ 32,821,179
4805	0.17	€ 269,168	€ 538,336	€ 807,503	##########	-€ 6,508,397	-€ 13,016,794	-€ 19,525,191	-€ 26,033,588
4806	0.18	€ 278,442	€ 556,885	€ 835,327	##########	-€ 6,732,655	-€ 13,465,310	-€ 20,197,965	-€ 26,930,620
4807	0.17	€ 269,502	€ 539,003	€ 808,505	##########	-€ 6,516,471	-€ 13,032,942	-€ 19,549,413	-€ 26,065,883
4808	0.22	€ 338,635	€ 677,271	##########	##########	-€ 8,188,106	-€ 16,376,211	-€ 24,564,317	-€ 32,752,423
4809	0.19	€ 295,724	€ 591,449	€ 887,173	##########	-€ 7,150,530	-€ 14,301,059	-€ 21,451,589	-€ 28,602,119
4810	0.22	€ 339,508	€ 679,017	##########	##########	-€ 8,209,209	-€ 16,418,419	-€ 24,627,628	-€ 32,836,837
4811	0.26	€ 415,762	€ 831,524	##########	##########	-€ 10,053,000	-€ 20,105,999	-€ 30,158,999	-€ 40,211,998
4812	0.15	€ 236,984	€ 473,969	€ 710,953	€ 947,938	-€ 5,730,214	-€ 11,460,428	-€ 17,190,643	-€ 22,920,857
4813	0.27	€ 429,000	€ 857,999	##########	##########	-€ 10,373,081	-€ 20,746,162	-€ 31,119,243	-€ 41,492,324
4814	0.22	€ 347,265	€ 694,529	##########	##########	-€ 8,396,754	-€ 16,793,508	-€ 25,190,263	-€ 33,587,017
4815	0.17	€ 273,655	€ 547,311	€ 820,966	##########	-€ 6,616,908	-€ 13,233,816	-€ 19,850,723	-€ 26,467,631
4816	0.21	€ 336,765	€ 673,531	##########	##########	-€ 8,142,888	-€ 16,285,776	-€ 24,428,664	-€ 32,571,552
4817	0.23	€ 366,781	€ 733,562	##########	##########	-€ 8,868,657	-€ 17,737,314	-€ 26,605,971	-€ 35,474,628
4818	0.22	€ 353,718	€ 707,437	##########	##########	-€ 8,552,808	-€ 17,105,616	-€ 25,658,424	-€ 34,211,232
4819	0.18	€ 283,121	€ 566,243	€ 849,364	##########	-€ 6,845,793	-€ 13,691,586	-€ 20,537,379	-€ 27,383,171
4820	0.15	€ 234,045	€ 468,091	€ 702,136	€ 936,181	-€ 5,659,147	-€ 11,318,294	-€ 16,977,441	-€ 22,636,588
4821	0.12	€ 193,488	€ 386,976	€ 580,463	€ 773,951	-€ 4,678,477	-€ 9,356,954	-€ 14,035,431	-€ 18,713,908
4822	0.20	€ 308,399	€ 616,798	€ 925,197	##########	-€ 7,456,993	-€ 14,913,986	-€ 22,370,979	-€ 29,827,972
4823	0.20	€ 313,125	€ 626,250	€ 939,375	##########	-€ 7,571,266	-€ 15,142,533	-€ 22,713,799	-€ 30,285,066
4824	0.20	€ 315,955	€ 631,910	€ 947,865	##########	-€ 7,639,698	-€ 15,279,396	-€ 22,919,094	-€ 30,558,792
4825	0.21	€ 323,210	€ 646,420	€ 969,629	##########	-€ 7,815,116	-€ 15,630,232	-€ 23,445,349	-€ 31,260,465
4826	0.22	€ 352,633	€ 705,265	##########	##########	-€ 8,526,553	-€ 17,053,106	-€ 25,579,660	-€ 34,106,213
4827	0.17	€ 270,911	€ 541,822	€ 812,733	##########	-€ 6,550,544	-€ 13,101,089	-€ 19,651,633	-€ 26,202,177
4828	0.22	€ 345,122	€ 690,245	##########	##########	-€ 8,344,957	-€ 16,689,914	-€ 25,034,871	-€ 33,379,828
4829	0.24	€ 370,402	€ 740,804	##########	##########	-€ 8,956,206	-€ 17,912,412	-€ 26,868,618	-€ 35,824,824
4830	0.18	€ 285,597	€ 571,193	€ 856,790	##########	-€ 6,905,645	-€ 13,811,289	-€ 20,716,934	-€ 27,622,578
4831	0.20	€ 319,118	€ 638,235	€ 957,353	##########	-€ 7,716,172	-€ 15,432,344	-€ 23,148,516	-€ 30,864,688
4832	0.20	€ 311,174	€ 622,349	€ 933,523	##########	-€ 7,524,106	-€ 15,048,212	-€ 22,572,319	-€ 30,096,425
4833	0.13	€ 208,033	€ 416,065	€ 624,098	€ 832,131	-€ 5,030,169	-€ 10,060,339	-€ 15,090,508	-€ 20,120,677
4834	0.20	€ 312,645	€ 625,289	€ 937,934	##########	-€ 7,559,656	-€ 15,119,312	-€ 22,678,967	-€ 30,238,623
4835	0.21	€ 329,860	€ 659,720	€ 989,580	##########	-€ 7,975,916	-€ 15,951,831	-€ 23,927,747	-€ 31,903,662
4836	0.19	€ 305,535	€ 611,071	€ 916,606	##########	-€ 7,387,757	-€ 14,775,513	-€ 22,163,270	-€ 29,551,027
4837	0.18	€ 277,623	€ 555,245	€ 832,868	##########	-€ 6,712,836	-€ 13,425,671	-€ 20,138,507	-€ 26,851,343
4838	0.16	€ 250,630	€ 501,261	€ 751,891	##########	-€ 6,060,169	-€ 12,120,338	-€ 18,180,508	-€ 24,240,677
4839	0.22	€ 353,400	€ 706,799	##########	##########	-€ 8,545,097	-€ 17,090,194	-€ 25,635,291	-€ 34,180,388
4840	0.18	€ 282,985	€ 565,969	€ 848,954	##########	-€ 6,842,482	-€ 13,684,964	-€ 20,527,446	-€ 27,369,927
4841	0.19	€ 306,939	€ 613,877	€ 920,816	##########	-€ 7,421,686	-€ 14,843,373	-€ 22,265,059	-€ 29,686,746
4842	0.12	€ 195,189	€ 390,378	€ 585,567	€ 780,756	-€ 4,719,610	-€ 9,439,221	-€ 14,158,831	-€ 18,878,442
4843	0.23	€ 357,323	€ 714,647	##########	##########	-€ 8,639,973	-€ 17,279,947	-€ 25,919,920	-€ 34,559,894
4844	0.22	€ 340,939	€ 681,877	##########	##########	-€ 8,243,793	-€ 16,487,587	-€ 24,731,380	-€ 32,975,174
4845	0.18	€ 289,588	€ 579,176	€ 868,764	##########	-€ 7,002,151	-€ 14,004,302	-€ 21,006,453	-€ 28,008,604
4846	0.19	€ 299,283	€ 598,567	€ 897,850	##########	-€ 7,236,583	-€ 14,473,167	-€ 21,709,750	-€ 28,946,334
4847	0.17	€ 266,026	€ 532,052	€ 798,078	##########	-€ 6,432,431	-€ 12,864,861	-€ 19,297,292	-€ 25,729,723
4848	0.19	€ 293,260	€ 586,520	€ 879,780	##########	-€ 7,090,938	-€ 14,181,876	-€ 21,272,813	-€ 28,363,751
4849	0.25	€ 390,911	€ 781,821	##########	##########	-€ 9,452,104	-€ 18,904,208	-€ 28,356,312	-€ 37,808,416
4850	0.14	€ 220,113	€ 440,227	€ 660,340	€ 880,454	-€ 5,322,278	-€ 10,644,555	-€ 15,966,833	-€ 21,289,110
4851	0.29	€ 452,469	€ 904,938	##########	##########	-€ 10,940,570	-€ 21,881,141	-€ 32,821,711	-€ 43,762,282
4852	0.25	€ 391,747	€ 783,495	##########	##########	-€ 9,472,334	-€ 18,944,668	-€ 28,417,002	-€ 37,889,336
4853	0.18	€ 287,798	€ 575,596	€ 863,394	##########	-€ 6,958,872	-€ 13,917,744	-€ 20,876,615	-€ 27,835,487
4854	0.15	€ 231,640	€ 463,280	€ 694,920	€ 926,560	-€ 5,600,989	-€ 11,201,977	-€ 16,802,966	-€ 22,403,954
4855	0.17	€ 264,103	€ 528,205	€ 792,308	##########	-€ 6,385,922	-€ 12,771,844	-€ 19,157,766	-€ 25,543,688
4856	0.24	€ 383,757	€ 767,513	##########	##########	-€ 9,279,121	-€ 18,558,243	-€ 27,837,364	-€ 37,116,486
4857	0.18	€ 278,279	€ 556,559	€ 834,838	##########	-€ 6,728,712	-€ 13,457,423	-€ 20,186,135	-€ 26,914,846
4858	0.25	€ 394,470	€ 788,941	##########	##########	-€ 9,538,175	-€ 19,076,351	-€ 28,614,526	-€ 38,152,702
4859	0.18	€ 284,521	€ 569,041	€ 853,562	##########	-€ 6,879,627	-€ 13,759,254	-€ 20,638,881	-€ 27,518,508
4860	0.18	€ 280,051	€ 560,102	€ 840,154	##########	-€ 6,771,556	-€ 13,543,112	-€ 20,314,668	-€ 27,086,224
4861	0.25	€ 386,776	€ 773,552	##########	##########	-€ 9,352,125	-€ 18,704,251	-€ 28,056,376	-€ 37,408,501
4862	0.17	€ 270,065	€ 540,130	€ 810,195	##########	-€ 6,530,093	-€ 13,060,186	-€ 19,590,279	-€ 26,120,372
4863	0.24	€ 372,096	€ 744,192	##########	##########	-€ 8,997,167	-€ 17,994,334	-€ 26,991,502	-€ 35,988,669
4864	0.17	€ 267,211	€ 534,422	€ 801,633	##########	-€ 6,461,080	-€ 12,922,160	-€ 19,383,240	-€ 25,844,319
4865	0.23	€ 367,614	€ 735,228	##########	##########	-€ 8,888,793	-€ 17,777,587	-€ 26,666,380	-€ 35,555,174
4866	0.20	€ 311,659	€ 623,319	€ 934,978	##########	-€ 7,535,829	-€ 15,071,658	-€ 22,607,487	-€ 30,143,317
4867	0.23	€ 360,097	€ 720,194	##########	##########	-€ 8,707,036	-€ 17,414,071	-€ 26,121,107	-€ 34,828,143
4868	0.15	€ 229,493	€ 458,987	€ 688,480	€ 917,973	-€ 5,549,080	-€ 11,098,159	-€ 16,647,239	-€ 22,196,318
4869	0.19	€ 306,465	€ 612,930	€ 919,396	##########	-€ 7,410,238	-€ 14,820,476	-€ 22,230,714	-€ 29,640,952
4870	0.21	€ 336,505	€ 673,009	##########	##########	-€ 8,136,583	-€ 16,273,167	-€ 24,409,750	-€ 32,546,333
4871	0.09	€ 148,495	€ 296,991	€ 445,486	€ 593,982	-€ 3,590,575	-€ 7,181,151	-€ 10,771,726	-€ 14,362,301
4872	0.29	€ 454,665	€ 909,330	##########	##########	-€ 10,993,665	-€ 21,987,330	-€ 32,980,995	-€ 43,974,659
4873	0.16	€ 256,909	€ 513,819	€ 770,728	##########	-€ 6,211,994	-€ 12,423,988	-€ 18,635,982	-€ 24,847,976
4874	0.17	€ 267,680	€ 535,361	€ 803,041	##########	-€ 6,472,434	-€ 12,944,868	-€ 19,417,302	-€ 25,889,736
4875	0.18	€ 282,171	€ 564,343	€ 846,514	##########	-€ 6,822,819	-€ 13,645,638	-€ 20,468,457	-€ 27,291,276
4876	0.11	€ 167,508	€ 335,016	€ 502,525	€ 670,033	-€ 4,050,300	-€ 8,100,599	-€ 12,150,899	-€ 16,201,198
4877	0.22	€ 339,133	€ 678,266	##########	##########	-€ 8,200,130	-€ 16,400,260	-€ 24,600,390	-€ 32,800,520
4878	0.11	€ 173,334	€ 346,669	€ 520,003	€ 693,338	-€ 4,191,176	-€ 8,382,352	-€ 12,573,528	-€ 16,764,704
4879	0.20	€ 309,623	€ 619,246	€ 928,869	##########	-€ 7,486,592	-€ 14,973,183	-€ 22,459,775	-€ 29,946,367
4880	0.18	€ 283,812	€ 567,624	€ 851,436	##########	-€ 6,862,489	-€ 13,724,977	-€ 20,587,466	-€ 27,449,955
4881	0.16	€ 250,246	€ 500,492	€ 750,739	##########	-€ 6,050,880	-€ 12,101,760	-€ 18,152,640	-€ 24,203,519
4882	0.23	€ 368,767	€ 737,534	##########	##########	-€ 8,916,672	-€ 17,833,345	-€ 26,750,017	-€ 35,666,690
4883	0.14	€ 214,549	€ 429,099	€ 643,648	€ 858,198	-€ 5,187,741	-€ 10,375,482	-€ 15,563,223	-€ 20,750,964
4884	0.22	€ 340,610	€ 681,220	##########	##########	-€ 8,235,849	-€ 16,471,698	-€ 24,707,546	-€ 32,943,395
4885	0.20	€ 307,356	€ 614,712	€ 922,069	##########	-€ 7,431,781	-€ 14,863,563	-€ 22,295,344	-€ 29,727,126
4886	0.23	€ 369,022	€ 738,044	##########	##########	-€ 8,922,839	-€ 17,845,679	-€ 26,768,518	-€ 35,691,357
4887	0.17	€ 264,005	€ 528,010	€ 792,016	##########	-€ 6,383,568	-€ 12,767,135	-€ 19,150,703	-€ 25,534,270
4888	0.18	€ 290,145	€ 580,290	€ 870,435	##########	-€ 7,015,621	-€ 14,031,242	-€ 21,046,864	-€ 28,062,485
4889	0.15	€ 239,391	€ 478,781	€ 718,172	€ 957,562	-€ 5,788,393	-€ 11,576,785	-€ 17,365,178	-€ 23,153,571
4890	0.12	€ 191,501	€ 383,001	€ 574,502	€ 766,003	-€ 4,630,428	-€ 9,260,857	-€ 13,891,285	-€ 18,521,714
4891	0.14	€ 222,199	€ 444,398	€ 666,597	€ 888,795	-€ 5,372,702	-€ 10,745,404	-€ 16,118,106	-€ 21,490,808
4892	0.24	€ 384,553	€ 769,105	##########	##########	-€ 9,298,367	-€ 18,596,734	-€ 27,895,101	-€ 37,193,469

4893	0.17	€ 268,939	€ 537,878	€ 806,817	##########	-€ 6,502,863	-€ 13,005,726	-€ 19,508,589	-€ 26,011,452
4894	0.20	€ 316,088	€ 632,177	€ 948,265	##########	-€ 7,642,924	-€ 15,285,848	-€ 22,928,772	-€ 30,571,696
4895	0.23	€ 361,707	€ 723,415	##########	##########	-€ 8,745,974	-€ 17,491,948	-€ 26,237,922	-€ 34,983,897
4896	0.27	€ 424,503	€ 849,006	##########	##########	-€ 10,264,359	-€ 20,528,718	-€ 30,793,078	-€ 41,057,437
4897	0.27	€ 422,172	€ 844,343	##########	##########	-€ 10,207,984	-€ 20,415,968	-€ 30,623,952	-€ 40,831,936
4898	0.14	€ 223,548	€ 447,096	€ 670,645	€ 894,193	-€ 5,405,330	-€ 10,810,659	-€ 16,215,989	-€ 21,621,318
4899	0.16	€ 258,943	€ 517,886	€ 776,829	##########	-€ 6,261,162	-€ 12,522,325	-€ 18,783,487	-€ 25,044,649
4900	0.16	€ 253,224	€ 506,448	€ 759,672	##########	-€ 6,122,882	-€ 12,245,765	-€ 18,368,647	-€ 24,491,529
4901	0.16	€ 258,246	€ 516,491	€ 774,737	##########	-€ 6,244,303	-€ 12,488,606	-€ 18,732,910	-€ 24,977,213
4902	0.23	€ 355,712	€ 711,424	##########	##########	-€ 8,601,007	-€ 17,202,015	-€ 25,803,022	-€ 34,404,029
4903	0.26	€ 407,866	€ 815,731	##########	##########	-€ 9,862,070	-€ 19,724,139	-€ 29,586,209	-€ 39,448,278
4904	0.17	€ 260,622	€ 521,243	€ 781,865	##########	-€ 6,301,752	-€ 12,603,503	-€ 18,905,255	-€ 25,207,007
4905	0.18	€ 281,023	€ 562,046	€ 843,069	##########	-€ 6,795,050	-€ 13,590,100	-€ 20,385,150	-€ 27,180,201
4906	0.16	€ 253,481	€ 506,963	€ 760,444	##########	-€ 6,129,107	-€ 12,258,213	-€ 18,387,320	-€ 24,516,426
4907	0.18	€ 280,529	€ 561,058	€ 841,587	##########	-€ 6,783,107	-€ 13,566,213	-€ 20,349,320	-€ 27,132,426
4908	0.26	€ 403,478	€ 806,956	##########	##########	-€ 9,755,976	-€ 19,511,952	-€ 29,267,928	-€ 39,023,904
4909	0.18	€ 280,713	€ 561,426	€ 842,139	##########	-€ 6,787,558	-€ 13,575,116	-€ 20,362,674	-€ 27,150,232
4910	0.18	€ 281,984	€ 563,968	€ 845,952	##########	-€ 6,818,290	-€ 13,636,581	-€ 20,454,871	-€ 27,273,161
4911	0.23	€ 360,434	€ 720,867	##########	##########	-€ 8,715,176	-€ 17,430,352	-€ 26,145,528	-€ 34,860,704
4912	0.25	€ 396,741	€ 793,483	##########	##########	-€ 9,593,090	-€ 19,186,180	-€ 28,779,270	-€ 38,372,360
4913	0.26	€ 406,179	€ 812,358	##########	##########	-€ 9,821,289	-€ 19,642,578	-€ 29,463,867	-€ 39,285,156
4914	0.25	€ 393,961	€ 787,922	##########	##########	-€ 9,525,864	-€ 19,051,727	-€ 28,577,591	-€ 38,103,455
4915	0.29	€ 448,853	€ 897,706	##########	##########	-€ 10,853,127	-€ 21,706,254	-€ 32,559,381	-€ 43,412,508
4916	0.21	€ 326,538	€ 653,076	€ 979,614	##########	-€ 7,895,592	-€ 15,791,184	-€ 23,686,776	-€ 31,582,368
4917	0.23	€ 361,506	€ 723,011	##########	##########	-€ 8,741,101	-€ 17,482,203	-€ 26,223,304	-€ 34,964,406
4918	0.25	€ 400,951	€ 801,901	##########	##########	-€ 9,694,864	-€ 19,389,728	-€ 29,084,593	-€ 38,779,457
4919	0.18	€ 285,976	€ 571,953	€ 857,929	##########	-€ 6,914,821	-€ 13,829,642	-€ 20,744,463	-€ 27,659,284
4920	0.20	€ 319,838	€ 639,676	€ 959,514	##########	-€ 7,733,586	-€ 15,467,171	-€ 23,200,757	-€ 30,934,343
4921	0.24	€ 370,552	€ 741,105	##########	##########	-€ 8,959,848	-€ 17,919,696	-€ 26,879,543	-€ 35,839,391
4922	0.21	€ 332,902	€ 665,804	€ 998,706	##########	-€ 8,049,471	-€ 16,098,942	-€ 24,148,413	-€ 32,197,883
4923	0.19	€ 303,690	€ 607,380	€ 911,070	##########	-€ 7,343,132	-€ 14,686,265	-€ 22,029,397	-€ 29,372,529
4924	0.20	€ 311,197	€ 622,393	€ 933,590	##########	-€ 7,524,640	-€ 15,049,279	-€ 22,573,919	-€ 30,098,558
4925	0.20	€ 313,709	€ 627,419	€ 941,128	##########	-€ 7,585,402	-€ 15,170,803	-€ 22,756,205	-€ 30,341,606
4926	0.19	€ 298,273	€ 596,546	€ 894,819	##########	-€ 7,212,155	-€ 14,424,309	-€ 21,636,464	-€ 28,848,618
4927	0.19	€ 296,450	€ 592,899	€ 889,349	##########	-€ 7,168,066	-€ 14,336,132	-€ 21,504,198	-€ 28,672,264
4928	0.16	€ 259,373	€ 518,746	€ 778,119	##########	-€ 6,271,563	-€ 12,543,126	-€ 18,814,689	-€ 25,086,251
4929	0.20	€ 308,045	€ 616,091	€ 924,136	##########	-€ 7,448,446	-€ 14,896,893	-€ 22,345,339	-€ 29,793,786
4930	0.13	€ 206,128	€ 412,255	€ 618,383	€ 824,510	-€ 4,984,103	-€ 9,968,206	-€ 14,952,309	-€ 19,936,413
4931	0.21	€ 338,244	€ 676,488	##########	##########	-€ 8,178,645	-€ 16,357,289	-€ 24,535,934	-€ 32,714,579
4932	0.17	€ 261,825	€ 523,650	€ 785,476	##########	-€ 6,330,856	-€ 12,661,712	-€ 18,992,568	-€ 25,323,424
4933	0.14	€ 214,064	€ 428,127	€ 642,191	€ 856,254	-€ 5,175,994	-€ 10,351,988	-€ 15,527,982	-€ 20,703,977
4934	0.18	€ 286,872	€ 573,744	€ 860,616	##########	-€ 6,936,481	-€ 13,872,963	-€ 20,809,444	-€ 27,745,926
4935	0.19	€ 306,666	€ 613,331	€ 919,997	##########	-€ 7,415,083	-€ 14,830,165	-€ 22,245,248	-€ 29,660,330
4936	0.15	€ 228,711	€ 457,421	€ 686,132	€ 914,843	-€ 5,530,156	-€ 11,060,313	-€ 16,590,469	-€ 22,120,626
4937	0.17	€ 275,386	€ 550,772	€ 826,158	##########	-€ 6,658,755	-€ 13,317,511	-€ 19,976,266	-€ 26,635,021
4938	0.23	€ 360,522	€ 721,044	##########	##########	-€ 8,717,318	-€ 17,434,637	-€ 26,151,955	-€ 34,869,274
4939	0.18	€ 283,660	€ 567,320	€ 850,981	##########	-€ 6,858,819	-€ 13,717,638	-€ 20,576,457	-€ 27,435,276
4940	0.17	€ 262,030	€ 524,061	€ 786,091	##########	-€ 6,335,816	-€ 12,671,631	-€ 19,007,447	-€ 25,343,263
4941	0.21	€ 330,126	€ 660,251	€ 990,377	##########	-€ 7,982,342	-€ 15,964,685	-€ 23,947,027	-€ 31,929,369
4942	0.10	€ 155,662	€ 311,324	€ 466,986	€ 622,648	-€ 3,763,862	-€ 7,527,723	-€ 11,291,585	-€ 15,055,446
4943	0.17	€ 265,086	€ 530,172	€ 795,258	##########	-€ 6,409,701	-€ 12,819,402	-€ 19,229,103	-€ 25,638,804
4944	0.20	€ 319,148	€ 638,295	€ 957,443	##########	-€ 7,716,897	-€ 15,433,794	-€ 23,150,692	-€ 30,867,589
4945	0.16	€ 245,418	€ 490,837	€ 736,255	€ 981,673	-€ 5,934,143	-€ 11,868,287	-€ 17,802,430	-€ 23,736,574
4946	0.19	€ 301,861	€ 603,721	€ 905,582	##########	-€ 7,298,900	-€ 14,597,800	-€ 21,896,700	-€ 29,195,600
4947	0.23	€ 369,814	€ 739,628	##########	##########	-€ 8,941,989	-€ 17,883,979	-€ 26,825,968	-€ 35,767,958
4948	0.16	€ 252,929	€ 505,857	€ 758,786	##########	-€ 6,115,737	-€ 12,231,474	-€ 18,347,211	-€ 24,462,948
4949	0.28	€ 437,847	€ 875,695	##########	##########	-€ 10,587,022	-€ 21,174,044	-€ 31,761,066	-€ 42,348,087
4950	0.23	€ 364,703	€ 729,407	##########	##########	-€ 8,818,419	-€ 17,636,839	-€ 26,455,258	-€ 35,273,677
4951	0.22	€ 340,750	€ 681,501	##########	##########	-€ 8,239,240	-€ 16,478,480	-€ 24,717,721	-€ 32,956,961
4952	0.19	€ 306,815	€ 613,630	€ 920,444	##########	-€ 7,418,690	-€ 14,837,381	-€ 22,256,071	-€ 29,674,762
4953	0.15	€ 243,175	€ 486,349	€ 729,524	€ 972,698	-€ 5,879,888	-€ 11,759,775	-€ 17,639,663	-€ 23,519,551
4954	0.19	€ 305,364	€ 610,729	€ 916,093	##########	-€ 7,383,623	-€ 14,767,245	-€ 22,150,868	-€ 29,534,490
4955	0.20	€ 309,651	€ 619,302	€ 928,954	##########	-€ 7,487,273	-€ 14,974,547	-€ 22,461,820	-€ 29,949,093
4956	0.11	€ 167,633	€ 335,266	€ 502,899	€ 670,532	-€ 4,053,318	-€ 8,106,636	-€ 12,159,954	-€ 16,213,271
4957	0.20	€ 313,978	€ 627,956	€ 941,933	##########	-€ 7,591,890	-€ 15,183,779	-€ 22,775,669	-€ 30,367,558
4958	0.17	€ 271,665	€ 543,329	€ 814,994	##########	-€ 6,568,771	-€ 13,137,542	-€ 19,706,313	-€ 26,275,084
4959	0.18	€ 290,519	€ 581,038	€ 871,557	##########	-€ 7,024,659	-€ 14,049,319	-€ 21,073,978	-€ 28,098,638
4960	0.15	€ 230,592	€ 461,184	€ 691,777	€ 922,369	-€ 5,575,651	-€ 11,151,302	-€ 16,726,953	-€ 22,302,604
4961	0.17	€ 274,134	€ 548,268	€ 822,402	##########	-€ 6,628,478	-€ 13,256,957	-€ 19,885,435	-€ 26,513,914
4962	0.14	€ 214,574	€ 429,148	€ 643,722	€ 858,295	-€ 5,188,332	-€ 10,376,664	-€ 15,564,995	-€ 20,753,327
4963	0.11	€ 168,125	€ 336,249	€ 504,374	€ 672,499	-€ 4,065,205	-€ 8,130,410	-€ 12,195,614	-€ 16,260,819
4964	0.27	€ 421,375	€ 842,749	##########	##########	-€ 10,188,715	-€ 20,377,430	-€ 30,566,145	-€ 40,754,860
4965	0.14	€ 217,333	€ 434,666	€ 651,998	€ 869,331	-€ 5,255,042	-€ 10,510,084	-€ 15,765,126	-€ 21,020,168
4966	0.16	€ 251,975	€ 503,950	€ 755,925	##########	-€ 6,092,685	-€ 12,185,369	-€ 18,278,054	-€ 24,370,738
4967	0.22	€ 340,405	€ 680,811	##########	##########	-€ 8,230,904	-€ 16,461,807	-€ 24,692,711	-€ 32,923,615
4968	0.16	€ 259,707	€ 519,414	€ 779,120	##########	-€ 6,279,633	-€ 12,559,266	-€ 18,838,898	-€ 25,118,531
4969	0.17	€ 274,934	€ 549,868	€ 824,803	##########	-€ 6,647,827	-€ 13,295,653	-€ 19,943,480	-€ 26,591,307
4970	0.19	€ 293,108	€ 586,216	€ 879,324	##########	-€ 7,087,265	-€ 14,174,529	-€ 21,261,794	-€ 28,349,059
4971	0.13	€ 198,833	€ 397,665	€ 596,498	€ 795,330	-€ 4,807,714	-€ 9,615,428	-€ 14,423,141	-€ 19,230,855
4972	0.24	€ 370,491	€ 740,982	##########	##########	-€ 8,958,359	-€ 17,916,719	-€ 26,875,078	-€ 35,833,438
4973	0.12	€ 190,330	€ 380,660	€ 570,989	€ 761,319	-€ 4,602,117	-€ 9,204,234	-€ 13,806,351	-€ 18,408,468
4974	0.19	€ 294,866	€ 589,732	€ 884,598	##########	-€ 7,129,774	-€ 14,259,548	-€ 21,389,321	-€ 28,519,095
4975	0.17	€ 272,136	€ 544,271	€ 816,407	##########	-€ 6,580,159	-€ 13,160,317	-€ 19,740,476	-€ 26,320,635
4976	0.16	€ 258,970	€ 517,941	€ 776,911	##########	-€ 6,261,828	-€ 12,523,655	-€ 18,785,483	-€ 25,047,310
4977	0.17	€ 260,557	€ 521,114	€ 781,670	##########	-€ 6,300,186	-€ 12,600,371	-€ 18,900,557	-€ 25,200,743
4978	0.21	€ 335,428	€ 670,857	##########	##########	-€ 8,110,560	-€ 16,221,120	-€ 24,331,680	-€ 32,442,240
4979	0.22	€ 348,818	€ 697,636	##########	##########	-€ 8,434,314	-€ 16,868,629	-€ 25,302,943	-€ 33,737,258
4980	0.18	€ 285,219	€ 570,437	€ 855,656	##########	-€ 6,896,504	-€ 13,793,008	-€ 20,689,513	-€ 27,586,017
4981	0.22	€ 351,029	€ 702,057	##########	##########	-€ 8,487,770	-€ 16,975,539	-€ 25,463,309	-€ 33,951,078
4982	0.12	€ 183,860	€ 367,720	€ 551,579	€ 735,439	-€ 4,445,675	-€ 8,891,350	-€ 13,337,025	-€ 17,782,701
4983	0.21	€ 332,826	€ 665,653	€ 998,479	##########	-€ 8,047,643	-€ 16,095,286	-€ 24,142,929	-€ 32,190,571
4984	0.18	€ 280,001	€ 560,002	€ 840,002	##########	-€ 6,770,336	-€ 13,540,672	-€ 20,311,009	-€ 27,081,345
4985	0.20	€ 309,863	€ 619,727	€ 929,590	##########	-€ 7,492,403	-€ 14,984,806	-€ 22,477,209	-€ 29,969,612
4986	0.15	€ 240,273	€ 480,547	€ 720,820	€ 961,094	-€ 5,809,739	-€ 11,619,479	-€ 17,429,218	-€ 23,238,957
4987	0.20	€ 310,636	€ 621,272	€ 931,908	##########	-€ 7,511,083	-€ 15,022,166	-€ 22,533,249	-€ 30,044,333
4988	0.27	€ 418,160	€ 836,320	##########	##########	-€ 10,110,985	-€ 20,221,970	-€ 30,332,955	-€ 40,443,940
4989	0.17	€ 268,487	€ 536,974	€ 805,461	##########	-€ 6,491,939	-€ 12,983,879	-€ 19,475,818	-€ 25,967,757
4990	0.17	€ 273,667	€ 547,335	€ 821,002	##########	-€ 6,617,195	-€ 13,234,389	-€ 19,851,584	-€ 26,468,779
4991	0.24	€ 374,618	€ 749,235	##########	##########	-€ 9,058,146	-€ 18,116,292	-€ 27,174,437	-€ 36,232,583
4992	0.20	€ 320,975	€ 641,950	€ 962,925	##########	-€ 7,761,081	-€ 15,522,162	-€ 23,283,243	-€ 31,044,324
4993	0.19	€ 292,599	€ 585,197	€ 877,796	##########	-€ 7,074,946	-€ 14,149,892	-€ 21,224,838	-€ 28,299,785
4994	0.20	€ 322,611	€ 645,222	€ 967,833	##########	-€ 7,800,639	-€ 15,601,278	-€ 23,401,918	-€ 31,202,557

4995	0.22	€ 343,036	€ 686,071	##########	##########	-€ 8,294,502	-€ 16,589,004	-€ 24,883,505	-€ 33,178,007
4996	0.27	€ 431,763	€ 863,527	##########	##########	-€ 10,439,913	-€ 20,879,826	-€ 31,319,738	-€ 41,759,651
4997	0.21	€ 338,281	€ 676,562	##########	##########	-€ 8,179,537	-€ 16,359,073	-€ 24,538,610	-€ 32,718,146
4998	0.21	€ 330,378	€ 660,757	€ 991,135	##########	-€ 7,988,451	-€ 15,976,902	-€ 23,965,353	-€ 31,953,803
4999	0.23	€ 362,938	€ 725,876	##########	##########	-€ 8,775,739	-€ 17,551,478	-€ 26,327,217	-€ 35,102,956
5000	0.15	€ 242,467	€ 484,934	€ 727,402	€ 969,869	-€ 5,862,785	-€ 11,725,571	-€ 17,588,356	-€ 23,451,142
5000	0.16	€ 252,376	€ 504,752	€ 757,127	##########	-€ 6,102,371	-€ 12,204,742	-€ 18,307,114	-€ 24,409,485

Sensitivity Analysis

Number aCTGs under 1 indication in 2020	**21375**

Implementation rates of MLC-aCTG	*Actual costs*	*Budget impact: actual costs*
0% (reference scenario OLC-aCTG)	€ 5,020,892	NA
25%	€ 5,253,927	€ 233,035
50%	€ 5,486,962	€ 466,070
75%	€ 5,719,997	€ 699,105
100%	€ 5,953,032	€ 932,140

	Reimbursed costs	*Budget impact: reimbursed costs*
0% (reference scenario OLC-aCTG)	€ 31,891,792	NA
25%	€ 26,257,077	-€ 5,634,715
50%	€ 20,622,362	-€ 11,269,431
75%	€ 14,987,646	-€ 16,904,146
100%	€ 9,352,931	-€ 22,538,862

**PSA**	**0**
**Prev**	**0.15**

Iterations	Prevalence	Actual costs by implementation rate 25%	50%	75%	100%	Reimbursed costs by implementation rate 25%	50%	75%	100%
1	0.15	########	########	€ 699,105	€ 932,140	-€ 5,634,715	###########	###########	###########
2	0.20	########	########	€ 929,243	#########	-€ 7,489,607	###########	###########	###########
3	0.16	########	########	€ 756,185	#########	-€ 6,094,775	###########	###########	###########
4	0.17	########	########	€ 799,807	#########	-€ 6,446,364	###########	###########	###########
5	0.14	########	########	€ 657,165	€ 876,220	-€ 5,296,686	###########	###########	###########
6	0.18	########	########	€ 828,978	#########	-€ 6,681,481	###########	###########	###########
7	0.19	########	########	€ 899,555	#########	-€ 7,250,320	###########	###########	###########
8	0.15	########	########	€ 722,549	€ 963,398	-€ 5,823,670	###########	###########	###########
9	0.17	########	########	€ 818,544	#########	-€ 6,597,385	###########	###########	###########
10	0.11	########	########	€ 527,181	€ 702,908	-€ 4,249,028	-€ 8,498,057	###########	###########
11	0.14	########	########	€ 646,327	€ 861,769	-€ 5,209,331	###########	###########	###########
12	0.17	########	########	€ 814,190	#########	-€ 6,562,294	###########	###########	###########
13	0.09	########	########	€ 424,836	€ 566,448	-€ 3,424,138	-€ 6,848,276	###########	###########
14	0.16	########	########	€ 776,708	#########	-€ 6,260,186	###########	###########	###########
15	0.11	########	########	€ 528,996	€ 705,328	-€ 4,263,657	-€ 8,527,315	###########	###########
16	0.21	########	########	€ 992,275	#########	-€ 7,997,634	###########	###########	###########
17	0.20	########	########	€ 926,023	#########	-€ 7,463,651	###########	###########	###########
18	0.17	########	########	€ 813,806	#########	-€ 6,559,197	###########	###########	###########
19	0.16	########	########	€ 759,546	#########	-€ 6,121,869	###########	###########	###########
20	0.14	########	########	€ 649,531	€ 866,041	-€ 5,235,152	###########	###########	###########
21	0.14	########	########	€ 675,723	€ 900,964	-€ 5,446,261	###########	###########	###########
22	0.20	########	########	€ 927,412	#########	-€ 7,474,851	###########	###########	###########
23	0.22	########	########	#########	#########	-€ 8,265,815	###########	###########	###########
24	0.13	########	########	€ 613,994	€ 818,658	-€ 4,948,728	-€ 9,897,456	###########	###########
25	0.16	########	########	€ 767,847	#########	-€ 6,188,773	###########	###########	###########
26	0.18	########	########	€ 849,602	#########	-€ 6,847,705	###########	###########	###########
27	0.15	########	########	€ 704,090	€ 938,786	-€ 5,674,894	###########	###########	###########
28	0.21	########	########	#########	#########	-€ 8,064,898	###########	###########	###########
29	0.12	########	########	€ 553,720	€ 738,293	-€ 4,462,926	-€ 8,925,852	###########	###########
30	0.15	########	########	€ 697,839	€ 930,452	-€ 5,624,516	###########	###########	###########
31	0.15	########	########	€ 686,096	€ 914,794	-€ 5,529,864	###########	###########	###########
32	0.10	########	########	€ 457,632	€ 610,176	-€ 3,688,468	-€ 7,376,936	###########	###########
33	0.21	########	########	€ 990,753	#########	-€ 7,985,368	###########	###########	###########
34	0.19	########	########	€ 894,443	#########	-€ 7,209,121	###########	###########	###########
35	0.17	########	########	€ 782,102	#########	-€ 6,303,666	###########	###########	###########
36	0.13	########	########	€ 631,981	€ 842,641	-€ 5,093,704	###########	###########	###########
37	0.14	########	########	€ 665,270	€ 887,027	-€ 5,362,012	###########	###########	###########
38	0.13	########	########	€ 592,798	€ 790,397	-€ 4,777,893	-€ 9,555,786	###########	###########
39	0.19	########	########	€ 886,810	#########	-€ 7,147,603	###########	###########	###########
40	0.15	########	########	€ 730,523	€ 974,030	-€ 5,887,941	###########	###########	###########
41	0.13	########	########	€ 618,563	€ 824,751	-€ 4,985,559	-€ 9,971,117	###########	###########
42	0.24	########	########	#########	#########	-€ 9,248,378	###########	###########	###########
43	0.16	########	########	€ 756,688	#########	-€ 6,098,829	###########	###########	###########
44	0.19	########	########	€ 893,078	#########	-€ 7,198,123	###########	###########	###########
45	0.18	########	########	€ 826,808	#########	-€ 6,663,989	###########	###########	###########
46	0.18	########	########	€ 845,149	#########	-€ 6,811,821	###########	###########	###########
47	0.15	########	########	€ 711,400	€ 948,533	-€ 5,733,812	###########	###########	###########
48	0.21	########	########	#########	#########	-€ 8,120,347	###########	###########	###########
49	0.17	########	########	€ 823,661	#########	-€ 6,638,627	###########	###########	###########
50	0.12	########	########	€ 585,685	€ 780,913	-€ 4,720,560	-€ 9,441,119	###########	###########
51	0.15	########	########	€ 709,462	€ 945,949	-€ 5,718,190	###########	###########	###########
52	0.13	########	########	€ 604,941	€ 806,589	-€ 4,875,768	-€ 9,751,537	###########	###########
53	0.14	########	########	€ 655,477	€ 873,970	-€ 5,283,082	###########	###########	###########
54	0.19	########	########	€ 912,655	#########	-€ 7,355,910	###########	###########	###########
55	0.18	########	########	€ 849,961	#########	-€ 6,850,603	###########	###########	###########
56	0.15	########	########	€ 727,672	€ 970,229	-€ 5,864,962	###########	###########	###########
57	0.23	########	########	#########	#########	-€ 8,571,931	###########	###########	###########
58	0.20	########	########	€ 934,735	#########	-€ 7,533,873	###########	###########	###########
59	0.16	########	########	€ 764,675	#########	-€ 6,163,203	###########	###########	###########
60	0.16	########	########	€ 741,865	€ 989,153	-€ 5,979,355	###########	###########	###########
61	0.19	########	########	€ 894,378	#########	-€ 7,208,597	###########	###########	###########
62	0.21	########	########	€ 975,995	#########	-€ 7,866,422	###########	###########	###########
63	0.15	########	########	€ 713,430	€ 951,240	-€ 5,750,177	###########	###########	###########
64	0.14	########	########	€ 637,785	€ 850,380	-€ 5,140,483	###########	###########	###########
65	0.14	########	########	€ 662,731	€ 883,641	-€ 5,341,545	###########	###########	###########
66	0.10	########	########	€ 455,507	€ 607,343	-€ 3,671,340	-€ 7,342,681	###########	###########
67	0.22	########	########	#########	#########	-€ 8,485,774	###########	###########	###########
68	0.16	########	########	€ 758,422	#########	-€ 6,112,805	###########	###########	###########
69	0.11	########	########	€ 528,856	€ 705,141	-€ 4,262,525	-€ 8,525,050	###########	###########
70	0.15	########	########	€ 707,622	€ 943,496	-€ 5,703,362	###########	###########	###########
71	0.11	########	########	€ 534,867	€ 713,156	-€ 4,310,976	-€ 8,621,952	###########	###########
72	0.21	########	########	€ 993,573	#########	-€ 8,008,102	###########	###########	###########
73	0.21	########	########	€ 992,284	#########	-€ 7,997,709	###########	###########	###########
74	0.17	########	########	€ 816,059	#########	-€ 6,577,354	###########	###########	###########
75	0.14	########	########	€ 684,420	€ 912,560	-€ 5,516,357	###########	###########	###########
76	0.13	########	########	€ 603,432	€ 804,576	-€ 4,863,604	-€ 9,727,208	###########	###########
77	0.19	########	########	€ 894,687	#########	-€ 7,211,089	###########	###########	###########
78	0.20	########	########	€ 960,125	#########	-€ 7,738,516	###########	###########	###########
79	0.17	########	########	€ 802,961	#########	-€ 6,471,785	###########	###########	###########
80	0.23	########	########	#########	#########	-€ 8,672,600	###########	###########	###########
81	0.28	########	########	#########	#########	###########	###########	###########	###########
82	0.16	########	########	€ 765,387	#########	-€ 6,168,943	###########	###########	###########
83	0.12	########	########	€ 552,898	€ 737,197	-€ 4,456,301	-€ 8,912,603	###########	###########
84	0.13	########	########	€ 633,095	€ 844,127	-€ 5,102,687	###########	###########	###########
85	0.17	########	########	€ 780,483	#########	-€ 6,290,613	###########	###########	###########
86	0.17	########	########	€ 826,605	#########	-€ 6,662,357	###########	###########	###########
87	0.16	########	########	€ 779,131	#########	-€ 6,279,718	###########	###########	###########
88	0.20	########	########	€ 951,219	#########	-€ 7,666,735	###########	###########	###########
89	0.18	########	########	€ 840,081	#########	-€ 6,770,966	###########	###########	###########
90	0.13	########	########	€ 622,066	€ 829,422	-€ 5,013,794	###########	###########	###########
91	0.16	########	########	€ 764,170	#########	-€ 6,159,135	###########	###########	###########
92	0.13	########	########	€ 602,285	€ 803,047	-€ 4,854,360	-€ 9,708,719	###########	###########
93	0.11	########	########	€ 530,883	€ 707,844	-€ 4,278,867	-€ 8,557,733	###########	###########
94	0.15	########	########	€ 707,351	€ 943,135	-€ 5,701,181	###########	###########	###########
95	0.17	########	########	€ 814,079	#########	-€ 6,561,393	###########	###########	###########
96	0.15	########	########	€ 712,743	€ 950,324	-€ 5,744,639	###########	###########	###########
97	0.10	########	########	€ 466,757	€ 622,342	-€ 3,762,012	-€ 7,524,024	###########	###########
98	0.19	########	########	€ 909,356	#########	-€ 7,329,317	###########	###########	###########
99	0.17	########	########	€ 810,187	#########	-€ 6,530,030	###########	###########	###########
100	0.21	########	########	€ 995,340	#########	-€ 8,022,345	###########	###########	###########
101	0.19	########	########	€ 904,365	#########	-€ 7,289,090	###########	###########	###########
102	0.11	########	########	€ 535,008	€ 713,345	-€ 4,312,115	-€ 8,624,231	###########	###########
103	0.12	########	########	€ 547,985	€ 730,646	-€ 4,416,704	-€ 8,833,407	###########	###########
104	0.18	########	########	€ 837,289	#########	-€ 6,748,467	###########	###########	###########
105	0.19	########	########	€ 884,058	#########	-€ 7,125,417	###########	###########	###########
106	0.15	########	########	€ 685,399	€ 913,866	-€ 5,524,249	###########	###########	###########
107	0.15	########	########	€ 724,124	€ 965,499	-€ 5,836,369	###########	###########	###########
108	0.11	########	########	€ 538,174	€ 717,566	-€ 4,337,632	-€ 8,675,264	###########	###########
109	0.12	########	########	€ 544,228	€ 725,637	-€ 4,386,424	-€ 8,772,847	###########	###########
110	0.13	########	########	€ 631,184	€ 841,579	-€ 5,087,280	###########	###########	###########
111	0.11	########	########	€ 535,280	€ 713,707	-€ 4,314,308	-€ 8,628,615	###########	###########
112	0.20	########	########	€ 949,588	#########	-€ 7,653,588	###########	###########	###########
113	0.15	########	########	€ 731,821	€ 975,761	-€ 5,898,402	###########	###########	###########
114	0.16	########	########	€ 733,655	€ 978,206	-€ 5,913,185	###########	###########	###########
115	0.11	########	########	€ 508,576	€ 678,102	-€ 4,099,074	-€ 8,198,148	###########	###########
116	0.25	########	########	#########	#########	-€ 9,445,338	###########	###########	###########
117	0.20	########	########	€ 935,769	#########	-€ 7,542,206	###########	###########	###########
118	0.10	########	########	€ 457,403	€ 609,870	-€ 3,686,622	-€ 7,373,243	###########	###########
119	0.12	########	########	€ 590,149	€ 786,866	-€ 4,756,545	-€ 9,513,090	###########	###########
120	0.17	########	########	€ 799,323	#########	-€ 6,442,463	###########	###########	###########
121	0.12	########	########	€ 557,551	€ 743,402	-€ 4,493,808	-€ 8,987,615	###########	###########
122	0.16	########	########	€ 761,587	#########	-€ 6,138,318	###########	###########	###########
123	0.16	########	########	€ 771,462	#########	-€ 6,217,910	###########	###########	###########
124	0.17	########	########	€ 787,740	#########	-€ 6,349,107	###########	###########	###########
125	0.16	########	########	€ 742,633	€ 990,177	-€ 5,985,548	###########	###########	###########
126	0.19	########	########	€ 907,359	#########	-€ 7,313,225	###########	###########	###########
127	0.17	########	########	€ 788,069	#########	-€ 6,351,760	###########	###########	###########
128	0.11	########	########	€ 532,108	€ 709,478	-€ 4,288,741	-€ 8,577,483	###########	###########
129	0.17	########	########	€ 783,993	#########	-€ 6,318,909	###########	###########	###########
130	0.15	########	########	€ 690,367	€ 920,490	-€ 5,564,293	###########	###########	###########
131	0.22	########	########	#########	#########	-€ 8,284,327	###########	###########	###########
132	0.16	########	########	€ 733,563	€ 978,084	-€ 5,912,443	###########	###########	###########
133	0.20	########	########	€ 923,976	#########	-€ 7,447,155	###########	###########	###########
134	0.15	########	########	€ 706,562	€ 942,083	-€ 5,694,821	###########	###########	###########
135	0.18	########	########	€ 838,430	#########	-€ 6,757,666	###########	###########	###########
136	0.14	########	########	€ 672,065	€ 896,087	-€ 5,416,778	###########	###########	###########
137	0.14	########	########	€ 641,792	€ 855,722	-€ 5,172,777	###########	###########	###########
138	0.19	########	########	€ 874,898	#########	-€ 7,051,595	###########	###########	###########
139	0.21	########	########	€ 976,367	#########	-€ 7,869,423	###########	###########	###########
140	0.19	########	########	€ 885,601	#########	-€ 7,137,855	###########	###########	###########
141	0.14	########	########	€ 644,687	€ 859,582	-€ 5,196,110	###########	###########	###########
142	0.17	########	########	€ 802,964	#########	-€ 6,471,807	###########	###########	###########
143	0.17	########	########	€ 798,098	#########	-€ 6,432,594	###########	###########	###########
144	0.13	########	########	€ 615,830	€ 821,107	-€ 4,963,532	-€ 9,927,063	###########	###########
145	0.19	########	########	€ 880,164	#########	-€ 7,094,034	###########	###########	###########
146	0.19	########	########	€ 919,194	#########	-€ 7,408,609	###########	###########	###########
147	0.16	########	########	€ 737,814	€ 983,753	-€ 5,946,711	###########	###########	###########
148	0.14	########	########	€ 655,036	€ 873,381	-€ 5,279,521	###########	###########	###########
149	0.12	########	########	€ 559,843	€ 746,457	-€ 4,512,277	-€ 9,024,554	###########	###########
150	0.10	########	########	€ 473,067	€ 630,756	-€ 3,812,873	-€ 7,625,745	###########	###########
151	0.19	########	########	€ 906,853	#########	-€ 7,309,145	###########	###########	###########
152	0.18	########	########	€ 865,265	#########	-€ 6,973,948	###########	###########	###########
153	0.18	########	########	€ 845,644	#########	-€ 6,815,810	###########	###########	###########
154	0.12	########	########	€ 561,632	€ 748,843	-€ 4,526,701	-€ 9,053,401	###########	###########
155	0.16	########	########	€ 773,288	#########	-€ 6,232,628	###########	###########	###########
156	0.16	########	########	€ 775,178	#########	-€ 6,247,858	###########	###########	###########
157	0.15	########	########	€ 718,998	€ 958,663	-€ 5,795,050	###########	###########	###########
158	0.22	########	########	#########	#########	-€ 8,497,642	###########	###########	###########
159	0.13	########	########	€ 634,249	€ 845,666	-€ 5,111,986	###########	###########	###########
160	0.13	########	########	€ 604,506	€ 806,008	-€ 4,872,259	-€ 9,744,518	###########	###########
161	0.14	########	########	€ 678,694	€ 904,925	-€ 5,470,207	###########	###########	###########
162	0.14	########	########	€ 641,645	€ 855,527	-€ 5,171,596	###########	###########	###########
163	0.09	########	########	€ 402,288	€ 536,384	-€ 3,242,404	-€ 6,484,809	-€ 9,727,213	###########
164	0.16	########	########	€ 756,245	#########	-€ 6,095,260	###########	###########	###########
165	0.15	########	########	€ 704,901	€ 939,869	-€ 5,681,436	###########	###########	###########
166	0.20	########	########	€ 961,488	#########	-€ 7,749,495	###########	###########	###########
167	0.18	########	########	€ 865,911	#########	-€ 6,979,157	###########	###########	###########
168	0.15	########	########	€ 717,184	€ 956,246	-€ 5,780,436	###########	###########	###########

**min_prevalence**	**0.06**
**max_prevalence**	**0.34**

**25% actual costs**	**2.5 percentile**	**€ 149,260**
	**97.5 percentile**	**€ 372,683**
**50% actual costs**	**2.5 percentile**	**€ 298,520**
	**97.5 percentile**	**€ 745,366**
**75% actual costs**	**2.5 percentile**	**€ 447,781**
	**97.5 percentile**	**#########**
**100% actual costs**	**2.5 percentile**	**€ 597,041**
	**97.5 percentile**	**#########**

**25% reiml**	**2.5 percentile**	**-€ 9,011,368**
	**97.5 percentile**	**-€ 3,609,068**
**50% reiml**	**2.5 percentile**	**###########**
	**97.5 percentile**	**-€ 7,218,136**
**75% reiml**	**2.5 percentile**	**###########**
	**97.5 percentile**	**###########**
**100% reir**	**2.5 percentile**	**###########**
	**97.5 percentile**	**###########**

169	0.12	########	########	€ 563,468	€ 751,290	-€ 4,541,495	-€ 9,082,989	###########	###########
170	0.19	########	########	€ 916,072	#########	-€ 7,383,446	###########	###########	###########
171	0.11	########	########	€ 499,948	€ 666,598	-€ 4,029,535	-€ 8,059,070	###########	###########
172	0.14	########	########	€ 658,983	€ 878,644	-€ 5,311,338	###########	###########	###########
173	0.21	########	########	#########	#########	-€ 8,099,770	###########	###########	###########
174	0.15	########	########	€ 725,366	€ 967,155	-€ 5,846,379	###########	###########	###########
175	0.21	########	########	#########	#########	-€ 8,146,614	###########	###########	###########
176	0.16	########	########	€ 778,964	#########	-€ 6,278,377	###########	###########	###########
177	0.15	########	########	€ 702,667	€ 936,890	-€ 5,663,429	###########	###########	###########
178	0.20	########	########	€ 946,305	#########	-€ 7,627,123	###########	###########	###########
179	0.20	########	########	€ 955,501	#########	-€ 7,701,242	###########	###########	###########
180	0.12	########	########	€ 566,824	€ 755,765	-€ 4,568,543	-€ 9,137,086	###########	###########
181	0.17	########	########	€ 798,392	#########	-€ 6,434,959	###########	###########	###########
182	0.13	########	########	€ 608,316	€ 811,089	-€ 4,902,970	-€ 9,805,941	###########	###########
183	0.12	########	########	€ 590,212	€ 786,949	-€ 4,757,047	-€ 9,514,095	###########	###########
184	0.21	########	########	#########	#########	-€ 8,155,345	###########	###########	###########
185	0.13	########	########	€ 596,600	€ 795,467	-€ 4,808,537	-€ 9,617,074	###########	###########
186	0.15	########	########	€ 691,161	€ 921,548	-€ 5,570,691	###########	###########	###########
187	0.15	########	########	€ 687,988	€ 917,318	-€ 5,545,116	###########	###########	###########
188	0.16	########	########	€ 777,732	#########	-€ 6,268,440	###########	###########	###########
189	0.12	########	########	€ 588,021	€ 784,028	-€ 4,739,389	-€ 9,478,777	###########	###########
190	0.10	########	########	€ 487,129	€ 649,506	-€ 3,926,215	-€ 7,852,430	###########	###########
191	0.16	########	########	€ 746,250	€ 995,000	-€ 6,014,700	###########	###########	###########
192	0.14	########	########	€ 662,396	€ 883,195	-€ 5,338,849	###########	###########	###########
193	0.17	########	########	€ 798,406	#########	-€ 6,435,075	###########	###########	###########
194	0.21	########	########	€ 972,597	#########	-€ 7,839,032	###########	###########	###########
195	0.11	########	########	€ 504,886	€ 673,181	-€ 4,069,329	-€ 8,138,658	###########	###########
196	0.19	########	########	€ 910,214	#########	-€ 7,336,232	###########	###########	###########
197	0.14	########	########	€ 674,734	€ 899,645	-€ 5,438,286	###########	###########	###########
198	0.17	########	########	€ 794,882	#########	-€ 6,406,670	###########	###########	###########
199	0.17	########	########	€ 802,277	#########	-€ 6,466,272	###########	###########	###########
200	0.18	########	########	€ 857,913	#########	-€ 6,914,692	###########	###########	###########
201	0.21	########	########	#########	#########	-€ 8,068,129	###########	###########	###########
202	0.18	########	########	€ 844,063	#########	-€ 6,803,067	###########	###########	###########
203	0.12	########	########	€ 580,731	€ 774,308	-€ 4,680,634	-€ 9,361,268	###########	###########
204	0.11	########	########	€ 509,859	€ 679,812	-€ 4,109,415	-€ 8,218,830	###########	###########
205	0.15	########	########	€ 709,671	€ 946,228	-€ 5,719,880	###########	###########	###########
206	0.13	########	########	€ 606,668	€ 808,890	-€ 4,889,682	-€ 9,779,365	###########	###########
207	0.15	########	########	€ 692,163	€ 922,885	-€ 5,578,769	###########	###########	###########
208	0.09	########	########	€ 433,037	€ 577,383	-€ 3,490,237	-€ 6,980,473	###########	###########
209	0.15	########	########	€ 703,796	€ 938,395	-€ 5,672,528	###########	###########	###########
210	0.18	########	########	€ 844,496	#########	-€ 6,806,555	###########	###########	###########
211	0.21	########	########	#########	#########	-€ 8,155,620	###########	###########	###########
212	0.19	########	########	€ 919,711	#########	-€ 7,412,779	###########	###########	###########
213	0.12	########	########	€ 583,857	€ 778,476	-€ 4,705,829	-€ 9,411,659	###########	###########
214	0.16	########	########	€ 735,856	€ 981,142	-€ 5,930,929	###########	###########	###########
215	0.14	########	########	€ 679,696	€ 906,261	-€ 5,478,282	###########	###########	###########
216	0.16	########	########	€ 767,826	#########	-€ 6,188,601	###########	###########	###########
217	0.16	########	########	€ 773,473	#########	-€ 6,234,112	###########	###########	###########
218	0.15	########	########	€ 685,620	€ 914,160	-€ 5,526,029	###########	###########	###########
219	0.15	########	########	€ 713,283	€ 951,044	-€ 5,748,989	###########	###########	###########
220	0.22	########	########	#########	#########	-€ 8,353,056	###########	###########	###########
221	0.08	########	########	€ 375,314	€ 500,418	-€ 3,024,992	-€ 6,049,985	-€ 9,074,977	###########
222	0.10	########	########	€ 487,304	€ 649,739	-€ 3,927,623	-€ 7,855,247	###########	###########
223	0.10	########	########	€ 466,992	€ 622,656	-€ 3,763,910	-€ 7,527,820	###########	###########
224	0.13	########	########	€ 596,802	€ 795,736	-€ 4,810,168	-€ 9,620,336	###########	###########
225	0.20	########	########	€ 936,678	#########	-€ 7,549,529	###########	###########	###########
226	0.14	########	########	€ 651,119	€ 868,159	-€ 5,247,958	###########	###########	###########
227	0.17	########	########	€ 811,253	#########	-€ 6,538,622	###########	###########	###########
228	0.14	########	########	€ 656,479	€ 875,306	-€ 5,291,159	###########	###########	###########
229	0.14	########	########	€ 676,076	€ 901,435	-€ 5,449,105	###########	###########	###########
230	0.15	########	########	€ 689,033	€ 918,711	-€ 5,553,540	###########	###########	###########
231	0.12	########	########	€ 588,669	€ 784,892	-€ 4,744,615	-€ 9,489,229	###########	###########
232	0.20	########	########	€ 949,573	#########	-€ 7,653,461	###########	###########	###########
233	0.20	########	########	€ 963,114	#########	-€ 7,762,603	###########	###########	###########
234	0.15	########	########	€ 694,889	€ 926,518	-€ 5,600,733	###########	###########	###########
235	0.25	########	########	#########	#########	-€ 9,526,060	###########	###########	###########
236	0.11	########	########	€ 541,261	€ 721,681	-€ 4,362,510	-€ 8,725,021	###########	###########
237	0.13	########	########	€ 618,010	€ 824,013	-€ 4,981,100	-€ 9,962,200	###########	###########
238	0.14	########	########	€ 672,017	€ 896,023	-€ 5,416,394	###########	###########	###########
239	0.10	########	########	€ 490,115	€ 653,486	-€ 3,950,277	-€ 7,900,554	###########	###########
240	0.17	########	########	€ 786,623	#########	-€ 6,340,103	###########	###########	###########
241	0.12	########	########	€ 588,124	€ 784,165	-€ 4,740,220	-€ 9,480,440	###########	###########
242	0.17	########	########	€ 821,859	#########	-€ 6,624,106	###########	###########	###########
243	0.09	########	########	€ 441,436	€ 588,581	-€ 3,557,929	-€ 7,115,859	###########	###########
244	0.12	########	########	€ 567,850	€ 757,133	-€ 4,576,815	-€ 9,153,631	###########	###########
245	0.19	########	########	€ 894,220	#########	-€ 7,207,325	###########	###########	###########
246	0.15	########	########	€ 693,193	€ 924,257	-€ 5,587,068	###########	###########	###########
247	0.14	########	########	€ 650,503	€ 867,337	-€ 5,242,986	###########	###########	###########
248	0.19	########	########	€ 882,533	#########	-€ 7,113,131	###########	###########	###########
249	0.13	########	########	€ 623,918	€ 831,890	-€ 5,028,715	###########	###########	###########
250	0.09	########	########	€ 447,802	€ 597,069	-€ 3,609,236	-€ 7,218,473	###########	###########
251	0.13	########	########	€ 627,245	€ 836,327	-€ 5,055,532	###########	###########	###########
252	0.15	########	########	€ 729,944	€ 973,259	-€ 5,883,278	###########	###########	###########
253	0.12	########	########	€ 573,811	€ 765,082	-€ 4,624,861	-€ 9,249,722	###########	###########
254	0.20	########	########	€ 934,698	#########	-€ 7,533,570	###########	###########	###########
255	0.13	########	########	€ 603,781	€ 805,042	-€ 4,866,417	-€ 9,732,835	###########	###########
256	0.10	########	########	€ 485,693	€ 647,591	-€ 3,914,638	-€ 7,829,276	###########	###########
257	0.21	########	########	€ 984,320	#########	-€ 7,933,524	###########	###########	###########
258	0.22	########	########	#########	#########	-€ 8,421,296	###########	###########	###########
259	0.17	########	########	€ 790,585	#########	-€ 6,372,037	###########	###########	###########
260	0.13	########	########	€ 593,645	€ 791,527	-€ 4,784,722	-€ 9,569,444	###########	###########
261	0.17	########	########	€ 817,772	#########	-€ 6,591,160	###########	###########	###########
262	0.16	########	########	€ 765,405	#########	-€ 6,169,088	###########	###########	###########
263	0.16	########	########	€ 752,939	#########	-€ 6,068,615	###########	###########	###########
264	0.14	########	########	€ 656,556	€ 875,408	-€ 5,291,774	###########	###########	###########
265	0.22	########	########	#########	#########	-€ 8,554,991	###########	###########	###########
266	0.21	########	########	€ 997,123	#########	-€ 8,036,714	###########	###########	###########
267	0.18	########	########	€ 867,438	#########	-€ 6,991,464	###########	###########	###########
268	0.14	########	########	€ 645,568	€ 860,758	-€ 5,203,217	###########	###########	###########
269	0.20	########	########	€ 960,136	#########	-€ 7,738,598	###########	###########	###########
270	0.13	########	########	€ 614,762	€ 819,683	-€ 4,954,921	-€ 9,909,841	###########	###########
271	0.14	########	########	€ 642,357	€ 856,476	-€ 5,177,334	###########	###########	###########
272	0.20	########	########	€ 955,333	#########	-€ 7,699,892	###########	###########	###########
273	0.17	########	########	€ 805,927	#########	-€ 6,495,688	###########	###########	###########
274	0.16	########	########	€ 748,797	€ 998,396	-€ 6,035,230	###########	###########	###########
275	0.18	########	########	€ 864,172	#########	-€ 6,965,142	###########	###########	###########
276	0.13	########	########	€ 636,571	€ 848,761	-€ 5,130,697	###########	###########	###########
277	0.18	########	########	€ 859,459	#########	-€ 6,927,151	###########	###########	###########
278	0.15	########	########	€ 694,248	€ 925,664	-€ 5,595,572	###########	###########	###########
279	0.14	########	########	€ 647,600	€ 863,467	-€ 5,219,593	###########	###########	###########
280	0.10	########	########	€ 482,316	€ 643,088	-€ 3,887,421	-€ 7,774,843	###########	###########
281	0.14	########	########	€ 668,818	€ 891,758	-€ 5,390,610	###########	###########	###########
282	0.15	########	########	€ 709,342	€ 945,789	-€ 5,717,226	###########	###########	###########
283	0.19	########	########	€ 887,867	#########	-€ 7,156,117	###########	###########	###########
284	0.12	########	########	€ 566,221	€ 754,961	-€ 4,563,682	-€ 9,127,364	###########	###########
285	0.18	########	########	€ 851,116	#########	-€ 6,859,907	###########	###########	###########
286	0.17	########	########	€ 785,887	#########	-€ 6,334,169	###########	###########	###########
287	0.11	########	########	€ 528,642	€ 704,856	-€ 4,260,804	-€ 8,521,607	###########	###########
288	0.16	########	########	€ 752,713	#########	-€ 6,066,792	###########	###########	###########
289	0.17	########	########	€ 794,792	#########	-€ 6,405,941	###########	###########	###########
290	0.19	########	########	€ 876,599	#########	-€ 7,065,301	###########	###########	###########
291	0.16	########	########	€ 777,327	#########	-€ 6,265,176	###########	###########	###########
292	0.15	########	########	€ 694,827	€ 926,436	-€ 5,600,235	###########	###########	###########
293	0.23	########	########	#########	#########	-€ 8,615,340	###########	###########	###########
294	0.19	########	########	€ 909,363	#########	-€ 7,329,376	###########	###########	###########
295	0.24	########	########	#########	#########	-€ 9,010,493	###########	###########	###########
296	0.15	########	########	€ 718,511	€ 958,015	-€ 5,791,127	###########	###########	###########
297	0.09	########	########	€ 410,734	€ 547,645	-€ 3,310,475	-€ 6,620,951	-€ 9,931,426	###########
298	0.14	########	########	€ 684,710	€ 912,946	-€ 5,518,693	###########	###########	###########
299	0.20	########	########	€ 966,742	#########	-€ 7,791,844	###########	###########	###########
300	0.17	########	########	€ 799,575	#########	-€ 6,444,493	###########	###########	###########
301	0.21	########	########	€ 978,689	#########	-€ 7,888,139	###########	###########	###########
302	0.13	########	########	€ 635,138	€ 846,851	-€ 5,119,153	###########	###########	###########
303	0.20	########	########	€ 957,593	#########	-€ 7,718,106	###########	###########	###########
304	0.17	########	########	€ 820,893	#########	-€ 6,616,320	###########	###########	###########
305	0.23	########	########	#########	#########	-€ 8,774,104	###########	###########	###########
306	0.16	########	########	€ 753,003	#########	-€ 6,069,131	###########	###########	###########
307	0.11	########	########	€ 505,962	€ 674,616	-€ 4,078,003	-€ 8,156,005	###########	###########
308	0.17	########	########	€ 813,858	#########	-€ 6,559,613	###########	###########	###########
309	0.13	########	########	€ 607,656	€ 810,207	-€ 4,897,644	-€ 9,795,287	###########	###########
310	0.13	########	########	€ 599,910	€ 799,879	-€ 4,835,212	-€ 9,670,423	###########	###########
311	0.11	########	########	€ 504,003	€ 672,004	-€ 4,062,215	-€ 8,124,431	###########	###########
312	0.18	########	########	€ 838,534	#########	-€ 6,758,504	###########	###########	###########
313	0.13	########	########	€ 600,617	€ 800,823	-€ 4,840,914	-€ 9,681,828	###########	###########
314	0.16	########	########	€ 734,460	€ 979,280	-€ 5,919,677	###########	###########	###########
315	0.15	########	########	€ 695,658	€ 927,543	-€ 5,606,931	###########	###########	###########
316	0.10	########	########	€ 484,522	€ 646,029	-€ 3,905,198	-€ 7,810,395	###########	###########
317	0.19	########	########	€ 910,392	#########	-€ 7,337,672	###########	###########	###########
318	0.19	########	########	€ 880,940	#########	-€ 7,100,286	###########	###########	###########
319	0.21	########	########	€ 986,489	#########	-€ 7,951,007	###########	###########	###########
320	0.20	########	########	€ 935,678	#########	-€ 7,541,475	###########	###########	###########
321	0.11	########	########	€ 541,750	€ 722,334	-€ 4,366,453	-€ 8,732,906	###########	###########
322	0.18	########	########	€ 837,325	#########	-€ 6,748,754	###########	###########	###########
323	0.10	########	########	€ 487,607	€ 650,142	-€ 3,930,061	-€ 7,860,122	###########	###########
324	0.20	########	########	€ 961,557	#########	-€ 7,750,057	###########	###########	###########
325	0.25	########	########	#########	#########	-€ 9,669,037	###########	###########	###########
326	0.23	########	########	#########	#########	-€ 8,838,367	###########	###########	###########
327	0.17	########	########	€ 796,308	#########	-€ 6,418,161	###########	###########	###########
328	0.18	########	########	€ 837,183	#########	-€ 6,747,616	###########	###########	###########
329	0.21	########	########	#########	#########	-€ 8,080,673	###########	###########	###########
330	0.15	########	########	€ 715,732	€ 954,310	-€ 5,768,730	###########	###########	###########
331	0.15	########	########	€ 708,272	€ 944,362	-€ 5,708,601	###########	###########	###########
332	0.13	########	########	€ 623,629	€ 831,505	-€ 5,026,388	###########	###########	###########
333	0.18	########	########	€ 853,490	#########	-€ 6,879,047	###########	###########	###########
334	0.20	########	########	€ 936,824	#########	-€ 7,550,713	###########	###########	###########
335	0.16	########	########	€ 775,424	#########	-€ 6,249,841	###########	###########	###########
336	0.14	########	########	€ 660,870	€ 881,160	-€ 5,326,548	###########	###########	###########
337	0.13	########	########	€ 591,657	€ 788,876	-€ 4,768,699	-€ 9,537,397	###########	###########
338	0.16	########	########	€ 754,593	#########	-€ 6,081,944	###########	###########	###########
339	0.13	########	########	€ 606,725	€ 808,966	-€ 4,890,141	-€ 9,780,282	###########	###########
340	0.12	########	########	€ 578,252	€ 771,002	-€ 4,660,651	-€ 9,321,302	###########	###########

341	0.18	########	########	€ 841,749	#########	-€ 6,784,410	###########	###########	###########
342	0.16	########	########	€ 753,760	#########	-€ 6,075,229	###########	###########	###########
343	0.13	########	########	€ 633,734	€ 844,979	-€ 5,107,836	###########	###########	###########
344	0.13	########	########	€ 616,987	€ 822,650	-€ 4,972,857	-€ 9,945,713	###########	###########
345	0.16	########	########	€ 764,842	#########	-€ 6,164,554	###########	###########	###########
346	0.12	########	########	€ 584,812	€ 779,749	-€ 4,713,523	-€ 9,427,047	###########	###########
347	0.17	########	########	€ 799,836	#########	-€ 6,446,603	###########	###########	###########
348	0.12	########	########	€ 551,467	€ 735,290	-€ 4,444,771	-€ 8,889,542	###########	###########
349	0.25	########	########	#########	#########	-€ 9,370,815	###########	###########	###########
350	0.17	########	########	€ 791,320	#########	-€ 6,377,961	###########	###########	###########
351	0.16	########	########	€ 759,247	#########	-€ 6,119,459	###########	###########	###########
352	0.22	########	########	#########	#########	-€ 8,244,145	###########	###########	###########
353	0.18	########	########	€ 830,763	#########	-€ 6,695,867	###########	###########	###########
354	0.21	########	########	€ 994,147	#########	-€ 8,012,728	###########	###########	###########
355	0.19	########	########	€ 873,888	#########	-€ 7,043,449	###########	###########	###########
356	0.14	########	########	€ 647,796	€ 863,728	-€ 5,221,170	###########	###########	###########
357	0.19	########	########	€ 916,482	#########	-€ 7,386,751	###########	###########	###########
358	0.14	########	########	€ 648,005	€ 864,007	-€ 5,222,857	###########	###########	###########
359	0.18	########	########	€ 871,920	#########	-€ 7,027,590	###########	###########	###########
360	0.10	########	########	€ 460,738	€ 614,318	-€ 3,713,506	-€ 7,427,013	###########	###########
361	0.18	########	########	€ 832,098	#########	-€ 6,706,630	###########	###########	###########
362	0.14	########	########	€ 647,118	€ 862,824	-€ 5,215,706	###########	###########	###########
363	0.17	########	########	€ 791,230	#########	-€ 6,377,238	###########	###########	###########
364	0.19	########	########	€ 914,883	#########	-€ 7,373,869	###########	###########	###########
365	0.16	########	########	€ 742,627	€ 990,169	-€ 5,985,501	###########	###########	###########
366	0.15	########	########	€ 718,378	€ 957,838	-€ 5,790,058	###########	###########	###########
367	0.16	########	########	€ 762,696	#########	-€ 6,147,255	###########	###########	###########
368	0.22	########	########	#########	#########	-€ 8,251,251	###########	###########	###########
369	0.19	########	########	€ 880,874	#########	-€ 7,099,756	###########	###########	###########
370	0.17	########	########	€ 810,648	#########	-€ 6,533,743	###########	###########	###########
371	0.14	########	########	€ 666,384	€ 888,512	-€ 5,370,989	###########	###########	###########
372	0.20	########	########	€ 950,850	#########	-€ 7,663,761	###########	###########	###########
373	0.15	########	########	€ 722,588	€ 963,451	-€ 5,823,988	###########	###########	###########
374	0.18	########	########	€ 844,475	#########	-€ 6,806,384	###########	###########	###########
375	0.19	########	########	€ 917,145	#########	-€ 7,392,101	###########	###########	###########
376	0.11	########	########	€ 543,189	€ 724,253	-€ 4,378,053	-€ 8,756,107	###########	###########
377	0.18	########	########	€ 836,653	#########	-€ 6,743,340	###########	###########	###########
378	0.14	########	########	€ 684,323	€ 912,431	-€ 5,515,578	###########	###########	###########
379	0.17	########	########	€ 826,181	#########	-€ 6,658,938	###########	###########	###########
380	0.18	########	########	€ 862,172	#########	-€ 6,949,018	###########	###########	###########
381	0.10	########	########	€ 452,565	€ 603,420	-€ 3,647,631	-€ 7,295,262	###########	###########
382	0.17	########	########	€ 784,941	#########	-€ 6,326,543	###########	###########	###########
383	0.18	########	########	€ 845,290	#########	-€ 6,812,956	###########	###########	###########
384	0.24	########	########	#########	#########	-€ 9,274,541	###########	###########	###########
385	0.09	########	########	€ 446,966	€ 595,955	-€ 3,602,506	-€ 7,205,012	###########	###########
386	0.14	########	########	€ 672,591	€ 896,789	-€ 5,421,021	###########	###########	###########
387	0.14	########	########	€ 666,466	€ 888,622	-€ 5,371,652	###########	###########	###########
388	0.17	########	########	€ 794,185	#########	-€ 6,401,053	###########	###########	###########
389	0.12	########	########	€ 543,917	€ 725,222	-€ 4,383,913	-€ 8,767,827	###########	###########
390	0.12	########	########	€ 564,005	€ 752,007	-€ 4,545,826	-€ 9,091,653	###########	###########
391	0.15	########	########	€ 731,588	€ 975,451	-€ 5,896,527	###########	###########	###########
392	0.14	########	########	€ 652,044	€ 869,393	-€ 5,255,414	###########	###########	###########
393	0.17	########	########	€ 821,370	#########	-€ 6,620,160	###########	###########	###########
394	0.13	########	########	€ 612,638	€ 816,851	-€ 4,937,802	-€ 9,875,604	###########	###########
395	0.21	########	########	#########	#########	-€ 8,146,087	###########	###########	###########
396	0.13	########	########	€ 630,700	€ 840,933	-€ 5,083,377	###########	###########	###########
397	0.19	########	########	€ 874,077	#########	-€ 7,044,972	###########	###########	###########
398	0.19	########	########	€ 919,297	#########	-€ 7,409,445	###########	###########	###########
399	0.13	########	########	€ 599,151	€ 798,868	-€ 4,829,101	-€ 9,658,201	###########	###########
400	0.17	########	########	€ 797,606	#########	-€ 6,428,622	###########	###########	###########
401	0.18	########	########	€ 844,392	#########	-€ 6,805,716	###########	###########	###########
402	0.13	########	########	€ 603,171	€ 804,227	-€ 4,861,495	-€ 9,722,990	###########	###########
403	0.21	########	########	€ 987,996	#########	-€ 7,963,151	###########	###########	###########
404	0.22	########	########	#########	#########	-€ 8,193,978	###########	###########	###########
405	0.23	########	########	#########	#########	-€ 8,583,870	###########	###########	###########
406	0.17	########	########	€ 787,778	#########	-€ 6,349,416	###########	###########	###########
407	0.11	########	########	€ 516,139	€ 688,185	-€ 4,160,030	-€ 8,320,060	###########	###########
408	0.15	########	########	€ 730,006	€ 973,341	-€ 5,883,774	###########	###########	###########
409	0.17	########	########	€ 793,883	#########	-€ 6,398,620	###########	###########	###########
410	0.17	########	########	€ 817,913	#########	-€ 6,592,299	###########	###########	###########
411	0.17	########	########	€ 791,848	#########	-€ 6,382,216	###########	###########	###########
412	0.11	########	########	€ 502,868	€ 670,491	-€ 4,053,069	-€ 8,106,139	###########	###########
413	0.12	########	########	€ 552,446	€ 736,595	-€ 4,452,664	-€ 8,905,328	###########	###########
414	0.12	########	########	€ 588,826	€ 785,102	-€ 4,745,881	-€ 9,491,762	###########	###########
415	0.16	########	########	€ 745,558	€ 994,078	-€ 6,009,125	###########	###########	###########
416	0.19	########	########	€ 882,222	#########	-€ 7,110,619	###########	###########	###########
417	0.21	########	########	#########	#########	-€ 8,117,906	###########	###########	###########
418	0.12	########	########	€ 575,731	€ 767,642	-€ 4,640,339	-€ 9,280,677	###########	###########
419	0.14	########	########	€ 639,294	€ 852,393	-€ 5,152,650	###########	###########	###########
420	0.24	########	########	#########	#########	-€ 9,125,273	###########	###########	###########
421	0.18	########	########	€ 860,313	#########	-€ 6,934,037	###########	###########	###########
422	0.18	########	########	€ 848,704	#########	-€ 6,840,474	###########	###########	###########
423	0.14	########	########	€ 679,794	€ 906,392	-€ 5,479,072	###########	###########	###########
424	0.18	########	########	€ 860,491	#########	-€ 6,935,471	###########	###########	###########
425	0.16	########	########	€ 776,442	#########	-€ 6,258,045	###########	###########	###########
426	0.17	########	########	€ 791,687	#########	-€ 6,380,919	###########	###########	###########
427	0.20	########	########	€ 950,444	#########	-€ 7,660,486	###########	###########	###########
428	0.11	########	########	€ 509,296	€ 679,062	-€ 4,104,878	-€ 8,209,757	###########	###########
429	0.12	########	########	€ 569,601	€ 759,467	-€ 4,590,924	-€ 9,181,847	###########	###########
430	0.18	########	########	€ 830,232	#########	-€ 6,691,590	###########	###########	###########
431	0.18	########	########	€ 848,760	#########	-€ 6,840,918	###########	###########	###########
432	0.09	########	########	€ 439,960	€ 586,614	-€ 3,546,036	-€ 7,092,072	###########	###########
433	0.15	########	########	€ 699,684	€ 932,912	-€ 5,639,384	###########	###########	###########
434	0.12	########	########	€ 554,284	€ 739,045	-€ 4,467,471	-€ 8,934,942	###########	###########
435	0.15	########	########	€ 720,281	€ 960,374	-€ 5,805,391	###########	###########	###########
436	0.13	########	########	€ 600,132	€ 800,177	-€ 4,837,008	-€ 9,674,015	###########	###########
437	0.14	########	########	€ 657,603	€ 876,804	-€ 5,300,217	###########	###########	###########
438	0.23	########	########	#########	#########	-€ 8,828,768	###########	###########	###########
439	0.22	########	########	#########	#########	-€ 8,503,619	###########	###########	###########
440	0.09	########	########	€ 402,567	€ 536,756	-€ 3,244,651	-€ 6,489,303	-€ 9,733,954	###########
441	0.15	########	########	€ 701,524	€ 935,366	-€ 5,654,217	###########	###########	###########
442	0.16	########	########	€ 777,210	#########	-€ 6,264,237	###########	###########	###########
443	0.11	########	########	€ 511,012	€ 681,349	-€ 4,118,707	-€ 8,237,413	###########	###########
444	0.17	########	########	€ 787,519	#########	-€ 6,347,324	###########	###########	###########
445	0.09	########	########	€ 418,681	€ 558,241	-€ 3,374,526	-€ 6,749,053	###########	###########
446	0.16	########	########	€ 751,997	#########	-€ 6,061,023	###########	###########	###########
447	0.17	########	########	€ 809,415	#########	-€ 6,523,809	###########	###########	###########
448	0.19	########	########	€ 917,755	#########	-€ 7,397,014	###########	###########	###########
449	0.19	########	########	€ 914,154	#########	-€ 7,367,987	###########	###########	###########
450	0.22	########	########	#########	#########	-€ 8,433,045	###########	###########	###########
451	0.18	########	########	€ 854,017	#########	-€ 6,883,289	###########	###########	###########
452	0.13	########	########	€ 625,059	€ 833,413	-€ 5,037,917	###########	###########	###########
453	0.15	########	########	€ 717,368	€ 956,490	-€ 5,781,912	###########	###########	###########
454	0.18	########	########	€ 848,949	#########	-€ 6,842,443	###########	###########	###########
455	0.21	########	########	€ 973,099	#########	-€ 7,843,085	###########	###########	###########
456	0.15	########	########	€ 726,143	€ 968,191	-€ 5,852,640	###########	###########	###########
457	0.20	########	########	€ 948,334	#########	-€ 7,643,475	###########	###########	###########
458	0.10	########	########	€ 455,786	€ 607,715	-€ 3,673,594	-€ 7,347,188	###########	###########
459	0.18	########	########	€ 846,105	#########	-€ 6,819,523	###########	###########	###########
460	0.12	########	########	€ 553,802	€ 738,403	-€ 4,463,591	-€ 8,927,182	###########	###########
461	0.17	########	########	€ 816,067	#########	-€ 6,577,422	###########	###########	###########
462	0.13	########	########	€ 607,305	€ 809,740	-€ 4,894,820	-€ 9,789,639	###########	###########
463	0.16	########	########	€ 742,054	€ 989,405	-€ 5,980,882	###########	###########	###########
464	0.20	########	########	€ 943,866	#########	-€ 7,607,468	###########	###########	###########
465	0.14	########	########	€ 663,118	€ 884,157	-€ 5,344,661	###########	###########	###########
466	0.17	########	########	€ 790,380	#########	-€ 6,370,387	###########	###########	###########
467	0.13	########	########	€ 606,568	€ 808,758	-€ 4,888,879	-€ 9,777,759	###########	###########
468	0.13	########	########	€ 602,871	€ 803,828	-€ 4,859,081	-€ 9,718,162	###########	###########
469	0.14	########	########	€ 639,844	€ 853,126	-€ 5,157,083	###########	###########	###########
470	0.11	########	########	€ 503,891	€ 671,855	-€ 4,061,313	-€ 8,122,626	###########	###########
471	0.11	########	########	€ 520,006	€ 693,341	-€ 4,191,195	-€ 8,382,390	###########	###########
472	0.13	########	########	€ 627,508	€ 836,677	-€ 5,057,650	###########	###########	###########
473	0.15	########	########	€ 696,332	€ 928,443	-€ 5,612,370	###########	###########	###########
474	0.16	########	########	€ 774,217	#########	-€ 6,240,113	###########	###########	###########
475	0.21	########	########	#########	#########	-€ 8,063,504	###########	###########	###########
476	0.20	########	########	€ 956,806	#########	-€ 7,711,761	###########	###########	###########
477	0.10	########	########	€ 484,059	€ 645,412	-€ 3,901,469	-€ 7,802,939	###########	###########
478	0.15	########	########	€ 703,294	€ 937,725	-€ 5,668,478	###########	###########	###########
479	0.12	########	########	€ 574,183	€ 765,577	-€ 4,627,856	-€ 9,255,712	###########	###########
480	0.13	########	########	€ 598,362	€ 797,817	-€ 4,822,742	-€ 9,645,484	###########	###########
481	0.12	########	########	€ 543,538	€ 724,718	-€ 4,380,866	-€ 8,761,733	###########	###########
482	0.18	########	########	€ 858,559	#########	-€ 6,919,897	###########	###########	###########
483	0.11	########	########	€ 537,552	€ 716,735	-€ 4,332,612	-€ 8,665,225	###########	###########
484	0.12	########	########	€ 553,079	€ 737,438	-€ 4,457,759	-€ 8,915,518	###########	###########
485	0.21	########	########	#########	#########	-€ 8,112,636	###########	###########	###########
486	0.25	########	########	#########	#########	-€ 9,457,433	###########	###########	###########
487	0.21	########	########	€ 989,863	#########	-€ 7,978,200	###########	###########	###########
488	0.18	########	########	€ 870,161	#########	-€ 7,013,412	###########	###########	###########
489	0.14	########	########	€ 683,232	€ 910,977	-€ 5,506,786	###########	###########	###########
490	0.16	########	########	€ 776,310	#########	-€ 6,256,979	###########	###########	###########
491	0.13	########	########	€ 623,995	€ 831,993	-€ 5,029,335	###########	###########	###########
492	0.16	########	########	€ 738,749	€ 984,999	-€ 5,954,244	###########	###########	###########
493	0.15	########	########	€ 707,942	€ 943,923	-€ 5,705,943	###########	###########	###########
494	0.15	########	########	€ 693,003	€ 924,004	-€ 5,585,536	###########	###########	###########
495	0.13	########	########	€ 617,885	€ 823,846	-€ 4,980,091	-€ 9,960,181	###########	###########
496	0.16	########	########	€ 751,945	#########	-€ 6,060,600	###########	###########	###########
497	0.14	########	########	€ 665,457	€ 887,276	-€ 5,363,520	###########	###########	###########
498	0.15	########	########	€ 724,375	€ 965,833	-€ 5,838,388	###########	###########	###########
499	0.13	########	########	€ 622,059	€ 829,412	-€ 5,013,734	###########	###########	###########
500	0.16	########	########	€ 772,170	#########	-€ 6,223,612	###########	###########	###########
501	0.13	########	########	€ 629,092	€ 838,790	-€ 5,070,420	###########	###########	###########
502	0.11	########	########	€ 511,799	€ 682,399	-€ 4,125,052	-€ 8,250,104	###########	###########
503	0.10	########	########	€ 454,634	€ 606,179	-€ 3,664,306	-€ 7,328,612	###########	###########
504	0.20	########	########	€ 929,750	#########	-€ 7,493,692	###########	###########	###########
505	0.11	########	########	€ 499,083	€ 665,444	-€ 4,022,561	-€ 8,045,122	###########	###########
506	0.17	########	########	€ 805,297	#########	-€ 6,490,610	###########	###########	###########
507	0.13	########	########	€ 636,933	€ 849,244	-€ 5,133,615	###########	###########	###########
508	0.15	########	########	€ 720,096	€ 960,128	-€ 5,803,903	###########	###########	###########
509	0.16	########	########	€ 734,514	€ 979,352	-€ 5,920,113	###########	###########	###########
510	0.11	########	########	€ 537,868	€ 717,157	-€ 4,335,163	-€ 8,670,325	###########	###########
511	0.19	########	########	€ 910,345	#########	-€ 7,337,289	###########	###########	###########
512	0.20	########	########	€ 961,550	#########	-€ 7,749,994	###########	###########	###########

513	0.19	########	########	€ 897,889	#########	-€ 7,236,896	###########	###########	###########
514	0.15	########	########	€ 723,527	€ 964,703	-€ 5,831,557	###########	###########	###########
515	0.17	########	########	€ 794,804	#########	-€ 6,406,041	###########	###########	###########
516	0.15	########	########	€ 701,717	€ 935,623	-€ 5,655,770	###########	###########	###########
517	0.17	########	########	€ 794,804	#########	-€ 6,406,042	###########	###########	###########
518	0.17	########	########	€ 785,661	#########	-€ 6,332,353	###########	###########	###########
519	0.10	########	########	€ 470,328	€ 627,104	-€ 3,790,799	-€ 7,581,599	###########	###########
520	0.20	########	########	€ 935,264	#########	-€ 7,538,132	###########	###########	###########
521	0.18	########	########	€ 861,380	#########	-€ 6,942,642	###########	###########	###########
522	0.15	########	########	€ 703,803	€ 938,405	-€ 5,672,586	###########	###########	###########
523	0.14	########	########	€ 669,972	€ 893,296	-€ 5,399,910	###########	###########	###########
524	0.17	########	########	€ 796,483	#########	-€ 6,419,577	###########	###########	###########
525	0.21	########	########	€ 983,932	#########	-€ 7,930,394	###########	###########	###########
526	0.11	########	########	€ 507,846	€ 677,128	-€ 4,093,189	-€ 8,186,378	###########	###########
527	0.16	########	########	€ 763,717	#########	-€ 6,155,484	###########	###########	###########
528	0.16	########	########	€ 751,583	#########	-€ 6,057,682	###########	###########	###########
529	0.17	########	########	€ 790,223	#########	-€ 6,369,117	###########	###########	###########
530	0.16	########	########	€ 732,540	€ 976,720	-€ 5,904,200	###########	###########	###########
531	0.24	########	########	#########	#########	-€ 8,981,862	###########	###########	###########
532	0.19	########	########	€ 906,158	#########	-€ 7,303,545	###########	###########	###########
533	0.12	########	########	€ 586,069	€ 781,426	-€ 4,723,662	-€ 9,447,324	###########	###########
534	0.12	########	########	€ 575,736	€ 767,649	-€ 4,640,379	-€ 9,280,758	###########	###########
535	0.15	########	########	€ 722,903	€ 963,871	-€ 5,826,528	###########	###########	###########
536	0.22	########	########	#########	#########	-€ 8,566,090	###########	###########	###########
537	0.19	########	########	€ 888,916	#########	-€ 7,164,575	###########	###########	###########
538	0.11	########	########	€ 505,377	€ 673,836	-€ 4,073,287	-€ 8,146,574	###########	###########
539	0.16	########	########	€ 746,195	€ 994,927	-€ 6,014,261	###########	###########	###########
540	0.18	########	########	€ 852,057	#########	-€ 6,867,499	###########	###########	###########
541	0.14	########	########	€ 669,488	€ 892,651	-€ 5,396,007	###########	###########	###########
542	0.12	########	########	€ 566,780	€ 755,706	-€ 4,568,189	-€ 9,136,377	###########	###########
543	0.08	########	########	€ 390,491	€ 520,655	-€ 3,147,318	-€ 6,294,636	-€ 9,441,954	###########
544	0.15	########	########	€ 692,229	€ 922,973	-€ 5,579,301	###########	###########	###########
545	0.17	########	########	€ 801,451	#########	-€ 6,459,613	###########	###########	###########
546	0.20	########	########	€ 963,755	#########	-€ 7,767,767	###########	###########	###########
547	0.14	########	########	€ 642,939	€ 857,252	-€ 5,182,022	###########	###########	###########
548	0.17	########	########	€ 806,350	#########	-€ 6,499,102	###########	###########	###########
549	0.16	########	########	€ 772,713	#########	-€ 6,227,994	###########	###########	###########
550	0.15	########	########	€ 712,931	€ 950,575	-€ 5,746,156	###########	###########	###########
551	0.20	########	########	€ 947,503	#########	-€ 7,636,779	###########	###########	###########
552	0.20	########	########	€ 961,240	#########	-€ 7,747,500	###########	###########	###########
553	0.18	########	########	€ 871,531	#########	-€ 7,024,454	###########	###########	###########
554	0.12	########	########	€ 577,743	€ 770,323	-€ 4,656,547	-€ 9,313,095	###########	###########
555	0.13	########	########	€ 633,801	€ 845,069	-€ 5,108,377	###########	###########	###########
556	0.24	########	########	#########	#########	-€ 9,305,593	###########	###########	###########
557	0.11	########	########	€ 521,228	€ 694,970	-€ 4,201,044	-€ 8,402,089	###########	###########
558	0.12	########	########	€ 563,476	€ 751,301	-€ 4,541,559	-€ 9,083,118	###########	###########
559	0.18	########	########	€ 839,824	#########	-€ 6,768,899	###########	###########	###########
560	0.16	########	########	€ 774,649	#########	-€ 6,243,596	###########	###########	###########
561	0.18	########	########	€ 844,210	#########	-€ 6,804,251	###########	###########	###########
562	0.18	########	########	€ 831,604	#########	-€ 6,702,648	###########	###########	###########
563	0.22	########	########	#########	#########	-€ 8,204,265	###########	###########	###########
564	0.10	########	########	€ 480,838	€ 641,118	-€ 3,875,509	-€ 7,751,018	###########	###########
565	0.15	########	########	€ 725,291	€ 967,055	-€ 5,845,773	###########	###########	###########
566	0.21	########	########	€ 970,455	#########	-€ 7,821,770	###########	###########	###########
567	0.20	########	########	€ 940,164	#########	-€ 7,577,628	###########	###########	###########
568	0.13	########	########	€ 630,093	€ 840,124	-€ 5,078,489	###########	###########	###########
569	0.09	########	########	€ 424,380	€ 565,840	-€ 3,420,461	-€ 6,840,922	###########	###########
570	0.15	########	########	€ 725,126	€ 966,834	-€ 5,844,441	###########	###########	###########
571	0.11	########	########	€ 532,952	€ 710,603	-€ 4,295,540	-€ 8,591,080	###########	###########
572	0.17	########	########	€ 805,553	#########	-€ 6,492,679	###########	###########	###########
573	0.13	########	########	€ 609,363	€ 812,484	-€ 4,911,404	-€ 9,822,808	###########	###########
574	0.09	########	########	€ 425,472	€ 567,296	-€ 3,429,263	-€ 6,858,525	###########	###########
575	0.14	########	########	€ 679,615	€ 906,153	-€ 5,477,629	###########	###########	###########
576	0.16	########	########	€ 735,062	€ 980,083	-€ 5,924,529	###########	###########	###########
577	0.18	########	########	€ 860,101	#########	-€ 6,932,329	###########	###########	###########
578	0.19	########	########	€ 887,599	#########	-€ 7,153,960	###########	###########	###########
579	0.17	########	########	€ 812,364	#########	-€ 6,547,572	###########	###########	###########
580	0.18	########	########	€ 868,978	#########	-€ 7,003,873	###########	###########	###########
581	0.15	########	########	€ 690,725	€ 920,966	-€ 5,567,173	###########	###########	###########
582	0.15	########	########	€ 712,090	€ 949,454	-€ 5,739,378	###########	###########	###########
583	0.14	########	########	€ 671,603	€ 895,470	-€ 5,413,051	###########	###########	###########
584	0.17	########	########	€ 816,336	#########	-€ 6,579,584	###########	###########	###########
585	0.13	########	########	€ 599,861	€ 799,814	-€ 4,834,817	-€ 9,669,634	###########	###########
586	0.13	########	########	€ 614,117	€ 818,823	-€ 4,949,721	-€ 9,899,443	###########	###########
587	0.16	########	########	€ 745,837	€ 994,450	-€ 6,011,374	###########	###########	###########
588	0.10	########	########	€ 476,592	€ 635,457	-€ 3,841,288	-€ 7,682,577	###########	###########
589	0.18	########	########	€ 838,321	#########	-€ 6,756,788	###########	###########	###########
590	0.12	########	########	€ 590,396	€ 787,195	-€ 4,758,535	-€ 9,517,069	###########	###########
591	0.16	########	########	€ 770,988	#########	-€ 6,214,090	###########	###########	###########
592	0.16	########	########	€ 739,933	€ 986,577	-€ 5,963,785	###########	###########	###########
593	0.23	########	########	#########	#########	-€ 8,746,810	###########	###########	###########
594	0.17	########	########	€ 809,113	#########	-€ 6,521,373	###########	###########	###########
595	0.16	########	########	€ 732,495	€ 976,660	-€ 5,903,840	###########	###########	###########
596	0.19	########	########	€ 910,020	#########	-€ 7,334,670	###########	###########	###########
597	0.12	########	########	€ 564,257	€ 752,343	-€ 4,547,858	-€ 9,095,715	###########	###########
598	0.08	########	########	€ 388,920	€ 518,561	-€ 3,134,661	-€ 6,269,321	-€ 9,403,982	###########
599	0.19	########	########	€ 899,289	#########	-€ 7,248,177	###########	###########	###########
600	0.18	########	########	€ 834,826	#########	-€ 6,728,619	###########	###########	###########
601	0.19	########	########	€ 901,697	#########	-€ 7,267,589	###########	###########	###########
602	0.11	########	########	€ 541,064	€ 721,418	-€ 4,360,919	-€ 8,721,838	###########	###########
603	0.22	########	########	#########	#########	-€ 8,205,224	###########	###########	###########
604	0.15	########	########	€ 687,044	€ 916,059	-€ 5,537,508	###########	###########	###########
605	0.18	########	########	€ 862,776	#########	-€ 6,953,891	###########	###########	###########
606	0.16	########	########	€ 771,594	#########	-€ 6,218,970	###########	###########	###########
607	0.14	########	########	€ 646,141	€ 861,522	-€ 5,207,834	###########	###########	###########
608	0.15	########	########	€ 703,180	€ 937,573	-€ 5,667,560	###########	###########	###########
609	0.24	########	########	#########	#########	-€ 9,018,153	###########	###########	###########
610	0.18	########	########	€ 862,293	#########	-€ 6,949,998	###########	###########	###########
611	0.10	########	########	€ 454,644	€ 606,192	-€ 3,664,386	-€ 7,328,771	###########	###########
612	0.13	########	########	€ 633,635	€ 844,846	-€ 5,107,032	###########	###########	###########
613	0.19	########	########	€ 897,806	#########	-€ 7,236,227	###########	###########	###########
614	0.14	########	########	€ 641,311	€ 855,081	-€ 5,168,902	###########	###########	###########
615	0.16	########	########	€ 750,763	#########	-€ 6,051,075	###########	###########	###########
616	0.13	########	########	€ 612,099	€ 816,133	-€ 4,933,460	-€ 9,866,921	###########	###########
617	0.23	########	########	#########	#########	-€ 8,679,410	###########	###########	###########
618	0.18	########	########	€ 869,880	#########	-€ 7,011,147	###########	###########	###########
619	0.17	########	########	€ 794,029	#########	-€ 6,399,794	###########	###########	###########
620	0.16	########	########	€ 742,646	€ 990,195	-€ 5,985,656	###########	###########	###########
621	0.14	########	########	€ 650,887	€ 867,849	-€ 5,246,085	###########	###########	###########
622	0.14	########	########	€ 669,135	€ 892,180	-€ 5,393,163	###########	###########	###########
623	0.16	########	########	€ 763,658	#########	-€ 6,155,012	###########	###########	###########
624	0.15	########	########	€ 719,567	€ 959,422	-€ 5,799,636	###########	###########	###########
625	0.16	########	########	€ 751,466	#########	-€ 6,056,743	###########	###########	###########
626	0.21	########	########	€ 993,123	#########	-€ 8,004,473	###########	###########	###########
627	0.14	########	########	€ 646,871	€ 862,495	-€ 5,213,719	###########	###########	###########
628	0.14	########	########	€ 679,322	€ 905,762	-€ 5,475,266	###########	###########	###########
629	0.16	########	########	€ 745,472	€ 993,963	-€ 6,008,434	###########	###########	###########
630	0.13	########	########	€ 591,635	€ 788,846	-€ 4,768,517	-€ 9,537,034	###########	###########
631	0.19	########	########	€ 894,021	#########	-€ 7,205,724	###########	###########	###########
632	0.09	########	########	€ 444,477	€ 592,636	-€ 3,582,442	-€ 7,164,884	###########	###########
633	0.10	########	########	€ 482,374	€ 643,166	-€ 3,887,890	-€ 7,775,780	###########	###########
634	0.17	########	########	€ 810,399	#########	-€ 6,531,733	###########	###########	###########
635	0.15	########	########	€ 698,559	€ 931,411	-€ 5,630,313	###########	###########	###########
636	0.14	########	########	€ 679,877	€ 906,503	-€ 5,479,743	###########	###########	###########
637	0.12	########	########	€ 574,878	€ 766,504	-€ 4,633,460	-€ 9,266,920	###########	###########
638	0.20	########	########	€ 954,476	#########	-€ 7,692,981	###########	###########	###########
639	0.11	########	########	€ 538,597	€ 718,129	-€ 4,341,039	-€ 8,682,079	###########	###########
640	0.15	########	########	€ 686,037	€ 914,716	-€ 5,529,388	###########	###########	###########
641	0.19	########	########	€ 898,266	#########	-€ 7,239,932	###########	###########	###########
642	0.24	########	########	#########	#########	-€ 9,038,316	###########	###########	###########
643	0.10	########	########	€ 494,950	€ 659,933	-€ 3,989,245	-€ 7,978,489	###########	###########
644	0.14	########	########	€ 663,820	€ 885,093	-€ 5,350,320	###########	###########	###########
645	0.18	########	########	€ 827,603	#########	-€ 6,670,397	###########	###########	###########
646	0.16	########	########	€ 744,816	€ 993,088	-€ 6,003,141	###########	###########	###########
647	0.15	########	########	€ 726,133	€ 968,177	-€ 5,852,561	###########	###########	###########
648	0.14	########	########	€ 667,602	€ 890,137	-€ 5,380,810	###########	###########	###########
649	0.12	########	########	€ 565,648	€ 754,197	-€ 4,559,065	-€ 9,118,130	###########	###########
650	0.20	########	########	€ 967,247	#########	-€ 7,795,916	###########	###########	###########
651	0.12	########	########	€ 572,249	€ 762,998	-€ 4,612,268	-€ 9,224,537	###########	###########
652	0.19	########	########	€ 888,293	#########	-€ 7,159,552	###########	###########	###########
653	0.20	########	########	€ 925,386	#########	-€ 7,458,519	###########	###########	###########
654	0.11	########	########	€ 535,529	€ 714,039	-€ 4,316,310	-€ 8,632,621	###########	###########
655	0.13	########	########	€ 633,173	€ 844,230	-€ 5,103,309	###########	###########	###########
656	0.18	########	########	€ 846,248	#########	-€ 6,820,675	###########	###########	###########
657	0.14	########	########	€ 678,090	€ 904,120	-€ 5,465,339	###########	###########	###########
658	0.19	########	########	€ 919,332	#########	-€ 7,409,727	###########	###########	###########
659	0.21	########	########	€ 987,355	#########	-€ 7,957,985	###########	###########	###########
660	0.17	########	########	€ 802,717	#########	-€ 6,469,821	###########	###########	###########
661	0.20	########	########	€ 930,387	#########	-€ 7,498,826	###########	###########	###########
662	0.21	########	########	€ 982,940	#########	-€ 7,922,396	###########	###########	###########
663	0.13	########	########	€ 625,161	€ 833,548	-€ 5,038,737	###########	###########	###########
664	0.22	########	########	#########	#########	-€ 8,535,643	###########	###########	###########
665	0.15	########	########	€ 696,972	€ 929,296	-€ 5,617,526	###########	###########	###########
666	0.19	########	########	€ 910,408	#########	-€ 7,337,797	###########	###########	###########
667	0.22	########	########	#########	#########	-€ 8,199,537	###########	###########	###########
668	0.16	########	########	€ 778,607	#########	-€ 6,275,495	###########	###########	###########
669	0.19	########	########	€ 918,622	#########	-€ 7,404,006	###########	###########	###########
670	0.15	########	########	€ 694,853	€ 926,470	-€ 5,600,444	###########	###########	###########
671	0.21	########	########	€ 977,796	#########	-€ 7,880,937	###########	###########	###########
672	0.20	########	########	€ 941,481	#########	-€ 7,588,242	###########	###########	###########
673	0.12	########	########	€ 556,316	€ 741,755	-€ 4,483,856	-€ 8,967,712	###########	###########
674	0.19	########	########	€ 908,151	#########	-€ 7,319,608	###########	###########	###########
675	0.17	########	########	€ 813,348	#########	-€ 6,555,506	###########	###########	###########
676	0.17	########	########	€ 790,385	#########	-€ 6,370,427	###########	###########	###########
677	0.19	########	########	€ 908,002	#########	-€ 7,318,408	###########	###########	###########
678	0.11	########	########	€ 543,109	€ 724,145	-€ 4,377,403	-€ 8,754,805	###########	###########
679	0.07	########	########	€ 332,771	€ 443,694	-€ 2,682,097	-€ 5,364,195	-€ 8,046,292	###########
680	0.19	########	########	€ 892,017	#########	-€ 7,189,565	###########	###########	###########
681	0.16	########	########	€ 747,228	€ 996,304	-€ 6,022,583	###########	###########	###########
682	0.21	########	########	#########	#########	-€ 8,145,529	###########	###########	###########
683	0.16	########	########	€ 776,774	#########	-€ 6,260,718	###########	###########	###########
684	0.14	########	########	€ 683,822	€ 911,763	-€ 5,511,539	###########	###########	###########

685	0.22	########	########	#########	#########	-€ 8,288,584	###########	###########	###########
686	0.20	########	########	€ 943,627	#########	-€ 7,605,540	###########	###########	###########
687	0.18	########	########	€ 869,437	#########	-€ 7,007,576	###########	###########	###########
688	0.10	########	########	€ 492,291	€ 656,388	-€ 3,967,815	-€ 7,935,631	###########	###########
689	0.17	########	########	€ 813,864	#########	-€ 6,559,661	###########	###########	###########
690	0.18	########	########	€ 838,010	#########	-€ 6,754,278	###########	###########	###########
691	0.17	########	########	€ 818,285	#########	-€ 6,595,299	###########	###########	###########
692	0.16	########	########	€ 750,976	#########	-€ 6,052,792	###########	###########	###########
693	0.12	########	########	€ 577,705	€ 770,274	-€ 4,656,247	-€ 9,312,495	###########	###########
694	0.14	########	########	€ 675,622	€ 900,829	-€ 5,445,447	###########	###########	###########
695	0.14	########	########	€ 666,038	€ 888,051	-€ 5,368,200	###########	###########	###########
696	0.09	########	########	€ 440,661	€ 587,548	-€ 3,551,682	-€ 7,103,363	###########	###########
697	0.17	########	########	€ 813,018	#########	-€ 6,552,844	###########	###########	###########
698	0.10	########	########	€ 481,284	€ 641,712	-€ 3,879,101	-€ 7,758,203	###########	###########
699	0.12	########	########	€ 580,801	€ 774,402	-€ 4,681,201	-€ 9,362,402	###########	###########
700	0.18	########	########	€ 829,499	#########	-€ 6,685,682	###########	###########	###########
701	0.21	########	########	#########	#########	-€ 8,070,788	###########	###########	###########
702	0.13	########	########	€ 617,176	€ 822,901	-€ 4,974,375	-€ 9,948,750	###########	###########
703	0.18	########	########	€ 834,984	#########	-€ 6,729,891	###########	###########	###########
704	0.18	########	########	€ 867,907	#########	-€ 6,995,247	###########	###########	###########
705	0.13	########	########	€ 613,746	€ 818,328	-€ 4,946,733	-€ 9,893,465	###########	###########
706	0.17	########	########	€ 803,569	#########	-€ 6,476,684	###########	###########	###########
707	0.12	########	########	€ 584,600	€ 779,466	-€ 4,711,815	-€ 9,423,629	###########	###########
708	0.17	########	########	€ 801,566	#########	-€ 6,460,543	###########	###########	###########
709	0.18	########	########	€ 847,250	#########	-€ 6,828,747	###########	###########	###########
710	0.11	########	########	€ 514,935	€ 686,580	-€ 4,150,324	-€ 8,300,648	###########	###########
711	0.09	########	########	€ 444,594	€ 592,791	-€ 3,583,380	-€ 7,166,760	###########	###########
712	0.15	########	########	€ 717,092	€ 956,123	-€ 5,779,690	###########	###########	###########
713	0.12	########	########	€ 549,001	€ 732,002	-€ 4,424,896	-€ 8,849,793	###########	###########
714	0.12	########	########	€ 588,983	€ 785,311	-€ 4,747,147	-€ 9,494,294	###########	###########
715	0.15	########	########	€ 714,911	€ 953,215	-€ 5,762,115	###########	###########	###########
716	0.12	########	########	€ 572,146	€ 762,861	-€ 4,611,439	-€ 9,222,878	###########	###########
717	0.13	########	########	€ 619,648	€ 826,197	-€ 4,994,300	-€ 9,988,600	###########	###########
718	0.15	########	########	€ 707,143	€ 942,858	-€ 5,699,506	###########	###########	###########
719	0.21	########	########	#########	#########	-€ 8,104,279	###########	###########	###########
720	0.16	########	########	€ 746,333	€ 995,110	-€ 6,015,367	###########	###########	###########
721	0.16	########	########	€ 740,273	€ 987,031	-€ 5,966,530	###########	###########	###########
722	0.19	########	########	€ 910,488	#########	-€ 7,338,440	###########	###########	###########
723	0.14	########	########	€ 654,305	€ 872,407	-€ 5,273,636	###########	###########	###########
724	0.16	########	########	€ 772,283	#########	-€ 6,224,525	###########	###########	###########
725	0.16	########	########	€ 771,760	#########	-€ 6,220,311	###########	###########	###########
726	0.18	########	########	€ 862,839	#########	-€ 6,954,394	###########	###########	###########
727	0.16	########	########	€ 761,902	#########	-€ 6,140,858	###########	###########	###########
728	0.20	########	########	€ 953,744	#########	-€ 7,687,083	###########	###########	###########
729	0.16	########	########	€ 759,262	#########	-€ 6,119,576	###########	###########	###########
730	0.17	########	########	€ 794,146	#########	-€ 6,400,740	###########	###########	###########
731	0.16	########	########	€ 766,528	#########	-€ 6,178,139	###########	###########	###########
732	0.13	########	########	€ 614,404	€ 819,205	-€ 4,952,034	-€ 9,904,068	###########	###########
733	0.14	########	########	€ 675,074	€ 900,099	-€ 5,441,032	###########	###########	###########
734	0.07	########	########	€ 321,761	€ 429,015	-€ 2,593,363	-€ 5,186,726	-€ 7,780,090	###########
735	0.15	########	########	€ 720,049	€ 960,065	-€ 5,803,520	###########	###########	###########
736	0.19	########	########	€ 897,253	#########	-€ 7,231,767	###########	###########	###########
737	0.16	########	########	€ 766,704	#########	-€ 6,179,559	###########	###########	###########
738	0.14	########	########	€ 661,686	€ 882,248	-€ 5,333,124	###########	###########	###########
739	0.13	########	########	€ 621,271	€ 828,362	-€ 5,007,385	###########	###########	###########
740	0.20	########	########	€ 954,564	#########	-€ 7,693,694	###########	###########	###########
741	0.17	########	########	€ 792,913	#########	-€ 6,390,800	###########	###########	###########
742	0.19	########	########	€ 887,726	#########	-€ 7,154,981	###########	###########	###########
743	0.14	########	########	€ 682,581	€ 910,108	-€ 5,501,536	###########	###########	###########
744	0.19	########	########	€ 899,188	#########	-€ 7,247,367	###########	###########	###########
745	0.20	########	########	€ 960,827	#########	-€ 7,744,167	###########	###########	###########
746	0.20	########	########	€ 932,866	#########	-€ 7,518,804	###########	###########	###########
747	0.15	########	########	€ 708,874	€ 945,166	-€ 5,713,456	###########	###########	###########
748	0.10	########	########	€ 463,347	€ 617,796	-€ 3,734,533	-€ 7,469,065	###########	###########
749	0.17	########	########	€ 824,842	#########	-€ 6,648,143	###########	###########	###########
750	0.13	########	########	€ 616,183	€ 821,577	-€ 4,966,372	-€ 9,932,744	###########	###########
751	0.17	########	########	€ 786,867	#########	-€ 6,342,071	###########	###########	###########
752	0.14	########	########	€ 669,219	€ 892,292	-€ 5,393,841	###########	###########	###########
753	0.11	########	########	€ 507,070	€ 676,093	-€ 4,086,930	-€ 8,173,861	###########	###########
754	0.14	########	########	€ 678,723	€ 904,964	-€ 5,470,442	###########	###########	###########
755	0.16	########	########	€ 767,383	#########	-€ 6,185,032	###########	###########	###########
756	0.15	########	########	€ 720,038	€ 960,051	-€ 5,803,436	###########	###########	###########
757	0.15	########	########	€ 722,624	€ 963,499	-€ 5,824,279	###########	###########	###########
758	0.12	########	########	€ 584,922	€ 779,896	-€ 4,714,412	-€ 9,428,825	###########	###########
759	0.09	########	########	€ 412,515	€ 550,020	-€ 3,324,828	-€ 6,649,656	-€ 9,974,484	###########
760	0.19	########	########	€ 900,058	#########	-€ 7,254,382	###########	###########	###########
761	0.17	########	########	€ 816,536	#########	-€ 6,581,201	###########	###########	###########
762	0.18	########	########	€ 865,612	#########	-€ 6,976,747	###########	###########	###########
763	0.15	########	########	€ 722,538	€ 963,384	-€ 5,823,586	###########	###########	###########
764	0.19	########	########	€ 917,300	#########	-€ 7,393,346	###########	###########	###########
765	0.11	########	########	€ 509,536	€ 679,382	-€ 4,106,812	-€ 8,213,625	###########	###########
766	0.12	########	########	€ 582,392	€ 776,523	-€ 4,694,026	-€ 9,388,051	###########	###########
767	0.27	########	########	#########	#########	###########	###########	###########	###########
768	0.12	########	########	€ 564,656	€ 752,874	-€ 4,551,069	-€ 9,102,139	###########	###########
769	0.21	########	########	#########	#########	-€ 8,082,594	###########	###########	###########
770	0.17	########	########	€ 817,969	#########	-€ 6,592,748	###########	###########	###########
771	0.10	########	########	€ 480,061	€ 640,082	-€ 3,869,247	-€ 7,738,495	###########	###########
772	0.16	########	########	€ 760,834	#########	-€ 6,132,243	###########	###########	###########
773	0.19	########	########	€ 883,650	#########	-€ 7,122,134	###########	###########	###########
774	0.07	########	########	€ 342,778	€ 457,037	-€ 2,762,754	-€ 5,525,508	-€ 8,288,262	###########
775	0.22	########	########	#########	#########	-€ 8,528,090	###########	###########	###########
776	0.20	########	########	€ 937,392	#########	-€ 7,555,291	###########	###########	###########
777	0.11	########	########	€ 528,931	€ 705,241	-€ 4,263,128	-€ 8,526,256	###########	###########
778	0.14	########	########	€ 650,807	€ 867,743	-€ 5,245,443	###########	###########	###########
779	0.17	########	########	€ 785,786	#########	-€ 6,333,357	###########	###########	###########
780	0.10	########	########	€ 483,167	€ 644,223	-€ 3,894,279	-€ 7,788,559	###########	###########
781	0.11	########	########	€ 530,710	€ 707,614	-€ 4,277,473	-€ 8,554,947	###########	###########
782	0.12	########	########	€ 558,996	€ 745,328	-€ 4,505,453	-€ 9,010,906	###########	###########
783	0.13	########	########	€ 599,294	€ 799,059	-€ 4,830,253	-€ 9,660,506	###########	###########
784	0.27	########	########	#########	#########	###########	###########	###########	###########
785	0.14	########	########	€ 681,645	€ 908,860	-€ 5,493,990	###########	###########	###########
786	0.14	########	########	€ 659,006	€ 878,674	-€ 5,311,519	###########	###########	###########
787	0.22	########	########	#########	#########	-€ 8,552,972	###########	###########	###########
788	0.17	########	########	€ 795,755	#########	-€ 6,413,706	###########	###########	###########
789	0.21	########	########	€ 999,921	#########	-€ 8,059,261	###########	###########	###########
790	0.15	########	########	€ 731,147	€ 974,863	-€ 5,892,974	###########	###########	###########
791	0.16	########	########	€ 757,971	#########	-€ 6,109,173	###########	###########	###########
792	0.17	########	########	€ 783,630	#########	-€ 6,315,982	###########	###########	###########
793	0.15	########	########	€ 724,990	€ 966,654	-€ 5,843,350	###########	###########	###########
794	0.18	########	########	€ 830,430	#########	-€ 6,693,180	###########	###########	###########
795	0.20	########	########	€ 960,654	#########	-€ 7,742,778	###########	###########	###########
796	0.12	########	########	€ 562,958	€ 750,610	-€ 4,537,383	-€ 9,074,766	###########	###########
797	0.17	########	########	€ 803,388	#########	-€ 6,475,225	###########	###########	###########
798	0.22	########	########	#########	#########	-€ 8,255,570	###########	###########	###########
799	0.18	########	########	€ 861,422	#########	-€ 6,942,975	###########	###########	###########
800	0.17	########	########	€ 783,881	#########	-€ 6,318,000	###########	###########	###########
801	0.21	########	########	€ 991,354	#########	-€ 7,990,211	###########	###########	###########
802	0.21	########	########	€ 969,786	#########	-€ 7,816,380	###########	###########	###########
803	0.16	########	########	€ 743,195	€ 990,926	-€ 5,990,074	###########	###########	###########
804	0.20	########	########	€ 931,961	#########	-€ 7,511,513	###########	###########	###########
805	0.16	########	########	€ 756,741	#########	-€ 6,099,261	###########	###########	###########
806	0.11	########	########	€ 539,799	€ 719,732	-€ 4,350,728	-€ 8,701,455	###########	###########
807	0.10	########	########	€ 477,490	€ 636,653	-€ 3,848,521	-€ 7,697,041	###########	###########
808	0.12	########	########	€ 583,264	€ 777,686	-€ 4,701,052	-€ 9,402,103	###########	###########
809	0.11	########	########	€ 535,813	€ 714,417	-€ 4,318,600	-€ 8,637,201	###########	###########
810	0.24	########	########	#########	#########	-€ 9,115,292	###########	###########	###########
811	0.25	########	########	#########	#########	-€ 9,573,087	###########	###########	###########
812	0.22	########	########	#########	#########	-€ 8,412,742	###########	###########	###########
813	0.16	########	########	€ 750,876	#########	-€ 6,051,990	###########	###########	###########
814	0.10	########	########	€ 490,623	€ 654,164	-€ 3,954,370	-€ 7,908,741	###########	###########
815	0.13	########	########	€ 617,456	€ 823,274	-€ 4,976,633	-€ 9,953,265	###########	###########
816	0.13	########	########	€ 615,859	€ 821,146	-€ 4,963,764	-€ 9,927,528	###########	###########
817	0.16	########	########	€ 777,425	#########	-€ 6,265,968	###########	###########	###########
818	0.13	########	########	€ 634,165	€ 845,553	-€ 5,111,306	###########	###########	###########
819	0.12	########	########	€ 568,242	€ 757,656	-€ 4,579,975	-€ 9,159,950	###########	###########
820	0.13	########	########	€ 626,510	€ 835,347	-€ 5,049,612	###########	###########	###########
821	0.15	########	########	€ 717,356	€ 956,475	-€ 5,781,818	###########	###########	###########
822	0.11	########	########	€ 512,592	€ 683,456	-€ 4,131,439	-€ 8,262,877	###########	###########
823	0.12	########	########	€ 573,657	€ 764,876	-€ 4,623,622	-€ 9,247,243	###########	###########
824	0.19	########	########	€ 895,542	#########	-€ 7,217,981	###########	###########	###########
825	0.13	########	########	€ 619,967	€ 826,622	-€ 4,996,870	-€ 9,993,740	###########	###########
826	0.20	########	########	€ 953,018	#########	-€ 7,681,235	###########	###########	###########
827	0.15	########	########	€ 697,123	€ 929,498	-€ 5,618,745	###########	###########	###########
828	0.14	########	########	€ 667,365	€ 889,821	-€ 5,378,899	###########	###########	###########
829	0.17	########	########	€ 779,424	#########	-€ 6,282,078	###########	###########	###########
830	0.12	########	########	€ 544,121	€ 725,495	-€ 4,385,563	-€ 8,771,126	###########	###########
831	0.15	########	########	€ 725,946	€ 967,929	-€ 5,851,057	###########	###########	###########
832	0.15	########	########	€ 730,695	€ 974,259	-€ 5,889,326	###########	###########	###########
833	0.19	########	########	€ 884,121	#########	-€ 7,125,929	###########	###########	###########
834	0.13	########	########	€ 598,569	€ 798,092	-€ 4,824,409	-€ 9,648,817	###########	###########
835	0.17	########	########	€ 779,449	#########	-€ 6,282,282	###########	###########	###########
836	0.13	########	########	€ 612,201	€ 816,268	-€ 4,934,277	-€ 9,868,554	###########	###########
837	0.17	########	########	€ 811,552	#########	-€ 6,541,032	###########	###########	###########
838	0.23	########	########	#########	#########	-€ 8,904,416	###########	###########	###########
839	0.14	########	########	€ 638,653	€ 851,537	-€ 5,147,480	###########	###########	###########
840	0.19	########	########	€ 878,455	#########	-€ 7,080,261	###########	###########	###########
841	0.15	########	########	€ 697,477	€ 929,969	-€ 5,621,593	###########	###########	###########
842	0.16	########	########	€ 760,939	#########	-€ 6,133,097	###########	###########	###########
843	0.15	########	########	€ 701,226	€ 934,968	-€ 5,651,814	###########	###########	###########
844	0.12	########	########	€ 558,854	€ 745,138	-€ 4,504,307	-€ 9,008,614	###########	###########
845	0.16	########	########	€ 776,946	#########	-€ 6,262,111	###########	###########	###########
846	0.16	########	########	€ 750,263	#########	-€ 6,047,048	###########	###########	###########
847	0.13	########	########	€ 591,986	€ 789,314	-€ 4,771,345	-€ 9,542,691	###########	###########
848	0.20	########	########	€ 949,948	#########	-€ 7,656,491	###########	###########	###########
849	0.11	########	########	€ 541,430	€ 721,907	-€ 4,363,876	-€ 8,727,751	###########	###########
850	0.25	########	########	#########	#########	-€ 9,529,462	###########	###########	###########
851	0.09	########	########	€ 406,231	€ 541,642	-€ 3,274,184	-€ 6,548,367	-€ 9,822,551	###########
852	0.14	########	########	€ 673,550	€ 898,067	-€ 5,428,748	###########	###########	###########
853	0.13	########	########	€ 605,668	€ 807,558	-€ 4,881,625	-€ 9,763,251	###########	###########
854	0.18	########	########	€ 828,205	#########	-€ 6,675,247	###########	###########	###########
855	0.11	########	########	€ 516,924	€ 689,232	-€ 4,166,357	-€ 8,332,715	###########	###########
856	0.20	########	########	€ 958,169	#########	-€ 7,722,750	###########	###########	###########

857	0.21	########	########	€ 992,181	#########	-€ 7,996,884	###########	###########	###########
858	0.17	########	########	€ 819,563	#########	-€ 6,605,598	###########	###########	###########
859	0.16	########	########	€ 745,772	€ 994,363	-€ 6,010,848	###########	###########	###########
860	0.17	########	########	€ 799,999	#########	-€ 6,447,914	###########	###########	###########
861	0.20	########	########	€ 935,612	#########	-€ 7,540,938	###########	###########	###########
862	0.17	########	########	€ 810,229	#########	-€ 6,530,364	###########	###########	###########
863	0.29	########	########	#########	#########	###########	###########	###########	###########
864	0.21	########	########	€ 982,482	#########	-€ 7,918,710	###########	###########	###########
865	0.20	########	########	€ 956,151	#########	-€ 7,706,484	###########	###########	###########
866	0.18	########	########	€ 849,483	#########	-€ 6,846,745	###########	###########	###########
867	0.22	########	########	#########	#########	-€ 8,234,769	###########	###########	###########
868	0.12	########	########	€ 582,850	€ 777,134	-€ 4,697,716	-€ 9,395,433	###########	###########
869	0.15	########	########	€ 703,956	€ 938,608	-€ 5,673,817	###########	###########	###########
870	0.13	########	########	€ 611,564	€ 815,419	-€ 4,929,145	-€ 9,858,290	###########	###########
871	0.19	########	########	€ 916,545	#########	-€ 7,387,266	###########	###########	###########
872	0.12	########	########	€ 579,199	€ 772,266	-€ 4,668,288	-€ 9,336,577	###########	###########
873	0.16	########	########	€ 735,550	€ 980,734	-€ 5,928,462	###########	###########	###########
874	0.21	########	########	€ 994,518	#########	-€ 8,015,720	###########	###########	###########
875	0.12	########	########	€ 557,770	€ 743,693	-€ 4,495,568	-€ 8,991,135	###########	###########
876	0.14	########	########	€ 682,374	€ 909,832	-€ 5,499,866	###########	###########	###########
877	0.18	########	########	€ 832,851	#########	-€ 6,712,695	###########	###########	###########
878	0.17	########	########	€ 794,143	#########	-€ 6,400,712	###########	###########	###########
879	0.14	########	########	€ 684,235	€ 912,314	-€ 5,514,868	###########	###########	###########
880	0.14	########	########	€ 684,153	€ 912,204	-€ 5,514,206	###########	###########	###########
881	0.16	########	########	€ 745,068	€ 993,424	-€ 6,005,174	###########	###########	###########
882	0.15	########	########	€ 723,300	€ 964,400	-€ 5,829,729	###########	###########	###########
883	0.19	########	########	€ 875,387	#########	-€ 7,055,530	###########	###########	###########
884	0.15	########	########	€ 691,814	€ 922,419	-€ 5,575,952	###########	###########	###########
885	0.22	########	########	#########	#########	-€ 8,534,782	###########	###########	###########
886	0.20	########	########	€ 943,613	#########	-€ 7,605,430	###########	###########	###########
887	0.15	########	########	€ 706,926	€ 942,568	-€ 5,697,754	###########	###########	###########
888	0.18	########	########	€ 855,491	#########	-€ 6,895,172	###########	###########	###########
889	0.18	########	########	€ 828,191	#########	-€ 6,675,140	###########	###########	###########
890	0.14	########	########	€ 684,088	€ 912,117	-€ 5,513,682	###########	###########	###########
891	0.17	########	########	€ 789,654	#########	-€ 6,364,530	###########	###########	###########
892	0.12	########	########	€ 546,569	€ 728,758	-€ 4,405,290	-€ 8,810,580	###########	###########
893	0.12	########	########	€ 581,205	€ 774,940	-€ 4,684,455	-€ 9,368,910	###########	###########
894	0.16	########	########	€ 753,107	#########	-€ 6,069,967	###########	###########	###########
895	0.14	########	########	€ 653,968	€ 871,958	-€ 5,270,919	###########	###########	###########
896	0.13	########	########	€ 633,894	€ 845,192	-€ 5,109,124	###########	###########	###########
897	0.16	########	########	€ 774,669	#########	-€ 6,243,752	###########	###########	###########
898	0.16	########	########	€ 777,074	#########	-€ 6,263,137	###########	###########	###########
899	0.20	########	########	€ 928,630	#########	-€ 7,484,665	###########	###########	###########
900	0.18	########	########	€ 837,697	#########	-€ 6,751,755	###########	###########	###########
901	0.11	########	########	€ 508,369	€ 677,825	-€ 4,097,404	-€ 8,194,807	###########	###########
902	0.19	########	########	€ 901,556	#########	-€ 7,266,455	###########	###########	###########
903	0.17	########	########	€ 782,376	#########	-€ 6,305,875	###########	###########	###########
904	0.12	########	########	€ 577,691	€ 770,255	-€ 4,656,133	-€ 9,312,265	###########	###########
905	0.16	########	########	€ 773,155	#########	-€ 6,231,557	###########	###########	###########
906	0.17	########	########	€ 782,693	#########	-€ 6,308,429	###########	###########	###########
907	0.18	########	########	€ 841,065	#########	-€ 6,778,901	###########	###########	###########
908	0.18	########	########	€ 856,238	#########	-€ 6,901,191	###########	###########	###########
909	0.19	########	########	€ 918,840	#########	-€ 7,405,757	###########	###########	###########
910	0.22	########	########	#########	#########	-€ 8,248,703	###########	###########	###########
911	0.16	########	########	€ 759,031	#########	-€ 6,117,716	###########	###########	###########
912	0.21	########	########	#########	#########	-€ 8,089,139	###########	###########	###########
913	0.15	########	########	€ 709,023	€ 945,364	-€ 5,714,657	###########	###########	###########
914	0.14	########	########	€ 645,833	€ 861,110	-€ 5,205,347	###########	###########	###########
915	0.13	########	########	€ 629,490	€ 839,320	-€ 5,073,625	###########	###########	###########
916	0.15	########	########	€ 695,512	€ 927,349	-€ 5,605,754	###########	###########	###########
917	0.13	########	########	€ 591,916	€ 789,221	-€ 4,770,782	-€ 9,541,564	###########	###########
918	0.14	########	########	€ 672,141	€ 896,188	-€ 5,417,390	###########	###########	###########
919	0.09	########	########	€ 423,723	€ 564,964	-€ 3,415,167	-€ 6,830,333	###########	###########
920	0.09	########	########	€ 410,461	€ 547,281	-€ 3,308,274	-€ 6,616,548	-€ 9,924,822	###########
921	0.19	########	########	€ 900,796	#########	-€ 7,260,329	###########	###########	###########
922	0.21	########	########	€ 995,233	#########	-€ 8,021,481	###########	###########	###########
923	0.13	########	########	€ 602,919	€ 803,892	-€ 4,859,467	-€ 9,718,934	###########	###########
924	0.17	########	########	€ 788,218	#########	-€ 6,352,963	###########	###########	###########
925	0.14	########	########	€ 665,442	€ 887,256	-€ 5,363,398	###########	###########	###########
926	0.15	########	########	€ 695,749	€ 927,666	-€ 5,607,670	###########	###########	###########
927	0.14	########	########	€ 648,861	€ 865,148	-€ 5,229,755	###########	###########	###########
928	0.18	########	########	€ 851,587	#########	-€ 6,863,705	###########	###########	###########
929	0.14	########	########	€ 647,357	€ 863,143	-€ 5,217,636	###########	###########	###########
930	0.17	########	########	€ 790,331	#########	-€ 6,369,992	###########	###########	###########
931	0.10	########	########	€ 485,729	€ 647,638	-€ 3,914,924	-€ 7,829,849	###########	###########
932	0.23	########	########	#########	#########	-€ 8,936,776	###########	###########	###########
933	0.16	########	########	€ 741,659	€ 988,879	-€ 5,977,699	###########	###########	###########
934	0.19	########	########	€ 907,990	#########	-€ 7,318,313	###########	###########	###########
935	0.19	########	########	€ 889,441	#########	-€ 7,168,804	###########	###########	###########
936	0.15	########	########	€ 687,678	€ 916,904	-€ 5,542,618	###########	###########	###########
937	0.14	########	########	€ 651,989	€ 869,318	-€ 5,254,964	###########	###########	###########
938	0.13	########	########	€ 598,816	€ 798,421	-€ 4,826,396	-€ 9,652,791	###########	###########
939	0.14	########	########	€ 660,979	€ 881,305	-€ 5,327,426	###########	###########	###########
940	0.17	########	########	€ 815,711	#########	-€ 6,574,551	###########	###########	###########
941	0.10	########	########	€ 491,945	€ 655,927	-€ 3,965,031	-€ 7,930,062	###########	###########
942	0.08	########	########	€ 382,248	€ 509,664	-€ 3,080,880	-€ 6,161,759	-€ 9,242,639	###########
943	0.12	########	########	€ 552,212	€ 736,282	-€ 4,450,772	-€ 8,901,545	###########	###########
944	0.15	########	########	€ 731,741	€ 975,654	-€ 5,897,759	###########	###########	###########
945	0.18	########	########	€ 832,624	#########	-€ 6,710,863	###########	###########	###########
946	0.14	########	########	€ 674,484	€ 899,311	-€ 5,436,271	###########	###########	###########
947	0.13	########	########	€ 606,344	€ 808,458	-€ 4,887,069	-€ 9,774,139	###########	###########
948	0.23	########	########	#########	#########	-€ 8,668,576	###########	###########	###########
949	0.13	########	########	€ 620,466	€ 827,289	-€ 5,000,898	###########	###########	###########
950	0.13	########	########	€ 593,521	€ 791,362	-€ 4,783,723	-€ 9,567,447	###########	###########
951	0.18	########	########	€ 834,421	#########	-€ 6,725,352	###########	###########	###########
952	0.18	########	########	€ 857,033	#########	-€ 6,907,600	###########	###########	###########
953	0.21	########	########	€ 979,197	#########	-€ 7,892,232	###########	###########	###########
954	0.19	########	########	€ 896,908	#########	-€ 7,228,992	###########	###########	###########
955	0.16	########	########	€ 772,122	#########	-€ 6,223,227	###########	###########	###########
956	0.28	########	########	#########	#########	###########	###########	###########	###########
957	0.09	########	########	€ 430,469	€ 573,958	-€ 3,469,535	-€ 6,939,070	###########	###########
958	0.12	########	########	€ 586,962	€ 782,616	-€ 4,730,858	-€ 9,461,715	###########	###########
959	0.17	########	########	€ 821,668	#########	-€ 6,622,562	###########	###########	###########
960	0.24	########	########	#########	#########	-€ 9,102,721	###########	###########	###########
961	0.15	########	########	€ 718,504	€ 958,006	-€ 5,791,072	###########	###########	###########
962	0.25	########	########	#########	#########	-€ 9,352,091	###########	###########	###########
963	0.19	########	########	€ 901,480	#########	-€ 7,265,843	###########	###########	###########
964	0.19	########	########	€ 917,432	#########	-€ 7,394,407	###########	###########	###########
965	0.11	########	########	€ 503,064	€ 670,753	-€ 4,054,649	-€ 8,109,298	###########	###########
966	0.10	########	########	€ 480,976	€ 641,301	-€ 3,876,620	-€ 7,753,239	###########	###########
967	0.14	########	########	€ 649,745	€ 866,327	-€ 5,236,881	###########	###########	###########
968	0.21	########	########	#########	#########	-€ 8,128,962	###########	###########	###########
969	0.10	########	########	€ 486,476	€ 648,635	-€ 3,920,948	-€ 7,841,897	###########	###########
970	0.20	########	########	€ 938,624	#########	-€ 7,565,216	###########	###########	###########
971	0.17	########	########	€ 810,087	#########	-€ 6,529,221	###########	###########	###########
972	0.19	########	########	€ 875,318	#########	-€ 7,054,973	###########	###########	###########
973	0.12	########	########	€ 545,338	€ 727,118	-€ 4,395,371	-€ 8,790,743	###########	###########
974	0.13	########	########	€ 613,047	€ 817,396	-€ 4,941,098	-€ 9,882,196	###########	###########
975	0.11	########	########	€ 520,528	€ 694,037	-€ 4,195,402	-€ 8,390,804	###########	###########
976	0.19	########	########	€ 878,382	#########	-€ 7,079,672	###########	###########	###########
977	0.14	########	########	€ 649,868	€ 866,491	-€ 5,237,875	###########	###########	###########
978	0.14	########	########	€ 667,061	€ 889,415	-€ 5,376,449	###########	###########	###########
979	0.17	########	########	€ 805,124	#########	-€ 6,489,217	###########	###########	###########
980	0.13	########	########	€ 615,133	€ 820,178	-€ 4,957,913	-€ 9,915,826	###########	###########
981	0.13	########	########	€ 617,720	€ 823,627	-€ 4,978,765	-€ 9,957,530	###########	###########
982	0.17	########	########	€ 805,296	#########	-€ 6,490,604	###########	###########	###########
983	0.12	########	########	€ 588,674	€ 784,898	-€ 4,744,653	-€ 9,489,306	###########	###########
984	0.17	########	########	€ 817,683	#########	-€ 6,590,443	###########	###########	###########
985	0.16	########	########	€ 767,837	#########	-€ 6,188,694	###########	###########	###########
986	0.23	########	########	#########	#########	-€ 8,891,169	###########	###########	###########
987	0.21	########	########	€ 980,283	#########	-€ 7,900,987	###########	###########	###########
988	0.17	########	########	€ 814,630	#########	-€ 6,565,834	###########	###########	###########
989	0.14	########	########	€ 658,323	€ 877,764	-€ 5,306,017	###########	###########	###########
990	0.18	########	########	€ 855,356	#########	-€ 6,894,088	###########	###########	###########
991	0.18	########	########	€ 872,998	#########	-€ 7,036,277	###########	###########	###########
992	0.23	########	########	#########	#########	-€ 8,894,696	###########	###########	###########
993	0.13	########	########	€ 634,415	€ 845,887	-€ 5,113,323	###########	###########	###########
994	0.16	########	########	€ 762,881	#########	-€ 6,148,746	###########	###########	###########
995	0.17	########	########	€ 808,741	#########	-€ 6,518,372	###########	###########	###########
996	0.13	########	########	€ 618,184	€ 824,245	-€ 4,982,498	-€ 9,964,996	###########	###########
997	0.15	########	########	€ 707,519	€ 943,359	-€ 5,702,536	###########	###########	###########
998	0.20	########	########	€ 957,500	#########	-€ 7,717,358	###########	###########	###########
999	0.15	########	########	€ 698,435	€ 931,247	-€ 5,629,317	###########	###########	###########
1000	0.17	########	########	€ 779,625	#########	-€ 6,283,696	###########	###########	###########
1001	0.20	########	########	€ 936,410	#########	-€ 7,547,371	###########	###########	###########
1002	0.15	########	########	€ 705,123	€ 940,164	-€ 5,683,223	###########	###########	###########
1003	0.31	########	########	#########	#########	###########	###########	###########	###########
1004	0.20	########	########	€ 924,176	#########	-€ 7,448,765	###########	###########	###########
1005	0.15	########	########	€ 695,936	€ 927,915	-€ 5,609,179	###########	###########	###########
1006	0.18	########	########	€ 827,328	#########	-€ 6,668,184	###########	###########	###########
1007	0.11	########	########	€ 511,943	€ 682,590	-€ 4,126,209	-€ 8,252,418	###########	###########
1008	0.12	########	########	€ 575,465	€ 767,287	-€ 4,638,192	-€ 9,276,383	###########	###########
1009	0.12	########	########	€ 543,567	€ 724,755	-€ 4,381,093	-€ 8,762,186	###########	###########
1010	0.17	########	########	€ 803,341	#########	-€ 6,474,849	###########	###########	###########
1011	0.10	########	########	€ 495,955	€ 661,274	-€ 3,997,350	-€ 7,994,700	###########	###########
1012	0.22	########	########	#########	#########	-€ 8,424,897	###########	###########	###########
1013	0.16	########	########	€ 732,456	€ 976,608	-€ 5,903,526	###########	###########	###########
1014	0.14	########	########	€ 682,729	€ 910,305	-€ 5,502,728	###########	###########	###########
1015	0.15	########	########	€ 719,123	€ 958,831	-€ 5,796,063	###########	###########	###########
1016	0.12	########	########	€ 585,568	€ 780,757	-€ 4,719,616	-€ 9,439,233	###########	###########
1017	0.15	########	########	€ 693,065	€ 924,087	-€ 5,586,036	###########	###########	###########
1018	0.10	########	########	€ 465,560	€ 620,747	-€ 3,752,369	-€ 7,504,737	###########	###########
1019	0.11	########	########	€ 529,380	€ 705,840	-€ 4,266,752	-€ 8,533,503	###########	###########
1020	0.17	########	########	€ 815,884	#########	-€ 6,575,944	###########	###########	###########
1021	0.30	########	########	#########	#########	###########	###########	###########	###########
1022	0.19	########	########	€ 912,233	#########	-€ 7,352,504	###########	###########	###########
1023	0.12	########	########	€ 553,771	€ 738,361	-€ 4,463,340	-€ 8,926,679	###########	###########
1024	0.20	########	########	€ 949,867	#########	-€ 7,655,833	###########	###########	###########
1025	0.18	########	########	€ 833,191	#########	-€ 6,715,438	###########	###########	###########
1026	0.12	########	########	€ 560,017	€ 746,690	-€ 4,513,684	-€ 9,027,368	###########	###########
1027	0.19	########	########	€ 918,212	#########	-€ 7,400,700	###########	###########	###########
1028	0.19	########	########	€ 910,734	#########	-€ 7,340,429	###########	###########	###########

1029	0.14	########	########	€ 677,851	€ 903,801	-€ 5,463,408	###########	###########	###########
1030	0.17	########	########	€ 807,127	#########	-€ 6,505,362	###########	###########	###########
1031	0.15	########	########	€ 711,939	€ 949,251	-€ 5,738,154	###########	###########	###########
1032	0.17	########	########	€ 806,974	#########	-€ 6,504,135	###########	###########	###########
1033	0.16	########	########	€ 755,079	#########	-€ 6,085,864	###########	###########	###########
1034	0.18	########	########	€ 860,112	#########	-€ 6,932,415	###########	###########	###########
1035	0.16	########	########	€ 768,370	#########	-€ 6,192,985	###########	###########	###########
1036	0.13	########	########	€ 625,455	€ 833,940	-€ 5,041,105	###########	###########	###########
1037	0.17	########	########	€ 818,852	#########	-€ 6,599,870	###########	###########	###########
1038	0.16	########	########	€ 750,375	#########	-€ 6,047,948	###########	###########	###########
1039	0.10	########	########	€ 480,374	€ 640,498	-€ 3,871,765	-€ 7,743,529	###########	###########
1040	0.11	########	########	€ 499,468	€ 665,958	-€ 4,025,666	-€ 8,051,333	###########	###########
1041	0.16	########	########	€ 735,351	€ 980,468	-€ 5,926,859	###########	###########	###########
1042	0.17	########	########	€ 820,594	#########	-€ 6,613,909	###########	###########	###########
1043	0.21	########	########	€ 992,346	#########	-€ 7,998,214	###########	###########	###########
1044	0.14	########	########	€ 684,783	€ 913,044	-€ 5,519,282	###########	###########	###########
1045	0.19	########	########	€ 891,008	#########	-€ 7,181,434	###########	###########	###########
1046	0.11	########	########	€ 529,817	€ 706,422	-€ 4,270,271	-€ 8,540,542	###########	###########
1047	0.17	########	########	€ 786,356	#########	-€ 6,337,953	###########	###########	###########
1048	0.17	########	########	€ 789,568	#########	-€ 6,363,841	###########	###########	###########
1049	0.20	########	########	€ 924,726	#########	-€ 7,453,199	###########	###########	###########
1050	0.12	########	########	€ 580,445	€ 773,927	-€ 4,678,332	-€ 9,356,664	###########	###########
1051	0.21	########	########	€ 972,328	#########	-€ 7,836,869	###########	###########	###########
1052	0.16	########	########	€ 750,090	#########	-€ 6,045,652	###########	###########	###########
1053	0.21	########	########	#########	#########	-€ 8,159,398	###########	###########	###########
1054	0.21	########	########	€ 997,802	#########	-€ 8,042,185	###########	###########	###########
1055	0.25	########	########	#########	#########	-€ 9,443,419	###########	###########	###########
1056	0.15	########	########	€ 690,034	€ 920,046	-€ 5,561,607	###########	###########	###########
1057	0.11	########	########	€ 516,360	€ 688,480	-€ 4,161,811	-€ 8,323,622	###########	###########
1058	0.15	########	########	€ 718,159	€ 957,545	-€ 5,788,289	###########	###########	###########
1059	0.24	########	########	#########	#########	-€ 9,265,601	###########	###########	###########
1060	0.17	########	########	€ 790,333	#########	-€ 6,370,007	###########	###########	###########
1061	0.23	########	########	#########	#########	-€ 8,912,584	###########	###########	###########
1062	0.20	########	########	€ 964,740	#########	-€ 7,775,713	###########	###########	###########
1063	0.10	########	########	€ 482,962	€ 643,949	-€ 3,892,622	-€ 7,785,245	###########	###########
1064	0.17	########	########	€ 798,893	#########	-€ 6,439,000	###########	###########	###########
1065	0.14	########	########	€ 642,679	€ 856,906	-€ 5,179,931	###########	###########	###########
1066	0.18	########	########	€ 847,625	#########	-€ 6,831,777	###########	###########	###########
1067	0.10	########	########	€ 470,222	€ 626,962	-€ 3,789,939	-€ 7,579,878	###########	###########
1068	0.19	########	########	€ 885,601	#########	-€ 7,137,858	###########	###########	###########
1069	0.19	########	########	€ 887,288	#########	-€ 7,151,457	###########	###########	###########
1070	0.23	########	########	#########	#########	-€ 8,749,148	###########	###########	###########
1071	0.15	########	########	€ 697,888	€ 930,518	-€ 5,624,911	###########	###########	###########
1072	0.21	########	########	€ 970,952	#########	-€ 7,825,781	###########	###########	###########
1073	0.14	########	########	€ 654,486	€ 872,649	-€ 5,275,095	###########	###########	###########
1074	0.15	########	########	€ 691,009	€ 921,346	-€ 5,569,468	###########	###########	###########
1075	0.08	########	########	€ 399,268	€ 532,357	-€ 3,218,060	-€ 6,436,120	-€ 9,654,180	###########
1076	0.19	########	########	€ 892,245	#########	-€ 7,191,407	###########	###########	###########
1077	0.16	########	########	€ 772,130	#########	-€ 6,223,293	###########	###########	###########
1078	0.15	########	########	€ 725,610	€ 967,480	-€ 5,848,342	###########	###########	###########
1079	0.15	########	########	€ 696,707	€ 928,942	-€ 5,615,388	###########	###########	###########
1080	0.22	########	########	#########	#########	-€ 8,397,205	###########	###########	###########
1081	0.16	########	########	€ 760,830	#########	-€ 6,132,212	###########	###########	###########
1082	0.17	########	########	€ 808,700	#########	-€ 6,518,043	###########	###########	###########
1083	0.13	########	########	€ 621,214	€ 828,285	-€ 5,006,923	###########	###########	###########
1084	0.17	########	########	€ 784,038	#########	-€ 6,319,272	###########	###########	###########
1085	0.19	########	########	€ 904,216	#########	-€ 7,287,890	###########	###########	###########
1086	0.20	########	########	€ 934,900	#########	-€ 7,535,204	###########	###########	###########
1087	0.16	########	########	€ 745,395	€ 993,860	-€ 6,007,809	###########	###########	###########
1088	0.13	########	########	€ 608,595	€ 811,460	-€ 4,905,218	-€ 9,810,436	###########	###########
1089	0.17	########	########	€ 824,406	#########	-€ 6,644,634	###########	###########	###########
1090	0.14	########	########	€ 638,783	€ 851,710	-€ 5,148,525	###########	###########	###########
1091	0.17	########	########	€ 803,880	#########	-€ 6,479,190	###########	###########	###########
1092	0.27	########	########	#########	#########	###########	###########	###########	###########
1093	0.18	########	########	€ 840,337	#########	-€ 6,773,030	###########	###########	###########
1094	0.15	########	########	€ 695,738	€ 927,651	-€ 5,607,581	###########	###########	###########
1095	0.20	########	########	€ 967,783	#########	-€ 7,800,234	###########	###########	###########
1096	0.20	########	########	€ 951,496	#########	-€ 7,668,967	###########	###########	###########
1097	0.18	########	########	€ 857,407	#########	-€ 6,910,616	###########	###########	###########
1098	0.10	########	########	€ 464,275	€ 619,034	-€ 3,742,014	-€ 7,484,029	###########	###########
1099	0.17	########	########	€ 805,671	#########	-€ 6,493,632	###########	###########	###########
1100	0.22	########	########	#########	#########	-€ 8,371,925	###########	###########	###########
1101	0.17	########	########	€ 823,915	#########	-€ 6,640,676	###########	###########	###########
1102	0.16	########	########	€ 763,006	#########	-€ 6,149,754	###########	###########	###########
1103	0.13	########	########	€ 598,007	€ 797,342	-€ 4,819,874	-€ 9,639,749	###########	###########
1104	0.12	########	########	€ 557,598	€ 743,464	-€ 4,494,184	-€ 8,988,368	###########	###########
1105	0.18	########	########	€ 862,356	#########	-€ 6,950,504	###########	###########	###########
1106	0.12	########	########	€ 569,901	€ 759,869	-€ 4,593,349	-€ 9,186,698	###########	###########
1107	0.12	########	########	€ 584,378	€ 779,171	-€ 4,710,032	-€ 9,420,063	###########	###########
1108	0.15	########	########	€ 714,637	€ 952,849	-€ 5,759,903	###########	###########	###########
1109	0.15	########	########	€ 699,547	€ 932,729	-€ 5,638,276	###########	###########	###########
1110	0.16	########	########	€ 778,234	#########	-€ 6,272,486	###########	###########	###########
1111	0.18	########	########	€ 847,736	#########	-€ 6,832,670	###########	###########	###########
1112	0.15	########	########	€ 697,714	€ 930,285	-€ 5,623,504	###########	###########	###########
1113	0.17	########	########	€ 824,793	#########	-€ 6,647,752	###########	###########	###########
1114	0.20	########	########	€ 932,239	#########	-€ 7,513,757	###########	###########	###########
1115	0.15	########	########	€ 689,517	€ 919,356	-€ 5,557,440	###########	###########	###########
1116	0.21	########	########	€ 989,103	#########	-€ 7,972,069	###########	###########	###########
1117	0.17	########	########	€ 796,000	#########	-€ 6,415,678	###########	###########	###########
1118	0.14	########	########	€ 656,542	€ 875,390	-€ 5,291,667	###########	###########	###########
1119	0.14	########	########	€ 655,362	€ 873,817	-€ 5,282,156	###########	###########	###########
1120	0.13	########	########	€ 601,399	€ 801,865	-€ 4,847,216	-€ 9,694,432	###########	###########
1121	0.18	########	########	€ 834,341	#########	-€ 6,724,704	###########	###########	###########
1122	0.10	########	########	€ 477,444	€ 636,592	-€ 3,848,151	-€ 7,696,302	###########	###########
1123	0.15	########	########	€ 693,903	€ 925,204	-€ 5,592,792	###########	###########	###########
1124	0.13	########	########	€ 635,418	€ 847,225	-€ 5,121,410	###########	###########	###########
1125	0.15	########	########	€ 717,794	€ 957,058	-€ 5,785,345	###########	###########	###########
1126	0.15	########	########	€ 727,964	€ 970,619	-€ 5,867,318	###########	###########	###########
1127	0.17	########	########	€ 818,410	#########	-€ 6,596,307	###########	###########	###########
1128	0.19	########	########	€ 876,516	#########	-€ 7,064,631	###########	###########	###########
1129	0.14	########	########	€ 645,673	€ 860,898	-€ 5,204,064	###########	###########	###########
1130	0.09	########	########	€ 429,936	€ 573,249	-€ 3,465,245	-€ 6,930,491	###########	###########
1131	0.21	########	########	#########	#########	-€ 8,062,283	###########	###########	###########
1132	0.18	########	########	€ 831,365	#########	-€ 6,700,720	###########	###########	###########
1133	0.21	########	########	€ 993,218	#########	-€ 8,005,239	###########	###########	###########
1134	0.17	########	########	€ 791,977	#########	-€ 6,383,257	###########	###########	###########
1135	0.20	########	########	€ 934,345	#########	-€ 7,530,731	###########	###########	###########
1136	0.16	########	########	€ 752,364	#########	-€ 6,063,976	###########	###########	###########
1137	0.15	########	########	€ 698,849	€ 931,799	-€ 5,632,657	###########	###########	###########
1138	0.14	########	########	€ 666,856	€ 889,141	-€ 5,374,793	###########	###########	###########
1139	0.11	########	########	€ 521,685	€ 695,580	-€ 4,204,727	-€ 8,409,454	###########	###########
1140	0.11	########	########	€ 533,924	€ 711,898	-€ 4,303,373	-€ 8,606,747	###########	###########
1141	0.15	########	########	€ 728,269	€ 971,025	-€ 5,869,773	###########	###########	###########
1142	0.19	########	########	€ 894,893	#########	-€ 7,212,747	###########	###########	###########
1143	0.18	########	########	€ 848,168	#########	-€ 6,836,151	###########	###########	###########
1144	0.16	########	########	€ 767,846	#########	-€ 6,188,765	###########	###########	###########
1145	0.18	########	########	€ 836,804	#########	-€ 6,744,554	###########	###########	###########
1146	0.19	########	########	€ 913,511	#########	-€ 7,362,808	###########	###########	###########
1147	0.13	########	########	€ 632,095	€ 842,793	-€ 5,094,620	###########	###########	###########
1148	0.13	########	########	€ 632,346	€ 843,128	-€ 5,096,648	###########	###########	###########
1149	0.16	########	########	€ 775,301	#########	-€ 6,248,852	###########	###########	###########
1150	0.17	########	########	€ 781,816	#########	-€ 6,301,362	###########	###########	###########
1151	0.14	########	########	€ 653,084	€ 870,779	-€ 5,263,795	###########	###########	###########
1152	0.13	########	########	€ 608,459	€ 811,279	-€ 4,904,120	-€ 9,808,240	###########	###########
1153	0.11	########	########	€ 506,654	€ 675,539	-€ 4,083,582	-€ 8,167,163	###########	###########
1154	0.18	########	########	€ 857,305	#########	-€ 6,909,794	###########	###########	###########
1155	0.19	########	########	€ 884,222	#########	-€ 7,126,744	###########	###########	###########
1156	0.15	########	########	€ 715,341	€ 953,789	-€ 5,765,581	###########	###########	###########
1157	0.12	########	########	€ 550,611	€ 734,147	-€ 4,437,867	-€ 8,875,734	###########	###########
1158	0.16	########	########	€ 771,929	#########	-€ 6,221,668	###########	###########	###########
1159	0.15	########	########	€ 732,078	€ 976,105	-€ 5,900,480	###########	###########	###########
1160	0.17	########	########	€ 788,381	#########	-€ 6,354,271	###########	###########	###########
1161	0.17	########	########	€ 802,628	#########	-€ 6,469,098	###########	###########	###########
1162	0.18	########	########	€ 839,069	#########	-€ 6,762,812	###########	###########	###########
1163	0.18	########	########	€ 872,554	#########	-€ 7,032,695	###########	###########	###########
1164	0.18	########	########	€ 858,115	#########	-€ 6,916,325	###########	###########	###########
1165	0.15	########	########	€ 720,131	€ 960,175	-€ 5,804,185	###########	###########	###########
1166	0.19	########	########	€ 882,377	#########	-€ 7,111,870	###########	###########	###########
1167	0.19	########	########	€ 880,716	#########	-€ 7,098,487	###########	###########	###########
1168	0.21	########	########	#########	#########	-€ 8,139,740	###########	###########	###########
1169	0.25	########	########	#########	#########	-€ 9,527,027	###########	###########	###########
1170	0.21	########	########	#########	#########	-€ 8,129,394	###########	###########	###########
1171	0.19	########	########	€ 892,368	#########	-€ 7,192,399	###########	###########	###########
1172	0.17	########	########	€ 819,660	#########	-€ 6,606,380	###########	###########	###########
1173	0.18	########	########	€ 840,302	#########	-€ 6,772,750	###########	###########	###########
1174	0.23	########	########	#########	#########	-€ 8,801,926	###########	###########	###########
1175	0.21	########	########	#########	#########	-€ 8,087,285	###########	###########	###########
1176	0.16	########	########	€ 738,578	€ 984,771	-€ 5,952,869	###########	###########	###########
1177	0.14	########	########	€ 679,510	€ 906,013	-€ 5,476,782	###########	###########	###########
1178	0.22	########	########	#########	#########	-€ 8,431,126	###########	###########	###########
1179	0.15	########	########	€ 705,164	€ 940,219	-€ 5,683,554	###########	###########	###########
1180	0.23	########	########	#########	#########	-€ 8,881,338	###########	###########	###########
1181	0.17	########	########	€ 779,953	#########	-€ 6,286,347	###########	###########	###########
1182	0.15	########	########	€ 689,852	€ 919,802	-€ 5,560,135	###########	###########	###########
1183	0.18	########	########	€ 858,968	#########	-€ 6,923,197	###########	###########	###########
1184	0.17	########	########	€ 822,086	#########	-€ 6,625,933	###########	###########	###########
1185	0.14	########	########	€ 658,852	€ 878,469	-€ 5,310,282	###########	###########	###########
1186	0.09	########	########	€ 439,212	€ 585,617	-€ 3,540,008	-€ 7,080,017	###########	###########
1187	0.09	########	########	€ 428,360	€ 571,147	-€ 3,452,539	-€ 6,905,078	###########	###########
1188	0.20	########	########	€ 952,145	#########	-€ 7,674,192	###########	###########	###########
1189	0.17	########	########	€ 826,440	#########	-€ 6,661,026	###########	###########	###########
1190	0.13	########	########	€ 620,583	€ 827,443	-€ 5,001,834	###########	###########	###########
1191	0.20	########	########	€ 951,285	#########	-€ 7,667,262	###########	###########	###########
1192	0.09	########	########	€ 432,180	€ 576,240	-€ 3,483,329	-€ 6,966,659	###########	###########
1193	0.13	########	########	€ 625,928	€ 834,570	-€ 5,044,915	###########	###########	###########
1194	0.14	########	########	€ 657,691	€ 876,921	-€ 5,300,921	###########	###########	###########
1195	0.14	########	########	€ 681,522	€ 908,696	-€ 5,493,000	###########	###########	###########
1196	0.13	########	########	€ 631,536	€ 842,048	-€ 5,090,120	###########	###########	###########
1197	0.19	########	########	€ 902,171	#########	-€ 7,271,412	###########	###########	###########
1198	0.17	########	########	€ 795,095	#########	-€ 6,408,388	###########	###########	###########
1199	0.24	########	########	#########	#########	-€ 9,243,691	###########	###########	###########
1200	0.14	########	########	€ 643,815	€ 858,421	-€ 5,189,089	###########	###########	###########

1201	0.16	########	########	€ 752,928	#########	-€ 6,068,527	###########	###########	###########
1202	0.19	########	########	€ 892,374	#########	-€ 7,192,442	###########	###########	###########
1203	0.24	########	########	#########	#########	-€ 9,303,153	###########	###########	###########
1204	0.13	########	########	€ 637,282	€ 849,710	-€ 5,136,432	###########	###########	###########
1205	0.13	########	########	€ 619,456	€ 825,941	-€ 4,992,754	-€ 9,985,507	###########	###########
1206	0.10	########	########	€ 467,088	€ 622,784	-€ 3,764,686	-€ 7,529,372	###########	###########
1207	0.17	########	########	€ 824,567	#########	-€ 6,645,926	###########	###########	###########
1208	0.23	########	########	#########	#########	-€ 8,667,931	###########	###########	###########
1209	0.19	########	########	€ 902,895	#########	-€ 7,277,246	###########	###########	###########
1210	0.19	########	########	€ 882,250	#########	-€ 7,110,849	###########	###########	###########
1211	0.28	########	########	#########	#########	###########	###########	###########	###########
1212	0.14	########	########	€ 638,810	€ 851,746	-€ 5,148,742	###########	###########	###########
1213	0.16	########	########	€ 768,600	#########	-€ 6,194,840	###########	###########	###########
1214	0.17	########	########	€ 791,208	#########	-€ 6,377,057	###########	###########	###########
1215	0.16	########	########	€ 734,788	€ 979,717	-€ 5,922,315	###########	###########	###########
1216	0.19	########	########	€ 884,606	#########	-€ 7,129,833	###########	###########	###########
1217	0.16	########	########	€ 758,982	#########	-€ 6,117,318	###########	###########	###########
1218	0.19	########	########	€ 920,376	#########	-€ 7,418,139	###########	###########	###########
1219	0.12	########	########	€ 551,238	€ 734,983	-€ 4,442,920	-€ 8,885,840	###########	###########
1220	0.21	########	########	€ 979,217	#########	-€ 7,892,395	###########	###########	###########
1221	0.15	########	########	€ 716,153	€ 954,870	-€ 5,772,121	###########	###########	###########
1222	0.15	########	########	€ 698,374	€ 931,166	-€ 5,628,829	###########	###########	###########
1223	0.09	########	########	€ 417,161	€ 556,214	-€ 3,362,275	-€ 6,724,550	###########	###########
1224	0.18	########	########	€ 835,666	#########	-€ 6,735,384	###########	###########	###########
1225	0.10	########	########	€ 484,575	€ 646,100	-€ 3,905,627	-€ 7,811,255	###########	###########
1226	0.18	########	########	€ 862,649	#########	-€ 6,952,867	###########	###########	###########
1227	0.11	########	########	€ 502,334	€ 669,778	-€ 4,048,759	-€ 8,097,517	###########	###########
1228	0.14	########	########	€ 681,923	€ 909,230	-€ 5,496,230	###########	###########	###########
1229	0.18	########	########	€ 832,271	#########	-€ 6,708,025	###########	###########	###########
1230	0.20	########	########	€ 960,796	#########	-€ 7,743,921	###########	###########	###########
1231	0.08	########	########	€ 362,228	€ 482,970	-€ 2,919,519	-€ 5,839,039	-€ 8,758,558	###########
1232	0.19	########	########	€ 878,198	#########	-€ 7,078,192	###########	###########	###########
1233	0.19	########	########	€ 874,711	#########	-€ 7,050,083	###########	###########	###########
1234	0.17	########	########	€ 802,968	#########	-€ 6,471,845	###########	###########	###########
1235	0.18	########	########	€ 837,023	#########	-€ 6,746,322	###########	###########	###########
1236	0.19	########	########	€ 893,705	#########	-€ 7,203,174	###########	###########	###########
1237	0.17	########	########	€ 795,334	#########	-€ 6,410,310	###########	###########	###########
1238	0.18	########	########	€ 848,555	#########	-€ 6,839,270	###########	###########	###########
1239	0.12	########	########	€ 564,929	€ 753,239	-€ 4,553,274	-€ 9,106,548	###########	###########
1240	0.18	########	########	€ 829,908	#########	-€ 6,688,975	###########	###########	###########
1241	0.08	########	########	€ 374,111	€ 498,815	-€ 3,015,299	-€ 6,030,597	-€ 9,045,896	###########
1242	0.18	########	########	€ 853,905	#########	-€ 6,882,388	###########	###########	###########
1243	0.14	########	########	€ 672,547	€ 896,729	-€ 5,420,662	###########	###########	###########
1244	0.13	########	########	€ 592,957	€ 790,609	-€ 4,779,175	-€ 9,558,351	###########	###########
1245	0.14	########	########	€ 683,742	€ 911,656	-€ 5,510,893	###########	###########	###########
1246	0.12	########	########	€ 590,346	€ 787,128	-€ 4,758,129	-€ 9,516,257	###########	###########
1247	0.13	########	########	€ 595,788	€ 794,384	-€ 4,801,993	-€ 9,603,986	###########	###########
1248	0.18	########	########	€ 860,055	#########	-€ 6,931,959	###########	###########	###########
1249	0.12	########	########	€ 573,866	€ 765,154	-€ 4,625,300	-€ 9,250,599	###########	###########
1250	0.14	########	########	€ 681,611	€ 908,815	-€ 5,493,717	###########	###########	###########
1251	0.16	########	########	€ 745,701	€ 994,268	-€ 6,010,276	###########	###########	###########
1252	0.17	########	########	€ 782,458	#########	-€ 6,306,530	###########	###########	###########
1253	0.23	########	########	#########	#########	-€ 8,654,771	###########	###########	###########
1254	0.17	########	########	€ 798,523	#########	-€ 6,436,017	###########	###########	###########
1255	0.19	########	########	€ 916,621	#########	-€ 7,387,872	###########	###########	###########
1256	0.17	########	########	€ 799,662	#########	-€ 6,445,195	###########	###########	###########
1257	0.22	########	########	#########	#########	-€ 8,463,163	###########	###########	###########
1258	0.24	########	########	#########	#########	-€ 9,038,094	###########	###########	###########
1259	0.14	########	########	€ 653,524	€ 871,366	-€ 5,267,340	###########	###########	###########
1260	0.08	########	########	€ 397,352	€ 529,803	-€ 3,202,619	-€ 6,405,238	-€ 9,607,858	###########
1261	0.14	########	########	€ 644,335	€ 859,113	-€ 5,193,275	###########	###########	###########
1262	0.14	########	########	€ 667,053	€ 889,404	-€ 5,376,381	###########	###########	###########
1263	0.19	########	########	€ 885,484	#########	-€ 7,136,913	###########	###########	###########
1264	0.22	########	########	#########	#########	-€ 8,551,608	###########	###########	###########
1265	0.19	########	########	€ 882,102	#########	-€ 7,109,653	###########	###########	###########
1266	0.17	########	########	€ 784,569	#########	-€ 6,323,551	###########	###########	###########
1267	0.20	########	########	€ 940,054	#########	-€ 7,576,745	###########	###########	###########
1268	0.13	########	########	€ 619,240	€ 825,654	-€ 4,991,015	-€ 9,982,029	###########	###########
1269	0.11	########	########	€ 524,387	€ 699,183	-€ 4,226,511	-€ 8,453,022	###########	###########
1270	0.14	########	########	€ 667,335	€ 889,781	-€ 5,378,658	###########	###########	###########
1271	0.13	########	########	€ 592,176	€ 789,568	-€ 4,772,881	-€ 9,545,762	###########	###########
1272	0.12	########	########	€ 556,304	€ 741,738	-€ 4,483,754	-€ 8,967,507	###########	###########
1273	0.15	########	########	€ 718,919	€ 958,558	-€ 5,794,414	###########	###########	###########
1274	0.15	########	########	€ 700,192	€ 933,590	-€ 5,643,480	###########	###########	###########
1275	0.20	########	########	€ 943,235	#########	-€ 7,602,381	###########	###########	###########
1276	0.15	########	########	€ 724,385	€ 965,846	-€ 5,838,470	###########	###########	###########
1277	0.16	########	########	€ 778,188	#########	-€ 6,272,115	###########	###########	###########
1278	0.11	########	########	€ 514,525	€ 686,033	-€ 4,147,018	-€ 8,294,036	###########	###########
1279	0.22	########	########	#########	#########	-€ 8,274,047	###########	###########	###########
1280	0.11	########	########	€ 496,124	€ 661,498	-€ 3,998,708	-€ 7,997,416	###########	###########
1281	0.16	########	########	€ 772,384	#########	-€ 6,225,338	###########	###########	###########
1282	0.12	########	########	€ 581,037	€ 774,716	-€ 4,683,099	-€ 9,366,198	###########	###########
1283	0.17	########	########	€ 819,303	#########	-€ 6,603,498	###########	###########	###########
1284	0.17	########	########	€ 813,990	#########	-€ 6,560,682	###########	###########	###########
1285	0.19	########	########	€ 878,755	#########	-€ 7,082,677	###########	###########	###########
1286	0.12	########	########	€ 564,378	€ 752,504	-€ 4,548,830	-€ 9,097,660	###########	###########
1287	0.16	########	########	€ 752,869	#########	-€ 6,068,050	###########	###########	###########
1288	0.18	########	########	€ 872,482	#########	-€ 7,032,119	###########	###########	###########
1289	0.13	########	########	€ 609,297	€ 812,396	-€ 4,910,874	-€ 9,821,748	###########	###########
1290	0.22	########	########	#########	#########	-€ 8,296,565	###########	###########	###########
1291	0.15	########	########	€ 694,991	€ 926,655	-€ 5,601,562	###########	###########	###########
1292	0.16	########	########	€ 774,428	#########	-€ 6,241,816	###########	###########	###########
1293	0.14	########	########	€ 664,203	€ 885,604	-€ 5,353,413	###########	###########	###########
1294	0.15	########	########	€ 717,941	€ 957,255	-€ 5,786,533	###########	###########	###########
1295	0.22	########	########	#########	#########	-€ 8,278,233	###########	###########	###########
1296	0.19	########	########	€ 904,540	#########	-€ 7,290,502	###########	###########	###########
1297	0.22	########	########	#########	#########	-€ 8,529,698	###########	###########	###########
1298	0.11	########	########	€ 535,205	€ 713,606	-€ 4,313,696	-€ 8,627,393	###########	###########
1299	0.14	########	########	€ 644,555	€ 859,406	-€ 5,195,046	###########	###########	###########
1300	0.18	########	########	€ 871,800	#########	-€ 7,026,619	###########	###########	###########
1301	0.10	########	########	€ 489,380	€ 652,507	-€ 3,944,358	-€ 7,888,716	###########	###########
1302	0.16	########	########	€ 766,272	#########	-€ 6,176,074	###########	###########	###########
1303	0.18	########	########	€ 834,468	#########	-€ 6,725,727	###########	###########	###########
1304	0.10	########	########	€ 465,495	€ 620,659	-€ 3,751,840	-€ 7,503,680	###########	###########
1305	0.15	########	########	€ 703,527	€ 938,036	-€ 5,670,356	###########	###########	###########
1306	0.20	########	########	€ 960,736	#########	-€ 7,743,436	###########	###########	###########
1307	0.19	########	########	€ 895,863	#########	-€ 7,220,564	###########	###########	###########
1308	0.16	########	########	€ 748,419	€ 997,892	-€ 6,032,180	###########	###########	###########
1309	0.14	########	########	€ 671,973	€ 895,963	-€ 5,416,033	###########	###########	###########
1310	0.11	########	########	€ 535,581	€ 714,108	-€ 4,316,730	-€ 8,633,460	###########	###########
1311	0.24	########	########	#########	#########	-€ 9,263,385	###########	###########	###########
1312	0.11	########	########	€ 535,147	€ 713,529	-€ 4,313,232	-€ 8,626,463	###########	###########
1313	0.17	########	########	€ 809,700	#########	-€ 6,526,105	###########	###########	###########
1314	0.12	########	########	€ 570,038	€ 760,051	-€ 4,594,450	-€ 9,188,901	###########	###########
1315	0.19	########	########	€ 893,673	#########	-€ 7,202,914	###########	###########	###########
1316	0.16	########	########	€ 762,555	#########	-€ 6,146,114	###########	###########	###########
1317	0.08	########	########	€ 389,551	€ 519,401	-€ 3,139,740	-€ 6,279,480	-€ 9,419,219	###########
1318	0.15	########	########	€ 722,928	€ 963,904	-€ 5,826,731	###########	###########	###########
1319	0.20	########	########	€ 933,426	#########	-€ 7,523,318	###########	###########	###########
1320	0.20	########	########	€ 952,483	#########	-€ 7,676,919	###########	###########	###########
1321	0.13	########	########	€ 592,688	€ 790,250	-€ 4,777,004	-€ 9,554,009	###########	###########
1322	0.14	########	########	€ 656,244	€ 874,992	-€ 5,289,265	###########	###########	###########
1323	0.20	########	########	€ 952,439	#########	-€ 7,676,562	###########	###########	###########
1324	0.13	########	########	€ 621,835	€ 829,113	-€ 5,011,925	###########	###########	###########
1325	0.16	########	########	€ 737,766	€ 983,688	-€ 5,946,319	###########	###########	###########
1326	0.10	########	########	€ 485,335	€ 647,114	-€ 3,911,754	-€ 7,823,507	###########	###########
1327	0.21	########	########	€ 991,538	#########	-€ 7,991,699	###########	###########	###########
1328	0.11	########	########	€ 536,695	€ 715,593	-€ 4,325,708	-€ 8,651,415	###########	###########
1329	0.14	########	########	€ 662,339	€ 883,119	-€ 5,338,390	###########	###########	###########
1330	0.15	########	########	€ 690,646	€ 920,861	-€ 5,566,535	###########	###########	###########
1331	0.18	########	########	€ 857,538	#########	-€ 6,911,675	###########	###########	###########
1332	0.17	########	########	€ 805,918	#########	-€ 6,495,622	###########	###########	###########
1333	0.18	########	########	€ 838,386	#########	-€ 6,757,312	###########	###########	###########
1334	0.13	########	########	€ 634,501	€ 846,001	-€ 5,114,016	###########	###########	###########
1335	0.19	########	########	€ 878,109	#########	-€ 7,077,472	###########	###########	###########
1336	0.20	########	########	€ 949,549	#########	-€ 7,653,269	###########	###########	###########
1337	0.15	########	########	€ 700,242	€ 933,656	-€ 5,643,883	###########	###########	###########
1338	0.11	########	########	€ 513,189	€ 684,252	-€ 4,136,251	-€ 8,272,501	###########	###########
1339	0.18	########	########	€ 846,358	#########	-€ 6,821,562	###########	###########	###########
1340	0.09	########	########	€ 412,881	€ 550,507	-€ 3,327,777	-€ 6,655,553	-€ 9,983,330	###########
1341	0.15	########	########	€ 698,243	€ 930,991	-€ 5,627,769	###########	###########	###########
1342	0.17	########	########	€ 820,583	#########	-€ 6,613,821	###########	###########	###########
1343	0.24	########	########	#########	#########	-€ 9,242,780	###########	###########	###########
1344	0.15	########	########	€ 690,830	€ 921,107	-€ 5,568,024	###########	###########	###########
1345	0.12	########	########	€ 582,153	€ 776,203	-€ 4,692,092	-€ 9,384,184	###########	###########
1346	0.20	########	########	€ 923,007	#########	-€ 7,439,344	###########	###########	###########
1347	0.16	########	########	€ 747,535	€ 996,713	-€ 6,025,055	###########	###########	###########
1348	0.23	########	########	#########	#########	-€ 8,746,490	###########	###########	###########
1349	0.10	########	########	€ 463,344	€ 617,792	-€ 3,734,508	-€ 7,469,016	###########	###########
1350	0.16	########	########	€ 748,840	€ 998,454	-€ 6,035,579	###########	###########	###########
1351	0.19	########	########	€ 918,606	#########	-€ 7,403,872	###########	###########	###########
1352	0.20	########	########	€ 945,997	#########	-€ 7,624,644	###########	###########	###########
1353	0.16	########	########	€ 763,621	#########	-€ 6,154,711	###########	###########	###########
1354	0.15	########	########	€ 700,152	€ 933,536	-€ 5,643,156	###########	###########	###########
1355	0.17	########	########	€ 806,368	#########	-€ 6,499,245	###########	###########	###########
1356	0.14	########	########	€ 671,047	€ 894,729	-€ 5,408,573	###########	###########	###########
1357	0.14	########	########	€ 682,190	€ 909,587	-€ 5,498,386	###########	###########	###########
1358	0.16	########	########	€ 774,926	#########	-€ 6,245,829	###########	###########	###########
1359	0.18	########	########	€ 869,571	#########	-€ 7,008,656	###########	###########	###########
1360	0.18	########	########	€ 868,483	#########	-€ 6,999,889	###########	###########	###########
1361	0.14	########	########	€ 680,884	€ 907,845	-€ 5,487,858	###########	###########	###########
1362	0.23	########	########	#########	#########	-€ 8,736,528	###########	###########	###########
1363	0.15	########	########	€ 721,321	€ 961,761	-€ 5,813,776	###########	###########	###########
1364	0.20	########	########	€ 921,177	#########	-€ 7,424,593	###########	###########	###########
1365	0.19	########	########	€ 910,861	#########	-€ 7,341,452	###########	###########	###########
1366	0.17	########	########	€ 797,966	#########	-€ 6,431,530	###########	###########	###########
1367	0.16	########	########	€ 744,150	€ 992,200	-€ 5,997,775	###########	###########	###########
1368	0.22	########	########	#########	#########	-€ 8,257,930	###########	###########	###########
1369	0.13	########	########	€ 627,332	€ 836,442	-€ 5,056,231	###########	###########	###########
1370	0.18	########	########	€ 828,029	#########	-€ 6,673,835	###########	###########	###########
1371	0.13	########	########	€ 628,738	€ 838,318	-€ 5,067,570	###########	###########	###########
1372	0.24	########	########	#########	#########	-€ 9,227,932	###########	###########	###########

1373	0.20	########	########	€ 955,315	#########	-€ 7,699,744	###########	###########	###########
1374	0.14	########	########	€ 683,937	€ 911,916	-€ 5,512,463	###########	###########	###########
1375	0.11	########	########	€ 537,988	€ 717,317	-€ 4,336,127	-€ 8,672,254	###########	###########
1376	0.16	########	########	€ 748,306	€ 997,742	-€ 6,031,275	###########	###########	###########
1377	0.11	########	########	€ 519,379	€ 692,505	-€ 4,186,143	-€ 8,372,286	###########	###########
1378	0.12	########	########	€ 562,877	€ 750,502	-€ 4,536,732	-€ 9,073,463	###########	###########
1379	0.19	########	########	€ 908,775	#########	-€ 7,324,634	###########	###########	###########
1380	0.16	########	########	€ 742,789	€ 990,386	-€ 5,986,809	###########	###########	###########
1381	0.11	########	########	€ 500,467	€ 667,290	-€ 4,033,717	-€ 8,067,434	###########	###########
1382	0.08	########	########	€ 397,534	€ 530,045	-€ 3,204,083	-€ 6,408,166	-€ 9,612,249	###########
1383	0.25	########	########	#########	#########	-€ 9,348,341	###########	###########	###########
1384	0.09	########	########	€ 445,686	€ 594,248	-€ 3,592,186	-€ 7,184,372	###########	###########
1385	0.20	########	########	€ 923,347	#########	-€ 7,442,082	###########	###########	###########
1386	0.19	########	########	€ 896,832	#########	-€ 7,228,373	###########	###########	###########
1387	0.16	########	########	€ 756,223	#########	-€ 6,095,080	###########	###########	###########
1388	0.15	########	########	€ 723,068	€ 964,091	-€ 5,827,856	###########	###########	###########
1389	0.16	########	########	€ 746,983	€ 995,977	-€ 6,020,609	###########	###########	###########
1390	0.16	########	########	€ 762,385	#########	-€ 6,144,749	###########	###########	###########
1391	0.15	########	########	€ 691,026	€ 921,367	-€ 5,569,598	###########	###########	###########
1392	0.12	########	########	€ 574,194	€ 765,593	-€ 4,627,951	-€ 9,255,901	###########	###########
1393	0.14	########	########	€ 643,483	€ 857,978	-€ 5,186,411	###########	###########	###########
1394	0.14	########	########	€ 651,886	€ 869,181	-€ 5,254,137	###########	###########	###########
1395	0.11	########	########	€ 536,263	€ 715,017	-€ 4,322,224	-€ 8,644,447	###########	###########
1396	0.19	########	########	€ 885,643	#########	-€ 7,138,197	###########	###########	###########
1397	0.14	########	########	€ 676,998	€ 902,663	-€ 5,456,533	###########	###########	###########
1398	0.17	########	########	€ 800,267	#########	-€ 6,450,077	###########	###########	###########
1399	0.19	########	########	€ 888,071	#########	-€ 7,157,767	###########	###########	###########
1400	0.13	########	########	€ 631,651	€ 842,201	-€ 5,091,043	###########	###########	###########
1401	0.17	########	########	€ 815,991	#########	-€ 6,576,809	###########	###########	###########
1402	0.18	########	########	€ 843,288	#########	-€ 6,796,820	###########	###########	###########
1403	0.10	########	########	€ 454,298	€ 605,731	-€ 3,661,598	-€ 7,323,196	###########	###########
1404	0.18	########	########	€ 871,996	#########	-€ 7,028,205	###########	###########	###########
1405	0.15	########	########	€ 728,583	€ 971,444	-€ 5,872,309	###########	###########	###########
1406	0.15	########	########	€ 715,597	€ 954,129	-€ 5,767,638	###########	###########	###########
1407	0.12	########	########	€ 589,765	€ 786,353	-€ 4,753,445	-€ 9,506,889	###########	###########
1408	0.19	########	########	€ 905,041	#########	-€ 7,294,538	###########	###########	###########
1409	0.15	########	########	€ 707,497	€ 943,330	-€ 5,702,359	###########	###########	###########
1410	0.14	########	########	€ 644,927	€ 859,903	-€ 5,198,050	###########	###########	###########
1411	0.19	########	########	€ 882,725	#########	-€ 7,114,680	###########	###########	###########
1412	0.12	########	########	€ 583,375	€ 777,833	-€ 4,701,943	-€ 9,403,886	###########	###########
1413	0.10	########	########	€ 485,769	€ 647,691	-€ 3,915,247	-€ 7,830,493	###########	###########
1414	0.23	########	########	#########	#########	-€ 8,773,875	###########	###########	###########
1415	0.13	########	########	€ 621,320	€ 828,427	-€ 5,007,779	###########	###########	###########
1416	0.17	########	########	€ 781,953	#########	-€ 6,302,462	###########	###########	###########
1417	0.15	########	########	€ 717,066	€ 956,088	-€ 5,779,483	###########	###########	###########
1418	0.15	########	########	€ 716,594	€ 955,459	-€ 5,775,680	###########	###########	###########
1419	0.20	########	########	€ 922,112	#########	-€ 7,432,135	###########	###########	###########
1420	0.14	########	########	€ 667,951	€ 890,601	-€ 5,383,617	###########	###########	###########
1421	0.18	########	########	€ 836,086	#########	-€ 6,738,773	###########	###########	###########
1422	0.15	########	########	€ 704,253	€ 939,005	-€ 5,676,213	###########	###########	###########
1423	0.15	########	########	€ 697,455	€ 929,940	-€ 5,621,420	###########	###########	###########
1424	0.16	########	########	€ 770,921	#########	-€ 6,213,548	###########	###########	###########
1425	0.08	########	########	€ 378,457	€ 504,610	-€ 3,050,327	-€ 6,100,654	-€ 9,150,981	###########
1426	0.21	########	########	€ 976,469	#########	-€ 7,870,242	###########	###########	###########
1427	0.10	########	########	€ 460,539	€ 614,052	-€ 3,711,898	-€ 7,423,797	###########	###########
1428	0.17	########	########	€ 807,082	#########	-€ 6,504,999	###########	###########	###########
1429	0.18	########	########	€ 843,513	#########	-€ 6,798,634	###########	###########	###########
1430	0.18	########	########	€ 844,624	#########	-€ 6,807,589	###########	###########	###########
1431	0.18	########	########	€ 841,771	#########	-€ 6,784,587	###########	###########	###########
1432	0.24	########	########	#########	#########	-€ 9,100,887	###########	###########	###########
1433	0.12	########	########	€ 551,229	€ 734,972	-€ 4,442,851	-€ 8,885,702	###########	###########
1434	0.19	########	########	€ 895,307	#########	-€ 7,216,086	###########	###########	###########
1435	0.24	########	########	#########	#########	-€ 9,232,914	###########	###########	###########
1436	0.20	########	########	€ 922,944	#########	-€ 7,438,841	###########	###########	###########
1437	0.22	########	########	#########	#########	-€ 8,258,420	###########	###########	###########
1438	0.16	########	########	€ 768,507	#########	-€ 6,194,093	###########	###########	###########
1439	0.19	########	########	€ 876,174	#########	-€ 7,061,873	###########	###########	###########
1440	0.12	########	########	€ 572,264	€ 763,018	-€ 4,612,389	-€ 9,224,779	###########	###########
1441	0.11	########	########	€ 522,825	€ 697,100	-€ 4,213,916	-€ 8,427,833	###########	###########
1442	0.17	########	########	€ 800,028	#########	-€ 6,448,143	###########	###########	###########
1443	0.15	########	########	€ 702,345	€ 936,460	-€ 5,660,831	###########	###########	###########
1444	0.18	########	########	€ 836,046	#########	-€ 6,738,450	###########	###########	###########
1445	0.15	########	########	€ 707,677	€ 943,569	-€ 5,703,803	###########	###########	###########
1446	0.16	########	########	€ 775,063	#########	-€ 6,246,930	###########	###########	###########
1447	0.20	########	########	€ 966,844	#########	-€ 7,792,670	###########	###########	###########
1448	0.12	########	########	€ 550,495	€ 733,993	-€ 4,436,933	-€ 8,873,866	###########	###########
1449	0.14	########	########	€ 667,557	€ 890,077	-€ 5,380,447	###########	###########	###########
1450	0.16	########	########	€ 739,235	€ 985,646	-€ 5,958,160	###########	###########	###########
1451	0.19	########	########	€ 878,874	#########	-€ 7,083,638	###########	###########	###########
1452	0.14	########	########	€ 676,241	€ 901,654	-€ 5,450,432	###########	###########	###########
1453	0.13	########	########	€ 626,357	€ 835,143	-€ 5,048,378	###########	###########	###########
1454	0.20	########	########	€ 952,809	#########	-€ 7,679,547	###########	###########	###########
1455	0.08	########	########	€ 398,779	€ 531,706	-€ 3,214,123	-€ 6,428,246	-€ 9,642,368	###########
1456	0.16	########	########	€ 748,563	€ 998,084	-€ 6,033,341	###########	###########	###########
1457	0.14	########	########	€ 681,096	€ 908,128	-€ 5,489,567	###########	###########	###########
1458	0.19	########	########	€ 900,205	#########	-€ 7,255,567	###########	###########	###########
1459	0.16	########	########	€ 760,373	#########	-€ 6,128,527	###########	###########	###########
1460	0.15	########	########	€ 701,590	€ 935,453	-€ 5,654,742	###########	###########	###########
1461	0.13	########	########	€ 593,582	€ 791,443	-€ 4,784,214	-€ 9,568,427	###########	###########
1462	0.15	########	########	€ 711,399	€ 948,532	-€ 5,733,807	###########	###########	###########
1463	0.19	########	########	€ 901,682	#########	-€ 7,267,469	###########	###########	###########
1464	0.17	########	########	€ 782,145	#########	-€ 6,304,015	###########	###########	###########
1465	0.17	########	########	€ 803,090	#########	-€ 6,472,824	###########	###########	###########
1466	0.14	########	########	€ 655,790	€ 874,387	-€ 5,285,603	###########	###########	###########
1467	0.16	########	########	€ 738,806	€ 985,074	-€ 5,954,700	###########	###########	###########
1468	0.20	########	########	€ 927,767	#########	-€ 7,477,707	###########	###########	###########
1469	0.19	########	########	€ 896,101	#########	-€ 7,222,489	###########	###########	###########
1470	0.22	########	########	#########	#########	-€ 8,545,392	###########	###########	###########
1471	0.19	########	########	€ 895,123	#########	-€ 7,214,602	###########	###########	###########
1472	0.15	########	########	€ 728,809	€ 971,745	-€ 5,874,127	###########	###########	###########
1473	0.16	########	########	€ 767,077	#########	-€ 6,182,562	###########	###########	###########
1474	0.15	########	########	€ 692,837	€ 923,782	-€ 5,584,196	###########	###########	###########
1475	0.12	########	########	€ 588,976	€ 785,301	-€ 4,747,086	-€ 9,494,171	###########	###########
1476	0.21	########	########	#########	#########	-€ 8,124,657	###########	###########	###########
1477	0.19	########	########	€ 905,575	#########	-€ 7,298,842	###########	###########	###########
1478	0.18	########	########	€ 833,579	#########	-€ 6,718,568	###########	###########	###########
1479	0.20	########	########	€ 947,252	#########	-€ 7,634,754	###########	###########	###########
1480	0.09	########	########	€ 442,239	€ 589,652	-€ 3,564,400	-€ 7,128,801	###########	###########
1481	0.16	########	########	€ 756,684	#########	-€ 6,098,800	###########	###########	###########
1482	0.17	########	########	€ 787,161	#########	-€ 6,344,436	###########	###########	###########
1483	0.16	########	########	€ 762,957	#########	-€ 6,149,357	###########	###########	###########
1484	0.20	########	########	€ 924,790	#########	-€ 7,453,714	###########	###########	###########
1485	0.17	########	########	€ 787,219	#########	-€ 6,344,905	###########	###########	###########
1486	0.10	########	########	€ 475,271	€ 633,694	-€ 3,830,636	-€ 7,661,272	###########	###########
1487	0.12	########	########	€ 550,850	€ 734,466	-€ 4,439,795	-€ 8,879,590	###########	###########
1488	0.17	########	########	€ 810,977	#########	-€ 6,536,391	###########	###########	###########
1489	0.18	########	########	€ 831,055	#########	-€ 6,698,221	###########	###########	###########
1490	0.15	########	########	€ 686,446	€ 915,261	-€ 5,532,687	###########	###########	###########
1491	0.21	########	########	€ 993,497	#########	-€ 8,007,487	###########	###########	###########
1492	0.15	########	########	€ 713,546	€ 951,394	-€ 5,751,109	###########	###########	###########
1493	0.17	########	########	€ 808,166	#########	-€ 6,513,740	###########	###########	###########
1494	0.13	########	########	€ 626,886	€ 835,848	-€ 5,052,642	###########	###########	###########
1495	0.17	########	########	€ 792,681	#########	-€ 6,388,933	###########	###########	###########
1496	0.16	########	########	€ 757,506	#########	-€ 6,105,424	###########	###########	###########
1497	0.06	########	########	€ 306,159	€ 408,212	-€ 2,467,614	-€ 4,935,227	-€ 7,402,841	-€ 9,870,454
1498	0.13	########	########	€ 615,396	€ 820,528	-€ 4,960,028	-€ 9,920,056	###########	###########
1499	0.12	########	########	€ 561,967	€ 749,290	-€ 4,529,400	-€ 9,058,801	###########	###########
1500	0.15	########	########	€ 690,381	€ 920,508	-€ 5,564,405	###########	###########	###########
1501	0.11	########	########	€ 534,685	€ 712,913	-€ 4,309,505	-€ 8,619,011	###########	###########
1502	0.19	########	########	€ 908,066	#########	-€ 7,318,921	###########	###########	###########
1503	0.21	########	########	#########	#########	-€ 8,157,554	###########	###########	###########
1504	0.13	########	########	€ 594,654	€ 792,872	-€ 4,792,855	-€ 9,585,710	###########	###########
1505	0.25	########	########	#########	#########	-€ 9,600,749	###########	###########	###########
1506	0.11	########	########	€ 514,792	€ 686,389	-€ 4,149,171	-€ 8,298,341	###########	###########
1507	0.14	########	########	€ 669,159	€ 892,211	-€ 5,393,352	###########	###########	###########
1508	0.12	########	########	€ 554,030	€ 738,707	-€ 4,465,429	-€ 8,930,857	###########	###########
1509	0.14	########	########	€ 681,476	€ 908,635	-€ 5,492,632	###########	###########	###########
1510	0.19	########	########	€ 886,779	#########	-€ 7,147,352	###########	###########	###########
1511	0.15	########	########	€ 706,706	€ 942,274	-€ 5,695,979	###########	###########	###########
1512	0.18	########	########	€ 838,746	#########	-€ 6,760,210	###########	###########	###########
1513	0.19	########	########	€ 889,414	#########	-€ 7,168,586	###########	###########	###########
1514	0.14	########	########	€ 638,675	€ 851,566	-€ 5,147,654	###########	###########	###########
1515	0.11	########	########	€ 508,439	€ 677,918	-€ 4,097,965	-€ 8,195,929	###########	###########
1516	0.14	########	########	€ 662,411	€ 883,215	-€ 5,338,967	###########	###########	###########
1517	0.20	########	########	€ 963,762	#########	-€ 7,767,826	###########	###########	###########
1518	0.16	########	########	€ 769,281	#########	-€ 6,200,329	###########	###########	###########
1519	0.20	########	########	€ 924,773	#########	-€ 7,453,582	###########	###########	###########
1520	0.19	########	########	€ 900,323	#########	-€ 7,256,513	###########	###########	###########
1521	0.13	########	########	€ 627,948	€ 837,264	-€ 5,061,200	###########	###########	###########
1522	0.16	########	########	€ 745,842	€ 994,456	-€ 6,011,415	###########	###########	###########
1523	0.16	########	########	€ 762,801	#########	-€ 6,148,098	###########	###########	###########
1524	0.16	########	########	€ 772,629	#########	-€ 6,227,314	###########	###########	###########
1525	0.15	########	########	€ 691,851	€ 922,468	-€ 5,576,252	###########	###########	###########
1526	0.15	########	########	€ 712,976	€ 950,635	-€ 5,746,516	###########	###########	###########
1527	0.18	########	########	€ 846,987	#########	-€ 6,826,633	###########	###########	###########
1528	0.14	########	########	€ 650,360	€ 867,147	-€ 5,241,840	###########	###########	###########
1529	0.14	########	########	€ 644,250	€ 859,000	-€ 5,192,591	###########	###########	###########
1530	0.11	########	########	€ 508,660	€ 678,213	-€ 4,099,749	-€ 8,199,498	###########	###########
1531	0.10	########	########	€ 456,169	€ 608,225	-€ 3,676,677	-€ 7,353,354	###########	###########
1532	0.17	########	########	€ 792,978	#########	-€ 6,391,327	###########	###########	###########
1533	0.19	########	########	€ 920,464	#########	-€ 7,418,847	###########	###########	###########
1534	0.17	########	########	€ 791,978	#########	-€ 6,383,268	###########	###########	###########
1535	0.15	########	########	€ 688,367	€ 917,822	-€ 5,548,167	###########	###########	###########
1536	0.14	########	########	€ 649,986	€ 866,647	-€ 5,238,819	###########	###########	###########
1537	0.24	########	########	#########	#########	-€ 9,320,605	###########	###########	###########
1538	0.16	########	########	€ 759,346	#########	-€ 6,120,257	###########	###########	###########
1539	0.17	########	########	€ 818,738	#########	-€ 6,598,944	###########	###########	###########
1540	0.19	########	########	€ 912,094	#########	-€ 7,351,390	###########	###########	###########
1541	0.21	########	########	#########	#########	-€ 8,140,461	###########	###########	###########
1542	0.17	########	########	€ 795,819	#########	-€ 6,414,221	###########	###########	###########
1543	0.12	########	########	€ 549,688	€ 732,917	-€ 4,430,429	-€ 8,860,858	###########	###########
1544	0.22	########	########	#########	#########	-€ 8,529,805	###########	###########	###########

1545	0.20	########	########	€ 951,541	#########	-€ 7,669,328	###########	###########	###########
1546	0.12	########	########	€ 577,101	€ 769,468	-€ 4,651,375	-€ 9,302,751	###########	###########
1547	0.19	########	########	€ 918,130	#########	-€ 7,400,035	###########	###########	###########
1548	0.13	########	########	€ 622,918	€ 830,557	-€ 5,020,655	###########	###########	###########
1549	0.11	########	########	€ 540,967	€ 721,289	-€ 4,360,137	-€ 8,720,275	###########	###########
1550	0.14	########	########	€ 675,859	€ 901,145	-€ 5,447,355	###########	###########	###########
1551	0.19	########	########	€ 884,719	#########	-€ 7,130,745	###########	###########	###########
1552	0.17	########	########	€ 792,142	#########	-€ 6,384,583	###########	###########	###########
1553	0.16	########	########	€ 746,623	€ 995,498	-€ 6,017,712	###########	###########	###########
1554	0.12	########	########	€ 588,863	€ 785,151	-€ 4,746,180	-€ 9,492,360	###########	###########
1555	0.12	########	########	€ 577,483	€ 769,977	-€ 4,654,452	-€ 9,308,904	###########	###########
1556	0.16	########	########	€ 741,796	€ 989,061	-€ 5,978,802	###########	###########	###########
1557	0.13	########	########	€ 630,829	€ 841,105	-€ 5,084,416	###########	###########	###########
1558	0.15	########	########	€ 707,371	€ 943,161	-€ 5,701,337	###########	###########	###########
1559	0.19	########	########	€ 874,309	#########	-€ 7,046,841	###########	###########	###########
1560	0.11	########	########	€ 506,553	€ 675,404	-€ 4,082,770	-€ 8,165,540	###########	###########
1561	0.12	########	########	€ 578,827	€ 771,769	-€ 4,665,289	-€ 9,330,577	###########	###########
1562	0.21	########	########	€ 971,382	#########	-€ 7,829,243	###########	###########	###########
1563	0.14	########	########	€ 644,096	€ 858,794	-€ 5,191,346	###########	###########	###########
1564	0.13	########	########	€ 635,961	€ 847,948	-€ 5,125,781	###########	###########	###########
1565	0.11	########	########	€ 539,521	€ 719,362	-€ 4,348,488	-€ 8,696,976	###########	###########
1566	0.17	########	########	€ 812,932	#########	-€ 6,552,148	###########	###########	###########
1567	0.13	########	########	€ 591,108	€ 788,145	-€ 4,764,275	-€ 9,528,551	###########	###########
1568	0.13	########	########	€ 610,288	€ 813,717	-€ 4,918,858	-€ 9,837,717	###########	###########
1569	0.14	########	########	€ 646,382	€ 861,842	-€ 5,209,771	###########	###########	###########
1570	0.16	########	########	€ 751,604	#########	-€ 6,057,855	###########	###########	###########
1571	0.19	########	########	€ 917,542	#########	-€ 7,395,301	###########	###########	###########
1572	0.12	########	########	€ 557,040	€ 742,720	-€ 4,489,685	-€ 8,979,370	###########	###########
1573	0.15	########	########	€ 687,125	€ 916,167	-€ 5,538,161	###########	###########	###########
1574	0.17	########	########	€ 790,307	#########	-€ 6,369,798	###########	###########	###########
1575	0.14	########	########	€ 662,947	€ 883,930	-€ 5,343,291	###########	###########	###########
1576	0.23	########	########	#########	#########	-€ 8,796,581	###########	###########	###########
1577	0.17	########	########	€ 806,326	#########	-€ 6,498,906	###########	###########	###########
1578	0.17	########	########	€ 805,172	#########	-€ 6,489,606	###########	###########	###########
1579	0.19	########	########	€ 894,376	#########	-€ 7,208,579	###########	###########	###########
1580	0.20	########	########	€ 964,694	#########	-€ 7,775,338	###########	###########	###########
1581	0.12	########	########	€ 579,647	€ 772,863	-€ 4,671,898	-€ 9,343,797	###########	###########
1582	0.17	########	########	€ 812,277	#########	-€ 6,546,871	###########	###########	###########
1583	0.23	########	########	#########	#########	-€ 8,767,302	###########	###########	###########
1584	0.20	########	########	€ 954,178	#########	-€ 7,690,582	###########	###########	###########
1585	0.08	########	########	€ 393,439	€ 524,585	-€ 3,171,078	-€ 6,342,156	-€ 9,513,234	###########
1586	0.12	########	########	€ 561,771	€ 749,029	-€ 4,527,822	-€ 9,055,645	###########	###########
1587	0.21	########	########	€ 995,343	#########	-€ 8,022,367	###########	###########	###########
1588	0.11	########	########	€ 532,096	€ 709,462	-€ 4,288,644	-€ 8,577,288	###########	###########
1589	0.14	########	########	€ 645,256	€ 860,341	-€ 5,200,698	###########	###########	###########
1590	0.16	########	########	€ 742,441	€ 989,921	-€ 5,983,998	###########	###########	###########
1591	0.21	########	########	#########	#########	-€ 8,150,806	###########	###########	###########
1592	0.15	########	########	€ 703,865	€ 938,486	-€ 5,673,079	###########	###########	###########
1593	0.14	########	########	€ 652,729	€ 870,306	-€ 5,260,934	###########	###########	###########
1594	0.19	########	########	€ 913,459	#########	-€ 7,362,386	###########	###########	###########
1595	0.18	########	########	€ 858,922	#########	-€ 6,922,825	###########	###########	###########
1596	0.16	########	########	€ 743,856	€ 991,808	-€ 5,995,406	###########	###########	###########
1597	0.13	########	########	€ 632,901	€ 843,868	-€ 5,101,118	###########	###########	###########
1598	0.15	########	########	€ 697,757	€ 930,343	-€ 5,623,856	###########	###########	###########
1599	0.17	########	########	€ 814,160	#########	-€ 6,562,046	###########	###########	###########
1600	0.14	########	########	€ 643,592	€ 858,122	-€ 5,187,286	###########	###########	###########
1601	0.17	########	########	€ 825,215	#########	-€ 6,651,150	###########	###########	###########
1602	0.12	########	########	€ 547,029	€ 729,372	-€ 4,409,002	-€ 8,818,004	###########	###########
1603	0.23	########	########	#########	#########	-€ 8,623,299	###########	###########	###########
1604	0.16	########	########	€ 741,777	€ 989,036	-€ 5,978,648	###########	###########	###########
1605	0.20	########	########	€ 945,173	#########	-€ 7,618,002	###########	###########	###########
1606	0.13	########	########	€ 599,728	€ 799,638	-€ 4,833,751	-€ 9,667,503	###########	###########
1607	0.15	########	########	€ 719,944	€ 959,926	-€ 5,802,679	###########	###########	###########
1608	0.21	########	########	€ 998,057	#########	-€ 8,044,241	###########	###########	###########
1609	0.14	########	########	€ 684,191	€ 912,255	-€ 5,514,513	###########	###########	###########
1610	0.17	########	########	€ 821,419	#########	-€ 6,620,553	###########	###########	###########
1611	0.09	########	########	€ 411,794	€ 549,059	-€ 3,319,021	-€ 6,638,041	-€ 9,957,062	###########
1612	0.14	########	########	€ 675,210	€ 900,280	-€ 5,442,123	###########	###########	###########
1613	0.25	########	########	#########	#########	-€ 9,663,089	###########	###########	###########
1614	0.21	########	########	€ 971,796	#########	-€ 7,832,579	###########	###########	###########
1615	0.19	########	########	€ 898,027	#########	-€ 7,238,006	###########	###########	###########
1616	0.23	########	########	#########	#########	-€ 8,593,324	###########	###########	###########
1617	0.17	########	########	€ 795,655	#########	-€ 6,412,898	###########	###########	###########
1618	0.14	########	########	€ 641,208	€ 854,944	-€ 5,168,070	###########	###########	###########
1619	0.15	########	########	€ 698,986	€ 931,981	-€ 5,633,758	###########	###########	###########
1620	0.14	########	########	€ 660,409	€ 880,545	-€ 5,322,827	###########	###########	###########
1621	0.15	########	########	€ 687,399	€ 916,532	-€ 5,540,370	###########	###########	###########
1622	0.10	########	########	€ 492,657	€ 656,875	-€ 3,970,764	-€ 7,941,527	###########	###########
1623	0.13	########	########	€ 600,228	€ 800,304	-€ 4,837,775	-€ 9,675,551	###########	###########
1624	0.18	########	########	€ 843,666	#########	-€ 6,799,862	###########	###########	###########
1625	0.16	########	########	€ 777,557	#########	-€ 6,267,033	###########	###########	###########
1626	0.18	########	########	€ 860,416	#########	-€ 6,934,870	###########	###########	###########
1627	0.22	########	########	#########	#########	-€ 8,457,869	###########	###########	###########
1628	0.16	########	########	€ 772,771	#########	-€ 6,228,457	###########	###########	###########
1629	0.12	########	########	€ 586,545	€ 782,060	-€ 4,727,497	-€ 9,454,994	###########	###########
1630	0.14	########	########	€ 668,457	€ 891,276	-€ 5,387,700	###########	###########	###########
1631	0.17	########	########	€ 815,140	#########	-€ 6,569,951	###########	###########	###########
1632	0.12	########	########	€ 565,077	€ 753,436	-€ 4,554,467	-€ 9,108,933	###########	###########
1633	0.20	########	########	€ 957,752	#########	-€ 7,719,388	###########	###########	###########
1634	0.14	########	########	€ 650,689	€ 867,585	-€ 5,244,486	###########	###########	###########
1635	0.16	########	########	€ 736,111	€ 981,481	-€ 5,932,981	###########	###########	###########
1636	0.17	########	########	€ 786,687	#########	-€ 6,340,618	###########	###########	###########
1637	0.14	########	########	€ 658,820	€ 878,427	-€ 5,310,025	###########	###########	###########
1638	0.17	########	########	€ 790,116	#########	-€ 6,368,259	###########	###########	###########
1639	0.13	########	########	€ 633,261	€ 844,347	-€ 5,104,017	###########	###########	###########
1640	0.14	########	########	€ 678,483	€ 904,644	-€ 5,468,503	###########	###########	###########
1641	0.10	########	########	€ 495,717	€ 660,956	-€ 3,995,432	-€ 7,990,864	###########	###########
1642	0.14	########	########	€ 672,870	€ 897,160	-€ 5,423,263	###########	###########	###########
1643	0.16	########	########	€ 753,269	#########	-€ 6,071,277	###########	###########	###########
1644	0.13	########	########	€ 631,605	€ 842,140	-€ 5,090,672	###########	###########	###########
1645	0.20	########	########	€ 961,706	#########	-€ 7,751,254	###########	###########	###########
1646	0.13	########	########	€ 599,669	€ 799,558	-€ 4,833,271	-€ 9,666,543	###########	###########
1647	0.19	########	########	€ 909,528	#########	-€ 7,330,709	###########	###########	###########
1648	0.16	########	########	€ 771,947	#########	-€ 6,221,814	###########	###########	###########
1649	0.19	########	########	€ 919,363	#########	-€ 7,409,977	###########	###########	###########
1650	0.15	########	########	€ 703,956	€ 938,609	-€ 5,673,820	###########	###########	###########
1651	0.12	########	########	€ 572,336	€ 763,115	-€ 4,612,975	-€ 9,225,951	###########	###########
1652	0.09	########	########	€ 415,830	€ 554,440	-€ 3,351,546	-€ 6,703,092	###########	###########
1653	0.16	########	########	€ 762,081	#########	-€ 6,142,298	###########	###########	###########
1654	0.16	########	########	€ 762,840	#########	-€ 6,148,414	###########	###########	###########
1655	0.17	########	########	€ 789,915	#########	-€ 6,366,641	###########	###########	###########
1656	0.15	########	########	€ 730,534	€ 974,045	-€ 5,888,030	###########	###########	###########
1657	0.15	########	########	€ 691,210	€ 921,613	-€ 5,571,084	###########	###########	###########
1658	0.21	########	########	€ 971,108	#########	-€ 7,827,036	###########	###########	###########
1659	0.17	########	########	€ 791,277	#########	-€ 6,377,615	###########	###########	###########
1660	0.21	########	########	€ 973,615	#########	-€ 7,847,238	###########	###########	###########
1661	0.27	########	########	#########	#########	###########	###########	###########	###########
1662	0.14	########	########	€ 659,531	€ 879,375	-€ 5,315,756	###########	###########	###########
1663	0.14	########	########	€ 672,116	€ 896,155	-€ 5,417,191	###########	###########	###########
1664	0.17	########	########	€ 783,525	#########	-€ 6,315,133	###########	###########	###########
1665	0.13	########	########	€ 630,903	€ 841,204	-€ 5,085,014	###########	###########	###########
1666	0.17	########	########	€ 807,845	#########	-€ 6,511,154	###########	###########	###########
1667	0.13	########	########	€ 606,331	€ 808,442	-€ 4,886,969	-€ 9,773,939	###########	###########
1668	0.13	########	########	€ 604,406	€ 805,874	-€ 4,871,451	-€ 9,742,901	###########	###########
1669	0.10	########	########	€ 468,187	€ 624,250	-€ 3,773,543	-€ 7,547,086	###########	###########
1670	0.16	########	########	€ 756,967	#########	-€ 6,101,077	###########	###########	###########
1671	0.12	########	########	€ 588,806	€ 785,075	-€ 4,745,721	-€ 9,491,442	###########	###########
1672	0.16	########	########	€ 773,027	#########	-€ 6,230,525	###########	###########	###########
1673	0.18	########	########	€ 840,808	#########	-€ 6,776,828	###########	###########	###########
1674	0.15	########	########	€ 708,970	€ 945,294	-€ 5,714,230	###########	###########	###########
1675	0.12	########	########	€ 586,408	€ 781,877	-€ 4,726,391	-€ 9,452,782	###########	###########
1676	0.22	########	########	#########	#########	-€ 8,288,009	###########	###########	###########
1677	0.14	########	########	€ 665,675	€ 887,566	-€ 5,365,274	###########	###########	###########
1678	0.15	########	########	€ 711,544	€ 948,726	-€ 5,734,978	###########	###########	###########
1679	0.15	########	########	€ 701,785	€ 935,713	-€ 5,656,318	###########	###########	###########
1680	0.14	########	########	€ 668,779	€ 891,706	-€ 5,390,294	###########	###########	###########
1681	0.15	########	########	€ 687,549	€ 916,732	-€ 5,541,579	###########	###########	###########
1682	0.12	########	########	€ 574,508	€ 766,011	-€ 4,630,477	-€ 9,260,954	###########	###########
1683	0.19	########	########	€ 889,210	#########	-€ 7,166,948	###########	###########	###########
1684	0.10	########	########	€ 494,960	€ 659,947	-€ 3,989,329	-€ 7,978,658	###########	###########
1685	0.22	########	########	#########	#########	-€ 8,251,529	###########	###########	###########
1686	0.16	########	########	€ 747,888	€ 997,184	-€ 6,027,901	###########	###########	###########
1687	0.12	########	########	€ 573,618	€ 764,824	-€ 4,623,302	-€ 9,246,603	###########	###########
1688	0.16	########	########	€ 764,986	#########	-€ 6,165,710	###########	###########	###########
1689	0.21	########	########	€ 983,806	#########	-€ 7,929,381	###########	###########	###########
1690	0.15	########	########	€ 701,312	€ 935,083	-€ 5,652,509	###########	###########	###########
1691	0.17	########	########	€ 812,873	#########	-€ 6,551,679	###########	###########	###########
1692	0.19	########	########	€ 893,240	#########	-€ 7,199,425	###########	###########	###########
1693	0.19	########	########	€ 900,321	#########	-€ 7,256,497	###########	###########	###########
1694	0.11	########	########	€ 522,839	€ 697,118	-€ 4,214,027	-€ 8,428,054	###########	###########
1695	0.13	########	########	€ 596,779	€ 795,705	-€ 4,809,978	-€ 9,619,957	###########	###########
1696	0.19	########	########	€ 875,727	#########	-€ 7,058,271	###########	###########	###########
1697	0.10	########	########	€ 485,494	€ 647,325	-€ 3,913,035	-€ 7,826,069	###########	###########
1698	0.15	########	########	€ 709,086	€ 945,448	-€ 5,715,161	###########	###########	###########
1699	0.14	########	########	€ 682,012	€ 909,350	-€ 5,496,951	###########	###########	###########
1700	0.18	########	########	€ 851,368	#########	-€ 6,861,945	###########	###########	###########
1701	0.13	########	########	€ 624,966	€ 833,289	-€ 5,037,168	###########	###########	###########
1702	0.14	########	########	€ 651,783	€ 869,044	-€ 5,253,309	###########	###########	###########
1703	0.10	########	########	€ 490,586	€ 654,114	-€ 3,954,072	-€ 7,908,145	###########	###########
1704	0.18	########	########	€ 837,245	#########	-€ 6,748,108	###########	###########	###########
1705	0.22	########	########	#########	#########	-€ 8,306,597	###########	###########	###########
1706	0.13	########	########	€ 607,753	€ 810,337	-€ 4,898,426	-€ 9,796,852	###########	###########
1707	0.11	########	########	€ 516,167	€ 688,223	-€ 4,160,256	-€ 8,320,511	###########	###########
1708	0.10	########	########	€ 493,094	€ 657,459	-€ 3,974,292	-€ 7,948,584	###########	###########
1709	0.12	########	########	€ 548,073	€ 730,764	-€ 4,417,413	-€ 8,834,827	###########	###########
1710	0.14	########	########	€ 663,257	€ 884,343	-€ 5,345,789	###########	###########	###########
1711	0.20	########	########	€ 943,843	#########	-€ 7,607,284	###########	###########	###########
1712	0.16	########	########	€ 774,460	#########	-€ 6,242,074	###########	###########	###########
1713	0.17	########	########	€ 824,980	#########	-€ 6,649,257	###########	###########	###########
1714	0.21	########	########	€ 987,041	#########	-€ 7,955,451	###########	###########	###########
1715	0.16	########	########	€ 748,381	€ 997,841	-€ 6,031,878	###########	###########	###########
1716	0.19	########	########	€ 888,496	#########	-€ 7,161,193	###########	###########	###########

1717	0.13	########	########	€ 609,233	€ 812,310	-€ 4,910,355	-€ 9,820,710	###########	###########
1718	0.18	########	########	€ 849,659	#########	-€ 6,848,164	###########	###########	###########
1719	0.14	########	########	€ 673,931	€ 898,575	-€ 5,431,817	###########	###########	###########
1720	0.16	########	########	€ 768,496	#########	-€ 6,194,003	###########	###########	###########
1721	0.11	########	########	€ 518,193	€ 690,924	-€ 4,176,582	-€ 8,353,165	###########	###########
1722	0.17	########	########	€ 789,875	#########	-€ 6,366,314	###########	###########	###########
1723	0.12	########	########	€ 564,871	€ 753,161	-€ 4,552,801	-€ 9,105,602	###########	###########
1724	0.22	########	########	#########	#########	-€ 8,442,956	###########	###########	###########
1725	0.18	########	########	€ 837,080	#########	-€ 6,746,785	###########	###########	###########
1726	0.12	########	########	€ 579,048	€ 772,064	-€ 4,667,070	-€ 9,334,140	###########	###########
1727	0.24	########	########	#########	#########	-€ 9,273,579	###########	###########	###########
1728	0.12	########	########	€ 544,110	€ 725,480	-€ 4,385,476	-€ 8,770,951	###########	###########
1729	0.20	########	########	€ 936,061	#########	-€ 7,544,561	###########	###########	###########
1730	0.24	########	########	#########	#########	-€ 8,981,181	###########	###########	###########
1731	0.12	########	########	€ 582,240	€ 776,320	-€ 4,692,798	-€ 9,385,596	###########	###########
1732	0.17	########	########	€ 806,527	#########	-€ 6,500,529	###########	###########	###########
1733	0.18	########	########	€ 855,944	#########	-€ 6,898,826	###########	###########	###########
1734	0.12	########	########	€ 573,570	€ 764,760	-€ 4,622,917	-€ 9,245,834	###########	###########
1735	0.12	########	########	€ 585,006	€ 780,008	-€ 4,715,091	-€ 9,430,182	###########	###########
1736	0.23	########	########	#########	#########	-€ 8,903,561	###########	###########	###########
1737	0.15	########	########	€ 723,390	€ 964,521	-€ 5,830,455	###########	###########	###########
1738	0.11	########	########	€ 512,566	€ 683,421	-€ 4,131,230	-€ 8,262,459	###########	###########
1739	0.13	########	########	€ 625,542	€ 834,056	-€ 5,041,808	###########	###########	###########
1740	0.13	########	########	€ 606,887	€ 809,182	-€ 4,891,446	-€ 9,782,892	###########	###########
1741	0.17	########	########	€ 813,704	#########	-€ 6,558,370	###########	###########	###########
1742	0.20	########	########	€ 947,066	#########	-€ 7,633,256	###########	###########	###########
1743	0.11	########	########	€ 519,087	€ 692,116	-€ 4,183,788	-€ 8,367,576	###########	###########
1744	0.12	########	########	€ 584,075	€ 778,767	-€ 4,707,586	-€ 9,415,172	###########	###########
1745	0.17	########	########	€ 795,226	#########	-€ 6,409,443	###########	###########	###########
1746	0.20	########	########	€ 930,143	#########	-€ 7,496,858	###########	###########	###########
1747	0.16	########	########	€ 741,809	€ 989,078	-€ 5,978,906	###########	###########	###########
1748	0.13	########	########	€ 636,069	€ 848,092	-€ 5,126,652	###########	###########	###########
1749	0.20	########	########	€ 930,461	#########	-€ 7,499,423	###########	###########	###########
1750	0.18	########	########	€ 834,405	#########	-€ 6,725,220	###########	###########	###########
1751	0.17	########	########	€ 816,351	#########	-€ 6,579,708	###########	###########	###########
1752	0.12	########	########	€ 580,751	€ 774,334	-€ 4,680,793	-€ 9,361,586	###########	###########
1753	0.14	########	########	€ 642,070	€ 856,093	-€ 5,175,021	###########	###########	###########
1754	0.14	########	########	€ 682,704	€ 910,271	-€ 5,502,523	###########	###########	###########
1755	0.17	########	########	€ 787,676	#########	-€ 6,348,590	###########	###########	###########
1756	0.13	########	########	€ 590,536	€ 787,381	-€ 4,759,658	-€ 9,519,316	###########	###########
1757	0.11	########	########	€ 531,189	€ 708,252	-€ 4,281,334	-€ 8,562,667	###########	###########
1758	0.17	########	########	€ 812,981	#########	-€ 6,552,548	###########	###########	###########
1759	0.15	########	########	€ 685,407	€ 913,876	-€ 5,524,314	###########	###########	###########
1760	0.11	########	########	€ 540,953	€ 721,270	-€ 4,360,024	-€ 8,720,049	###########	###########
1761	0.17	########	########	€ 795,745	#########	-€ 6,413,630	###########	###########	###########
1762	0.18	########	########	€ 830,043	#########	-€ 6,690,067	###########	###########	###########
1763	0.22	########	########	#########	#########	-€ 8,283,984	###########	###########	###########
1764	0.19	########	########	€ 913,025	#########	-€ 7,358,894	###########	###########	###########
1765	0.21	########	########	#########	#########	-€ 8,140,132	###########	###########	###########
1766	0.13	########	########	€ 606,246	€ 808,328	-€ 4,886,283	-€ 9,772,567	###########	###########
1767	0.11	########	########	€ 497,847	€ 663,796	-€ 4,012,595	-€ 8,025,190	###########	###########
1768	0.18	########	########	€ 831,151	#########	-€ 6,698,993	###########	###########	###########
1769	0.20	########	########	€ 950,151	#########	-€ 7,658,120	###########	###########	###########
1770	0.16	########	########	€ 746,265	€ 995,020	-€ 6,014,825	###########	###########	###########
1771	0.15	########	########	€ 704,128	€ 938,837	-€ 5,675,202	###########	###########	###########
1772	0.17	########	########	€ 805,152	#########	-€ 6,489,444	###########	###########	###########
1773	0.16	########	########	€ 768,045	#########	-€ 6,190,364	###########	###########	###########
1774	0.22	########	########	#########	#########	-€ 8,308,036	###########	###########	###########
1775	0.15	########	########	€ 731,860	€ 975,813	-€ 5,898,719	###########	###########	###########
1776	0.16	########	########	€ 752,173	#########	-€ 6,062,443	###########	###########	###########
1777	0.14	########	########	€ 665,761	€ 887,681	-€ 5,365,968	###########	###########	###########
1778	0.19	########	########	€ 880,923	#########	-€ 7,100,155	###########	###########	###########
1779	0.10	########	########	€ 483,475	€ 644,633	-€ 3,896,760	-€ 7,793,520	###########	###########
1780	0.12	########	########	€ 564,201	€ 752,268	-€ 4,547,404	-€ 9,094,809	###########	###########
1781	0.15	########	########	€ 706,463	€ 941,950	-€ 5,694,019	###########	###########	###########
1782	0.16	########	########	€ 775,408	#########	-€ 6,249,713	###########	###########	###########
1783	0.17	########	########	€ 793,438	#########	-€ 6,395,033	###########	###########	###########
1784	0.16	########	########	€ 761,632	#########	-€ 6,138,679	###########	###########	###########
1785	0.20	########	########	€ 947,535	#########	-€ 7,637,040	###########	###########	###########
1786	0.12	########	########	€ 561,444	€ 748,592	-€ 4,525,181	-€ 9,050,361	###########	###########
1787	0.15	########	########	€ 690,333	€ 920,444	-€ 5,564,017	###########	###########	###########
1788	0.17	########	########	€ 788,451	#########	-€ 6,354,837	###########	###########	###########
1789	0.17	########	########	€ 786,540	#########	-€ 6,339,437	###########	###########	###########
1790	0.15	########	########	€ 724,273	€ 965,697	-€ 5,837,569	###########	###########	###########
1791	0.17	########	########	€ 796,491	#########	-€ 6,419,637	###########	###########	###########
1792	0.21	########	########	€ 974,582	#########	-€ 7,855,037	###########	###########	###########
1793	0.17	########	########	€ 779,962	#########	-€ 6,286,415	###########	###########	###########
1794	0.16	########	########	€ 779,390	#########	-€ 6,281,810	###########	###########	###########
1795	0.18	########	########	€ 861,429	#########	-€ 6,943,030	###########	###########	###########
1796	0.16	########	########	€ 733,156	€ 977,541	-€ 5,909,163	###########	###########	###########
1797	0.18	########	########	€ 840,270	#########	-€ 6,772,493	###########	###########	###########
1798	0.17	########	########	€ 817,722	#########	-€ 6,590,757	###########	###########	###########
1799	0.16	########	########	€ 754,479	#########	-€ 6,081,023	###########	###########	###########
1800	0.15	########	########	€ 689,789	€ 919,719	-€ 5,559,633	###########	###########	###########
1801	0.14	########	########	€ 667,000	€ 889,333	-€ 5,375,951	###########	###########	###########
1802	0.21	########	########	#########	#########	-€ 8,112,385	###########	###########	###########
1803	0.17	########	########	€ 790,347	#########	-€ 6,370,118	###########	###########	###########
1804	0.17	########	########	€ 803,441	#########	-€ 6,475,653	###########	###########	###########
1805	0.16	########	########	€ 745,504	€ 994,005	-€ 6,008,687	###########	###########	###########
1806	0.18	########	########	€ 852,108	#########	-€ 6,867,908	###########	###########	###########
1807	0.16	########	########	€ 749,987	€ 999,983	-€ 6,044,823	###########	###########	###########
1808	0.14	########	########	€ 658,345	€ 877,793	-€ 5,306,196	###########	###########	###########
1809	0.15	########	########	€ 687,702	€ 916,936	-€ 5,542,809	###########	###########	###########
1810	0.16	########	########	€ 737,337	€ 983,116	-€ 5,942,860	###########	###########	###########
1811	0.17	########	########	€ 783,771	#########	-€ 6,317,120	###########	###########	###########
1812	0.18	########	########	€ 830,022	#########	-€ 6,689,896	###########	###########	###########
1813	0.24	########	########	#########	#########	-€ 9,202,712	###########	###########	###########
1814	0.13	########	########	€ 623,585	€ 831,447	-€ 5,026,036	###########	###########	###########
1815	0.13	########	########	€ 634,788	€ 846,384	-€ 5,116,328	###########	###########	###########
1816	0.20	########	########	€ 929,731	#########	-€ 7,493,538	###########	###########	###########
1817	0.17	########	########	€ 802,965	#########	-€ 6,471,816	###########	###########	###########
1818	0.20	########	########	€ 960,360	#########	-€ 7,740,403	###########	###########	###########
1819	0.19	########	########	€ 903,485	#########	-€ 7,281,997	###########	###########	###########
1820	0.22	########	########	#########	#########	-€ 8,197,630	###########	###########	###########
1821	0.19	########	########	€ 900,696	#########	-€ 7,259,517	###########	###########	###########
1822	0.18	########	########	€ 845,516	#########	-€ 6,814,775	###########	###########	###########
1823	0.15	########	########	€ 713,877	€ 951,836	-€ 5,753,776	###########	###########	###########
1824	0.11	########	########	€ 502,924	€ 670,566	-€ 4,053,520	-€ 8,107,039	###########	###########
1825	0.09	########	########	€ 427,833	€ 570,444	-€ 3,448,292	-€ 6,896,585	###########	###########
1826	0.14	########	########	€ 681,909	€ 909,212	-€ 5,496,119	###########	###########	###########
1827	0.12	########	########	€ 563,695	€ 751,594	-€ 4,543,328	-€ 9,086,656	###########	###########
1828	0.12	########	########	€ 547,337	€ 729,782	-€ 4,411,479	-€ 8,822,959	###########	###########
1829	0.24	########	########	#########	#########	-€ 8,978,356	###########	###########	###########
1830	0.13	########	########	€ 627,215	€ 836,287	-€ 5,055,294	###########	###########	###########
1831	0.10	########	########	€ 493,412	€ 657,883	-€ 3,976,852	-€ 7,953,703	###########	###########
1832	0.17	########	########	€ 791,465	#########	-€ 6,379,131	###########	###########	###########
1833	0.18	########	########	€ 850,372	#########	-€ 6,853,915	###########	###########	###########
1834	0.19	########	########	€ 876,107	#########	-€ 7,061,340	###########	###########	###########
1835	0.12	########	########	€ 543,707	€ 724,943	-€ 4,382,224	-€ 8,764,448	###########	###########
1836	0.16	########	########	€ 768,504	#########	-€ 6,194,070	###########	###########	###########
1837	0.19	########	########	€ 914,962	#########	-€ 7,374,503	###########	###########	###########
1838	0.19	########	########	€ 877,291	#########	-€ 7,070,877	###########	###########	###########
1839	0.23	########	########	#########	#########	-€ 8,941,010	###########	###########	###########
1840	0.20	########	########	€ 930,751	#########	-€ 7,501,759	###########	###########	###########
1841	0.17	########	########	€ 826,135	#########	-€ 6,658,568	###########	###########	###########
1842	0.13	########	########	€ 625,419	€ 833,892	-€ 5,040,816	###########	###########	###########
1843	0.16	########	########	€ 750,635	#########	-€ 6,050,042	###########	###########	###########
1844	0.15	########	########	€ 698,514	€ 931,352	-€ 5,629,956	###########	###########	###########
1845	0.19	########	########	€ 875,693	#########	-€ 7,057,999	###########	###########	###########
1846	0.19	########	########	€ 903,074	#########	-€ 7,278,685	###########	###########	###########
1847	0.16	########	########	€ 740,355	€ 987,140	-€ 5,967,188	###########	###########	###########
1848	0.22	########	########	#########	#########	-€ 8,313,221	###########	###########	###########
1849	0.15	########	########	€ 710,839	€ 947,786	-€ 5,729,294	###########	###########	###########
1850	0.16	########	########	€ 755,350	#########	-€ 6,088,046	###########	###########	###########
1851	0.14	########	########	€ 684,883	€ 913,178	-€ 5,520,093	###########	###########	###########
1852	0.13	########	########	€ 632,631	€ 843,508	-€ 5,098,945	###########	###########	###########
1853	0.17	########	########	€ 786,244	#########	-€ 6,337,045	###########	###########	###########
1854	0.13	########	########	€ 601,758	€ 802,344	-€ 4,850,112	-€ 9,700,223	###########	###########
1855	0.15	########	########	€ 703,930	€ 938,574	-€ 5,673,609	###########	###########	###########
1856	0.23	########	########	#########	#########	-€ 8,707,055	###########	###########	###########
1857	0.13	########	########	€ 596,103	€ 794,804	-€ 4,804,529	-€ 9,609,058	###########	###########
1858	0.14	########	########	€ 666,876	€ 889,168	-€ 5,374,957	###########	###########	###########
1859	0.21	########	########	€ 986,588	#########	-€ 7,951,799	###########	###########	###########
1860	0.17	########	########	€ 800,951	#########	-€ 6,455,584	###########	###########	###########
1861	0.17	########	########	€ 800,649	#########	-€ 6,453,152	###########	###########	###########
1862	0.19	########	########	€ 880,918	#########	-€ 7,100,115	###########	###########	###########
1863	0.25	########	########	#########	#########	-€ 9,359,178	###########	###########	###########
1864	0.19	########	########	€ 911,026	#########	-€ 7,342,779	###########	###########	###########
1865	0.18	########	########	€ 849,474	#########	-€ 6,846,676	###########	###########	###########
1866	0.12	########	########	€ 549,650	€ 732,867	-€ 4,430,126	-€ 8,860,253	###########	###########
1867	0.14	########	########	€ 669,870	€ 893,160	-€ 5,399,084	###########	###########	###########
1868	0.16	########	########	€ 733,214	€ 977,619	-€ 5,909,636	###########	###########	###########
1869	0.15	########	########	€ 710,958	€ 947,944	-€ 5,730,253	###########	###########	###########
1870	0.21	########	########	€ 974,400	#########	-€ 7,853,564	###########	###########	###########
1871	0.20	########	########	€ 923,231	#########	-€ 7,441,152	###########	###########	###########
1872	0.19	########	########	€ 893,349	#########	-€ 7,200,308	###########	###########	###########
1873	0.15	########	########	€ 726,048	€ 968,064	-€ 5,851,873	###########	###########	###########
1874	0.11	########	########	€ 539,386	€ 719,181	-€ 4,347,396	-€ 8,694,791	###########	###########
1875	0.09	########	########	€ 445,844	€ 594,459	-€ 3,593,462	-€ 7,186,924	###########	###########
1876	0.11	########	########	€ 520,902	€ 694,535	-€ 4,198,415	-€ 8,396,829	###########	###########
1877	0.17	########	########	€ 783,474	#########	-€ 6,314,719	###########	###########	###########
1878	0.19	########	########	€ 919,349	#########	-€ 7,409,861	###########	###########	###########
1879	0.18	########	########	€ 872,094	#########	-€ 7,028,989	###########	###########	###########
1880	0.22	########	########	#########	#########	-€ 8,513,376	###########	###########	###########
1881	0.11	########	########	€ 538,241	€ 717,654	-€ 4,338,165	-€ 8,676,331	###########	###########
1882	0.18	########	########	€ 838,187	#########	-€ 6,755,704	###########	###########	###########
1883	0.14	########	########	€ 659,484	€ 879,312	-€ 5,315,377	###########	###########	###########
1884	0.16	########	########	€ 764,002	#########	-€ 6,157,780	###########	###########	###########
1885	0.10	########	########	€ 449,687	€ 599,582	-€ 3,624,429	-€ 7,248,858	###########	###########
1886	0.18	########	########	€ 853,444	#########	-€ 6,878,675	###########	###########	###########
1887	0.15	########	########	€ 715,219	€ 953,626	-€ 5,764,598	###########	###########	###########
1888	0.13	########	########	€ 628,334	€ 837,779	-€ 5,064,313	###########	###########	###########

1889	0.16	########	########	€ 755,553	#########	-€ 6,089,683	###########	###########	###########
1890	0.10	########	########	€ 456,068	€ 608,091	-€ 3,675,866	-€ 7,351,732	###########	###########
1891	0.17	########	########	€ 820,790	#########	-€ 6,615,483	###########	###########	###########
1892	0.15	########	########	€ 704,646	€ 939,528	-€ 5,679,378	###########	###########	###########
1893	0.21	########	########	€ 985,756	#########	-€ 7,945,099	###########	###########	###########
1894	0.21	########	########	#########	#########	-€ 8,171,447	###########	###########	###########
1895	0.17	########	########	€ 780,298	#########	-€ 6,289,122	###########	###########	###########
1896	0.17	########	########	€ 783,071	#########	-€ 6,311,471	###########	###########	###########
1897	0.15	########	########	€ 705,395	€ 940,527	-€ 5,685,417	###########	###########	###########
1898	0.17	########	########	€ 796,751	#########	-€ 6,421,738	###########	###########	###########
1899	0.17	########	########	€ 782,353	#########	-€ 6,305,687	###########	###########	###########
1900	0.11	########	########	€ 540,488	€ 720,651	-€ 4,356,281	-€ 8,712,561	###########	###########
1901	0.16	########	########	€ 740,244	€ 986,992	-€ 5,966,293	###########	###########	###########
1902	0.16	########	########	€ 747,448	€ 996,597	-€ 6,024,353	###########	###########	###########
1903	0.14	########	########	€ 669,866	€ 893,155	-€ 5,399,055	###########	###########	###########
1904	0.19	########	########	€ 913,003	#########	-€ 7,358,710	###########	###########	###########
1905	0.16	########	########	€ 776,258	#########	-€ 6,256,566	###########	###########	###########
1906	0.19	########	########	€ 879,709	#########	-€ 7,090,366	###########	###########	###########
1907	0.10	########	########	€ 451,713	€ 602,285	-€ 3,640,766	-€ 7,281,532	###########	###########
1908	0.15	########	########	€ 697,406	€ 929,875	-€ 5,621,025	###########	###########	###########
1909	0.15	########	########	€ 686,462	€ 915,283	-€ 5,532,817	###########	###########	###########
1910	0.14	########	########	€ 656,481	€ 875,308	-€ 5,291,172	###########	###########	###########
1911	0.22	########	########	#########	#########	-€ 8,308,607	###########	###########	###########
1912	0.09	########	########	€ 403,470	€ 537,960	-€ 3,251,926	-€ 6,503,851	-€ 9,755,777	###########
1913	0.16	########	########	€ 773,701	#########	-€ 6,235,953	###########	###########	###########
1914	0.18	########	########	€ 862,108	#########	-€ 6,948,503	###########	###########	###########
1915	0.18	########	########	€ 843,561	#########	-€ 6,799,018	###########	###########	###########
1916	0.15	########	########	€ 712,979	€ 950,638	-€ 5,746,537	###########	###########	###########
1917	0.15	########	########	€ 706,195	€ 941,593	-€ 5,691,860	###########	###########	###########
1918	0.15	########	########	€ 686,933	€ 915,911	-€ 5,536,612	###########	###########	###########
1919	0.16	########	########	€ 777,559	#########	-€ 6,267,051	###########	###########	###########
1920	0.13	########	########	€ 600,354	€ 800,472	-€ 4,838,793	-€ 9,677,586	###########	###########
1921	0.20	########	########	€ 944,179	#########	-€ 7,609,986	###########	###########	###########
1922	0.22	########	########	#########	#########	-€ 8,229,573	###########	###########	###########
1923	0.20	########	########	€ 933,327	#########	-€ 7,522,524	###########	###########	###########
1924	0.15	########	########	€ 716,353	€ 955,137	-€ 5,773,732	###########	###########	###########
1925	0.15	########	########	€ 702,121	€ 936,161	-€ 5,659,025	###########	###########	###########
1926	0.14	########	########	€ 676,504	€ 902,006	-€ 5,452,557	###########	###########	###########
1927	0.11	########	########	€ 528,604	€ 704,805	-€ 4,260,496	-€ 8,520,992	###########	###########
1928	0.20	########	########	€ 921,295	#########	-€ 7,425,544	###########	###########	###########
1929	0.17	########	########	€ 794,931	#########	-€ 6,407,067	###########	###########	###########
1930	0.20	########	########	€ 951,795	#########	-€ 7,671,372	###########	###########	###########
1931	0.13	########	########	€ 620,690	€ 827,587	-€ 5,002,701	###########	###########	###########
1932	0.15	########	########	€ 687,588	€ 916,784	-€ 5,541,894	###########	###########	###########
1933	0.19	########	########	€ 879,893	#########	-€ 7,091,852	###########	###########	###########
1934	0.09	########	########	€ 438,055	€ 584,073	-€ 3,530,680	-€ 7,061,359	###########	###########
1935	0.27	########	########	#########	#########	###########	###########	###########	###########
1936	0.15	########	########	€ 729,214	€ 972,286	-€ 5,877,395	###########	###########	###########
1937	0.12	########	########	€ 580,751	€ 774,335	-€ 4,680,798	-€ 9,361,595	###########	###########
1938	0.09	########	########	€ 428,336	€ 571,114	-€ 3,452,343	-€ 6,904,686	###########	###########
1939	0.15	########	########	€ 718,730	€ 958,307	-€ 5,792,896	###########	###########	###########
1940	0.08	########	########	€ 389,369	€ 519,158	-€ 3,138,272	-€ 6,276,544	-€ 9,414,816	###########
1941	0.15	########	########	€ 711,147	€ 948,196	-€ 5,731,774	###########	###########	###########
1942	0.19	########	########	€ 889,194	#########	-€ 7,166,813	###########	###########	###########
1943	0.10	########	########	€ 479,355	€ 639,141	-€ 3,863,558	-€ 7,727,116	###########	###########
1944	0.08	########	########	€ 365,439	€ 487,252	-€ 2,945,402	-€ 5,890,804	-€ 8,836,207	###########
1945	0.10	########	########	€ 456,581	€ 608,775	-€ 3,679,997	-€ 7,359,994	###########	###########
1946	0.12	########	########	€ 561,890	€ 749,187	-€ 4,528,779	-€ 9,057,558	###########	###########
1947	0.17	########	########	€ 799,713	#########	-€ 6,445,609	###########	###########	###########
1948	0.16	########	########	€ 740,365	€ 987,154	-€ 5,967,271	###########	###########	###########
1949	0.17	########	########	€ 801,364	#########	-€ 6,458,918	###########	###########	###########
1950	0.22	########	########	#########	#########	-€ 8,473,089	###########	###########	###########
1951	0.16	########	########	€ 767,371	#########	-€ 6,184,938	###########	###########	###########
1952	0.16	########	########	€ 739,028	€ 985,371	-€ 5,956,493	###########	###########	###########
1953	0.18	########	########	€ 845,350	#########	-€ 6,813,435	###########	###########	###########
1954	0.17	########	########	€ 792,832	#########	-€ 6,390,146	###########	###########	###########
1955	0.20	########	########	€ 955,562	#########	-€ 7,701,731	###########	###########	###########
1956	0.13	########	########	€ 616,431	€ 821,908	-€ 4,968,372	-€ 9,936,745	###########	###########
1957	0.14	########	########	€ 640,625	€ 854,166	-€ 5,163,373	###########	###########	###########
1958	0.17	########	########	€ 803,425	#########	-€ 6,475,530	###########	###########	###########
1959	0.23	########	########	#########	#########	-€ 8,696,655	###########	###########	###########
1960	0.17	########	########	€ 816,085	#########	-€ 6,577,560	###########	###########	###########
1961	0.19	########	########	€ 878,472	#########	-€ 7,080,401	###########	###########	###########
1962	0.22	########	########	#########	#########	-€ 8,337,407	###########	###########	###########
1963	0.16	########	########	€ 769,059	#########	-€ 6,198,536	###########	###########	###########
1964	0.22	########	########	#########	#########	-€ 8,237,909	###########	###########	###########
1965	0.10	########	########	€ 456,281	€ 608,375	-€ 3,677,580	-€ 7,355,161	###########	###########
1966	0.13	########	########	€ 603,088	€ 804,118	-€ 4,860,831	-€ 9,721,662	###########	###########
1967	0.15	########	########	€ 694,459	€ 925,945	-€ 5,597,268	###########	###########	###########
1968	0.12	########	########	€ 569,442	€ 759,256	-€ 4,589,644	-€ 9,179,287	###########	###########
1969	0.15	########	########	€ 714,284	€ 952,378	-€ 5,757,055	###########	###########	###########
1970	0.15	########	########	€ 693,378	€ 924,504	-€ 5,588,557	###########	###########	###########
1971	0.15	########	########	€ 714,112	€ 952,149	-€ 5,755,671	###########	###########	###########
1972	0.19	########	########	€ 905,737	#########	-€ 7,300,154	###########	###########	###########
1973	0.18	########	########	€ 868,232	#########	-€ 6,997,864	###########	###########	###########
1974	0.16	########	########	€ 739,481	€ 985,975	-€ 5,960,146	###########	###########	###########
1975	0.18	########	########	€ 850,368	#########	-€ 6,853,883	###########	###########	###########
1976	0.21	########	########	#########	#########	-€ 8,181,259	###########	###########	###########
1977	0.15	########	########	€ 727,634	€ 970,178	-€ 5,864,655	###########	###########	###########
1978	0.13	########	########	€ 611,243	€ 814,990	-€ 4,926,556	-€ 9,853,113	###########	###########
1979	0.17	########	########	€ 826,305	#########	-€ 6,659,938	###########	###########	###########
1980	0.20	########	########	€ 927,958	#########	-€ 7,479,247	###########	###########	###########
1981	0.14	########	########	€ 671,924	€ 895,899	-€ 5,415,643	###########	###########	###########
1982	0.20	########	########	€ 935,463	#########	-€ 7,539,739	###########	###########	###########
1983	0.12	########	########	€ 562,715	€ 750,287	-€ 4,535,429	-€ 9,070,858	###########	###########
1984	0.23	########	########	#########	#########	-€ 8,853,463	###########	###########	###########
1985	0.09	########	########	€ 423,425	€ 564,566	-€ 3,412,760	-€ 6,825,519	###########	###########
1986	0.26	########	########	#########	#########	-€ 9,712,583	###########	###########	###########
1987	0.22	########	########	#########	#########	-€ 8,556,324	###########	###########	###########
1988	0.15	########	########	€ 723,086	€ 964,115	-€ 5,828,002	###########	###########	###########
1989	0.16	########	########	€ 732,207	€ 976,276	-€ 5,901,515	###########	###########	###########
1990	0.13	########	########	€ 596,376	€ 795,168	-€ 4,806,732	-€ 9,613,464	###########	###########
1991	0.14	########	########	€ 674,908	€ 899,877	-€ 5,439,691	###########	###########	###########
1992	0.16	########	########	€ 767,821	#########	-€ 6,188,560	###########	###########	###########
1993	0.17	########	########	€ 804,123	#########	-€ 6,481,148	###########	###########	###########
1994	0.25	########	########	#########	#########	-€ 9,451,046	###########	###########	###########
1995	0.17	########	########	€ 825,554	#########	-€ 6,653,883	###########	###########	###########
1996	0.15	########	########	€ 697,475	€ 929,967	-€ 5,621,583	###########	###########	###########
1997	0.14	########	########	€ 639,600	€ 852,800	-€ 5,155,115	###########	###########	###########
1998	0.13	########	########	€ 605,304	€ 807,072	-€ 4,878,688	-€ 9,757,375	###########	###########
1999	0.20	########	########	€ 937,313	#########	-€ 7,554,648	###########	###########	###########
2000	0.14	########	########	€ 638,646	€ 851,528	-€ 5,147,422	###########	###########	###########
2001	0.19	########	########	€ 874,896	#########	-€ 7,051,576	###########	###########	###########
2002	0.11	########	########	€ 535,680	€ 714,240	-€ 4,317,530	-€ 8,635,060	###########	###########
2003	0.15	########	########	€ 724,449	€ 965,932	-€ 5,838,988	###########	###########	###########
2004	0.17	########	########	€ 805,830	#########	-€ 6,494,912	###########	###########	###########
2005	0.18	########	########	€ 853,862	#########	-€ 6,882,041	###########	###########	###########
2006	0.15	########	########	€ 715,933	€ 954,577	-€ 5,770,350	###########	###########	###########
2007	0.12	########	########	€ 556,788	€ 742,384	-€ 4,487,656	-€ 8,975,312	###########	###########
2008	0.11	########	########	€ 542,961	€ 723,948	-€ 4,376,209	-€ 8,752,418	###########	###########
2009	0.17	########	########	€ 802,618	#########	-€ 6,469,026	###########	###########	###########
2010	0.11	########	########	€ 536,350	€ 715,134	-€ 4,322,931	-€ 8,645,863	###########	###########
2011	0.18	########	########	€ 871,876	#########	-€ 7,027,232	###########	###########	###########
2012	0.11	########	########	€ 506,888	€ 675,851	-€ 4,085,469	-€ 8,170,937	###########	###########
2013	0.17	########	########	€ 788,321	#########	-€ 6,353,792	###########	###########	###########
2014	0.20	########	########	€ 964,005	#########	-€ 7,769,787	###########	###########	###########
2015	0.11	########	########	€ 538,723	€ 718,297	-€ 4,342,051	-€ 8,684,101	###########	###########
2016	0.13	########	########	€ 633,207	€ 844,276	-€ 5,103,588	###########	###########	###########
2017	0.20	########	########	€ 955,134	#########	-€ 7,698,287	###########	###########	###########
2018	0.16	########	########	€ 757,154	#########	-€ 6,102,586	###########	###########	###########
2019	0.18	########	########	€ 859,361	#########	-€ 6,926,364	###########	###########	###########
2020	0.18	########	########	€ 836,204	#########	-€ 6,739,723	###########	###########	###########
2021	0.18	########	########	€ 841,813	#########	-€ 6,784,930	###########	###########	###########
2022	0.17	########	########	€ 790,785	#########	-€ 6,373,650	###########	###########	###########
2023	0.17	########	########	€ 807,834	#########	-€ 6,511,060	###########	###########	###########
2024	0.11	########	########	€ 508,461	€ 677,948	-€ 4,098,148	-€ 8,196,296	###########	###########
2025	0.20	########	########	€ 941,984	#########	-€ 7,592,299	###########	###########	###########
2026	0.16	########	########	€ 770,342	#########	-€ 6,208,882	###########	###########	###########
2027	0.18	########	########	€ 856,415	#########	-€ 6,902,620	###########	###########	###########
2028	0.17	########	########	€ 801,385	#########	-€ 6,459,087	###########	###########	###########
2029	0.19	########	########	€ 882,967	#########	-€ 7,116,625	###########	###########	###########
2030	0.17	########	########	€ 802,542	#########	-€ 6,468,410	###########	###########	###########
2031	0.19	########	########	€ 891,920	#########	-€ 7,188,784	###########	###########	###########
2032	0.16	########	########	€ 736,466	€ 981,955	-€ 5,935,842	###########	###########	###########
2033	0.12	########	########	€ 577,161	€ 769,548	-€ 4,651,863	-€ 9,303,725	###########	###########
2034	0.09	########	########	€ 404,888	€ 539,851	-€ 3,263,359	-€ 6,526,717	-€ 9,790,076	###########
2035	0.26	########	########	#########	#########	-€ 9,906,158	###########	###########	###########
2036	0.15	########	########	€ 693,136	€ 924,181	-€ 5,586,606	###########	###########	###########
2037	0.15	########	########	€ 731,102	€ 974,803	-€ 5,892,613	###########	###########	###########
2038	0.24	########	########	#########	#########	-€ 9,277,203	###########	###########	###########
2039	0.14	########	########	€ 638,551	€ 851,401	-€ 5,146,655	###########	###########	###########
2040	0.30	########	########	#########	#########	###########	###########	###########	###########
2041	0.19	########	########	€ 914,174	#########	-€ 7,368,148	###########	###########	###########
2042	0.17	########	########	€ 826,531	#########	-€ 6,661,761	###########	###########	###########
2043	0.16	########	########	€ 735,622	€ 980,829	-€ 5,929,041	###########	###########	###########
2044	0.16	########	########	€ 735,933	€ 981,243	-€ 5,931,543	###########	###########	###########
2045	0.25	########	########	#########	#########	-€ 9,527,011	###########	###########	###########
2046	0.15	########	########	€ 713,252	€ 951,003	-€ 5,748,743	###########	###########	###########
2047	0.19	########	########	€ 901,931	#########	-€ 7,269,471	###########	###########	###########
2048	0.14	########	########	€ 646,062	€ 861,416	-€ 5,207,194	###########	###########	###########
2049	0.14	########	########	€ 644,552	€ 859,403	-€ 5,195,027	###########	###########	###########
2050	0.13	########	########	€ 600,941	€ 801,255	-€ 4,843,527	-€ 9,687,053	###########	###########
2051	0.12	########	########	€ 570,659	€ 760,878	-€ 4,599,454	-€ 9,198,908	###########	###########
2052	0.16	########	########	€ 755,652	#########	-€ 6,090,479	###########	###########	###########
2053	0.14	########	########	€ 671,263	€ 895,018	-€ 5,410,317	###########	###########	###########
2054	0.19	########	########	€ 910,863	#########	-€ 7,341,468	###########	###########	###########
2055	0.12	########	########	€ 551,685	€ 735,580	-€ 4,446,527	-€ 8,893,054	###########	###########
2056	0.12	########	########	€ 546,885	€ 729,180	-€ 4,407,838	-€ 8,815,677	###########	###########
2057	0.20	########	########	€ 940,082	#########	-€ 7,576,968	###########	###########	###########
2058	0.15	########	########	€ 723,493	€ 964,658	-€ 5,831,285	###########	###########	###########
2059	0.15	########	########	€ 730,560	€ 974,079	-€ 5,888,238	###########	###########	###########
2060	0.16	########	########	€ 743,694	€ 991,592	-€ 5,994,103	###########	###########	###########

2061	0.15	########	########	€ 731,223	€ 974,963	-€ 5,893,581	###########	###########	###########
2062	0.14	########	########	€ 648,887	€ 865,182	-€ 5,229,962	###########	###########	###########
2063	0.16	########	########	€ 746,703	€ 995,604	-€ 6,018,350	###########	###########	###########
2064	0.15	########	########	€ 707,100	€ 942,800	-€ 5,699,155	###########	###########	###########
2065	0.09	########	########	€ 409,280	€ 545,706	-€ 3,298,755	-€ 6,597,510	-€ 9,896,265	###########
2066	0.21	########	########	€ 985,491	#########	-€ 7,942,963	###########	###########	###########
2067	0.17	########	########	€ 794,983	#########	-€ 6,407,481	###########	###########	###########
2068	0.12	########	########	€ 552,625	€ 736,833	-€ 4,454,103	-€ 8,908,205	###########	###########
2069	0.16	########	########	€ 757,288	#########	-€ 6,103,668	###########	###########	###########
2070	0.11	########	########	€ 512,438	€ 683,251	-€ 4,130,202	-€ 8,260,405	###########	###########
2071	0.19	########	########	€ 879,216	#########	-€ 7,086,391	###########	###########	###########
2072	0.12	########	########	€ 575,875	€ 767,833	-€ 4,641,494	-€ 9,282,989	###########	###########
2073	0.20	########	########	€ 941,724	#########	-€ 7,590,200	###########	###########	###########
2074	0.14	########	########	€ 666,822	€ 889,096	-€ 5,374,521	###########	###########	###########
2075	0.14	########	########	€ 656,799	€ 875,731	-€ 5,293,731	###########	###########	###########
2076	0.19	########	########	€ 890,793	#########	-€ 7,179,703	###########	###########	###########
2077	0.18	########	########	€ 866,352	#########	-€ 6,982,711	###########	###########	###########
2078	0.12	########	########	€ 569,609	€ 759,478	-€ 4,590,988	-€ 9,181,977	###########	###########
2079	0.11	########	########	€ 506,882	€ 675,843	-€ 4,085,422	-€ 8,170,843	###########	###########
2080	0.17	########	########	€ 810,247	#########	-€ 6,530,510	###########	###########	###########
2081	0.09	########	########	€ 424,278	€ 565,704	-€ 3,419,641	-€ 6,839,281	###########	###########
2082	0.19	########	########	€ 877,556	#########	-€ 7,073,013	###########	###########	###########
2083	0.19	########	########	€ 895,759	#########	-€ 7,219,725	###########	###########	###########
2084	0.15	########	########	€ 697,377	€ 929,836	-€ 5,620,790	###########	###########	###########
2085	0.18	########	########	€ 873,062	#########	-€ 7,036,795	###########	###########	###########
2086	0.12	########	########	€ 564,074	€ 752,098	-€ 4,546,380	-€ 9,092,759	###########	###########
2087	0.09	########	########	€ 439,971	€ 586,628	-€ 3,546,123	-€ 7,092,247	###########	###########
2088	0.13	########	########	€ 594,295	€ 792,393	-€ 4,789,958	-€ 9,579,916	###########	###########
2089	0.16	########	########	€ 752,130	#########	-€ 6,062,092	###########	###########	###########
2090	0.15	########	########	€ 722,903	€ 963,871	-€ 5,826,526	###########	###########	###########
2091	0.17	########	########	€ 780,964	#########	-€ 6,294,495	###########	###########	###########
2092	0.20	########	########	€ 966,895	#########	-€ 7,793,081	###########	###########	###########
2093	0.15	########	########	€ 721,062	€ 961,417	-€ 5,811,692	###########	###########	###########
2094	0.14	########	########	€ 670,299	€ 893,732	-€ 5,402,547	###########	###########	###########
2095	0.25	########	########	#########	#########	-€ 9,558,606	###########	###########	###########
2096	0.20	########	########	€ 922,542	#########	-€ 7,435,598	###########	###########	###########
2097	0.15	########	########	€ 718,321	€ 957,762	-€ 5,789,597	###########	###########	###########
2098	0.20	########	########	€ 929,662	#########	-€ 7,492,987	###########	###########	###########
2099	0.18	########	########	€ 845,815	#########	-€ 6,817,183	###########	###########	###########
2100	0.18	########	########	€ 859,101	#########	-€ 6,924,273	###########	###########	###########
2101	0.13	########	########	€ 615,016	€ 820,021	-€ 4,956,966	-€ 9,913,931	###########	###########
2102	0.14	########	########	€ 652,918	€ 870,557	-€ 5,262,454	###########	###########	###########
2103	0.14	########	########	€ 673,065	€ 897,420	-€ 5,424,839	###########	###########	###########
2104	0.16	########	########	€ 763,402	#########	-€ 6,152,945	###########	###########	###########
2105	0.14	########	########	€ 679,168	€ 905,557	-€ 5,474,026	###########	###########	###########
2106	0.16	########	########	€ 741,145	€ 988,193	-€ 5,973,556	###########	###########	###########
2107	0.15	########	########	€ 716,373	€ 955,164	-€ 5,773,893	###########	###########	###########
2108	0.18	########	########	€ 846,679	#########	-€ 6,824,149	###########	###########	###########
2109	0.11	########	########	€ 536,618	€ 715,491	-€ 4,325,091	-€ 8,650,182	###########	###########
2110	0.10	########	########	€ 467,288	€ 623,051	-€ 3,766,294	-€ 7,532,588	###########	###########
2111	0.19	########	########	€ 878,605	#########	-€ 7,081,466	###########	###########	###########
2112	0.15	########	########	€ 727,044	€ 969,392	-€ 5,859,901	###########	###########	###########
2113	0.16	########	########	€ 737,150	€ 982,866	-€ 5,941,353	###########	###########	###########
2114	0.10	########	########	€ 472,026	€ 629,369	-€ 3,804,487	-€ 7,608,973	###########	###########
2115	0.25	########	########	#########	#########	-€ 9,354,785	###########	###########	###########
2116	0.15	########	########	€ 712,851	€ 950,468	-€ 5,745,511	###########	###########	###########
2117	0.16	########	########	€ 766,258	#########	-€ 6,175,966	###########	###########	###########
2118	0.13	########	########	€ 631,977	€ 842,636	-€ 5,093,669	###########	###########	###########
2119	0.15	########	########	€ 710,627	€ 947,503	-€ 5,727,584	###########	###########	###########
2120	0.15	########	########	€ 688,696	€ 918,261	-€ 5,550,822	###########	###########	###########
2121	0.11	########	########	€ 517,718	€ 690,291	-€ 4,172,757	-€ 8,345,515	###########	###########
2122	0.16	########	########	€ 760,997	#########	-€ 6,133,564	###########	###########	###########
2123	0.18	########	########	€ 873,029	#########	-€ 7,036,529	###########	###########	###########
2124	0.19	########	########	€ 908,443	#########	-€ 7,321,964	###########	###########	###########
2125	0.21	########	########	#########	#########	-€ 8,130,465	###########	###########	###########
2126	0.22	########	########	#########	#########	-€ 8,284,917	###########	###########	###########
2127	0.10	########	########	€ 472,250	€ 629,667	-€ 3,806,291	-€ 7,612,581	###########	###########
2128	0.18	########	########	€ 853,425	#########	-€ 6,878,521	###########	###########	###########
2129	0.19	########	########	€ 916,539	#########	-€ 7,387,210	###########	###########	###########
2130	0.13	########	########	€ 592,454	€ 789,938	-€ 4,775,119	-€ 9,550,238	###########	###########
2131	0.11	########	########	€ 542,215	€ 722,953	-€ 4,370,196	-€ 8,740,392	###########	###########
2132	0.15	########	########	€ 709,959	€ 946,613	-€ 5,722,203	###########	###########	###########
2133	0.16	########	########	€ 744,986	€ 993,315	-€ 6,004,516	###########	###########	###########
2134	0.12	########	########	€ 575,628	€ 767,504	-€ 4,639,508	-€ 9,279,015	###########	###########
2135	0.15	########	########	€ 728,234	€ 970,978	-€ 5,869,490	###########	###########	###########
2136	0.15	########	########	€ 703,770	€ 938,360	-€ 5,672,314	###########	###########	###########
2137	0.11	########	########	€ 505,409	€ 673,879	-€ 4,073,550	-€ 8,147,100	###########	###########
2138	0.16	########	########	€ 760,533	#########	-€ 6,129,817	###########	###########	###########
2139	0.13	########	########	€ 599,808	€ 799,744	-€ 4,834,392	-€ 9,668,784	###########	###########
2140	0.15	########	########	€ 705,179	€ 940,239	-€ 5,683,675	###########	###########	###########
2141	0.13	########	########	€ 622,114	€ 829,486	-€ 5,014,180	###########	###########	###########
2142	0.15	########	########	€ 707,273	€ 943,031	-€ 5,700,551	###########	###########	###########
2143	0.12	########	########	€ 567,978	€ 757,304	-€ 4,577,849	-€ 9,155,698	###########	###########
2144	0.15	########	########	€ 709,588	€ 946,117	-€ 5,719,208	###########	###########	###########
2145	0.19	########	########	€ 905,596	#########	-€ 7,299,015	###########	###########	###########
2146	0.19	########	########	€ 887,232	#########	-€ 7,151,002	###########	###########	###########
2147	0.19	########	########	€ 882,462	#########	-€ 7,112,556	###########	###########	###########
2148	0.09	########	########	€ 425,129	€ 566,839	-€ 3,426,498	-€ 6,852,995	###########	###########
2149	0.14	########	########	€ 683,023	€ 910,697	-€ 5,505,098	###########	###########	###########
2150	0.18	########	########	€ 845,006	#########	-€ 6,810,668	###########	###########	###########
2151	0.12	########	########	€ 577,598	€ 770,131	-€ 4,655,387	-€ 9,310,773	###########	###########
2152	0.15	########	########	€ 694,961	€ 926,615	-€ 5,601,321	###########	###########	###########
2153	0.13	########	########	€ 615,343	€ 820,458	-€ 4,959,605	-€ 9,919,210	###########	###########
2154	0.12	########	########	€ 554,110	€ 738,814	-€ 4,466,073	-€ 8,932,146	###########	###########
2155	0.22	########	########	#########	#########	-€ 8,265,122	###########	###########	###########
2156	0.13	########	########	€ 599,669	€ 799,559	-€ 4,833,273	-€ 9,666,546	###########	###########
2157	0.25	########	########	#########	#########	-€ 9,481,782	###########	###########	###########
2158	0.11	########	########	€ 531,765	€ 709,021	-€ 4,285,977	-€ 8,571,954	###########	###########
2159	0.14	########	########	€ 681,290	€ 908,386	-€ 5,491,128	###########	###########	###########
2160	0.19	########	########	€ 890,519	#########	-€ 7,177,494	###########	###########	###########
2161	0.10	########	########	€ 471,015	€ 628,020	-€ 3,796,332	-€ 7,592,664	###########	###########
2162	0.16	########	########	€ 751,507	#########	-€ 6,057,076	###########	###########	###########
2163	0.16	########	########	€ 754,245	#########	-€ 6,079,137	###########	###########	###########
2164	0.16	########	########	€ 745,981	€ 994,641	-€ 6,012,530	###########	###########	###########
2165	0.18	########	########	€ 848,275	#########	-€ 6,837,011	###########	###########	###########
2166	0.16	########	########	€ 765,888	#########	-€ 6,172,984	###########	###########	###########
2167	0.12	########	########	€ 570,958	€ 761,277	-€ 4,601,862	-€ 9,203,725	###########	###########
2168	0.14	########	########	€ 651,326	€ 868,434	-€ 5,249,621	###########	###########	###########
2169	0.22	########	########	#########	#########	-€ 8,423,058	###########	###########	###########
2170	0.19	########	########	€ 899,225	#########	-€ 7,247,665	###########	###########	###########
2171	0.19	########	########	€ 915,701	#########	-€ 7,380,458	###########	###########	###########
2172	0.15	########	########	€ 697,804	€ 930,406	-€ 5,624,233	###########	###########	###########
2173	0.14	########	########	€ 656,105	€ 874,806	-€ 5,288,140	###########	###########	###########
2174	0.12	########	########	€ 543,437	€ 724,583	-€ 4,380,051	-€ 8,760,101	###########	###########
2175	0.15	########	########	€ 693,274	€ 924,365	-€ 5,587,717	###########	###########	###########
2176	0.14	########	########	€ 671,512	€ 895,349	-€ 5,412,318	###########	###########	###########
2177	0.12	########	########	€ 580,846	€ 774,461	-€ 4,681,559	-€ 9,363,117	###########	###########
2178	0.14	########	########	€ 639,833	€ 853,110	-€ 5,156,988	###########	###########	###########
2179	0.16	########	########	€ 754,163	#########	-€ 6,078,476	###########	###########	###########
2180	0.17	########	########	€ 783,240	#########	-€ 6,312,835	###########	###########	###########
2181	0.18	########	########	€ 834,874	#########	-€ 6,729,003	###########	###########	###########
2182	0.17	########	########	€ 781,894	#########	-€ 6,301,989	###########	###########	###########
2183	0.21	########	########	€ 997,556	#########	-€ 8,040,199	###########	###########	###########
2184	0.18	########	########	€ 848,368	#########	-€ 6,837,759	###########	###########	###########
2185	0.11	########	########	€ 531,716	€ 708,954	-€ 4,285,577	-€ 8,571,153	###########	###########
2186	0.22	########	########	#########	#########	-€ 8,310,283	###########	###########	###########
2187	0.20	########	########	€ 921,292	#########	-€ 7,425,526	###########	###########	###########
2188	0.18	########	########	€ 839,055	#########	-€ 6,762,699	###########	###########	###########
2189	0.17	########	########	€ 788,275	#########	-€ 6,353,416	###########	###########	###########
2190	0.12	########	########	€ 550,316	€ 733,755	-€ 4,435,492	-€ 8,870,984	###########	###########
2191	0.16	########	########	€ 760,259	#########	-€ 6,127,613	###########	###########	###########
2192	0.22	########	########	#########	#########	-€ 8,331,756	###########	###########	###########
2193	0.16	########	########	€ 759,593	#########	-€ 6,122,244	###########	###########	###########
2194	0.10	########	########	€ 474,831	€ 633,108	-€ 3,827,088	-€ 7,654,176	###########	###########
2195	0.10	########	########	€ 463,306	€ 617,742	-€ 3,734,202	-€ 7,468,404	###########	###########
2196	0.28	########	########	#########	#########	###########	###########	###########	###########
2197	0.13	########	########	€ 632,682	€ 843,576	-€ 5,099,353	###########	###########	###########
2198	0.15	########	########	€ 701,478	€ 935,304	-€ 5,653,846	###########	###########	###########
2199	0.14	########	########	€ 637,915	€ 850,553	-€ 5,141,531	###########	###########	###########
2200	0.18	########	########	€ 829,125	#########	-€ 6,682,669	###########	###########	###########
2201	0.20	########	########	€ 963,711	#########	-€ 7,767,414	###########	###########	###########
2202	0.14	########	########	€ 672,586	€ 896,782	-€ 5,420,980	###########	###########	###########
2203	0.17	########	########	€ 825,577	#########	-€ 6,654,071	###########	###########	###########
2204	0.15	########	########	€ 692,089	€ 922,785	-€ 5,578,168	###########	###########	###########
2205	0.14	########	########	€ 646,291	€ 861,721	-€ 5,209,040	###########	###########	###########
2206	0.17	########	########	€ 780,851	#########	-€ 6,293,583	###########	###########	###########
2207	0.08	########	########	€ 386,152	€ 514,870	-€ 3,112,349	-€ 6,224,697	-€ 9,337,046	###########
2208	0.26	########	########	#########	#########	-€ 9,824,213	###########	###########	###########
2209	0.17	########	########	€ 779,969	#########	-€ 6,286,471	###########	###########	###########
2210	0.16	########	########	€ 735,153	€ 980,204	-€ 5,925,260	###########	###########	###########
2211	0.09	########	########	€ 434,596	€ 579,462	-€ 3,502,803	-€ 7,005,605	###########	###########
2212	0.20	########	########	€ 949,513	#########	-€ 7,652,981	###########	###########	###########
2213	0.16	########	########	€ 743,078	€ 990,771	-€ 5,989,138	###########	###########	###########
2214	0.13	########	########	€ 616,480	€ 821,974	-€ 4,968,769	-€ 9,937,539	###########	###########
2215	0.19	########	########	€ 896,109	#########	-€ 7,222,547	###########	###########	###########
2216	0.17	########	########	€ 806,196	#########	-€ 6,497,856	###########	###########	###########
2217	0.14	########	########	€ 671,371	€ 895,161	-€ 5,411,184	###########	###########	###########
2218	0.26	########	########	#########	#########	###########	###########	###########	###########
2219	0.12	########	########	€ 555,720	€ 740,961	-€ 4,479,052	-€ 8,958,104	###########	###########
2220	0.17	########	########	€ 784,595	#########	-€ 6,323,757	###########	###########	###########
2221	0.12	########	########	€ 575,570	€ 767,427	-€ 4,639,040	-€ 9,278,080	###########	###########
2222	0.16	########	########	€ 755,142	#########	-€ 6,086,368	###########	###########	###########
2223	0.13	########	########	€ 635,416	€ 847,221	-€ 5,121,388	###########	###########	###########
2224	0.16	########	########	€ 762,149	#########	-€ 6,142,848	###########	###########	###########
2225	0.17	########	########	€ 780,476	#########	-€ 6,290,557	###########	###########	###########
2226	0.22	########	########	#########	#########	-€ 8,377,008	###########	###########	###########
2227	0.15	########	########	€ 719,670	€ 959,560	-€ 5,800,468	###########	###########	###########
2228	0.19	########	########	€ 909,588	#########	-€ 7,331,190	###########	###########	###########
2229	0.17	########	########	€ 812,058	#########	-€ 6,545,107	###########	###########	###########
2230	0.13	########	########	€ 621,398	€ 828,531	-€ 5,008,406	###########	###########	###########
2231	0.20	########	########	€ 961,374	#########	-€ 7,748,579	###########	###########	###########
2232	0.14	########	########	€ 663,651	€ 884,868	-€ 5,348,963	###########	###########	###########

2233	0.18	########	########	€ 851,769	#########	-€ 6,865,172	###########	###########	###########
2234	0.20	########	########	€ 930,613	#########	-€ 7,500,649	###########	###########	###########
2235	0.18	########	########	€ 851,158	#########	-€ 6,860,252	###########	###########	###########
2236	0.14	########	########	€ 658,937	€ 878,583	-€ 5,310,967	###########	###########	###########
2237	0.16	########	########	€ 752,806	#########	-€ 6,067,541	###########	###########	###########
2238	0.16	########	########	€ 751,285	#########	-€ 6,055,284	###########	###########	###########
2239	0.12	########	########	€ 545,054	€ 726,738	-€ 4,393,078	-€ 8,786,156	###########	###########
2240	0.09	########	########	€ 424,650	€ 566,200	-€ 3,422,635	-€ 6,845,270	###########	###########
2241	0.18	########	########	€ 829,097	#########	-€ 6,682,438	###########	###########	###########
2242	0.24	########	########	#########	#########	-€ 9,058,407	###########	###########	###########
2243	0.15	########	########	€ 698,462	€ 931,283	-€ 5,629,536	###########	###########	###########
2244	0.15	########	########	€ 720,999	€ 961,333	-€ 5,811,184	###########	###########	###########
2245	0.17	########	########	€ 794,319	#########	-€ 6,402,134	###########	###########	###########
2246	0.17	########	########	€ 794,094	#########	-€ 6,400,316	###########	###########	###########
2247	0.11	########	########	€ 537,260	€ 716,346	-€ 4,330,260	-€ 8,660,519	###########	###########
2248	0.24	########	########	#########	#########	-€ 9,154,916	###########	###########	###########
2249	0.20	########	########	€ 925,672	#########	-€ 7,460,825	###########	###########	###########
2250	0.17	########	########	€ 794,691	#########	-€ 6,405,131	###########	###########	###########
2251	0.13	########	########	€ 616,408	€ 821,877	-€ 4,968,184	-€ 9,936,369	###########	###########
2252	0.17	########	########	€ 780,017	#########	-€ 6,286,862	###########	###########	###########
2253	0.14	########	########	€ 674,442	€ 899,257	-€ 5,435,940	###########	###########	###########
2254	0.07	########	########	€ 343,512	€ 458,016	-€ 2,768,675	-€ 5,537,350	-€ 8,306,025	###########
2255	0.19	########	########	€ 878,705	#########	-€ 7,082,273	###########	###########	###########
2256	0.12	########	########	€ 573,306	€ 764,409	-€ 4,620,793	-€ 9,241,586	###########	###########
2257	0.21	########	########	#########	#########	-€ 8,108,158	###########	###########	###########
2258	0.15	########	########	€ 718,781	€ 958,374	-€ 5,793,301	###########	###########	###########
2259	0.15	########	########	€ 688,584	€ 918,112	-€ 5,549,922	###########	###########	###########
2260	0.17	########	########	€ 812,553	#########	-€ 6,549,100	###########	###########	###########
2261	0.16	########	########	€ 734,643	€ 979,525	-€ 5,921,153	###########	###########	###########
2262	0.14	########	########	€ 657,129	€ 876,172	-€ 5,296,397	###########	###########	###########
2263	0.15	########	########	€ 716,542	€ 955,389	-€ 5,775,255	###########	###########	###########
2264	0.18	########	########	€ 837,151	#########	-€ 6,747,356	###########	###########	###########
2265	0.13	########	########	€ 603,379	€ 804,505	-€ 4,863,171	-€ 9,726,342	###########	###########
2266	0.19	########	########	€ 893,427	#########	-€ 7,200,933	###########	###########	###########
2267	0.20	########	########	€ 930,692	#########	-€ 7,501,286	###########	###########	###########
2268	0.17	########	########	€ 780,831	#########	-€ 6,293,418	###########	###########	###########
2269	0.16	########	########	€ 773,743	#########	-€ 6,236,294	###########	###########	###########
2270	0.10	########	########	€ 487,995	€ 650,660	-€ 3,933,191	-€ 7,866,382	###########	###########
2271	0.13	########	########	€ 618,074	€ 824,099	-€ 4,981,614	-€ 9,963,229	###########	###########
2272	0.10	########	########	€ 495,641	€ 660,854	-€ 3,994,814	-€ 7,989,628	###########	###########
2273	0.21	########	########	€ 984,105	#########	-€ 7,931,792	###########	###########	###########
2274	0.13	########	########	€ 626,793	€ 835,724	-€ 5,051,891	###########	###########	###########
2275	0.11	########	########	€ 517,434	€ 689,912	-€ 4,170,468	-€ 8,340,935	###########	###########
2276	0.21	########	########	€ 974,031	#########	-€ 7,850,592	###########	###########	###########
2277	0.12	########	########	€ 589,667	€ 786,222	-€ 4,752,654	-€ 9,505,308	###########	###########
2278	0.13	########	########	€ 602,966	€ 803,955	-€ 4,859,847	-€ 9,719,693	###########	###########
2279	0.17	########	########	€ 780,674	#########	-€ 6,292,153	###########	###########	###########
2280	0.19	########	########	€ 876,854	#########	-€ 7,067,354	###########	###########	###########
2281	0.15	########	########	€ 724,464	€ 965,952	-€ 5,839,110	###########	###########	###########
2282	0.18	########	########	€ 844,173	#########	-€ 6,803,952	###########	###########	###########
2283	0.15	########	########	€ 713,154	€ 950,872	-€ 5,747,951	###########	###########	###########
2284	0.24	########	########	#########	#########	-€ 8,966,650	###########	###########	###########
2285	0.11	########	########	€ 520,729	€ 694,305	-€ 4,197,020	-€ 8,394,041	###########	###########
2286	0.15	########	########	€ 708,639	€ 944,852	-€ 5,711,561	###########	###########	###########
2287	0.16	########	########	€ 732,177	€ 976,236	-€ 5,901,271	###########	###########	###########
2288	0.14	########	########	€ 669,054	€ 892,072	-€ 5,392,512	###########	###########	###########
2289	0.11	########	########	€ 497,212	€ 662,949	-€ 4,007,479	-€ 8,014,958	###########	###########
2290	0.16	########	########	€ 734,219	€ 978,959	-€ 5,917,736	###########	###########	###########
2291	0.19	########	########	€ 877,252	#########	-€ 7,070,567	###########	###########	###########
2292	0.21	########	########	#########	#########	-€ 8,122,772	###########	###########	###########
2293	0.12	########	########	€ 579,275	€ 772,367	-€ 4,668,899	-€ 9,337,797	###########	###########
2294	0.21	########	########	€ 980,805	#########	-€ 7,905,189	###########	###########	###########
2295	0.16	########	########	€ 752,658	#########	-€ 6,066,350	###########	###########	###########
2296	0.16	########	########	€ 775,723	#########	-€ 6,252,252	###########	###########	###########
2297	0.17	########	########	€ 789,056	#########	-€ 6,359,714	###########	###########	###########
2298	0.14	########	########	€ 647,903	€ 863,870	-€ 5,222,031	###########	###########	###########
2299	0.15	########	########	€ 710,078	€ 946,771	-€ 5,723,161	###########	###########	###########
2300	0.12	########	########	€ 556,032	€ 741,376	-€ 4,481,561	-€ 8,963,122	###########	###########
2301	0.17	########	########	€ 810,590	#########	-€ 6,533,274	###########	###########	###########
2302	0.17	########	########	€ 807,265	#########	-€ 6,506,474	###########	###########	###########
2303	0.15	########	########	€ 700,678	€ 934,238	-€ 5,647,397	###########	###########	###########
2304	0.17	########	########	€ 801,447	#########	-€ 6,459,583	###########	###########	###########
2305	0.22	########	########	#########	#########	-€ 8,454,495	###########	###########	###########
2306	0.18	########	########	€ 839,657	#########	-€ 6,767,552	###########	###########	###########
2307	0.16	########	########	€ 751,058	#########	-€ 6,053,451	###########	###########	###########
2308	0.13	########	########	€ 629,360	€ 839,147	-€ 5,072,583	###########	###########	###########
2309	0.13	########	########	€ 625,315	€ 833,753	-€ 5,039,974	###########	###########	###########
2310	0.14	########	########	€ 671,688	€ 895,584	-€ 5,413,740	###########	###########	###########
2311	0.20	########	########	€ 923,128	#########	-€ 7,440,318	###########	###########	###########
2312	0.10	########	########	€ 463,347	€ 617,796	-€ 3,734,529	-€ 7,469,057	###########	###########
2313	0.14	########	########	€ 678,559	€ 904,746	-€ 5,469,120	###########	###########	###########
2314	0.20	########	########	€ 958,933	#########	-€ 7,728,908	###########	###########	###########
2315	0.17	########	########	€ 813,002	#########	-€ 6,552,715	###########	###########	###########
2316	0.17	########	########	€ 811,874	#########	-€ 6,543,625	###########	###########	###########
2317	0.19	########	########	€ 905,502	#########	-€ 7,298,259	###########	###########	###########
2318	0.16	########	########	€ 756,370	#########	-€ 6,096,268	###########	###########	###########
2319	0.10	########	########	€ 466,338	€ 621,784	-€ 3,758,638	-€ 7,517,276	###########	###########
2320	0.15	########	########	€ 690,055	€ 920,073	-€ 5,561,772	###########	###########	###########
2321	0.15	########	########	€ 699,588	€ 932,784	-€ 5,638,612	###########	###########	###########
2322	0.18	########	########	€ 828,952	#########	-€ 6,681,269	###########	###########	###########
2323	0.18	########	########	€ 840,376	#########	-€ 6,773,349	###########	###########	###########
2324	0.13	########	########	€ 628,444	€ 837,926	-€ 5,065,199	###########	###########	###########
2325	0.12	########	########	€ 560,766	€ 747,688	-€ 4,519,719	-€ 9,039,437	###########	###########
2326	0.17	########	########	€ 788,657	#########	-€ 6,356,497	###########	###########	###########
2327	0.14	########	########	€ 670,814	€ 894,419	-€ 5,406,698	###########	###########	###########
2328	0.10	########	########	€ 454,794	€ 606,392	-€ 3,665,594	-€ 7,331,188	###########	###########
2329	0.19	########	########	€ 917,751	#########	-€ 7,396,985	###########	###########	###########
2330	0.25	########	########	#########	#########	-€ 9,389,838	###########	###########	###########
2331	0.10	########	########	€ 472,080	€ 629,439	-€ 3,804,915	-€ 7,609,830	###########	###########
2332	0.14	########	########	€ 641,513	€ 855,351	-€ 5,170,534	###########	###########	###########
2333	0.17	########	########	€ 780,035	#########	-€ 6,287,003	###########	###########	###########
2334	0.19	########	########	€ 876,360	#########	-€ 7,063,373	###########	###########	###########
2335	0.20	########	########	€ 944,707	#########	-€ 7,614,247	###########	###########	###########
2336	0.18	########	########	€ 841,202	#########	-€ 6,780,006	###########	###########	###########
2337	0.23	########	########	#########	#########	-€ 8,649,823	###########	###########	###########
2338	0.14	########	########	€ 639,807	€ 853,076	-€ 5,156,781	###########	###########	###########
2339	0.16	########	########	€ 773,244	#########	-€ 6,232,268	###########	###########	###########
2340	0.20	########	########	€ 948,059	#########	-€ 7,641,259	###########	###########	###########
2341	0.20	########	########	€ 947,908	#########	-€ 7,640,045	###########	###########	###########
2342	0.11	########	########	€ 497,687	€ 663,582	-€ 4,011,307	-€ 8,022,613	###########	###########
2343	0.17	########	########	€ 795,736	#########	-€ 6,413,557	###########	###########	###########
2344	0.14	########	########	€ 652,895	€ 870,526	-€ 5,262,267	###########	###########	###########
2345	0.19	########	########	€ 910,008	#########	-€ 7,334,575	###########	###########	###########
2346	0.18	########	########	€ 868,327	#########	-€ 6,998,630	###########	###########	###########
2347	0.13	########	########	€ 629,456	€ 839,274	-€ 5,073,349	###########	###########	###########
2348	0.14	########	########	€ 669,660	€ 892,879	-€ 5,397,389	###########	###########	###########
2349	0.16	########	########	€ 778,043	#########	-€ 6,270,948	###########	###########	###########
2350	0.13	########	########	€ 633,233	€ 844,310	-€ 5,103,792	###########	###########	###########
2351	0.15	########	########	€ 712,250	€ 949,667	-€ 5,740,668	###########	###########	###########
2352	0.16	########	########	€ 748,967	€ 998,623	-€ 6,036,599	###########	###########	###########
2353	0.13	########	########	€ 593,932	€ 791,909	-€ 4,787,033	-€ 9,574,065	###########	###########
2354	0.18	########	########	€ 837,174	#########	-€ 6,747,543	###########	###########	###########
2355	0.17	########	########	€ 817,825	#########	-€ 6,591,587	###########	###########	###########
2356	0.24	########	########	#########	#########	-€ 9,067,826	###########	###########	###########
2357	0.14	########	########	€ 643,701	€ 858,268	-€ 5,188,167	###########	###########	###########
2358	0.13	########	########	€ 621,494	€ 828,659	-€ 5,009,179	###########	###########	###########
2359	0.16	########	########	€ 742,345	€ 989,793	-€ 5,983,224	###########	###########	###########
2360	0.15	########	########	€ 724,789	€ 966,386	-€ 5,841,729	###########	###########	###########
2361	0.15	########	########	€ 724,576	€ 966,101	-€ 5,840,008	###########	###########	###########
2362	0.17	########	########	€ 798,532	#########	-€ 6,436,087	###########	###########	###########
2363	0.20	########	########	€ 921,282	#########	-€ 7,425,444	###########	###########	###########
2364	0.16	########	########	€ 740,762	€ 987,682	-€ 5,970,466	###########	###########	###########
2365	0.17	########	########	€ 789,312	#########	-€ 6,361,778	###########	###########	###########
2366	0.22	########	########	#########	#########	-€ 8,318,799	###########	###########	###########
2367	0.09	########	########	€ 409,556	€ 546,074	-€ 3,300,977	-€ 6,601,954	-€ 9,902,931	###########
2368	0.12	########	########	€ 557,093	€ 742,791	-€ 4,490,116	-€ 8,980,232	###########	###########
2369	0.15	########	########	€ 701,758	€ 935,678	-€ 5,656,103	###########	###########	###########
2370	0.16	########	########	€ 771,338	#########	-€ 6,216,911	###########	###########	###########
2371	0.21	########	########	€ 975,311	#########	-€ 7,860,908	###########	###########	###########
2372	0.14	########	########	€ 679,245	€ 905,660	-€ 5,474,649	###########	###########	###########
2373	0.21	########	########	€ 989,968	#########	-€ 7,979,044	###########	###########	###########
2374	0.16	########	########	€ 735,841	€ 981,121	-€ 5,930,805	###########	###########	###########
2375	0.15	########	########	€ 718,884	€ 958,512	-€ 5,794,133	###########	###########	###########
2376	0.17	########	########	€ 808,420	#########	-€ 6,515,784	###########	###########	###########
2377	0.23	########	########	#########	#########	-€ 8,711,751	###########	###########	###########
2378	0.12	########	########	€ 549,343	€ 732,458	-€ 4,427,652	-€ 8,855,304	###########	###########
2379	0.23	########	########	#########	#########	-€ 8,609,675	###########	###########	###########
2380	0.15	########	########	€ 686,801	€ 915,734	-€ 5,535,546	###########	###########	###########
2381	0.12	########	########	€ 589,187	€ 785,582	-€ 4,748,785	-€ 9,497,570	###########	###########
2382	0.24	########	########	#########	#########	-€ 9,224,476	###########	###########	###########
2383	0.13	########	########	€ 636,984	€ 849,312	-€ 5,134,030	###########	###########	###########
2384	0.15	########	########	€ 731,873	€ 975,830	-€ 5,898,820	###########	###########	###########
2385	0.13	########	########	€ 604,773	€ 806,364	-€ 4,874,412	-€ 9,748,823	###########	###########
2386	0.10	########	########	€ 480,750	€ 641,001	-€ 3,874,801	-€ 7,749,602	###########	###########
2387	0.12	########	########	€ 544,192	€ 725,589	-€ 4,386,134	-€ 8,772,268	###########	###########
2388	0.16	########	########	€ 767,216	#########	-€ 6,183,685	###########	###########	###########
2389	0.19	########	########	€ 880,020	#########	-€ 7,092,872	###########	###########	###########
2390	0.23	########	########	#########	#########	-€ 8,657,396	###########	###########	###########
2391	0.20	########	########	€ 928,213	#########	-€ 7,481,307	###########	###########	###########
2392	0.23	########	########	#########	#########	-€ 8,802,795	###########	###########	###########
2393	0.15	########	########	€ 728,980	€ 971,973	-€ 5,875,504	###########	###########	###########
2394	0.18	########	########	€ 866,943	#########	-€ 6,987,473	###########	###########	###########
2395	0.14	########	########	€ 668,747	€ 891,662	-€ 5,390,031	###########	###########	###########
2396	0.16	########	########	€ 736,625	€ 982,166	-€ 5,937,123	###########	###########	###########
2397	0.11	########	########	€ 509,892	€ 679,856	-€ 4,109,676	-€ 8,219,353	###########	###########
2398	0.12	########	########	€ 549,468	€ 732,624	-€ 4,428,659	-€ 8,857,319	###########	###########
2399	0.20	########	########	€ 966,159	#########	-€ 7,787,147	###########	###########	###########
2400	0.18	########	########	€ 832,302	#########	-€ 6,708,272	###########	###########	###########
2401	0.20	########	########	€ 921,551	#########	-€ 7,427,610	###########	###########	###########
2402	0.16	########	########	€ 757,781	#########	-€ 6,107,643	###########	###########	###########
2403	0.19	########	########	€ 885,521	#########	-€ 7,137,208	###########	###########	###########
2404	0.19	########	########	€ 909,289	#########	-€ 7,328,779	###########	###########	###########

2405	0.20	########	########	€ 946,625	#########	-€ 7,629,705	###########	###########	###########
2406	0.20	########	########	€ 931,437	#########	-€ 7,507,289	###########	###########	###########
2407	0.13	########	########	€ 632,808	€ 843,744	-€ 5,100,368	###########	###########	###########
2408	0.17	########	########	€ 787,968	#########	-€ 6,350,942	###########	###########	###########
2409	0.17	########	########	€ 810,109	#########	-€ 6,529,401	###########	###########	###########
2410	0.16	########	########	€ 758,452	#########	-€ 6,113,050	###########	###########	###########
2411	0.15	########	########	€ 699,324	€ 932,432	-€ 5,636,484	###########	###########	###########
2412	0.16	########	########	€ 756,429	#########	-€ 6,096,741	###########	###########	###########
2413	0.14	########	########	€ 656,857	€ 875,809	-€ 5,294,200	###########	###########	###########
2414	0.12	########	########	€ 584,270	€ 779,026	-€ 4,709,157	-€ 9,418,313	###########	###########
2415	0.12	########	########	€ 588,903	€ 785,204	-€ 4,746,498	-€ 9,492,997	###########	###########
2416	0.18	########	########	€ 863,702	#########	-€ 6,961,352	###########	###########	###########
2417	0.12	########	########	€ 577,960	€ 770,613	-€ 4,658,299	-€ 9,316,599	###########	###########
2418	0.15	########	########	€ 711,455	€ 948,606	-€ 5,734,256	###########	###########	###########
2419	0.13	########	########	€ 637,292	€ 849,723	-€ 5,136,510	###########	###########	###########
2420	0.19	########	########	€ 901,220	#########	-€ 7,263,741	###########	###########	###########
2421	0.13	########	########	€ 626,825	€ 835,766	-€ 5,052,145	###########	###########	###########
2422	0.18	########	########	€ 869,160	#########	-€ 7,005,343	###########	###########	###########
2423	0.14	########	########	€ 644,236	€ 858,981	-€ 5,192,478	###########	###########	###########
2424	0.21	########	########	#########	#########	-€ 8,171,212	###########	###########	###########
2425	0.13	########	########	€ 613,001	€ 817,334	-€ 4,940,726	-€ 9,881,451	###########	###########
2426	0.11	########	########	€ 519,949	€ 693,266	-€ 4,190,740	-€ 8,381,480	###########	###########
2427	0.13	########	########	€ 595,333	€ 793,777	-€ 4,798,326	-€ 9,596,651	###########	###########
2428	0.16	########	########	€ 736,776	€ 982,368	-€ 5,938,340	###########	###########	###########
2429	0.15	########	########	€ 730,181	€ 973,575	-€ 5,885,188	###########	###########	###########
2430	0.20	########	########	€ 949,470	#########	-€ 7,652,631	###########	###########	###########
2431	0.16	########	########	€ 758,267	#########	-€ 6,111,555	###########	###########	###########
2432	0.14	########	########	€ 669,336	€ 892,448	-€ 5,394,783	###########	###########	###########
2433	0.15	########	########	€ 698,614	€ 931,486	-€ 5,630,761	###########	###########	###########
2434	0.13	########	########	€ 637,293	€ 849,723	-€ 5,136,515	###########	###########	###########
2435	0.17	########	########	€ 826,055	#########	-€ 6,657,919	###########	###########	###########
2436	0.24	########	########	#########	#########	-€ 8,960,413	###########	###########	###########
2437	0.10	########	########	€ 460,483	€ 613,977	-€ 3,711,443	-€ 7,422,887	###########	###########
2438	0.19	########	########	€ 874,006	#########	-€ 7,044,399	###########	###########	###########
2439	0.16	########	########	€ 740,214	€ 986,952	-€ 5,966,052	###########	###########	###########
2440	0.21	########	########	#########	#########	-€ 8,107,070	###########	###########	###########
2441	0.17	########	########	€ 802,678	#########	-€ 6,469,508	###########	###########	###########
2442	0.14	########	########	€ 639,588	€ 852,784	-€ 5,155,017	###########	###########	###########
2443	0.16	########	########	€ 748,649	€ 998,198	-€ 6,034,033	###########	###########	###########
2444	0.10	########	########	€ 479,937	€ 639,916	-€ 3,868,243	-€ 7,736,486	###########	###########
2445	0.17	########	########	€ 812,988	#########	-€ 6,552,606	###########	###########	###########
2446	0.23	########	########	#########	#########	-€ 8,657,379	###########	###########	###########
2447	0.12	########	########	€ 584,426	€ 779,235	-€ 4,710,417	-€ 9,420,833	###########	###########
2448	0.19	########	########	€ 881,930	#########	-€ 7,108,268	###########	###########	###########
2449	0.20	########	########	€ 929,987	#########	-€ 7,495,604	###########	###########	###########
2450	0.12	########	########	€ 556,056	€ 741,408	-€ 4,481,758	-€ 8,963,515	###########	###########
2451	0.16	########	########	€ 761,698	#########	-€ 6,139,207	###########	###########	###########
2452	0.19	########	########	€ 914,515	#########	-€ 7,370,902	###########	###########	###########
2453	0.11	########	########	€ 532,732	€ 710,309	-€ 4,293,768	-€ 8,587,536	###########	###########
2454	0.13	########	########	€ 595,482	€ 793,976	-€ 4,799,528	-€ 9,599,055	###########	###########
2455	0.14	########	########	€ 669,916	€ 893,222	-€ 5,399,459	###########	###########	###########
2456	0.24	########	########	#########	#########	-€ 8,983,813	###########	###########	###########
2457	0.18	########	########	€ 831,186	#########	-€ 6,699,280	###########	###########	###########
2458	0.18	########	########	€ 853,862	#########	-€ 6,882,040	###########	###########	###########
2459	0.16	########	########	€ 760,829	#########	-€ 6,132,205	###########	###########	###########
2460	0.15	########	########	€ 704,998	€ 939,997	-€ 5,682,213	###########	###########	###########
2461	0.13	########	########	€ 607,192	€ 809,589	-€ 4,893,907	-€ 9,787,815	###########	###########
2462	0.14	########	########	€ 670,533	€ 894,044	-€ 5,404,427	###########	###########	###########
2463	0.12	########	########	€ 569,825	€ 759,766	-€ 4,592,729	-€ 9,185,459	###########	###########
2464	0.19	########	########	€ 904,925	#########	-€ 7,293,608	###########	###########	###########
2465	0.20	########	########	€ 935,728	#########	-€ 7,541,878	###########	###########	###########
2466	0.12	########	########	€ 549,712	€ 732,950	-€ 4,430,626	-€ 8,861,252	###########	###########
2467	0.15	########	########	€ 701,233	€ 934,977	-€ 5,651,869	###########	###########	###########
2468	0.19	########	########	€ 875,254	#########	-€ 7,054,464	###########	###########	###########
2469	0.16	########	########	€ 775,370	#########	-€ 6,249,405	###########	###########	###########
2470	0.15	########	########	€ 699,798	€ 933,064	-€ 5,640,300	###########	###########	###########
2471	0.21	########	########	€ 992,291	#########	-€ 7,997,768	###########	###########	###########
2472	0.12	########	########	€ 550,102	€ 733,470	-€ 4,433,772	-€ 8,867,543	###########	###########
2473	0.18	########	########	€ 861,430	#########	-€ 6,943,037	###########	###########	###########
2474	0.15	########	########	€ 719,283	€ 959,044	-€ 5,797,352	###########	###########	###########
2475	0.11	########	########	€ 513,302	€ 684,403	-€ 4,137,167	-€ 8,274,334	###########	###########
2476	0.14	########	########	€ 683,744	€ 911,659	-€ 5,510,909	###########	###########	###########
2477	0.20	########	########	€ 967,167	#########	-€ 7,795,269	###########	###########	###########
2478	0.11	########	########	€ 504,183	€ 672,244	-€ 4,063,668	-€ 8,127,336	###########	###########
2479	0.24	########	########	#########	#########	-€ 9,103,548	###########	###########	###########
2480	0.15	########	########	€ 723,691	€ 964,922	-€ 5,832,882	###########	###########	###########
2481	0.12	########	########	€ 559,394	€ 745,859	-€ 4,508,662	-€ 9,017,325	###########	###########
2482	0.16	########	########	€ 744,921	€ 993,228	-€ 6,003,988	###########	###########	###########
2483	0.13	########	########	€ 628,874	€ 838,499	-€ 5,068,662	###########	###########	###########
2484	0.19	########	########	€ 887,769	#########	-€ 7,155,331	###########	###########	###########
2485	0.15	########	########	€ 725,538	€ 967,384	-€ 5,847,767	###########	###########	###########
2486	0.18	########	########	€ 848,948	#########	-€ 6,842,436	###########	###########	###########
2487	0.15	########	########	€ 704,781	€ 939,707	-€ 5,680,461	###########	###########	###########
2488	0.23	########	########	#########	#########	-€ 8,688,841	###########	###########	###########
2489	0.15	########	########	€ 732,022	€ 976,029	-€ 5,900,025	###########	###########	###########
2490	0.22	########	########	#########	#########	-€ 8,219,255	###########	###########	###########
2491	0.11	########	########	€ 525,630	€ 700,840	-€ 4,236,528	-€ 8,473,056	###########	###########
2492	0.15	########	########	€ 687,819	€ 917,092	-€ 5,543,752	###########	###########	###########
2493	0.20	########	########	€ 931,606	#########	-€ 7,508,652	###########	###########	###########
2494	0.15	########	########	€ 724,169	€ 965,558	-€ 5,836,728	###########	###########	###########
2495	0.19	########	########	€ 890,568	#########	-€ 7,177,887	###########	###########	###########
2496	0.16	########	########	€ 762,288	#########	-€ 6,143,970	###########	###########	###########
2497	0.17	########	########	€ 813,596	#########	-€ 6,557,501	###########	###########	###########
2498	0.17	########	########	€ 800,190	#########	-€ 6,449,454	###########	###########	###########
2499	0.13	########	########	€ 614,517	€ 819,355	-€ 4,952,943	-€ 9,905,886	###########	###########
2500	0.21	########	########	€ 985,905	#########	-€ 7,946,300	###########	###########	###########
2501	0.16	########	########	€ 778,957	#########	-€ 6,278,320	###########	###########	###########
2502	0.13	########	########	€ 599,814	€ 799,752	-€ 4,834,442	-€ 9,668,885	###########	###########
2503	0.14	########	########	€ 682,655	€ 910,207	-€ 5,502,131	###########	###########	###########
2504	0.16	########	########	€ 742,207	€ 989,610	-€ 5,982,118	###########	###########	###########
2505	0.23	########	########	#########	#########	-€ 8,779,269	###########	###########	###########
2506	0.20	########	########	€ 956,341	#########	-€ 7,708,012	###########	###########	###########
2507	0.21	########	########	#########	#########	-€ 8,116,937	###########	###########	###########
2508	0.15	########	########	€ 706,823	€ 942,430	-€ 5,696,920	###########	###########	###########
2509	0.16	########	########	€ 747,830	€ 997,107	-€ 6,027,438	###########	###########	###########
2510	0.11	########	########	€ 509,212	€ 678,949	-€ 4,104,197	-€ 8,208,395	###########	###########
2511	0.18	########	########	€ 827,810	#########	-€ 6,672,069	###########	###########	###########
2512	0.21	########	########	#########	#########	-€ 8,148,460	###########	###########	###########
2513	0.23	########	########	#########	#########	-€ 8,938,732	###########	###########	###########
2514	0.31	########	########	#########	#########	###########	###########	###########	###########
2515	0.10	########	########	€ 456,184	€ 608,246	-€ 3,676,799	-€ 7,353,599	###########	###########
2516	0.15	########	########	€ 725,194	€ 966,925	-€ 5,844,988	###########	###########	###########
2517	0.20	########	########	€ 941,178	#########	-€ 7,585,798	###########	###########	###########
2518	0.14	########	########	€ 671,200	€ 894,933	-€ 5,409,806	###########	###########	###########
2519	0.17	########	########	€ 799,394	#########	-€ 6,443,033	###########	###########	###########
2520	0.16	########	########	€ 755,913	#########	-€ 6,092,580	###########	###########	###########
2521	0.13	########	########	€ 607,730	€ 810,306	-€ 4,898,241	-€ 9,796,483	###########	###########
2522	0.16	########	########	€ 767,841	#########	-€ 6,188,721	###########	###########	###########
2523	0.13	########	########	€ 609,102	€ 812,136	-€ 4,909,301	-€ 9,818,602	###########	###########
2524	0.12	########	########	€ 545,140	€ 726,854	-€ 4,393,778	-€ 8,787,555	###########	###########
2525	0.16	########	########	€ 755,981	#########	-€ 6,093,131	###########	###########	###########
2526	0.11	########	########	€ 531,989	€ 709,319	-€ 4,287,780	-€ 8,575,561	###########	###########
2527	0.10	########	########	€ 493,622	€ 658,162	-€ 3,978,542	-€ 7,957,084	###########	###########
2528	0.16	########	########	€ 747,034	€ 996,045	-€ 6,021,019	###########	###########	###########
2529	0.16	########	########	€ 738,429	€ 984,572	-€ 5,951,667	###########	###########	###########
2530	0.15	########	########	€ 720,932	€ 961,242	-€ 5,810,638	###########	###########	###########
2531	0.13	########	########	€ 628,652	€ 838,203	-€ 5,066,875	###########	###########	###########
2532	0.12	########	########	€ 584,946	€ 779,928	-€ 4,714,605	-€ 9,429,211	###########	###########
2533	0.15	########	########	€ 706,612	€ 942,149	-€ 5,695,220	###########	###########	###########
2534	0.12	########	########	€ 568,367	€ 757,822	-€ 4,580,980	-€ 9,161,960	###########	###########
2535	0.11	########	########	€ 534,728	€ 712,971	-€ 4,309,855	-€ 8,619,710	###########	###########
2536	0.18	########	########	€ 853,181	#########	-€ 6,876,551	###########	###########	###########
2537	0.18	########	########	€ 856,639	#########	-€ 6,904,422	###########	###########	###########
2538	0.23	########	########	#########	#########	-€ 8,752,212	###########	###########	###########
2539	0.22	########	########	#########	#########	-€ 8,204,720	###########	###########	###########
2540	0.15	########	########	€ 692,702	€ 923,603	-€ 5,583,110	###########	###########	###########
2541	0.19	########	########	€ 897,046	#########	-€ 7,230,102	###########	###########	###########
2542	0.14	########	########	€ 642,998	€ 857,330	-€ 5,182,497	###########	###########	###########
2543	0.20	########	########	€ 930,313	#########	-€ 7,498,229	###########	###########	###########
2544	0.27	########	########	#########	#########	###########	###########	###########	###########
2545	0.10	########	########	€ 465,368	€ 620,490	-€ 3,750,818	-€ 7,501,635	###########	###########
2546	0.18	########	########	€ 865,567	#########	-€ 6,976,384	###########	###########	###########
2547	0.13	########	########	€ 626,845	€ 835,793	-€ 5,052,308	###########	###########	###########
2548	0.11	########	########	€ 542,642	€ 723,522	-€ 4,373,639	-€ 8,747,277	###########	###########
2549	0.19	########	########	€ 906,587	#########	-€ 7,306,998	###########	###########	###########
2550	0.15	########	########	€ 698,804	€ 931,739	-€ 5,632,295	###########	###########	###########
2551	0.14	########	########	€ 645,801	€ 861,068	-€ 5,205,092	###########	###########	###########
2552	0.20	########	########	€ 935,199	#########	-€ 7,537,613	###########	###########	###########
2553	0.17	########	########	€ 806,358	#########	-€ 6,499,167	###########	###########	###########
2554	0.16	########	########	€ 778,234	#########	-€ 6,272,490	###########	###########	###########
2555	0.12	########	########	€ 586,743	€ 782,323	-€ 4,729,087	-€ 9,458,173	###########	###########
2556	0.16	########	########	€ 768,319	#########	-€ 6,192,573	###########	###########	###########
2557	0.08	########	########	€ 370,452	€ 493,936	-€ 2,985,805	-€ 5,971,611	-€ 8,957,416	###########
2558	0.15	########	########	€ 723,201	€ 964,268	-€ 5,828,928	###########	###########	###########
2559	0.16	########	########	€ 745,445	€ 993,926	-€ 6,008,210	###########	###########	###########
2560	0.21	########	########	€ 986,995	#########	-€ 7,955,086	###########	###########	###########
2561	0.08	########	########	€ 394,639	€ 526,186	-€ 3,180,753	-€ 6,361,507	-€ 9,542,260	###########
2562	0.15	########	########	€ 705,393	€ 940,524	-€ 5,685,395	###########	###########	###########
2563	0.15	########	########	€ 724,629	€ 966,173	-€ 5,840,442	###########	###########	###########
2564	0.13	########	########	€ 628,643	€ 838,191	-€ 5,066,801	###########	###########	###########
2565	0.12	########	########	€ 566,296	€ 755,062	-€ 4,564,292	-€ 9,128,585	###########	###########
2566	0.19	########	########	€ 882,581	#########	-€ 7,113,518	###########	###########	###########
2567	0.11	########	########	€ 541,805	€ 722,407	-€ 4,366,896	-€ 8,733,793	###########	###########
2568	0.13	########	########	€ 602,838	€ 803,784	-€ 4,858,812	-€ 9,717,625	###########	###########
2569	0.17	########	########	€ 793,804	#########	-€ 6,397,981	###########	###########	###########
2570	0.17	########	########	€ 797,765	#########	-€ 6,429,904	###########	###########	###########
2571	0.14	########	########	€ 672,056	€ 896,075	-€ 5,416,707	###########	###########	###########
2572	0.21	########	########	€ 983,207	#########	-€ 7,924,555	###########	###########	###########
2573	0.21	########	########	€ 981,691	#########	-€ 7,912,333	###########	###########	###########
2574	0.15	########	########	€ 727,078	€ 969,437	-€ 5,860,174	###########	###########	###########
2575	0.14	########	########	€ 673,222	€ 897,629	-€ 5,426,099	###########	###########	###########
2576	0.15	########	########	€ 721,068	€ 961,425	-€ 5,811,740	###########	###########	###########

2577	0.11	########	########	€ 518,009	€ 690,679	-€ 4,175,101	-€ 8,350,202	###########	###########
2578	0.10	########	########	€ 484,510	€ 646,013	-€ 3,905,101	-€ 7,810,202	###########	###########
2579	0.13	########	########	€ 622,289	€ 829,719	-€ 5,015,588	###########	###########	###########
2580	0.15	########	########	€ 691,496	€ 921,995	-€ 5,573,390	###########	###########	###########
2581	0.12	########	########	€ 585,380	€ 780,507	-€ 4,718,107	-€ 9,436,214	###########	###########
2582	0.15	########	########	€ 685,767	€ 914,356	-€ 5,527,215	###########	###########	###########
2583	0.14	########	########	€ 639,031	€ 852,041	-€ 5,150,523	###########	###########	###########
2584	0.21	########	########	€ 981,434	#########	-€ 7,910,263	###########	###########	###########
2585	0.08	########	########	€ 399,087	€ 532,116	-€ 3,216,601	-€ 6,433,201	-€ 9,649,802	###########
2586	0.18	########	########	€ 833,669	#########	-€ 6,719,291	###########	###########	###########
2587	0.12	########	########	€ 581,157	€ 774,876	-€ 4,684,071	-€ 9,368,142	###########	###########
2588	0.17	########	########	€ 823,983	#########	-€ 6,641,218	###########	###########	###########
2589	0.14	########	########	€ 639,408	€ 852,544	-€ 5,153,565	###########	###########	###########
2590	0.18	########	########	€ 852,300	#########	-€ 6,869,451	###########	###########	###########
2591	0.15	########	########	€ 685,073	€ 913,430	-€ 5,521,618	###########	###########	###########
2592	0.14	########	########	€ 670,434	€ 893,911	-€ 5,403,628	###########	###########	###########
2593	0.17	########	########	€ 824,271	#########	-€ 6,643,544	###########	###########	###########
2594	0.21	########	########	€ 986,788	#########	-€ 7,953,410	###########	###########	###########
2595	0.17	########	########	€ 796,171	#########	-€ 6,417,063	###########	###########	###########
2596	0.12	########	########	€ 575,593	€ 767,457	-€ 4,639,221	-€ 9,278,442	###########	###########
2597	0.17	########	########	€ 823,862	#########	-€ 6,640,243	###########	###########	###########
2598	0.17	########	########	€ 783,663	#########	-€ 6,316,244	###########	###########	###########
2599	0.13	########	########	€ 602,938	€ 803,917	-€ 4,859,618	-€ 9,719,236	###########	###########
2600	0.15	########	########	€ 721,219	€ 961,625	-€ 5,812,955	###########	###########	###########
2601	0.12	########	########	€ 582,148	€ 776,197	-€ 4,692,055	-€ 9,384,109	###########	###########
2602	0.17	########	########	€ 820,692	#########	-€ 6,614,693	###########	###########	###########
2603	0.12	########	########	€ 548,454	€ 731,273	-€ 4,420,489	-€ 8,840,978	###########	###########
2604	0.17	########	########	€ 818,576	#########	-€ 6,597,638	###########	###########	###########
2605	0.12	########	########	€ 544,784	€ 726,378	-€ 4,390,903	-€ 8,781,805	###########	###########
2606	0.14	########	########	€ 668,445	€ 891,260	-€ 5,387,601	###########	###########	###########
2607	0.19	########	########	€ 912,821	#########	-€ 7,357,247	###########	###########	###########
2608	0.20	########	########	€ 954,809	#########	-€ 7,695,664	###########	###########	###########
2609	0.23	########	########	#########	#########	-€ 8,753,803	###########	###########	###########
2610	0.09	########	########	€ 439,812	€ 586,417	-€ 3,544,845	-€ 7,089,690	###########	###########
2611	0.15	########	########	€ 723,747	€ 964,996	-€ 5,833,329	###########	###########	###########
2612	0.20	########	########	€ 936,951	#########	-€ 7,551,735	###########	###########	###########
2613	0.29	########	########	#########	#########	###########	###########	###########	###########
2614	0.17	########	########	€ 812,953	#########	-€ 6,552,324	###########	###########	###########
2615	0.17	########	########	€ 816,246	#########	-€ 6,578,863	###########	###########	###########
2616	0.12	########	########	€ 572,174	€ 762,898	-€ 4,611,663	-€ 9,223,327	###########	###########
2617	0.24	########	########	#########	#########	-€ 9,277,250	###########	###########	###########
2618	0.24	########	########	#########	#########	-€ 9,057,165	###########	###########	###########
2619	0.20	########	########	€ 951,881	#########	-€ 7,672,068	###########	###########	###########
2620	0.13	########	########	€ 591,966	€ 789,288	-€ 4,771,186	-€ 9,542,371	###########	###########
2621	0.21	########	########	#########	#########	-€ 8,060,826	###########	###########	###########
2622	0.20	########	########	€ 927,814	#########	-€ 7,478,087	###########	###########	###########
2623	0.20	########	########	€ 946,380	#########	-€ 7,627,730	###########	###########	###########
2624	0.12	########	########	€ 587,842	€ 783,790	-€ 4,737,951	-€ 9,475,901	###########	###########
2625	0.17	########	########	€ 817,234	#########	-€ 6,586,825	###########	###########	###########
2626	0.22	########	########	#########	#########	-€ 8,473,567	###########	###########	###########
2627	0.11	########	########	€ 539,024	€ 718,699	-€ 4,344,484	-€ 8,688,967	###########	###########
2628	0.18	########	########	€ 862,776	#########	-€ 6,953,893	###########	###########	###########
2629	0.15	########	########	€ 697,051	€ 929,402	-€ 5,618,163	###########	###########	###########
2630	0.12	########	########	€ 574,428	€ 765,905	-€ 4,629,836	-€ 9,259,672	###########	###########
2631	0.16	########	########	€ 748,517	€ 998,022	-€ 6,032,971	###########	###########	###########
2632	0.19	########	########	€ 895,755	#########	-€ 7,219,699	###########	###########	###########
2633	0.15	########	########	€ 704,383	€ 939,177	-€ 5,677,255	###########	###########	###########
2634	0.12	########	########	€ 578,587	€ 771,450	-€ 4,663,358	-€ 9,326,716	###########	###########
2635	0.10	########	########	€ 494,228	€ 658,970	-€ 3,983,425	-€ 7,966,851	###########	###########
2636	0.12	########	########	€ 583,119	€ 777,493	-€ 4,699,885	-€ 9,399,771	###########	###########
2637	0.16	########	########	€ 762,317	#########	-€ 6,144,203	###########	###########	###########
2638	0.16	########	########	€ 755,471	#########	-€ 6,089,021	###########	###########	###########
2639	0.16	########	########	€ 740,039	€ 986,719	-€ 5,964,641	###########	###########	###########
2640	0.10	########	########	€ 481,313	€ 641,750	-€ 3,879,333	-€ 7,758,666	###########	###########
2641	0.15	########	########	€ 716,092	€ 954,789	-€ 5,771,629	###########	###########	###########
2642	0.20	########	########	€ 933,204	#########	-€ 7,521,528	###########	###########	###########
2643	0.14	########	########	€ 660,225	€ 880,300	-€ 5,321,348	###########	###########	###########
2644	0.16	########	########	€ 773,086	#########	-€ 6,231,000	###########	###########	###########
2645	0.19	########	########	€ 881,865	#########	-€ 7,107,741	###########	###########	###########
2646	0.16	########	########	€ 762,677	#########	-€ 6,147,100	###########	###########	###########
2647	0.13	########	########	€ 603,565	€ 804,754	-€ 4,864,677	-€ 9,729,355	###########	###########
2648	0.18	########	########	€ 837,745	#########	-€ 6,752,146	###########	###########	###########
2649	0.10	########	########	€ 495,366	€ 660,488	-€ 3,992,601	-€ 7,985,202	###########	###########
2650	0.22	########	########	#########	#########	-€ 8,214,537	###########	###########	###########
2651	0.23	########	########	#########	#########	-€ 8,721,948	###########	###########	###########
2652	0.16	########	########	€ 771,716	#########	-€ 6,219,955	###########	###########	###########
2653	0.19	########	########	€ 892,189	#########	-€ 7,190,956	###########	###########	###########
2654	0.15	########	########	€ 709,913	€ 946,550	-€ 5,721,826	###########	###########	###########
2655	0.17	########	########	€ 791,151	#########	-€ 6,376,601	###########	###########	###########
2656	0.16	########	########	€ 737,168	€ 982,891	-€ 5,941,504	###########	###########	###########
2657	0.12	########	########	€ 555,115	€ 740,154	-€ 4,474,174	-€ 8,948,349	###########	###########
2658	0.19	########	########	€ 914,710	#########	-€ 7,372,470	###########	###########	###########
2659	0.20	########	########	€ 924,995	#########	-€ 7,455,366	###########	###########	###########
2660	0.13	########	########	€ 619,088	€ 825,451	-€ 4,989,791	-€ 9,979,583	###########	###########
2661	0.11	########	########	€ 513,995	€ 685,326	-€ 4,142,747	-€ 8,285,494	###########	###########
2662	0.22	########	########	#########	#########	-€ 8,520,606	###########	###########	###########
2663	0.18	########	########	€ 830,465	#########	-€ 6,693,464	###########	###########	###########
2664	0.14	########	########	€ 655,699	€ 874,266	-€ 5,284,871	###########	###########	###########
2665	0.22	########	########	#########	#########	-€ 8,270,629	###########	###########	###########
2666	0.16	########	########	€ 768,408	#########	-€ 6,193,291	###########	###########	###########
2667	0.13	########	########	€ 614,958	€ 819,944	-€ 4,956,499	-€ 9,912,999	###########	###########
2668	0.19	########	########	€ 899,535	#########	-€ 7,250,165	###########	###########	###########
2669	0.16	########	########	€ 753,737	#########	-€ 6,075,046	###########	###########	###########
2670	0.16	########	########	€ 759,407	#########	-€ 6,120,744	###########	###########	###########
2671	0.16	########	########	€ 761,439	#########	-€ 6,137,126	###########	###########	###########
2672	0.16	########	########	€ 733,384	€ 977,845	-€ 5,911,002	###########	###########	###########
2673	0.17	########	########	€ 817,908	#########	-€ 6,592,254	###########	###########	###########
2674	0.08	########	########	€ 365,481	€ 487,308	-€ 2,945,741	-€ 5,891,481	-€ 8,837,222	###########
2675	0.14	########	########	€ 658,620	€ 878,160	-€ 5,308,411	###########	###########	###########
2676	0.13	########	########	€ 624,672	€ 832,895	-€ 5,034,791	###########	###########	###########
2677	0.16	########	########	€ 764,085	#########	-€ 6,158,448	###########	###########	###########
2678	0.12	########	########	€ 560,420	€ 747,227	-€ 4,516,931	-€ 9,033,861	###########	###########
2679	0.13	########	########	€ 610,856	€ 814,475	-€ 4,923,439	-€ 9,846,878	###########	###########
2680	0.15	########	########	€ 719,238	€ 958,983	-€ 5,796,984	###########	###########	###########
2681	0.10	########	########	€ 483,220	€ 644,293	-€ 3,894,704	-€ 7,789,408	###########	###########
2682	0.18	########	########	€ 828,156	#########	-€ 6,674,858	###########	###########	###########
2683	0.15	########	########	€ 695,648	€ 927,530	-€ 5,606,851	###########	###########	###########
2684	0.10	########	########	€ 493,573	€ 658,098	-€ 3,978,153	-€ 7,956,306	###########	###########
2685	0.16	########	########	€ 737,540	€ 983,387	-€ 5,944,499	###########	###########	###########
2686	0.18	########	########	€ 862,650	#########	-€ 6,952,870	###########	###########	###########
2687	0.18	########	########	€ 843,538	#########	-€ 6,798,834	###########	###########	###########
2688	0.17	########	########	€ 803,575	#########	-€ 6,476,738	###########	###########	###########
2689	0.11	########	########	€ 533,577	€ 711,436	-€ 4,300,580	-€ 8,601,160	###########	###########
2690	0.16	########	########	€ 733,137	€ 977,516	-€ 5,909,012	###########	###########	###########
2691	0.12	########	########	€ 578,763	€ 771,684	-€ 4,664,771	-€ 9,329,541	###########	###########
2692	0.20	########	########	€ 957,001	#########	-€ 7,713,336	###########	###########	###########
2693	0.09	########	########	€ 448,716	€ 598,288	-€ 3,616,608	-€ 7,233,217	###########	###########
2694	0.11	########	########	€ 537,736	€ 716,982	-€ 4,334,101	-€ 8,668,202	###########	###########
2695	0.20	########	########	€ 951,219	#########	-€ 7,666,735	###########	###########	###########
2696	0.22	########	########	#########	#########	-€ 8,476,953	###########	###########	###########
2697	0.16	########	########	€ 748,238	€ 997,650	-€ 6,030,721	###########	###########	###########
2698	0.19	########	########	€ 912,461	#########	-€ 7,354,349	###########	###########	###########
2699	0.11	########	########	€ 532,027	€ 709,369	-€ 4,288,085	-€ 8,576,170	###########	###########
2700	0.18	########	########	€ 830,648	#########	-€ 6,694,942	###########	###########	###########
2701	0.17	########	########	€ 814,631	#########	-€ 6,565,844	###########	###########	###########
2702	0.15	########	########	€ 714,159	€ 952,211	-€ 5,756,048	###########	###########	###########
2703	0.15	########	########	€ 692,298	€ 923,064	-€ 5,579,855	###########	###########	###########
2704	0.12	########	########	€ 568,407	€ 757,877	-€ 4,581,308	-€ 9,162,615	###########	###########
2705	0.22	########	########	#########	#########	-€ 8,354,146	###########	###########	###########
2706	0.20	########	########	€ 965,383	#########	-€ 7,780,888	###########	###########	###########
2707	0.14	########	########	€ 654,128	€ 872,170	-€ 5,272,206	###########	###########	###########
2708	0.15	########	########	€ 701,419	€ 935,226	-€ 5,653,370	###########	###########	###########
2709	0.24	########	########	#########	#########	-€ 9,244,445	###########	###########	###########
2710	0.16	########	########	€ 763,221	#########	-€ 6,151,487	###########	###########	###########
2711	0.15	########	########	€ 717,367	€ 956,489	-€ 5,781,905	###########	###########	###########
2712	0.14	########	########	€ 674,841	€ 899,788	-€ 5,439,154	###########	###########	###########
2713	0.15	########	########	€ 728,532	€ 971,376	-€ 5,871,896	###########	###########	###########
2714	0.15	########	########	€ 700,311	€ 933,748	-€ 5,644,436	###########	###########	###########
2715	0.23	########	########	#########	#########	-€ 8,669,667	###########	###########	###########
2716	0.19	########	########	€ 883,900	#########	-€ 7,124,148	###########	###########	###########
2717	0.20	########	########	€ 949,592	#########	-€ 7,653,619	###########	###########	###########
2718	0.14	########	########	€ 639,240	€ 852,319	-€ 5,152,208	###########	###########	###########
2719	0.13	########	########	€ 613,339	€ 817,785	-€ 4,943,451	-€ 9,886,901	###########	###########
2720	0.17	########	########	€ 819,182	#########	-€ 6,602,527	###########	###########	###########
2721	0.14	########	########	€ 649,610	€ 866,147	-€ 5,235,794	###########	###########	###########
2722	0.12	########	########	€ 569,591	€ 759,454	-€ 4,590,843	-€ 9,181,686	###########	###########
2723	0.14	########	########	€ 680,128	€ 906,837	-€ 5,481,765	###########	###########	###########
2724	0.15	########	########	€ 697,882	€ 930,509	-€ 5,624,857	###########	###########	###########
2725	0.14	########	########	€ 652,422	€ 869,896	-€ 5,258,454	###########	###########	###########
2726	0.14	########	########	€ 684,870	€ 913,160	-€ 5,519,987	###########	###########	###########
2727	0.14	########	########	€ 662,346	€ 883,127	-€ 5,338,440	###########	###########	###########
2728	0.14	########	########	€ 642,021	€ 856,028	-€ 5,174,625	###########	###########	###########
2729	0.20	########	########	€ 956,434	#########	-€ 7,708,763	###########	###########	###########
2730	0.10	########	########	€ 483,554	€ 644,739	-€ 3,897,399	-€ 7,794,798	###########	###########
2731	0.18	########	########	€ 873,593	#########	-€ 7,041,071	###########	###########	###########
2732	0.18	########	########	€ 832,619	#########	-€ 6,710,824	###########	###########	###########
2733	0.14	########	########	€ 668,911	€ 891,881	-€ 5,391,357	###########	###########	###########
2734	0.20	########	########	€ 962,692	#########	-€ 7,759,201	###########	###########	###########
2735	0.15	########	########	€ 689,735	€ 919,647	-€ 5,559,196	###########	###########	###########
2736	0.16	########	########	€ 766,349	#########	-€ 6,176,695	###########	###########	###########
2737	0.14	########	########	€ 643,698	€ 858,264	-€ 5,188,144	###########	###########	###########
2738	0.12	########	########	€ 586,527	€ 782,035	-€ 4,727,346	-€ 9,454,691	###########	###########
2739	0.13	########	########	€ 597,348	€ 796,464	-€ 4,814,563	-€ 9,629,127	###########	###########
2740	0.18	########	########	€ 844,628	#########	-€ 6,807,621	###########	###########	###########
2741	0.13	########	########	€ 621,107	€ 828,143	-€ 5,006,060	###########	###########	###########
2742	0.21	########	########	€ 998,401	#########	-€ 8,047,009	###########	###########	###########
2743	0.20	########	########	€ 943,299	#########	-€ 7,602,898	###########	###########	###########
2744	0.20	########	########	€ 935,580	#########	-€ 7,540,682	###########	###########	###########
2745	0.19	########	########	€ 892,500	#########	-€ 7,193,463	###########	###########	###########
2746	0.14	########	########	€ 664,692	€ 886,256	-€ 5,357,351	###########	###########	###########
2747	0.11	########	########	€ 497,406	€ 663,208	-€ 4,009,040	-€ 8,018,081	###########	###########
2748	0.16	########	########	€ 777,137	#########	-€ 6,263,646	###########	###########	###########

2749	0.10	########	########	€ 478,579	€ 638,106	-€ 3,857,302	-€ 7,714,603	###########	###########
2750	0.18	########	########	€ 828,262	#########	-€ 6,675,713	###########	###########	###########
2751	0.18	########	########	€ 850,327	#########	-€ 6,853,549	###########	###########	###########
2752	0.17	########	########	€ 800,997	#########	-€ 6,455,958	###########	###########	###########
2753	0.18	########	########	€ 852,173	#########	-€ 6,868,427	###########	###########	###########
2754	0.20	########	########	€ 939,668	#########	-€ 7,573,631	###########	###########	###########
2755	0.17	########	########	€ 780,910	#########	-€ 6,294,061	###########	###########	###########
2756	0.13	########	########	€ 634,794	€ 846,392	-€ 5,116,374	###########	###########	###########
2757	0.10	########	########	€ 492,524	€ 656,699	-€ 3,969,698	-€ 7,939,396	###########	###########
2758	0.16	########	########	€ 733,068	€ 977,423	-€ 5,908,452	###########	###########	###########
2759	0.14	########	########	€ 640,354	€ 853,806	-€ 5,161,193	###########	###########	###########
2760	0.21	########	########	#########	#########	-€ 8,141,478	###########	###########	###########
2761	0.15	########	########	€ 730,493	€ 973,991	-€ 5,887,703	###########	###########	###########
2762	0.10	########	########	€ 490,033	€ 653,378	-€ 3,949,619	-€ 7,899,239	###########	###########
2763	0.12	########	########	€ 543,274	€ 724,365	-€ 4,378,734	-€ 8,757,469	###########	###########
2764	0.13	########	########	€ 614,453	€ 819,271	-€ 4,952,434	-€ 9,904,868	###########	###########
2765	0.10	########	########	€ 483,579	€ 644,773	-€ 3,897,602	-€ 7,795,204	###########	###########
2766	0.14	########	########	€ 677,117	€ 902,823	-€ 5,457,496	###########	###########	###########
2767	0.14	########	########	€ 656,643	€ 875,525	-€ 5,292,481	###########	###########	###########
2768	0.20	########	########	€ 961,150	#########	-€ 7,746,775	###########	###########	###########
2769	0.19	########	########	€ 892,146	#########	-€ 7,190,605	###########	###########	###########
2770	0.13	########	########	€ 606,584	€ 808,779	-€ 4,889,007	-€ 9,778,014	###########	###########
2771	0.11	########	########	€ 501,824	€ 669,099	-€ 4,044,654	-€ 8,089,307	###########	###########
2772	0.13	########	########	€ 617,663	€ 823,551	-€ 4,978,306	-€ 9,956,612	###########	###########
2773	0.15	########	########	€ 709,734	€ 946,313	-€ 5,720,389	###########	###########	###########
2774	0.12	########	########	€ 589,426	€ 785,901	-€ 4,750,716	-€ 9,501,432	###########	###########
2775	0.19	########	########	€ 879,181	#########	-€ 7,086,115	###########	###########	###########
2776	0.14	########	########	€ 651,171	€ 868,228	-€ 5,248,376	###########	###########	###########
2777	0.11	########	########	€ 541,538	€ 722,051	-€ 4,364,746	-€ 8,729,492	###########	###########
2778	0.20	########	########	€ 936,257	#########	-€ 7,546,139	###########	###########	###########
2779	0.12	########	########	€ 578,816	€ 771,755	-€ 4,665,200	-€ 9,330,399	###########	###########
2780	0.19	########	########	€ 877,651	#########	-€ 7,073,783	###########	###########	###########
2781	0.23	########	########	#########	#########	-€ 8,686,670	###########	###########	###########
2782	0.17	########	########	€ 801,773	#########	-€ 6,462,213	###########	###########	###########
2783	0.20	########	########	€ 931,644	#########	-€ 7,508,959	###########	###########	###########
2784	0.17	########	########	€ 780,968	#########	-€ 6,294,522	###########	###########	###########
2785	0.18	########	########	€ 832,383	#########	-€ 6,708,922	###########	###########	###########
2786	0.22	########	########	#########	#########	-€ 8,363,871	###########	###########	###########
2787	0.22	########	########	#########	#########	-€ 8,364,148	###########	###########	###########
2788	0.17	########	########	€ 818,447	#########	-€ 6,596,603	###########	###########	###########
2789	0.19	########	########	€ 880,701	#########	-€ 7,098,366	###########	###########	###########
2790	0.15	########	########	€ 722,373	€ 963,164	-€ 5,822,252	###########	###########	###########
2791	0.17	########	########	€ 817,678	#########	-€ 6,590,401	###########	###########	###########
2792	0.13	########	########	€ 634,055	€ 845,406	-€ 5,110,418	###########	###########	###########
2793	0.15	########	########	€ 724,623	€ 966,164	-€ 5,840,391	###########	###########	###########
2794	0.15	########	########	€ 717,200	€ 956,267	-€ 5,780,562	###########	###########	###########
2795	0.16	########	########	€ 772,584	#########	-€ 6,226,951	###########	###########	###########
2796	0.19	########	########	€ 910,266	#########	-€ 7,336,657	###########	###########	###########
2797	0.10	########	########	€ 487,423	€ 649,897	-€ 3,928,578	-€ 7,857,155	###########	###########
2798	0.19	########	########	€ 900,274	#########	-€ 7,256,123	###########	###########	###########
2799	0.10	########	########	€ 476,258	€ 635,011	-€ 3,838,592	-€ 7,677,184	###########	###########
2800	0.19	########	########	€ 883,896	#########	-€ 7,124,110	###########	###########	###########
2801	0.15	########	########	€ 714,632	€ 952,843	-€ 5,759,864	###########	###########	###########
2802	0.16	########	########	€ 758,837	#########	-€ 6,116,150	###########	###########	###########
2803	0.17	########	########	€ 791,055	#########	-€ 6,375,829	###########	###########	###########
2804	0.11	########	########	€ 523,240	€ 697,654	-€ 4,217,266	-€ 8,434,532	###########	###########
2805	0.19	########	########	€ 876,971	#########	-€ 7,068,299	###########	###########	###########
2806	0.24	########	########	#########	#########	-€ 9,263,931	###########	###########	###########
2807	0.22	########	########	#########	#########	-€ 8,432,259	###########	###########	###########
2808	0.08	########	########	€ 375,261	€ 500,348	-€ 3,024,564	-€ 6,049,128	-€ 9,073,692	###########
2809	0.13	########	########	€ 614,387	€ 819,183	-€ 4,951,897	-€ 9,903,795	###########	###########
2810	0.19	########	########	€ 905,976	#########	-€ 7,302,076	###########	###########	###########
2811	0.16	########	########	€ 753,498	#########	-€ 6,073,120	###########	###########	###########
2812	0.18	########	########	€ 837,059	#########	-€ 6,746,615	###########	###########	###########
2813	0.15	########	########	€ 730,264	€ 973,685	-€ 5,885,857	###########	###########	###########
2814	0.11	########	########	€ 503,666	€ 671,554	-€ 4,059,495	-€ 8,118,989	###########	###########
2815	0.18	########	########	€ 869,537	#########	-€ 7,008,386	###########	###########	###########
2816	0.13	########	########	€ 617,071	€ 822,762	-€ 4,973,533	-€ 9,947,067	###########	###########
2817	0.23	########	########	#########	#########	-€ 8,864,071	###########	###########	###########
2818	0.16	########	########	€ 744,761	€ 993,015	-€ 6,002,701	###########	###########	###########
2819	0.14	########	########	€ 683,165	€ 910,887	-€ 5,506,244	###########	###########	###########
2820	0.17	########	########	€ 815,117	#########	-€ 6,569,766	###########	###########	###########
2821	0.14	########	########	€ 659,691	€ 879,588	-€ 5,317,045	###########	###########	###########
2822	0.21	########	########	€ 977,515	#########	-€ 7,878,675	###########	###########	###########
2823	0.14	########	########	€ 669,603	€ 892,804	-€ 5,396,935	###########	###########	###########
2824	0.18	########	########	€ 870,837	#########	-€ 7,018,858	###########	###########	###########
2825	0.14	########	########	€ 658,481	€ 877,975	-€ 5,307,292	###########	###########	###########
2826	0.18	########	########	€ 846,393	#########	-€ 6,821,841	###########	###########	###########
2827	0.14	########	########	€ 669,474	€ 892,631	-€ 5,395,891	###########	###########	###########
2828	0.21	########	########	€ 979,822	#########	-€ 7,897,269	###########	###########	###########
2829	0.18	########	########	€ 856,377	#########	-€ 6,902,310	###########	###########	###########
2830	0.18	########	########	€ 841,690	#########	-€ 6,783,938	###########	###########	###########
2831	0.21	########	########	€ 985,489	#########	-€ 7,942,947	###########	###########	###########
2832	0.11	########	########	€ 521,394	€ 695,191	-€ 4,202,381	-€ 8,404,762	###########	###########
2833	0.11	########	########	€ 527,237	€ 702,982	-€ 4,249,474	-€ 8,498,948	###########	###########
2834	0.12	########	########	€ 549,887	€ 733,183	-€ 4,432,036	-€ 8,864,073	###########	###########
2835	0.18	########	########	€ 855,960	#########	-€ 6,898,956	###########	###########	###########
2836	0.17	########	########	€ 818,455	#########	-€ 6,596,663	###########	###########	###########
2837	0.20	########	########	€ 931,261	#########	-€ 7,505,873	###########	###########	###########
2838	0.18	########	########	€ 828,679	#########	-€ 6,679,071	###########	###########	###########
2839	0.13	########	########	€ 630,895	€ 841,193	-€ 5,084,948	###########	###########	###########
2840	0.23	########	########	#########	#########	-€ 8,918,619	###########	###########	###########
2841	0.18	########	########	€ 857,166	#########	-€ 6,908,671	###########	###########	###########
2842	0.15	########	########	€ 686,381	€ 915,174	-€ 5,532,159	###########	###########	###########
2843	0.15	########	########	€ 708,826	€ 945,102	-€ 5,713,069	###########	###########	###########
2844	0.15	########	########	€ 699,138	€ 932,184	-€ 5,634,986	###########	###########	###########
2845	0.14	########	########	€ 672,323	€ 896,430	-€ 5,418,854	###########	###########	###########
2846	0.11	########	########	€ 500,607	€ 667,476	-€ 4,034,842	-€ 8,069,683	###########	###########
2847	0.19	########	########	€ 875,548	#########	-€ 7,056,833	###########	###########	###########
2848	0.15	########	########	€ 699,883	€ 933,177	-€ 5,640,985	###########	###########	###########
2849	0.15	########	########	€ 725,670	€ 967,561	-€ 5,848,832	###########	###########	###########
2850	0.13	########	########	€ 605,382	€ 807,176	-€ 4,879,319	-€ 9,758,638	###########	###########
2851	0.23	########	########	#########	#########	-€ 8,854,281	###########	###########	###########
2852	0.17	########	########	€ 799,455	#########	-€ 6,443,527	###########	###########	###########
2853	0.16	########	########	€ 757,638	#########	-€ 6,106,491	###########	###########	###########
2854	0.24	########	########	#########	#########	-€ 9,140,049	###########	###########	###########
2855	0.14	########	########	€ 684,221	€ 912,294	-€ 5,514,751	###########	###########	###########
2856	0.14	########	########	€ 651,622	€ 868,830	-€ 5,252,011	###########	###########	###########
2857	0.12	########	########	€ 589,083	€ 785,445	-€ 4,747,954	-€ 9,495,907	###########	###########
2858	0.16	########	########	€ 739,636	€ 986,181	-€ 5,961,390	###########	###########	###########
2859	0.09	########	########	€ 436,246	€ 581,661	-€ 3,516,098	-€ 7,032,197	###########	###########
2860	0.15	########	########	€ 698,849	€ 931,799	-€ 5,632,657	###########	###########	###########
2861	0.22	########	########	#########	#########	-€ 8,328,297	###########	###########	###########
2862	0.14	########	########	€ 679,315	€ 905,754	-€ 5,475,214	###########	###########	###########
2863	0.14	########	########	€ 677,053	€ 902,737	-€ 5,456,979	###########	###########	###########
2864	0.11	########	########	€ 530,815	€ 707,753	-€ 4,278,313	-€ 8,556,626	###########	###########
2865	0.14	########	########	€ 667,227	€ 889,635	-€ 5,377,780	###########	###########	###########
2866	0.19	########	########	€ 889,840	#########	-€ 7,172,023	###########	###########	###########
2867	0.17	########	########	€ 792,458	#########	-€ 6,387,136	###########	###########	###########
2868	0.14	########	########	€ 638,013	€ 850,684	-€ 5,142,320	###########	###########	###########
2869	0.11	########	########	€ 524,103	€ 698,803	-€ 4,224,215	-€ 8,448,430	###########	###########
2870	0.19	########	########	€ 887,282	#########	-€ 7,151,402	###########	###########	###########
2871	0.09	########	########	€ 412,902	€ 550,537	-€ 3,327,953	-€ 6,655,906	-€ 9,983,859	###########
2872	0.18	########	########	€ 831,361	#########	-€ 6,700,687	###########	###########	###########
2873	0.12	########	########	€ 578,603	€ 771,471	-€ 4,663,484	-€ 9,326,969	###########	###########
2874	0.14	########	########	€ 669,462	€ 892,615	-€ 5,395,794	###########	###########	###########
2875	0.24	########	########	#########	#########	-€ 9,011,194	###########	###########	###########
2876	0.19	########	########	€ 878,641	#########	-€ 7,081,759	###########	###########	###########
2877	0.18	########	########	€ 857,692	#########	-€ 6,912,910	###########	###########	###########
2878	0.11	########	########	€ 527,748	€ 703,663	-€ 4,253,593	-€ 8,507,187	###########	###########
2879	0.21	########	########	€ 977,841	#########	-€ 7,881,302	###########	###########	###########
2880	0.22	########	########	#########	#########	-€ 8,393,141	###########	###########	###########
2881	0.13	########	########	€ 595,133	€ 793,511	-€ 4,796,715	-€ 9,593,429	###########	###########
2882	0.14	########	########	€ 676,770	€ 902,360	-€ 5,454,700	###########	###########	###########
2883	0.21	########	########	#########	#########	-€ 8,129,628	###########	###########	###########
2884	0.22	########	########	#########	#########	-€ 8,425,231	###########	###########	###########
2885	0.18	########	########	€ 838,918	#########	-€ 6,761,598	###########	###########	###########
2886	0.20	########	########	€ 928,104	#########	-€ 7,480,426	###########	###########	###########
2887	0.12	########	########	€ 562,428	€ 749,904	-€ 4,533,112	-€ 9,066,224	###########	###########
2888	0.19	########	########	€ 916,777	#########	-€ 7,389,134	###########	###########	###########
2889	0.14	########	########	€ 662,593	€ 883,457	-€ 5,340,430	###########	###########	###########
2890	0.14	########	########	€ 646,861	€ 862,482	-€ 5,213,639	###########	###########	###########
2891	0.17	########	########	€ 811,749	#########	-€ 6,542,616	###########	###########	###########
2892	0.19	########	########	€ 876,921	#########	-€ 7,067,898	###########	###########	###########
2893	0.15	########	########	€ 705,611	€ 940,815	-€ 5,687,158	###########	###########	###########
2894	0.10	########	########	€ 476,153	€ 634,870	-€ 3,837,743	-€ 7,675,485	###########	###########
2895	0.13	########	########	€ 631,048	€ 841,397	-€ 5,086,181	###########	###########	###########
2896	0.19	########	########	€ 879,315	#########	-€ 7,087,195	###########	###########	###########
2897	0.14	########	########	€ 676,900	€ 902,534	-€ 5,455,749	###########	###########	###########
2898	0.12	########	########	€ 560,293	€ 747,058	-€ 4,515,908	-€ 9,031,815	###########	###########
2899	0.10	########	########	€ 484,015	€ 645,354	-€ 3,901,115	-€ 7,802,231	###########	###########
2900	0.15	########	########	€ 707,520	€ 943,359	-€ 5,702,538	###########	###########	###########
2901	0.20	########	########	€ 965,433	#########	-€ 7,781,295	###########	###########	###########
2902	0.13	########	########	€ 620,943	€ 827,924	-€ 5,004,737	###########	###########	###########
2903	0.11	########	########	€ 496,176	€ 661,568	-€ 3,999,129	-€ 7,998,257	###########	###########
2904	0.21	########	########	#########	#########	-€ 8,137,650	###########	###########	###########
2905	0.17	########	########	€ 787,699	#########	-€ 6,348,772	###########	###########	###########
2906	0.17	########	########	€ 791,671	#########	-€ 6,380,788	###########	###########	###########
2907	0.14	########	########	€ 675,365	€ 900,487	-€ 5,443,377	###########	###########	###########
2908	0.19	########	########	€ 882,220	#########	-€ 7,110,608	###########	###########	###########
2909	0.16	########	########	€ 737,653	€ 983,537	-€ 5,945,409	###########	###########	###########
2910	0.14	########	########	€ 644,954	€ 859,939	-€ 5,198,269	###########	###########	###########
2911	0.07	########	########	€ 329,574	€ 439,432	-€ 2,656,335	-€ 5,312,670	-€ 7,969,005	###########
2912	0.24	########	########	#########	#########	-€ 9,044,575	###########	###########	###########
2913	0.16	########	########	€ 732,266	€ 976,354	-€ 5,901,988	###########	###########	###########
2914	0.18	########	########	€ 857,812	#########	-€ 6,913,877	###########	###########	###########
2915	0.21	########	########	€ 988,379	#########	-€ 7,966,239	###########	###########	###########
2916	0.17	########	########	€ 821,222	#########	-€ 6,618,972	###########	###########	###########
2917	0.12	########	########	€ 550,299	€ 733,733	-€ 4,435,359	-€ 8,870,719	###########	###########
2918	0.16	########	########	€ 773,790	#########	-€ 6,236,669	###########	###########	###########
2919	0.14	########	########	€ 682,921	€ 910,561	-€ 5,504,273	###########	###########	###########
2920	0.20	########	########	€ 936,915	#########	-€ 7,551,438	###########	###########	###########

2921	0.15	########	########	€ 699,521	€ 932,694	-€ 5,638,069	###########	###########	###########
2922	0.14	########	########	€ 649,069	€ 865,425	-€ 5,231,432	###########	###########	###########
2923	0.18	########	########	€ 871,785	#########	-€ 7,026,501	###########	###########	###########
2924	0.14	########	########	€ 679,010	€ 905,347	-€ 5,472,755	###########	###########	###########
2925	0.24	########	########	#########	#########	-€ 9,144,063	###########	###########	###########
2926	0.17	########	########	€ 795,500	#########	-€ 6,411,653	###########	###########	###########
2927	0.08	########	########	€ 385,227	€ 513,636	-€ 3,104,890	-€ 6,209,780	-€ 9,314,671	###########
2928	0.19	########	########	€ 878,200	#########	-€ 7,078,207	###########	###########	###########
2929	0.18	########	########	€ 845,602	#########	-€ 6,815,466	###########	###########	###########
2930	0.18	########	########	€ 840,716	#########	-€ 6,776,088	###########	###########	###########
2931	0.17	########	########	€ 821,097	#########	-€ 6,617,962	###########	###########	###########
2932	0.12	########	########	€ 543,334	€ 724,446	-€ 4,379,219	-€ 8,758,439	###########	###########
2933	0.22	########	########	#########	#########	-€ 8,501,925	###########	###########	###########
2934	0.17	########	########	€ 799,670	#########	-€ 6,445,261	###########	###########	###########
2935	0.16	########	########	€ 760,908	#########	-€ 6,132,843	###########	###########	###########
2936	0.21	########	########	€ 976,192	#########	-€ 7,868,013	###########	###########	###########
2937	0.20	########	########	€ 968,015	#########	-€ 7,802,104	###########	###########	###########
2938	0.14	########	########	€ 655,686	€ 874,248	-€ 5,284,763	###########	###########	###########
2939	0.15	########	########	€ 698,931	€ 931,908	-€ 5,633,317	###########	###########	###########
2940	0.09	########	########	€ 414,879	€ 553,172	-€ 3,343,882	-€ 6,687,764	###########	###########
2941	0.14	########	########	€ 643,285	€ 857,713	-€ 5,184,813	###########	###########	###########
2942	0.19	########	########	€ 878,526	#########	-€ 7,080,830	###########	###########	###########
2943	0.13	########	########	€ 595,084	€ 793,445	-€ 4,796,315	-€ 9,592,630	###########	###########
2944	0.26	########	########	#########	#########	###########	###########	###########	###########
2945	0.17	########	########	€ 807,431	#########	-€ 6,507,818	###########	###########	###########
2946	0.17	########	########	€ 823,214	#########	-€ 6,635,023	###########	###########	###########
2947	0.16	########	########	€ 766,355	#########	-€ 6,176,742	###########	###########	###########
2948	0.11	########	########	€ 508,802	€ 678,402	-€ 4,100,892	-€ 8,201,783	###########	###########
2949	0.15	########	########	€ 692,939	€ 923,918	-€ 5,585,017	###########	###########	###########
2950	0.24	########	########	#########	#########	-€ 8,955,177	###########	###########	###########
2951	0.18	########	########	€ 855,558	#########	-€ 6,895,716	###########	###########	###########
2952	0.19	########	########	€ 911,529	#########	-€ 7,346,835	###########	###########	###########
2953	0.19	########	########	€ 893,165	#########	-€ 7,198,823	###########	###########	###########
2954	0.12	########	########	€ 580,843	€ 774,458	-€ 4,681,541	-€ 9,363,082	###########	###########
2955	0.14	########	########	€ 645,464	€ 860,619	-€ 5,202,378	###########	###########	###########
2956	0.18	########	########	€ 858,642	#########	-€ 6,920,569	###########	###########	###########
2957	0.16	########	########	€ 749,809	€ 999,746	-€ 6,043,388	###########	###########	###########
2958	0.19	########	########	€ 892,767	#########	-€ 7,195,610	###########	###########	###########
2959	0.14	########	########	€ 643,643	€ 858,190	-€ 5,187,696	###########	###########	###########
2960	0.16	########	########	€ 741,992	€ 989,323	-€ 5,980,386	###########	###########	###########
2961	0.17	########	########	€ 798,119	#########	-€ 6,432,758	###########	###########	###########
2962	0.17	########	########	€ 826,574	#########	-€ 6,662,102	###########	###########	###########
2963	0.19	########	########	€ 888,544	#########	-€ 7,161,577	###########	###########	###########
2964	0.18	########	########	€ 838,486	#########	-€ 6,758,114	###########	###########	###########
2965	0.12	########	########	€ 587,133	€ 782,844	-€ 4,732,232	-€ 9,464,463	###########	###########
2966	0.16	########	########	€ 762,231	#########	-€ 6,143,504	###########	###########	###########
2967	0.16	########	########	€ 777,852	#########	-€ 6,269,412	###########	###########	###########
2968	0.17	########	########	€ 785,429	#########	-€ 6,330,480	###########	###########	###########
2969	0.17	########	########	€ 826,191	#########	-€ 6,659,018	###########	###########	###########
2970	0.12	########	########	€ 560,749	€ 747,665	-€ 4,519,579	-€ 9,039,157	###########	###########
2971	0.26	########	########	#########	#########	-€ 9,741,262	###########	###########	###########
2972	0.12	########	########	€ 577,691	€ 770,254	-€ 4,656,129	-€ 9,312,258	###########	###########
2973	0.11	########	########	€ 526,452	€ 701,936	-€ 4,243,150	-€ 8,486,300	###########	###########
2974	0.13	########	########	€ 602,396	€ 803,194	-€ 4,855,250	-€ 9,710,500	###########	###########
2975	0.23	########	########	#########	#########	-€ 8,607,576	###########	###########	###########
2976	0.22	########	########	#########	#########	-€ 8,501,044	###########	###########	###########
2977	0.12	########	########	€ 583,486	€ 777,981	-€ 4,702,840	-€ 9,405,679	###########	###########
2978	0.08	########	########	€ 383,120	€ 510,826	-€ 3,087,907	-€ 6,175,815	-€ 9,263,722	###########
2979	0.13	########	########	€ 634,541	€ 846,055	-€ 5,114,340	###########	###########	###########
2980	0.10	########	########	€ 495,512	€ 660,683	-€ 3,993,778	-€ 7,987,557	###########	###########
2981	0.17	########	########	€ 779,608	#########	-€ 6,283,566	###########	###########	###########
2982	0.14	########	########	€ 645,631	€ 860,841	-€ 5,203,718	###########	###########	###########
2983	0.11	########	########	€ 521,939	€ 695,919	-€ 4,206,777	-€ 8,413,553	###########	###########
2984	0.10	########	########	€ 493,558	€ 658,078	-€ 3,978,031	-€ 7,956,062	###########	###########
2985	0.20	########	########	€ 967,062	#########	-€ 7,794,425	###########	###########	###########
2986	0.14	########	########	€ 648,975	€ 865,300	-€ 5,230,677	###########	###########	###########
2987	0.14	########	########	€ 652,674	€ 870,233	-€ 5,260,491	###########	###########	###########
2988	0.17	########	########	€ 820,920	#########	-€ 6,616,537	###########	###########	###########
2989	0.19	########	########	€ 874,603	#########	-€ 7,049,211	###########	###########	###########
2990	0.21	########	########	€ 970,440	#########	-€ 7,821,648	###########	###########	###########
2991	0.15	########	########	€ 708,854	€ 945,139	-€ 5,713,296	###########	###########	###########
2992	0.19	########	########	€ 885,166	#########	-€ 7,134,351	###########	###########	###########
2993	0.17	########	########	€ 824,837	#########	-€ 6,648,105	###########	###########	###########
2994	0.17	########	########	€ 785,216	#########	-€ 6,328,760	###########	###########	###########
2995	0.16	########	########	€ 739,218	€ 985,624	-€ 5,958,025	###########	###########	###########
2996	0.17	########	########	€ 796,121	#########	-€ 6,416,653	###########	###########	###########
2997	0.15	########	########	€ 717,621	€ 956,828	-€ 5,783,952	###########	###########	###########
2998	0.13	########	########	€ 606,876	€ 809,168	-€ 4,891,361	-€ 9,782,721	###########	###########
2999	0.20	########	########	€ 951,128	#########	-€ 7,665,998	###########	###########	###########
3000	0.19	########	########	€ 898,872	#########	-€ 7,244,819	###########	###########	###########
3001	0.11	########	########	€ 497,123	€ 662,831	-€ 4,006,764	-€ 8,013,528	###########	###########
3002	0.14	########	########	€ 647,353	€ 863,138	-€ 5,217,605	###########	###########	###########
3003	0.14	########	########	€ 639,923	€ 853,231	-€ 5,157,715	###########	###########	###########
3004	0.17	########	########	€ 812,919	#########	-€ 6,552,043	###########	###########	###########
3005	0.16	########	########	€ 768,695	#########	-€ 6,195,602	###########	###########	###########
3006	0.14	########	########	€ 653,854	€ 871,805	-€ 5,269,997	###########	###########	###########
3007	0.16	########	########	€ 760,759	#########	-€ 6,131,641	###########	###########	###########
3008	0.23	########	########	#########	#########	-€ 8,909,640	###########	###########	###########
3009	0.09	########	########	€ 428,814	€ 571,753	-€ 3,456,202	-€ 6,912,405	###########	###########
3010	0.21	########	########	#########	#########	-€ 8,165,742	###########	###########	###########
3011	0.12	########	########	€ 589,258	€ 785,677	-€ 4,749,360	-€ 9,498,719	###########	###########
3012	0.10	########	########	€ 479,842	€ 639,789	-€ 3,867,476	-€ 7,734,952	###########	###########
3013	0.15	########	########	€ 686,789	€ 915,719	-€ 5,535,453	###########	###########	###########
3014	0.18	########	########	€ 848,618	#########	-€ 6,839,778	###########	###########	###########
3015	0.16	########	########	€ 751,356	#########	-€ 6,055,856	###########	###########	###########
3016	0.19	########	########	€ 916,314	#########	-€ 7,385,403	###########	###########	###########
3017	0.06	########	########	€ 304,048	€ 405,397	-€ 2,450,596	-€ 4,901,193	-€ 7,351,789	-€ 9,802,386
3018	0.11	########	########	€ 497,285	€ 663,047	-€ 4,008,069	-€ 8,016,138	###########	###########
3019	0.17	########	########	€ 795,113	#########	-€ 6,408,529	###########	###########	###########
3020	0.15	########	########	€ 694,785	€ 926,380	-€ 5,599,896	###########	###########	###########
3021	0.18	########	########	€ 864,015	#########	-€ 6,963,872	###########	###########	###########
3022	0.16	########	########	€ 740,766	€ 987,688	-€ 5,970,501	###########	###########	###########
3023	0.11	########	########	€ 525,246	€ 700,328	-€ 4,233,430	-€ 8,466,861	###########	###########
3024	0.14	########	########	€ 660,309	€ 880,411	-€ 5,322,022	###########	###########	###########
3025	0.16	########	########	€ 759,006	#########	-€ 6,117,513	###########	###########	###########
3026	0.17	########	########	€ 799,514	#########	-€ 6,444,000	###########	###########	###########
3027	0.20	########	########	€ 958,561	#########	-€ 7,725,911	###########	###########	###########
3028	0.14	########	########	€ 674,741	€ 899,654	-€ 5,438,345	###########	###########	###########
3029	0.16	########	########	€ 775,839	#########	-€ 6,253,190	###########	###########	###########
3030	0.15	########	########	€ 717,942	€ 957,256	-€ 5,786,541	###########	###########	###########
3031	0.18	########	########	€ 839,922	#########	-€ 6,769,690	###########	###########	###########
3032	0.13	########	########	€ 622,967	€ 830,623	-€ 5,021,054	###########	###########	###########
3033	0.20	########	########	€ 929,504	#########	-€ 7,491,708	###########	###########	###########
3034	0.17	########	########	€ 788,142	#########	-€ 6,352,344	###########	###########	###########
3035	0.21	########	########	#########	#########	-€ 8,146,493	###########	###########	###########
3036	0.12	########	########	€ 585,351	€ 780,468	-€ 4,717,870	-€ 9,435,740	###########	###########
3037	0.17	########	########	€ 798,745	#########	-€ 6,437,803	###########	###########	###########
3038	0.17	########	########	€ 800,207	#########	-€ 6,449,589	###########	###########	###########
3039	0.14	########	########	€ 643,823	€ 858,431	-€ 5,189,149	###########	###########	###########
3040	0.16	########	########	€ 745,868	€ 994,490	-€ 6,011,621	###########	###########	###########
3041	0.15	########	########	€ 695,999	€ 927,998	-€ 5,609,681	###########	###########	###########
3042	0.20	########	########	€ 965,024	#########	-€ 7,777,998	###########	###########	###########
3043	0.20	########	########	€ 942,581	#########	-€ 7,597,108	###########	###########	###########
3044	0.14	########	########	€ 649,346	€ 865,794	-€ 5,233,663	###########	###########	###########
3045	0.18	########	########	€ 838,849	#########	-€ 6,761,044	###########	###########	###########
3046	0.22	########	########	#########	#########	-€ 8,233,341	###########	###########	###########
3047	0.12	########	########	€ 555,574	€ 740,766	-€ 4,477,874	-€ 8,955,749	###########	###########
3048	0.17	########	########	€ 824,067	#########	-€ 6,641,902	###########	###########	###########
3049	0.20	########	########	€ 955,413	#########	-€ 7,700,538	###########	###########	###########
3050	0.12	########	########	€ 577,515	€ 770,020	-€ 4,654,715	-€ 9,309,430	###########	###########
3051	0.23	########	########	#########	#########	-€ 8,848,848	###########	###########	###########
3052	0.12	########	########	€ 555,236	€ 740,314	-€ 4,475,145	-€ 8,950,291	###########	###########
3053	0.18	########	########	€ 838,062	#########	-€ 6,754,697	###########	###########	###########
3054	0.20	########	########	€ 934,099	#########	-€ 7,528,747	###########	###########	###########
3055	0.17	########	########	€ 788,663	#########	-€ 6,356,542	###########	###########	###########
3056	0.15	########	########	€ 723,314	€ 964,419	-€ 5,829,838	###########	###########	###########
3057	0.21	########	########	€ 973,452	#########	-€ 7,845,930	###########	###########	###########
3058	0.13	########	########	€ 600,836	€ 801,115	-€ 4,842,678	-€ 9,685,357	###########	###########
3059	0.14	########	########	€ 670,794	€ 894,392	-€ 5,406,534	###########	###########	###########
3060	0.19	########	########	€ 879,938	#########	-€ 7,092,216	###########	###########	###########
3061	0.13	########	########	€ 628,142	€ 837,522	-€ 5,062,761	###########	###########	###########
3062	0.17	########	########	€ 803,278	#########	-€ 6,474,345	###########	###########	###########
3063	0.21	########	########	€ 973,861	#########	-€ 7,849,226	###########	###########	###########
3064	0.10	########	########	€ 456,258	€ 608,344	-€ 3,677,396	-€ 7,354,792	###########	###########
3065	0.13	########	########	€ 597,620	€ 796,826	-€ 4,816,756	-€ 9,633,512	###########	###########
3066	0.21	########	########	€ 994,773	#########	-€ 8,017,775	###########	###########	###########
3067	0.11	########	########	€ 539,765	€ 719,686	-€ 4,350,451	-€ 8,700,902	###########	###########
3068	0.13	########	########	€ 624,359	€ 832,479	-€ 5,032,274	###########	###########	###########
3069	0.12	########	########	€ 546,151	€ 728,201	-€ 4,401,923	-€ 8,803,846	###########	###########
3070	0.19	########	########	€ 907,177	#########	-€ 7,311,758	###########	###########	###########
3071	0.12	########	########	€ 587,672	€ 783,563	-€ 4,736,580	-€ 9,473,159	###########	###########
3072	0.16	########	########	€ 765,761	#########	-€ 6,171,962	###########	###########	###########
3073	0.12	########	########	€ 582,062	€ 776,083	-€ 4,691,365	-€ 9,382,729	###########	###########
3074	0.10	########	########	€ 477,301	€ 636,401	-€ 3,846,999	-€ 7,693,999	###########	###########
3075	0.18	########	########	€ 850,703	#########	-€ 6,856,583	###########	###########	###########
3076	0.14	########	########	€ 658,589	€ 878,119	-€ 5,308,164	###########	###########	###########
3077	0.12	########	########	€ 585,735	€ 780,979	-€ 4,720,963	-€ 9,441,926	###########	###########
3078	0.16	########	########	€ 739,668	€ 986,223	-€ 5,961,647	###########	###########	###########
3079	0.18	########	########	€ 844,694	#########	-€ 6,808,152	###########	###########	###########
3080	0.12	########	########	€ 589,940	€ 786,587	-€ 4,754,859	-€ 9,509,717	###########	###########
3081	0.17	########	########	€ 805,110	#########	-€ 6,489,107	###########	###########	###########
3082	0.24	########	########	#########	#########	-€ 8,984,518	###########	###########	###########
3083	0.19	########	########	€ 919,356	#########	-€ 7,409,917	###########	###########	###########
3084	0.17	########	########	€ 794,052	#########	-€ 6,399,982	###########	###########	###########
3085	0.13	########	########	€ 635,811	€ 847,748	-€ 5,124,576	###########	###########	###########
3086	0.18	########	########	€ 838,751	#########	-€ 6,760,252	###########	###########	###########
3087	0.17	########	########	€ 825,130	#########	-€ 6,650,463	###########	###########	###########
3088	0.15	########	########	€ 699,139	€ 932,185	-€ 5,634,989	###########	###########	###########
3089	0.12	########	########	€ 559,905	€ 746,540	-€ 4,512,781	-€ 9,025,561	###########	###########
3090	0.13	########	########	€ 599,534	€ 799,379	-€ 4,832,188	-€ 9,664,376	###########	###########
3091	0.17	########	########	€ 780,369	#########	-€ 6,289,700	###########	###########	###########
3092	0.13	########	########	€ 621,660	€ 828,880	-€ 5,010,516	###########	###########	###########

3093	0.07	########	########	€ 344,866	€ 459,822	-€ 2,779,587	-€ 5,559,175	-€ 8,338,762	###########
3094	0.10	########	########	€ 488,342	€ 651,123	-€ 3,935,991	-€ 7,871,983	###########	###########
3095	0.18	########	########	€ 862,328	#########	-€ 6,950,278	###########	###########	###########
3096	0.18	########	########	€ 832,914	#########	-€ 6,713,201	###########	###########	###########
3097	0.15	########	########	€ 717,869	€ 957,158	-€ 5,785,951	###########	###########	###########
3098	0.17	########	########	€ 800,447	#########	-€ 6,451,523	###########	###########	###########
3099	0.16	########	########	€ 758,074	#########	-€ 6,109,998	###########	###########	###########
3100	0.13	########	########	€ 631,320	€ 841,761	-€ 5,088,381	###########	###########	###########
3101	0.17	########	########	€ 807,313	#########	-€ 6,506,860	###########	###########	###########
3102	0.18	########	########	€ 854,676	#########	-€ 6,888,603	###########	###########	###########
3103	0.14	########	########	€ 682,358	€ 909,811	-€ 5,499,740	###########	###########	###########
3104	0.19	########	########	€ 912,187	#########	-€ 7,352,136	###########	###########	###########
3105	0.17	########	########	€ 783,742	#########	-€ 6,316,883	###########	###########	###########
3106	0.15	########	########	€ 685,079	€ 913,439	-€ 5,521,669	###########	###########	###########
3107	0.18	########	########	€ 852,105	#########	-€ 6,867,880	###########	###########	###########
3108	0.16	########	########	€ 745,630	€ 994,174	-€ 6,009,707	###########	###########	###########
3109	0.17	########	########	€ 798,081	#########	-€ 6,432,452	###########	###########	###########
3110	0.14	########	########	€ 643,971	€ 858,627	-€ 5,190,339	###########	###########	###########
3111	0.19	########	########	€ 877,708	#########	-€ 7,074,242	###########	###########	###########
3112	0.15	########	########	€ 731,208	€ 974,944	-€ 5,893,462	###########	###########	###########
3113	0.18	########	########	€ 831,485	#########	-€ 6,701,689	###########	###########	###########
3114	0.18	########	########	€ 831,243	#########	-€ 6,699,739	###########	###########	###########
3115	0.16	########	########	€ 753,968	#########	-€ 6,076,906	###########	###########	###########
3116	0.12	########	########	€ 560,801	€ 747,735	-€ 4,520,002	-€ 9,040,003	###########	###########
3117	0.15	########	########	€ 721,748	€ 962,331	-€ 5,817,220	###########	###########	###########
3118	0.14	########	########	€ 655,040	€ 873,386	-€ 5,279,554	###########	###########	###########
3119	0.14	########	########	€ 648,452	€ 864,602	-€ 5,226,455	###########	###########	###########
3120	0.15	########	########	€ 712,771	€ 950,361	-€ 5,744,860	###########	###########	###########
3121	0.17	########	########	€ 796,044	#########	-€ 6,416,038	###########	###########	###########
3122	0.15	########	########	€ 706,618	€ 942,158	-€ 5,695,274	###########	###########	###########
3123	0.23	########	########	#########	#########	-€ 8,863,590	###########	###########	###########
3124	0.19	########	########	€ 883,829	#########	-€ 7,123,575	###########	###########	###########
3125	0.18	########	########	€ 830,626	#########	-€ 6,694,764	###########	###########	###########
3126	0.11	########	########	€ 531,425	€ 708,566	-€ 4,283,231	-€ 8,566,463	###########	###########
3127	0.19	########	########	€ 882,743	#########	-€ 7,114,821	###########	###########	###########
3128	0.17	########	########	€ 798,891	#########	-€ 6,438,984	###########	###########	###########
3129	0.24	########	########	#########	#########	-€ 9,066,775	###########	###########	###########
3130	0.15	########	########	€ 724,082	€ 965,443	-€ 5,836,030	###########	###########	###########
3131	0.16	########	########	€ 770,391	#########	-€ 6,209,278	###########	###########	###########
3132	0.13	########	########	€ 590,553	€ 787,403	-€ 4,759,796	-€ 9,519,591	###########	###########
3133	0.14	########	########	€ 666,085	€ 888,113	-€ 5,368,579	###########	###########	###########
3134	0.16	########	########	€ 777,124	#########	-€ 6,263,541	###########	###########	###########
3135	0.14	########	########	€ 679,769	€ 906,359	-€ 5,478,874	###########	###########	###########
3136	0.19	########	########	€ 890,243	#########	-€ 7,175,268	###########	###########	###########
3137	0.13	########	########	€ 615,740	€ 820,986	-€ 4,962,801	-€ 9,925,603	###########	###########
3138	0.15	########	########	€ 711,384	€ 948,512	-€ 5,733,686	###########	###########	###########
3139	0.18	########	########	€ 835,481	#########	-€ 6,733,895	###########	###########	###########
3140	0.18	########	########	€ 864,952	#########	-€ 6,971,431	###########	###########	###########
3141	0.21	########	########	#########	#########	-€ 8,098,504	###########	###########	###########
3142	0.20	########	########	€ 934,898	#########	-€ 7,535,189	###########	###########	###########
3143	0.14	########	########	€ 665,018	€ 886,690	-€ 5,359,976	###########	###########	###########
3144	0.18	########	########	€ 855,978	#########	-€ 6,899,095	###########	###########	###########
3145	0.11	########	########	€ 519,178	€ 692,237	-€ 4,184,522	-€ 8,369,043	###########	###########
3146	0.10	########	########	€ 489,381	€ 652,508	-€ 3,944,363	-€ 7,888,726	###########	###########
3147	0.19	########	########	€ 912,565	#########	-€ 7,355,183	###########	###########	###########
3148	0.13	########	########	€ 618,073	€ 824,097	-€ 4,981,605	-€ 9,963,209	###########	###########
3149	0.21	########	########	#########	#########	-€ 8,180,729	###########	###########	###########
3150	0.16	########	########	€ 761,551	#########	-€ 6,138,027	###########	###########	###########
3151	0.20	########	########	€ 935,263	#########	-€ 7,538,124	###########	###########	###########
3152	0.18	########	########	€ 870,863	#########	-€ 7,019,070	###########	###########	###########
3153	0.27	########	########	#########	#########	###########	###########	###########	###########
3154	0.13	########	########	€ 618,490	€ 824,654	-€ 4,984,970	-€ 9,969,940	###########	###########
3155	0.13	########	########	€ 594,623	€ 792,831	-€ 4,792,605	-€ 9,585,210	###########	###########
3156	0.14	########	########	€ 663,869	€ 885,158	-€ 5,350,717	###########	###########	###########
3157	0.19	########	########	€ 880,300	#########	-€ 7,095,133	###########	###########	###########
3158	0.13	########	########	€ 618,451	€ 824,601	-€ 4,984,654	-€ 9,969,308	###########	###########
3159	0.14	########	########	€ 656,976	€ 875,968	-€ 5,295,160	###########	###########	###########
3160	0.13	########	########	€ 603,624	€ 804,832	-€ 4,865,152	-€ 9,730,304	###########	###########
3161	0.15	########	########	€ 696,158	€ 928,211	-€ 5,610,965	###########	###########	###########
3162	0.23	########	########	#########	#########	-€ 8,871,266	###########	###########	###########
3163	0.16	########	########	€ 763,019	#########	-€ 6,149,861	###########	###########	###########
3164	0.14	########	########	€ 682,663	€ 910,218	-€ 5,502,199	###########	###########	###########
3165	0.09	########	########	€ 403,393	€ 537,858	-€ 3,251,312	-€ 6,502,623	-€ 9,753,935	###########
3166	0.21	########	########	€ 970,197	#########	-€ 7,819,690	###########	###########	###########
3167	0.21	########	########	#########	#########	-€ 8,144,312	###########	###########	###########
3168	0.19	########	########	€ 911,184	#########	-€ 7,344,054	###########	###########	###########
3169	0.17	########	########	€ 797,166	#########	-€ 6,425,082	###########	###########	###########
3170	0.19	########	########	€ 874,389	#########	-€ 7,047,492	###########	###########	###########
3171	0.20	########	########	€ 942,977	#########	-€ 7,600,299	###########	###########	###########
3172	0.18	########	########	€ 841,362	#########	-€ 6,781,298	###########	###########	###########
3173	0.18	########	########	€ 844,068	#########	-€ 6,803,107	###########	###########	###########
3174	0.14	########	########	€ 662,337	€ 883,116	-€ 5,338,371	###########	###########	###########
3175	0.15	########	########	€ 700,109	€ 933,479	-€ 5,642,811	###########	###########	###########
3176	0.13	########	########	€ 632,667	€ 843,556	-€ 5,099,232	###########	###########	###########
3177	0.12	########	########	€ 565,635	€ 754,179	-€ 4,558,958	-€ 9,117,917	###########	###########
3178	0.23	########	########	#########	#########	-€ 8,569,051	###########	###########	###########
3179	0.19	########	########	€ 884,357	#########	-€ 7,127,831	###########	###########	###########
3180	0.10	########	########	€ 490,365	€ 653,819	-€ 3,952,289	-€ 7,904,579	###########	###########
3181	0.14	########	########	€ 658,696	€ 878,261	-€ 5,309,023	###########	###########	###########
3182	0.11	########	########	€ 537,791	€ 717,054	-€ 4,334,540	-€ 8,669,080	###########	###########
3183	0.11	########	########	€ 511,974	€ 682,633	-€ 4,126,464	-€ 8,252,927	###########	###########
3184	0.13	########	########	€ 618,903	€ 825,204	-€ 4,988,295	-€ 9,976,590	###########	###########
3185	0.14	########	########	€ 668,671	€ 891,561	-€ 5,389,419	###########	###########	###########
3186	0.13	########	########	€ 591,984	€ 789,311	-€ 4,771,329	-€ 9,542,658	###########	###########
3187	0.13	########	########	€ 615,962	€ 821,283	-€ 4,964,592	-€ 9,929,184	###########	###########
3188	0.19	########	########	€ 877,062	#########	-€ 7,069,036	###########	###########	###########
3189	0.21	########	########	#########	#########	-€ 8,080,381	###########	###########	###########
3190	0.11	########	########	€ 506,227	€ 674,969	-€ 4,080,138	-€ 8,160,277	###########	###########
3191	0.17	########	########	€ 783,247	#########	-€ 6,312,897	###########	###########	###########
3192	0.13	########	########	€ 610,641	€ 814,187	-€ 4,921,703	-€ 9,843,405	###########	###########
3193	0.15	########	########	€ 726,060	€ 968,080	-€ 5,851,969	###########	###########	###########
3194	0.12	########	########	€ 572,865	€ 763,820	-€ 4,617,234	-€ 9,234,469	###########	###########
3195	0.13	########	########	€ 590,635	€ 787,513	-€ 4,760,459	-€ 9,520,917	###########	###########
3196	0.20	########	########	€ 928,123	#########	-€ 7,480,582	###########	###########	###########
3197	0.13	########	########	€ 606,462	€ 808,616	-€ 4,888,026	-€ 9,776,051	###########	###########
3198	0.17	########	########	€ 800,202	#########	-€ 6,449,548	###########	###########	###########
3199	0.12	########	########	€ 571,562	€ 762,083	-€ 4,606,736	-€ 9,213,472	###########	###########
3200	0.20	########	########	€ 935,250	#########	-€ 7,538,019	###########	###########	###########
3201	0.19	########	########	€ 902,174	#########	-€ 7,271,432	###########	###########	###########
3202	0.11	########	########	€ 503,035	€ 670,714	-€ 4,054,415	-€ 8,108,829	###########	###########
3203	0.09	########	########	€ 432,587	€ 576,783	-€ 3,486,609	-€ 6,973,217	###########	###########
3204	0.17	########	########	€ 786,990	#########	-€ 6,343,059	###########	###########	###########
3205	0.12	########	########	€ 585,741	€ 780,987	-€ 4,721,011	-€ 9,442,023	###########	###########
3206	0.24	########	########	#########	#########	-€ 8,961,252	###########	###########	###########
3207	0.14	########	########	€ 679,774	€ 906,365	-€ 5,478,911	###########	###########	###########
3208	0.11	########	########	€ 534,576	€ 712,768	-€ 4,308,629	-€ 8,617,258	###########	###########
3209	0.14	########	########	€ 646,240	€ 861,654	-€ 5,208,634	###########	###########	###########
3210	0.13	########	########	€ 633,363	€ 844,483	-€ 5,104,840	###########	###########	###########
3211	0.14	########	########	€ 669,054	€ 892,071	-€ 5,392,506	###########	###########	###########
3212	0.19	########	########	€ 889,865	#########	-€ 7,172,225	###########	###########	###########
3213	0.19	########	########	€ 911,877	#########	-€ 7,349,641	###########	###########	###########
3214	0.11	########	########	€ 541,646	€ 722,194	-€ 4,365,610	-€ 8,731,220	###########	###########
3215	0.18	########	########	€ 834,671	#########	-€ 6,727,366	###########	###########	###########
3216	0.15	########	########	€ 713,868	€ 951,824	-€ 5,753,704	###########	###########	###########
3217	0.20	########	########	€ 935,111	#########	-€ 7,536,900	###########	###########	###########
3218	0.13	########	########	€ 603,501	€ 804,668	-€ 4,864,160	-€ 9,728,320	###########	###########
3219	0.19	########	########	€ 916,888	#########	-€ 7,390,024	###########	###########	###########
3220	0.20	########	########	€ 922,327	#########	-€ 7,433,862	###########	###########	###########
3221	0.12	########	########	€ 560,922	€ 747,896	-€ 4,520,979	-€ 9,041,957	###########	###########
3222	0.14	########	########	€ 649,344	€ 865,792	-€ 5,233,647	###########	###########	###########
3223	0.22	########	########	#########	#########	-€ 8,308,599	###########	###########	###########
3224	0.12	########	########	€ 550,809	€ 734,412	-€ 4,439,466	-€ 8,878,931	###########	###########
3225	0.14	########	########	€ 675,174	€ 900,232	-€ 5,441,833	###########	###########	###########
3226	0.15	########	########	€ 695,245	€ 926,994	-€ 5,603,609	###########	###########	###########
3227	0.14	########	########	€ 668,606	€ 891,475	-€ 5,388,899	###########	###########	###########
3228	0.14	########	########	€ 663,135	€ 884,181	-€ 5,344,806	###########	###########	###########
3229	0.20	########	########	€ 963,409	#########	-€ 7,764,985	###########	###########	###########
3230	0.14	########	########	€ 661,295	€ 881,727	-€ 5,329,974	###########	###########	###########
3231	0.10	########	########	€ 459,115	€ 612,153	-€ 3,700,422	-€ 7,400,844	###########	###########
3232	0.14	########	########	€ 668,320	€ 891,093	-€ 5,386,594	###########	###########	###########
3233	0.11	########	########	€ 528,843	€ 705,123	-€ 4,262,418	-€ 8,524,837	###########	###########
3234	0.17	########	########	€ 779,520	#########	-€ 6,282,851	###########	###########	###########
3235	0.19	########	########	€ 918,214	#########	-€ 7,400,713	###########	###########	###########
3236	0.14	########	########	€ 642,297	€ 856,395	-€ 5,176,847	###########	###########	###########
3237	0.20	########	########	€ 964,568	#########	-€ 7,774,321	###########	###########	###########
3238	0.16	########	########	€ 765,862	#########	-€ 6,172,772	###########	###########	###########
3239	0.15	########	########	€ 703,142	€ 937,523	-€ 5,667,254	###########	###########	###########
3240	0.12	########	########	€ 587,470	€ 783,293	-€ 4,734,948	-€ 9,469,897	###########	###########
3241	0.12	########	########	€ 579,536	€ 772,715	-€ 4,671,004	-€ 9,342,008	###########	###########
3242	0.18	########	########	€ 860,057	#########	-€ 6,931,978	###########	###########	###########
3243	0.14	########	########	€ 652,137	€ 869,516	-€ 5,256,162	###########	###########	###########
3244	0.18	########	########	€ 867,595	#########	-€ 6,992,730	###########	###########	###########
3245	0.15	########	########	€ 718,299	€ 957,732	-€ 5,789,418	###########	###########	###########
3246	0.14	########	########	€ 677,873	€ 903,831	-€ 5,463,592	###########	###########	###########
3247	0.19	########	########	€ 888,512	#########	-€ 7,161,320	###########	###########	###########
3248	0.12	########	########	€ 589,291	€ 785,721	-€ 4,749,624	-€ 9,499,247	###########	###########
3249	0.20	########	########	€ 960,570	#########	-€ 7,742,099	###########	###########	###########
3250	0.13	########	########	€ 616,455	€ 821,940	-€ 4,968,563	-€ 9,937,127	###########	###########
3251	0.17	########	########	€ 810,114	#########	-€ 6,529,439	###########	###########	###########
3252	0.27	########	########	#########	#########	###########	###########	###########	###########
3253	0.18	########	########	€ 871,145	#########	-€ 7,021,342	###########	###########	###########
3254	0.18	########	########	€ 869,807	#########	-€ 7,010,560	###########	###########	###########
3255	0.19	########	########	€ 910,364	#########	-€ 7,337,444	###########	###########	###########
3256	0.13	########	########	€ 599,930	€ 799,907	-€ 4,835,380	-€ 9,670,760	###########	###########
3257	0.21	########	########	#########	#########	-€ 8,105,812	###########	###########	###########
3258	0.12	########	########	€ 543,634	€ 724,845	-€ 4,381,636	-€ 8,763,273	###########	###########
3259	0.17	########	########	€ 809,271	#########	-€ 6,522,642	###########	###########	###########
3260	0.15	########	########	€ 696,344	€ 928,459	-€ 5,612,467	###########	###########	###########
3261	0.18	########	########	€ 830,779	#########	-€ 6,695,999	###########	###########	###########
3262	0.14	########	########	€ 680,291	€ 907,054	-€ 5,483,075	###########	###########	###########
3263	0.18	########	########	€ 849,736	#########	-€ 6,848,787	###########	###########	###########
3264	0.18	########	########	€ 827,883	#########	-€ 6,672,657	###########	###########	###########

3265	0.13	########	########	€ 625,424	€ 833,898	-€ 5,040,853	###########	###########	###########
3266	0.10	########	########	€ 492,440	€ 656,587	-€ 3,969,018	-€ 7,938,036	###########	###########
3267	0.16	########	########	€ 756,747	#########	-€ 6,099,302	###########	###########	###########
3268	0.21	########	########	€ 987,554	#########	-€ 7,959,591	###########	###########	###########
3269	0.12	########	########	€ 579,329	€ 772,439	-€ 4,669,335	-€ 9,338,670	###########	###########
3270	0.20	########	########	€ 929,019	#########	-€ 7,487,805	###########	###########	###########
3271	0.10	########	########	€ 460,361	€ 613,815	-€ 3,710,467	-€ 7,420,933	###########	###########
3272	0.14	########	########	€ 668,543	€ 891,390	-€ 5,388,389	###########	###########	###########
3273	0.17	########	########	€ 786,375	#########	-€ 6,338,104	###########	###########	###########
3274	0.14	########	########	€ 684,066	€ 912,088	-€ 5,513,506	###########	###########	###########
3275	0.15	########	########	€ 699,142	€ 932,190	-€ 5,635,016	###########	###########	###########
3276	0.16	########	########	€ 739,192	€ 985,589	-€ 5,957,811	###########	###########	###########
3277	0.20	########	########	€ 935,831	#########	-€ 7,542,707	###########	###########	###########
3278	0.16	########	########	€ 743,716	€ 991,621	-€ 5,994,274	###########	###########	###########
3279	0.21	########	########	€ 969,488	#########	-€ 7,813,979	###########	###########	###########
3280	0.17	########	########	€ 811,831	#########	-€ 6,543,277	###########	###########	###########
3281	0.15	########	########	€ 687,692	€ 916,923	-€ 5,542,733	###########	###########	###########
3282	0.12	########	########	€ 555,713	€ 740,950	-€ 4,478,989	-€ 8,957,978	###########	###########
3283	0.15	########	########	€ 697,768	€ 930,357	-€ 5,623,942	###########	###########	###########
3284	0.18	########	########	€ 860,755	#########	-€ 6,937,604	###########	###########	###########
3285	0.14	########	########	€ 681,164	€ 908,219	-€ 5,490,116	###########	###########	###########
3286	0.14	########	########	€ 669,387	€ 892,516	-€ 5,395,190	###########	###########	###########
3287	0.12	########	########	€ 575,363	€ 767,151	-€ 4,637,372	-€ 9,274,744	###########	###########
3288	0.25	########	########	#########	#########	-€ 9,640,750	###########	###########	###########
3289	0.21	########	########	#########	#########	-€ 8,085,131	###########	###########	###########
3290	0.16	########	########	€ 762,365	#########	-€ 6,144,584	###########	###########	###########
3291	0.15	########	########	€ 697,694	€ 930,258	-€ 5,623,343	###########	###########	###########
3292	0.14	########	########	€ 660,069	€ 880,093	-€ 5,320,095	###########	###########	###########
3293	0.22	########	########	#########	#########	-€ 8,352,728	###########	###########	###########
3294	0.16	########	########	€ 751,824	#########	-€ 6,059,631	###########	###########	###########
3295	0.14	########	########	€ 642,625	€ 856,834	-€ 5,179,497	###########	###########	###########
3296	0.17	########	########	€ 815,229	#########	-€ 6,570,663	###########	###########	###########
3297	0.23	########	########	#########	#########	-€ 8,704,726	###########	###########	###########
3298	0.19	########	########	€ 890,188	#########	-€ 7,174,824	###########	###########	###########
3299	0.22	########	########	#########	#########	-€ 8,407,022	###########	###########	###########
3300	0.19	########	########	€ 882,222	#########	-€ 7,110,620	###########	###########	###########
3301	0.17	########	########	€ 812,244	#########	-€ 6,546,604	###########	###########	###########
3302	0.14	########	########	€ 644,542	€ 859,390	-€ 5,194,947	###########	###########	###########
3303	0.12	########	########	€ 589,819	€ 786,426	-€ 4,753,887	-€ 9,507,773	###########	###########
3304	0.17	########	########	€ 825,418	#########	-€ 6,652,787	###########	###########	###########
3305	0.17	########	########	€ 780,972	#########	-€ 6,294,556	###########	###########	###########
3306	0.15	########	########	€ 712,563	€ 950,084	-€ 5,743,190	###########	###########	###########
3307	0.20	########	########	€ 921,697	#########	-€ 7,428,789	###########	###########	###########
3308	0.14	########	########	€ 665,435	€ 887,247	-€ 5,363,342	###########	###########	###########
3309	0.17	########	########	€ 799,356	#########	-€ 6,442,734	###########	###########	###########
3310	0.15	########	########	€ 708,717	€ 944,956	-€ 5,712,189	###########	###########	###########
3311	0.21	########	########	€ 980,566	#########	-€ 7,903,268	###########	###########	###########
3312	0.16	########	########	€ 742,585	€ 990,113	-€ 5,985,161	###########	###########	###########
3313	0.17	########	########	€ 815,777	#########	-€ 6,575,085	###########	###########	###########
3314	0.20	########	########	€ 941,729	#########	-€ 7,590,242	###########	###########	###########
3315	0.13	########	########	€ 611,296	€ 815,061	-€ 4,926,981	-€ 9,853,963	###########	###########
3316	0.13	########	########	€ 597,961	€ 797,281	-€ 4,819,506	-€ 9,639,012	###########	###########
3317	0.09	########	########	€ 416,038	€ 554,717	-€ 3,353,221	-€ 6,706,443	###########	###########
3318	0.17	########	########	€ 805,146	#########	-€ 6,489,401	###########	###########	###########
3319	0.22	########	########	#########	#########	-€ 8,242,172	###########	###########	###########
3320	0.17	########	########	€ 801,255	#########	-€ 6,458,037	###########	###########	###########
3321	0.12	########	########	€ 548,709	€ 731,612	-€ 4,422,539	-€ 8,845,078	###########	###########
3322	0.14	########	########	€ 646,886	€ 862,515	-€ 5,213,838	###########	###########	###########
3323	0.13	########	########	€ 598,734	€ 798,312	-€ 4,825,736	-€ 9,651,472	###########	###########
3324	0.18	########	########	€ 830,625	#########	-€ 6,694,759	###########	###########	###########
3325	0.17	########	########	€ 813,752	#########	-€ 6,558,761	###########	###########	###########
3326	0.10	########	########	€ 478,245	€ 637,660	-€ 3,854,604	-€ 7,709,209	###########	###########
3327	0.22	########	########	#########	#########	-€ 8,505,570	###########	###########	###########
3328	0.12	########	########	€ 588,512	€ 784,683	-€ 4,743,351	-€ 9,486,703	###########	###########
3329	0.14	########	########	€ 683,105	€ 910,807	-€ 5,505,762	###########	###########	###########
3330	0.15	########	########	€ 720,057	€ 960,076	-€ 5,803,588	###########	###########	###########
3331	0.19	########	########	€ 887,830	#########	-€ 7,155,823	###########	###########	###########
3332	0.25	########	########	#########	#########	-€ 9,594,352	###########	###########	###########
3333	0.13	########	########	€ 625,692	€ 834,256	-€ 5,043,016	###########	###########	###########
3334	0.13	########	########	€ 590,797	€ 787,730	-€ 4,761,767	-€ 9,523,534	###########	###########
3335	0.17	########	########	€ 805,311	#########	-€ 6,490,730	###########	###########	###########
3336	0.10	########	########	€ 465,881	€ 621,175	-€ 3,754,955	-€ 7,509,911	###########	###########
3337	0.15	########	########	€ 699,591	€ 932,789	-€ 5,638,638	###########	###########	###########
3338	0.14	########	########	€ 674,628	€ 899,504	-€ 5,437,437	###########	###########	###########
3339	0.07	########	########	€ 314,626	€ 419,501	-€ 2,535,851	-€ 5,071,702	-€ 7,607,553	###########
3340	0.22	########	########	#########	#########	-€ 8,565,464	###########	###########	###########
3341	0.15	########	########	€ 724,594	€ 966,126	-€ 5,840,158	###########	###########	###########
3342	0.14	########	########	€ 660,262	€ 880,349	-€ 5,321,644	###########	###########	###########
3343	0.17	########	########	€ 806,220	#########	-€ 6,498,053	###########	###########	###########
3344	0.17	########	########	€ 817,942	#########	-€ 6,592,529	###########	###########	###########
3345	0.17	########	########	€ 814,702	#########	-€ 6,566,414	###########	###########	###########
3346	0.17	########	########	€ 802,984	#########	-€ 6,471,975	###########	###########	###########
3347	0.14	########	########	€ 659,920	€ 879,893	-€ 5,318,888	###########	###########	###########
3348	0.08	########	########	€ 362,744	€ 483,659	-€ 2,923,681	-€ 5,847,363	-€ 8,771,044	###########
3349	0.19	########	########	€ 912,760	#########	-€ 7,356,753	###########	###########	###########
3350	0.10	########	########	€ 485,796	€ 647,728	-€ 3,915,466	-€ 7,830,932	###########	###########
3351	0.12	########	########	€ 565,196	€ 753,595	-€ 4,555,426	-€ 9,110,852	###########	###########
3352	0.15	########	########	€ 731,599	€ 975,465	-€ 5,896,616	###########	###########	###########
3353	0.12	########	########	€ 573,374	€ 764,498	-€ 4,621,335	-€ 9,242,670	###########	###########
3354	0.13	########	########	€ 622,195	€ 829,594	-€ 5,014,833	###########	###########	###########
3355	0.13	########	########	€ 602,103	€ 802,804	-€ 4,852,888	-€ 9,705,776	###########	###########
3356	0.23	########	########	#########	#########	-€ 8,709,650	###########	###########	###########
3357	0.10	########	########	€ 482,201	€ 642,934	-€ 3,886,490	-€ 7,772,980	###########	###########
3358	0.16	########	########	€ 761,784	#########	-€ 6,139,906	###########	###########	###########
3359	0.11	########	########	€ 516,687	€ 688,915	-€ 4,164,443	-€ 8,328,886	###########	###########
3360	0.17	########	########	€ 784,176	#########	-€ 6,320,378	###########	###########	###########
3361	0.15	########	########	€ 711,947	€ 949,263	-€ 5,738,225	###########	###########	###########
3362	0.15	########	########	€ 725,988	€ 967,983	-€ 5,851,388	###########	###########	###########
3363	0.19	########	########	€ 893,793	#########	-€ 7,203,880	###########	###########	###########
3364	0.16	########	########	€ 765,341	#########	-€ 6,168,573	###########	###########	###########
3365	0.20	########	########	€ 957,258	#########	-€ 7,715,403	###########	###########	###########
3366	0.15	########	########	€ 716,408	€ 955,210	-€ 5,774,175	###########	###########	###########
3367	0.18	########	########	€ 849,230	#########	-€ 6,844,708	###########	###########	###########
3368	0.17	########	########	€ 819,017	#########	-€ 6,601,199	###########	###########	###########
3369	0.18	########	########	€ 872,814	#########	-€ 7,034,795	###########	###########	###########
3370	0.18	########	########	€ 865,433	#########	-€ 6,975,307	###########	###########	###########
3371	0.15	########	########	€ 707,681	€ 943,575	-€ 5,703,842	###########	###########	###########
3372	0.21	########	########	#########	#########	-€ 8,147,003	###########	###########	###########
3373	0.17	########	########	€ 787,531	#########	-€ 6,347,421	###########	###########	###########
3374	0.14	########	########	€ 670,820	€ 894,426	-€ 5,406,742	###########	###########	###########
3375	0.16	########	########	€ 732,258	€ 976,343	-€ 5,901,923	###########	###########	###########
3376	0.15	########	########	€ 715,420	€ 953,893	-€ 5,766,215	###########	###########	###########
3377	0.15	########	########	€ 686,935	€ 915,913	-€ 5,536,628	###########	###########	###########
3378	0.15	########	########	€ 694,135	€ 925,514	-€ 5,594,662	###########	###########	###########
3379	0.21	########	########	€ 999,232	#########	-€ 8,053,711	###########	###########	###########
3380	0.14	########	########	€ 648,487	€ 864,649	-€ 5,226,742	###########	###########	###########
3381	0.12	########	########	€ 555,583	€ 740,778	-€ 4,477,946	-€ 8,955,893	###########	###########
3382	0.17	########	########	€ 781,137	#########	-€ 6,295,885	###########	###########	###########
3383	0.20	########	########	€ 954,931	#########	-€ 7,696,649	###########	###########	###########
3384	0.24	########	########	#########	#########	-€ 9,003,514	###########	###########	###########
3385	0.14	########	########	€ 672,885	€ 897,179	-€ 5,423,383	###########	###########	###########
3386	0.17	########	########	€ 782,621	#########	-€ 6,307,852	###########	###########	###########
3387	0.11	########	########	€ 522,653	€ 696,870	-€ 4,212,529	-€ 8,425,058	###########	###########
3388	0.16	########	########	€ 733,101	€ 977,468	-€ 5,908,719	###########	###########	###########
3389	0.18	########	########	€ 827,348	#########	-€ 6,668,345	###########	###########	###########
3390	0.10	########	########	€ 495,504	€ 660,672	-€ 3,993,716	-€ 7,987,431	###########	###########
3391	0.15	########	########	€ 731,274	€ 975,032	-€ 5,893,999	###########	###########	###########
3392	0.10	########	########	€ 461,667	€ 615,556	-€ 3,720,990	-€ 7,441,979	###########	###########
3393	0.14	########	########	€ 653,815	€ 871,753	-€ 5,269,684	###########	###########	###########
3394	0.16	########	########	€ 779,140	#########	-€ 6,279,791	###########	###########	###########
3395	0.23	########	########	#########	#########	-€ 8,780,202	###########	###########	###########
3396	0.22	########	########	#########	#########	-€ 8,378,094	###########	###########	###########
3397	0.19	########	########	€ 896,348	#########	-€ 7,224,478	###########	###########	###########
3398	0.17	########	########	€ 818,084	#########	-€ 6,593,679	###########	###########	###########
3399	0.18	########	########	€ 840,583	#########	-€ 6,775,018	###########	###########	###########
3400	0.11	########	########	€ 529,109	€ 705,479	-€ 4,264,565	-€ 8,529,131	###########	###########
3401	0.19	########	########	€ 882,097	#########	-€ 7,109,614	###########	###########	###########
3402	0.15	########	########	€ 704,169	€ 938,892	-€ 5,675,531	###########	###########	###########
3403	0.14	########	########	€ 654,172	€ 872,230	-€ 5,272,564	###########	###########	###########
3404	0.12	########	########	€ 559,382	€ 745,842	-€ 4,508,561	-€ 9,017,122	###########	###########
3405	0.12	########	########	€ 563,637	€ 751,516	-€ 4,542,860	-€ 9,085,720	###########	###########
3406	0.15	########	########	€ 685,990	€ 914,654	-€ 5,529,013	###########	###########	###########
3407	0.18	########	########	€ 868,370	#########	-€ 6,998,974	###########	###########	###########
3408	0.15	########	########	€ 709,813	€ 946,417	-€ 5,721,019	###########	###########	###########
3409	0.18	########	########	€ 857,472	#########	-€ 6,911,142	###########	###########	###########
3410	0.17	########	########	€ 819,831	#########	-€ 6,607,758	###########	###########	###########
3411	0.18	########	########	€ 851,686	#########	-€ 6,864,509	###########	###########	###########
3412	0.20	########	########	€ 941,410	#########	-€ 7,587,674	###########	###########	###########
3413	0.21	########	########	€ 996,145	#########	-€ 8,028,833	###########	###########	###########
3414	0.16	########	########	€ 754,328	#########	-€ 6,079,808	###########	###########	###########
3415	0.14	########	########	€ 656,067	€ 874,756	-€ 5,287,837	###########	###########	###########
3416	0.11	########	########	€ 518,661	€ 691,548	-€ 4,180,357	-€ 8,360,715	###########	###########
3417	0.18	########	########	€ 849,332	#########	-€ 6,845,534	###########	###########	###########
3418	0.20	########	########	€ 948,108	#########	-€ 7,641,659	###########	###########	###########
3419	0.15	########	########	€ 715,296	€ 953,728	-€ 5,765,214	###########	###########	###########
3420	0.21	########	########	€ 972,250	#########	-€ 7,836,237	###########	###########	###########
3421	0.12	########	########	€ 589,756	€ 786,342	-€ 4,753,378	-€ 9,506,757	###########	###########
3422	0.18	########	########	€ 855,723	#########	-€ 6,897,044	###########	###########	###########
3423	0.16	########	########	€ 773,896	#########	-€ 6,237,528	###########	###########	###########
3424	0.15	########	########	€ 696,369	€ 928,492	-€ 5,612,663	###########	###########	###########
3425	0.12	########	########	€ 581,962	€ 775,949	-€ 4,690,556	-€ 9,381,112	###########	###########
3426	0.14	########	########	€ 684,781	€ 913,041	-€ 5,519,264	###########	###########	###########
3427	0.14	########	########	€ 679,398	€ 905,864	-€ 5,475,883	###########	###########	###########
3428	0.11	########	########	€ 510,752	€ 681,002	-€ 4,116,609	-€ 8,233,219	###########	###########
3429	0.19	########	########	€ 882,064	#########	-€ 7,109,351	###########	###########	###########
3430	0.10	########	########	€ 477,562	€ 636,749	-€ 3,849,102	-€ 7,698,204	###########	###########
3431	0.19	########	########	€ 907,040	#########	-€ 7,310,652	###########	###########	###########
3432	0.15	########	########	€ 713,935	€ 951,913	-€ 5,754,244	###########	###########	###########
3433	0.17	########	########	€ 792,993	#########	-€ 6,391,446	###########	###########	###########
3434	0.16	########	########	€ 732,696	€ 976,929	-€ 5,905,461	###########	###########	###########
3435	0.13	########	########	€ 624,180	€ 832,240	-€ 5,030,832	###########	###########	###########
3436	0.11	########	########	€ 498,221	€ 664,294	-€ 4,015,608	-€ 8,031,217	###########	###########

3437	0.17	########	########	€ 787,056	########	-€ 6,343,593	########	########	########
3438	0.09	########	########	€ 445,833	€ 594,443	-€ 3,593,366	-€ 7,186,733	########	########
3439	0.12	########	########	€ 547,218	€ 729,624	-€ 4,410,525	-€ 8,821,051	########	########
3440	0.12	########	########	€ 547,795	€ 730,393	-€ 4,415,171	-€ 8,830,342	########	########
3441	0.16	########	########	€ 758,428	########	-€ 6,112,852	########	########	########
3442	0.19	########	########	€ 902,017	########	-€ 7,270,171	########	########	########
3443	0.13	########	########	€ 600,636	€ 800,848	-€ 4,841,066	-€ 9,682,133	########	########
3444	0.19	########	########	€ 912,256	########	-€ 7,352,697	########	########	########
3445	0.16	########	########	€ 758,564	########	-€ 6,113,951	########	########	########
3446	0.15	########	########	€ 696,110	€ 928,147	-€ 5,610,577	########	########	########
3447	0.11	########	########	€ 520,137	€ 693,516	-€ 4,192,255	-€ 8,384,509	########	########
3448	0.18	########	########	€ 866,649	########	-€ 6,985,104	########	########	########
3449	0.19	########	########	€ 885,623	########	-€ 7,138,031	########	########	########
3450	0.21	########	########	€ 971,545	########	-€ 7,830,556	########	########	########
3451	0.27	########	########	########	########	########	########	########	########
3452	0.16	########	########	€ 734,933	€ 979,910	-€ 5,923,484	########	########	########
3453	0.12	########	########	€ 585,143	€ 780,191	-€ 4,716,198	-€ 9,432,397	########	########
3454	0.17	########	########	€ 787,932	########	-€ 6,350,656	########	########	########
3455	0.20	########	########	€ 929,271	########	-€ 7,489,834	########	########	########
3456	0.14	########	########	€ 676,402	€ 901,869	-€ 5,451,730	########	########	########
3457	0.12	########	########	€ 578,810	€ 771,746	-€ 4,665,148	-€ 9,330,296	########	########
3458	0.13	########	########	€ 613,339	€ 817,786	-€ 4,943,454	-€ 9,886,908	########	########
3459	0.16	########	########	€ 749,901	€ 999,867	-€ 6,044,124	########	########	########
3460	0.19	########	########	€ 907,628	########	-€ 7,315,395	########	########	########
3461	0.16	########	########	€ 766,183	########	-€ 6,175,359	########	########	########
3462	0.27	########	########	########	########	########	########	########	########
3463	0.22	########	########	########	########	-€ 8,269,840	########	########	########
3464	0.19	########	########	€ 902,710	########	-€ 7,275,755	########	########	########
3465	0.16	########	########	€ 735,759	€ 981,013	-€ 5,930,148	########	########	########
3466	0.14	########	########	€ 682,223	€ 909,630	-€ 5,498,649	########	########	########
3467	0.17	########	########	€ 790,897	########	-€ 6,374,552	########	########	########
3468	0.18	########	########	€ 871,325	########	-€ 7,022,797	########	########	########
3469	0.22	########	########	########	########	-€ 8,414,863	########	########	########
3470	0.21	########	########	€ 980,890	########	-€ 7,905,873	########	########	########
3471	0.16	########	########	€ 769,606	########	-€ 6,202,947	########	########	########
3472	0.18	########	########	€ 864,116	########	-€ 6,964,686	########	########	########
3473	0.14	########	########	€ 679,616	€ 906,155	-€ 5,477,640	########	########	########
3474	0.12	########	########	€ 575,218	€ 766,958	-€ 4,636,202	-€ 9,272,405	########	########
3475	0.15	########	########	€ 718,244	€ 957,658	-€ 5,788,972	########	########	########
3476	0.13	########	########	€ 599,220	€ 798,961	-€ 4,829,658	-€ 9,659,316	########	########
3477	0.21	########	########	########	########	-€ 8,136,851	########	########	########
3478	0.19	########	########	€ 895,244	########	-€ 7,215,582	########	########	########
3479	0.16	########	########	€ 776,462	########	-€ 6,258,210	########	########	########
3480	0.11	########	########	€ 529,205	€ 705,606	-€ 4,265,338	-€ 8,530,676	########	########
3481	0.15	########	########	€ 717,871	€ 957,161	-€ 5,785,969	########	########	########
3482	0.12	########	########	€ 565,270	€ 753,694	-€ 4,556,023	-€ 9,112,046	########	########
3483	0.23	########	########	########	########	-€ 8,688,944	########	########	########
3484	0.17	########	########	€ 786,158	########	-€ 6,336,357	########	########	########
3485	0.08	########	########	€ 369,789	€ 493,052	-€ 2,980,466	-€ 5,960,931	-€ 8,941,397	########
3486	0.18	########	########	€ 834,830	########	-€ 6,728,650	########	########	########
3487	0.15	########	########	€ 729,456	€ 972,608	-€ 5,879,344	########	########	########
3488	0.17	########	########	€ 806,093	########	-€ 6,497,027	########	########	########
3489	0.19	########	########	€ 897,966	########	-€ 7,237,520	########	########	########
3490	0.15	########	########	€ 711,385	€ 948,513	-€ 5,733,691	########	########	########
3491	0.08	########	########	€ 400,444	€ 533,926	-€ 3,227,540	-€ 6,455,080	-€ 9,682,621	########
3492	0.23	########	########	########	########	-€ 8,779,158	########	########	########
3493	0.17	########	########	€ 796,531	########	-€ 6,419,964	########	########	########
3494	0.14	########	########	€ 640,231	€ 853,641	-€ 5,160,196	########	########	########
3495	0.12	########	########	€ 575,678	€ 767,571	-€ 4,639,910	-€ 9,279,820	########	########
3496	0.21	########	########	€ 989,917	########	-€ 7,978,635	########	########	########
3497	0.16	########	########	€ 771,561	########	-€ 6,218,705	########	########	########
3498	0.17	########	########	€ 789,283	########	-€ 6,361,544	########	########	########
3499	0.18	########	########	€ 849,712	########	-€ 6,848,597	########	########	########
3500	0.19	########	########	€ 875,541	########	-€ 7,056,771	########	########	########
3501	0.14	########	########	€ 658,157	€ 877,543	-€ 5,304,684	########	########	########
3502	0.15	########	########	€ 701,573	€ 935,431	-€ 5,654,613	########	########	########
3503	0.19	########	########	€ 894,006	########	-€ 7,205,597	########	########	########
3504	0.12	########	########	€ 589,071	€ 785,428	-€ 4,747,852	-€ 9,495,704	########	########
3505	0.19	########	########	€ 897,437	########	-€ 7,233,253	########	########	########
3506	0.21	########	########	€ 979,573	########	-€ 7,895,260	########	########	########
3507	0.15	########	########	€ 728,126	€ 970,835	-€ 5,868,627	########	########	########
3508	0.16	########	########	€ 753,328	########	-€ 6,071,750	########	########	########
3509	0.14	########	########	€ 659,334	€ 879,112	-€ 5,314,167	########	########	########
3510	0.18	########	########	€ 841,983	########	-€ 6,786,300	########	########	########
3511	0.23	########	########	########	########	-€ 8,767,459	########	########	########
3512	0.15	########	########	€ 727,883	€ 970,510	-€ 5,866,663	########	########	########
3513	0.12	########	########	€ 588,935	€ 785,247	-€ 4,746,760	-€ 9,493,521	########	########
3514	0.18	########	########	€ 839,780	########	-€ 6,768,546	########	########	########
3515	0.17	########	########	€ 805,920	########	-€ 6,495,638	########	########	########
3516	0.15	########	########	€ 731,428	€ 975,238	-€ 5,895,239	########	########	########
3517	0.13	########	########	€ 628,615	€ 838,153	-€ 5,066,573	########	########	########
3518	0.14	########	########	€ 666,786	€ 889,049	-€ 5,374,233	########	########	########
3519	0.16	########	########	€ 733,571	€ 978,094	-€ 5,912,506	########	########	########
3520	0.20	########	########	€ 957,268	########	-€ 7,715,485	########	########	########
3521	0.13	########	########	€ 602,821	€ 803,762	-€ 4,858,681	-€ 9,717,363	########	########
3522	0.12	########	########	€ 578,871	€ 771,829	-€ 4,665,646	-€ 9,331,292	########	########
3523	0.16	########	########	€ 749,847	€ 999,796	-€ 6,043,693	########	########	########
3524	0.20	########	########	€ 938,050	########	-€ 7,560,591	########	########	########
3525	0.10	########	########	€ 484,008	€ 645,344	-€ 3,901,057	-€ 7,802,115	########	########
3526	0.16	########	########	€ 743,213	€ 990,951	-€ 5,990,226	########	########	########
3527	0.16	########	########	€ 779,181	########	-€ 6,280,123	########	########	########
3528	0.13	########	########	€ 606,139	€ 808,185	-€ 4,885,419	-€ 9,770,839	########	########
3529	0.18	########	########	€ 870,315	########	-€ 7,014,649	########	########	########
3530	0.09	########	########	€ 417,324	€ 556,433	-€ 3,363,594	-€ 6,727,188	########	########
3531	0.27	########	########	########	########	########	########	########	########
3532	0.12	########	########	€ 551,674	€ 735,565	-€ 4,446,436	-€ 8,892,873	########	########
3533	0.17	########	########	€ 796,089	########	-€ 6,416,395	########	########	########
3534	0.17	########	########	€ 781,663	########	-€ 6,300,126	########	########	########
3535	0.12	########	########	€ 581,896	€ 775,861	-€ 4,690,024	-€ 9,380,047	########	########
3536	0.15	########	########	€ 710,787	€ 947,716	-€ 5,728,875	########	########	########
3537	0.22	########	########	########	########	-€ 8,196,736	########	########	########
3538	0.08	########	########	€ 356,983	€ 475,977	-€ 2,877,247	-€ 5,754,494	-€ 8,631,741	########
3539	0.10	########	########	€ 479,785	€ 639,713	-€ 3,867,016	-€ 7,734,031	########	########
3540	0.16	########	########	€ 768,424	########	-€ 6,193,418	########	########	########
3541	0.21	########	########	########	########	-€ 8,096,638	########	########	########
3542	0.10	########	########	€ 457,567	€ 610,089	-€ 3,687,943	-€ 7,375,887	########	########
3543	0.24	########	########	########	########	-€ 9,176,168	########	########	########
3544	0.19	########	########	€ 917,856	########	-€ 7,397,830	########	########	########
3545	0.17	########	########	€ 790,308	########	-€ 6,369,808	########	########	########
3546	0.10	########	########	€ 463,066	€ 617,422	-€ 3,732,269	-€ 7,464,539	########	########
3547	0.12	########	########	€ 554,200	€ 738,934	-€ 4,466,800	-€ 8,933,600	########	########
3548	0.15	########	########	€ 720,818	€ 961,090	-€ 5,809,719	########	########	########
3549	0.17	########	########	€ 824,309	########	-€ 6,643,852	########	########	########
3550	0.10	########	########	€ 473,098	€ 630,797	-€ 3,813,123	-€ 7,626,246	########	########
3551	0.23	########	########	########	########	-€ 8,929,008	########	########	########
3552	0.16	########	########	€ 771,530	########	-€ 6,218,455	########	########	########
3553	0.17	########	########	€ 796,758	########	-€ 6,421,795	########	########	########
3554	0.17	########	########	€ 801,274	########	-€ 6,458,192	########	########	########
3555	0.14	########	########	€ 641,372	€ 855,163	-€ 5,169,399	########	########	########
3556	0.17	########	########	€ 798,364	########	-€ 6,434,732	########	########	########
3557	0.14	########	########	€ 651,316	€ 868,421	-€ 5,249,540	########	########	########
3558	0.18	########	########	€ 862,794	########	-€ 6,954,038	########	########	########
3559	0.17	########	########	€ 791,472	########	-€ 6,379,188	########	########	########
3560	0.09	########	########	€ 420,323	€ 560,431	-€ 3,387,764	-€ 6,775,527	########	########
3561	0.08	########	########	€ 385,345	€ 513,794	-€ 3,105,844	-€ 6,211,687	-€ 9,317,531	########
3562	0.14	########	########	€ 660,837	€ 881,116	-€ 5,326,282	########	########	########
3563	0.16	########	########	€ 753,656	########	-€ 6,074,390	########	########	########
3564	0.19	########	########	€ 915,166	########	-€ 7,376,151	########	########	########
3565	0.20	########	########	€ 954,428	########	-€ 7,692,598	########	########	########
3566	0.22	########	########	########	########	-€ 8,255,861	########	########	########
3567	0.23	########	########	########	########	-€ 8,907,587	########	########	########
3568	0.16	########	########	€ 746,702	€ 995,603	-€ 6,018,346	########	########	########
3569	0.11	########	########	€ 517,714	€ 690,285	-€ 4,172,723	-€ 8,345,446	########	########
3570	0.17	########	########	€ 802,663	########	-€ 6,469,381	########	########	########
3571	0.18	########	########	€ 841,317	########	-€ 6,780,934	########	########	########
3572	0.12	########	########	€ 553,902	€ 738,535	-€ 4,464,392	-€ 8,928,784	########	########
3573	0.18	########	########	€ 827,165	########	-€ 6,666,866	########	########	########
3574	0.15	########	########	€ 706,058	€ 941,410	-€ 5,690,755	########	########	########
3575	0.16	########	########	€ 750,067	########	-€ 6,045,463	########	########	########
3576	0.19	########	########	€ 881,502	########	-€ 7,104,816	########	########	########
3577	0.21	########	########	########	########	-€ 8,151,894	########	########	########
3578	0.16	########	########	€ 778,998	########	-€ 6,278,650	########	########	########
3579	0.18	########	########	€ 833,221	########	-€ 6,715,682	########	########	########
3580	0.14	########	########	€ 683,336	€ 911,114	-€ 5,507,617	########	########	########
3581	0.15	########	########	€ 721,567	€ 962,090	-€ 5,815,760	########	########	########
3582	0.11	########	########	€ 511,801	€ 682,401	-€ 4,125,062	-€ 8,250,123	########	########
3583	0.21	########	########	########	########	-€ 8,085,758	########	########	########
3584	0.14	########	########	€ 666,977	€ 889,303	-€ 5,375,769	########	########	########
3585	0.08	########	########	€ 377,719	€ 503,626	-€ 3,044,379	-€ 6,088,758	-€ 9,133,136	########
3586	0.12	########	########	€ 576,040	€ 768,053	-€ 4,642,823	-€ 9,285,646	########	########
3587	0.12	########	########	€ 563,938	€ 751,918	-€ 4,545,287	-€ 9,090,575	########	########
3588	0.12	########	########	€ 559,597	€ 746,129	-€ 4,510,295	-€ 9,020,590	########	########
3589	0.14	########	########	€ 671,832	€ 895,776	-€ 5,414,900	########	########	########
3590	0.14	########	########	€ 662,549	€ 883,399	-€ 5,340,079	########	########	########
3591	0.21	########	########	########	########	-€ 8,065,147	########	########	########
3592	0.14	########	########	€ 680,391	€ 907,188	-€ 5,483,883	########	########	########
3593	0.15	########	########	€ 696,453	€ 928,604	-€ 5,613,341	########	########	########
3594	0.18	########	########	€ 849,329	########	-€ 6,845,508	########	########	########
3595	0.07	########	########	€ 333,260	€ 444,347	-€ 2,686,045	-€ 5,372,090	-€ 8,058,135	########
3596	0.17	########	########	€ 788,494	########	-€ 6,355,185	########	########	########
3597	0.17	########	########	€ 821,313	########	-€ 6,619,705	########	########	########
3598	0.21	########	########	€ 980,894	########	-€ 7,905,906	########	########	########
3599	0.21	########	########	€ 990,963	########	-€ 7,987,060	########	########	########
3600	0.17	########	########	€ 790,449	########	-€ 6,370,941	########	########	########
3601	0.17	########	########	€ 823,386	########	-€ 6,636,412	########	########	########
3602	0.11	########	########	€ 533,441	€ 711,254	-€ 4,299,478	-€ 8,598,956	########	########
3603	0.15	########	########	€ 711,058	€ 948,078	-€ 5,731,059	########	########	########
3604	0.23	########	########	########	########	-€ 8,896,579	########	########	########
3605	0.17	########	########	€ 824,081	########	-€ 6,642,013	########	########	########
3606	0.12	########	########	€ 584,076	€ 778,768	-€ 4,707,593	-€ 9,415,186	########	########
3607	0.14	########	########	€ 644,620	€ 859,494	-€ 5,195,576	########	########	########
3608	0.18	########	########	€ 865,291	########	-€ 6,974,157	########	########	########

3609	0.16	########	########	€ 747,296	€ 996,395	-€ 6,023,132	###########	###########	###########
3610	0.15	########	########	€ 705,836	€ 941,115	-€ 5,688,971	###########	###########	###########
3611	0.15	########	########	€ 701,253	€ 935,004	-€ 5,652,030	###########	###########	###########
3612	0.18	########	########	€ 869,999	#########	-€ 7,012,106	###########	###########	###########
3613	0.19	########	########	€ 899,966	#########	-€ 7,253,633	###########	###########	###########
3614	0.18	########	########	€ 865,089	#########	-€ 6,972,529	###########	###########	###########
3615	0.23	########	########	#########	#########	-€ 8,595,601	###########	###########	###########
3616	0.19	########	########	€ 898,073	#########	-€ 7,238,377	###########	###########	###########
3617	0.21	########	########	€ 983,659	#########	-€ 7,928,197	###########	###########	###########
3618	0.16	########	########	€ 739,896	€ 986,528	-€ 5,963,486	###########	###########	###########
3619	0.15	########	########	€ 691,230	€ 921,640	-€ 5,571,246	###########	###########	###########
3620	0.12	########	########	€ 578,430	€ 771,240	-€ 4,662,091	-€ 9,324,183	###########	###########
3621	0.18	########	########	€ 839,515	#########	-€ 6,766,408	###########	###########	###########
3622	0.15	########	########	€ 689,362	€ 919,149	-€ 5,556,186	###########	###########	###########
3623	0.14	########	########	€ 680,420	€ 907,227	-€ 5,484,121	###########	###########	###########
3624	0.10	########	########	€ 471,750	€ 629,000	-€ 3,802,261	-€ 7,604,522	###########	###########
3625	0.12	########	########	€ 570,414	€ 760,552	-€ 4,597,479	-€ 9,194,958	###########	###########
3626	0.16	########	########	€ 737,219	€ 982,959	-€ 5,941,914	###########	###########	###########
3627	0.21	########	########	€ 983,687	#########	-€ 7,928,421	###########	###########	###########
3628	0.16	########	########	€ 732,620	€ 976,827	-€ 5,904,849	###########	###########	###########
3629	0.17	########	########	€ 817,579	#########	-€ 6,589,608	###########	###########	###########
3630	0.12	########	########	€ 552,652	€ 736,869	-€ 4,454,318	-€ 8,908,636	###########	###########
3631	0.15	########	########	€ 684,950	€ 913,267	-€ 5,520,631	###########	###########	###########
3632	0.11	########	########	€ 514,931	€ 686,575	-€ 4,150,297	-€ 8,300,593	###########	###########
3633	0.13	########	########	€ 616,048	€ 821,398	-€ 4,965,289	-€ 9,930,578	###########	###########
3634	0.17	########	########	€ 813,412	#########	-€ 6,556,024	###########	###########	###########
3635	0.15	########	########	€ 717,782	€ 957,043	-€ 5,785,255	###########	###########	###########
3636	0.14	########	########	€ 665,789	€ 887,719	-€ 5,366,195	###########	###########	###########
3637	0.17	########	########	€ 793,962	#########	-€ 6,399,257	###########	###########	###########
3638	0.21	########	########	#########	#########	-€ 8,116,148	###########	###########	###########
3639	0.14	########	########	€ 647,881	€ 863,842	-€ 5,221,858	###########	###########	###########
3640	0.12	########	########	€ 557,718	€ 743,624	-€ 4,495,152	-€ 8,990,304	###########	###########
3641	0.17	########	########	€ 796,995	#########	-€ 6,423,702	###########	###########	###########
3642	0.15	########	########	€ 705,094	€ 940,125	-€ 5,682,986	###########	###########	###########
3643	0.14	########	########	€ 666,477	€ 888,636	-€ 5,371,738	###########	###########	###########
3644	0.13	########	########	€ 595,935	€ 794,580	-€ 4,803,178	-€ 9,606,355	###########	###########
3645	0.19	########	########	€ 881,747	#########	-€ 7,106,793	###########	###########	###########
3646	0.19	########	########	€ 884,740	#########	-€ 7,130,919	###########	###########	###########
3647	0.15	########	########	€ 690,434	€ 920,579	-€ 5,564,834	###########	###########	###########
3648	0.12	########	########	€ 556,979	€ 742,639	-€ 4,489,196	-€ 8,978,391	###########	###########
3649	0.25	########	########	#########	#########	-€ 9,408,388	###########	###########	###########
3650	0.18	########	########	€ 867,248	#########	-€ 6,989,934	###########	###########	###########
3651	0.19	########	########	€ 897,231	#########	-€ 7,231,589	###########	###########	###########
3652	0.21	########	########	€ 986,771	#########	-€ 7,953,280	###########	###########	###########
3653	0.13	########	########	€ 599,700	€ 799,600	-€ 4,833,522	-€ 9,667,044	###########	###########
3654	0.16	########	########	€ 757,947	#########	-€ 6,108,981	###########	###########	###########
3655	0.17	########	########	€ 787,286	#########	-€ 6,345,443	###########	###########	###########
3656	0.19	########	########	€ 877,034	#########	-€ 7,068,803	###########	###########	###########
3657	0.12	########	########	€ 568,327	€ 757,770	-€ 4,580,662	-€ 9,161,324	###########	###########
3658	0.13	########	########	€ 596,978	€ 795,970	-€ 4,811,582	-€ 9,623,164	###########	###########
3659	0.15	########	########	€ 685,625	€ 914,167	-€ 5,526,073	###########	###########	###########
3660	0.13	########	########	€ 629,541	€ 839,388	-€ 5,074,039	###########	###########	###########
3661	0.10	########	########	€ 470,669	€ 627,558	-€ 3,793,543	-€ 7,587,085	###########	###########
3662	0.21	########	########	#########	#########	-€ 8,098,670	###########	###########	###########
3663	0.11	########	########	€ 518,020	€ 690,693	-€ 4,175,191	-€ 8,350,381	###########	###########
3664	0.20	########	########	€ 928,323	#########	-€ 7,482,191	###########	###########	###########
3665	0.16	########	########	€ 735,996	€ 981,328	-€ 5,932,054	###########	###########	###########
3666	0.09	########	########	€ 427,597	€ 570,129	-€ 3,446,389	-€ 6,892,778	###########	###########
3667	0.16	########	########	€ 777,728	#########	-€ 6,268,413	###########	###########	###########
3668	0.20	########	########	€ 924,290	#########	-€ 7,449,687	###########	###########	###########
3669	0.16	########	########	€ 750,432	#########	-€ 6,048,411	###########	###########	###########
3670	0.17	########	########	€ 779,833	#########	-€ 6,285,377	###########	###########	###########
3671	0.14	########	########	€ 639,166	€ 852,222	-€ 5,151,616	###########	###########	###########
3672	0.15	########	########	€ 712,587	€ 950,116	-€ 5,743,382	###########	###########	###########
3673	0.15	########	########	€ 723,270	€ 964,359	-€ 5,829,481	###########	###########	###########
3674	0.18	########	########	€ 870,750	#########	-€ 7,018,162	###########	###########	###########
3675	0.09	########	########	€ 438,628	€ 584,837	-€ 3,535,299	-€ 7,070,598	###########	###########
3676	0.12	########	########	€ 567,099	€ 756,132	-€ 4,570,759	-€ 9,141,518	###########	###########
3677	0.15	########	########	€ 731,269	€ 975,025	-€ 5,893,953	###########	###########	###########
3678	0.15	########	########	€ 722,883	€ 963,844	-€ 5,826,366	###########	###########	###########
3679	0.21	########	########	#########	#########	-€ 8,128,762	###########	###########	###########
3680	0.21	########	########	€ 994,144	#########	-€ 8,012,705	###########	###########	###########
3681	0.11	########	########	€ 518,973	€ 691,964	-€ 4,182,872	-€ 8,365,745	###########	###########
3682	0.22	########	########	#########	#########	-€ 8,255,466	###########	###########	###########
3683	0.19	########	########	€ 910,674	#########	-€ 7,339,940	###########	###########	###########
3684	0.17	########	########	€ 817,881	#########	-€ 6,592,041	###########	###########	###########
3685	0.19	########	########	€ 881,628	#########	-€ 7,105,838	###########	###########	###########
3686	0.21	########	########	€ 979,207	#########	-€ 7,892,309	###########	###########	###########
3687	0.13	########	########	€ 615,534	€ 820,712	-€ 4,961,141	-€ 9,922,282	###########	###########
3688	0.13	########	########	€ 621,407	€ 828,543	-€ 5,008,483	###########	###########	###########
3689	0.20	########	########	€ 933,071	#########	-€ 7,520,456	###########	###########	###########
3690	0.21	########	########	€ 978,353	#########	-€ 7,885,430	###########	###########	###########
3691	0.17	########	########	€ 783,454	#########	-€ 6,314,562	###########	###########	###########
3692	0.16	########	########	€ 771,656	#########	-€ 6,219,473	###########	###########	###########
3693	0.10	########	########	€ 467,830	€ 623,773	-€ 3,770,663	-€ 7,541,325	###########	###########
3694	0.13	########	########	€ 595,562	€ 794,083	-€ 4,800,172	-€ 9,600,343	###########	###########
3695	0.10	########	########	€ 475,899	€ 634,532	-€ 3,835,697	-€ 7,671,395	###########	###########
3696	0.14	########	########	€ 674,482	€ 899,310	-€ 5,436,262	###########	###########	###########
3697	0.16	########	########	€ 758,497	#########	-€ 6,113,407	###########	###########	###########
3698	0.10	########	########	€ 472,537	€ 630,049	-€ 3,808,599	-€ 7,617,199	###########	###########
3699	0.16	########	########	€ 765,588	#########	-€ 6,170,561	###########	###########	###########
3700	0.14	########	########	€ 682,024	€ 909,365	-€ 5,497,042	###########	###########	###########
3701	0.15	########	########	€ 691,848	€ 922,464	-€ 5,576,225	###########	###########	###########
3702	0.20	########	########	€ 935,726	#########	-€ 7,541,857	###########	###########	###########
3703	0.17	########	########	€ 797,452	#########	-€ 6,427,384	###########	###########	###########
3704	0.12	########	########	€ 557,071	€ 742,761	-€ 4,489,936	-€ 8,979,871	###########	###########
3705	0.16	########	########	€ 771,285	#########	-€ 6,216,483	###########	###########	###########
3706	0.10	########	########	€ 455,512	€ 607,349	-€ 3,671,378	-€ 7,342,756	###########	###########
3707	0.10	########	########	€ 489,501	€ 652,668	-€ 3,945,328	-€ 7,890,656	###########	###########
3708	0.12	########	########	€ 567,637	€ 756,849	-€ 4,575,096	-€ 9,150,192	###########	###########
3709	0.10	########	########	€ 473,109	€ 630,811	-€ 3,813,208	-€ 7,626,416	###########	###########
3710	0.17	########	########	€ 815,710	#########	-€ 6,574,542	###########	###########	###########
3711	0.21	########	########	€ 998,770	#########	-€ 8,049,988	###########	###########	###########
3712	0.16	########	########	€ 747,058	€ 996,077	-€ 6,021,213	###########	###########	###########
3713	0.13	########	########	€ 615,190	€ 820,254	-€ 4,958,372	-€ 9,916,744	###########	###########
3714	0.17	########	########	€ 816,052	#########	-€ 6,577,297	###########	###########	###########
3715	0.18	########	########	€ 826,986	#########	-€ 6,665,426	###########	###########	###########
3716	0.13	########	########	€ 637,683	€ 850,244	-€ 5,139,660	###########	###########	###########
3717	0.12	########	########	€ 585,671	€ 780,894	-€ 4,720,448	-€ 9,440,896	###########	###########
3718	0.18	########	########	€ 851,195	#########	-€ 6,860,545	###########	###########	###########
3719	0.20	########	########	€ 966,135	#########	-€ 7,786,951	###########	###########	###########
3720	0.23	########	########	#########	#########	-€ 8,711,202	###########	###########	###########
3721	0.10	########	########	€ 483,152	€ 644,203	-€ 3,894,159	-€ 7,788,318	###########	###########
3722	0.15	########	########	€ 691,865	€ 922,487	-€ 5,576,365	###########	###########	###########
3723	0.20	########	########	€ 932,930	#########	-€ 7,519,321	###########	###########	###########
3724	0.17	########	########	€ 801,505	#########	-€ 6,460,050	###########	###########	###########
3725	0.14	########	########	€ 667,742	€ 890,322	-€ 5,381,932	###########	###########	###########
3726	0.13	########	########	€ 626,503	€ 835,338	-€ 5,049,555	###########	###########	###########
3727	0.11	########	########	€ 531,477	€ 708,636	-€ 4,283,652	-€ 8,567,304	###########	###########
3728	0.19	########	########	€ 885,919	#########	-€ 7,140,422	###########	###########	###########
3729	0.18	########	########	€ 872,678	#########	-€ 7,033,701	###########	###########	###########
3730	0.16	########	########	€ 740,408	€ 987,211	-€ 5,967,614	###########	###########	###########
3731	0.17	########	########	€ 793,979	#########	-€ 6,399,390	###########	###########	###########
3732	0.10	########	########	€ 457,685	€ 610,246	-€ 3,688,892	-€ 7,377,785	###########	###########
3733	0.15	########	########	€ 703,588	€ 938,118	-€ 5,670,852	###########	###########	###########
3734	0.20	########	########	€ 924,914	#########	-€ 7,454,719	###########	###########	###########
3735	0.15	########	########	€ 719,363	€ 959,150	-€ 5,797,992	###########	###########	###########
3736	0.18	########	########	€ 864,361	#########	-€ 6,966,667	###########	###########	###########
3737	0.17	########	########	€ 780,280	#########	-€ 6,288,976	###########	###########	###########
3738	0.13	########	########	€ 629,480	€ 839,306	-€ 5,073,543	###########	###########	###########
3739	0.25	########	########	#########	#########	-€ 9,386,580	###########	###########	###########
3740	0.12	########	########	€ 575,860	€ 767,814	-€ 4,641,377	-€ 9,282,753	###########	###########
3741	0.17	########	########	€ 810,336	#########	-€ 6,531,228	###########	###########	###########
3742	0.15	########	########	€ 700,649	€ 934,199	-€ 5,647,162	###########	###########	###########
3743	0.12	########	########	€ 548,010	€ 730,681	-€ 4,416,910	-€ 8,833,820	###########	###########
3744	0.14	########	########	€ 683,487	€ 911,316	-€ 5,508,839	###########	###########	###########
3745	0.25	########	########	#########	#########	-€ 9,384,664	###########	###########	###########
3746	0.14	########	########	€ 675,710	€ 900,947	-€ 5,446,158	###########	###########	###########
3747	0.12	########	########	€ 589,888	€ 786,518	-€ 4,754,443	-€ 9,508,886	###########	###########
3748	0.08	########	########	€ 377,129	€ 502,839	-€ 3,039,622	-€ 6,079,244	-€ 9,118,866	###########
3749	0.18	########	########	€ 852,426	#########	-€ 6,870,468	###########	###########	###########
3750	0.16	########	########	€ 739,194	€ 985,592	-€ 5,957,829	###########	###########	###########
3751	0.17	########	########	€ 807,829	#########	-€ 6,511,022	###########	###########	###########
3752	0.13	########	########	€ 602,384	€ 803,179	-€ 4,855,157	-€ 9,710,313	###########	###########
3753	0.17	########	########	€ 805,867	#########	-€ 6,495,212	###########	###########	###########
3754	0.21	########	########	#########	#########	-€ 8,161,718	###########	###########	###########
3755	0.24	########	########	#########	#########	-€ 9,110,443	###########	###########	###########
3756	0.18	########	########	€ 861,871	#########	-€ 6,946,595	###########	###########	###########
3757	0.13	########	########	€ 622,443	€ 829,924	-€ 5,016,830	###########	###########	###########
3758	0.16	########	########	€ 757,586	#########	-€ 6,106,072	###########	###########	###########
3759	0.11	########	########	€ 508,127	€ 677,502	-€ 4,095,450	-€ 8,190,899	###########	###########
3760	0.19	########	########	€ 899,239	#########	-€ 7,247,779	###########	###########	###########
3761	0.17	########	########	€ 813,580	#########	-€ 6,557,373	###########	###########	###########
3762	0.20	########	########	€ 965,928	#########	-€ 7,785,286	###########	###########	###########
3763	0.15	########	########	€ 707,565	€ 943,420	-€ 5,702,902	###########	###########	###########
3764	0.21	########	########	€ 975,233	#########	-€ 7,860,282	###########	###########	###########
3765	0.15	########	########	€ 712,298	€ 949,730	-€ 5,741,050	###########	###########	###########
3766	0.17	########	########	€ 783,778	#########	-€ 6,317,174	###########	###########	###########
3767	0.21	########	########	€ 976,104	#########	-€ 7,867,302	###########	###########	###########
3768	0.16	########	########	€ 763,167	#########	-€ 6,151,049	###########	###########	###########
3769	0.14	########	########	€ 666,811	€ 889,082	-€ 5,374,432	###########	###########	###########
3770	0.16	########	########	€ 762,503	#########	-€ 6,145,699	###########	###########	###########
3771	0.11	########	########	€ 541,381	€ 721,842	-€ 4,363,479	-€ 8,726,959	###########	###########
3772	0.14	########	########	€ 651,933	€ 869,243	-€ 5,254,512	###########	###########	###########
3773	0.20	########	########	€ 953,339	#########	-€ 7,683,816	###########	###########	###########
3774	0.12	########	########	€ 581,847	€ 775,796	-€ 4,689,627	-€ 9,379,255	###########	###########
3775	0.22	########	########	#########	#########	-€ 8,369,255	###########	###########	###########
3776	0.12	########	########	€ 580,091	€ 773,455	-€ 4,675,475	-€ 9,350,950	###########	###########
3777	0.16	########	########	€ 737,273	€ 983,030	-€ 5,942,345	###########	###########	###########
3778	0.16	########	########	€ 769,716	#########	-€ 6,203,837	###########	###########	###########
3779	0.11	########	########	€ 526,465	€ 701,953	-€ 4,243,257	-€ 8,486,513	###########	###########
3780	0.14	########	########	€ 651,243	€ 868,324	-€ 5,248,956	###########	###########	###########

3781	0.13	########	########	€ 611,884	€ 815,845	-€ 4,931,725	-€ 9,863,449	##########	##########
3782	0.20	########	########	€ 924,723	#########	-€ 7,453,175	##########	##########	##########
3783	0.21	########	########	€ 986,816	#########	-€ 7,953,641	##########	##########	##########
3784	0.17	########	########	€ 792,774	#########	-€ 6,389,681	##########	##########	##########
3785	0.15	########	########	€ 703,913	€ 938,551	-€ 5,673,469	##########	##########	##########
3786	0.14	########	########	€ 667,201	€ 889,601	-€ 5,377,573	##########	##########	##########
3787	0.15	########	########	€ 729,685	€ 972,913	-€ 5,881,189	##########	##########	##########
3788	0.13	########	########	€ 634,657	€ 846,210	-€ 5,115,275	##########	##########	##########
3789	0.16	########	########	€ 743,075	€ 990,767	-€ 5,989,112	##########	##########	##########
3790	0.10	########	########	€ 495,279	€ 660,372	-€ 3,991,901	-€ 7,983,803	##########	##########
3791	0.10	########	########	€ 454,870	€ 606,494	-€ 3,666,211	-€ 7,332,421	##########	##########
3792	0.20	########	########	€ 941,780	#########	-€ 7,590,654	##########	##########	##########
3793	0.14	########	########	€ 679,488	€ 905,984	-€ 5,476,606	##########	##########	##########
3794	0.25	########	########	#########	#########	-€ 9,541,100	##########	##########	##########
3795	0.14	########	########	€ 668,738	€ 891,650	-€ 5,389,958	##########	##########	##########
3796	0.11	########	########	€ 537,477	€ 716,636	-€ 4,332,012	-€ 8,664,024	##########	##########
3797	0.11	########	########	€ 499,358	€ 665,810	-€ 4,024,775	-€ 8,049,549	##########	##########
3798	0.17	########	########	€ 825,619	#########	-€ 6,654,404	##########	##########	##########
3799	0.18	########	########	€ 871,984	#########	-€ 7,028,105	##########	##########	##########
3800	0.18	########	########	€ 846,341	#########	-€ 6,821,422	##########	##########	##########
3801	0.11	########	########	€ 514,589	€ 686,119	-€ 4,147,538	-€ 8,295,076	##########	##########
3802	0.17	########	########	€ 819,580	#########	-€ 6,605,730	##########	##########	##########
3803	0.16	########	########	€ 763,166	#########	-€ 6,151,046	##########	##########	##########
3804	0.19	########	########	€ 894,368	#########	-€ 7,208,518	##########	##########	##########
3805	0.20	########	########	€ 959,384	#########	-€ 7,732,539	##########	##########	##########
3806	0.13	########	########	€ 635,564	€ 847,418	-€ 5,122,580	##########	##########	##########
3807	0.14	########	########	€ 658,384	€ 877,845	-€ 5,306,506	##########	##########	##########
3808	0.15	########	########	€ 730,620	€ 974,159	-€ 5,888,722	##########	##########	##########
3809	0.11	########	########	€ 514,990	€ 686,653	-€ 4,150,765	-€ 8,301,531	##########	##########
3810	0.14	########	########	€ 643,532	€ 858,043	-€ 5,186,804	##########	##########	##########
3811	0.20	########	########	€ 959,631	#########	-€ 7,734,530	##########	##########	##########
3812	0.16	########	########	€ 754,170	#########	-€ 6,078,535	##########	##########	##########
3813	0.16	########	########	€ 772,745	#########	-€ 6,228,245	##########	##########	##########
3814	0.15	########	########	€ 722,687	€ 963,582	-€ 5,824,784	##########	##########	##########
3815	0.14	########	########	€ 651,125	€ 868,166	-€ 5,248,001	##########	##########	##########
3816	0.11	########	########	€ 514,215	€ 685,620	-€ 4,144,524	-€ 8,289,048	##########	##########
3817	0.20	########	########	€ 935,938	#########	-€ 7,543,570	##########	##########	##########
3818	0.17	########	########	€ 791,536	#########	-€ 6,379,704	##########	##########	##########
3819	0.18	########	########	€ 858,642	#########	-€ 6,920,571	##########	##########	##########
3820	0.14	########	########	€ 643,989	€ 858,652	-€ 5,190,485	##########	##########	##########
3821	0.17	########	########	€ 784,595	#########	-€ 6,323,760	##########	##########	##########
3822	0.16	########	########	€ 750,155	#########	-€ 6,046,179	##########	##########	##########
3823	0.20	########	########	€ 959,381	#########	-€ 7,732,514	##########	##########	##########
3824	0.18	########	########	€ 828,653	#########	-€ 6,678,863	##########	##########	##########
3825	0.21	########	########	€ 989,393	#########	-€ 7,974,410	##########	##########	##########
3826	0.17	########	########	€ 791,534	#########	-€ 6,379,687	##########	##########	##########
3827	0.13	########	########	€ 619,489	€ 825,985	-€ 4,993,019	-€ 9,986,038	##########	##########
3828	0.13	########	########	€ 625,603	€ 834,138	-€ 5,042,301	##########	##########	##########
3829	0.17	########	########	€ 801,937	#########	-€ 6,463,536	##########	##########	##########
3830	0.16	########	########	€ 735,605	€ 980,807	-€ 5,928,903	##########	##########	##########
3831	0.12	########	########	€ 578,186	€ 770,914	-€ 4,660,118	-€ 9,320,237	##########	##########
3832	0.14	########	########	€ 680,577	€ 907,436	-€ 5,485,383	##########	##########	##########
3833	0.16	########	########	€ 767,332	#########	-€ 6,184,616	##########	##########	##########
3834	0.21	########	########	#########	#########	-€ 8,070,226	##########	##########	##########
3835	0.14	########	########	€ 668,229	€ 890,972	-€ 5,385,860	##########	##########	##########
3836	0.19	########	########	€ 881,142	#########	-€ 7,101,913	##########	##########	##########
3837	0.19	########	########	€ 889,852	#########	-€ 7,172,122	##########	##########	##########
3838	0.18	########	########	€ 861,362	#########	-€ 6,942,490	##########	##########	##########
3839	0.20	########	########	€ 963,333	#########	-€ 7,764,367	##########	##########	##########
3840	0.15	########	########	€ 725,901	€ 967,868	-€ 5,850,691	##########	##########	##########
3841	0.12	########	########	€ 573,695	€ 764,927	-€ 4,623,925	-€ 9,247,849	##########	##########
3842	0.18	########	########	€ 857,025	#########	-€ 6,907,537	##########	##########	##########
3843	0.14	########	########	€ 680,864	€ 907,819	-€ 5,487,696	##########	##########	##########
3844	0.20	########	########	€ 950,590	#########	-€ 7,661,661	##########	##########	##########
3845	0.17	########	########	€ 787,798	#########	-€ 6,349,576	##########	##########	##########
3846	0.15	########	########	€ 695,172	€ 926,897	-€ 5,603,021	##########	##########	##########
3847	0.18	########	########	€ 839,568	#########	-€ 6,766,838	##########	##########	##########
3848	0.12	########	########	€ 587,853	€ 783,805	-€ 4,738,041	-€ 9,476,081	##########	##########
3849	0.16	########	########	€ 772,977	#########	-€ 6,230,115	##########	##########	##########
3850	0.19	########	########	€ 913,762	#########	-€ 7,364,828	##########	##########	##########
3851	0.09	########	########	€ 425,319	€ 567,093	-€ 3,428,033	-€ 6,856,065	##########	##########
3852	0.18	########	########	€ 857,606	#########	-€ 6,912,219	##########	##########	##########
3853	0.16	########	########	€ 757,700	#########	-€ 6,106,989	##########	##########	##########
3854	0.10	########	########	€ 451,544	€ 602,059	-€ 3,639,400	-€ 7,278,801	##########	##########
3855	0.17	########	########	€ 793,114	#########	-€ 6,392,420	##########	##########	##########
3856	0.21	########	########	€ 974,253	#########	-€ 7,852,384	##########	##########	##########
3857	0.17	########	########	€ 801,681	#########	-€ 6,461,468	##########	##########	##########
3858	0.13	########	########	€ 599,463	€ 799,283	-€ 4,831,609	-€ 9,663,218	##########	##########
3859	0.15	########	########	€ 722,046	€ 962,728	-€ 5,819,620	##########	##########	##########
3860	0.14	########	########	€ 683,652	€ 911,536	-€ 5,510,167	##########	##########	##########
3861	0.19	########	########	€ 904,354	#########	-€ 7,289,000	##########	##########	##########
3862	0.17	########	########	€ 810,662	#########	-€ 6,533,857	##########	##########	##########
3863	0.16	########	########	€ 767,421	#########	-€ 6,185,334	##########	##########	##########
3864	0.16	########	########	€ 778,606	#########	-€ 6,275,486	##########	##########	##########
3865	0.15	########	########	€ 718,395	€ 957,860	-€ 5,790,190	##########	##########	##########
3866	0.15	########	########	€ 687,326	€ 916,434	-€ 5,539,776	##########	##########	##########
3867	0.16	########	########	€ 759,296	#########	-€ 6,119,850	##########	##########	##########
3868	0.20	########	########	€ 922,314	#########	-€ 7,433,757	##########	##########	##########
3869	0.19	########	########	€ 882,571	#########	-€ 7,113,431	##########	##########	##########
3870	0.14	########	########	€ 643,402	€ 857,869	-€ 5,185,753	##########	##########	##########
3871	0.15	########	########	€ 712,859	€ 950,479	-€ 5,745,574	##########	##########	##########
3872	0.18	########	########	€ 864,129	#########	-€ 6,964,791	##########	##########	##########
3873	0.12	########	########	€ 577,588	€ 770,117	-€ 4,655,300	-€ 9,310,599	##########	##########
3874	0.13	########	########	€ 599,725	€ 799,634	-€ 4,833,726	-€ 9,667,451	##########	##########
3875	0.18	########	########	€ 855,973	#########	-€ 6,899,062	##########	##########	##########
3876	0.19	########	########	€ 893,689	#########	-€ 7,203,046	##########	##########	##########
3877	0.13	########	########	€ 632,524	€ 843,366	-€ 5,098,085	##########	##########	##########
3878	0.09	########	########	€ 444,279	€ 592,372	-€ 3,580,847	-€ 7,161,694	##########	##########
3879	0.17	########	########	€ 781,640	#########	-€ 6,299,939	##########	##########	##########
3880	0.25	########	########	#########	#########	-€ 9,450,922	##########	##########	##########
3881	0.14	########	########	€ 666,125	€ 888,166	-€ 5,368,899	##########	##########	##########
3882	0.16	########	########	€ 744,494	€ 992,659	-€ 6,000,549	##########	##########	##########
3883	0.10	########	########	€ 479,655	€ 639,540	-€ 3,865,970	-€ 7,731,939	##########	##########
3884	0.23	########	########	#########	#########	-€ 8,705,826	##########	##########	##########
3885	0.11	########	########	€ 503,781	€ 671,708	-€ 4,060,427	-€ 8,120,855	##########	##########
3886	0.11	########	########	€ 523,480	€ 697,973	-€ 4,219,196	-€ 8,438,392	##########	##########
3887	0.13	########	########	€ 601,494	€ 801,992	-€ 4,847,982	-€ 9,695,963	##########	##########
3888	0.14	########	########	€ 659,104	€ 878,806	-€ 5,312,314	##########	##########	##########
3889	0.12	########	########	€ 564,600	€ 752,800	-€ 4,550,619	-€ 9,101,238	##########	##########
3890	0.16	########	########	€ 749,029	€ 998,705	-€ 6,037,100	##########	##########	##########
3891	0.15	########	########	€ 694,438	€ 925,917	-€ 5,597,098	##########	##########	##########
3892	0.16	########	########	€ 751,381	#########	-€ 6,056,060	##########	##########	##########
3893	0.18	########	########	€ 838,103	#########	-€ 6,755,023	##########	##########	##########
3894	0.16	########	########	€ 752,313	#########	-€ 6,063,565	##########	##########	##########
3895	0.23	########	########	#########	#########	-€ 8,641,409	##########	##########	##########
3896	0.20	########	########	€ 928,999	#########	-€ 7,487,642	##########	##########	##########
3897	0.17	########	########	€ 809,435	#########	-€ 6,523,967	##########	##########	##########
3898	0.15	########	########	€ 690,173	€ 920,230	-€ 5,562,724	##########	##########	##########
3899	0.16	########	########	€ 777,155	#########	-€ 6,263,791	##########	##########	##########
3900	0.13	########	########	€ 600,223	€ 800,298	-€ 4,837,740	-€ 9,675,480	##########	##########
3901	0.14	########	########	€ 674,558	€ 899,410	-€ 5,436,869	##########	##########	##########
3902	0.17	########	########	€ 795,537	#########	-€ 6,411,948	##########	##########	##########
3903	0.13	########	########	€ 636,359	€ 848,478	-€ 5,128,988	##########	##########	##########
3904	0.13	########	########	€ 611,159	€ 814,879	-€ 4,925,884	-€ 9,851,768	##########	##########
3905	0.22	########	########	#########	#########	-€ 8,483,876	##########	##########	##########
3906	0.14	########	########	€ 651,163	€ 868,217	-€ 5,248,309	##########	##########	##########
3907	0.20	########	########	€ 943,192	#########	-€ 7,602,034	##########	##########	##########
3908	0.28	########	########	#########	#########	##########	##########	##########	##########
3909	0.22	########	########	#########	#########	-€ 8,382,337	##########	##########	##########
3910	0.16	########	########	€ 749,896	€ 999,861	-€ 6,044,087	##########	##########	##########
3911	0.19	########	########	€ 919,367	#########	-€ 7,410,004	##########	##########	##########
3912	0.19	########	########	€ 914,908	#########	-€ 7,374,067	##########	##########	##########
3913	0.18	########	########	€ 856,305	#########	-€ 6,901,733	##########	##########	##########
3914	0.16	########	########	€ 752,070	#########	-€ 6,061,611	##########	##########	##########
3915	0.18	########	########	€ 830,146	#########	-€ 6,690,891	##########	##########	##########
3916	0.15	########	########	€ 695,801	€ 927,735	-€ 5,608,089	##########	##########	##########
3917	0.17	########	########	€ 808,567	#########	-€ 6,516,969	##########	##########	##########
3918	0.19	########	########	€ 888,617	#########	-€ 7,162,163	##########	##########	##########
3919	0.22	########	########	#########	#########	-€ 8,387,186	##########	##########	##########
3920	0.16	########	########	€ 774,868	#########	-€ 6,245,360	##########	##########	##########
3921	0.17	########	########	€ 793,523	#########	-€ 6,395,716	##########	##########	##########
3922	0.21	########	########	#########	#########	-€ 8,124,742	##########	##########	##########
3923	0.09	########	########	€ 421,660	€ 562,213	-€ 3,398,537	-€ 6,797,075	##########	##########
3924	0.14	########	########	€ 684,177	€ 912,237	-€ 5,514,403	##########	##########	##########
3925	0.16	########	########	€ 770,149	#########	-€ 6,207,327	##########	##########	##########
3926	0.14	########	########	€ 649,023	€ 865,364	-€ 5,231,060	##########	##########	##########
3927	0.15	########	########	€ 686,774	€ 915,698	-€ 5,535,328	##########	##########	##########
3928	0.19	########	########	€ 889,117	#########	-€ 7,166,191	##########	##########	##########
3929	0.15	########	########	€ 700,908	€ 934,544	-€ 5,649,250	##########	##########	##########
3930	0.20	########	########	€ 929,388	#########	-€ 7,490,776	##########	##########	##########
3931	0.12	########	########	€ 560,026	€ 746,702	-€ 4,513,756	-€ 9,027,512	##########	##########
3932	0.15	########	########	€ 695,662	€ 927,550	-€ 5,606,971	##########	##########	##########
3933	0.18	########	########	€ 840,895	#########	-€ 6,777,532	##########	##########	##########
3934	0.15	########	########	€ 731,932	€ 975,909	-€ 5,899,298	##########	##########	##########
3935	0.19	########	########	€ 896,694	#########	-€ 7,227,269	##########	##########	##########
3936	0.16	########	########	€ 757,483	#########	-€ 6,105,238	##########	##########	##########
3937	0.15	########	########	€ 713,030	€ 950,707	-€ 5,746,950	##########	##########	##########
3938	0.19	########	########	€ 894,476	#########	-€ 7,209,384	##########	##########	##########
3939	0.18	########	########	€ 845,351	#########	-€ 6,813,442	##########	##########	##########
3940	0.14	########	########	€ 648,399	€ 864,532	-€ 5,226,034	##########	##########	##########
3941	0.11	########	########	€ 518,299	€ 691,066	-€ 4,177,441	-€ 8,354,882	##########	##########
3942	0.09	########	########	€ 408,099	€ 544,132	-€ 3,289,236	-€ 6,578,473	-€ 9,867,709	##########
3943	0.17	########	########	€ 821,102	#########	-€ 6,618,000	##########	##########	##########
3944	0.17	########	########	€ 795,964	#########	-€ 6,415,395	##########	##########	##########
3945	0.18	########	########	€ 840,456	#########	-€ 6,773,992	##########	##########	##########
3946	0.14	########	########	€ 676,274	€ 901,698	-€ 5,450,698	##########	##########	##########
3947	0.17	########	########	€ 782,160	#########	-€ 6,304,135	##########	##########	##########
3948	0.16	########	########	€ 732,921	€ 977,229	-€ 5,907,274	##########	##########	##########
3949	0.14	########	########	€ 656,789	€ 875,718	-€ 5,293,651	##########	##########	##########
3950	0.14	########	########	€ 679,052	€ 905,403	-€ 5,473,094	##########	##########	##########
3951	0.19	########	########	€ 894,833	#########	-€ 7,212,262	##########	##########	##########
3952	0.10	########	########	€ 472,656	€ 630,208	-€ 3,809,559	-€ 7,619,119	##########	##########

3953	0.15	########	########	€ 725,975	€ 967,967	-€ 5,851,286	###########	###########	###########
3954	0.15	########	########	€ 712,449	€ 949,932	-€ 5,742,267	###########	###########	###########
3955	0.14	########	########	€ 655,704	€ 874,272	-€ 5,284,908	###########	###########	###########
3956	0.19	########	########	€ 892,597	#########	-€ 7,194,247	###########	###########	###########
3957	0.13	########	########	€ 607,940	€ 810,587	-€ 4,899,940	-€ 9,799,879	###########	###########
3958	0.17	########	########	€ 787,562	#########	-€ 6,347,674	###########	###########	###########
3959	0.20	########	########	€ 961,108	#########	-€ 7,746,432	###########	###########	###########
3960	0.09	########	########	€ 430,565	€ 574,087	-€ 3,470,312	-€ 6,940,623	###########	###########
3961	0.16	########	########	€ 748,424	€ 997,898	-€ 6,032,219	###########	###########	###########
3962	0.15	########	########	€ 705,238	€ 940,318	-€ 5,684,150	###########	###########	###########
3963	0.20	########	########	€ 956,039	#########	-€ 7,705,577	###########	###########	###########
3964	0.11	########	########	€ 527,514	€ 703,353	-€ 4,251,714	-€ 8,503,429	###########	###########
3965	0.16	########	########	€ 734,019	€ 978,692	-€ 5,916,120	###########	###########	###########
3966	0.13	########	########	€ 622,370	€ 829,827	-€ 5,016,241	###########	###########	###########
3967	0.21	########	########	#########	#########	-€ 8,133,169	###########	###########	###########
3968	0.18	########	########	€ 854,037	#########	-€ 6,883,452	###########	###########	###########
3969	0.12	########	########	€ 577,355	€ 769,807	-€ 4,653,424	-€ 9,306,848	###########	###########
3970	0.15	########	########	€ 721,186	€ 961,582	-€ 5,812,692	###########	###########	###########
3971	0.23	########	########	#########	#########	-€ 8,891,995	###########	###########	###########
3972	0.25	########	########	#########	#########	-€ 9,426,799	###########	###########	###########
3973	0.09	########	########	€ 426,923	€ 569,231	-€ 3,440,956	-€ 6,881,913	###########	###########
3974	0.15	########	########	€ 721,812	€ 962,416	-€ 5,817,731	###########	###########	###########
3975	0.14	########	########	€ 656,906	€ 875,875	-€ 5,294,598	###########	###########	###########
3976	0.17	########	########	€ 810,194	#########	-€ 6,530,083	###########	###########	###########
3977	0.16	########	########	€ 759,064	#########	-€ 6,117,984	###########	###########	###########
3978	0.20	########	########	€ 951,174	#########	-€ 7,666,367	###########	###########	###########
3979	0.12	########	########	€ 566,470	€ 755,293	-€ 4,565,689	-€ 9,131,378	###########	###########
3980	0.13	########	########	€ 622,846	€ 830,461	-€ 5,020,074	###########	###########	###########
3981	0.15	########	########	€ 706,081	€ 941,441	-€ 5,690,941	###########	###########	###########
3982	0.19	########	########	€ 884,353	#########	-€ 7,127,797	###########	###########	###########
3983	0.18	########	########	€ 843,003	#########	-€ 6,794,520	###########	###########	###########
3984	0.14	########	########	€ 647,578	€ 863,437	-€ 5,219,415	###########	###########	###########
3985	0.16	########	########	€ 758,625	#########	-€ 6,114,444	###########	###########	###########
3986	0.19	########	########	€ 904,006	#########	-€ 7,286,196	###########	###########	###########
3987	0.18	########	########	€ 833,311	#########	-€ 6,716,403	###########	###########	###########
3988	0.17	########	########	€ 811,076	#########	-€ 6,537,190	###########	###########	###########
3989	0.11	########	########	€ 506,749	€ 675,665	-€ 4,084,345	-€ 8,168,690	###########	###########
3990	0.17	########	########	€ 816,248	#########	-€ 6,578,877	###########	###########	###########
3991	0.18	########	########	€ 830,982	#########	-€ 6,697,637	###########	###########	###########
3992	0.22	########	########	#########	#########	-€ 8,458,841	###########	###########	###########
3993	0.24	########	########	#########	#########	-€ 9,134,412	###########	###########	###########
3994	0.16	########	########	€ 763,431	#########	-€ 6,153,175	###########	###########	###########
3995	0.14	########	########	€ 675,200	€ 900,267	-€ 5,442,045	###########	###########	###########
3996	0.14	########	########	€ 669,943	€ 893,258	-€ 5,399,677	###########	###########	###########
3997	0.18	########	########	€ 862,291	#########	-€ 6,949,976	###########	###########	###########
3998	0.21	########	########	€ 976,881	#########	-€ 7,873,563	###########	###########	###########
3999	0.21	########	########	€ 976,633	#########	-€ 7,871,569	###########	###########	###########
4000	0.08	########	########	€ 390,898	€ 521,197	-€ 3,150,597	-€ 6,301,195	-€ 9,451,792	###########
4001	0.18	########	########	€ 827,729	#########	-€ 6,671,410	###########	###########	###########
4002	0.15	########	########	€ 686,412	€ 915,217	-€ 5,532,416	###########	###########	###########
4003	0.15	########	########	€ 687,670	€ 916,894	-€ 5,542,556	###########	###########	###########
4004	0.15	########	########	€ 689,612	€ 919,483	-€ 5,558,206	###########	###########	###########
4005	0.16	########	########	€ 762,688	#########	-€ 6,147,187	###########	###########	###########
4006	0.19	########	########	€ 876,685	#########	-€ 7,065,996	###########	###########	###########
4007	0.16	########	########	€ 758,881	#########	-€ 6,116,502	###########	###########	###########
4008	0.16	########	########	€ 772,232	#########	-€ 6,224,113	###########	###########	###########
4009	0.18	########	########	€ 841,082	#########	-€ 6,779,040	###########	###########	###########
4010	0.21	########	########	€ 969,215	#########	-€ 7,811,779	###########	###########	###########
4011	0.18	########	########	€ 861,243	#########	-€ 6,941,537	###########	###########	###########
4012	0.12	########	########	€ 577,203	€ 769,604	-€ 4,652,201	-€ 9,304,403	###########	###########
4013	0.11	########	########	€ 502,763	€ 670,351	-€ 4,052,220	-€ 8,104,439	###########	###########
4014	0.20	########	########	€ 929,801	#########	-€ 7,494,101	###########	###########	###########
4015	0.11	########	########	€ 505,856	€ 674,475	-€ 4,077,151	-€ 8,154,302	###########	###########
4016	0.13	########	########	€ 633,102	€ 844,136	-€ 5,102,742	###########	###########	###########
4017	0.20	########	########	€ 953,398	#########	-€ 7,684,297	###########	###########	###########
4018	0.13	########	########	€ 592,848	€ 790,464	-€ 4,778,296	-€ 9,556,593	###########	###########
4019	0.12	########	########	€ 581,393	€ 775,191	-€ 4,685,973	-€ 9,371,947	###########	###########
4020	0.16	########	########	€ 743,010	€ 990,680	-€ 5,988,586	###########	###########	###########
4021	0.20	########	########	€ 952,762	#########	-€ 7,679,170	###########	###########	###########
4022	0.24	########	########	#########	#########	-€ 9,063,663	###########	###########	###########
4023	0.16	########	########	€ 773,785	#########	-€ 6,236,634	###########	###########	###########
4024	0.17	########	########	€ 818,637	#########	-€ 6,598,133	###########	###########	###########
4025	0.14	########	########	€ 673,986	€ 898,648	-€ 5,432,262	###########	###########	###########
4026	0.13	########	########	€ 599,392	€ 799,189	-€ 4,831,037	-€ 9,662,074	###########	###########
4027	0.14	########	########	€ 673,056	€ 897,408	-€ 5,424,764	###########	###########	###########
4028	0.16	########	########	€ 774,566	#########	-€ 6,242,926	###########	###########	###########
4029	0.15	########	########	€ 689,988	€ 919,984	-€ 5,561,235	###########	###########	###########
4030	0.12	########	########	€ 552,471	€ 736,628	-€ 4,452,861	-€ 8,905,722	###########	###########
4031	0.19	########	########	€ 897,856	#########	-€ 7,236,628	###########	###########	###########
4032	0.17	########	########	€ 793,452	#########	-€ 6,395,148	###########	###########	###########
4033	0.17	########	########	€ 817,857	#########	-€ 6,591,847	###########	###########	###########
4034	0.18	########	########	€ 831,253	#########	-€ 6,699,820	###########	###########	###########
4035	0.17	########	########	€ 812,725	#########	-€ 6,550,485	###########	###########	###########
4036	0.15	########	########	€ 689,519	€ 919,359	-€ 5,557,457	###########	###########	###########
4037	0.14	########	########	€ 655,888	€ 874,518	-€ 5,286,396	###########	###########	###########
4038	0.17	########	########	€ 822,074	#########	-€ 6,625,839	###########	###########	###########
4039	0.12	########	########	€ 576,860	€ 769,146	-€ 4,649,433	-€ 9,298,867	###########	###########
4040	0.19	########	########	€ 911,786	#########	-€ 7,348,908	###########	###########	###########
4041	0.17	########	########	€ 817,459	#########	-€ 6,588,637	###########	###########	###########
4042	0.14	########	########	€ 640,355	€ 853,806	-€ 5,161,194	###########	###########	###########
4043	0.18	########	########	€ 831,149	#########	-€ 6,698,981	###########	###########	###########
4044	0.16	########	########	€ 748,589	€ 998,119	-€ 6,033,556	###########	###########	###########
4045	0.21	########	########	€ 981,909	#########	-€ 7,914,088	###########	###########	###########
4046	0.16	########	########	€ 758,715	#########	-€ 6,115,170	###########	###########	###########
4047	0.16	########	########	€ 744,109	€ 992,145	-€ 5,997,445	###########	###########	###########
4048	0.20	########	########	€ 945,947	#########	-€ 7,624,242	###########	###########	###########
4049	0.22	########	########	#########	#########	-€ 8,309,508	###########	###########	###########
4050	0.12	########	########	€ 587,357	€ 783,143	-€ 4,734,040	-€ 9,468,080	###########	###########
4051	0.16	########	########	€ 737,030	€ 982,707	-€ 5,940,389	###########	###########	###########
4052	0.13	########	########	€ 603,651	€ 804,868	-€ 4,865,365	-€ 9,730,731	###########	###########
4053	0.14	########	########	€ 676,407	€ 901,875	-€ 5,451,770	###########	###########	###########
4054	0.12	########	########	€ 570,970	€ 761,293	-€ 4,601,960	-€ 9,203,920	###########	###########
4055	0.12	########	########	€ 581,246	€ 774,995	-€ 4,684,789	-€ 9,369,578	###########	###########
4056	0.13	########	########	€ 629,273	€ 839,031	-€ 5,071,878	###########	###########	###########
4057	0.12	########	########	€ 567,412	€ 756,550	-€ 4,573,287	-€ 9,146,575	###########	###########
4058	0.13	########	########	€ 631,752	€ 842,337	-€ 5,091,862	###########	###########	###########
4059	0.11	########	########	€ 525,201	€ 700,268	-€ 4,233,070	-€ 8,466,140	###########	###########
4060	0.16	########	########	€ 748,282	€ 997,710	-€ 6,031,082	###########	###########	###########
4061	0.19	########	########	€ 889,737	#########	-€ 7,171,194	###########	###########	###########
4062	0.16	########	########	€ 743,700	€ 991,600	-€ 5,994,146	###########	###########	###########
4063	0.18	########	########	€ 867,461	#########	-€ 6,991,646	###########	###########	###########
4064	0.21	########	########	€ 986,509	#########	-€ 7,951,168	###########	###########	###########
4065	0.21	########	########	€ 978,757	#########	-€ 7,888,687	###########	###########	###########
4066	0.14	########	########	€ 672,132	€ 896,176	-€ 5,417,320	###########	###########	###########
4067	0.14	########	########	€ 664,834	€ 886,446	-€ 5,358,499	###########	###########	###########
4068	0.18	########	########	€ 873,246	#########	-€ 7,038,276	###########	###########	###########
4069	0.18	########	########	€ 834,646	#########	-€ 6,727,161	###########	###########	###########
4070	0.11	########	########	€ 511,191	€ 681,588	-€ 4,120,148	-€ 8,240,296	###########	###########
4071	0.10	########	########	€ 488,682	€ 651,575	-€ 3,938,725	-€ 7,877,451	###########	###########
4072	0.13	########	########	€ 625,511	€ 834,014	-€ 5,041,554	###########	###########	###########
4073	0.13	########	########	€ 593,554	€ 791,405	-€ 4,783,984	-€ 9,567,968	###########	###########
4074	0.22	########	########	#########	#########	-€ 8,420,533	###########	###########	###########
4075	0.19	########	########	€ 915,334	#########	-€ 7,377,500	###########	###########	###########
4076	0.14	########	########	€ 681,550	€ 908,733	-€ 5,493,222	###########	###########	###########
4077	0.15	########	########	€ 728,682	€ 971,576	-€ 5,873,106	###########	###########	###########
4078	0.08	########	########	€ 385,764	€ 514,352	-€ 3,109,219	-€ 6,218,439	-€ 9,327,658	###########
4079	0.16	########	########	€ 755,978	#########	-€ 6,093,109	###########	###########	###########
4080	0.15	########	########	€ 688,231	€ 917,641	-€ 5,547,070	###########	###########	###########
4081	0.11	########	########	€ 498,931	€ 665,241	-€ 4,021,333	-€ 8,042,665	###########	###########
4082	0.21	########	########	€ 973,056	#########	-€ 7,842,734	###########	###########	###########
4083	0.15	########	########	€ 696,219	€ 928,293	-€ 5,611,459	###########	###########	###########
4084	0.17	########	########	€ 825,340	#########	-€ 6,652,158	###########	###########	###########
4085	0.12	########	########	€ 579,492	€ 772,656	-€ 4,670,650	-€ 9,341,301	###########	###########
4086	0.15	########	########	€ 710,700	€ 947,600	-€ 5,728,175	###########	###########	###########
4087	0.16	########	########	€ 744,970	€ 993,293	-€ 6,004,382	###########	###########	###########
4088	0.23	########	########	#########	#########	-€ 8,845,333	###########	###########	###########
4089	0.09	########	########	€ 408,204	€ 544,272	-€ 3,290,082	-€ 6,580,165	-€ 9,870,247	###########
4090	0.11	########	########	€ 532,724	€ 710,299	-€ 4,293,706	-€ 8,587,411	###########	###########
4091	0.13	########	########	€ 619,334	€ 825,779	-€ 4,991,772	-€ 9,983,544	###########	###########
4092	0.12	########	########	€ 574,571	€ 766,095	-€ 4,630,985	-€ 9,261,969	###########	###########
4093	0.11	########	########	€ 540,707	€ 720,943	-€ 4,358,049	-€ 8,716,097	###########	###########
4094	0.18	########	########	€ 849,618	#########	-€ 6,847,835	###########	###########	###########
4095	0.19	########	########	€ 883,337	#########	-€ 7,119,610	###########	###########	###########
4096	0.12	########	########	€ 545,493	€ 727,325	-€ 4,396,623	-€ 8,793,245	###########	###########
4097	0.16	########	########	€ 760,372	#########	-€ 6,128,523	###########	###########	###########
4098	0.22	########	########	#########	#########	-€ 8,551,899	###########	###########	###########
4099	0.14	########	########	€ 661,953	€ 882,603	-€ 5,335,272	###########	###########	###########
4100	0.19	########	########	€ 889,403	#########	-€ 7,168,504	###########	###########	###########
4101	0.14	########	########	€ 641,887	€ 855,850	-€ 5,173,549	###########	###########	###########
4102	0.18	########	########	€ 858,864	#########	-€ 6,922,358	###########	###########	###########
4103	0.14	########	########	€ 654,563	€ 872,751	-€ 5,275,717	###########	###########	###########
4104	0.20	########	########	€ 925,698	#########	-€ 7,461,037	###########	###########	###########
4105	0.23	########	########	#########	#########	-€ 8,767,205	###########	###########	###########
4106	0.20	########	########	€ 941,386	#########	-€ 7,587,476	###########	###########	###########
4107	0.19	########	########	€ 917,006	#########	-€ 7,390,982	###########	###########	###########
4108	0.17	########	########	€ 821,313	#########	-€ 6,619,698	###########	###########	###########
4109	0.14	########	########	€ 652,214	€ 869,619	-€ 5,256,783	###########	###########	###########
4110	0.12	########	########	€ 547,318	€ 729,757	-€ 4,411,325	-€ 8,822,650	###########	###########
4111	0.19	########	########	€ 914,812	#########	-€ 7,373,293	###########	###########	###########
4112	0.25	########	########	#########	#########	-€ 9,668,851	###########	###########	###########
4113	0.11	########	########	€ 512,966	€ 683,955	-€ 4,134,459	-€ 8,268,918	###########	###########
4114	0.12	########	########	€ 568,033	€ 757,377	-€ 4,578,289	-€ 9,156,579	###########	###########
4115	0.20	########	########	€ 963,812	#########	-€ 7,768,233	###########	###########	###########
4116	0.18	########	########	€ 868,256	#########	-€ 6,998,059	###########	###########	###########
4117	0.17	########	########	€ 786,163	#########	-€ 6,336,398	###########	###########	###########
4118	0.18	########	########	€ 831,858	#########	-€ 6,704,692	###########	###########	###########
4119	0.15	########	########	€ 695,410	€ 927,213	-€ 5,604,937	###########	###########	###########
4120	0.12	########	########	€ 572,669	€ 763,559	-€ 4,615,655	-€ 9,231,310	###########	###########
4121	0.15	########	########	€ 694,175	€ 925,567	-€ 5,594,981	###########	###########	###########
4122	0.21	########	########	#########	#########	-€ 8,132,961	###########	###########	###########
4123	0.14	########	########	€ 667,369	€ 889,826	-€ 5,378,930	###########	###########	###########
4124	0.14	########	########	€ 670,968	€ 894,624	-€ 5,407,937	###########	###########	###########

4125	0.09	########	########	€ 408,518	€ 544,690	-€ 3,292,611	-€ 6,585,222	-€ 9,877,833	###########
4126	0.17	########	########	€ 821,598	#########	-€ 6,621,996	###########	###########	###########
4127	0.15	########	########	€ 704,956	€ 939,941	-€ 5,681,875	###########	###########	###########
4128	0.13	########	########	€ 602,390	€ 803,187	-€ 4,855,206	-€ 9,710,413	###########	###########
4129	0.18	########	########	€ 833,685	#########	-€ 6,719,415	###########	###########	###########
4130	0.12	########	########	€ 563,997	€ 751,996	-€ 4,545,761	-€ 9,091,521	###########	###########
4131	0.14	########	########	€ 661,821	€ 882,428	-€ 5,334,212	###########	###########	###########
4132	0.16	########	########	€ 732,582	€ 976,776	-€ 5,904,540	###########	###########	###########
4133	0.19	########	########	€ 905,190	#########	-€ 7,295,745	###########	###########	###########
4134	0.25	########	########	#########	#########	-€ 9,578,304	###########	###########	###########
4135	0.08	########	########	€ 371,400	€ 495,200	-€ 2,993,446	-€ 5,986,893	-€ 8,980,339	###########
4136	0.13	########	########	€ 622,789	€ 830,385	-€ 5,019,619	###########	###########	###########
4137	0.15	########	########	€ 696,907	€ 929,209	-€ 5,616,999	###########	###########	###########
4138	0.14	########	########	€ 643,045	€ 857,394	-€ 5,182,880	###########	###########	###########
4139	0.19	########	########	€ 874,944	#########	-€ 7,051,960	###########	###########	###########
4140	0.11	########	########	€ 500,082	€ 666,776	-€ 4,030,611	-€ 8,061,222	###########	###########
4141	0.19	########	########	€ 897,894	#########	-€ 7,236,936	###########	###########	###########
4142	0.19	########	########	€ 887,558	#########	-€ 7,153,627	###########	###########	###########
4143	0.17	########	########	€ 801,751	#########	-€ 6,462,037	###########	###########	###########
4144	0.22	########	########	#########	#########	-€ 8,297,208	###########	###########	###########
4145	0.20	########	########	€ 952,934	#########	-€ 7,680,555	###########	###########	###########
4146	0.20	########	########	€ 955,673	#########	-€ 7,702,629	###########	###########	###########
4147	0.20	########	########	€ 966,538	#########	-€ 7,790,201	###########	###########	###########
4148	0.21	########	########	€ 995,746	#########	-€ 8,025,615	###########	###########	###########
4149	0.15	########	########	€ 692,637	€ 923,516	-€ 5,582,589	###########	###########	###########
4150	0.24	########	########	#########	#########	-€ 9,275,570	###########	###########	###########
4151	0.22	########	########	#########	#########	-€ 8,525,589	###########	###########	###########
4152	0.12	########	########	€ 575,226	€ 766,968	-€ 4,636,263	-€ 9,272,527	###########	###########
4153	0.14	########	########	€ 652,525	€ 870,033	-€ 5,259,285	###########	###########	###########
4154	0.13	########	########	€ 619,126	€ 825,501	-€ 4,990,093	-€ 9,980,185	###########	###########
4155	0.15	########	########	€ 729,207	€ 972,277	-€ 5,877,340	###########	###########	###########
4156	0.19	########	########	€ 910,988	#########	-€ 7,342,476	###########	###########	###########
4157	0.11	########	########	€ 517,205	€ 689,606	-€ 4,168,618	-€ 8,337,236	###########	###########
4158	0.17	########	########	€ 815,693	#########	-€ 6,574,407	###########	###########	###########
4159	0.13	########	########	€ 596,158	€ 794,878	-€ 4,804,976	-€ 9,609,951	###########	###########
4160	0.16	########	########	€ 757,028	#########	-€ 6,101,572	###########	###########	###########
4161	0.21	########	########	€ 997,306	#########	-€ 8,038,184	###########	###########	###########
4162	0.17	########	########	€ 780,007	#########	-€ 6,286,783	###########	###########	###########
4163	0.11	########	########	€ 540,890	€ 721,187	-€ 4,359,522	-€ 8,719,045	###########	###########
4164	0.18	########	########	€ 834,275	#########	-€ 6,724,170	###########	###########	###########
4165	0.19	########	########	€ 905,939	#########	-€ 7,301,777	###########	###########	###########
4166	0.10	########	########	€ 471,381	€ 628,508	-€ 3,799,283	-€ 7,598,566	###########	###########
4167	0.20	########	########	€ 962,816	#########	-€ 7,760,202	###########	###########	###########
4168	0.17	########	########	€ 785,033	#########	-€ 6,327,288	###########	###########	###########
4169	0.16	########	########	€ 767,585	#########	-€ 6,186,661	###########	###########	###########
4170	0.16	########	########	€ 776,471	#########	-€ 6,258,277	###########	###########	###########
4171	0.15	########	########	€ 709,573	€ 946,098	-€ 5,719,089	###########	###########	###########
4172	0.15	########	########	€ 709,421	€ 945,895	-€ 5,717,865	###########	###########	###########
4173	0.14	########	########	€ 647,990	€ 863,987	-€ 5,222,737	###########	###########	###########
4174	0.16	########	########	€ 742,719	€ 990,292	-€ 5,986,242	###########	###########	###########
4175	0.12	########	########	€ 565,716	€ 754,288	-€ 4,559,616	-€ 9,119,232	###########	###########
4176	0.17	########	########	€ 808,474	#########	-€ 6,516,224	###########	###########	###########
4177	0.12	########	########	€ 559,533	€ 746,044	-€ 4,509,781	-€ 9,019,562	###########	###########
4178	0.14	########	########	€ 656,290	€ 875,054	-€ 5,289,635	###########	###########	###########
4179	0.15	########	########	€ 727,097	€ 969,462	-€ 5,860,328	###########	###########	###########
4180	0.18	########	########	€ 852,216	#########	-€ 6,868,780	###########	###########	###########
4181	0.14	########	########	€ 647,316	€ 863,088	-€ 5,217,304	###########	###########	###########
4182	0.22	########	########	#########	#########	-€ 8,547,347	###########	###########	###########
4183	0.16	########	########	€ 778,854	#########	-€ 6,277,486	###########	###########	###########
4184	0.19	########	########	€ 909,358	#########	-€ 7,329,338	###########	###########	###########
4185	0.16	########	########	€ 736,777	€ 982,370	-€ 5,938,354	###########	###########	###########
4186	0.15	########	########	€ 693,021	€ 924,028	-€ 5,585,681	###########	###########	###########
4187	0.19	########	########	€ 919,911	#########	-€ 7,414,391	###########	###########	###########
4188	0.11	########	########	€ 520,994	€ 694,658	-€ 4,199,156	-€ 8,398,313	###########	###########
4189	0.14	########	########	€ 680,355	€ 907,140	-€ 5,483,595	###########	###########	###########
4190	0.17	########	########	€ 822,484	#########	-€ 6,629,138	###########	###########	###########
4191	0.14	########	########	€ 652,486	€ 869,981	-€ 5,258,972	###########	###########	###########
4192	0.16	########	########	€ 747,928	€ 997,237	-€ 6,028,222	###########	###########	###########
4193	0.15	########	########	€ 716,754	€ 955,672	-€ 5,776,966	###########	###########	###########
4194	0.17	########	########	€ 786,071	#########	-€ 6,335,657	###########	###########	###########
4195	0.06	€ 99,110	########	€ 297,329	€ 396,438	-€ 2,396,440	-€ 4,792,881	-€ 7,189,321	-€ 9,585,761
4196	0.26	########	########	#########	#########	-€ 9,759,090	###########	###########	###########
4197	0.22	########	########	#########	#########	-€ 8,492,763	###########	###########	###########
4198	0.21	########	########	#########	#########	-€ 8,083,693	###########	###########	###########
4199	0.21	########	########	€ 987,979	#########	-€ 7,963,016	###########	###########	###########
4200	0.19	########	########	€ 906,411	#########	-€ 7,305,586	###########	###########	###########
4201	0.19	########	########	€ 918,426	#########	-€ 7,402,426	###########	###########	###########
4202	0.17	########	########	€ 800,603	#########	-€ 6,452,778	###########	###########	###########
4203	0.18	########	########	€ 868,873	#########	-€ 7,003,029	###########	###########	###########
4204	0.18	########	########	€ 834,751	#########	-€ 6,728,008	###########	###########	###########
4205	0.13	########	########	€ 635,620	€ 847,494	-€ 5,123,037	###########	###########	###########
4206	0.15	########	########	€ 706,560	€ 942,080	-€ 5,694,804	###########	###########	###########
4207	0.15	########	########	€ 692,895	€ 923,861	-€ 5,584,669	###########	###########	###########
4208	0.14	########	########	€ 676,334	€ 901,779	-€ 5,451,185	###########	###########	###########
4209	0.16	########	########	€ 763,602	#########	-€ 6,154,559	###########	###########	###########
4210	0.18	########	########	€ 870,723	#########	-€ 7,017,940	###########	###########	###########
4211	0.20	########	########	€ 924,730	#########	-€ 7,453,229	###########	###########	###########
4212	0.20	########	########	€ 965,940	#########	-€ 7,785,379	###########	###########	###########
4213	0.16	########	########	€ 746,544	€ 995,392	-€ 6,017,073	###########	###########	###########
4214	0.18	########	########	€ 865,184	#########	-€ 6,973,296	###########	###########	###########
4215	0.15	########	########	€ 685,662	€ 914,215	-€ 5,526,364	###########	###########	###########
4216	0.19	########	########	€ 895,116	#########	-€ 7,214,548	###########	###########	###########
4217	0.14	########	########	€ 657,471	€ 876,628	-€ 5,299,151	###########	###########	###########
4218	0.24	########	########	#########	#########	-€ 9,070,640	###########	###########	###########
4219	0.12	########	########	€ 546,192	€ 728,257	-€ 4,402,257	-€ 8,804,513	###########	###########
4220	0.19	########	########	€ 912,026	#########	-€ 7,350,841	###########	###########	###########
4221	0.14	########	########	€ 679,289	€ 905,719	-€ 5,475,003	###########	###########	###########
4222	0.15	########	########	€ 692,772	€ 923,696	-€ 5,583,677	###########	###########	###########
4223	0.17	########	########	€ 820,833	#########	-€ 6,615,836	###########	###########	###########
4224	0.10	########	########	€ 492,138	€ 656,184	-€ 3,966,583	-€ 7,933,167	###########	###########
4225	0.20	########	########	€ 946,784	#########	-€ 7,630,984	###########	###########	###########
4226	0.14	########	########	€ 647,511	€ 863,348	-€ 5,218,877	###########	###########	###########
4227	0.17	########	########	€ 814,796	#########	-€ 6,567,175	###########	###########	###########
4228	0.17	########	########	€ 800,575	#########	-€ 6,452,556	###########	###########	###########
4229	0.15	########	########	€ 728,497	€ 971,329	-€ 5,871,613	###########	###########	###########
4230	0.20	########	########	€ 946,293	#########	-€ 7,627,029	###########	###########	###########
4231	0.20	########	########	€ 944,060	#########	-€ 7,609,030	###########	###########	###########
4232	0.16	########	########	€ 758,913	#########	-€ 6,116,761	###########	###########	###########
4233	0.24	########	########	#########	#########	-€ 8,974,009	###########	###########	###########
4234	0.18	########	########	€ 834,973	#########	-€ 6,729,800	###########	###########	###########
4235	0.16	########	########	€ 736,176	€ 981,567	-€ 5,933,502	###########	###########	###########
4236	0.17	########	########	€ 797,618	#########	-€ 6,428,724	###########	###########	###########
4237	0.12	########	########	€ 545,179	€ 726,905	-€ 4,394,087	-€ 8,788,175	###########	###########
4238	0.18	########	########	€ 834,760	#########	-€ 6,728,083	###########	###########	###########
4239	0.15	########	########	€ 715,969	€ 954,625	-€ 5,770,636	###########	###########	###########
4240	0.15	########	########	€ 727,315	€ 969,753	-€ 5,862,083	###########	###########	###########
4241	0.21	########	########	€ 968,976	#########	-€ 7,809,851	###########	###########	###########
4242	0.16	########	########	€ 773,410	#########	-€ 6,233,605	###########	###########	###########
4243	0.14	########	########	€ 651,679	€ 868,905	-€ 5,252,467	###########	###########	###########
4244	0.11	########	########	€ 505,157	€ 673,542	-€ 4,071,513	-€ 8,143,027	###########	###########
4245	0.12	########	########	€ 558,839	€ 745,119	-€ 4,504,186	-€ 9,008,373	###########	###########
4246	0.13	########	########	€ 615,888	€ 821,183	-€ 4,963,992	-€ 9,927,985	###########	###########
4247	0.18	########	########	€ 839,804	#########	-€ 6,768,734	###########	###########	###########
4248	0.11	########	########	€ 515,530	€ 687,374	-€ 4,155,123	-€ 8,310,245	###########	###########
4249	0.17	########	########	€ 804,219	#########	-€ 6,481,925	###########	###########	###########
4250	0.18	########	########	€ 832,785	#########	-€ 6,712,166	###########	###########	###########
4251	0.22	########	########	#########	#########	-€ 8,444,557	###########	###########	###########
4252	0.18	########	########	€ 831,096	#########	-€ 6,698,554	###########	###########	###########
4253	0.18	########	########	€ 867,222	#########	-€ 6,989,726	###########	###########	###########
4254	0.23	########	########	#########	#########	-€ 8,860,569	###########	###########	###########
4255	0.11	########	########	€ 537,915	€ 717,220	-€ 4,335,543	-€ 8,671,086	###########	###########
4256	0.18	########	########	€ 845,715	#########	-€ 6,816,378	###########	###########	###########
4257	0.18	########	########	€ 855,302	#########	-€ 6,893,652	###########	###########	###########
4258	0.17	########	########	€ 793,098	#########	-€ 6,392,290	###########	###########	###########
4259	0.16	########	########	€ 769,046	#########	-€ 6,198,434	###########	###########	###########
4260	0.16	########	########	€ 760,126	#########	-€ 6,126,544	###########	###########	###########
4261	0.12	########	########	€ 551,103	€ 734,805	-€ 4,441,840	-€ 8,883,679	###########	###########
4262	0.15	########	########	€ 714,850	€ 953,133	-€ 5,761,617	###########	###########	###########
4263	0.18	########	########	€ 870,975	#########	-€ 7,019,971	###########	###########	###########
4264	0.18	########	########	€ 863,241	#########	-€ 6,957,637	###########	###########	###########
4265	0.13	########	########	€ 611,470	€ 815,293	-€ 4,928,387	-€ 9,856,775	###########	###########
4266	0.16	########	########	€ 744,256	€ 992,342	-€ 5,998,633	###########	###########	###########
4267	0.20	########	########	€ 930,749	#########	-€ 7,501,747	###########	###########	###########
4268	0.10	########	########	€ 455,924	€ 607,899	-€ 3,674,702	-€ 7,349,404	###########	###########
4269	0.20	########	########	€ 934,466	#########	-€ 7,531,700	###########	###########	###########
4270	0.20	########	########	€ 928,402	#########	-€ 7,482,829	###########	###########	###########
4271	0.21	########	########	€ 976,247	#########	-€ 7,868,455	###########	###########	###########
4272	0.21	########	########	€ 990,244	#########	-€ 7,981,269	###########	###########	###########
4273	0.13	########	########	€ 594,183	€ 792,244	-€ 4,789,055	-€ 9,578,110	###########	###########
4274	0.25	########	########	#########	#########	-€ 9,428,798	###########	###########	###########
4275	0.18	########	########	€ 862,380	#########	-€ 6,950,699	###########	###########	###########
4276	0.21	########	########	#########	#########	-€ 8,085,354	###########	###########	###########
4277	0.16	########	########	€ 734,375	€ 979,167	-€ 5,918,991	###########	###########	###########
4278	0.20	########	########	€ 946,778	#########	-€ 7,630,938	###########	###########	###########
4279	0.12	########	########	€ 565,994	€ 754,658	-€ 4,561,852	-€ 9,123,704	###########	###########
4280	0.12	########	########	€ 563,614	€ 751,486	-€ 4,542,676	-€ 9,085,352	###########	###########
4281	0.13	########	########	€ 624,437	€ 832,583	-€ 5,032,902	###########	###########	###########
4282	0.16	########	########	€ 777,113	#########	-€ 6,263,456	###########	###########	###########
4283	0.18	########	########	€ 868,571	#########	-€ 7,000,598	###########	###########	###########
4284	0.12	########	########	€ 580,499	€ 773,999	-€ 4,678,764	-€ 9,357,528	###########	###########
4285	0.24	########	########	#########	#########	-€ 9,155,668	###########	###########	###########
4286	0.22	########	########	#########	#########	-€ 8,319,443	###########	###########	###########
4287	0.15	########	########	€ 710,633	€ 947,510	-€ 5,727,630	###########	###########	###########
4288	0.11	########	########	€ 522,543	€ 696,723	-€ 4,211,641	-€ 8,423,282	###########	###########
4289	0.14	########	########	€ 684,038	€ 912,051	-€ 5,513,280	###########	###########	###########
4290	0.17	########	########	€ 810,967	#########	-€ 6,536,316	###########	###########	###########
4291	0.18	########	########	€ 863,158	#########	-€ 6,956,965	###########	###########	###########
4292	0.20	########	########	€ 938,503	#########	-€ 7,564,242	###########	###########	###########
4293	0.21	########	########	#########	#########	-€ 8,174,106	###########	###########	###########
4294	0.12	########	########	€ 571,783	€ 762,378	-€ 4,608,518	-€ 9,217,036	###########	###########
4295	0.23	########	########	#########	#########	-€ 8,878,414	###########	###########	###########
4296	0.20	########	########	€ 952,060	#########	-€ 7,673,508	###########	###########	###########

4297	0.13	########	########	€ 617,378	€ 823,171	-€ 4,976,005	-€ 9,952,011	###########	###########
4298	0.14	########	########	€ 678,479	€ 904,639	-€ 5,468,473	###########	###########	###########
4299	0.15	########	########	€ 727,227	€ 969,636	-€ 5,861,378	###########	###########	###########
4300	0.12	########	########	€ 544,285	€ 725,714	-€ 4,386,887	-€ 8,773,774	###########	###########
4301	0.22	########	########	#########	#########	-€ 8,217,880	###########	###########	###########
4302	0.11	########	########	€ 501,353	€ 668,470	-€ 4,040,853	-€ 8,081,707	###########	###########
4303	0.20	########	########	€ 965,385	#########	-€ 7,780,906	###########	###########	###########
4304	0.25	########	########	#########	#########	-€ 9,461,594	###########	###########	###########
4305	0.15	########	########	€ 726,699	€ 968,932	-€ 5,857,125	###########	###########	###########
4306	0.17	########	########	€ 816,868	#########	-€ 6,583,874	###########	###########	###########
4307	0.13	########	########	€ 610,721	€ 814,295	-€ 4,922,351	-€ 9,844,701	###########	###########
4308	0.10	########	########	€ 456,068	€ 608,091	-€ 3,675,867	-€ 7,351,734	###########	###########
4309	0.15	########	########	€ 705,750	€ 941,000	-€ 5,688,274	###########	###########	###########
4310	0.14	########	########	€ 660,967	€ 881,289	-€ 5,327,327	###########	###########	###########
4311	0.17	########	########	€ 792,062	#########	-€ 6,383,942	###########	###########	###########
4312	0.18	########	########	€ 859,281	#########	-€ 6,925,720	###########	###########	###########
4313	0.14	########	########	€ 682,921	€ 910,561	-€ 5,504,274	###########	###########	###########
4314	0.20	########	########	€ 961,995	#########	-€ 7,753,587	###########	###########	###########
4315	0.14	########	########	€ 676,336	€ 901,782	-€ 5,451,204	###########	###########	###########
4316	0.12	########	########	€ 564,036	€ 752,048	-€ 4,546,073	-€ 9,092,145	###########	###########
4317	0.15	########	########	€ 709,090	€ 945,453	-€ 5,715,195	###########	###########	###########
4318	0.20	########	########	€ 928,556	#########	-€ 7,484,067	###########	###########	###########
4319	0.14	########	########	€ 650,545	€ 867,393	-€ 5,243,327	###########	###########	###########
4320	0.13	########	########	€ 598,988	€ 798,650	-€ 4,827,782	-€ 9,655,564	###########	###########
4321	0.14	########	########	€ 644,191	€ 858,921	-€ 5,192,115	###########	###########	###########
4322	0.13	########	########	€ 636,776	€ 849,035	-€ 5,132,352	###########	###########	###########
4323	0.22	########	########	#########	#########	-€ 8,243,089	###########	###########	###########
4324	0.22	########	########	#########	#########	-€ 8,280,255	###########	###########	###########
4325	0.14	########	########	€ 656,184	€ 874,913	-€ 5,288,782	###########	###########	###########
4326	0.14	########	########	€ 667,127	€ 889,503	-€ 5,376,982	###########	###########	###########
4327	0.24	########	########	#########	#########	-€ 9,288,144	###########	###########	###########
4328	0.14	########	########	€ 642,907	€ 857,209	-€ 5,181,766	###########	###########	###########
4329	0.15	########	########	€ 723,839	€ 965,119	-€ 5,834,074	###########	###########	###########
4330	0.18	########	########	€ 856,723	#########	-€ 6,905,102	###########	###########	###########
4331	0.14	########	########	€ 668,999	€ 891,999	-€ 5,392,066	###########	###########	###########
4332	0.20	########	########	€ 934,151	#########	-€ 7,529,162	###########	###########	###########
4333	0.14	########	########	€ 682,459	€ 909,945	-€ 5,500,553	###########	###########	###########
4334	0.13	########	########	€ 634,968	€ 846,623	-€ 5,117,776	###########	###########	###########
4335	0.24	########	########	#########	#########	-€ 9,136,746	###########	###########	###########
4336	0.12	########	########	€ 579,152	€ 772,202	-€ 4,667,907	-€ 9,335,814	###########	###########
4337	0.16	########	########	€ 733,093	€ 977,457	-€ 5,908,653	###########	###########	###########
4338	0.21	########	########	#########	#########	-€ 8,182,559	###########	###########	###########
4339	0.13	########	########	€ 594,482	€ 792,643	-€ 4,791,466	-€ 9,582,932	###########	###########
4340	0.19	########	########	€ 908,115	#########	-€ 7,319,314	###########	###########	###########
4341	0.19	########	########	€ 915,594	#########	-€ 7,379,598	###########	###########	###########
4342	0.22	########	########	#########	#########	-€ 8,313,639	###########	###########	###########
4343	0.18	########	########	€ 843,481	#########	-€ 6,798,372	###########	###########	###########
4344	0.15	########	########	€ 720,513	€ 960,684	-€ 5,807,264	###########	###########	###########
4345	0.18	########	########	€ 831,517	#########	-€ 6,701,949	###########	###########	###########
4346	0.15	########	########	€ 692,144	€ 922,859	-€ 5,578,613	###########	###########	###########
4347	0.16	########	########	€ 773,439	#########	-€ 6,233,840	###########	###########	###########
4348	0.11	########	########	€ 525,832	€ 701,109	-€ 4,238,152	-€ 8,476,304	###########	###########
4349	0.16	########	########	€ 775,196	#########	-€ 6,248,004	###########	###########	###########
4350	0.15	########	########	€ 692,839	€ 923,785	-€ 5,584,213	###########	###########	###########
4351	0.12	########	########	€ 573,240	€ 764,320	-€ 4,620,257	-€ 9,240,513	###########	###########
4352	0.17	########	########	€ 793,803	#########	-€ 6,397,972	###########	###########	###########
4353	0.21	########	########	€ 986,012	#########	-€ 7,947,159	###########	###########	###########
4354	0.15	########	########	€ 687,444	€ 916,593	-€ 5,540,734	###########	###########	###########
4355	0.14	########	########	€ 650,884	€ 867,846	-€ 5,246,064	###########	###########	###########
4356	0.19	########	########	€ 894,994	#########	-€ 7,213,567	###########	###########	###########
4357	0.11	########	########	€ 496,704	€ 662,272	-€ 4,003,385	-€ 8,006,770	###########	###########
4358	0.18	########	########	€ 851,130	#########	-€ 6,860,022	###########	###########	###########
4359	0.14	########	########	€ 641,609	€ 855,479	-€ 5,171,304	###########	###########	###########
4360	0.14	########	########	€ 668,487	€ 891,316	-€ 5,387,941	###########	###########	###########
4361	0.24	########	########	#########	#########	-€ 9,028,834	###########	###########	###########
4362	0.21	########	########	€ 968,532	#########	-€ 7,806,269	###########	###########	###########
4363	0.22	########	########	#########	#########	-€ 8,254,358	###########	###########	###########
4364	0.09	########	########	€ 406,446	€ 541,927	-€ 3,275,911	-€ 6,551,821	-€ 9,827,732	###########
4365	0.15	########	########	€ 707,857	€ 943,809	-€ 5,705,254	###########	###########	###########
4366	0.22	########	########	#########	#########	-€ 8,278,908	###########	###########	###########
4367	0.22	########	########	#########	#########	-€ 8,264,582	###########	###########	###########
4368	0.19	########	########	€ 915,334	#########	-€ 7,377,498	###########	###########	###########
4369	0.20	########	########	€ 959,562	#########	-€ 7,733,973	###########	###########	###########
4370	0.15	########	########	€ 695,493	€ 927,325	-€ 5,605,609	###########	###########	###########
4371	0.13	########	########	€ 615,803	€ 821,071	-€ 4,963,312	-€ 9,926,625	###########	###########
4372	0.18	########	########	€ 844,925	#########	-€ 6,810,015	###########	###########	###########
4373	0.22	########	########	#########	#########	-€ 8,253,594	###########	###########	###########
4374	0.15	########	########	€ 725,858	€ 967,810	-€ 5,850,342	###########	###########	###########
4375	0.10	########	########	€ 480,925	€ 641,233	-€ 3,876,209	-€ 7,752,417	###########	###########
4376	0.15	########	########	€ 718,480	€ 957,974	-€ 5,790,882	###########	###########	###########
4377	0.17	########	########	€ 793,860	#########	-€ 6,398,430	###########	###########	###########
4378	0.13	########	########	€ 627,571	€ 836,761	-€ 5,058,160	###########	###########	###########
4379	0.18	########	########	€ 848,409	#########	-€ 6,838,094	###########	###########	###########
4380	0.18	########	########	€ 849,598	#########	-€ 6,847,680	###########	###########	###########
4381	0.13	########	########	€ 616,527	€ 822,036	-€ 4,969,148	-€ 9,938,296	###########	###########
4382	0.12	########	########	€ 577,672	€ 770,229	-€ 4,655,978	-€ 9,311,956	###########	###########
4383	0.19	########	########	€ 912,775	#########	-€ 7,356,873	###########	###########	###########
4384	0.18	########	########	€ 860,985	#########	-€ 6,939,450	###########	###########	###########
4385	0.22	########	########	#########	#########	-€ 8,394,714	###########	###########	###########
4386	0.17	########	########	€ 809,443	#########	-€ 6,524,034	###########	###########	###########
4387	0.20	########	########	€ 956,026	#########	-€ 7,705,474	###########	###########	###########
4388	0.19	########	########	€ 897,104	#########	-€ 7,230,569	###########	###########	###########
4389	0.18	########	########	€ 866,070	#########	-€ 6,980,436	###########	###########	###########
4390	0.10	########	########	€ 495,980	€ 661,307	-€ 3,997,551	-€ 7,995,102	###########	###########
4391	0.20	########	########	€ 936,984	#########	-€ 7,551,996	###########	###########	###########
4392	0.15	########	########	€ 709,152	€ 945,536	-€ 5,715,697	###########	###########	###########
4393	0.14	########	########	€ 678,281	€ 904,374	-€ 5,466,875	###########	###########	###########
4394	0.17	########	########	€ 792,464	#########	-€ 6,387,185	###########	###########	###########
4395	0.21	########	########	€ 996,871	#########	-€ 8,034,678	###########	###########	###########
4396	0.12	########	########	€ 549,335	€ 732,447	-€ 4,427,589	-€ 8,855,178	###########	###########
4397	0.17	########	########	€ 820,928	#########	-€ 6,616,599	###########	###########	###########
4398	0.14	########	########	€ 640,925	€ 854,567	-€ 5,165,791	###########	###########	###########
4399	0.19	########	########	€ 897,352	#########	-€ 7,232,566	###########	###########	###########
4400	0.20	########	########	€ 948,594	#########	-€ 7,645,575	###########	###########	###########
4401	0.16	########	########	€ 735,642	€ 980,855	-€ 5,929,199	###########	###########	###########
4402	0.17	########	########	€ 825,202	#########	-€ 6,651,043	###########	###########	###########
4403	0.17	########	########	€ 824,101	#########	-€ 6,642,176	###########	###########	###########
4404	0.17	########	########	€ 780,111	#########	-€ 6,287,613	###########	###########	###########
4405	0.15	########	########	€ 727,411	€ 969,881	-€ 5,862,862	###########	###########	###########
4406	0.13	########	########	€ 630,626	€ 840,834	-€ 5,082,781	###########	###########	###########
4407	0.16	########	########	€ 739,787	€ 986,382	-€ 5,962,607	###########	###########	###########
4408	0.14	########	########	€ 654,491	€ 872,655	-€ 5,275,134	###########	###########	###########
4409	0.12	########	########	€ 588,373	€ 784,497	-€ 4,742,226	-€ 9,484,453	###########	###########
4410	0.22	########	########	#########	#########	-€ 8,415,470	###########	###########	###########
4411	0.18	########	########	€ 831,761	#########	-€ 6,703,911	###########	###########	###########
4412	0.19	########	########	€ 912,404	#########	-€ 7,353,887	###########	###########	###########
4413	0.20	########	########	€ 945,222	#########	-€ 7,618,392	###########	###########	###########
4414	0.20	########	########	€ 952,653	#########	-€ 7,678,289	###########	###########	###########
4415	0.16	########	########	€ 737,790	€ 983,721	-€ 5,946,518	###########	###########	###########
4416	0.12	########	########	€ 550,341	€ 733,788	-€ 4,435,691	-€ 8,871,383	###########	###########
4417	0.23	########	########	#########	#########	-€ 8,845,222	###########	###########	###########
4418	0.18	########	########	€ 867,534	#########	-€ 6,992,237	###########	###########	###########
4419	0.19	########	########	€ 891,942	#########	-€ 7,188,961	###########	###########	###########
4420	0.18	########	########	€ 859,140	#########	-€ 6,924,581	###########	###########	###########
4421	0.12	########	########	€ 584,449	€ 779,266	-€ 4,710,602	-€ 9,421,204	###########	###########
4422	0.16	########	########	€ 766,038	#########	-€ 6,174,190	###########	###########	###########
4423	0.14	########	########	€ 668,729	€ 891,639	-€ 5,389,892	###########	###########	###########
4424	0.20	########	########	€ 925,976	#########	-€ 7,463,274	###########	###########	###########
4425	0.23	########	########	#########	#########	-€ 8,653,272	###########	###########	###########
4426	0.17	########	########	€ 811,207	#########	-€ 6,538,245	###########	###########	###########
4427	0.19	########	########	€ 913,831	#########	-€ 7,365,391	###########	###########	###########
4428	0.11	########	########	€ 503,438	€ 671,250	-€ 4,057,657	-€ 8,115,314	###########	###########
4429	0.16	########	########	€ 758,456	#########	-€ 6,113,084	###########	###########	###########
4430	0.19	########	########	€ 910,315	#########	-€ 7,337,053	###########	###########	###########
4431	0.14	########	########	€ 671,023	€ 894,697	-€ 5,408,378	###########	###########	###########
4432	0.13	########	########	€ 622,320	€ 829,761	-€ 5,015,841	###########	###########	###########
4433	0.15	########	########	€ 728,256	€ 971,008	-€ 5,869,672	###########	###########	###########
4434	0.21	########	########	€ 981,379	#########	-€ 7,909,818	###########	###########	###########
4435	0.19	########	########	€ 881,101	#########	-€ 7,101,586	###########	###########	###########
4436	0.15	########	########	€ 713,551	€ 951,402	-€ 5,751,154	###########	###########	###########
4437	0.18	########	########	€ 862,594	#########	-€ 6,952,420	###########	###########	###########
4438	0.16	########	########	€ 772,152	#########	-€ 6,223,472	###########	###########	###########
4439	0.17	########	########	€ 820,164	#########	-€ 6,610,441	###########	###########	###########
4440	0.17	########	########	€ 818,601	#########	-€ 6,597,842	###########	###########	###########
4441	0.34	########	########	#########	#########	###########	###########	###########	###########
4442	0.14	########	########	€ 647,611	€ 863,481	-€ 5,219,681	###########	###########	###########
4443	0.21	########	########	€ 978,967	#########	-€ 7,890,375	###########	###########	###########
4444	0.17	########	########	€ 804,509	#########	-€ 6,484,264	###########	###########	###########
4445	0.14	########	########	€ 684,720	€ 912,960	-€ 5,518,778	###########	###########	###########
4446	0.09	########	########	€ 408,394	€ 544,525	-€ 3,291,613	-€ 6,583,226	-€ 9,874,839	###########
4447	0.15	########	########	€ 685,270	€ 913,694	-€ 5,523,210	###########	###########	###########
4448	0.14	########	########	€ 674,848	€ 899,797	-€ 5,439,204	###########	###########	###########
4449	0.15	########	########	€ 702,847	€ 937,129	-€ 5,664,877	###########	###########	###########
4450	0.16	########	########	€ 745,005	€ 993,340	-€ 6,004,665	###########	###########	###########
4451	0.18	########	########	€ 853,862	#########	-€ 6,882,046	###########	###########	###########
4452	0.21	########	########	€ 970,093	#########	-€ 7,818,853	###########	###########	###########
4453	0.17	########	########	€ 809,623	#########	-€ 6,525,484	###########	###########	###########
4454	0.17	########	########	€ 823,292	#########	-€ 6,635,650	###########	###########	###########
4455	0.11	########	########	€ 508,797	€ 678,396	-€ 4,100,852	-€ 8,201,704	###########	###########
4456	0.14	########	########	€ 649,886	€ 866,515	-€ 5,238,017	###########	###########	###########
4457	0.17	########	########	€ 789,126	#########	-€ 6,360,280	###########	###########	###########
4458	0.14	########	########	€ 681,421	€ 908,561	-€ 5,492,185	###########	###########	###########
4459	0.15	########	########	€ 709,723	€ 946,297	-€ 5,720,294	###########	###########	###########
4460	0.15	########	########	€ 709,120	€ 945,494	-€ 5,715,439	###########	###########	###########
4461	0.17	########	########	€ 825,338	#########	-€ 6,652,143	###########	###########	###########
4462	0.19	########	########	€ 898,287	#########	-€ 7,240,108	###########	###########	###########
4463	0.14	########	########	€ 649,189	€ 865,586	-€ 5,232,403	###########	###########	###########
4464	0.16	########	########	€ 739,101	€ 985,468	-€ 5,957,080	###########	###########	###########
4465	0.17	########	########	€ 826,154	#########	-€ 6,658,723	###########	###########	###########
4466	0.12	########	########	€ 574,806	€ 766,408	-€ 4,632,882	-€ 9,265,763	###########	###########
4467	0.17	########	########	€ 808,198	#########	-€ 6,513,997	###########	###########	###########
4468	0.12	########	########	€ 554,190	€ 738,920	-€ 4,466,715	-€ 8,933,431	###########	###########

4469	0.14	########	########	€ 649,690	€ 866,254	-€ 5,236,441	###########	###########	###########
4470	0.13	########	########	€ 594,452	€ 792,603	-€ 4,791,228	-€ 9,582,455	###########	###########
4471	0.15	########	########	€ 695,925	€ 927,900	-€ 5,609,089	###########	###########	###########
4472	0.14	########	########	€ 671,821	€ 895,761	-€ 5,414,809	###########	###########	###########
4473	0.20	########	########	€ 934,879	#########	-€ 7,535,032	###########	###########	###########
4474	0.18	########	########	€ 848,971	#########	-€ 6,842,624	###########	###########	###########
4475	0.13	########	########	€ 595,869	€ 794,492	-€ 4,802,644	-€ 9,605,288	###########	###########
4476	0.16	########	########	€ 742,275	€ 989,700	-€ 5,982,662	###########	###########	###########
4477	0.10	########	########	€ 463,033	€ 617,378	-€ 3,732,002	-€ 7,464,004	###########	###########
4478	0.15	########	########	€ 716,829	€ 955,772	-€ 5,777,571	###########	###########	###########
4479	0.16	########	########	€ 736,217	€ 981,623	-€ 5,933,840	###########	###########	###########
4480	0.15	########	########	€ 686,147	€ 914,863	-€ 5,530,278	###########	###########	###########
4481	0.18	########	########	€ 840,391	#########	-€ 6,773,469	###########	###########	###########
4482	0.12	########	########	€ 576,888	€ 769,185	-€ 4,649,664	-€ 9,299,328	###########	###########
4483	0.18	########	########	€ 858,938	#########	-€ 6,922,957	###########	###########	###########
4484	0.12	########	########	€ 587,389	€ 783,186	-€ 4,734,300	-€ 9,468,599	###########	###########
4485	0.25	########	########	#########	#########	-€ 9,394,762	###########	###########	###########
4486	0.14	########	########	€ 638,649	€ 851,532	-€ 5,147,445	###########	###########	###########
4487	0.09	########	########	€ 426,849	€ 569,132	-€ 3,440,363	-€ 6,880,727	###########	###########
4488	0.14	########	########	€ 640,298	€ 853,730	-€ 5,160,737	###########	###########	###########
4489	0.21	########	########	€ 982,042	#########	-€ 7,915,164	###########	###########	###########
4490	0.18	########	########	€ 850,122	#########	-€ 6,851,896	###########	###########	###########
4491	0.12	########	########	€ 590,406	€ 787,208	-€ 4,758,614	-€ 9,517,229	###########	###########
4492	0.15	########	########	€ 699,177	€ 932,236	-€ 5,635,300	###########	###########	###########
4493	0.07	########	########	€ 321,065	€ 428,086	-€ 2,587,749	-€ 5,175,499	-€ 7,763,248	###########
4494	0.18	########	########	€ 849,365	#########	-€ 6,845,801	###########	###########	###########
4495	0.21	########	########	#########	#########	-€ 8,080,644	###########	###########	###########
4496	0.14	########	########	€ 645,575	€ 860,766	-€ 5,203,267	###########	###########	###########
4497	0.14	########	########	€ 668,181	€ 890,908	-€ 5,385,471	###########	###########	###########
4498	0.23	########	########	#########	#########	-€ 8,730,145	###########	###########	###########
4499	0.15	########	########	€ 688,786	€ 918,382	-€ 5,551,551	###########	###########	###########
4500	0.13	########	########	€ 591,372	€ 788,496	-€ 4,766,398	-€ 9,532,796	###########	###########
4501	0.15	########	########	€ 696,255	€ 928,340	-€ 5,611,747	###########	###########	###########
4502	0.16	########	########	€ 778,892	#########	-€ 6,277,793	###########	###########	###########
4503	0.13	########	########	€ 604,681	€ 806,242	-€ 4,873,671	-€ 9,747,342	###########	###########
4504	0.10	########	########	€ 491,867	€ 655,823	-€ 3,964,400	-€ 7,928,800	###########	###########
4505	0.11	########	########	€ 511,871	€ 682,495	-€ 4,125,633	-€ 8,251,266	###########	###########
4506	0.21	########	########	€ 997,899	#########	-€ 8,042,967	###########	###########	###########
4507	0.14	########	########	€ 650,331	€ 867,107	-€ 5,241,600	###########	###########	###########
4508	0.12	########	########	€ 578,401	€ 771,201	-€ 4,661,855	-€ 9,323,710	###########	###########
4509	0.20	########	########	€ 954,136	#########	-€ 7,690,240	###########	###########	###########
4510	0.14	########	########	€ 651,770	€ 869,027	-€ 5,253,205	###########	###########	###########
4511	0.16	########	########	€ 774,319	#########	-€ 6,240,935	###########	###########	###########
4512	0.19	########	########	€ 904,515	#########	-€ 7,290,304	###########	###########	###########
4513	0.13	########	########	€ 603,905	€ 805,207	-€ 4,867,414	-€ 9,734,828	###########	###########
4514	0.14	########	########	€ 642,505	€ 856,673	-€ 5,178,525	###########	###########	###########
4515	0.22	########	########	#########	#########	-€ 8,536,638	###########	###########	###########
4516	0.18	########	########	€ 862,994	#########	-€ 6,955,642	###########	###########	###########
4517	0.16	########	########	€ 767,385	#########	-€ 6,185,051	###########	###########	###########
4518	0.18	########	########	€ 854,356	#########	-€ 6,886,028	###########	###########	###########
4519	0.15	########	########	€ 720,295	€ 960,393	-€ 5,805,504	###########	###########	###########
4520	0.13	########	########	€ 614,039	€ 818,719	-€ 4,949,095	-€ 9,898,190	###########	###########
4521	0.12	########	########	€ 553,389	€ 737,852	-€ 4,460,259	-€ 8,920,518	###########	###########
4522	0.20	########	########	€ 924,865	#########	-€ 7,454,318	###########	###########	###########
4523	0.16	########	########	€ 737,630	€ 983,507	-€ 5,945,229	###########	###########	###########
4524	0.13	########	########	€ 621,773	€ 829,031	-€ 5,011,429	###########	###########	###########
4525	0.15	########	########	€ 713,894	€ 951,858	-€ 5,753,913	###########	###########	###########
4526	0.09	########	########	€ 439,051	€ 585,401	-€ 3,538,706	-€ 7,077,412	###########	###########
4527	0.16	########	########	€ 771,079	#########	-€ 6,214,821	###########	###########	###########
4528	0.20	########	########	€ 925,476	#########	-€ 7,459,242	###########	###########	###########
4529	0.15	########	########	€ 693,730	€ 924,974	-€ 5,591,398	###########	###########	###########
4530	0.19	########	########	€ 890,575	#########	-€ 7,177,945	###########	###########	###########
4531	0.15	########	########	€ 700,307	€ 933,743	-€ 5,644,407	###########	###########	###########
4532	0.10	########	########	€ 482,387	€ 643,183	-€ 3,887,991	-€ 7,775,983	###########	###########
4533	0.17	########	########	€ 808,674	#########	-€ 6,517,836	###########	###########	###########
4534	0.16	########	########	€ 772,351	#########	-€ 6,225,076	###########	###########	###########
4535	0.11	########	########	€ 525,150	€ 700,200	-€ 4,232,656	-€ 8,465,312	###########	###########
4536	0.19	########	########	€ 879,680	#########	-€ 7,090,136	###########	###########	###########
4537	0.15	########	########	€ 723,518	€ 964,691	-€ 5,831,487	###########	###########	###########
4538	0.16	########	########	€ 771,900	#########	-€ 6,221,438	###########	###########	###########
4539	0.09	########	########	€ 420,649	€ 560,865	-€ 3,390,389	-€ 6,780,777	###########	###########
4540	0.17	########	########	€ 782,391	#########	-€ 6,305,992	###########	###########	###########
4541	0.17	########	########	€ 786,989	#########	-€ 6,343,057	###########	###########	###########
4542	0.17	########	########	€ 808,050	#########	-€ 6,512,800	###########	###########	###########
4543	0.20	########	########	€ 954,300	#########	-€ 7,691,567	###########	###########	###########
4544	0.14	########	########	€ 684,464	€ 912,619	-€ 5,516,711	###########	###########	###########
4545	0.12	########	########	€ 565,996	€ 754,662	-€ 4,561,876	-€ 9,123,751	###########	###########
4546	0.11	########	########	€ 533,359	€ 711,146	-€ 4,298,824	-€ 8,597,648	###########	###########
4547	0.16	########	########	€ 747,951	€ 997,268	-€ 6,028,411	###########	###########	###########
4548	0.15	########	########	€ 704,542	€ 939,389	-€ 5,678,539	###########	###########	###########
4549	0.18	########	########	€ 832,373	#########	-€ 6,708,847	###########	###########	###########
4550	0.18	########	########	€ 830,645	#########	-€ 6,694,913	###########	###########	###########
4551	0.23	########	########	#########	#########	-€ 8,751,819	###########	###########	###########
4552	0.25	########	########	#########	#########	-€ 9,373,535	###########	###########	###########
4553	0.15	########	########	€ 712,626	€ 950,168	-€ 5,743,698	###########	###########	###########
4554	0.19	########	########	€ 885,755	#########	-€ 7,139,101	###########	###########	###########
4555	0.15	########	########	€ 707,102	€ 942,803	-€ 5,699,173	###########	###########	###########
4556	0.15	########	########	€ 701,250	€ 934,999	-€ 5,652,002	###########	###########	###########
4557	0.13	########	########	€ 609,123	€ 812,164	-€ 4,909,474	-€ 9,818,947	###########	###########
4558	0.15	########	########	€ 686,242	€ 914,989	-€ 5,531,042	###########	###########	###########
4559	0.11	########	########	€ 497,916	€ 663,888	-€ 4,013,155	-€ 8,026,310	###########	###########
4560	0.23	########	########	#########	#########	-€ 8,925,622	###########	###########	###########
4561	0.19	########	########	€ 896,316	#########	-€ 7,224,218	###########	###########	###########
4562	0.15	########	########	€ 690,999	€ 921,332	-€ 5,569,383	###########	###########	###########
4563	0.16	########	########	€ 733,771	€ 978,362	-€ 5,914,125	###########	###########	###########
4564	0.14	########	########	€ 662,723	€ 883,630	-€ 5,341,480	###########	###########	###########
4565	0.19	########	########	€ 899,411	#########	-€ 7,249,163	###########	###########	###########
4566	0.21	########	########	€ 971,693	#########	-€ 7,831,752	###########	###########	###########
4567	0.22	########	########	#########	#########	-€ 8,479,865	###########	###########	###########
4568	0.17	########	########	€ 788,278	#########	-€ 6,353,443	###########	###########	###########
4569	0.11	########	########	€ 525,667	€ 700,890	-€ 4,236,826	-€ 8,473,653	###########	###########
4570	0.20	########	########	€ 939,973	#########	-€ 7,576,088	###########	###########	###########
4571	0.20	########	########	€ 936,073	#########	-€ 7,544,658	###########	###########	###########
4572	0.16	########	########	€ 732,494	€ 976,658	-€ 5,903,825	###########	###########	###########
4573	0.18	########	########	€ 837,039	#########	-€ 6,746,449	###########	###########	###########
4574	0.13	########	########	€ 597,450	€ 796,600	-€ 4,815,389	-€ 9,630,778	###########	###########
4575	0.20	########	########	€ 944,356	#########	-€ 7,611,413	###########	###########	###########
4576	0.13	########	########	€ 610,842	€ 814,455	-€ 4,923,322	-€ 9,846,644	###########	###########
4577	0.13	########	########	€ 622,092	€ 829,456	-€ 5,013,999	###########	###########	###########
4578	0.13	########	########	€ 614,361	€ 819,149	-€ 4,951,692	-€ 9,903,384	###########	###########
4579	0.13	########	########	€ 622,081	€ 829,441	-€ 5,013,908	###########	###########	###########
4580	0.12	########	########	€ 553,320	€ 737,760	-€ 4,459,705	-€ 8,919,409	###########	###########
4581	0.13	########	########	€ 600,210	€ 800,280	-€ 4,837,631	-€ 9,675,262	###########	###########
4582	0.13	########	########	€ 614,693	€ 819,591	-€ 4,954,366	-€ 9,908,731	###########	###########
4583	0.19	########	########	€ 878,305	#########	-€ 7,079,049	###########	###########	###########
4584	0.17	########	########	€ 806,550	#########	-€ 6,500,714	###########	###########	###########
4585	0.12	########	########	€ 576,130	€ 768,173	-€ 4,643,547	-€ 9,287,094	###########	###########
4586	0.16	########	########	€ 752,405	#########	-€ 6,064,313	###########	###########	###########
4587	0.15	########	########	€ 723,155	€ 964,206	-€ 5,828,554	###########	###########	###########
4588	0.12	########	########	€ 559,696	€ 746,262	-€ 4,511,097	-€ 9,022,194	###########	###########
4589	0.13	########	########	€ 613,429	€ 817,905	-€ 4,944,177	-€ 9,888,353	###########	###########
4590	0.12	########	########	€ 589,104	€ 785,472	-€ 4,748,121	-€ 9,496,241	###########	###########
4591	0.14	########	########	€ 663,960	€ 885,280	-€ 5,351,451	###########	###########	###########
4592	0.26	########	########	#########	#########	-€ 9,790,830	###########	###########	###########
4593	0.17	########	########	€ 825,556	#########	-€ 6,653,900	###########	###########	###########
4594	0.14	########	########	€ 654,616	€ 872,822	-€ 5,276,142	###########	###########	###########
4595	0.16	########	########	€ 776,073	#########	-€ 6,255,068	###########	###########	###########
4596	0.20	########	########	€ 938,162	#########	-€ 7,561,494	###########	###########	###########
4597	0.19	########	########	€ 874,339	#########	-€ 7,047,086	###########	###########	###########
4598	0.18	########	########	€ 845,450	#########	-€ 6,814,245	###########	###########	###########
4599	0.13	########	########	€ 603,154	€ 804,205	-€ 4,861,358	-€ 9,722,716	###########	###########
4600	0.16	########	########	€ 739,618	€ 986,158	-€ 5,961,252	###########	###########	###########
4601	0.12	########	########	€ 584,269	€ 779,026	-€ 4,709,152	-€ 9,418,305	###########	###########
4602	0.09	########	########	€ 436,798	€ 582,398	-€ 3,520,551	-€ 7,041,102	###########	###########
4603	0.14	########	########	€ 665,356	€ 887,142	-€ 5,362,705	###########	###########	###########
4604	0.15	########	########	€ 704,307	€ 939,076	-€ 5,676,647	###########	###########	###########
4605	0.18	########	########	€ 868,838	#########	-€ 7,002,751	###########	###########	###########
4606	0.15	########	########	€ 719,481	€ 959,308	-€ 5,798,947	###########	###########	###########
4607	0.23	########	########	#########	#########	-€ 8,700,519	###########	###########	###########
4608	0.21	########	########	€ 999,434	#########	-€ 8,055,340	###########	###########	###########
4609	0.16	########	########	€ 778,509	#########	-€ 6,274,705	###########	###########	###########
4610	0.21	########	########	#########	#########	-€ 8,127,130	###########	###########	###########
4611	0.19	########	########	€ 892,647	#########	-€ 7,194,645	###########	###########	###########
4612	0.15	########	########	€ 709,828	€ 946,438	-€ 5,721,145	###########	###########	###########
4613	0.18	########	########	€ 870,541	#########	-€ 7,016,477	###########	###########	###########
4614	0.19	########	########	€ 885,137	#########	-€ 7,134,116	###########	###########	###########
4615	0.12	########	########	€ 555,002	€ 740,003	-€ 4,473,265	-€ 8,946,529	###########	###########
4616	0.12	########	########	€ 558,710	€ 744,946	-€ 4,503,144	-€ 9,006,288	###########	###########
4617	0.14	########	########	€ 660,483	€ 880,645	-€ 5,323,431	###########	###########	###########
4618	0.18	########	########	€ 862,638	#########	-€ 6,952,779	###########	###########	###########
4619	0.17	########	########	€ 792,948	#########	-€ 6,391,085	###########	###########	###########
4620	0.12	########	########	€ 548,606	€ 731,475	-€ 4,421,711	-€ 8,843,422	###########	###########
4621	0.17	########	########	€ 816,055	#########	-€ 6,577,326	###########	###########	###########
4622	0.16	########	########	€ 746,245	€ 994,993	-€ 6,014,660	###########	###########	###########
4623	0.19	########	########	€ 902,582	#########	-€ 7,274,724	###########	###########	###########
4624	0.22	########	########	#########	#########	-€ 8,515,746	###########	###########	###########
4625	0.09	########	########	€ 430,733	€ 574,311	-€ 3,471,666	-€ 6,943,331	###########	###########
4626	0.18	########	########	€ 840,285	#########	-€ 6,772,612	###########	###########	###########
4627	0.22	########	########	#########	#########	-€ 8,328,235	###########	###########	###########
4628	0.24	########	########	#########	#########	-€ 9,089,388	###########	###########	###########
4629	0.14	########	########	€ 679,262	€ 905,682	-€ 5,474,783	###########	###########	###########
4630	0.09	########	########	€ 439,358	€ 585,811	-€ 3,541,182	-€ 7,082,364	###########	###########
4631	0.15	########	########	€ 685,095	€ 913,461	-€ 5,521,801	###########	###########	###########
4632	0.16	########	########	€ 770,722	#########	-€ 6,211,942	###########	###########	###########
4633	0.20	########	########	€ 930,476	#########	-€ 7,499,547	###########	###########	###########
4634	0.15	########	########	€ 726,035	€ 968,047	-€ 5,851,773	###########	###########	###########
4635	0.11	########	########	€ 527,775	€ 703,700	-€ 4,253,811	-€ 8,507,623	###########	###########
4636	0.17	########	########	€ 814,701	#########	-€ 6,566,409	###########	###########	###########
4637	0.18	########	########	€ 859,904	#########	-€ 6,930,740	###########	###########	###########
4638	0.24	########	########	#########	#########	-€ 9,088,249	###########	###########	###########
4639	0.20	########	########	€ 955,201	#########	-€ 7,698,826	###########	###########	###########
4640	0.19	########	########	€ 916,975	#########	-€ 7,390,730	###########	###########	###########

4641	0.10	########	########	€ 492,635	€ 656,846	-€ 3,970,587	-€ 7,941,174	###########	###########
4642	0.14	########	########	€ 660,189	€ 880,251	-€ 5,321,055	###########	###########	###########
4643	0.18	########	########	€ 856,411	#########	-€ 6,902,588	###########	###########	###########
4644	0.11	########	########	€ 506,550	€ 675,400	-€ 4,082,743	-€ 8,165,485	###########	###########
4645	0.14	########	########	€ 646,276	€ 861,702	-€ 5,208,923	###########	###########	###########
4646	0.15	########	########	€ 721,675	€ 962,234	-€ 5,816,633	###########	###########	###########
4647	0.11	########	########	€ 541,820	€ 722,427	-€ 4,367,018	-€ 8,734,036	###########	###########
4648	0.17	########	########	€ 781,442	#########	-€ 6,298,349	###########	###########	###########
4649	0.21	########	########	€ 968,657	#########	-€ 7,807,277	###########	###########	###########
4650	0.11	########	########	€ 525,803	€ 701,071	-€ 4,237,923	-€ 8,475,846	###########	###########
4651	0.12	########	########	€ 567,487	€ 756,649	-€ 4,573,890	-€ 9,147,780	###########	###########
4652	0.18	########	########	€ 858,865	#########	-€ 6,922,367	###########	###########	###########
4653	0.17	########	########	€ 805,928	#########	-€ 6,495,700	###########	###########	###########
4654	0.22	########	########	#########	#########	-€ 8,357,717	###########	###########	###########
4655	0.15	########	########	€ 711,753	€ 949,005	-€ 5,736,663	###########	###########	###########
4656	0.09	########	########	€ 408,945	€ 545,260	-€ 3,296,058	-€ 6,592,115	-€ 9,888,173	###########
4657	0.17	########	########	€ 810,409	#########	-€ 6,531,813	###########	###########	###########
4658	0.16	########	########	€ 756,488	#########	-€ 6,097,217	###########	###########	###########
4659	0.18	########	########	€ 833,554	#########	-€ 6,718,361	###########	###########	###########
4660	0.22	########	########	#########	#########	-€ 8,292,651	###########	###########	###########
4661	0.16	########	########	€ 758,110	#########	-€ 6,110,295	###########	###########	###########
4662	0.14	########	########	€ 659,636	€ 879,515	-€ 5,316,604	###########	###########	###########
4663	0.21	########	########	€ 973,649	#########	-€ 7,847,511	###########	###########	###########
4664	0.16	########	########	€ 740,737	€ 987,649	-€ 5,970,263	###########	###########	###########
4665	0.21	########	########	€ 977,586	#########	-€ 7,879,244	###########	###########	###########
4666	0.25	########	########	#########	#########	-€ 9,415,848	###########	###########	###########
4667	0.11	########	########	€ 519,333	€ 692,444	-€ 4,185,772	-€ 8,371,543	###########	###########
4668	0.15	########	########	€ 710,453	€ 947,271	-€ 5,726,182	###########	###########	###########
4669	0.21	########	########	€ 989,049	#########	-€ 7,971,634	###########	###########	###########
4670	0.20	########	########	€ 963,291	#########	-€ 7,764,031	###########	###########	###########
4671	0.21	########	########	€ 974,058	#########	-€ 7,850,815	###########	###########	###########
4672	0.20	########	########	€ 951,836	#########	-€ 7,671,705	###########	###########	###########
4673	0.19	########	########	€ 900,553	#########	-€ 7,258,367	###########	###########	###########
4674	0.17	########	########	€ 786,282	#########	-€ 6,337,359	###########	###########	###########
4675	0.20	########	########	€ 927,816	#########	-€ 7,478,104	###########	###########	###########
4676	0.17	########	########	€ 800,345	#########	-€ 6,450,705	###########	###########	###########
4677	0.23	########	########	#########	#########	-€ 8,784,393	###########	###########	###########
4678	0.21	########	########	€ 991,501	#########	-€ 7,991,400	###########	###########	###########
4679	0.16	########	########	€ 774,539	#########	-€ 6,242,709	###########	###########	###########
4680	0.11	########	########	€ 508,995	€ 678,660	-€ 4,102,452	-€ 8,204,904	###########	###########
4681	0.15	########	########	€ 692,757	€ 923,676	-€ 5,583,552	###########	###########	###########
4682	0.17	########	########	€ 781,121	#########	-€ 6,295,757	###########	###########	###########
4683	0.15	########	########	€ 695,126	€ 926,835	-€ 5,602,646	###########	###########	###########
4684	0.17	########	########	€ 801,306	#########	-€ 6,458,448	###########	###########	###########
4685	0.14	########	########	€ 674,474	€ 899,298	-€ 5,436,191	###########	###########	###########
4686	0.26	########	########	#########	#########	-€ 9,923,413	###########	###########	###########
4687	0.15	########	########	€ 712,703	€ 950,270	-€ 5,744,314	###########	###########	###########
4688	0.21	########	########	#########	#########	-€ 8,128,875	###########	###########	###########
4689	0.15	########	########	€ 685,480	€ 913,973	-€ 5,524,901	###########	###########	###########
4690	0.16	########	########	€ 764,859	#########	-€ 6,164,692	###########	###########	###########
4691	0.19	########	########	€ 887,661	#########	-€ 7,154,462	###########	###########	###########
4692	0.15	########	########	€ 715,235	€ 953,647	-€ 5,764,724	###########	###########	###########
4693	0.21	########	########	€ 970,095	#########	-€ 7,818,873	###########	###########	###########
4694	0.12	########	########	€ 573,036	€ 764,048	-€ 4,618,614	-€ 9,237,229	###########	###########
4695	0.16	########	########	€ 741,021	€ 988,028	-€ 5,972,556	###########	###########	###########
4696	0.13	########	########	€ 608,139	€ 810,852	-€ 4,901,538	-€ 9,803,076	###########	###########
4697	0.17	########	########	€ 810,159	#########	-€ 6,529,797	###########	###########	###########
4698	0.23	########	########	#########	#########	-€ 8,617,531	###########	###########	###########
4699	0.17	########	########	€ 816,161	#########	-€ 6,578,176	###########	###########	###########
4700	0.14	########	########	€ 651,820	€ 869,093	-€ 5,253,603	###########	###########	###########
4701	0.16	########	########	€ 764,749	#########	-€ 6,163,805	###########	###########	###########
4702	0.11	########	########	€ 531,536	€ 708,714	-€ 4,284,124	-€ 8,568,247	###########	###########
4703	0.20	########	########	€ 949,333	#########	-€ 7,651,526	###########	###########	###########
4704	0.18	########	########	€ 840,833	#########	-€ 6,777,032	###########	###########	###########
4705	0.25	########	########	#########	#########	-€ 9,431,712	###########	###########	###########
4706	0.15	########	########	€ 725,023	€ 966,697	-€ 5,843,614	###########	###########	###########
4707	0.13	########	########	€ 605,196	€ 806,928	-€ 4,877,818	-€ 9,755,636	###########	###########
4708	0.17	########	########	€ 805,644	#########	-€ 6,493,413	###########	###########	###########
4709	0.22	########	########	#########	#########	-€ 8,443,804	###########	###########	###########
4710	0.12	########	########	€ 559,484	€ 745,979	-€ 4,509,389	-€ 9,018,778	###########	###########
4711	0.13	########	########	€ 636,834	€ 849,112	-€ 5,132,822	###########	###########	###########
4712	0.16	########	########	€ 742,094	€ 989,459	-€ 5,981,208	###########	###########	###########
4713	0.18	########	########	€ 859,573	#########	-€ 6,928,072	###########	###########	###########
4714	0.16	########	########	€ 749,112	€ 998,816	-€ 6,037,767	###########	###########	###########
4715	0.19	########	########	€ 887,033	#########	-€ 7,149,396	###########	###########	###########
4716	0.14	########	########	€ 642,897	€ 857,197	-€ 5,181,690	###########	###########	###########
4717	0.12	########	########	€ 590,013	€ 786,684	-€ 4,755,447	-€ 9,510,895	###########	###########
4718	0.20	########	########	€ 940,954	#########	-€ 7,583,999	###########	###########	###########
4719	0.14	########	########	€ 681,283	€ 908,378	-€ 5,491,075	###########	###########	###########
4720	0.15	########	########	€ 718,322	€ 957,763	-€ 5,789,605	###########	###########	###########
4721	0.12	########	########	€ 557,448	€ 743,264	-€ 4,492,974	-€ 8,985,947	###########	###########
4722	0.20	########	########	€ 934,144	#########	-€ 7,529,112	###########	###########	###########
4723	0.16	########	########	€ 765,969	#########	-€ 6,173,635	###########	###########	###########
4724	0.12	########	########	€ 563,740	€ 751,654	-€ 4,543,692	-€ 9,087,384	###########	###########
4725	0.14	########	########	€ 639,645	€ 852,860	-€ 5,155,473	###########	###########	###########
4726	0.20	########	########	€ 947,560	#########	-€ 7,637,236	###########	###########	###########
4727	0.17	########	########	€ 787,720	#########	-€ 6,348,946	###########	###########	###########
4728	0.18	########	########	€ 828,379	#########	-€ 6,676,652	###########	###########	###########
4729	0.10	########	########	€ 477,592	€ 636,790	-€ 3,849,348	-€ 7,698,695	###########	###########
4730	0.16	########	########	€ 758,266	#########	-€ 6,111,549	###########	###########	###########
4731	0.21	########	########	€ 978,264	#########	-€ 7,884,707	###########	###########	###########
4732	0.13	########	########	€ 622,792	€ 830,389	-€ 5,019,638	###########	###########	###########
4733	0.15	########	########	€ 716,958	€ 955,944	-€ 5,778,609	###########	###########	###########
4734	0.12	########	########	€ 568,992	€ 758,656	-€ 4,586,021	-€ 9,172,041	###########	###########
4735	0.11	########	########	€ 506,668	€ 675,557	-€ 4,083,691	-€ 8,167,382	###########	###########
4736	0.15	########	########	€ 700,185	€ 933,580	-€ 5,643,421	###########	###########	###########
4737	0.21	########	########	€ 998,007	#########	-€ 8,043,840	###########	###########	###########
4738	0.15	########	########	€ 709,935	€ 946,579	-€ 5,722,002	###########	###########	###########
4739	0.19	########	########	€ 878,383	#########	-€ 7,079,680	###########	###########	###########
4740	0.13	########	########	€ 618,932	€ 825,243	-€ 4,988,532	-€ 9,977,063	###########	###########
4741	0.17	########	########	€ 822,803	#########	-€ 6,631,708	###########	###########	###########
4742	0.17	########	########	€ 798,904	#########	-€ 6,439,084	###########	###########	###########
4743	0.26	########	########	#########	#########	-€ 9,733,894	###########	###########	###########
4744	0.16	########	########	€ 754,722	#########	-€ 6,082,985	###########	###########	###########
4745	0.15	########	########	€ 694,158	€ 925,543	-€ 5,594,841	###########	###########	###########
4746	0.14	########	########	€ 643,672	€ 858,230	-€ 5,187,935	###########	###########	###########
4747	0.15	########	########	€ 728,140	€ 970,854	-€ 5,868,740	###########	###########	###########
4748	0.15	########	########	€ 703,082	€ 937,443	-€ 5,666,775	###########	###########	###########
4749	0.16	########	########	€ 765,621	#########	-€ 6,170,833	###########	###########	###########
4750	0.12	########	########	€ 586,333	€ 781,777	-€ 4,725,783	-€ 9,451,567	###########	###########
4751	0.16	########	########	€ 733,930	€ 978,574	-€ 5,915,407	###########	###########	###########
4752	0.16	########	########	€ 778,694	#########	-€ 6,276,198	###########	###########	###########
4753	0.12	########	########	€ 588,602	€ 784,802	-€ 4,744,071	-€ 9,488,142	###########	###########
4754	0.16	########	########	€ 760,426	#########	-€ 6,128,962	###########	###########	###########
4755	0.17	########	########	€ 814,397	#########	-€ 6,563,960	###########	###########	###########
4756	0.13	########	########	€ 618,487	€ 824,649	-€ 4,984,944	-€ 9,969,887	###########	###########
4757	0.09	########	########	€ 420,165	€ 560,220	-€ 3,386,486	-€ 6,772,971	###########	###########
4758	0.13	########	########	€ 634,042	€ 845,389	-€ 5,110,314	###########	###########	###########
4759	0.21	########	########	€ 972,830	#########	-€ 7,840,913	###########	###########	###########
4760	0.20	########	########	€ 932,536	#########	-€ 7,516,145	###########	###########	###########
4761	0.14	########	########	€ 657,289	€ 876,385	-€ 5,297,685	###########	###########	###########
4762	0.20	########	########	€ 934,452	#########	-€ 7,531,591	###########	###########	###########
4763	0.19	########	########	€ 906,940	#########	-€ 7,309,844	###########	###########	###########
4764	0.14	########	########	€ 658,392	€ 877,856	-€ 5,306,574	###########	###########	###########
4765	0.15	########	########	€ 729,777	€ 973,035	-€ 5,881,927	###########	###########	###########
4766	0.14	########	########	€ 664,967	€ 886,623	-€ 5,359,568	###########	###########	###########
4767	0.13	########	########	€ 627,834	€ 837,111	-€ 5,060,276	###########	###########	###########
4768	0.14	########	########	€ 679,807	€ 906,409	-€ 5,479,176	###########	###########	###########
4769	0.13	########	########	€ 605,790	€ 807,720	-€ 4,882,609	-€ 9,765,218	###########	###########
4770	0.14	########	########	€ 670,089	€ 893,453	-€ 5,400,855	###########	###########	###########
4771	0.16	########	########	€ 748,366	€ 997,822	-€ 6,031,759	###########	###########	###########
4772	0.20	########	########	€ 950,736	#########	-€ 7,662,834	###########	###########	###########
4773	0.11	########	########	€ 499,734	€ 666,313	-€ 4,027,810	-€ 8,055,621	###########	###########
4774	0.16	########	########	€ 748,125	€ 997,500	-€ 6,029,811	###########	###########	###########
4775	0.15	########	########	€ 713,742	€ 951,655	-€ 5,752,687	###########	###########	###########
4776	0.15	########	########	€ 700,981	€ 934,641	-€ 5,649,838	###########	###########	###########
4777	0.12	########	########	€ 571,038	€ 761,384	-€ 4,602,510	-€ 9,205,020	###########	###########
4778	0.18	########	########	€ 868,595	#########	-€ 7,000,793	###########	###########	###########
4779	0.23	########	########	#########	#########	-€ 8,739,764	###########	###########	###########
4780	0.16	########	########	€ 752,915	#########	-€ 6,068,424	###########	###########	###########
4781	0.13	########	########	€ 634,770	€ 846,360	-€ 5,116,184	###########	###########	###########
4782	0.14	########	########	€ 651,529	€ 868,706	-€ 5,251,261	###########	###########	###########
4783	0.18	########	########	€ 854,489	#########	-€ 6,887,097	###########	###########	###########
4784	0.17	########	########	€ 810,223	#########	-€ 6,530,314	###########	###########	###########
4785	0.18	########	########	€ 861,875	#########	-€ 6,946,631	###########	###########	###########
4786	0.14	########	########	€ 667,043	€ 889,390	-€ 5,376,297	###########	###########	###########
4787	0.17	########	########	€ 789,165	#########	-€ 6,360,590	###########	###########	###########
4788	0.19	########	########	€ 911,645	#########	-€ 7,347,768	###########	###########	###########
4789	0.14	########	########	€ 648,591	€ 864,788	-€ 5,227,578	###########	###########	###########
4790	0.18	########	########	€ 852,197	#########	-€ 6,868,622	###########	###########	###########
4791	0.13	########	########	€ 615,472	€ 820,629	-€ 4,960,643	-€ 9,921,286	###########	###########
4792	0.22	########	########	#########	#########	-€ 8,487,336	###########	###########	###########
4793	0.20	########	########	€ 948,306	#########	-€ 7,643,249	###########	###########	###########
4794	0.20	########	########	€ 952,861	#########	-€ 7,679,968	###########	###########	###########
4795	0.16	########	########	€ 737,213	€ 982,951	-€ 5,941,864	###########	###########	###########
4796	0.18	########	########	€ 870,839	#########	-€ 7,018,873	###########	###########	###########
4797	0.18	########	########	€ 841,719	#########	-€ 6,784,169	###########	###########	###########
4798	0.11	########	########	€ 519,487	€ 692,649	-€ 4,187,013	-€ 8,374,025	###########	###########
4799	0.16	########	########	€ 761,434	#########	-€ 6,137,079	###########	###########	###########
4800	0.19	########	########	€ 882,578	#########	-€ 7,113,495	###########	###########	###########
4801	0.15	########	########	€ 700,564	€ 934,085	-€ 5,646,475	###########	###########	###########
4802	0.19	########	########	€ 889,952	#########	-€ 7,172,922	###########	###########	###########
4803	0.13	########	########	€ 616,504	€ 822,005	-€ 4,968,959	-€ 9,937,918	###########	###########
4804	0.11	########	########	€ 536,530	€ 715,374	-€ 4,324,381	-€ 8,648,762	###########	###########
4805	0.17	########	########	€ 797,202	#########	-€ 6,425,369	###########	###########	###########
4806	0.17	########	########	€ 789,366	#########	-€ 6,362,209	###########	###########	###########
4807	0.25	########	########	#########	#########	-€ 9,375,883	###########	###########	###########
4808	0.16	########	########	€ 747,533	€ 996,711	-€ 6,025,045	###########	###########	###########
4809	0.12	########	########	€ 586,170	€ 781,560	-€ 4,724,471	-€ 9,448,942	###########	###########
4810	0.16	########	########	€ 749,909	€ 999,879	-€ 6,044,196	###########	###########	###########
4811	0.23	########	########	#########	#########	-€ 8,780,266	###########	###########	###########
4812	0.12	########	########	€ 581,105	€ 774,807	-€ 4,683,650	-€ 9,367,300	###########	###########

4813	0.16	########	########	€ 750,546	#########	-€ 6,049,330	###########	###########	###########
4814	0.16	########	########	€ 747,264	€ 996,352	-€ 6,022,876	###########	###########	###########
4815	0.16	########	########	€ 738,735	€ 984,979	-€ 5,954,127	###########	###########	###########
4816	0.11	########	########	€ 514,488	€ 685,984	-€ 4,146,722	-€ 8,293,445	###########	###########
4817	0.25	########	########	#########	#########	-€ 9,701,814	###########	###########	###########
4818	0.24	########	########	#########	#########	-€ 9,072,368	###########	###########	###########
4819	0.21	########	########	€ 995,502	#########	-€ 8,023,644	###########	###########	###########
4820	0.24	########	########	#########	#########	-€ 8,977,609	###########	###########	###########
4821	0.18	########	########	€ 834,603	#########	-€ 6,726,821	###########	###########	###########
4822	0.13	########	########	€ 620,048	€ 826,730	-€ 4,997,522	-€ 9,995,045	###########	###########
4823	0.14	########	########	€ 644,906	€ 859,875	-€ 5,197,878	###########	###########	###########
4824	0.19	########	########	€ 921,074	#########	-€ 7,423,762	###########	###########	###########
4825	0.12	########	########	€ 588,381	€ 784,508	-€ 4,742,293	-€ 9,484,585	###########	###########
4826	0.14	########	########	€ 684,258	€ 912,344	-€ 5,515,054	###########	###########	###########
4827	0.18	########	########	€ 857,783	#########	-€ 6,913,648	###########	###########	###########
4828	0.24	########	########	#########	#########	-€ 9,007,776	###########	###########	###########
4829	0.18	########	########	€ 832,938	#########	-€ 6,713,394	###########	###########	###########
4830	0.14	########	########	€ 640,004	€ 853,338	-€ 5,158,366	###########	###########	###########
4831	0.11	########	########	€ 521,097	€ 694,796	-€ 4,199,993	-€ 8,399,986	###########	###########
4832	0.16	########	########	€ 751,024	#########	-€ 6,053,178	###########	###########	###########
4833	0.11	########	########	€ 514,104	€ 685,471	-€ 4,143,624	-€ 8,287,248	###########	###########
4834	0.17	########	########	€ 822,204	#########	-€ 6,626,880	###########	###########	###########
4835	0.15	########	########	€ 730,085	€ 973,447	-€ 5,884,412	###########	###########	###########
4836	0.12	########	########	€ 563,029	€ 750,705	-€ 4,537,959	-€ 9,075,918	###########	###########
4837	0.16	########	########	€ 774,813	#########	-€ 6,244,920	###########	###########	###########
4838	0.15	########	########	€ 728,828	€ 971,770	-€ 5,874,278	###########	###########	###########
4839	0.20	########	########	€ 948,226	#########	-€ 7,642,605	###########	###########	###########
4840	0.17	########	########	€ 788,633	#########	-€ 6,356,301	###########	###########	###########
4841	0.20	########	########	€ 925,878	#########	-€ 7,462,488	###########	###########	###########
4842	0.22	########	########	#########	#########	-€ 8,285,165	###########	###########	###########
4843	0.13	########	########	€ 631,946	€ 842,595	-€ 5,093,425	###########	###########	###########
4844	0.16	########	########	€ 746,631	€ 995,509	-€ 6,017,775	###########	###########	###########
4845	0.22	########	########	#########	#########	-€ 8,462,029	###########	###########	###########
4846	0.19	########	########	€ 916,696	#########	-€ 7,388,479	###########	###########	###########
4847	0.12	########	########	€ 576,635	€ 768,847	-€ 4,647,625	-€ 9,295,249	###########	###########
4848	0.13	########	########	€ 600,859	€ 801,145	-€ 4,842,864	-€ 9,685,727	###########	###########
4849	0.13	########	########	€ 625,225	€ 833,634	-€ 5,039,254	###########	###########	###########
4850	0.19	########	########	€ 884,296	#########	-€ 7,127,338	###########	###########	###########
4851	0.15	########	########	€ 727,496	€ 969,995	-€ 5,863,546	###########	###########	###########
4852	0.15	########	########	€ 700,442	€ 933,923	-€ 5,645,492	###########	###########	###########
4853	0.22	########	########	#########	#########	-€ 8,211,832	###########	###########	###########
4854	0.18	########	########	€ 856,809	#########	-€ 6,905,794	###########	###########	###########
4855	0.13	########	########	€ 609,004	€ 812,005	-€ 4,908,511	-€ 9,817,022	###########	###########
4856	0.26	########	########	#########	#########	-€ 9,843,869	###########	###########	###########
4857	0.16	########	########	€ 748,069	€ 997,425	-€ 6,029,358	###########	###########	###########
4858	0.14	########	########	€ 646,236	€ 861,648	-€ 5,208,600	###########	###########	###########
4859	0.20	########	########	€ 951,871	#########	-€ 7,671,987	###########	###########	###########
4860	0.15	########	########	€ 691,083	€ 921,443	-€ 5,570,057	###########	###########	###########
4861	0.17	########	########	€ 815,452	#########	-€ 6,572,464	###########	###########	###########
4862	0.15	########	########	€ 685,361	€ 913,815	-€ 5,523,944	###########	###########	###########
4863	0.17	########	########	€ 813,695	#########	-€ 6,558,300	###########	###########	###########
4864	0.14	########	########	€ 673,229	€ 897,639	-€ 5,426,160	###########	###########	###########
4865	0.17	########	########	€ 818,729	#########	-€ 6,598,871	###########	###########	###########
4866	0.18	########	########	€ 835,489	#########	-€ 6,733,962	###########	###########	###########
4867	0.16	########	########	€ 761,721	#########	-€ 6,139,395	###########	###########	###########
4868	0.18	########	########	€ 833,156	#########	-€ 6,715,152	###########	###########	###########
4869	0.17	########	########	€ 796,785	#########	-€ 6,422,006	###########	###########	###########
4870	0.23	########	########	#########	#########	-€ 8,859,458	###########	###########	###########
4871	0.19	########	########	€ 878,682	#########	-€ 7,082,087	###########	###########	###########
4872	0.11	########	########	€ 498,959	€ 665,278	-€ 4,021,557	-€ 8,043,114	###########	###########
4873	0.18	########	########	€ 854,636	#########	-€ 6,888,283	###########	###########	###########
4874	0.33	########	########	#########	#########	###########	###########	###########	###########
4875	0.10	########	########	€ 467,809	€ 623,745	-€ 3,770,490	-€ 7,540,981	###########	###########
4876	0.25	########	########	#########	#########	-€ 9,398,535	###########	###########	###########
4877	0.13	########	########	€ 620,505	€ 827,340	-€ 5,001,211	###########	###########	###########
4878	0.14	########	########	€ 676,216	€ 901,621	-€ 5,450,234	###########	###########	###########
4879	0.10	########	########	€ 455,331	€ 607,108	-€ 3,669,921	-€ 7,339,842	###########	###########
4880	0.20	########	########	€ 962,210	#########	-€ 7,755,319	###########	###########	###########
4881	0.23	########	########	#########	#########	-€ 8,611,048	###########	###########	###########
4882	0.20	########	########	€ 936,510	#########	-€ 7,548,181	###########	###########	###########
4883	0.21	########	########	€ 986,253	#########	-€ 7,949,101	###########	###########	###########
4884	0.16	########	########	€ 765,149	#########	-€ 6,167,023	###########	###########	###########
4885	0.21	########	########	€ 979,887	#########	-€ 7,897,795	###########	###########	###########
4886	0.12	########	########	€ 569,144	€ 758,858	-€ 4,587,241	-€ 9,174,482	###########	###########
4887	0.18	########	########	€ 831,314	#########	-€ 6,700,305	###########	###########	###########
4888	0.10	########	########	€ 493,650	€ 658,200	-€ 3,978,772	-€ 7,957,544	###########	###########
4889	0.18	########	########	€ 844,623	#########	-€ 6,807,581	###########	###########	###########
4890	0.07	########	########	€ 328,477	€ 437,970	-€ 2,647,494	-€ 5,294,987	-€ 7,942,481	###########
4891	0.13	########	########	€ 602,386	€ 803,181	-€ 4,855,172	-€ 9,710,343	###########	###########
4892	0.13	########	########	€ 602,079	€ 802,772	-€ 4,852,700	-€ 9,705,400	###########	###########
4893	0.18	########	########	€ 826,982	#########	-€ 6,665,390	###########	###########	###########
4894	0.16	########	########	€ 742,311	€ 989,748	-€ 5,982,952	###########	###########	###########
4895	0.16	########	########	€ 737,533	€ 983,377	-€ 5,944,444	###########	###########	###########
4896	0.20	########	########	€ 942,690	#########	-€ 7,597,986	###########	###########	###########
4897	0.11	########	########	€ 501,754	€ 669,005	-€ 4,044,086	-€ 8,088,173	###########	###########
4898	0.21	########	########	€ 994,719	#########	-€ 8,017,333	###########	###########	###########
4899	0.20	########	########	€ 923,057	#########	-€ 7,439,747	###########	###########	###########
4900	0.21	########	########	€ 974,482	#########	-€ 7,854,226	###########	###########	###########
4901	0.15	########	########	€ 703,566	€ 938,088	-€ 5,670,674	###########	###########	###########
4902	0.13	########	########	€ 618,836	€ 825,115	-€ 4,987,756	-€ 9,975,513	###########	###########
4903	0.16	########	########	€ 743,000	€ 990,666	-€ 5,988,505	###########	###########	###########
4904	0.16	########	########	€ 742,986	€ 990,648	-€ 5,988,396	###########	###########	###########
4905	0.16	########	########	€ 746,810	€ 995,747	-€ 6,019,217	###########	###########	###########
4906	0.14	########	########	€ 656,959	€ 875,946	-€ 5,295,028	###########	###########	###########
4907	0.20	########	########	€ 926,800	#########	-€ 7,469,914	###########	###########	###########
4908	0.21	########	########	€ 972,297	#########	-€ 7,836,620	###########	###########	###########
4909	0.11	########	########	€ 532,772	€ 710,362	-€ 4,294,086	-€ 8,588,171	###########	###########
4910	0.16	########	########	€ 766,509	#########	-€ 6,177,989	###########	###########	###########
4911	0.13	########	########	€ 596,007	€ 794,676	-€ 4,803,758	-€ 9,607,516	###########	###########
4912	0.24	########	########	#########	#########	-€ 9,221,978	###########	###########	###########
4913	0.21	########	########	#########	#########	-€ 8,100,564	###########	###########	###########
4914	0.21	########	########	€ 977,791	#########	-€ 7,880,897	###########	###########	###########
4915	0.19	########	########	€ 917,038	#########	-€ 7,391,234	###########	###########	###########
4916	0.15	########	########	€ 728,749	€ 971,666	-€ 5,873,648	###########	###########	###########
4917	0.16	########	########	€ 772,814	#########	-€ 6,228,806	###########	###########	###########
4918	0.18	########	########	€ 859,804	#########	-€ 6,929,933	###########	###########	###########
4919	0.13	########	########	€ 622,047	€ 829,396	-€ 5,013,639	###########	###########	###########
4920	0.14	########	########	€ 662,321	€ 883,095	-€ 5,338,241	###########	###########	###########
4921	0.09	########	########	€ 435,749	€ 580,999	-€ 3,512,096	-€ 7,024,192	###########	###########
4922	0.12	########	########	€ 561,341	€ 748,454	-€ 4,524,351	-€ 9,048,703	###########	###########
4923	0.19	########	########	€ 880,443	#########	-€ 7,096,287	###########	###########	###########
4924	0.13	########	########	€ 592,194	€ 789,592	-€ 4,773,028	-€ 9,546,056	###########	###########
4925	0.19	########	########	€ 902,922	#########	-€ 7,277,458	###########	###########	###########
4926	0.24	########	########	#########	#########	-€ 9,116,894	###########	###########	###########
4927	0.15	########	########	€ 722,185	€ 962,913	-€ 5,820,738	###########	###########	###########
4928	0.09	########	########	€ 413,485	€ 551,313	-€ 3,332,648	-€ 6,665,295	-€ 9,997,943	###########
4929	0.16	########	########	€ 778,079	#########	-€ 6,271,243	###########	###########	###########
4930	0.14	########	########	€ 669,511	€ 892,682	-€ 5,396,194	###########	###########	###########
4931	0.22	########	########	#########	#########	-€ 8,243,782	###########	###########	###########
4932	0.08	########	########	€ 398,249	€ 530,998	-€ 3,209,845	-€ 6,419,689	-€ 9,629,534	###########
4933	0.13	########	########	€ 593,374	€ 791,166	-€ 4,782,537	-€ 9,565,074	###########	###########
4934	0.19	########	########	€ 900,829	#########	-€ 7,260,595	###########	###########	###########
4935	0.10	########	########	€ 461,472	€ 615,296	-€ 3,719,417	-€ 7,438,835	###########	###########
4936	0.16	########	########	€ 774,375	#########	-€ 6,241,383	###########	###########	###########
4937	0.17	########	########	€ 801,424	#########	-€ 6,459,397	###########	###########	###########
4938	0.15	########	########	€ 702,739	€ 936,985	-€ 5,664,004	###########	###########	###########
4939	0.19	########	########	€ 882,937	#########	-€ 7,116,386	###########	###########	###########
4940	0.17	########	########	€ 816,835	#########	-€ 6,583,608	###########	###########	###########
4941	0.15	########	########	€ 689,905	€ 919,874	-€ 5,560,569	###########	###########	###########
4942	0.16	########	########	€ 745,636	€ 994,181	-€ 6,009,752	###########	###########	###########
4943	0.14	########	########	€ 653,665	€ 871,553	-€ 5,268,475	###########	###########	###########
4944	0.17	########	########	€ 782,132	#########	-€ 6,303,904	###########	###########	###########
4945	0.14	########	########	€ 640,770	€ 854,360	-€ 5,164,543	###########	###########	###########
4946	0.22	########	########	#########	#########	-€ 8,449,084	###########	###########	###########
4947	0.09	########	########	€ 445,388	€ 593,850	-€ 3,589,781	-€ 7,179,563	###########	###########
4948	0.13	########	########	€ 612,991	€ 817,321	-€ 4,940,645	-€ 9,881,289	###########	###########
4949	0.17	########	########	€ 784,574	#########	-€ 6,323,587	###########	###########	###########
4950	0.10	########	########	€ 478,682	€ 638,242	-€ 3,858,126	-€ 7,716,252	###########	###########
4951	0.22	########	########	#########	#########	-€ 8,509,379	###########	###########	###########
4952	0.17	########	########	€ 814,091	#########	-€ 6,561,493	###########	###########	###########
4953	0.14	########	########	€ 682,508	€ 910,011	-€ 5,500,946	###########	###########	###########
4954	0.13	########	########	€ 619,040	€ 825,387	-€ 4,989,405	-€ 9,978,810	###########	###########
4955	0.18	########	########	€ 841,125	#########	-€ 6,779,383	###########	###########	###########
4956	0.18	########	########	€ 843,993	#########	-€ 6,802,503	###########	###########	###########
4957	0.12	########	########	€ 567,213	€ 756,284	-€ 4,571,680	-€ 9,143,360	###########	###########
4958	0.11	########	########	€ 533,940	€ 711,920	-€ 4,303,505	-€ 8,607,010	###########	###########
4959	0.18	########	########	€ 869,209	#########	-€ 7,005,737	###########	###########	###########
4960	0.16	########	########	€ 752,064	#########	-€ 6,061,564	###########	###########	###########
4961	0.15	########	########	€ 715,904	€ 954,538	-€ 5,770,113	###########	###########	###########
4962	0.14	########	########	€ 640,811	€ 854,415	-€ 5,164,872	###########	###########	###########
4963	0.19	########	########	€ 898,378	#########	-€ 7,240,837	###########	###########	###########
4964	0.17	########	########	€ 791,109	#########	-€ 6,376,262	###########	###########	###########
4965	0.20	########	########	€ 924,607	#########	-€ 7,452,242	###########	###########	###########
4966	0.15	########	########	€ 699,805	€ 933,073	-€ 5,640,356	###########	###########	###########
4967	0.12	########	########	€ 553,795	€ 738,393	-€ 4,463,533	-€ 8,927,067	###########	###########
4968	0.13	########	########	€ 596,959	€ 795,945	-€ 4,811,430	-€ 9,622,859	###########	###########
4969	0.14	########	########	€ 684,193	€ 912,258	-€ 5,514,530	###########	###########	###########
4970	0.16	########	########	€ 740,042	€ 986,722	-€ 5,964,662	###########	###########	###########
4971	0.27	########	########	#########	#########	###########	###########	###########	###########
4972	0.15	########	########	€ 718,542	€ 958,056	-€ 5,791,376	###########	###########	###########
4973	0.31	########	########	#########	#########	###########	###########	###########	###########
4974	0.12	########	########	€ 583,485	€ 777,980	-€ 4,702,833	-€ 9,405,666	###########	###########
4975	0.19	########	########	€ 896,639	#########	-€ 7,226,820	###########	###########	###########
4976	0.13	########	########	€ 625,852	€ 834,469	-€ 5,044,305	###########	###########	###########
4977	0.15	########	########	€ 727,318	€ 969,757	-€ 5,862,108	###########	###########	###########
4978	0.15	########	########	€ 690,616	€ 920,821	-€ 5,566,297	###########	###########	###########
4979	0.09	########	########	€ 432,560	€ 576,747	-€ 3,486,393	-€ 6,972,785	###########	###########
4980	0.13	########	########	€ 619,328	€ 825,771	-€ 4,991,725	-€ 9,983,449	###########	###########
4981	0.20	########	########	€ 925,259	#########	-€ 7,457,494	###########	###########	###########
4982	0.16	########	########	€ 750,629	#########	-€ 6,049,994	###########	###########	###########
4983	0.16	########	########	€ 734,485	€ 979,314	-€ 5,919,878	###########	###########	###########
4984	0.16	########	########	€ 739,796	€ 986,395	-€ 5,962,682	###########	###########	###########

4985	0.19	########	########	€ 917,510	#########	-€ 7,395,041	###########	###########	###########
4986	0.17	########	########	€ 809,302	#########	-€ 6,522,893	###########	###########	###########
4987	0.20	########	########	€ 932,391	#########	-€ 7,514,976	###########	###########	###########
4988	0.19	########	########	€ 902,797	#########	-€ 7,276,454	###########	###########	###########
4989	0.15	########	########	€ 709,413	€ 945,884	-€ 5,717,801	###########	###########	###########
4990	0.16	########	########	€ 736,092	€ 981,456	-€ 5,932,829	###########	###########	###########
4991	0.17	########	########	€ 811,933	#########	-€ 6,544,100	###########	###########	###########
4992	0.14	########	########	€ 672,439	€ 896,585	-€ 5,419,791	###########	###########	###########
4993	0.21	########	########	€ 992,007	#########	-€ 7,995,480	###########	###########	###########
4994	0.21	########	########	#########	#########	-€ 8,095,805	###########	###########	###########
4995	0.15	########	########	€ 726,064	€ 968,085	-€ 5,852,001	###########	###########	###########
4996	0.13	########	########	€ 591,223	€ 788,298	-€ 4,765,202	-€ 9,530,405	###########	###########
4997	0.12	########	########	€ 573,996	€ 765,328	-€ 4,626,349	-€ 9,252,699	###########	###########
4998	0.16	########	########	€ 762,021	#########	-€ 6,141,818	###########	###########	###########
4999	0.15	########	########	€ 705,483	€ 940,643	-€ 5,686,119	###########	###########	###########
5000	0.12	########	########	€ 547,945	€ 730,594	-€ 4,416,384	-€ 8,832,768	###########	###########

Costing tool

© Amsterdam UMC en Vrije Universiteit Amsterdam, 2021

**Estimated costs per year (Prospective study)**	
**Variable costs**	
**Reassuring and non-reassuring aCTG**	€ 135,103
**Fixed costs**	
**Total training costs**	€ 13,800
**Total equipment costs**	€ 204,000
**Total quality assessment costs**	€ 70,838
**Total fixed costs**	€ 288,638
**Actual costs per year**	€ 423,741
**Reimbursed costs per year**	€ 316,400
**Difference between reimbursed and actual costs**	-€ 107,341
**Break-even point**	1799
**Fixed costs for the total number of estimated aCTGs in the Netherlands**	€ 4,588,026

Number of aCTGs	Actual costs per year	Reimbursed costs per year
0	€ 288,638	€ 0
25	€ 291,627	€ 7,000
50	€ 294,616	€ 14,000
75	€ 297,605	€ 21,000
100	€ 300,594	€ 28,000
125	€ 303,583	€ 35,000
150	€ 306,572	€ 42,000
175	€ 309,561	€ 49,000
200	€ 312,550	€ 56,000
225	€ 315,539	€ 63,000
250	€ 318,528	€ 70,000
275	€ 321,517	€ 77,000
300	€ 324,506	€ 84,000
325	€ 327,495	€ 91,000
350	€ 330,484	€ 98,000
375	€ 333,473	€ 105,000
400	€ 336,462	€ 112,000
425	€ 339,451	€ 119,000
450	€ 342,440	€ 126,000
475	€ 345,429	€ 133,000
500	€ 348,418	€ 140,000
525	€ 351,407	€ 147,000
550	€ 354,396	€ 154,000
575	€ 357,385	€ 161,000
600	€ 360,374	€ 168,000
625	€ 363,363	€ 175,000
650	€ 366,352	€ 182,000
675	€ 369,341	€ 189,000
700	€ 372,330	€ 196,000
725	€ 375,319	€ 203,000
750	€ 378,308	€ 210,000
775	€ 381,297	€ 217,000
800	€ 384,286	€ 224,000
825	€ 387,275	€ 231,000
850	€ 390,264	€ 238,000
875	€ 393,253	€ 245,000
900	€ 396,242	€ 252,000
925	€ 399,231	€ 259,000
950	€ 402,220	€ 266,000
975	€ 405,209	€ 273,000
1000	€ 408,198	€ 280,000
1025	€ 411,187	€ 287,000
1050	€ 414,176	€ 294,000
1075	€ 417,165	€ 301,000
1100	€ 420,154	€ 308,000
1125	€ 423,143	€ 315,000
1150	€ 426,132	€ 322,000
1175	€ 429,121	€ 329,000
1200	€ 432,110	€ 336,000
1225	€ 435,099	€ 343,000
1250	€ 438,088	€ 350,000
1275	€ 441,077	€ 357,000
1300	€ 444,066	€ 364,000
1325	€ 447,055	€ 371,000
1350	€ 450,044	€ 378,000
1375	€ 453,033	€ 385,000
1400	€ 456,022	€ 392,000
1425	€ 459,011	€ 399,000
1450	€ 462,000	€ 406,000
1475	€ 464,989	€ 413,000
1500	€ 467,978	€ 420,000
1525	€ 470,967	€ 427,000
1550	€ 473,956	€ 434,000
1575	€ 476,945	€ 441,000
1600	€ 479,934	€ 448,000
1625	€ 482,923	€ 455,000
1650	€ 485,912	€ 462,000
1675	€ 488,901	€ 469,000
1700	€ 491,890	€ 476,000
1725	€ 494,879	€ 483,000
1750	€ 497,868	€ 490,000
1775	€ 500,857	€ 497,000
1800	€ 503,846	€ 504,000
1825	€ 506,835	€ 511,000
1850	€ 509,824	€ 518,000
1875	€ 512,813	€ 525,000
1900	€ 515,802	€ 532,000
1925	€ 518,791	€ 539,000
1950	€ 521,780	€ 546,000
1975	€ 524,769	€ 553,000
2000	€ 527,758	€ 560,000

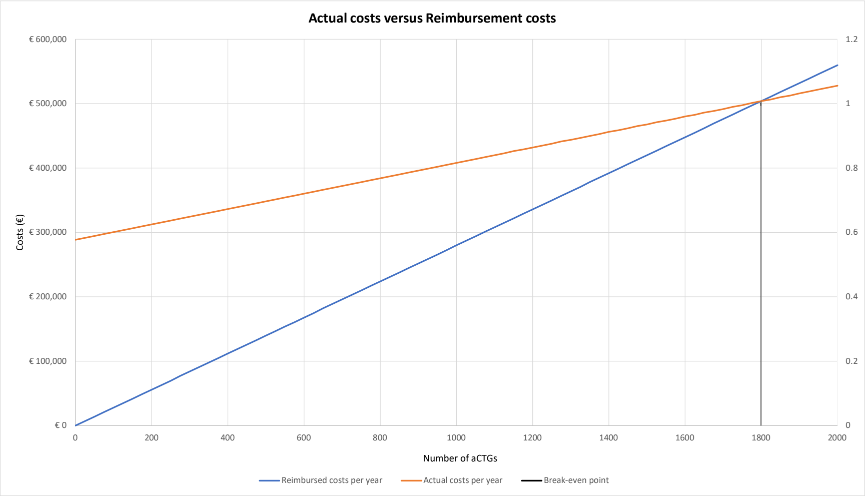

Supplement: online supplemental file 1 [file bmjoq-2023-002578supp001.pdf]
